# Rhodium-Catalyzed Synthesis of 2,3-Disubstituted Indoles from β,β-Disubstituted Stryryl Azides**

**DOI:** 10.1002/anie.201006917

**Published:** 2011-01-14

**Authors:** Ke Sun, Sheng Liu, Patryk M Bec, Tom G Driver

**Affiliations:** Department of Chemistry, University of Illinois at Chicago845 W. Taylor St., Chicago (USA), Fax: (+1) 312-996-0431

**Keywords:** 1,2-shift, azides, indoles, nitrenes, rhodium complexes

**Scheme 1:**
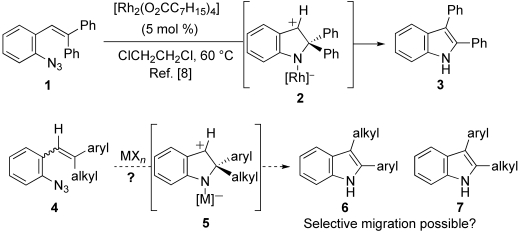
Potential for selective 2,3-disubstituted indole formation.

Transition metal-catalyzed migratorial processes that form new carbon–carbon bonds can enable the formation of complex products from readily accessible, simple starting materials. Controlling the selectivity of the migration step is critical to the success of these transformations.[1] Sequential reaction processes that involve metal nitrenes are rare despite their electrophilicity,[2] which enables reaction with carbon–hydrogen bonds or olefins.[3–6] Our mechanistic study of rhodium(II)-catalyzed carbazole formation from biaryl azides which suggested that C–N bond formation preceded C–H bond cleavage through a 4π-electron–5-atom electrocyclization.[7] Consequently, we anticipated that substrates lacking functionalizable C–H bonds might participate in a migratorial process where a new C–C bond is formed in addition to the C–N bond. In support of this hypothesis, rhodium octanoate catalyzed the conversion of β,β-diphenylstryryl azide **1** to 2,3-diphenylindole **3** (Scheme 1).[8] This result, however, does not indicate whether this process can be rendered selective for styryl azides **4** that contain two different β-substituents to form 2,3-disubstituted indoles. Because these N-heterocycles are important pharmaceutical scaffolds,[9] new methods, which streamline their synthesis, remain an ongoing goal.[10, 11] Herein, we report our initial studies that resulted in the development of a general method to form 2,3-disubstituted indoles—as single regioisomers—from readily available β,β-disubstituted stryryl azides.

The effect of transition metal complexes on the desired migration was investigated using a mixture of the *E*- and *Z*-isomer of β,β-disubstituted aryl azide **8** (Table 1). This azide is readily accessible in two steps from commercially available 2-nitrobenzaldehyde.[12] Examination of a range of dirhodium(II) complexes revealed that selective formation of **9** was obtained using with [Rh_2_(O_2_CC_3_F_7_)_4_],[8, 13] [Rh_2_(O_2_CC_7_H_15_)_4_], or [Rh_2_(esp)_2_][14] (Table 1, entries 1–7).[15] Importantly, both the *E*- and *Z*-isomer of **8** were converted to indole **9** revealing that the selectivity of the reaction did not depend on the stereochemistry of the starting material. Other rhodium carboxylate complexes provided attenuated selectivities or reduced yields. Other transition metal complexes, such as [(cod)Ir(OMe)_2_],[16] [Co(tpp)],[17] RuCl_3_,[18] or copper salts,[19] known to decompose azides or π-Lewis acids,[20] did not promote indole formation (Table 1, entries 8–13). Consequently, the reaction conditions were further optimized using rhodium hexaflourobutyrate, and incomplete conversions were observed when either the catalyst loading or the reaction temperature was lowered (<5 mol %; <70 °C). The optimal solvent was found to be either toluene or dichloroethane. Purification proved to be facile: analytically pure indole was obtained by filtering the reaction mixture through a pipette of alumina.

**Table 1:** Development of optimal conditions for indole formation

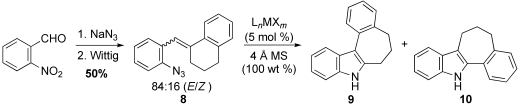				
Entry	L_*n*_MX_*m*_[a]	*T* [°C]	Yield [%][b]	**9**:**10**[c]
1	[Rh_2_(O_2_CCH_3_)_4_]	70	8	–
2	[Rh_2_(O_2_CC_7_H_15_)_4_]	70	93	96:4
3	[Rh_2_(esp)_2_]	70	98	98:2
4	[Rh_2_(O_2_CCF_3_)_4_]	70	86	99:1
5	[Rh_2_(O_2_CC_3_F_7_)_4_]	70	95	100:0
6[d]	[Rh_2_(O_2_CC_3_F_7_)_4_]	70	80[e]	100:0
7	[{(cod)Ir(OMe)}_2_]	70	0	–
8	[Co(tpp)]	80	0	–
9[f]	RuCl_3_⋅*n* H_2_O	65	trace	–
10	Cu(OTf)_2_	70	0	–
11	AgOTf	65	0	–
12	AuCl	65	0	–

[a] esp=α,α,α′,α′-tetramethyl-1,3-benzenedipropionate; cod=cyclooctadiene; tpp=tetraphenylporphyrin.

[b] Yield after Al_2_O_3_ chromatography.

[c] As determined by using ^1^H NMR spectroscopy.

[d] 3 mol % catalyst.

[e] 10 % aryl azide remained.

[f] No molecular sieve added.

Using these optimized conditions, the scope and limitations of the rhodium(II)-catalyzed formation of 2,3-disubstituted indoles from β,β-disubstituted stryryl azides was examined (Table 2). In every example, only aryl group migration was observed even if the electronic nature of the aryl azide moiety was modulated. High yields were observed with electron-donating substituents such as methoxide (Table 2, entries 1 and 2). Electron-withdrawing groups also did not lower the reaction yield or migration selectivity (Table 2, entries 3–8). Among these, azides bearing potentially reactive bromides, esters, or sulfones were competent substrates in our process. The reaction was also not sensitive to the steric nature around the azide: nearly quantitative yield of **12 a** was observed with **11 a**, which contained two *ortho*-substituents. Purification of every 2,3-disubstituted indole by simple filtration through alumina further underscores the synthetic utility of our reaction.

**Table 2:** Scope of Rh_2_^II^-catalyzed migratorial reactions

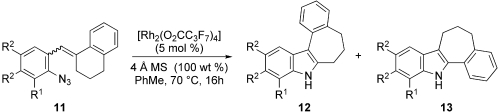						
Entry	**11**	R^1^	R^2^	R^3^	Yield [%][a]	**12**:**13**[b]
1	**a**	MeO	H	H	96	>95:5
2	**b**	H	MeO	H	97	>95:5
3	**c**	H	Cl	H	99	>95:5
4[c]	**d**	H	MeO_2_C	H	94	>95:5
5	**e**	H	F_3_C	H	90	>95:5
6[d]	**f**	H	MeO_2_S	H	95	>95:5
7	**g**	H	H	Br	95	>95:5
8[d]	**h**	H	H	MeO_2_C	95	>95:5

[a] Yield after Al_2_O_3_ chromatography.

[b] As determined by using ^1^H NMR spectroscopy.

[c] X-ray structure of product indole obtained.

[d] 5 mol % of [Rh_2_(esp)_2_] used.

The nature of the migrating group on the aryl azide was subsequently investigated (Table 3). For these substrates, only aryl group migration was observed. While rhodium perfluorobutyrate was a competent catalyst, [Rh_2_(esp)_2_] provided the highest yields of the reaction. Only indole **15 a** was observed when the tether was shortened (Table 3, entry 1). Appending the electron-withdrawing trifluoromethyl group or the electron-donating methoxy group to the migrating arene did not change the outcome of the reaction (Table 3, entries 2 and 3). In both cases, only aryl group migration was observed. High yields and selective formation of indole **15 d** was obtained when an oxygen atom was incorporated into the tether. The reaction was not limited to ring expansion: despite changing the electronic nature of the migrating aryl group, only indoles **15 e** and **15 f** were formed from azides **14 e** and **14 f**.

**Table 3:** Scope of Rh_2_^II^-catalyzed 2,3-disubstituted indole formation

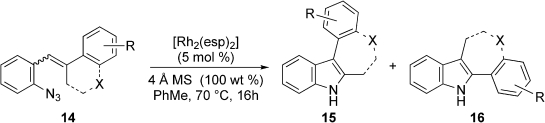				
Entry	**14, 17**	Styryl azide	Indole product	Yield [%][a]
1	**a**	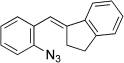	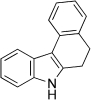	88
2	**b**	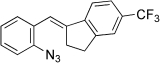	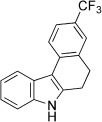	78
3	**c**	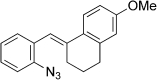	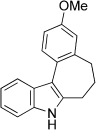	92
4	**d**	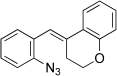	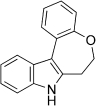	94
5	**e**	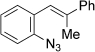	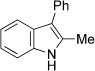	91
6	**f**	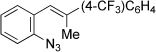	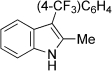	93

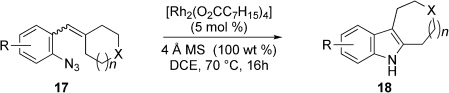				
7	**a**	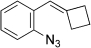	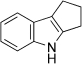	98
8	**b**	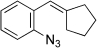	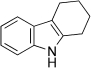	93
9	**c**	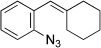	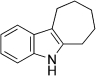	93
10	**d**	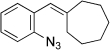	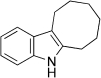	18^[b,c]^
11	**e**	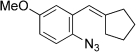	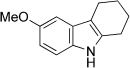	quant.
12	**f**	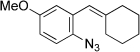	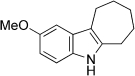	quant.
13	**g**	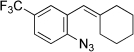	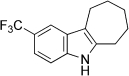	88
14	**h**	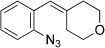	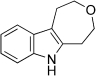	97

[a] Yield after Al_2_O_3_ chromatography. [b] As determined by using ^1^H NMR spectroscopy. [c] 35 % remaining **17 d**. DCE=dichloroethane.

The effect of ring size on the reaction efficiency was further examined using styryl azides **17**. For this series of substrates, rhodium octanoate proved to be the most reliable catalyst. While ring-expanded products were formed from 4-, 5-, and 6-membered substrates, poor conversion was observed for 7-membered **17 d** (Table 3, entries 7–10). Varying the electronic nature of the aryl azide did not attenuate the yield of the reaction (Table 3, entries 11–13). Oxygen atoms were tolerated in the tether without lowering the yield of the ring expansion (Table 3, entry 14).

**Scheme 2:**
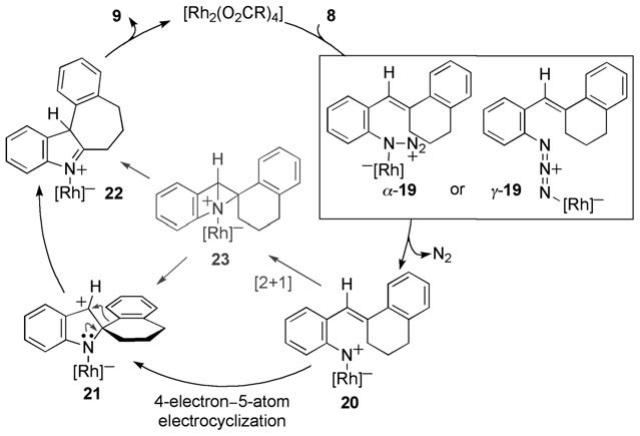
Potential mechanisms for indole formation.

While many mechanisms are possible to explain the reaction outcome, our data suggests that the migration occurs once an intermediate (**21**) is generated with positive charge on the benzylic carbon. We propose that this intermediate is formed by the mechanism outlined in Scheme 2. Coordination of the rhodium carboxylate complex to the azide produces either α-**19** or γ-**19**.[21] Extrusion of N_2_ from **19** forms rhodium nitrene **20**,[22] which participates in a 4π-electron–5-atom electrocyclization to establish the carbon–nitrogen bond in **21**.[7] Aryl migration forms the more stable tertiary iminium ion **22**, which tautermizes to produce **9**. Alternatively, the *ortho*-double bond could assist in N_2_ extrusion to form the intermediate **23**, or this intermediate could be formed from [2+1] cycloaddition of the pendant double bond with the electrophilic metallonitrene **20**. While **23** is strained,[23] its intermediacy would account for the enhanced reactivity of azides with unsaturated *ortho*-substituents.

**Scheme 3:**
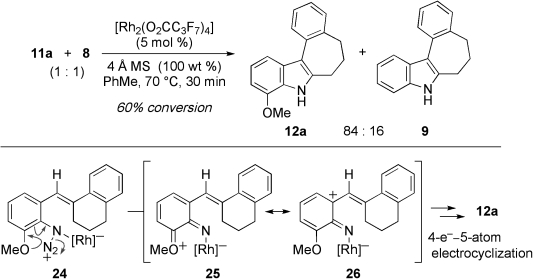
Intermolecular competition experiment.

We performed several experiments to test the validity of our mechanism. To examine whether N_2_ was lost before C–N bond formation, we performed an intermolecular competition experiment between azides **11 a** and **8** (Scheme 3). Our previous Hammett correlation study indicated that N_2_ extrusion occurred faster with electron-rich aryl azides.[7] Acceleration of metallonitrene formation was attributed to the ability of the electron-donating group to assist in N_2_ loss (**24** to **25**). In contrast, if N_2_ loss occurred simultaneously with C–N bond formation, we anticipated that **8** would react faster because the azide moiety was more electrophilic than in **11 a**. To test these assertions, a 1:1 mixture of styryl azides **11 a** and **8** were exposed to reaction conditions. Despite the increased steric pressure around the azide, the more electron-rich substrate reacted faster to produce indole **12 a** as the major product to support our proposed electrocyclization mechanism.

**Figure 1:**
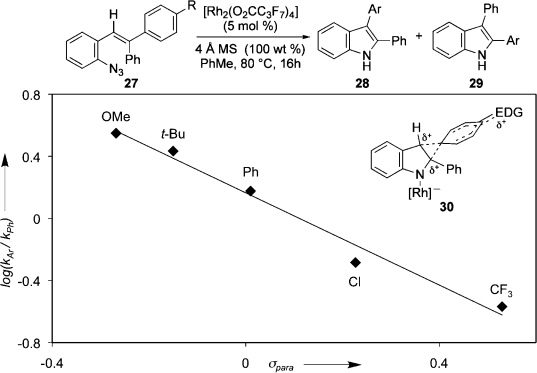
Correlation of product ratios with the Hammett equation. *y*=−1.49 *x*+0.17; *R*^2^=0.98.

If the migration mechanism involved the formation of a partial positive charge on the α-carbon, we anticipated that electron-rich aryl groups would migrate preferentially. To test this hypothesis, a series of styryl azides, which systematically varied the identity of the *para*-substituent R, were exposed to reaction conditions (Figure 1). Examination of the product ratios using the Hammett equation revealed that the best linear correlation was obtained with *σ_para_* values to give a *ρ* value of −1.49. The greater propensity of the more electron-rich aryl group to participate in the 1,2-shift was interpreted to suggest that the migration occurs through phenonium ion reactive intermediate **30**,[24, 25] where the more stable ion leads to the major product.

In conclusion, we have demonstrated that rhodium carboxylate complexes catalyze cascade reactions of β,β-disubstituted styryl azides to selectively produce 2,3-disubstituted indoles. Our data suggests that the selectivity of the migratorial process is controlled by the formation of a phenonium ion. Future experiments will be aimed at clarifying the mechanism of this reaction as well as determining if the benzylic cation can be intercepted with additional nucleophiles to produce complex, functionalized N-heterocycles from simple, readily available styryl azides.
