# Supplementary material for: Rhodium-Catalyzed Synthesis of 2,3-Disubstituted Indoles from β,β-Disubstituted Stryryl Azides
Source: Angew Chem Int Ed Engl. 2011 Jan 14;50(7):1702–6. doi: 10.1002/anie.201006917 (PMC3154515; doi:10.1002/anie.201006917)

Angewandte Chemie
Eine Zeitschrift der Gesellschaft Deutscher Chemiker

Supporting Information

© Wiley-VCH 2011

69451 Weinheim, Germany

Rhodium-Catalyzed Synthesis of 2,3-Disubstituted Indoles from β,β-Disubstituted Stryryl Azides**

*Ke Sun, Sheng Liu, Patryk M. Bec, and Tom G. Driver**

anie_201006917_sm_miscellaneous_information.pdf

Contents:

I. Preparation of Substrates using a Wittig Reaction……………………………….*s*-2
II. Preparation of Substrates using a Horner–Wadsworth–Emmons Reaction……….*s*-11
III. Preparation of Substrates using a Suzuki Reaction………………………………..*s*-14
IV. Development of Rhodium-Catalyzed Migration Reaction………………………..*s*-21
V. Mechanistic Experiments………………………………………………………..*s*-32
VI. References………………..…………………………………………………...*s*-44
V. Spectral Data…………………………………………………………………..*s*-45

General. ^{1}H NMR and ^{13}C NMR spectra were recorded at ambient temperature using 500 MHz or 300 MHz spectrometers. The data are reported as follows: chemical shift in ppm from internal tetramethylsilane on the δ scale, multiplicity (br = broad, s = singlet, d = doublet, t = triplet, q = quartet, m = multiplet), coupling constants (Hz) and integration. High resolution mass spectra were were obtained by peak matching. Melting points are reported uncorrected. Infared spectroscopy was obtained using a diamond attenuated total reflectance (ATR) accessory. Analytical thin layer chromatography was performed on 0.25 mm extra hard silica gel plates with UV254 fluorescent indicator. Liquid chromatography was performed using forced flow (flash chromatography) of the indicated solvent system on 60Å (40 – 60 μm) mesh silica gel (SiO_2). Medium pressure liquid chromatography (MPLC) was performed to force flow the indicated solvent system down columns that had been packed with 60Å (40 – 60 μm) mesh silica gel (SiO_2). All reactions were carried out under an atmosphere of nitrogen in glassware, which had been oven-dried. Unless otherwise noted, all reagents were commercially obtained and, where appropriate, purified prior to use. Acetonitrile, Methanol, Toluene, THF, Et_2O, and CH_2Cl_2 were dried by filtration through alumina according to the procedure of Grubbs.[1] Metal salts were stored in a nitrogen atmosphere dry box.

I. Preparation of Substrates using Wittig Reaction.

A. Routes to Substrates

The stryryl azides investigated in this study were synthesized by one three different routes (Scheme s1). The majority of the substrates were formed in two steps from commercially available *ortho*-nitro-substituted benzaldehydes starting by a nucleophilic aromatic substitution using NaN_3 followed by a Wittig reaction. Alternatively the styryl azides were constructed from phosphonate **s3** using a Horner–Wadworth–Emmons reaction of phosphonate **s3**, followed by an iron-mediated reduction to form aniline **s4**. Azidation then provided the required styryl azide. Finally styryl azides, **14b** and **14c**, were accessed from boronic acid **s5** and the corresponding vinyl triflate **s6**.

Scheme s1. Routes to Styryl Azides.

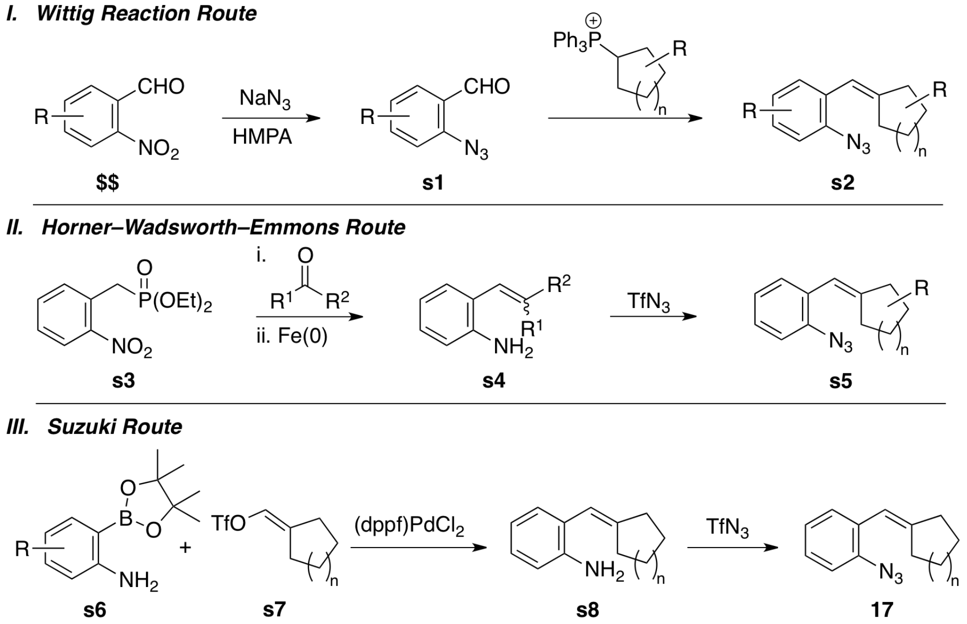

B. General Procedure for the Nucleophilic Substitution of 2-Nitrobenzaldehydes.

Following the procedure of Driver and co-workers,[2] the 2-azidobenzaldehydes were prepared. Yields were not optimized.

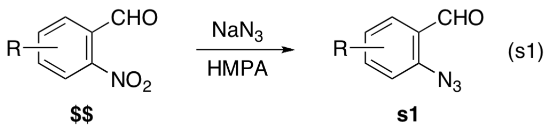

To a dry, stir bar-equipped scintillation vial were added 0.453 g of 2-nitro-benzaldehyde (3.0 mmol), 0.585 g of NaN_3 (9.0 mmol), and 9.0 mL of HMPA. The reaction was stirred overnight at room temperature, then was taken up in diethyl ether (100 mL) and washed with water (5 × 50 mL). The organic layer was dried over Na_2SO_4 and filtered. The filtrate was concentrated *in vacuo*. Purification with MPLC afford the 2-azido-benzaldehyde.

C. Synthesis of 2-Azidobenzaldehydes.

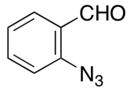

s9

Benzaldehyde s9.[2] The general procedure was followed using 2.10 g of 2-nitrobenzaldehyde (13.9 mmol) and 2.92 g of NaN_3 (45.0 mmol, 3.3 eq) in 40 mL of HMPA. Purification by MPLC (1:100 EtOAc: hexanes) afforded the product as a light yellow solid (1.70 g, 83%); mp 33 - 36 °C; The spectral data matched that reported by Driver and co-workers: ^{1}H NMR (500 MHz, $CDCl_3$) 10.25 (s, 1H), 7.79 (dd, J = 1.5 Hz, 7.5 Hz, 1H), 7.54 (dt, J = 1.5 Hz, 8.0 Hz, 1H) 7.14 – 7.19 (m, 2H); ^{13}C NMR (125 MHz, $CDCl_3$) δ 188.4 (CH), 142.8 (C), 135.4 (CH), 128.9 (CH), 126.9 (C), 124.8 (C), 119.1 (CH); IR (thin film): 2971, 2121, 1690, 1594, 1477, 1290, 1274 cm^{-1}.

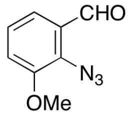

s10

Benzaldehyde s10. The general procedure was followed using 1.81 g of 2-nitro-3-methoxybenzaldehyde (10 mmol) and 1.31 g of NaN_3 (20 mmol) in 50 mL of HMPA at 50 °C. Purification of the reaction mixture using MPLC (1:30 EtOAc:hexanes) afforded the product as a white solid (1.61 g, 91%); mp 54 – 56 °C; ^{1}H NMR (500 MHz; $CDCl_3$) δ 10.15 (s, 1H), 7.82 (d, J = 8.5 Hz, 1H), 6.72 (dd, J = 2.5 Hz, 8.5 Hz, 1H), 6.67 (d, J = 2.5 Hz, 1H), 3.88 (s, 3H); ^{13}C NMR (125 MHz, $CDCl_3$) δ 187.2 (CH), 165.3 (C), 144.9 (C), 131.1 (CH), 121.0 (C), 111.1 (CH), 103.9 (CH), 55.8 (CH_3); IR (thin film): 2359, 2341, 2110, 1723, 1264, 730, 703 cm^{-1}. HRMS (EI) m / z calcd for $C_8H_7O_2N_3$ $(M)^+$ 177.05383, found 177.05529.

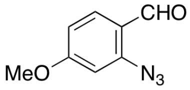

s11

Benzaldehyde s11. The general procedure was followed using 1.81 g of 2-nitro-4-methoxybenzaldehyde (10 mmol) and 1.31 g of NaN_3 (20 mmol) in 50 mL of HMPA at 50 °C. Purification of the reaction mixture using MPLC (1:30 EtOAc: hexanes) afforded the product as a white solid (1.62 g, 92%); mp 73 – 75 °C; ^{1}H NMR (500 MHz; $CDCl_3$) δ 10.17 (s, 1H), 7.84 (d, J = 9.0 Hz, 1H), 6.74 (dd, J = 2.5 Hz, 9.0 Hz, 1H), 6.68 (d, J = 2.5 Hz, 1H), 3.90 (s, 3H); ^{13}C NMR (125 MHz, $CDCl_3$) δ 187.2 (CH), 165.3 (C), 144.9 (C), 131.1 (CH), 121.1 (C), 111.1 (CH), 104.0 (CH), 55.8 (CH_3); IR (thin film): 3457, 3015, 2970, 2116, 1744, 1367, 1229, 750, 528 cm^{-1}. HRMS (EI) m / z calcd for $C_8H_7O_2N_3$ $(M)^+$ 177.05383, found 177.05520.

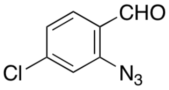

s12

Benzaldehyde s12. The general procedure was followed using 1.85 g of 2-nitro-4-chlorobenzaldehyde (10 mmol) and 0.98 g of NaN_3 (15 mmol) in 50 mL of HMPA. Purification of the reaction mixture using MPLC (1:100 EtOAc:hexanes) afforded the product as a white solid (1.45 g, 80%); mp 91 – 93 °C; ^{1}H NMR (500 MHz; $CDCl_3$) δ 10.28 (s, 1H), 7.82 (d, J = 8.5 Hz, 1H), 7.26 (d, J = 2.0 Hz, 1H), 7.21 (dd, J = 1.5 Hz, 8.5 Hz, 1H); ^{13}C NMR (125 MHz, $CDCl_3$) δ 187.4 (CH), 144.0 (C), 141.7 (C), 130.2 (CH), 125.6 (CH), 125.4 (C),

119.2 (CH); IR (thin film): 3455, 3016, 2969, 2123, 1739, 1366, 1216, 750, 528 cm^{-1}. HRMS (EI) *m / z* calcd for $C_7H_4ON_3Cl$ $(M)^+$ 183.00134, found 183.00217.

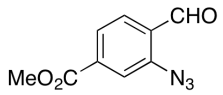

s13

Benzaldehyde s13. The general procedure was followed using 2.09 g of methyl 4-formyl-3-nitrobenzoate (10 mmol) and 1.31 g of NaN_3 (20 mmol) in 50 mL of HMPA. Purification of the reaction mixture using MPLC (1:30 EtOAc: hexanes) afforded the product as a white solid (1.95 g, 95 %); mp 112 – 114 °C; ^{1}H NMR (500 MHz; $CDCl_3$) δ 10.38 (s, 1H), 7.94 – 7.92 (m, 2H), 7.85 – 7.83 (m, 1H), 3.97 (s, 3H); ^{13}C NMR (125 MHz, $CDCl_3$) δ 188.1 (CH), 165.3 (C), 143.1 (C), 136.2 (C), 129.4 (C), 129.1 (CH), 125.6 (CH), 120.3 (C), 52.8 (CH_3); IR (thin film): 3458, 3015, 2970, 2122, 1738, 1366, 1216, 762, 527 cm^{-1}. HRMS (EI) *m / z* calcd for $C_9H_7O_3N_3$ $(M)^+$ 205.04875, found 205.04990.

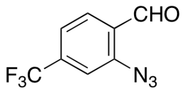

s14

Benzaldehyde s14.[3] The general procedure was followed using 2.69 g of 2-nitro-4-trifluoromethylbenzaldehyde (12.3 mmol) and 2.21 g of NaN_3 (34.0 mmol) in 50 mL of HMPA. Purification by MPLC (1:100 EtOAc: hexanes) afforded the product as a yellow solid (2.49 g, 94%): mp 89 -91 °C; The spectral data matched that reported by Driver and co-workers:[2] ^{1}H NMR (500 MHz, $CDCl_3$) 10.38 (s, 1H), 8.00 (d, J = 8.0 Hz, 1H), 7.50 (s, 1H) 7.48 (d, J = 8.0 Hz, 1H); ^{13}C NMR (125 MHz, $CDCl_3$): δ 187.5 (CH), 143.5 (C), 136.8 (q, J_{CF} = 31.4 Hz, C), 129.8 (CH), 126.1 (C), 122.9 (q, J_{CF} = 270.9 Hz, C), 121.6 (q, J_{CF} = 3.5 Hz, CH) 116.3 (q, J_{CF} = 36.3 Hz, CH); ^{19}F NMR ($CDCl_3$, 282 MHz) δ –61.10; IR (thin film): 2980, 2901, 2122, 1689, 1414, 1392, 1328, 1072. 750 cm^{-1}.

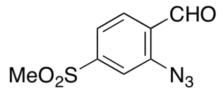

s15

Benzaldehyde s15. The general procedure was followed using 2.29 g of 4-(methylsulfonyl)-2-nitrobenzaldehyde (10 mmol) and 1.31 g of NaN_3 (20 mmol) in 50 mL of HMPA. Purification of the reaction mixture using MPLC (1:10 EtOAc: hexanes) afforded the product as a white solid (1.96 g, 87%); mp 125 – 127 °C; ^{1}H NMR (500 MHz; $CDCl_3$) δ 10.40 (s, 1H), 8.05 (d, J = 8.0 Hz, 1H), 7.85 (s, 1H), 7.5 (d, J = 8.0 Hz, 1H), 3.12 (s, 3H); ^{13}C NMR (125 MHz, $CDCl_3$) δ 187.3 (CH), 146.5 (C), 144.1 (C), 130.3 (CH), 129.8 (C), 132.3 (CH), 118.4 (CH), 44.2 (CH_3); IR (thin film): 3023, 2925, 2852, 2115, 1681, 1398, 1279, 1159, 1135 cm^{-1}.

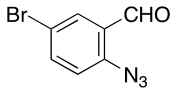

s16

Benzaldehyde s16.[4] The general procedure was followed using 2.03 g of 2-nitro-5-bromobenzaldehyde (10 mmol) and 1.3 g of NaN_3 (20 mmol) in 40 mL of DMSO at 50 °C. Purification of the reaction mixture using MPLC (1:100 EtOAc:hexanes) afforded the product as a white solid (2.19 g, 97%); mp 94 – 96 °C; ^{1}H NMR (500 MHz; $CDCl_3$) δ 10.27 (s, 1H), 7.98 (d, J = 2.5 Hz, 1H), 7.70 (dd, J = 2.5 Hz, 8.5 Hz, 1H), 7.16 (d, J = 8.5 Hz, 1H); ^{13}C NMR (125 MHz, $CDCl_3$) δ 187.1 (CH), 141.9 (C), 138.0 (CH), 131.7 (CH), 128.0 (C), 120.8

(CH), 118.3 (C); IR (thin film): 3457, 3005, 2970, 2130, 1725, 1368, 1214, 750, 527 cm^{-1}. HRMS (EI) *m / z* calcd for $C_7H_4ON_3Br$ $(M)^+$ 224.95377, found 224.95459.

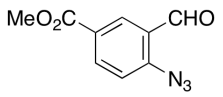

s17

Benzaldehyde s17. The general procedure was followed using 2.09 g of methyl 3-formyl-4-nitrobenzoate (10 mmol) and 1.31 g of NaN_3 (20 mmol) in 50 mL of HMPA. Purification of the reaction mixture using MPLC (1:30 EtOAc:hexanes) afforded the product as a white solid (1.97 g, 96%); mp 118 –110 °C; ^{1}H NMR (500 MHz; $CDCl_3$) δ 10.31 (s, 1H), 8.49 (d, *J* = 2.0 Hz, 1H), 8.23 (dd, *J* = 2.0 Hz, 8.5 Hz, 1H), 7.31 (d, *J* = 8.5 Hz, 1H), 3.97 (s, 3H); ^{13}C NMR (125 MHz, $CDCl_3$) δ 187.7 (CH), 165.4 (C), 146.8 (C), 136.0 (CH), 130.8 (CH), 127.0 (C), 127.6 (C), 119.2 (CH), 52.4 (CH_3); IR (thin film): 3458, 3015, 2970, 2122, 1738, 1366, 1216, 762, 527 cm^{-1}. HRMS (EI) *m / z* calcd for $C_9H_7O_3N_3$ $(M)^+$ 205.04875, found 205.04980.

D. General Procedure for the Phosphonium Salt Synthesis.

Unless otherwise noted, the phosphonium salt was synthesized from the corresponding cycloketone. Yields were not optimized.

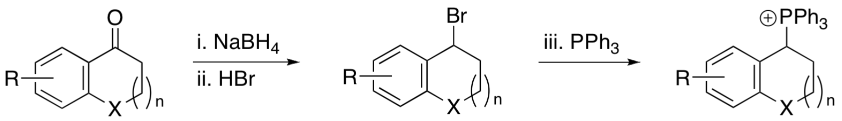

(s2)

To a solution of 2.92 g of α-tetralone (20 mmol) in 30 mL of EtOH was added 1.52 g of $NaBH_4$ (40 mmol). The mixture was monitored by thin layer chromatography. Once the starting material spot had disappeared, the mixture was concentrated *in vacuo*, and the resulting material was diluted with 50 mL of water. The resulting aqueous mixture was extracted with dichloromethane (3 × 10 mL). The organic phase (containing the alcohol) was treated with proportional HBr. The progress of the reaction was monitored by thin layer chromatography. Once the alcohol completely consumed, the mixture was diluted with 100 mL of water, then extracted with dichloromethane (3 × 20 mL). The resulting organic phase was dried over Na_2SO_4. The mixture was filtered and the filtrate was concentrated *in vacuo* to afford bromide. To a solution of the bromide in 60 mL of PhMe was added 5.76 g of PPh_3. The reaction mixture was heated to reflux. After 16h, the mixture was cooled down to room temperature, then filtrated. The solid was washed with diethyl ether (3 × 30 mL), and dried under high vacuum to afford phosphonium salt.

E. General Procedure for the Wittig Reaction of 2-Azidobenzaldehydes.

Following the procedure of Scott and co-workers,[5] the 2-azidobenzaldehydes were prepared. Yields were not optimized.

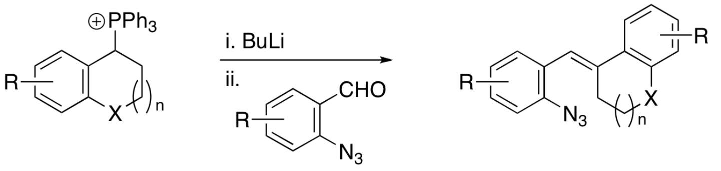

(s3)

To a solution of 4.0 mmol of the phosphonium salt in 12 mL of THF at –78 °C, was added 1.6 mL of *n*-BuLi (2.5 M in hexanes, 4.0 mmol) dropwise. After stirring 1h at –78 °C, the mixture was warmed to room

temperature and stirred additional one hour. Then 0.647 g of 2-azidobenzaldehyde (4.4 mmol) was added and the mixture was stirred for 1 h at ambient temperature. The resulting mixture was diluted with 15 mL of water and extracted with CH_2Cl_2 (3 × 10 mL). The resulting organic phase was washed with 20 mL of brine and dried over Na_2SO_4. After filtration, the filtrate was concentrated in *vacuo*. Purification of the reaction mixture using MPLC afforded the product.

E. Synthesis of Styryl Azides.

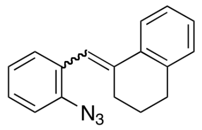

8

Styryl Azide 8. The general procedure was followed using 1.94 g of 2-azidobenzaldehyde **s9** (13.2 mmol), 6.85 g of the phosphonium salt (14.5 mmol), 5.8 mL of a 2.5 M solution of BuLi in hexanes in 50 mL of THF. Purification by MPLC (pure hexanes) afforded the product as a white powder, as an 84:16 mixture of *E*- and *Z*-isomers (2.24 g, 65%): R_f= 0.45 (18:82 EtOAc: hexane). Selected spectral data for the major (*E*) isomer: ^{1}H NMR (500 MHz, $CDCl_3$) δ 7.80 -7.99 (m, 1H), 7.33 – 7.37 (m, 2H), 7.13 – 7.26 (m, 5H), 7.07 (s, 1H), 2.91 (t, *J* = 6.5 Hz, 2H), 2.71 (t, *J* = 6.0 Hz, 2H), 1.87 – 1.92 (m, 2H); ^{13}C NMR (125 MHz, $CDCl_3$) δ 138.7 (C), 138.6 (C), 137.9 (C), 135.9 (C), 130.9 (CH), 130.1 (C), 129.2 (CH), 128.1 (CH), 127.6 (CH), 126.2 (CH), 124.7 (CH), 124.3 (CH), 118.7 (CH), 118.4 (CH); 30.4 (CH_2), 28.2 (CH_2), 23.8 (CH_2); Selected spectral data for the minor (*Z*) isomer: ^{1}H NMR (500 MHz, $CDCl_3$) δ 7.13 – 7.26 (m, 5H), 7.07 – 7.08 (m, 1H), 6.95 – 6.98 (m, 1H), 6.85 – 6.88 (m, 1H), 6.47 (s, 1H), 2.95 (t, *J* = 6.5 Hz, 2H), 2.63 (t, *J* = 6.5 Hz, 2H), 2.05 – 2.10 (m, 2H); ^{13}C NMR (125 MHz, $CDCl_3$) δ 139.8 (C), 138.7 (C), 138.0 (C), 134.7 (C), 131.3 (CH), 130.1 (C), 128.9 (CH), 128.0 (CH), 127.6 (CH), 124.7 (CH), 124.5 (CH), 119.9 (CH), 118.5 (CH), 35.0 (CH_2), 29.7 (CH_2), 24.2 (CH_2); Selected spectral data for the mixture: δ IR (thin film): 2117, 2083, 1486, 1445, 756, 745 cm^{-1}. HRMS (EI) *m* / *z* calcd for $C_{17}H_{15}N_3$ $(M – N_2)^+$ 261.12660, found 261.12679.

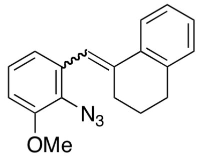

11a

Styryl Azide 11a. The general procedure was followed using 0.58 g of 2-azido-3-methoxybenzaldehyde **s10** (3.3 mmol), 1.42 g of the phosphonium salt (3.0 mmol), 1.2 mL of a 2.5 M solution of BuLi in hexanes in 25 mL of THF. Purification by MPLC (1:50 EtOAc:hexanes) afforded the product as a white powder, as a 76:24 mixture of *E*- and *Z*-isomers (0.63 g, 72%). Selected spectral data for the major (*E*) isomer: ^{1}H NMR (500 MHz, $CDCl_3$) δ 7.76 – 7.74 (m, 1H), 7.23 – 7.21 (m, 2H), 7.14 – 7.04 (m, 3H), 6.93 – 6.90 (m, 1H), 6.84 – 6.82 (m, 1H), 3.93 (s, 3H), 2.88 (t, *J* = 6.5 Hz, 2H), 2.66 (dt, *J* = 1.5 Hz, 6.0 Hz, 2H), 1.86 (pent, *J* = 6.5 Hz, 2H); ^{13}C NMR (125 MHz, $CDCl_3$) δ 153.9 (C), 138.5 (C), 137.8 (C), 135.9 (C), 131.8 (C), 129.2 (CH), 127.6 (CH), 126.9 (C), 126.2 (CH), 124.6 (CH), 124.6 (CH), 122.8 (CH), 119.4 (CH), 110.2 (CH), 56.1 (CH_3), 30.3 (CH_2), 28.2 (CH_2), 23.7 (CH_2); Selected spectral data for the minor (*Z*) isomer: ^{1}H NMR (500 MHz, $CDCl_3$) δ 7.27 – 7.25 (m, 1H), 7.14 – 7.04 (m, 3H), 6.88 – 6.85 (m, 1H), 6.75 – 6.71 (m, 2H), 6.41 (s, 1H), 3.91 (s, 3H), 2.92 (t, *J* = 6.5 Hz, 2H), 2.60 (dt, *J* = 1.5 Hz, 6.0 Hz, 2H), 2.04 (pent, *J* = 6.5 Hz, 2H); ^{13}C NMR (125 MHz, $CDCl_3$) δ 153.9 (C), 139.5 (C), 138.8 (C), 134.8 (C), 132.5 (C), 129.0 (CH), 128.8 (CH), 127.5 (CH), 125.7 (CH), 125.0 (C), 124.8 (CH), 123.1 (CH), 120.5 (CH), 109.9 (CH), 56.0 (CH_3), 34.9 (CH_2), 29.7 (CH_2), 24.2 (CH_2); Selected

spectral data for the mixture: δ IR (thin film): 2933, 2103, 1756, 1450, 1262, 1087, 737 cm^{-1}. HRMS (EI) *m / z* calcd for $C_{18}H_{17}ON$ $(M-N_2)^+$ 263.13102, found 163.132000.

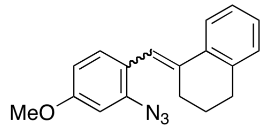

11b

Styryl Azide 11b. The general procedure was followed using 0.58 g of 2-azido-4-methoxybenzaldehyde **s11** (3.3 mmol), 1.42 g of the phosphonium salt (3.0 mmol), 1.2 mL of a 2.5 M solution of BuLi in hexanes in 25 mL of THF. Purification by MPLC (1:50 EtOAc: hexanes) afforded the product as a white powder, as an 83:17 mixture of *E*- and *Z*-isomers (0.37 g, 43%). Selected spectral data for the major (*E*) isomer: ^{1}H NMR (500 MHz, $CDCl_3$) δ 7.74 – 7.72 (m, 1H), 7.26 (d, *J* = 8.5 Hz, 1H), 7.23 – 7.19 (m, 2H), 7.14 – 7.12 (m, 1H), 6.97 (s, 1H), 6.74 – 6.71 (m, 2H), 3.87 (s, 3H), 2.87 (t, *J* = 6.5 Hz, 2H), 2.67 (dt, *J* = 1.5 Hz, 6.0 Hz, 2H), 1.87 (pent, *J* = 6.5 Hz, 2H); ^{13}C NMR (125 MHz, $CDCl_3$) δ 159.5 (C), 139.6 (C), 137.7 (C), 137.5 (C), 136.1 (C), 131.7 (CH), 129.1 (CH), 127.4 (CH), 126.2 (CH), 124.5 (CH), 122.8 (C), 118.3 (CH), 110.0 (CH), 104.2 (CH), 55.5 (CH_3), 30.3 (CH_2), 28.1 (CH_2), 23.7 (CH_2); Selected spectral data for the minor (*Z*) isomer: ^{1}H NMR (500 MHz, $CDCl_3$) δ 7.23 – 7.19 (m, 1H), 7.10 – 7.05 (m, 3H), 6.86 (t, *J* = 6.5 Hz, 1H), 6.69 (d, *J* = 2.5 Hz, 1H), 6.51 (dd, *J* = 2.5 Hz, 8.5 Hz, 1H), 6.33 (s, 1H), 3.83 (s, 3H), 2.91 (t, *J* = 6.5 Hz, 2H), 2.57 (dt, *J* = 1.5 Hz, 6.0 Hz, 2H), 2.03 (pent, *J* = 6.5 Hz, 2H); ^{13}C NMR (125 MHz, $CDCl_3$) δ 159.5 (C), 138.7 (C), 134.9 (C), 132.7 (C), 132.0 (CH), 128.9 (CH), 128.7 (CH), 128.6 (CH), 128.5 (C), 124.7 (CH), 123.1 (C), 119.4 (CH), 110.6 (CH), 104.0 (CH), 55.4 (CH_3), 34.9 (CH_2), 29.7 (CH_2), 24.2 (CH_2); Selected spectral data for the mixture: δ IR (thin film): 2935, 2101, 1753, 1436, 1266, 1084, 739 cm^{-1}. HRMS (EI) *m / z* calcd for $C_{18}H_{17}ON_3$ $(M)^+$ 291.13717, found 291.13649.

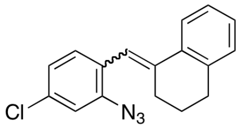

11c

Styryl Azide 11c. The general procedure was followed using 0.60 g of 2-azido-4-chlorobenzaldehyde **s12** (3.3 mmol), 1.42 g of the phosphonium salt (3.0 mmol), 1.2 mL of a 2.5 M solution of BuLi in hexanes in 25 mL of THF. Purification by MPLC (1:100 EtOAc: hexanes) afforded the product as a white powder, as an 82:18 mixture of *E*- and *Z*-isomers (0.49 g, 55%). Selected spectral data for the major (*E*) isomer: ^{1}H NMR (500 MHz, $CDCl_3$) δ 7.71 – 7.70 (m, 1H), 7.26 – 7.20 (m, 3H), 7.16 (d, *J* = 2.0 Hz, 1H), 7.13 – 7.12 (m, 2H), 6.91 (s, 1H), 2.86 (t, *J* = 6.5 Hz, 2H), 2.62 (dt, *J* = 1.5 Hz, 6.5 Hz, 2H), 1.85 (pent, *J* = 6.5 Hz, 2H); ^{13}C NMR (125 MHz, $CDCl_3$) δ 139.8 (C), 139.3 (C), 137.9 (C), 135.6 (C), 133.3 (C), 131.8 (CH), 129.2 (CH), 128.6 (C), 127.8 (CH), 126.3 (CH), 124.6 (CH), 124.6 (CH), 118.6 (CH), 117.6 (CH), 30.3 (CH_2), 28.2 (CH_2), 23.7 (CH_2); Selected spectral data for the minor (*Z*) isomer: ^{1}H NMR (500 MHz, $CDCl_3$) δ 7.23 – 7.19 (m, 1H), 7.11 – 7.09 (m, 1H), 7.04 (d, *J* = 8.0 Hz, 1H), 6.98 (d, *J* = 8.0 Hz, 1H), 6.88 (dd, *J* = 2.0 Hz, 8.5 Hz, 1H), 6.84 (t, *J* = 6.0 Hz, 1H), 6.27 (s, 1H), 2.89 (t, *J* = 6.5 Hz, 2H), 2.56 (dt, *J* = 1.5 Hz, 6.5 Hz, 2H), 2.03 (pent, *J* = 6.5 Hz, 2H); ^{13}C NMR (125 MHz, $CDCl_3$) δ 140.6 (C), 139.2 (C), 139.0 (C), 134.4 (C), 133.2 (C), 132.3 (CH), 129.1 (C), 129.0 (CH), 128.8 (CH), 124.8 (CH), 124.8 (CH), 124.6 (CH), 118.7 (CH), 118.6 (CH), 34.9 (CH_2), 29.7 (CH_2), 24.1 (CH_2); Selected spectral data for the mixture: δ IR (thin film): 2931, 2109, 1738, 1486, 1286, 756 cm^{-1}. HRMS (EI) *m / z* calcd for $C_{17}H_{14}N_3Cl$ $(M)^+$ 295.08762, found 295.08889.

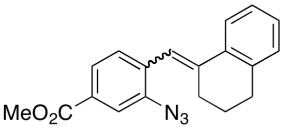

11d

Styryl Azide 11d. The general procedure was followed using 0.68 g of methyl 3-azido-4-formylbenzoate **s13** (3.3 mmol), 1.42 g of the phosphonium salt (3.0 mmol), 1.2 mL of a 2.5 M solution of BuLi in hexanes in 25 mL of THF. Purification by MPLC (1:50 EtOAc: hexanes) afforded the product as a white powder, as an 83:17 mixture of *E*- and *Z*-isomers (0.39 g, 41%). Selected spectral data for the major (*E*) isomer: ^{1}H NMR (500 MHz, $CDCl_3$) δ 7.85 (d, *J* = 1.5 Hz, 1H), 7.80 (dd, *J* = 1.5 Hz, 8.0 Hz, 1H), 7.73 (dd, *J* = 3.5 Hz, 5.5 Hz, 1H), 7.39 (d, *J* = 3.0 Hz, 1H), 7.22 (dd, *J* = 3.5 Hz, 5.5 Hz, 2H), 7.14 – 7.12 (m, 1H), 7.00 (s, 1H), 3.95 (s, 3H), 2.87 (t, *J* = 6.5 Hz, 2H), 2.67 (dt, *J* = 1.5 Hz, 6.5 Hz, 2H), 1.86 (pent, *J* = 6.5 Hz, 2H); ^{13}C NMR (125 MHz, $CDCl_3$) δ 166.2 (C), 140.4 (C), 139.1 (C), 138.1 (C), 135.5 (C), 134.6 (C), 130.7 (CH), 129.6 (C), 129.2 (CH), 128.0 (CH), 126.3 (CH), 125.4 (CH), 124.7 (CH), 119.4 (CH), 117.9 (CH), 52.4 (CH_3), 30.2 (CH_2), 28.3 (CH_2), 23.7 (CH_2); Selected spectral data for the minor (*Z*) isomer: ^{1}H NMR (500 MHz, $CDCl_3$) δ 7.82 (d, *J* = 1.5 Hz, 1H), 7.55 (dd, *J* = 3.0 Hz, 8.0 Hz, 1H), 7.23 – 7.18 (m, 1H), 7.14 – 7.12 (m, 2H), 6.94 (d, *J* = 8.5 Hz, 1H), 6.80 (t, *J* = 8.5 Hz, 1H), 6.35 (s, 1H), 3.92 (s, 3H), 2.90 (t, *J* = 6.5 Hz, 2H), 2.59 (dt, *J* = 1.5 Hz, 6.5 Hz, 2H), 2.03 (pent, *J* = 6.5 Hz, 2H); ^{13}C NMR (125 MHz, $CDCl_3$) δ 166.2 (C), 141.1 (C), 139.0 (C), 138.3 (C), 135.5 (C), 134.2 (C), 131.2 (CH), 129.2 (CH), 129.0 (CH), 128.8 (CH), 128.8 (C), 125.4 (CH), 124.8 (CH), 119.6 (CH), 119.0 (CH), 52.3 (CH_3), 34.9 (CH_2), 29.6 (CH_2), 24.0 (CH_2); Selected spectral data for the mixture: δ IR (thin film): 3456, 3015, 2970, 2116, 1739, 1436, 1367, 1228, 1092, 748, 527 cm^{-1}. HRMS (EI) *m / z* calcd for $C_{19}H_{17}O_2N_3$ $(M)^+$ 319.13208, found 319.13140.

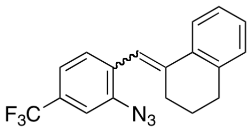

11e

Styryl Azide 11e. The general procedure was followed using 0.95 g of 2-azidobenzaldehyde **s14** (4.4 mmol), 1.89 g of thephosphonium salt (4.0 mmol), 1.8 mL of a 2.5 M solution of BuLi in hexanes in 20 mL of THF. Purification by MPLC (1:100 EtOAc: hexanes) afforded the product as a light yellow powder, as a 87:13 mixture of *E*- and *Z*-isomers (1.81 g, 81%): Selected spectral data for the major (*E*) isomer: ^{1}H NMR (500 MHz, $CDCl_3$) δ 7.75 – 7.77 (m, 1H), 7.40 – 7.46 (m, 3H), 7.24 – 7.28 (m, 2H), 7.14 – 7.18 (m, 1H), 7.02 (s, 1H), 2.91 (m, *J* = 6.5 Hz, 2H), 2.67 – 2.69 (m, 2H), 1.89 (pent, *J* = 6.5 Hz, 2H); ^{13}C NMR (125 MHz, $CDCl_3$) δ 140.5 (C), 139.4 (C), 133.6 (C), 131.3 (CH), 130.1 (q, J_{CF} = 32.6 Hz, C), 129.3 (CH), 129.0 (C), 128.1 (CH), 126.3 (CH), 124.7 (CH), 123.8 (q, J_{CF} = 270.8 Hz, C), 121.0 (q, J_{CF} = 3.5 Hz, CH), 117.4 (CH), 115.3 (q, J_{CF} = 3.8 Hz, CH), 30.2 (CH_2), 28.2 (CH_2), 23.7 (CH_2) one overlapping aromatic carbon signal; Selected spectral data for the minor (*Z*) isomer: ^{1}H NMR (500 MHz, $CDCl_3$) δ 7.40 – 7.46 (m, 1H), 7.24 – 7.28 (m, 1H), 7.14 – 7.18 (m, 3H), 6.99 (d, *J* = 8.0 Hz, 1H), 6.86 – 6.89 (m, 1H), 6.37 (s, 1H), 2.95 (t, *J* = 6.5 Hz, 2H), 2.62 – 2.64 (m, 2H), 2.01 (pent, *J* = 6.5 Hz, 2H); ^{13}C NMR (125 MHz, $CDCl_3$) δ 141.9 (C), 139.1 (C), 138.8 (C), 134.3 (C), 134.1 (C), 138.1 (CH), 135.3 (CH), 131.8 (CH), 129.0 (CH), 128.8 (CH), 124.9 (CH), 118.5 (CH), 115.5 (q, J_{CF} = 3.8 Hz, CH), 34.9 (CH_2), 29.6 (CH_2), 24.0 (CH_2); ^{19}F NMR ($CDCl_3$, 282 MHz) δ –63.12; Selected spectral data for the mixture: IR (thin film): 2111, 1739, 1328, 1273, 1123, 913, 745 cm^{-1}. HRMS (EI) *m / z* calcd for $C_{18}H_{14}N_3F_3$ $(M)^+$ 329.11398, found 329.11303.

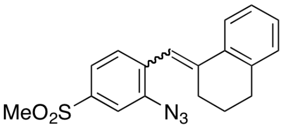

11f

Styryl Azide 11f. The general procedure was followed using 0.74 g of 2-azido-4-(methylsulfonyl)benzaldehyde **s15** (3.3 mmol), 1.42 g of the phosphonium salt (3.0 mmol), 1.2 mL of a 2.5 M solution of BuLi in hexanes in 25 mL of THF. Purification by MPLC (1:20 EtOAc: hexanes) afforded the product as a white powder as one isomer (0.47 g, 46%): ^{1}H NMR (500 MHz, $CDCl_3$) δ 7.73 (d, *J* = 1.5 Hz, 2H), 7.68 (dd, *J* = 1.5 Hz, 8.0 Hz, 1H), 7.50 (d, *J* = 8.0 Hz, 1H), 7.25 – 7.22 (m, 2H), 7.15 – 7.13 (m, 1H), 6.98 (s, 1H), 3.11 (s, 3H), 2.88 (t, *J* = 6.5 Hz, 2H), 2.66 (dt, *J* = 1.5 Hz, 6.5 Hz, 2H), 1.87 (pent, *J* = 6.5 Hz, 2H); ^{13}C NMR (125 MHz, $CDCl_3$) δ 141.5 (C), 140.1 (C), 139.7 (C), 138.2 (C), 135.5 (C), 135.1 (C), 131.7 (CH), 129.3 (CH), 128.3 (CH), 126.3 (CH), 124.7 (CH), 123.0 (CH), 117.2 (CH), 117.1 (CH), 44.6 (CH_3), 30.1 (CH_2), 28.3 (CH_2), 23.7 (CH_2); IR (thin film): 3454, 3026, 2969, 2116, 1738, 1366, 1206, 738, 536 cm^{-1}. HRMS (EI) *m* / *z* calcd for $C_{18}H_{17}O_2NS$ $(M-N_2)^+$ 311.09800, found 311.09698.

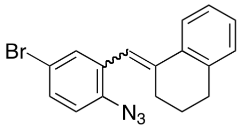

11g

Styryl Azide 11g. The general procedure was followed using 0.75 g of 2-azido-5-bromobenzaldehyde **s16** (3.3 mmol), 1.42 g of the phosphonium salt (3.0 mmol), 1.2 mL of a 2.5 M solution of BuLi in hexanes in 25 mL of THF. Purification by MPLC (1:100 EtOAc: hexanes) afforded the product as a white powder, as an 83:17 mixture of *E*- and *Z*-isomers (0.62 g, 61%). Selected spectral data for the major (*E*) isomer: ^{1}H NMR (500 MHz, $CDCl_3$) δ 7.73 – 7.71 (m, 1H), 7.45 (d, *J* = 2.5 Hz, 1H), 7.42 (dd, *J* = 2.5 Hz, 8.5 Hz, 1H), 7.25 – 7.21 (m, 2H), 7.17 – 7.14 (m, 1H), 7.05 (d, *J* = 8.5 Hz, 1H), 6.92 (s, 1H), 2.88 (t, *J* = 6.5 Hz, 2H), 2.66 (dt, *J* = 1.5 Hz, 6.5 Hz, 2H), 1.87 (pent, *J* = 6.5 Hz, 2H); ^{13}C NMR (125 MHz, $CDCl_3$) δ 139.9 (C), 138.0 (C), 137.8 (C), 135.4 (C), 133.4 (CH), 131.9 (C), 130.7 (CH), 129.2 (CH), 128.0 (C), 127.9 (CH), 126.2 (CH), 124.8 (CH), 120.0 (CH), 117.4 (CH), 30.3 (CH_2), 28.1 (CH_2), 23.7 (CH_2); Selected spectral data for the minor (*Z*) isomer: ^{1}H NMR (500 MHz, $CDCl_3$) δ 7.34 (dd, *J* = 2.0 Hz, 8.5 Hz, 1H), 7.27 (d, *J* = 2.0 Hz, 1H), 7.17 – 7.14 (m, 2H), 7.01 (d, *J* = 8.0 Hz, 2H), 6.90 – 6.87 (m, 1H), 6.28 (s, 1H), 2.92 (t, *J* = 6.5 Hz, 2H), 2.59 (dt, *J* = 1.5 Hz, 6.5 Hz, 2H), 2.04 (pent, *J* = 6.5 Hz, 2H); ^{13}C NMR (125 MHz, $CDCl_3$) δ 141.1 (C), 138.9 (C), 137.2 (C), 134.0 (C), 133.7 (CH), 132.6 (C), 129.0 (CH), 128.6 (CH), 128.0 (C), 124.8 (CH), 124.8 (CH), 120.1 (CH), 118.3 (CH), 117.2 (CH), 35.0 (CH_2), 29.7 (CH_2), 24.1 (CH_2); Selected spectral data for the mixture: δ IR (thin film): 3457, 3015, 2970, 2122, 1755, 1369, 1215, 747, 516 cm^{-1}; HRMS (EI) *m* / *z* calcd for $C_{17}H_{14}N_3Br$ $(M)^+$ 339.03710, found 339.03776.

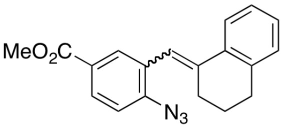

11h

Styryl Azide 11h. The general procedure was followed using 0.68 g of methyl 3-azido-4-formylbenzoate **s17** (3.3 mmol), 1.42 g of the phosphonium salt (3.0 mmol), 1.2 mL of a 2.5 M solution of BuLi in hexanes in 25 mL of THF. Purification by MPLC (1:50 EtOAc: hexanes) afforded the product as a white powder, as an 87:13 mixture of *E*- and *Z*-isomers (0.38 g, 40%). Selected spectral data for the major (*E*) isomer: ^{1}H NMR (500 MHz,

CDCl$_3$) δ 8.03 (d, *J* = 1.5 Hz, 1H), 7.98 (dd, *J* = 1.5 Hz, 8.0 Hz, 1H), 7.74 (dd, *J* = 3.5 Hz, 5.5 Hz, 1H), 7.23 – 7.20 (m, 3H), 7.15 – 7.14 (m, 1H), 6.98 (s, 1H), 3.94 (s, 3H), 2.87 (t, *J* = 6.5 Hz, 2H), 2.69 (dt, *J* = 1.5 Hz, 6.5 Hz, 2H), 1.86 (pent, *J* = 6.5 Hz, 2H); ^{13}C NMR (125 MHz, CDCl$_3$) δ 166.5 (C), 143.2 (C), 139.6 (C), 138.0 (C), 135.6 (C), 132.2 (CH), 129.9 (C), 129.3 (CH), 129.2 (CH), 127.8 (CH), 126.2 (CH), 126.1 (C), 124.6 (CH), 118.2 (CH), 117.7 (CH), 52.1 (CH$_3$), 30.3 (CH$_2$), 28.2 (CH$_2$), 23.7 (CH$_2$); Selected spectral data for the minor (*Z*) isomer: ^{1}H NMR (500 MHz, CDCl$_3$) δ 7.92 (dd, *J* = 3.0, 8.5 Hz, 1H), 7.83 (d, *J* = 3.0 Hz, 1H), 7.23 – 7.20 (m, 1H), 7.13 – 7.10 (m, 1H), 6.95 (d, *J* = 8.5 Hz, 1H), 6.80 (t, *J* = 6.5 Hz, 1H), 6.33 (s, 1H), 3.82 (s, 3H), 2.92 (t, *J* = 6.5 Hz, 2H), 2.60 (dt, *J* = 1.5 Hz, 6.5 Hz, 2H), 2.05 (pent, *J* = 6.5 Hz, 2H); ^{13}C NMR (125 MHz, CDCl$_3$) δ 166.3 (C), 142.6 (C), 140.9 (C), 138.9 (C), 135.5 (C), 134.2 (C), 132.7 (CH), 130.8 (CH), 129.0 (CH), 128.4 (CH), 126.4 (CH), 126.1 (C), 124.8 (CH), 118.7 (CH), 118.4 (CH), 52.0 (CH$_3$), 35.0 (CH$_2$), 29.7 (CH$_2$), 24.1 (CH$_2$); Selected spectral data for the mixture: δ IR (thin film): 3024, 2969, 2121, 1753, 1372, 1292, 762, 528 cm^{-1}. HRMS (EI) *m* / *z* calcd for $C_{19}H_{17}ON$ (M)$^+$ 291.12593, found 291.12443.

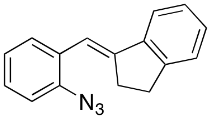

14a

Styryl Azide 14a. The general procedure was followed using 0.92 g of 2-azidobenzaldehyde **s9** (6.54 mmol), 2.40 g of the phosphonium salt (5.24 mmol), 2.3 mL of a 2.5 M solution of BuLi in hexanes in 15 mL of THF. Purification by MPLC (1:100 EtOAc: hexanes) afforded the product as a light yellow powder, as an 82:18 mixture of *E*- and *Z*-isomers (0.24 g, 19%): R$_f$ = 0.45 (18:82 EtOAc: hexane). Selected spectral data for the major (*E*) isomer: ^{1}H NMR (500 MHz, CDCl$_3$) δ 7.65 – 7.67 (m, 1H), 7.54 (dd, *J* = 1.5 Hz, 7.5 Hz, 1H), 7.10 – 7.35 (m, 7H), 3.00 – 3.08 (m, 4H); ^{13}C NMR (125 MHz, CDCl$_3$) δ 146.0 (C), 145.6 (C), 142.4 (C), 137.8 (C), 130.0 (C), 128.9 (CH), 128.5 (CH), 127.6 (CH), 126.8 (CH), 125.3 (CH), 124.5 (CH), 120.7 (CH), 118.4 (CH), 113.4 (CH), 30.8 (CH$_2$), 30.7 (CH$_2$); Selected spectral data for the minor (*Z*) isomer: ^{1}H NMR (500 MHz, CDCl$_3$) δ 7.43 (d, *J* = 8.0 Hz, 1H), 7.10 – 7.35 (m, 5H), 7.04 (d, *J* = 8.0 Hz, 1H), 6.92 – 6.95 (m, 1H) 6.47 (s, 1H), 3.00 – 3.08 (m, 2H), 2.94 – 2.97 (m, 2H); ^{13}C NMR (125 MHz, CDCl$_3$) δ 149.0 (C), 145.0 (C), 139.4 (C), 138.0 (C), 130.8 (CH), 130.1 (C), 129.0 (CH), 128.5 (CH), 125.9 (CH), 125.4 (CH), 124.7 (CH), 124.2 (CH), 118.7 (CH), 116.8 (CH), 33.9 (CH$_2$), 30.2 (CH$_2$); Selected spectral data for the mixture: IR (thin film): 2117, 2084, 1570, 1483, 1277, 1264, 749 cm^{-1}. HRMS (EI) *m* / *z* calcd for $C_{16}H_{13}N_3$ (M)$^+$ 247.11095, found 247.11013.

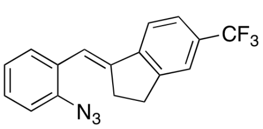

14b

Styryl Azide 14b. The general procedure was followed using 0.76 g of 2-azidobenzaldehyde **s9** (5.18 mmol), 2.48 g of the phosphonium salt (4.70 mmol), 2.1 mL of a 2.5 M solution of BuLi in hexanes in 15 mL of THF. Purification by MPLC (1:100 EtOAc: hexanes) afforded the product as a light yellow oil, as a 72:28 mixture of *E*- and *Z*-isomers (0.52 g, 35%): R$_f$ = 0.45 (18:82 EtOAc: hexane). Selected spectral data for the major (*E*) isomer: ^{1}H NMR (500 MHz, CDCl$_3$) δ 7.81 (d, J = 7.5 Hz, 1H), 7.51 – 7.54 (m, 2H), 7.30 -7.39 (m, 2H), 7.11 – 7.23 (m, 3H), 3.18 – 3.24 (m, 2H), 3.05 – 3.08 (m, 2H); ^{13}C NMR (125 MHz, CDCl$_3$) δ 144.3 (C), 143.7 (C), 143.5 (C), 143.3 (C), 138.0 (C), 129.4 (C), 128.9 (CH), 128.1 (CH), 127.1 (CH), 125.1 (q, J_{CF} = 4.0 Hz, CH), 124.6 (CH), 124.2 (q, J_{CF} = 271.9, C), 123.9 (CH), 118.4 (CH), 115.0 (CH), 30.4 (CH$_2$), 29.6 (CH$_2$); Selected spectral data for the minor (*Z*) isomer: ^{1}H NMR (500 MHz, CDCl$_3$) δ 7.43 (d, *J* = 7.5 Hz, 1H), 7.30 – 7.39 (m,

2H), 7.11 – 7.23 (m, 2H), 7.13 (dd, J = 0.5 Hz, 7.5 Hz, 1H), 7.05 (t, J = 7.5 Hz, 1H), 6.57 (s, 1H), 3.18 – 3.24 (m, 2H), 2.99 – 3.30 (m, 2H); ^{13}C NMR (125 MHz, $CDCl_3$) δ 146.5 (C), 141.3 (C), 138.1 (C), 130.5 (CH), 129.5 (C), 128.8 (CH), 127.5 (C), 127.4 (CH), 127.2 (C), 126.3 (CH), 125.1 (q, J_{CF} = 4.0 Hz, CH), 124.8 (CH), 118.8 (CH), 118.5 (CH), 33.5 (CH_2), 28.8 (CH_2); ^{19}F NMR ($CDCl_3$, 282 MHz) δ –63.12; Selected spectral data for the mixture: IR (thin film): 2124, 2083, 1595, 1318, 1160, 1118, 750 cm^{-1}. HRMS (EI) m / z calcd for $C_{17}H_{12}N_3F_3$ (M)$^+$ 315.09833, found 315.09885.

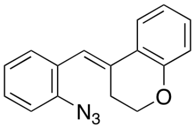

14d

Styryl Azide 14d. The general procedure was followed using 0.65 g of 2-azidobenzaldehyde **s9** (4.42 mmol), 1.87 g of the phosphonium salt (3.95 mmol), 1.73 mL of a 2.5 M solution of BuLi in hexanes in 20 mL of THF. Purification by MPLC (1:100 EtOAc: hexanes) afforded the product as a white powder, as an 83:17 mixture of E- and Z-isomers (0.28 g, 27%): R_f = 0.45 (18:82 EtOAc: hexane). Selected spectral data for the major (E) isomer: ^{1}H NMR (500 MHz, $CDCl_3$) δ 7.70 (dd, J = 1.5 Hz, 8.0 Hz, 1H), 7.32 – 7.35 (m, 1H), 7.27 (d, J = 8.0 Hz, 1H), 7.19 – 7.22 (m, 2H), 7.14 – 7.17 (m, 1H), 7.08 (s, 1H), 6.95 – 6.98 (m, 1H), 6.83 – 6.85 (m, 1H), 4.20 (t, J = 6.0 Hz, 2H), 2.80 – 2.83 (m, 2H); ^{13}C NMR (125 MHz, $CDCl_3$) δ 154.9 (C), 138.7 (C), 132.1 (C), 130.9 (CH), 129.5 (CH), 128.8 (C), 128.3 (CH), 124.8 (CH), 124.3 (CH), 122.4 (C), 120.9 (CH), 118.4 (CH), 117.5 (CH), 117.0 (CH), 66.2 (CH_2), 26.9 (CH_2); Selected spectral data for the minor (Z) isomer: ^{1}H NMR (500 MHz, $CDCl_3$): δ 7.38 (d, J = 7.5 Hz, 1H), 7.29 – 7.30 (m, 1H), 7.09 – 7.11 (m, 1H), 7.00 – 7.04 (m, 2H), 6.84 (dd, J = 1.0 Hz, 8.0 Hz, 1H), 6.65 – 6.58 (m, 1H), 6.29 (s, 1H), 4.38 (t, 5.5 Hz, 2H), 2.71- 2.73 (m, 2H); ^{13}C NMR (125 MHz, $CDCl_3$) 154.9 (C) 138.1 (C), 132.5 (C), 131.1 (CH), 129.7 (CH), 128.53 (CH), 128.50 (CH), 124.7 (CH), 120.6 (C), 120.0 (CH), 119.3 (CH), 118.6 (CH), 117.1 (CH), 67.4 (CH_2), 33.3 (CH_2); Selected spectral data for the mixture: δ IR (thin film): 2117, 2091, 1603, 1572, 1468, 1450, 1281, 1049, 749 cm^{-1}. HRMS (EI) m / z calcd for $C_{16}H_{13}ON_3$ (M)$^+$ 263.10587, found 263.10672.

II. Preparation of Substrates using a Horner–Wadsworth–Emmons Reaction.

A. General Procedure for the Horner–Wadsworth–Emmons Reaction and Fe-Mediated Nitro-Group Reduction

2-Amino substituted styrenes were synthesized via a two-step procedure of Horner-Wadsworth-Emmons reaction followed by nitro-group reduction. Yields were not optimized.

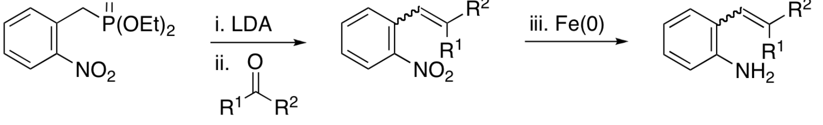

(s4)

To a –78 °C solution of LDA (4.4 mmol) in 10 mL of THF was added dropwise *ortho*-nitrobenzylphosphonate in 3 mL of THF. After an additional hour, a solution of the ketone in 5 mL of THF was added slowly to the mixture at –78 °C. The reaction mixture was allowed to warm to ambient temperature slowly. After 1h, 10 mL water was added to quench the reaction. The resulting mixture was taken up by dichloromethane and separated. The aqueous phase was washed with 3 × 5 mL dichloromethane. The resulting organic phase was washed with

20 mL of brine and dried over Na_2SO_4. After filtration, the filtrate was concentrated *in vacuo* to produce 2-nitro-styrene, which was submitted to the subsequent Fe-mediated nitro-group reduction without further purification.

To a solution of nitro-substituted styrene (prepared from 4.0 mmol of ortho-nitrobenzylphophonate) in 20 mL of AcOH and 20 mL of EtOH was added 1.8 g of Fe powder (32 mmol). The resultant mixture was allowd to reflux at 80 °C overnight. After cooled down to ambient temperature, the resulting mixture was filtrated with a pad of Celite. The filtrate was diluted with 50 mL of water and washed with 3 × 10 mL dichloromethane. The resulting organic phase was washed with 40 mL of brine and dried over Na_2SO_4. After filtration, the filtrate was concentrated in *vacuo*. Purification of the reaction mixture using MPLC afforded the product

B. Synthesis of *ortho*-Amino-Substituted Styrenes.

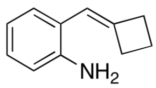

s18

Styrene s18. The general procedure of Horner–Wadsworth–Emmons reaction was followed using 1.09 g of *ortho*-nitrobenzylphosphonate[6] (4.0 mmol), 0.45 mL of cyclobutanone (6.0 mmol) and LDA (4.4 mmol) in 6 mL of THF. After work-up, the representative pathway of nitro-group reduction was followed by using 1.79 g of Fe (32.0 mmol), in 20.0 mL of AcOH and 20.0 mL of EtOH. Purification by MPLC (1:100 – 20:80 EtOAc: hexanes) afforded the product as a light yellow oil (0.25 g, 39% two steps): ^{1}H NMR (500 MHz, $CDCl_3$) δ 7.16 – 7.19 (m, 1H), 7.07 – 7.11 (m, 1H), 6.72 (d, J = 8.0 Hz, 1H), 6.79 – 6.83 (m, 1H), 6.11 (s, 1H), 3.71 (s, 2H), 2.92 – 2.99 (m, 4H), 2.10 – 2.16 (m, 2H); ^{13}C NMR (125 MHz, $CDCl_3$) δ 145.2 (C), 143.5 (C), 128.2 (CH), 127.3 (CH), 123.4 (C), 118.5 (CH), 115.8 (CH), 115.7 (CH), 32.6 (CH_2), 32.3 (CH_2), 18.2 (CH_2); IR (thin film): 3451, 3366, 2948, 1614, 1488 1297, 1263, 744cm^{-1}. HRMS (EI) *m* / *z* calcd for $C_{11}H_{13}N$ $(M)^+$ 159.10480, found 159.10560.

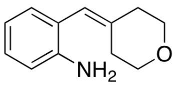

s19

Styrene s19. The general procedure of Horner–Wadsworth–Emmons reaction was followed using 1.09 g of *ortho*-nitrobenzylphosphonate[6] (4.0 mmol), 0.55 mL of tetrahydro-4*H*-pyran-4-one (6.0 mmol) and LDA (4.4 mmol) in 6 mL of THF. After work-up, the representative pathway of nitro-group reduction was followed by using 1.79 g of Fe (32.0 mmol), in 20.0 mL of AcOH and 20.0 mL of EtOH. Purification by MPLC (1:100 – 20:80 EtOAc: hexanes) afforded the product as a light yellow oil (0.50 g, 66% two steps): ^{1}H NMR (500 MHz, $CDCl_3$) 7.07 (t, J = 8.0 Hz, 1H), 6.99 (d, J = 7.5 Hz, 1H), 6.69 – 6.75 (m, 2H), 6.14 (s, 1H), 3.80 (t, J = 5.5 Hz, 2H), 3.69 (s, 2H), 3.66 (t, J = 5.5 Hz, 2H), 2.43 (t, J = 5.5 Hz, 2H), 2.36 (t, J = 5.5 Hz, 2H); ^{13}C NMR (125 MHz, $CDCl_3$) δ 144.2 (C), 139.8 (C), 130.1 (CH), 127.9 (CH), 122.9 (C), 119.6 (CH), 118.1 (CH), 115.1 (CH), 69.7 (CH_2), 69.0 (CH_2), 37.0 (CH_2), 31.0 (CH_2); IR (thin film): 3459, 3355, 1613, 1489, 1452, 1229, 1093, 995, 746 cm^{-1}. HRMS (EI) *m* / *z* calcd for $C_{12}H_{15}ON$ $(M)^+$ 189.11537, found 189.11613.

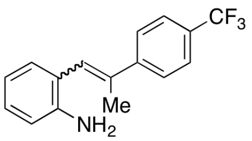

s20

Styrene s20. The general procedure of Horner–Wadsworth–Emmons reaction was followed using 1.09 g of *ortho*-nitrobenzylphosphonate[6] (4.0 mmol), 0.83 g of 4-acetylbenzotrifluoride (4.4 mmol) and LDA (4.4 mmol) in 6 mL of THF. After work-up, the representative pathway of nitro-group reduction was followed by using 1.79 g of Fe (32.0 mmol), in 20.0 mL of AcOH and 20.0 mL of EtOH. Purification by MPLC (1:100 – 20:80 EtOAc: hexanes) afforded the crude product as a brown oil (0.34 g, 31% two steps). The aniline was carried on to the azidation reaction without further purification.

C. General Procedure for the Azidation Reaction.

Following the procedure of Tor and co-workers,[7] the aniline group was transformed into an azide (s5). Yields were not optimized.

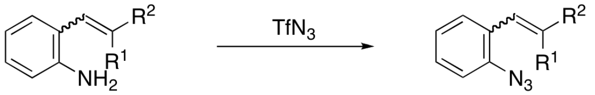

(s5)

To a solution of aniline (3 mmol) in 5 mL of CH_2Cl_2 was added subsequently 24 mg of $CuSO_4$, 1.2 mL of Et_3N, freshly prepared triflyl azide (9 mmol) in 15 mL of CH_2Cl_2, 1 mL of water and 2 mL of MeOH. The resulting mixture was allowed to react at room temperature overnight. Then, the reaction mixture was taken up by 15 mL dichloromethane, neutralized with a saturated aq. soln. of $NaHCO_3$ and washed with 3 × 10 mL of CH_2Cl_2. The resulting organic phase was washed with 20 mL of brine and dried over Na_2SO_4. After filtration, the filtrate was concentrated *in vacuo*. Purification of the reaction mixture using MPLC afforded the product.

D. Synthesis of Styryl Azides.

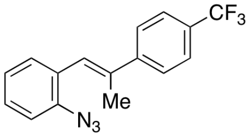

14f

Styryl Azide 14f. The general procedure for azidation was followed using 0.31 g of aniline **s20** (1.1 mmol), 0.01 g of $CuSO_4$, freshly prepared dichloromethane solution of TfN_3 (from 3.0 mmol of Tf_2O), and 0.4 mL of Et_3N (3.0 mmol) in 4 mL CH_2Cl_2, 2 mL of MeOH and 1 mL of H_2O. Purification by MPLC (1:100 EtOAc: hexanes) afforded the product as a light yellow solid (0.22 g, 66%): mp 62 - 65 °C; ^{1}H NMR (500 MHz, $CDCl_3$) δ 7.51 (d, J = 8.0 Hz, 2H), 7.28 (d, J = 8.0 Hz, 2H), 7.17 – 7.20 (m, 1H), 7.12 (dd, J = 1.0 Hz, 8.0 Hz, 1H), 6.80 – 6.83 (m, 1H), 6.74 (d, J = 7.5 Hz, 1H), 6.65 (s, 1H), 2.29 (s, 3H); ^{13}C NMR (125 MHz, $CDCl_3$) δ 145. 3 (C), 138.6 (C), 138.1 (C), 131.1 (CH), 129.076 (q, J_{CF} = 32.6 Hz, C), 129.075 (C), 128.7 (CH), 128.1 (CH), 125.2 (q, J_{CF} = 3.5 Hz, CH), 124.3 (CH), 124.2 (q, J_{CF} = 271 Hz, C), 123.5 (CH), 118.2 (CH), 23.0 (CH_3); ^{19}F NMR ($CDCl_3$, 282 MHz) δ –63.04; IR (thin film): 2122, 1615, 1482, 1323, 1123, 738 cm^{-1}. HRMS (EI) m / z calcd for $C_{16}H_{12}N_3F_3$ $(M)^+$ 303.09833, found 303.09697.

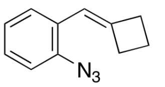

17a

Styryl Azide 17a. The general procedure for azidation was followed using 0.23 g of aniline **s18** (1.44 mmol), 0.02 g of $CuSO_4$, freshly prepared dichloromethane solution of TfN_3 (from 4.5 mmol of Tf_2O), and 0.6 mL of Et_3N (4.5 mmol) in 6 mL CH_2Cl_2, 3 mL of MeOH and 2 mL of H_2O. Purification by MPLC (100% hexanes) afforded the product as a light yellow oil (0.14 g, 54%): ^{1}H NMR (500 MHz, $CDCl_3$) δ 7.30 (d, J = 8.0 Hz, 1H), 7.23 (t, J = 8.0 Hz, 1H), 7.15 (d, J = 8.0 Hz, 1H), 7.11 (t, J = 7.5 Hz, 1H), 6.35 (s, 1H), 3.01 – 3.04 (m, 2H), 2.92 – 2.95 (m, 2H), 2.12 – 2.16 (m, 2H); ^{13}C NMR (125 MHz, $CDCl_3$) δ 146.5 (C), 136.5 (C), 129.5 (C), 127.9 (CH), 127.1 (CH), 124.5 (CH), 118.3 (CH), 115.1 (CH), 32.9 (CH_2), 32.7 (CH_2), 18.4 (CH_2); IR (thin film): 2951, 2113, 1482, 1285, 1099, 743, 665 cm^{-1}. HRMS (EI) m / z calcd for $C_{11}H_{11}N_3$ $(M)^+$ 185.09530, found 185.09451.

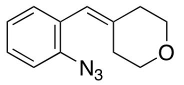

17h

Styryl Azide 17h. The general procedure for azidation was followed using 0.43 g of aniline **s19** (2.28 mmol), 0.02 g of $CuSO_4$, freshly prepared dichloromethane solution of TfN_3 (from 9.0 mmol of Tf_2O), and 1.3 mL of Et_3N (9.0 mmol) in 6 mL CH_2Cl_2, 3 mL of MeOH and 2 mL of H_2O. Purification by MPLC (1:100 EtOAc: hexanes) afforded the product as a light yellow oil (0.31 g, 63%): ^{1}H NMR (500 MHz, $CDCl_3$) 7.22 – 7.25 (m, 1H), 7.10 – 7.15 (m, 2H), 7.07 (t, J = 7.5 Hz, 1H), 6.24 (s, 1H), 3.77 (t, J = 5.5 Hz, 2H), 3.64 (t, J = 5.5 Hz, 2H), 2.37 – 2.41 (m, 4H); ^{13}C NMR (125 MHz, $CDCl_3$) δ 139.3 (C), 138.1 (C), 130.8 (CH), 129.1 (C), 128.0 (CH), 124.3 (CH), 119.3 (CH), 118.4 (CH), 69.4 (CH_2), 68.6 (CH_2), 37.2 (CH_2), 30.9 (CH_2); IR (thin film): 2115, 2082, 1482, 1279, 1230, 1097, 997, 748, 665 cm^{-1}. HRMS (EI) m / z calcd for $C_{12}H_{13}ON_3$ $(M)^+$ 215.10587, found 215.10709.

III. Preparation of Substrates using a Suzuki Reaction.

General Procedure for the Arylboronic Acid Pinacol Ester Syntheses.

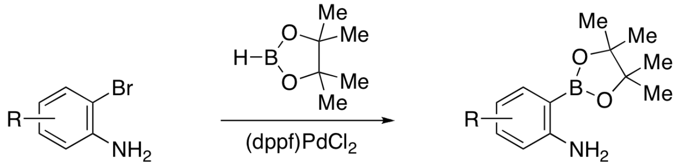

(s6)

To a mixture of 0.85 g of 2-bromo-aniline (5.0 mmol), 2.09 mL of Et_3N (20.0 mmol), 0.185 g of $(dppf)PdCl_2$ (0.25 mmol) in 15 mL 1,4-dioxane, was added dropwise 2.20 mL 4,4,5,5-tetramethyl-1,3,2-dioxaborolane (15.0 mmol). The resultant mixture was heated to 100 °C. After 12h, the mixture was cooled to room temperature and diluted with 20 mL of NH_4Cl. The resulting aqueous phase was extracted with an additional 2 × 20 mL of CH_2Cl_2. The combined organic phases were washed with 1 × 30 mL of brine. The resulting organic phase was dried over Na_2SO_4, and was concentrated *in vacuo*. Purification via MPLC afforded the product.

A. Syntheses of Arylboronic Acid Pinacol Esters.

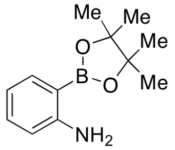

s21

Aniline s21.[8] The general procedure was followed using 3.4 g of 2-bromo-aniline (20.0 mmol), 8.7 mL of 4,4,5,5-tetramethyl-1,3,2-dioxaborolane (60.0 mmol), 0.82 g of (dppf)$PdCl_2$ (1.0 mmol), and 8.4 mL of Et_3N (80.0 mmol) in 100 mL of 1, 4-dioxane. Purification by MPLC (2:100 – 10:90 EtOAc: hexanes) afforded the product as a light yellow solid (3.02 g, 69%): mp 62 -64 °C; The spectral data matched that reported by Driver and co-workers:[8] ^{1}H NMR (500 MHz, $CDCl_3$) δ 7.64 (d, J = 7.5 Hz, 1H), 7.24 (t, J = 7.0 Hz, 1H), 6.70 (t, J = 7.0 Hz, 1H), 6.61 (d, J = 8.5 Hz, 1H), 4.76 (s, 2H), 1.36 (s, 12H); ^{13}C NMR (125 MHz, $CDCl_3$) δ 153.7 (C), 136.8 (CH), 132.8 (CH), 116.9 (CH), 114.8 (CH), 83.5 (C), 25.0 (CH_3); IR (thin film): 3486, 3380, 1624, 1605, 1352, 1311, 1244, 1135, 1086, 847, 758, 654 cm^{-1}.

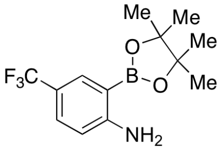

s22

Aniline s22.[8] The general procedure was followed using 2.65 g of 2-bromo-4-(trifluoromethyl)aniline (10.0 mmol), 4.4 mL of 4,4,5,5-tetramethyl-1,3,2-dioxaborolane (30.0 mmol), 0.41 g of (dppf)$PdCl_2$ (0.5 mmol), and 4.2 mL of Et_3N (40.0 mmol) in 50 mL of 1, 4-dioxane. Purification by MPLC (2:100 – 10:90 EtOAc: hexanes) afforded the product as a brown solid (1.81 g, 63%): mp 107 -109 °C. The spectral data matched that reported by Driver and co-workers:[8] ^{1}H NMR (500 MHz, $CDCl_3$) δ 7.86 (s, 1H), 7.41 (d, J = 8.5 Hz, 1H), 6.59 (d, J = 8.5 Hz, 1H), 5.08 (s, 2H), 1.35 (s, 12H); ^{13}C NMR (125 MHz, $CDCl_3$) δ 156.1 (C), 134.3 (q, J_{CF} = 3.8 Hz, CH), 129.5 (q, J_{CF} = 3.8 Hz, CH), 125.0 4 (q, J_{CF} = 268.8 Hz, C), 118.5 (q, J_{CF} = 32.5 Hz, C), 84.0 (C) 114.2 (CH), 24.9 (CH_3); ^{19}F NMR ($CDCl_3$, 282 MHz) δ –61.64; IR (thin film): 3472, 3375, 1625, 1370, 1304, 1256, 1140, 1097, 1072, 832.9 cm^{-1}.

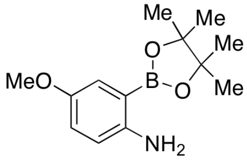

s23

Aniline s23.[8] The general procedure was followed using 2.02 g of 2-bromo-4-methoxyaniline (10.0 mmol), 4.4 mL of 4,4,5,5-tetramethyl-1,3,2-dioxaborolane (30.0 mmol), 0.41 g of (dppf)$PdCl_2$ (0.5 mmol), and 4.2 mL of Et_3N (40.0 mmol) in 50 mL of 1, 4-dioxane. Purification by MPLC (5:100 – 10:90 EtOAc: hexanes) afforded the product as a brown liquid (1.52 g, 61%). The spectral data matched that reported by Driver and co-workers:[8] ^{1}H NMR (500 MHz, $CDCl_3$) 7.15 (d, J = 3.5 Hz, 1H), 6.85 (dd, J = 3.0 Hz, 8.5 Hz, 1H), 6.58 (d, J = 8.5 Hz, 1H), 4.48 (s, 2H), 3.76 (s, 3H), 1.34 (s, 12H); ^{13}C NMR (125 MHz, $CDCl_3$) δ 151.3 (C), 147.9 (C), 120.7 (CH), 119.5 (CH), 116.5 (CH), 83.6 (C), 56.0 (CH_3), 25.0 (CH_3); IR (thin film): 3456, 3366, 1494, 1421, 1359, 1304, 1226, 1037, 855, 829, 750 cm^{-1}. HRMS (EI) m / z calcd for $C_{13}H_{20}O_3NB$ $(M)^+$ 249.1536, found 249.1540.

B. General Procedure for the Triflate Formation.

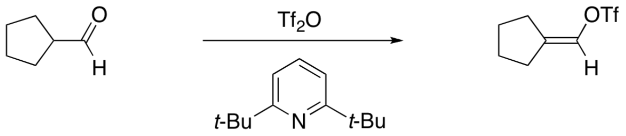

(s7)

To a mixture of 10.0 mmol of cyclopentanecarboxaldehyde and 2.65 mL 2,6-di-*tert*-butylpyridine (12.0 mmol) in 40 mL of 1,2-dichloroethane was added 1.85 mL of triflic anhydride (11.0 mmol). The resultant mixture was heated to 70 °C. After 2h, the mixture was cooled to room temperature and diluted with 40 mL CH_2Cl_2. The resulting aqueous phase was extracted with an additional 2 × 30 mL of CH_2Cl_2. The combined organic phases were washed with 1 × 30 mL of brine. The resulting organic phase was dried over Na_2SO_4, and was concentrated *in vacuo* to afford crude triflate.

C. General Procedure for the Suzuki Reaction.

Following the procedure of Driver and co-workers,[8] was aniline **s6** treated with vinyl triflate **s7** in the presence of (dppf)$PdCl_2$ to produce the desired aniline (s8). Yields were not optimized.

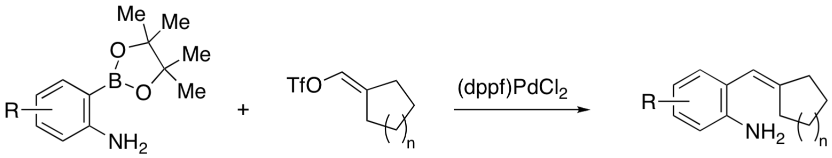

(s8)

To a mixture of 0.919 g of boronic ether **s6** (3.2 mmol), 0.261 g of (dppf)$PdCl_2$ (0.32 mmol) in 40 mL 1,4-dioxane was added 8 mL of a 3M solution of NaOH in water followed by 1.36 g of crude triflate **s7** (5.12 mmol). The resultant mixture was heated to 80 °C. After 12 h, the mixture was cooled to room temperature and filtrated through a pad of Celite. The filtrate was diluted with 20 mL saturated NH_4Cl and extracted with an additional 2 × 30 mL of CH_2Cl_2. The combined organic phases were washed with 1 × 30 mL of brine. The resulting organic phase was dried over Na_2SO_4, and was concentrated *in vacuo*. Purification via MPLC afforded the product.

D. Synthesis of Anilines.

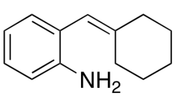

s24

Aniline s24. The general procedure was followed using 1.10 g of boronic ester **s6** (5.0 mmol), crude vinyl triflate (derived from 10.0 mmol of cyclohexanecarboxaldehyde), 0.82 g of (dppf)$PdCl_2$ (1.0 mmol), and 1.8 g of NaOH (45.0 mmol) in 75 mL of 1,4-dioxane and 15 mL of water. Purification by MPLC (2:100 – 10:90 EtOAc: hexanes) afforded the product as a light yellow oil (0.74 g, 79%): ^{1}H NMR (500 MHz, $CDCl_3$) δ 7.09 (t, J = 6.0 Hz, 1H), 7.04 (d, J = 7.5 Hz, 1H), 6.77 (t, J = 7.5 Hz, 1H), 6.72 (d, J = 8.0 Hz, 1H), 6.05 (s, 1H), 3.70 (s, 2H), 2.33 (t, J = 6.0 Hz, 2H), 2.24 (t, J = 6.0 Hz, 2H), 1.56 – 1.70 (m, 6H); ^{13}C NMR (125 MHz, $CDCl_3$) δ 145.4 (C), 144.3 (C), 130.3 (CH), 127.5 (CH), 124.0 (C), 118.0 (CH), 117.8 (CH), 115.0 (CH), 37.2 (CH_2), 30.0

(CH_2), 28.9 (CH_2), 28.1 (CH_2), 26.8 (CH_2); IR (thin film): 3466, 3368, 1161, 1489, 1448, 1297, 1265, 745 cm^{-1}. HRMS (EI) *m / z* calcd for $C_{13}H_{17}N$ $(M)^+$ 187.13610, found 187.13596.

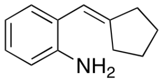

s25

Aniline s25. The general procedure was followed using 2.19 g of boronic ester **s6** (10.0 mmol), crude vinyl triflate (derived from 15.0 mmol of cyclopentenanecarboxaldehyde), 0.82 g of (dppf)$PdCl_2$ (1.0 mmol), and 3.6 g of NaOH (90.0 mmol) in 100 mL of 1, 4-dioxane and 30 mL of water. Purification by MPLC (2:100 – 10:90 EtOAc: hexanes) afforded the product as a light yellow oil (1.54 g, 89%): ^{1}H NMR (500 MHz, $CDCl_3$) δ 7.16 (d, *J* = 7.5 Hz, 1H), 7.05 (dt, *J* = 1.5 Hz, 7.5 Hz, 1H), 6.76 (t, *J* = 7.5 Hz, 1H), 6.70 (d, *J* = 7.5 Hz, 1H), 6.23 (s, 1H), 3.67 (s, 2H), 2.49 (s, 2H), 2.39 (s, 2H), 1.70 – 1.73 (m, 4H); ^{13}C NMR (125 MHz, $CDCl_3$) δ 148.6 (C), 143.7 (C), 129.1 (CH), 127.3 (CH), 124.9 (C), 118.2 (CH), 115.7 (CH), 115.2 (CH), 34.6 (CH_2), 30.7 (CH_2), 26.7 (CH_2), 25.8 (CH_2); IR (thin film): 3452, 3368, 1613, 1488, 1453, 1295, 1263, 1155, 871, 744 cm^{-1}. HRMS (EI) *m / z* calcd for $C_{12}H_{15}N$ $(M)^+$ 173.1164, found 173.1204.

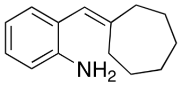

s26

Aniline s26. The general procedure was followed using 0.76 g of boronic ester **s6** (3.5 mmol), crude vinyl triflate (derived from 10.0 mmol of cycloheptanecarboxaldehyde), 0.25 g of (dppf)$PdCl_2$ (0.3 mmol), and 1.2 g of NaOH (30.0 mmol) in 40 mL of 1, 4-dioxane and 10 mL of water. Purification by MPLC (2:100 – 10:90 EtOAc: hexanes) afforded the product as a light yellow oil (0.25 g, 35%): ^{1}H NMR (500 MHz, $CDCl_3$) δ 7.07 (dt, *J* = 1.5 Hz, 7.5 Hz, 1H), 7.03 (d, *J* = 7.5 Hz, 1H), 6.74 (dt, *J* = 1.0 Hz, 7.5 Hz, 1H), 6.71 (dd, *J* = 1.5 Hz, 7.5 Hz, 1H), 6.10 (s, 1H), 3.68 (s, 2H), 2.43 – 2.45 (m, 2H), 2.31 – 2.33 (m, 2H) 1.68 – 1.72 (m, 2H), 1.52 – 1.62 (m, 6H); ^{13}C NMR (125 MHz, $CDCl_3$) δ 147.5 (C), 144.0 (C), 130.0 (CH), 127.4 (CH), 124.5 (C), 121.0 (CH), 118.0 (CH), 114.9 (CH), 37.8 (CH_2), 31.2 (CH_2), 29.9 (CH_2), 29.5 (CH_2), 29.37 (CH_2), 27.3 (CH_2); IR (thin film): 3464, 3372, 2918, 2849, 1611, 1489, 1451, 745 cm^{-1}. HRMS (EI) *m / z* calcd for $C_{14}H_{19}N$ $(M)^+$ 201.15175 found 201.15198

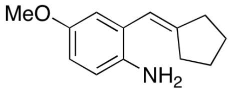

s27

Aniline s27. The general procedure was followed using 1.69 g of boronic ester **s6** (6.8 mmol), crude vinyl triflate (derived from 10.0 mmol of cyclopentanecarboxaldehyde), 0.57 g of (dppf)$PdCl_2$ (0.68 mmol), and 1.44 g of NaOH (36.0 mmol) in 40 mL of 1, 4-dioxane and 12 mL of water. Purification by MPLC (2:100 – 20:80 EtOAc: hexanes) afforded the product as a brown oil (0.97 g, 70%): ^{1}H NMR (500 MHz, $CDCl_3$) δ (500 MHz, $CDCl_3$): δ 6.77 (d, *J* = 1.8 Hz, 1H), 6.64 – 6.64 (m, 2H), 6.23 (t, *J* = 2.3 Hz, 1H), 3.75 (s, 3H), 3.44 (s, 2H), 2.48 (ddd, *J* = 7.7, 5.5, 1.9 Hz, 2H), 2.42-2.40 (m, 2H), 1.71 (m, 4H); ^{13}C NMR (125 MHz, $CDCl_3$) δ 152.4 (C), 148.8 (C), 137.5 (C), 126.2 (C), 116.4 (CH), 115.9 (CH), 114.6 (CH), 112.9 (CH), 55.8 (CH_3), 34.7 (CH_2), 30.8 (CH_2), 26.7 (CH_2), 25.8 (CH_2); IR (thin film): 3437, 3357, 2947, 1604, 1494, 1281, 1040, 904, 813 cm^{-1}. HRMS (EI) *m / z* calcd for $C_{13}H_{17}ON$ $(M)^+$ 203.13102, found 203.13075.

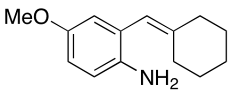

s28

Aniline s28. The general procedure was followed using 1.44 g of boronic ester **s6** (5.8 mmol), crude vinyl triflate (derived from 9.0 mmol of cyclohexanecarboxaldehyde), 0.57 g of (dppf)$PdCl_2$ (0.68 mmol), and 1.20 g of NaOH (30.0 mmol) in 40 mL of 1, 4-dioxane and 10 mL of water. Purification by MPLC (2:100 – 20:80 EtOAc: hexanes) afforded the product as a brown oil (0.21 g, 17%): ^{1}H NMR (500 MHz, $CDCl_3$): 6.60 – 6.68 (m, 3H), 6.01 (s, 1H), 3.74 (s, 3H), 3.13 (s, 2H), 2.29 (t, J = 6.0 Hz, 2H), 2.21 (t, J = 6.5 Hz, 2H), 1.52 – 1.66 (m, 6H); ^{13}C NMR (125 MHz, $CDCl_3$) δ 152.2 (C), 145.6 (C), 138.0 (C), 125.3 (C), 117.7 (CH), 116.1 (CH), 115.7 (CH), 113.1 (CH), 55.8 (CH_3), 37.2 (CH_2), 30.0 (CH_2), 28.8 (CH_2), 28.0 (CH_2), 26.7 (CH_2); IR (thin film): 3438, 3358, 1494, 1276, 1216, 1159, 1039, 811 cm^{-1}. HRMS (EI) m / z calcd for $C_{14}H_{19}ON$ $(M)^+$ 217.1467, found 217.1466.

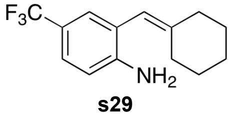

s29

Aniline s29. The general procedure was followed using 3.01 g of boronic ester **s6** (10.5 mmol), crude vinyl triflate (derived from 15.0 mmol corresponding aldehyde), 0.87 g of (dppf)$PdCl_2$ (1.05 mmol), and 3.60 g of NaOH (90.0 mmol) in 110 mL of 1, 4-dioxane and 30 mL of water. Purification by MPLC (2:100 – 10:90 EtOAc: hexanes) afforded the product as a brown liquid (1.82 g, 62%): ^{1}H NMR (500 MHz, $CDCl_3$) δ 7.30 (d, J = 8.5 Hz, 1H), 7.26 (s, 1H), 6.69 (d, J = 8.5 Hz, 1H), 5.98 (s, 1H), 4.01 (s, 2H), 2.32 (t, J = 5.5 Hz, 2H), 2.18 (t, J = 6.5 Hz, 2H), 1.54 – 1.71 (m, 6H); ^{13}C NMR (125 MHz, $CDCl_3$) δ 147.3 (C), 147.1 (C), 127.3 (q, J_{CF} = 3.8 Hz, CH), 125.1 (q, J_{CF} = 268.8 Hz, C), 124.6 (q, J_{CF} = 3.8 Hz, CH), 123.3 (C), 119.5 (q, J_{CF} = 31.3 Hz, C), 116.4 (CH), 114.1 (CH), 37.1 (CH_2), 30.0 (CH_2), 28.8 (CH_2), 28.0 (CH_2), 26.6 (CH_2); ^{19}F NMR ($CDCl_3$, 282 MHz) δ –61.47; IR (thin film): 3492, 3398, 1620, 1323, 1142, 1103, 1071, 820 cm^{-1}. HRMS (EI) m / z calcd for $C_{14}H_{16}NF_3$ $(M)^+$ 255.1235, found 255.1239.

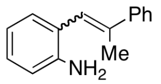

s30

Aniline s30. The general procedure was followed using 1.75 g of boronic ester **s6** (8.0 mmol), crude vinyl triflate (derived from 15.0 mmol corresponding aldehyde), 0.66 g (dppf)$PdCl_2$ (0.8 mmol), and 3.60 g of NaOH (90.0 mmol) in 110 mL of 1,4-dioxane and 30 mL of water. Purification by MPLC (1:100 – 10:90 EtOAc: hexanes) afforded the product as a light yellow oil, as a 69:31 mixture of *Z*- and *E*-isomers (1.41 g, 84%): R_f= 0.45 (18:82 EtOAc: hexane). Selected spectral data for the major (*Z*) isomer: ^{1}H NMR (500 MHz, $CDCl_3$) δ 7.11 – 7.23 (m, 5H), 6.97 (t, J = 8.0 Hz, 1H), 6.72 – 6.76 (m, 1H), 6.62 (d, J = 8.0 Hz, 1H), 6.52 (t, J = 7.5 Hz, 1H), 6.41 (s, 1H), 3.70 (br, 2H), 2.28 (s, 3H); ^{13}C NMR (125 MHz, $CDCl_3$) δ 144.0 (C), 141.2 (C), 139.5 (C), 130.0 (CH), 128.1 (CH), 127.5 (CH), 127.0 (CH), 123.98 (C), 123.1 (CH), 118.3 (CH), 115.3 (CH), 25.7 (CH_3) one overlapping aromatic C not distinguishable; Selected spectral data for the minor (*E*) isomer: ^{1}H NMR (500 MHz, $CDCl_3$) δ 7.57 – 7.58 (m, 2H), 7.38 – 7.41 (m, 2H), 7.31 – 7.34 (m, 1H), 7.11- 7.23 (m, 2H), 6.81 (t, J = 7.5 Hz, 1H), 6.72 – 6.76 (m, 2H), 3.70 (br s, 2H), 2.17 (s, 3H); ^{13}C NMR (125 MHz, $CDCl_3$) δ 144.4 (C), 142.9 (C), 139.1 (C), 130.5 (CH), 128.4 (CH), 128.1 (CH), 127.4 (CH), 124.01 (C), 123.9 (CH), 123.5 (CH), 118.2 (CH), 115.2 (CH), 17.2 (CH_3); Selected spectral data for the mixture: δ IR (thin film): 3464, 3374, 1613, 1489, 1454, 1275, 1264 cm^{-1}. HRMS (EI) m / z calcd for $C_{15}H_{15}N$ $(M)^+$ 209.12045, found 209.12206.

E. General Procedure for Styryl Azides Syntheses.

Following the procedure of Tor and co-workers,[7] the aniline group was transformed into an azide (s5). Yields were not optimized.

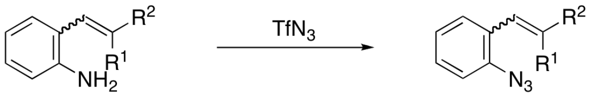

(s5)

To a solution of aniline (3 mmol) in 5 mL of CH_2Cl_2 was added subsequently 24 mg of $CuSO_4$, 1.2 mL of Et_3N, freshly prepared triflyl azide (9 mmol) in 15 mL of CH_2Cl_2, 1 mL of water and 2 mL of MeOH. The resulting mixture was allowed to react at room temperature overnight. Then, the reaction mixture was taken up by 15 mL dichloromethane, neutralized with a saturated aq. soln. of $NaHCO_3$ and washed with 3 × 10 mL of CH_2Cl_2. The resulting organic phase was washed with 20 mL of brine and dried over Na_2SO_4. After filtration, the filtrate was concentrated *in vacuo*. Purification of the reaction mixture using MPLC afforded the product.

F. Synthesis of Styryl Azides.

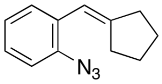

17b

Styryl Azide 17b. The general procedure for azidation was followed using 0.95 g of aniline **s39** (5.5 mmol), 0.05 g of $CuSO_4$, freshly prepared dichloromethane solution of TfN_3 (from 18.0 mmol of Tf_2O), and 2.5 mL of Et_3N (18.0 mmol) in 6 mL of CH_2Cl_2, 4 mL of MeOH and 2 mL of H_2O. Purification by MPLC (1:100 EtOAc: hexanes) afforded the product as a light yellow oil, as a 72:28 mixture of *Z*- and *E*-isomers (0.68 g, 62%): Selected spectral data for the major (*Z*) isomer: ^{1}H NMR (500 MHz, $CDCl_3$) δ 7.21 – 7.32 (m, 6H), 7.13 – 7.15 (d, *J* = 8.5 Hz, 1H), 6.80 – 6.84 (m, 2H), 6.62 (s, 1H), 2.33 (s, 3H); ^{13}C NMR (125 MHz, $CDCl_3$) δ 141.5 (C), 140.3 (C), 137.9 (C), 131.2 (CH), 129.8 (C), 128.4 (CH), 127.7 (CH), 127.1 (CH), 124.2 (CH), 121.9 (CH), 118.1 (CH), 26.5 (CH_3); Selected spectral data for the minor (*E*) isomer: ^{1}H NMR (500 MHz, $CDCl_3$) δ 7.64 (dd, *J* = 1.0 Hz, 8.5 Hz, 2H), 7.36 – 7.47 (m, 5H), 7.18 (dd, *J* = 2.0 Hz, 6.0 Hz, 2H), 6.92 (s, 1H), 2.72 (s, 3H); ^{13}C NMR (125 MHz, $CDCl_3$) δ 143.3 (C), 138.7 (C), 138.5 (C), 130.8 (CH), 130.2 (C), 128.5 (CH), 128.2 (CH), 127.5 (CH), 126.1 (CH), 124.4 (CH), 123.1 (CH), 118.5 (CH), 17.5 (CH_3); Selected spectral data for the mixture: δ IR (thin film): 2116, 2083, 1479, 1443, 1278, 759 cm^{-1}. HRMS (EI) *m* / *z* calcd for $C_{12}H_{13}N_3$ $(M)^+$ 199.1109, found 119.1112.

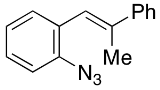

14e

Styryl Azide 14e.[9] The general procedure for azidation was followed using 0.65 g of aniline **s30** (3.1 mmol), 0.02 g of $CuSO_4$, freshly prepared dichloromethane solution of TfN_3 (from 9.0 mmol of Tf_2O), and 1.3 mL of Et_3N (9.0 mmol) in 4 mL of CH_2Cl_2, 2 mL of MeOH and 1 mL of H_2O. Purification by MPLC (1:100 – 20:80 EtOAc: hexanes) afforded the product as a light yellow oil, as a 72:28 mixture of *Z*- and *E*-isomers (0.59 g, 81%): Selected spectral data for the major (*Z*) isomer: ^{1}H NMR (500 MHz, $CDCl_3$) δ 7.21 – 7.32 (m, 6H), 7.13 – 7.15 (d, *J* = 8.5 Hz, 1H), 6.80 – 6.84 (m, 2H), 6.62 (s, 1H), 2.33 (s, 3H); ^{13}C NMR (125 MHz, $CDCl_3$) δ 141.5 (C), 140.3 (C), 137.9 (C), 131.2 (CH), 129.8 (C), 128.4 (CH), 127.7 (CH), 127.1 (CH), 124.2 (CH), 121.9 (CH), 118.1 (CH), 26.5 (CH_3) one overlapping aromatic C not distinguishable; Selected spectral data for the minor (*E*) isomer: ^{1}H NMR (500 MHz, $CDCl_3$) δ 7.64 (dd, *J* = 1.0 Hz, 8.5 Hz, 2H), 7.36 – 7.47 (m, 5H), 7.18 (dd, *J* = 2.0

Hz, 6.0 Hz, 2H), 6.92 (s, 1H), 2.72 (s, 3H); ^{13}C NMR (125 MHz, $CDCl_3$) δ 143.3 (C), 138.7 (C), 138.5 (C), 130.8 (CH), 130.2 (C), 128.5 (CH), 128.2 (CH), 127.5 (CH), 126.1 (CH), 124.4 (CH), 123.1 (CH), 118.5 (CH), 17.5 (CH_3); Selected spectral data for the mixture: δ IR (thin film): 2116, 2083, 1479, 1443, 1278, 759 cm^{-1}. HRMS (EI) *m / z* calcd for $C_{15}H_{13}N_3$ $(M)^+$ 235.11095, found 235.11230.

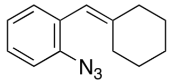

17c

Styryl Azide 17c. The general procedure for azidation was followed using 1.42 g of aniline **s39** (7.6 mmol), 0.06 g of $CuSO_4$, freshly prepared dichloromethane solution of TfN_3 (from 22.8 mmol of Tf_2O), and 3.2 mL of Et_3N (22.8 mmol) in 10 mL of CH_2Cl_2, 5 mL of MeOH and 3 mL of H_2O. Purification by MPLC (100% hexane) afforded the product as a light yellow oil (1.36 g, 84%): ^{1}H NMR (500 MHz, $CDCl_3$) δ 7.23 – 7.27 (m, 1H), 7.17 (d, *J* = 7.5 Hz, 1H), 7.13 (dd, *J* = 1.0 Hz, 8.0 Hz, 1H), 7.07 – 7.10 (m, 1H), 6.14 (s, 1H), 2.30 (t, *J* = 5.5 Hz, 2H), 2.25 (t, *J* = 5.5 Hz, 2H), 1.55 – 1.68 (m, 6H); ^{13}C NMR (125 MHz, $CDCl_3$) δ 145.0 (C), 138.0 (C), 131.0 (CH), 130.2 (C), 127.5 (CH), 124.3 (CH), 118.3 (CH), 117.3 (CH), 37.5 (CH_2), 29.8 (CH_2), 28.6 (CH_2), 27.9 (CH_2), 26.7 (CH_2); IR (thin film): 2112, 2080, 1482, 1444, 1281, 836, 746, 836, 746, 725, 671 cm^{-1}. HRMS (EI) *m / z* calcd for $C_{13}H_{15}N_3$ $(M)^+$ 213.12660, found 213.12686.

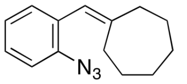

17d

Styryl Azide 17d. The general procedure for azidation was followed using 0.21 g of aniline **s39** (1.1 mmol), 0.01 g of $CuSO_4$, freshly prepared dichloromethane solution of TfN_3 (from 3.0 mmol of Tf_2O), and 0.4 mL of Et_3N (3.0 mmol) in 4 mL of CH_2Cl_2, 2 mL of MeOH and 1 mL of H_2O. Purification by MPLC (100% hexane) afforded the product as a light yellow oil (0.18 g, 76%): ^{1}H NMR (500 MHz, $CDCl_3$) δ 7.19 – 7.26 (m, 2H), 7.08 – 7.14 (m, 2H), 6.25 (s, 1H), 2.45 (t, *J* = 6.0 Hz, 2H), 2.37 (t, *J* = 6.0 Hz, 2H), 1.58 – 1.70 (m, 8H); ^{13}C NMR (125 MHz, $CDCl_3$) δ 146.8 (C), 137.8 (C), 130.6 (CH), 127.4 (CH), 124.3 (CH), 120.6 (CH), 118.3 (CH), 38.3 (CH_2), 31.3 (CH_2), 29.8 (CH_2), 29.3 (CH_2), 29.1 (CH_2), 27.3 (CH_2) one overlapping aromatic C not distinguishable; IR (thin film): 2919, 2113, 2079, 1572, 1480, 1443, 1281, 1085, 746 cm^{-1}. HRMS (EI) *m / z* calcd for $C_{14}H_{17}N_3$ $(M)^+$ 227.14225 found 227.14169.

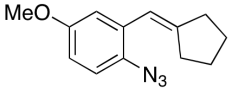

17e

Styryl Azide 17e. The general procedure for azidation was followed using 0.97 g of aniline **s39** (4.8 mmol), 0.02 g of $CuSO_4$, freshly prepared dichloromethane solution of TfN_3 (from 14.4 mmol of Tf_2O), and 2.0 mL of Et_3N (14.4 mmol) in 6 mL CH_2Cl_2, 3 mL of MeOH and 2 mL of H_2O. Purification by MPLC (100% hexane) afforded the product as a light yellow oil (0.80 g, 73%): ^{1}H NMR (500 MHz, $CDCl_3$) δ 7.04 (d, *J* = 8.7 Hz, 1H), 6.93 (d, *J* = 2.8 Hz, 1H), 6.77 (dd, *J* = 8.7, 2.9 Hz, 1H), 6.43 (s, 1H), 3.80 (s, 3H), 2.46 – 2.52 (m, 4H), 1.71 – 1.78 (m, 2H), 1.71 – 1.66 (m, 2H); ^{13}C NMR (125 MHz, $CDCl_3$) δ 156.4 (C), 149.3 (C), 131.6 (C), 129.8 (C), 119.1 (CH), 115.3 (CH), 114.6 (CH), 112.5 (CH), 55.5 (CH_3), 35.5 (CH_2), 31.2 (CH_2), 27.0 (CH_2), 25.6 (CH_2); IR (thin film): 2953, 2115, 2080, 1480, 1446, 744 cm^{-1}. HRMS (EI) *m / z* calcd for $C_{13}H_{15}ON_3$ $(M)^+$ 229.12152, found 229.12192.

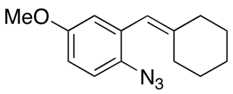

17f

Styryl Azide 17f. The general procedure for azidation was followed using 0.21 g of aniline **s39** (1.0 mmol), 0.01 g of $CuSO_4$, freshly prepared dichloromethane solution of TfN_3 (from 3.0 mmol of Tf_2O), and 1.3 mL of Et_3N (3.0 mmol) in 3 mL of CH_2Cl_2, 2 mL of MeOH and 1 mL of H_2O. Purification by MPLC (100% hexane) afforded the product as a light yellow oil (0.41 g, 43%): ^{1}H NMR (500 MHz, $CDCl_3$) δ 7.04 (d, J = 9.0 Hz, 1H), 6.80 (dd, J = 3.0 Hz, 8.5 Hz, 1H), 6.72 (d, J = 3.0Hz, 1H), 6.12 (s, 1H), 3.79 (s, 3H), 2.26 – 2.31 (m, 4H), 1.56 – 1.67 (m, 6H); ^{13}C NMR (125 MHz, $CDCl_3$) δ 156.3 (C), 145.3 (C), 131.3 (C), 130.6 (C), 119.3 (CH), 117.4 (CH), 116.4 (CH), 112.8 (CH), 55.5 (CH_3), 37.4 (CH_2), 29.9 (CH_2), 28.6 (CH_2), 27.8 (CH_2), 26.6 (CH_2); IR (thin film): 2106, 2084, 1486, 1284, 1239, 1165, 1035, 798, 732 cm^{-1}. HRMS (EI) m / z calcd for $C_{14}H_{17}ON_3$ $(M)^+$ 243.1372, found 243.1372.

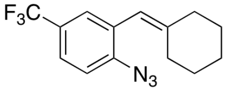

17g

Styryl Azide 17g. The general procedure for azidation was followed using 1.60 g of aniline **s39** (6.3 mmol), 0.04 g of $CuSO_4$, freshly prepared dichloromethane solution of TfN_3 (from 18.8 mmol of Tf_2O), and 2.6 mL of Et_3N (18.8 mmol) in 8 mL of CH_2Cl_2, 4 mL of MeOH and 3 mL of H_2O. Purification by MPLC (100% hexane) afforded the product as a light yellow oil (0.04 g, 3%): ^{1}H NMR (500 MHz, $CDCl_3$) δ 7.49 (dd, J = 8.5 Hz, 2.0 Hz, 1H), 7.40 (s, 1H), 7.20 (d, J = 7.0 Hz, 1H), 6.11 (s, 1H), 2.30 (t, J = 5.5 Hz, 2H), 2.22 (t, J = 5.5 Hz, 2H), 1.54 – 1.70 (m, 6H); ^{13}C NMR (125 MHz, $CDCl_3$) δ 146.7 (C), 141.5 (C), 130.6 (C), 128.2 (q, J_{CF} = 3.6 Hz, CH), 126.4 (q, J_{CF} = 31.4 Hz, C), 124.4 (q, J_{CF} = 3.4 Hz, CH), 124.0 (q, J_{CF} = 270.1 Hz, C), 118.5 (CH), 116.3 (CH), 37.4 (CH_2), 29.8 (CH_2), 28.5 (CH_2), 27.8 (CH_2), 26.5 (CH_2); ^{19}F NMR ($CDCl_3$, 282 MHz) δ –61.62; IR (thin film): 2929, 2120, 2088, 1329, 1289, 1106, 817 cm^{-1}. HRMS (EI) m / z calcd for $C_{14}H_{14}NF_3$ $(M - N_2)^+$ 255.1000, found 255.1000.

IV. Development of Rhodium-Catalyzed Migration Reaction.

A. General Procedure for the Screening of Catalysts to Promote the Migration.

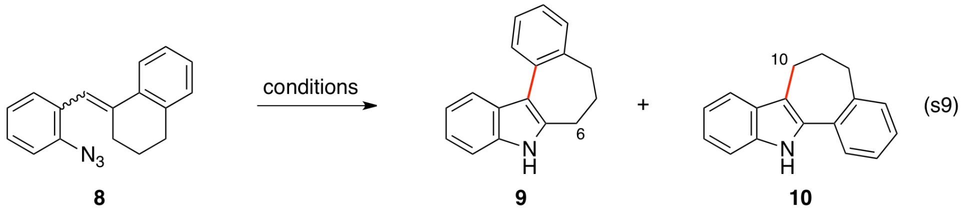

To a mixture of 0.026 g of aryl azide **8** (0.1 mmol), 0-100 % w/w of crushed 4 Å mol sieves, and metal salt (0 – 5 mol %) in Schlenk tube was added 0.50 mL of solvent. The resulting mixture was heated and, after 16 h, the heterogenous mixture was filtered through a short pad of Al_2O_3. The filtrate was concentrated *in vacuo*. The resulting solid (oil) was dissolved in 1.5 mL of $CDCl_3$ and 0.007 mL of dibromomethane (0.1 mmol) was added. The areas of the C–H peak on the carbon 6 position in **9** and carbon 10 in **10** were compared to the area of CH_2Br_2 to derive a yield.

Table s1. Optimization of Migration Process[a,b]

Entry	metal salt	Mol %	solvent	wt %, 4 Å MS	T (°C)	yield, %[c]	**6** : **7**
1	none	n.a.	xylenes	0	170		xx:xx
2	$Rh_2(O_2CCH_3)_4$	5	PhMe	100	70	8	n.a.
3	$Rh_2(O_2CCF_3)_4$	5	PhMe	100	70	86	100:0
4	$Rh_2(O_2CC_3F_7)_4$	5	PhMe	100	70	95	100:0
5	$Rh_2(O_2CC_7H_{15})_4$	5	PhMe	100	70	93	96:4
6	$Rh_2(O_2CC_7H_{15})_4$	3	PhMe	100	70	80[d]	96:4
7	$Rh_2(O_2CC_7H_{15})_4$	3	PhMe	100	60	88[e]	96:4
8	$Rh_2(O_2CC_7H_{15})_4$	5	PhMe	100	80	95	96:4
9	$Rh_2(O_2CC_7H_{15})_4$	5	DCE	100	60	74[f]	96:4
10	$Rh_2(O_2CC_7H_{15})_4$	5	DCE	100	70	94	98:2
11	$Rh_2(O_2CC_7H_{15})_4$	5	DCE	100	80	88	96:4
12	$Rh_2(esp)_2$	5	PhMe	100	70	98	>98:2
13	$RhCl_3$	5	PhMe	100	70	0	n.a.
14	$(Ph_3P)_3RuCl$	5	PhMe	100	70	trace	n.a.
15	$Cu(OTf)_2$	5	PhMe	100	70	0	n.a.
16	Ru(TPFPP)CO	5	IPAC	0		48	100:0

[a] Reaction performed in conical vial. [b] 16 hour reaction time. [c] Isolated yield after Al_2O_3 chromatography.

B. Optimized General Procedure.

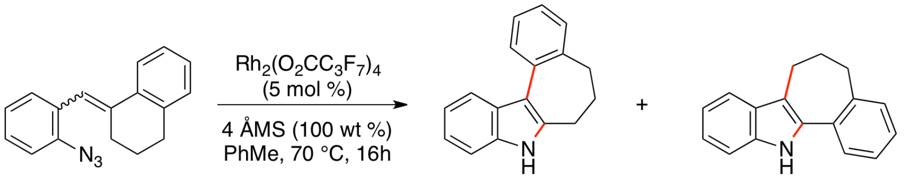

(s10)

8 **9** **10**

To a mixture of 0.026 g of aryl azide **5** (0.1 mmol), 100 % w/w of crushed 4 Å mol sieves, and metal salt ($Rh_2(esp)_4$ or $Rh_2(hpfb)_4$, 5 mol %) in Schlenk tube was added 0.50 mL of PhMe. The resulting mixture was heated at 70 °C for 16 h, then, the heterogenous mixture was filtered through a short pad of Al_2O_3. The filtrate was concentrated *in vacuo* to afford analytically pure indole. In some cases, purification by MPLC is required.

C. Effect of Aryl Azide Substitution on Rhodium-Catalyzed Migration Selectivity.

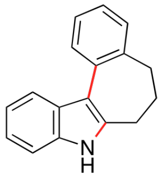

9

Indole 9. The general procedure was followed using 0.027 g of styryl azide **8** (0.102 mmol) and 0.005 g of $Rh_2(hpfb)_4$ (0.005 mmol) in 0.5 mL of PhMe. Filtration by Al_2O_3 afforded analytically pure compound **9** (0.025, 95%). mp 125 – 127 °C; ^1H NMR (500 MHz, $CDCl_3$) δ 7.94 – 7.96 (m, 2H), 7.87 (d, J = 7.5 Hz, 1H), 7.34 –

7.40 (m, 2H), 7.30 (d, J = 7.5 Hz, 1H), 7.18 – 7.24 (m, 3H), 2.94 (t, J = 7.5 Hz, 2H), 2.76 – 2.78 (m, 2H), 2.23 – 2.29 (m, 2H); ^{13}C NMR (125 MHz, $CDCl_3$) δ 141.0 (C), 136.9 (C), 135.7 (C), 135.1 (C), 129.3 (CH), 127.9 (CH), 127.3 (C), 126.3 (CH), 125.3 (CH), 121.6 (CH), 120.1 (CH), 119.2 (CH), 112.8 (C), 110.6 (CH), 33.5 (CH_2), 30.1 (CH_2), 26.5 (CH_2); IR (thin film): 3396, 1599, 1492, 1459, 1275, 906, 745 cm^{-1}. HRMS (EI) m / z calcd for $C_{17}H_{15}N$ $(M)^+$ 233.12054, found 233.11957.

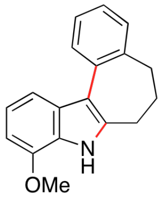

12a

Indoles 12a. The general procedure was followed using 0.0291 g of styryl azide **11a** (0.100 mmol) and 0.0053 g of $Rh_2(hpfb)_4$ (0.005 mmol) in 0.5 mL of PhMe. Filtration by Al_2O_3 afforded analytically pure compound **12a** (0.0252 g, 96%) as a colorless oil. ^{1}H NMR (500 MHz, $CDCl_3$) δ 8.31 (br, 1H), 7.88 (dd, J = 1.0 Hz, 7.5 Hz, 1H), 7.57 (d, J = 8.0 Hz, 1H), 7.38 (dt, J = 1.5 Hz, 7.5 Hz, 1H), 7.30 (dd, J = 1.0 Hz, 7.5 Hz, 1H), 7.21 (dt, J = 1.5 Hz, 7.5 Hz, 1H), 7.13 (t, J = 8.0 Hz, 1H), 6.71 (d, J = 8.0 Hz, 1H), 4.02 (s, 3H), 2.97 (t, J = 7.5 Hz, 2H), 2.79 – 2.76 (m, 2H), 2.30 – 2.25 (m, 2H); ^{13}C NMR (125 MHz, $CDCl_3$) δ 164.0 (C), 145.8 (C), 141.0 (C), 136.5 (C), 135.4 (C), 129.3 (CH), 128.6 (C), 128.0 (CH), 126.3 (CH), 125.2 (CH), 120.5 (CH), 113.3 (C), 112.2 (CH), 101.8 (CH), 55.4 (CH_3), 33.6 (CH_2), 30.2 (CH_2), 26.4 (CH_2); IR (thin film): 3443, 2941, 1497, 1264, 733 cm^{-1}. HRMS (EI) m / z calcd for $C_{18}H_{17}ON$ $(M)^+$ 263.13102, found 263.13028.

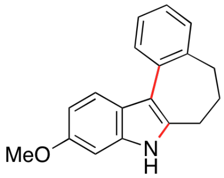

12b

Indoles 12b. The general procedure was followed using 0.0291 g of styryl azide **11b** (0.100 mmol) and 0.0053 g of $Rh_2(hpfb)_4$ (0.005 mmol) in 0.5 mL of PhMe. Filtration by Al_2O_3 afforded analytically pure compound **12b** (0.0254 g, 97%) as a white solid; mp 164 – 166 °C. ^{1}H NMR (500 MHz, $CDCl_3$) δ 7.85 – 7.83 (m, 2H), 7.81 (d, J = 7.5 Hz, 1H), 7.35 (dt, J = 1.5 Hz, 7.5 Hz, 1H), 7.27 (dd, J = 1.0 Hz, 7.5 Hz, 1H), 7.21 (dt, J = 1.0 Hz, 7.5 Hz, 1H), 6.85 (dd, J = 2.5 Hz, 8.5 Hz, 1H), 6.82 (d, J = 1.5 Hz, 1H), 3.85 (s, 3H), 2.91 (t, J = 7.5 Hz, 2H), 2.77 – 2.75 (m, 2H), 2.23 – 2.17 (m, 2H); ^{13}C NMR (125 MHz, $CDCl_3$) δ 156.0 (C), 141.1 (C), 136.6 (C), 135.6 (C), 135.0 (C), 129.3 (CH), 127.8 (CH), 126.3 (CH), 125.2 (CH), 121.7 (C), 120.0 (CH), 112.5 (C), 109.3 (CH), 94.7 (CH), 55.8 (CH_3), 33.7 (CH_2), 29.5 (CH_2), 26.8 (CH_2); IR (thin film): 3399, 2937, 1461, 1264, 738 cm^{-1}. HRMS (EI) m / z calcd for $C_{18}H_{17}ON$ $(M)^+$ 263.13102, found 263.13140.

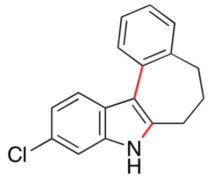

12c

Indoles 12c. The general procedure was followed using 0.0295 g of styryl azide **11c** (0.100 mmol) and 0.0053 g of $Rh_2(hpfb)_4$ (0.005 mmol) in 0.5 mL of PhMe. Filtration by Al_2O_3 afforded analytically pure compound **12c** (0.0264 g, 99%) as a colorless oil. ^{1}H NMR (500 MHz, $CDCl_3$) δ 7.92 (br, 1H), 7.82 (d, J = 8.5 Hz, 1H), 7.79 (dd, J = 1.5 Hz, 7.5 Hz, 1H), 7.37 (dt, J = 1.5 Hz, 7.5 Hz, 1H), 7.32 – 7.28 (m, 2H), 7.21 (dt, J = 1.5 Hz, 6.5 Hz, 1H), 7.14 (dd, J = 2.0 Hz, 6.5 Hz, 1H), 2.91 (t, J = 7.5 Hz, 2H), 2.78 – 2.74 (m, 2H), 2.26 – 2.21 (m, 2H); ^{13}C NMR (125 MHz, $CDCl_3$) δ 141.1 (C), 137.5 (C), 136.1 (C), 134.5 (C), 129.4 (CH), 127.8 (CH), 127.3 (C), 126.4 (CH), 126.0 (C), 125.6 (CH), 120.7 (CH), 120.1 (CH), 112.9 (C), 110.5 (CH), 33.5 (CH_2), 29.8 (CH_2), 26.6 (CH_2); IR (thin film): 3401, 2929, 1462, 1275, 749 cm^{-1}. HRMS (EI) m / z calcd for $C_{17}H_{14}NCl$ $(M)^+$ 267.08147, found 267.08182.

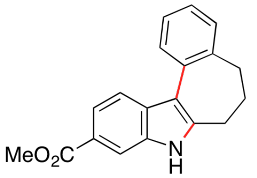

12d

Indoles 12d. The general procedure was followed using 0.0319 g of styryl azide **11d** (0.100 mmol) and 0.0053 g of $Rh_2(esp)_2$ (0.005 mmol) in 0.5 mL of PhMe. Filtration by Al_2O_3 afforded analytically pure compound **12d** (0.0274 g, 94%) as a white solid; mp 203 – 205 °C. ^{1}H NMR (500 MHz, $CDCl_3$) δ 8.55 (br, 1H), 8.11 (d, J = 1.5 Hz, 1H), 7.91 (d, J = 8.5 Hz, 1H), 7.85 (dd, J = 1.5 Hz, 8.5 Hz, 1H), 7.79 (dd, J = 1.0 Hz, 8.0 Hz, 1H), 7.37 (dt, J = 1.5 Hz, 7.5 Hz, 1H), 7.29 (d, J = 7.5 Hz, 1H), 7.20 (dt, J = 1.5 Hz, 7.5 Hz, 1H), 3.96 (s, 3H), 2.97 (t, J = 7.5 Hz, 2H), 2.75 – 2.72 (m, 2H), 2.30 – 2.25 (m, 2H); ^{13}C NMR (125 MHz, $CDCl_3$) δ 168.4 (C), 140.9 (C), 140.8 (C), 135.0 (C), 134.5 (C), 130.9 (C), 129.4 (CH), 127.9 (CH), 126.4 (CH), 125.7 (CH), 122.9 (C), 121.3 (CH), 118.6 (CH), 113.5 (C), 112.9 (CH), 52.0 (CH_3), 33.3 (CH_2), 30.2 (CH_2), 26.4 (CH_2); IR (thin film): 3656, 2950, 1690, 1503, 1217, 1062, 911 cm^{-1}. HRMS (EI) m / z calcd for $C_{19}H_{17}O_2N$ $(M)^+$ 291.12593, found 291.12553.

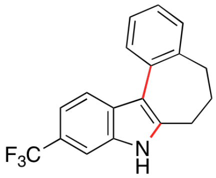

12e

Indole 12e. The general procedure was followed using 0.046 g of styryl azide **11e** (0.140 mmol) and 0.007 g of $Rh_2(hpfb)_4$ (0.007 mmol) in 0.6 mL of PhMe. Filtration by Al_2O_3 afforded analytically pure compound **12e** (0.038 g, 90%). ^{1}H NMR (500 MHz, $CDCl_3$): 8.18 (s, 1H), 7.98 (d, J = 8.5 Hz, 1H), 7.80 (d, J = 7.5 Hz, 1H), 7.06 (s, 1H), 7.38 – 7.42 (m, 2H), 7.31 -7.32 (m, 1H), 7.22 – 7.26 (m, 1H), 2.95 (t, 7.5 Hz, 2H), 2.73 – 2.76 (m, 2H), 2.24 – 2.30 (m, 2H); ^{13}C NMR (125 MHz, $CDCl_3$): 141.0 (C), 139.7 (C), 134.6 (C), 134.3 (C), 129.6 (C), 129.5 (CH), 127.9 (CH), 126.5 (CH), 125.8 (CH), 125.3 (q, J = 269.9 Hz, C), 123.5 (q, J = 31.8 Hz, C), 119.4 (CH), 116.8 (q, J = 3.5 Hz, CH), 113.2 (C), 108.1 (q, J = 4.8 Hz, CH), 33.3 (CH_2), 30.1 (CH_2), 26.4 (CH_2); ^{19}F

NMR ($CDCl_3$, 282 MHz) ; IR (thin film): 3401, 2940, 1740, 1728, 1333, 1217, 1111, 1057 cm^{-1}. HRMS (EI) *m* / *z* calcd for $C_{18}H_{14}NF_3$ $(M)^+$ 301.10783, found 301.10748.

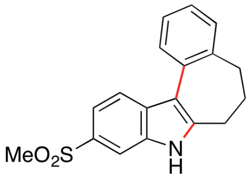

12f

Indoles 12f. The general procedure was followed using 0.0339 g of styryl azide **11f** (0.100 mmol) and 0.0053 g of $Rh_2(hpfb)_4$ (0.005 mmol) in 0.5 mL of PhMe. Filtration by Al_2O_3 afforded analytically pure compound **12f** (0.0295 g, 95%) as a white solid; mp 159 – 161 °C. ^{1}H NMR (500 MHz, $CDCl_3$) δ 9.23 (br, 1H), 8.11 (d, *J* = 1.0 Hz, 1H), 7.97 (d, *J* = 8.5 Hz, 1H), 7.71 (d, *J* = 8.0 Hz, 1H), 7.65 (dd, *J* = 1.0 Hz, 8.5 Hz, 1H), 7.36 (dt, *J* = 1.0 Hz, 7.5 Hz, 1H), 7.29 (d, *J* = 7.5 Hz, 1H), 7.21 (dt, *J* = 1.0 Hz, 7.5 Hz, 1H), 3.13 (s, 3H), 2.99 (t, *J* = 7.5 Hz, 2H), 2.71(t, *J* = 6.0 Hz, 2H), 2.29 – 2.24 (m, 2H); ^{13}C NMR (125 MHz, $CDCl_3$) δ 142.4 (C), 140.9 (C), 134.5 (C), 134.1 (C), 132.0 (C), 131.0 (C), 129.1 (CH), 127.8 (CH), 126.5 (CH), 126.0 (CH), 119.6 (CH), 118.2 (CH), 113.4 (C), 111.0 (CH), 45.3 (CH_3), 33.2 (CH_2), 30.2 (CH_2), 26.2 (CH_2); IR (thin film): 3351, 2928, 1498, 1265, 1141, 740 cm^{-1}. HRMS (EI) *m* / *z* calcd for $C_{18}H_{17}O_2NS$ $(M)^+$ 311.09800, found 311.09821.

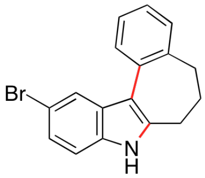

12g

Indoles 12g. The general procedure was followed using 0.0340 g of styryl azide **11g** (0.100 mmol) and 0.0053 g of $Rh_2(hpfb)_4$ (0.005 mmol) in 0.5 mL of PhMe. Filtration by Al_2O_3 afforded analytically pure compound **12g** (0.0295 g, 95%) as a white solid; mp 146 – 148 °C. ^{1}H NMR (500 MHz, $CDCl_3$) δ 8.03 (d, *J* = 1.5 Hz, 1H), 8.00 (br, 1H), 7.76 (d, *J* = 7.5 Hz, 1H), 7.37 (dt, *J* = 1.5 Hz, 7.5 Hz, 1H), 7.29 – 7.26 (m, 2H), 7.21 (dt, *J* = 1.0 Hz, 6.5 Hz, 1H), 7.19 (d, *J* = 8.5 Hz, 1H), 2.92 (t, *J* = 7.5 Hz, 2H), 2.74 – 2.72 (m, 2H), 2.27 – 2.22 (m, 2H); ^{13}C NMR (125 MHz, $CDCl_3$) δ 140.9 (C), 138.2 (C), 134.4 (C), 134.3 (C), 129.4 (CH), 129.0 (C), 127.8 (CH), 126.5 (CH), 125.7 (CH), 124.3 (CH), 121.6 (CH), 113.5 (C), 112.6 (C), 111.9 (CH), 33.4 (CH_2), 30.1 (CH_2), 26.4 (CH_2); IR (thin film): 3411, 2936, 1469, 1264, 740 cm^{-1}. HRMS (EI) *m* / *z* calcd for $C_{17}H_{14}NBr$ $(M)^+$ 313.02891, found 313.03030.

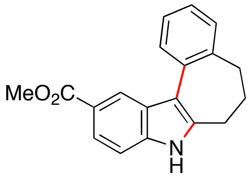

12h

Indole 12h. The general procedure was followed using 0.0319 g of styryl azide **11h** (0.100 mmol) and 0.0053 g of $Rh_2(esp)_2$ (0.005 mmol) in 0.5 mL of PhMe. Filtration by Al_2O_3 afforded analytically pure compound **12h** (0.0276 g, 95%) as a white solid; mp 244 – 246 °C. ^{1}H NMR (500 MHz, acetone-d_6) δ 10.65 (br, 1H), 8.57 (s, 1H), 7.82 (dd, *J* = 1.0 Hz, 8.5 Hz, 1H), 7.79 (d, *J* = 7.5 Hz, 1H), 7.46 (d, *J* = 8.5 Hz, 1H), 7.37 (dt, *J* = 1.5 Hz,

7.5 Hz, 1H), 7.28 (d, J = 7.5 Hz, 1H), 7.17 (dt, J = 1.5 Hz, 7.5 Hz, 1H), 3.87 (s, 3H), 3.04 (t, J = 7.5 Hz, 2H), 2.74 – 2.72 (m, 2H), 2.24 – 2.19 (m, 2H); ^{13}C NMR (125 MHz, acetone-d_6) δ 167.5 (C), 141.2 (C), 139.3 (C), 139.0 (C), 134.6 (C), 129.3 (CH), 127.7 (CH), 126.8 (C), 126.3 (CH), 125.4 (CH), 122.4 (CH), 121.6 (C), 121.2 (CH), 112.9 (C), 110.6 (CH), 51.0 (CH_3), 33.3 (CH_2), 29.6 (CH_2), 26.1 (CH_2); IR (thin film): 3643, 2951, 1737, 1231, 1062, 913, 857 cm^{-1}. HRMS (EI) m / z calcd for $C_{19}H_{17}O_2N$ (M)$^+$ 291.12593, found 291.12504.

D. Effect of Migrating Group Identity on Rhodium-Catalyzed Migration Selectivity.

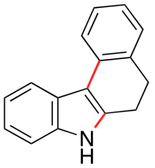

15a

Indole 15a.[10] The general procedure was followed using 0.041 g of styryl azide **14a** (0.17 mmol) and 0.006 g of $Rh_2(esp)_4$ (0.008 mmol) in 0.7 mL of PhMe. Filtration by Al_2O_3 afforded a 98:2 mixture of compound 12a and 12b; Purification by MPLC (10:100 EtOAc: hexanes) afforded compound **15a** as a white powder (0.032 g, 88%); Indole **15a** was previously reported by Katritzky and Wang:[10] ^{1}H NMR (500 MHz, $CDCl_3$) δ 8.05 (d, J = 7.5 Hz, 1H), 7.95 (s, 1H), 7.88 (d, J = 7.5 Hz, 1H), 7.31 – 7.36 (m, 2H), 7.19 – 7.27 (m, 3H), 7.09 – 7.12 (m, 1H), 3.05 – 3.08 (t, J = 7.5 Hz, 2H), 2.93 – 2.96 (t, J = 7.5 Hz, 2H); ^{13}C NMR (125 MHz, $CDCl_3$): δ 137.2 (C), 136.2 (C), 133.9 (C), 133.3 (C), 128.0 (CH), 127.0 (CH), 125.0 (C), 124.3 (CH), 122.3 (CH), 121.5 (CH), 120.6 (CH), 119.5 (CH), 111.1 (CH), 110.8 (C), 29.5 (CH_2), 22.5 (CH_2); IR (thin film): 3396, 1601, 1497, 1456, 1255, 908, 767, 731 cm^{-1}.

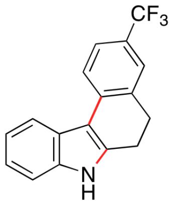

15b

Indole 15b. The general procedure was followed using 0.050 g of styryl azide **14b** (0.16 mmol) and 0.006 g of $Rh_2(esp)_4$ (0.008 mmol) in 0.6 mL of PhMe. Filtration by Al_2O_3 afforded a 95:5 mixture of compound **15b** and **16b**; Purification by MPLC (10:100 EtOAc: hexanes) afforded compound **15b** as a light yellow powder (0.031 g, 78%); mp 227 -230 °C; ^{1}H NMR (500 MHz, DMSO) δ 8.03 (d, J = 7.5 Hz, 1H), 7.93 – 7.95 (m, 1H), 7.37 – 7.45 (m, 3H), 7.11 – 7.13 (m, 2H), 3.10 (t, J = 7.5 Hz, 2H), 2.98 (t, J = 8.0 Hz, 2H), *N-H peak not available*; ^{13}C NMR (125 MHz, DMSO) δ 139.2 (C), 136.9 (C), 136.4 (C), 131.6 (C), 127.53 (CH), 127.49 (q, J_{CF} = 27.8 Hz, C), 126.0 (CH), 125.3 (q, J_{CF} = 272.0 Hz, C), 124.6, 121.5 (CH), 120.9 (q, J_{CF} = 5.5 Hz, CH), 120.6 (CH), 119.1 (CH), 112.2 (CH), 108.3 (C), 25.5 (CH_2), 21.5 (CH_2); IR (thin film): 3406, 1601, 1557, 1452, 1311, 1162, 1108, 749 cm^{-1}. HRMS (EI) m / z calcd for $C_{17}H_{12}NF_3$ (M)$^+$ 287.09218, found 287.09182.

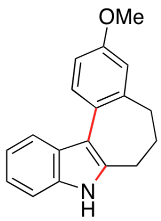

15c

Indole 15c. The general procedure was followed using 0.041 g of styryl azide **14c** (0.141 mmol) and 0.005 g of $Rh_2(esp)_4$ (0.007 mmol) in 0.5 mL of PhMe. Filtration by Al_2O_3 afforded analytically pure compound **15c** (0.034 g, 92%). ^{1}H NMR (500 MHz, $CDCl_3$) δ 7.99 (s, 1H), 7.56 (d, *J* = 7.5 Hz, 1H), 7.48 (d, *J* = 8.0 Hz, 1H), 7.36 (d, *J* = 8.0 Hz, 1H), 7.23 (t, *J* = 7.5 Hz, 1H), 7.15 (t, *J* = 7.5 Hz, 1H), 6.82 – 6.85 (m, 2H), 3.86 (s, 3H), 3.11 (t, *J* = 7.0 Hz, 2H), 2.89 – 2.91 (m, 2H), 2.15 – 2.19 (m, 2H); ^{13}C NMR (125 MHz, $CDCl_3$) δ 158.7 (C), 144.0 (C), 136.1 (C), 132.7 (C), 130.2 (C), 126.4 (CH), 124.6 (C), 122.4 (CH), 119.5 (CH), 118.4 (CH), 115.5 (CH), 113.1 (C), 111.5 (CH), 110.5 (CH), 55.3 (CH_3), 35.6 (CH_2), 26.7 (CH_2), 26.0 (CH_2); IR (thin film): 3414, 2926, 2834, 1605, 1461, 1433, 1260, 1243, 1033, 810, 735 cm^{-1}. HRMS (EI) *m* / *z* calcd for $C_{18}H_{17}ON$ $(M)^+$ 263.13102, found 263.13192.

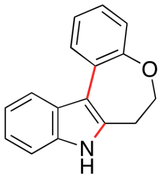

15d

Indole 15d. The general procedure was followed using 0.052 g of styryl azide **14d** (0.198 mmol) and 0.008 g of $Rh_2(esp)_4$ (0.010 mmol) in 0.7 mL of PhMe. Filtration by Al_2O_3 afforded analytically pure compound **15d** (0.044 g, 94%). mp 175 -177 °C; ^{1}H NMR (500 MHz, $CDCl_3$) δ 8.14 (d, *J* = 7.5 Hz, 1H), 8.10 (d, *J* = 7.5 Hz, 1H), 8.04 (s, 1H), 7.31 – 7.32 (m, 1H), 7.12 – 7.26 (m, 5H), 4.39 (t, *J* = 6.0 Hz, 2H), 3.32 (t, *J* = 6.0 Hz, 2H); ^{13}C NMR (125 MHz, $CDCl_3$) δ 158.7 (C), 136.5 (C), 134.8 (C), 128.4 (CH), 127.3 (C), 127.0 (C), 125.9 (CH), 123.8 (CH), 122.1 (CH), 121.1 (CH), 120.3 (CH), 120.2 (CH), 110.6 (CH), 109.7 (C), 70.0 (CH_2), 31.0 (CH_2); IR (thin film): 3348, 1492, 1459, 1441, 1196, 905, 748 cm^{-1}. HRMS (EI) *m* / *z* calcd for $C_{16}H_{13}ON$ $(M)^+$ 235.09972, found 235.10055.

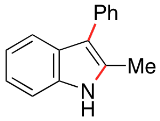

15e

Indole 15e.[11] The general procedure was followed using 0.044 g of styryl azide **14e** (0.187 mmol) and 0.007 g of $Rh_2(esp)_4$ (0.009 mmol) in 0.6 mL of PhMe (reaction is set up at 80 °C). Filtration by Al_2O_3 afforded analytically pure compound **15e** (0.035 g, 91%). Spectral data for **15e** matched that reported by Tan and Hartwig:[11] ^{1}H NMR (500 MHz, $CDCl_3$) δ 7.93 (s, 1H), 7.69 (d, *J* = 8.0 Hz, 1H), 7.53 – 7.55 (m, 2H), 7.47 – 7.50 (m, 2H), 7.31 – 7.35 (m, 2H), 7.17 – 7.20 (m, 1H), 7.12 – 7.15 (m, 1H), 2.52 (s, 3H); ^{13}C NMR (125 MHz, $CDCl_3$) δ 135.5 (C), 135.3 (C), 131.4 (C), 129.5 (CH), 128.5 (CH), 127.9 (C), 125.8 (CH), 121.6 (CH), 120.0 (CH), 118.8 (CH), 114.6 (C), 110.3 (CH), 12.6 (CH_3); IR (thin film): 3401, 1603, 1496, 1459, 1258, 908, 748, 702 cm^{-1}.

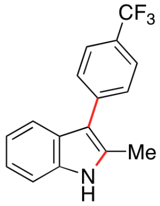

15f

Indole 15f.[12] The general procedure was followed using 0.036 g of styryl azide **14f** (0.117 mmol) and 0.005 g of $Rh_2(esp)_4$ (0.006 mmol) in 0.5 mL of PhMe (reaction is set up at 80 °C). Filtration by Al_2O_3 afforded analytically pure compound **15f** (0.030 g, 93%). Indole **15f** was previously reported by Chiba and co-workers.[12] Spectral data for **15f**: ^{1}H NMR (500 MHz, $CDCl_3$) δ 8.02 (s, 1H), 7.73 (d, J = 8.5 Hz, 1H), 7.66 (d, J = 7.5 Hz, 1H), 7.63 (d, J = 8.0 Hz, 1H), 7.36 (d, J = 8.0 Hz, 1H), 7.22 (dt, J = 1.0 Hz, 8.0 Hz, 1H), 7.16 (dt, J = 1.0 Hz, 7.5 Hz, 1H), 2.53 (s, 3H); ^{13}C NMR (125 MHz, $CDCl_3$) δ 139.4 (C), 135.3 (C), 132.2 (C), 129.4 (CH), 127.7 (q, J_{CF} = 32.8 Hz, C), 127.5 (C), 125.5 (q, J_{CF} = 3.5 Hz, CH), 124.5 (q, J_{CF} = 269.9 Hz, C), 122.0 (CH), 120.4 (CH), 118.5 (CH), 113.4 (C), 110.5 (CH), 12.6 (CH_3); IR (thin film): 3397, 1615, 1459, 1234, 1163, 1109, 1068, 747 cm^{-1}.

E. Effect of Ring Size on Rhodium-Catalyzed Migration.

1. General Procedure for the Optimization of Conditions to Promote the Migration.

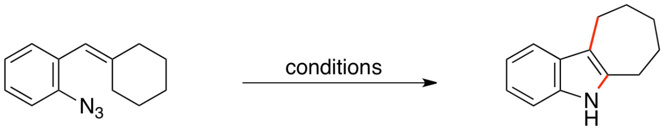

17c **18c** (s11)

To a mixture of 0.1 mmol of aryl azide **17c** (0.1 mmol), 0-100 % w/w of crushed 4 Å mol sieves, and metal salt (0 – 5 mol %) in Schlenk tube was added 0.50 mL of solvent. The resulting mixture was heated and, after 16 h, the heterogenous mixture was filtered through a short pad of Al_2O_3. The filtrate was concentrated *in vacuo*. The resulting solid (oil) was dissolved in 1.5 mL of $CDCl_3$ and 0.007 mL of dibromomethane (0.1 mmol) was added. The areas of the C–H peak on the carbon 13 position in **17c** and carbon 13 in **18c** were compared to the area of CH_2Br_2 to derive a yield.

Table s2. Optimization of Migration Process

entry	metal salt	mol %	wt %, 4 Å MS	temp (°C)	yield, %[a]
1	None	n.a.	0	150	>95
2	$Rh_2(O_2CC_7H_{15})_4$	5	100	55	33
3	$Rh_2(O_2CC_7H_{15})_4$	5	100	65	63
4	$Rh_2(O_2CC_7H_{15})_4$	5	0	65	53
5	$Rh_2(O_2CC_7H_{15})_4$	3	100	65	39
6	$Rh_2(O_2CC_7H_{15})_4$	5	100	80	90
7	$[Ir(cod)OMe]_2$	5	100	80	0
8	$Rh_2(esp)_2$	5	100	80	20
9	$RhCl(PPh_3)_3$	5	100	80	0
10	$Rh_2(O_2CC_3F_7)_4$	5	100	80	66
11	$RhCl_3$	5	100	80	0
12	$Rh_2(O_2CCH_3)_4$	5	100	80	0
13	$Rh_2(O_2CCF_3)_4$	5	100	80	34
14	$RuCl_3.xH_2O$	5	100	65	trace
15	$RuCl_3$.xH2O	5	0	65	trace
16	Rh octanoate	5	100	65(40h)	90
17	Co TPP	5	100	65	0
18	Ag(OTf)	5	100	65	0
19	$Cu(OTf)_2$	5	100	65	0
20	Rh octanoate (cyclopentane)	5	100	75	59
21	ZnI_2	10	100	65	0
22	$FeBr_2$	20	100	65	0
23	AuCl	10	100	65	0

[a] Isolated yield after Al_2O_3 chromatography.

2. Optimization of Solvent.

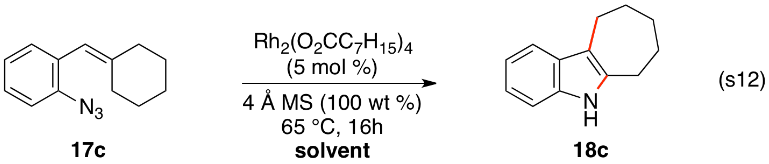

(s12)

Solvent	PhMe	1,2-DCE	1,4-dioxane	DME	DMF
Yield, %[a]	63%	66%	0	0	0

[a] Isolated yield after Al_2O_3 chromatography.

3. Optimized Procedure for Ring Expansion.

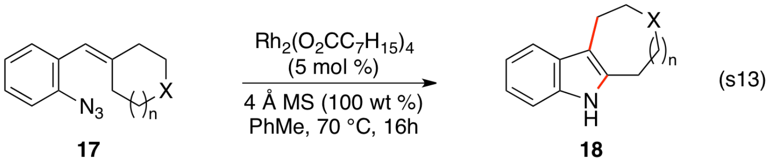

(s13)

To a mixture of 0.026 g of aryl azide **17** (0.1 mmol), 100 % w/w of crushed 4 Å mol sieves, and metal salt ($Rh_2(O_2CC_7H_{15})_4$, 5 mol %) in Schlenk tube was added 0.50 mL of PhMe. The resulting mixture was heated at

80 °C for 16 h, then, the heterogenous mixture was filtered through a short pad of Al_2O_3. The filtrate was concentrated *in vacuo* to afford analytically pure indole.

4. Scope and Limitations of Ring Expansion.

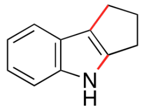

18a

Indole 18a.[11] The general procedure was followed using 0.035 g of styryl azide **17a** (0.189 mmol) and 0.007 g of $Rh_2(O_2CC_7H_{15})_4$ (0.009 mmol) in 0.6 mL of PhMe. Filtration by Al_2O_3 afforded analytically pure compound **18a** (0.029 g, 98%). Indole **18a** was previously reported by Tan and Hartwig.[11] mp 58 - 61 °C; ^1H NMR (500 MHz, $CDCl_3$) δ 7.74 (s, 1H), 7.47 – 7.49 (m, 1H), 7.29 – 7.31 (m, 1H), 7.12 – 7.14 (m, 2H), 2.86 -2.89 (m, 4H), 2.56 – 2.59 (m, 2H); ^{13}C NMR (125 MHz, $CDCl_3$) δ 143.8 (C), 141.0 (C), 124.8 (C), 120.5 (CH), 119.8 (C), 119.5 (CH), 118.5 (CH), 111.4 (CH), 28.7 (CH_2), 25.9 (CH_2), 24.5 (CH_2); IR (thin film): 3400, 2951, 2853, 1467, 1313, 913, 736 cm^{-1}.

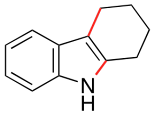

18b

Indole 18b.[13] The general procedure was followed using 0.055 g of styryl azide **17b** (0.276 mmol) and 0.011 g of $Rh_2(O_2CC_7H_{15})_4$ (0.014 mmol) in 0.7 mL of PhMe. Filtration by Al_2O_3 afforded analytically pure compound **18b** (0.044 g, 93%). The spectral data for **18b** matched that reported by Leogane and Lebel:[13] ^1H NMR (500 MHz, $CDCl_3$) δ 7.64 (s, 1H), 7.48 (d, J = 7.5 Hz, 1H), 7.28 (d, J = 7.5 Hz, 1H), 7.08 – 7.15 (m, 2H), 2.73 (t, J = 6.0 Hz, 4H), 1.87 – 1.96 (m, 4H); ^{13}C NMR (125 MHz, $CDCl_3$) δ 135.7 (C), 134.1 (C), 127.9 (C), 121.0 (CH), 119.1 (CH), 117.7 (CH), 110.4 (CH), 110.2 (C), 23.34 (CH_2), 23.26 (CH_2), 21.0 (CH_2) one overlapping aliphatic C not distinguishible; IR (thin film): 3397, 1667, 1603, 1497, 1456, 1276, 911, 749 cm^{-1}.

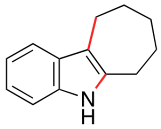

18c

Indole 18c.[14] The general procedure was followed using 0.051 g of styryl azide **17c** (0.239 mmol) and 0.009 g of $Rh_2(O_2CC_7H_{15})_4$ (0.012 mmol) in 0.6 mL of PhMe. Filtration by Al_2O_3 afforded analytically pure compound **18c** (0.041 g, 93%). The spectral data for **18c** matched that reported by Banwell and co-workers:[14] mp 140 - 142 °C; ^1H NMR (500 MHz, $CDCl_3$) δ 7.61 (s, 1H), 7.15 – 7.53 (m, 1H), 7.26 – 7.27 (m, 1H), 7.11 – 7.15 (m, 2H), 2.82 -2.87 (m, 4H), 1.91 – 1.94 (m, 2H), 1.81 – 1.83 (m, 4H); ^{13}C NMR (125 MHz, $CDCl_3$) δ 137.5 (C), 134.3 (C), 129.3 (C), 120.6 (CH), 119.1 (CH), 117.7 (CH), 113.8 (C), 110.3 (CH), 31.9 (CH_2), 29.6 (CH_2), 28.8 (CH_2), 27.6 (CH_2), 24.7 (CH_2); IR (thin film): 3391, 2914, 2844, 1465, 1438, 913, 743 cm^{-1}.

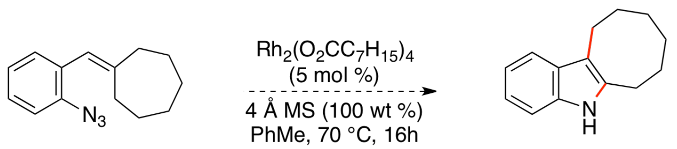

17d **18d** (s14)

Indole 18d. The general procedure was followed using 0.046 g of styryl azide **17d** (0.203 mmol) and 0.008 g of $Rh_2(O_2CC_7H_{15})_4$ (0.010 mmol) in 0.6 mL of PhMe. Analysis of the reaction progress using ^1H NMR spectroscopy revealed that 18% of indole **18d** formed and 35% starting of aryl azide **17d** remained.

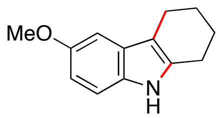

18e

Indole 18e. The general procedure was followed using 0.061 g of styryl azide **17e** (0.266 mmol) and 0.010 g of $Rh_2(O_2CC_7H_{15})_4$ (0.013 mmol) in 0.7 mL of PhMe. Filtration by Al_2O_3 afforded analytically pure compound **18e** (0.054 g, 100%). Spectral data for **18e**: ^1H NMR (500 MHz, $CDCl_3$) δ 7.56 (s, 1H), 7.16 (d, J = 9.0 Hz, 1H), 6.97(d, J = 2.5 Hz, 1H), 6.81 (dd, J = 2.5 Hz, 9.0 Hz, 1H), 3.89 (s, 3H), 2.70 – 2.72 (m, 4H), 1.89 – 1.95 (m, 4H); ^{13}C NMR (125 MHz, $CDCl_3$) δ 153.9 (C), 135.2 (C), 130.8 (C), 128.2 (C), 111.0 (CH), 110.5 (CH), 110.0 (C), 100.4 (CH), 56.0 (CH_3), 23.38 (CH_2), 23.27 (CH_2), 21.0 (CH_2) one overlapping aliphatic carbon signal not distinguishable; IR (thin film): 3281, 2936, 1659, 1492, 1274, 1214, 1030, 732 cm^{-1}. HRMS (EI) m / z calcd for $C_{13}H_{15}ON$ $(M)^+$ 201.11537, found 201.11616.

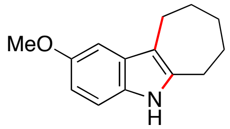

18f

Indole 18f. The general procedure was followed using 0.045 g of styryl azide **17f** (0.201 mmol) and 0.008 g of $Rh_2(O_2CC_7H_{15})_4$ (0.010 mmol) in 0.5 mL of PhMe. Filtration by Al_2O_3 afforded analytically pure compound **18e** (0.039 g, 100%). mp 42 - 46 °C; Spectral data for **18f**: ^1H NMR (500 MHz, $CDCl_3$) δ 7.58 (s, 1H), 7.14 (d, J = 8.5 Hz, 1H), 6.97(d, J = 2.5 Hz, 1H), 6.79 (dd, J = 2.5 Hz, 8.5 Hz, 1H), 3.89 (s, 3H), 2.79 – 2.81(m, 4H), 1.90 – 1.92 (m, 2H), 1.79 – 1.81 (m, 4H); ^{13}C NMR (125 MHz, $CDCl_3$) δ 153.9 (C), 138.6 (C), 129.6 (C), 129.4 (C), 113.6 (C), 110.9 (CH), 110.4 (CH), 100.1 (CH), 56.0 (CH_3), 31.9 (CH_2), 29.7 (CH_2), 28.8 (CH_2), 27.6 (CH_2), 24.8 (CH_2); IR (thin film): 3401, 2916, 1625, 1483, 1452, 913, 745 cm^{-1}. HRMS (EI) m / z calcd for $C_{14}H_{17}ON$ $(M)^+$ 215.13102, found 215.13248.

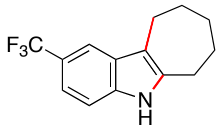

18g

Indole 18g. The general procedure was followed using 0.027 g of styryl azide **17g** (0.009 mmol) and 0.004 g of $Rh_2(O_2CC_7H_{15})_4$ (0.005 mmol) in 0.5 mL of PhMe. Filtration by Al_2O_3 afforded analytically pure compound **18g** (0.021 g, 88%). Spectral data for **18g**: ^1H NMR (500 MHz, $CDCl_3$) δ 7.89 (s, 1H), 7.76 (s, 1H), 7.29 – 7.34 (m, 2H), 2.82 – 2.86 (m, 4H), 1.90 – 1.94 (m, 2H), 1.76 – 1.82 (m, 4H); ^{13}C NMR (125 MHz, $CDCl_3$) δ 139.3 (C), 135.6 (C), 128.7 (C), 125.6 (q, J_{CF} = 270.1 Hz, C), 121.4 (q, J_{CF} = 31.3 Hz, C), 117.4 (q, J_{CF} = 3.6 Hz, CH), 115.4 (q, J_{CF} = 3.5 Hz, CH), 114.7 (C), 110.2 (CH), 31.6 (CH_2), 29.6 (CH_2), 28.5 (CH_2), 27.4 (CH_2), 24.6 (CH_2);

^{19}F NMR ($CDCl_3$, 282 MHz) δ –60.66; IR (thin film): 3474, 3419, 2921, 1626, 1463, 1327, 1108, 1050, 763 cm^{-1}. HRMS (EI) *m / z* calcd for $C_{14}H_{14}F_3N$ $(M)^+$ 253.10783, found 253.10944.

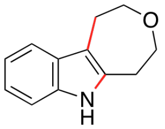

18h

Indole 18h. The general procedure was followed using 0.057 g of styryl azide **17h** (0.265 mmol) and 0.010 g of $Rh_2(O_2CC_7H_{15})_4$ (0.013 mmol) in 0.6 mL of PhMe. Filtration by Al_2O_3 afforded analytically pure compound **18h** (0.048 g, 97%). mp 116 - 119 °C; Spectral data for **18h**: ^1H NMR (500 MHz, $CDCl_3$) δ 7.85 (s, 1H), 7.49 (d, *J* = 7.5 Hz, 1H), 7.28 (d, *J* = 7.5 Hz, 1H), 7.12 – 7.18 (m, 2H), 3.97 – 4.02 (m, 4H), 3.00 – 3.03 (m, 4H); ^{13}C NMR (125 MHz, $CDCl_3$) δ 135.5 (C), 134.8 (C), 128.9 (C), 121.2 (CH), 119.4 (CH), 117.8 (CH), 112.4 (C), 110.4 (CH), 73.1 (CH_2), 70.7 (CH_2), 32.2 (CH_2), 27.6 (CH_2); IR (thin film): 3400, 3294, 2936, 1464, 1113, 913 cm^{-1}. HRMS (EI) *m / z* calcd for $C_{12}H_{13}ON$ $(M)^+$ 187.09972, found 187.10060.

V. Mechanistic Experiments

A. Synthesis of Aryl Azides 27.

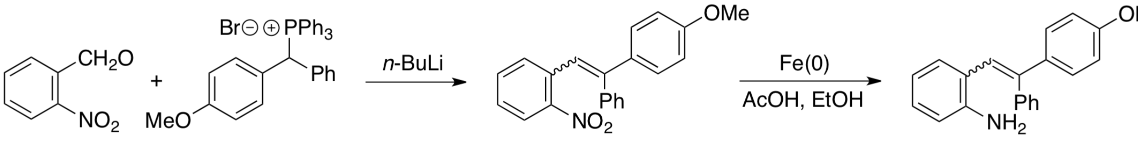

(s15)

Styrene s31. To a solution of 10.4 g of the phosphonium salt (19.3 mmol) in 100 mL of THF at –78 °C, was added 8.5 mL of *n*-BuLi (2.5 M in hexanes, 21.2 mmol) dropwise. After stirring for 1 h at –78 °C, the mixture was warmed to room temperature and stirred additional half an hour. Then 2.65 g of 2-nitrobenzaldehyde (17.5 mmol) was added, and the mixture was heated to reflux. After 16 h, the reaction mixture was diluted with 50 mL of water and extracted with CH_2Cl_2 (3 × 30 mL). The resulting organic phase was washed with 60 mL of brine and dried over Na_2SO_4. The mixture was filtered and the filtrate was concentrated *in vacuo* to afford styrene **s31**, which was carried on to the subsequent Fe-mediated reduction without any additional purification.

Aniline s32. To a solution of nitro-substituted styrene **s31** in 40 mL of AcOH and 40 mL of EtOH was added 8.6 g of Fe powder (154.4 mmol). The resultant mixture was heated to reflux at 80 °C. After 16 h, the reaction mixture was cooled down to ambient temperature. Once cool, the resulting mixture was filtered through a pad of Celite. The filtrate was diluted with 80 mL of water and washed with 3 × 30 mL of dichloromethane. The resulting organic phase was washed with 50 mL of brine and dried over Na_2SO_4. The resulting mixture was filtered, and the filtrate was concentrated in *vacuo*. Purification by MPLC (10:1 hexanes:EtOAc) afforded the product as a yellow viscous oil as an 67:33 mixture of *E*- and *Z*-isomers (1.37 g, 24%, two steps): Spectral data for the major (*E*) isomer: ^1H NMR (500 MHz, $CDCl_3$) δ 7.29 – 7.39 (m, 4H), 7.17 – 7.19 (m, 1H), 7.10 (d, *J* = 8.5 Hz, 2H), 6.89 (d, *J* = 8.5 Hz, 1H), 6.84 (d, *J* = 7.5 Hz, 1H), 6.74 – 6.80 (m, 4H), 6.53 (t, J = 7.5 Hz, 1H), 3.80 (s, 3H), *N-H peak not observed:* ^{13}C NMR (125 MHz, $CDCl_3$) δ 144.5 (C), 143.8 (C), 143.6 (C), 132.3 (C), 131.7 (CH), 130.4 (CH), 128.25 (CH), 128.19 (CH), 127.9 (CH), 127.6 (CH), 123.93 (C), 123.90 (C), 123.8 (CH), 118.3 (CH), 115.5 (CH), 113.6 (CH), 55.2 (CH_3); Selected spectral data for the minor (*Z*) isomer: ^1H NMR (500 MHz, $CDCl_3$) δ 7.29 – 7.39 (m, 4H), 7.24 – 7.26 (m, 3H), 6.96 – 7.02 (m, 3H), 6.64 -6.66 (m, 3H),

6.50 (t, J = 7.5 Hz, 1H), 3.84 (s, 3H), *N-H peak not available*; ^{13}C NMR (125 MHz, $CDCl_3$) δ 144.6 (C), 140.3 (C), 136.0 (C), 130.51 (CH), 130.43 (CH), 129.3 (CH), 127.8 (CH), 127.4 (CH), 122.8 (CH), 118.2 (CH), 115.4 (CH), 55.4 (CH_3) *these are the only visible peaks*; IR (thin film): 3462, 3375, 1739, 1605, 1508, 1242, 1176, 1031, 749 cm^{-1}.

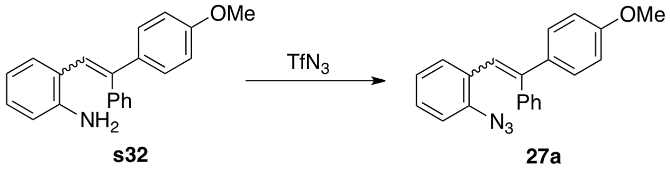

(s16)

Aryl Azide 27a. To a solution of 0.82 g of aniline **s32** (2.7 mmol) in 5 mL of CH_2Cl_2 was added subsequently 24 mg of $CuSO_4$, 1.2 mL of Et_3N, freshly prepared triflyl azide (9 mmol) in 15 mL of CH_2Cl_2, 1 mL of water and 2 mL of MeOH. The resulting mixture was stirred at room temperature. After 16 h,, the reaction mixture was diluted with 15 mL of CH_2Cl_2, and the resulting mixture was neutralized by the addition of a saturated aq. soln. of $NaHCO_3$. Once pH 7 was reached, the resulting mixture was washed with 3 × 10 mL of CH_2Cl_2. The resulting organic phase was washed with 20 mL of brine and dried over Na_2SO_4. After filtration, the filtrate was concentrated *in vacuo*. Purification by MPLC (10:1 hexane:EtOAc) afforded the product as a yellow viscous oil as an 69:31 mixture of *E*- and *Z*-isomers (0.42 g, 48%): Selected spectral data for the major (*E*) isomer: ^{1}H NMR (500 MHz, $CDCl_3$) δ 7.27 – 7.36 (m, 4H), 7.12 – 7.17 (m, 2H), 7.08 (d, *J* = 9.0 Hz, 2H), 6.87 – 6.88 (m, 2H), 6.80 – 6.83 (m, 3H), 3.82 (s, 3H); ^{13}C NMR (125 MHz, $CDCl_3$) δ 159.0 (C), 143.8 (C), 143.5 (C), 138.6 (C), 132.2 (C), 129.8 (C), 131.8 (CH), 130.7 (CH), 129.2 (CH), 128.37 (CH), 128.18 (CH), 128.11 (CH), 127.7 (CH), 124.1 (CH), 118.2 (CH), 113.8 (CH), 55.2 (CH_3); Selected spectral data for the minor (*Z*) isomer: ^{1}H NMR (500 MHz, $CDCl_3$) δ 7.27 – 7.36 (m, 5H), 7.12 – 7.17 (m, 4H), 6.95 – 6.96 (m, 3H), 6.77 – 6.78 (m, 2H), 3.83 (s, 3H); ^{13}C NMR (125 MHz, $CDCl_3$) δ 159.5 (C), 143.7 (C), 140.2 (C), 138.5 (C), 135.8 (C), 129.7 (C), 130.72 (CH), 130.58 (CH), 127.94 (CH), 127.82 (CH), 127.4 (CH), 124.0 (CH), 122.5 (CH), 121.3 (CH), 118.1 (CH), 113.6 (CH), 55.4 (CH_3); IR (thin film): 2117, 2085, 1738, 1604, 1509, 1289, 1246, 1176, 1034, 751 cm^{-1}.

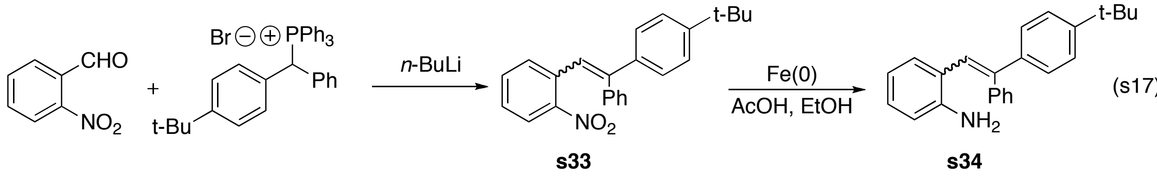

(s17)

Styrene s33. To a solution of 10.8 g of the phosphonium salt (19.1 mmol) in 60 mL of THF at –78 °C, was added 8.4 mL of *n*-BuLi (2.5 M in hexanes, 21.01 mmol) dropwise. After stirring for 1 h at –78 °C, the mixture was warmed to room temperature and stirred additional half an hour. Then 2.69 g of 2-nitrobenzaldehyde (19.1 mmol) was added, and the mixture was heated to reflux. After 16 h, the reaction mixture was diluted with 50 mL of water and extracted with CH_2Cl_2 (3 × 30 mL). The resulting organic phase was washed with 60 mL of brine and dried over Na_2SO_4. The mixture was filtered and the filtrate was concentrated *in vacuo* to afford styrene **s35**, which was carried on to the subsequent Fe-mediated reduction without any additional purification.

Aniline s34. To a solution of nitro-substituted styrene **s33** in 40 mL of AcOH and 40 mL of EtOH was added 8.6 g of Fe powder (152.8 mmol). The resultant mixture was heated to reflux at 80 °C. After 16 h, the reaction mixture was cooled down to ambient temperature. Once cool, the resulting mixture was filtered through a pad of Celite. The filtrate was diluted with 80 mL of water and washed with 3 × 30 mL of dichloromethane. The resulting organic phase was washed with 50 mL of brine and dried over Na_2SO_4. The resulting mixture was

filtered, and the filtrate was concentrated in *vacuo*. Purification by MPLC (10:1 hexanes:EtOAc) afforded the product as a yellow solid (1.5 g, 24%, two steps); Spectral data for the major isomer: ^{1}H NMR (500 MHz, $CDCl_3$) δ 7.37 – 7.39 (m, 2H), 7.31 – 7.33 (m, 2H), 7.25 – 7.27 (m, 3H), 7.19 – 7.21 (m, 2H), 6.97 – 7.00 (m, 1H), 6.84 (s, 1H), 6.77 (d, J = 8.0 Hz, 1H), 6.65 (d, J = 8.0 Hz, 1H), 6.51 (t, J = 7.5 Hz, 1H), 3.74 (br, 2H), 1.37 (s, 9H); ^{13}C NMR (125 MHz, $CDCl_3$) δ 150.8 (C), 144.7 (C), 143.9 (C), 140.4 (C), 140.2 (C), 130.53 (CH), 130.43 (CH), 128.2 (CH), 127.9 (CH), 127.7 (CH), 127.3 (CH), 125.2 (CH), 123.7 (CH), 118.2 (CH), 115.4 (CH), 34.6 (C), 31.4 (CH_3); IR (thin film): 3379, 1617, 1246, 905, 726, 701, 649 cm^{-1}.

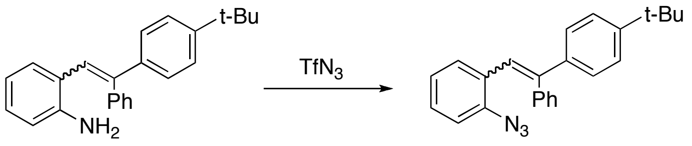

s34 **27b** (s18)

Aryl Azide 27b. To a solution of 1.2 g of aniline **s34** (3.8 mmol) in 5 mL of CH_2Cl_2 was added subsequently 32 mg of $CuSO_4$, 1.6 mL of Et_3N, freshly prepared triflyl azide (12 mmol) in 15 mL of CH_2Cl_2, 1 mL of water and 2 mL of MeOH. The resulting mixture was stirred at room temperature. After 16 h, the reaction mixture was diluted with 15 mL of CH_2Cl_2, and the resulting mixture was neutralized by the addition of a saturated aq. soln. of $NaHCO_3$. Once pH 7 was reached, the resulting mixture was washed with 3 × 10 mL of CH_2Cl_2. The resulting organic phase was washed with 20 mL of brine and dried over Na_2SO_4. After filtration, the filtrate was concentrated *in vacuo*. Purification by MPLC (100:1 hexane:EtOAc) afforded the product as a yellow solid as an 1:1 mixture of *E*- and *Z*-isomers (0.88 g, 66%): Spectral data for *E*- and *Z*- isomers: ^{1}H NMR (500 MHz, $CDCl_3$) δ 7.27 – 7.36 (m, 14H), 7.11 – 7.17 (m, 6H), 7.06 – 7.07 (m, 2H), 7.02 (s, 2H), 6.76 – 6.82 (m, 4H), 1.33 – 1.34 (m, 18H); ^{13}C NMR (125 MHz, $CDCl_3$) δ 150.9 (C), 143.4 (C), 140.1 (C), 138.6 (C), 136.8 (C), 130.78 (CH), 130.73 (CH), 130.56 (CH), 130.1 (CH), 129.70 (C), 129.63 (C), 128.35 (CH), 128.15 (CH), 128.08 (CH), 127.93 (CH), 127.90 (CH), 127.67 (CH), 127.55 (CH), 127.39 (CH), 125.20 (CH), 125.14 (CH), 124.01 (CH), 123.98 (CH), 122.7 (CH), 122.3 (CH), 118.1 (CH), 34.6 (CH_3), 31.4 (CH_3) *only distinguishable peaks*; IR (thin film): 2117, 2085, 1796, 1730, 1287, 908, 733 cm^{-1}. HRMS (EI) m / z calcd for $C_{24}H_{23}N_3$ $(M)^+$ 353.18920, found 353.19017.

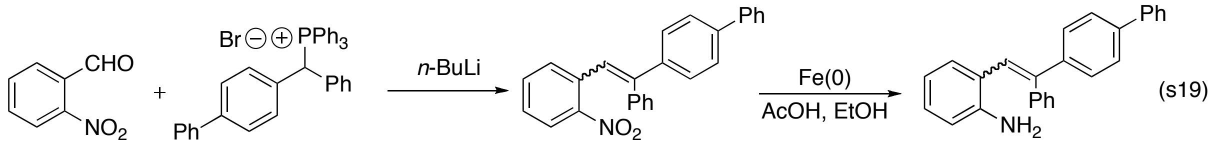

s35 **s36** (s19)

Styrene s35. To a solution of 9.6 g of the phosphonium salt (16.4 mmol) in 60 mL of THF at –78 °C, was added 7.2 mL of n-BuLi (2.5 M in hexanes, 18.1 mmol) dropwise. After stirring for 1 h at –78 °C, the mixture was warmed to room temperature and stirred additional half an hour. Then 2.48 g of 2-nitrobenzaldehyde (16.4 mmol) was added, and the mixture was heated to reflux. After 16 h, the reaction mixture was diluted with 50 mL of water and extracted with CH_2Cl_2 (3 × 30 mL). The resulting organic phase was washed with 60 mL of brine and dried over Na_2SO_4. The mixture was filtered and the filtrate was concentrated *in vacuo* to afford styrene **s33**, which was carried on to the subsequent Fe-mediated reduction without any additional purification.

Aniline s36. To a solution of nitro-substituted styrene **s35** in 30 mL of AcOH and 30 mL of EtOH was added 7.3 g of Fe powder (131.2 mmol). The resultant mixture was heated to reflux at 80 °C. After 16 h, the reaction

mixture was cooled down to ambient temperature. Once cool, the resulting mixture was filtered through a pad of Celite. The filtrate was diluted with 70 mL of water and washed with 3 × 30 mL of dichloromethane. The resulting organic phase was washed with 50 mL of brine and dried over Na_2SO_4. The resulting mixture was filtered, and the filtrate was concentrated in *vacuo*. Purification by MPLC (10:1 hexanes:EtOAc) afforded the product as a yellow solid (602 mg, 11%, two steps); Spectral data for the major isomer: ^{1}H NMR (500 MHz, $CDCl_3$) δ 7.63 – 7.70 (m, 4H), 7.47 – 7.56 (m, 5H), 7.41 – 7.43 (m, 1H), 7.27 – 7.34 (m, 4H), 7.03 – 7.07 (m, 1H), 6.95 (s, 1H), 6.86 (d, J = 7.5 Hz, 1H), 6.70 – 6.71 (m, 1H), 6.56 – 6.60 (m, 1H), 3.92 (br, 2H): ^{13}C NMR (125 MHz, $CDCl_3$) δ 144.8 (C), 143.7 (C), 142.4 (C), 140.8 (C), 140.5 (C), 131.1 (CH), 130.63 (CH), 130.48 (CH), 128.9 (CH), 128.6 (CH), 128.38 (CH), 128.20 (CH), 127.6 (CH), 127.12 (CH), 127.01 (CH), 124.4 (CH), 123.7 (C), 118.3 (CH), 115.6 (CH); IR (thin film): 3457, 1739, 1486, 1454, 1366, 1216, 905 cm^{-1}.

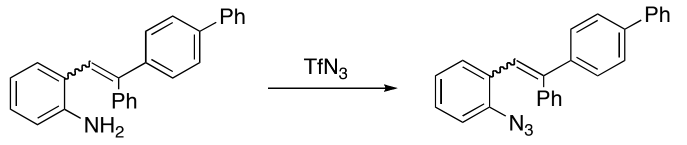

s36 **27c** (s20)

Aryl Azide 27c. To a solution of 0.55 g of aniline **s36** (1.6 mmol) in 5 mL of CH_2Cl_2 was added subsequently 16 mg of $CuSO_4$, 0.8 mL of Et_3N, freshly prepared triflyl azide (6 mmol) in 15 mL of CH_2Cl_2, 1 mL of water and 2 mL of MeOH. The resulting mixture was stirred at room temperature. After 16 h, the reaction mixture was diluted with 15 mL of CH_2Cl_2, and the resulting mixture was neutralized by the addition of a saturated aq. soln. of $NaHCO_3$. Once pH 7 was reached, the resulting mixture was washed with 3 × 10 mL of CH_2Cl_2. The resulting organic phase was washed with 20 mL of brine and dried over Na_2SO_4. After filtration, the filtrate was concentrated *in vacuo*. Purification by MPLC (100:1 hexane:EtOAc) afforded the product as a pale solid (0.26 g, 43%). Spectral data for *E*- and *Z* isomer mixture: ^{1}H NMR (500 MHz, $CDCl_3$) δ 7.63 – 7.66 (m, 4H), 7.54 – 7.59 (m, 4H), 7.10 – 7.49 (m, 25H), 7.06 (s, 1H), 6.91 – 6.93 (m, 1H), 6.78 – 6.84 (m, 3H); ^{13}C NMR (125 MHz, $CDCl_3$) δ 143.8 (C), 143.7 (C), 143.2 (C), 142.1 (C), 140.7 (C), 140.6 (C), 140.56 (C), 140.1 (C), 140.0 (C), 139.0 (C), 138.7 (C), 131.1 (CH), 130.84 (CH), 130.76 (CH), 130.6 (CH), 129.53, 129.46, 128.8 (CH), 128.5 (CH), 128.4 (CH), 128.3 (CH), 128.2 (CH), 127.9 (CH), 127.6 (CH), 127.4 (CH), 127.4 (CH), 127.1 (CH), 126.99 (CH), 126.94 (CH), 124.2 (CH), 124.1 (CH), 123.3 (CH), 123.0 (CH), 118.2 (CH), 118.2 (CH) *only distinguishable peaks*; IR (thin film): 2117, 2085, 1738, 1604, 1509, 1289, 1246, 1176, 1034, 751 cm^{-1}. HRMS (EI) *m* / *z* calcd for $C_{26}H_{19}N_3$ $(M)^+$ 373.15790, found 373.15891.

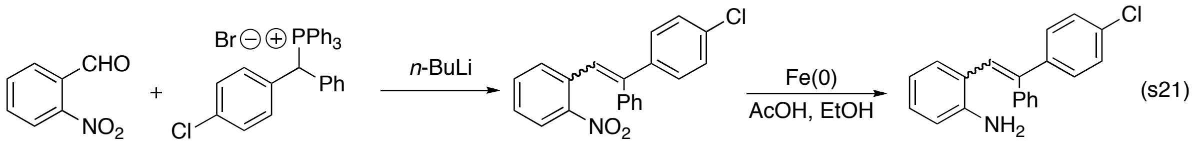

s37 **s38** (s21)

Styrene s37. To a solution of 8.5 g of the phosphonium salt (15.7 mmol) in 50 mL of THF at –78 °C, was added 6.9 mL of *n*-BuLi (2.5 M in hexanes, 17.3 mmol) dropwise. After stirring for 1 h at –78 °C, the mixture was warmed to room temperature and stirred additional half an hour. Then 2.4 g of 2-nitrobenzaldehyde (15.7 mmol) was added, and the mixture was heated to reflux. After 16 h, the reaction mixture was diluted with 50 mL of water and extracted with CH_2Cl_2 (3 × 30 mL). The resulting organic phase was washed with 60 mL of brine and dried over Na_2SO_4. The mixture was filtered and the filtrate was concentrated *in vacuo* to afford styrene **s37**, which was carried on to the subsequent Fe-mediated reduction without any additional purification.

Aniline s38. To a solution of nitro-substituted styrene **s37** in 40 mL of AcOH and 40 mL of EtOH was added 7.1 g of Fe powder (125.6 mmol). The resultant mixture was heated to reflux at 80 °C. After 16 h, the reaction mixture was cooled down to ambient temperature. Once cool, the resulting mixture was filtered through a pad of Celite. The filtrate was diluted with 80 mL of water and washed with 3 × 30 mL of dichloromethane. The resulting organic phase was washed with 50 mL of brine and dried over Na_2SO_4. The resulting mixture was filtered, and the filtrate was concentrated in *vacuo*. Purification by MPLC (10:1 hexanes:EtOAc) afforded the product as a pale solid (1024 mg, 21%, two steps); Spectral data for the major isomer: ^{1}H NMR (500 MHz, $CDCl_3$) δ 7.12 – 7.36 (m, 9H), 6.98 – 7.04 (m, 1H), 6.81 – 6.84 (m, 1H), 6.77 – 6.78 (m, 1H), 6.66 – 6.68 (m, 1H), 6.50 – 6.56 (m, 1H), 3.76 (br, 2H); spectral data for both the *E*- and *Z*-isomer: ^{13}C NMR (125 MHz, $CDCl_3$) δ 144.74 (C), 144.71 (C), 143.9 (C), 143.02 (C), 142.96 (C), 141.9 (C), 139.6 (C), 138.5 (C), 133.5 (C), 133.2 (C), 131.9 (CH), 130.45 (CH), 130.35 (CH), 130.29 (CH), 129.4 (CH), 128.52 (CH), 128.38 (CH), 128.36 (CH), 128.30 (CH), 128.13 (CH), 127.9 (CH), 127.6 (CH), 125.0 (CH), 124.8 (CH), 118.34 (CH), 118.28 (CH), 115.59 (CH), 115.54 (CH); IR (thin film): 3463, 3378, 1614, 1485, 1089, 728 cm^{-1}.

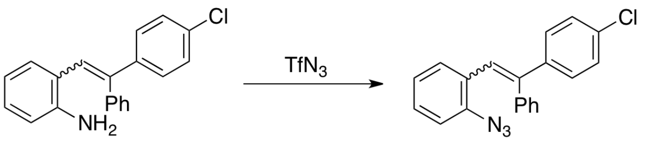

s38 **27d** (s22)

Aryl Azide 27d. To a solution of 0.90 g of aniline **s38** (3.0 mmol) in 5 mL of CH_2Cl_2 was added subsequently 24 mg of $CuSO_4$, 1.2 mL of Et_3N, freshly prepared triflyl azide (9 mmol) in 15 mL of CH_2Cl_2, 1 mL of water and 2 mL of MeOH. The resulting mixture was stirred at room temperature. After 16 h, the reaction mixture was diluted with 15 mL of CH_2Cl_2, and the resulting mixture was neutralized by the addition of a saturated aq. soln. of $NaHCO_3$. Once pH 7 was reached, the resulting mixture was washed with 3 × 10 mL of CH_2Cl_2. The resulting organic phase was washed with 20 mL of brine and dried over Na_2SO_4. After filtration, the filtrate was concentrated *in vacuo*. Purification by MPLC (100:1 hexane:EtOAc) afforded the product as a yellow viscous oil (0.44 g, 45%): Spectral data for the mixture: ^{1}H NMR (500 MHz, $CDCl_3$) δ 7.10 – 7.37 (m, 22H), 6.96 – 7.04 (m, 2H), 6.75 – 6.84 (m, 4H); ^{13}C NMR (125 MHz, $CDCl_3$) δ 143.0 (C), 142.7 (C), 141.6 (C), 139.6 (C), 138.78 (C), 138.74 (C), 138.5 (C), 133.7 (C), 133.4 (C), 132.0 (CH), 130.7 (CH), 130.51 (CH), 130.41 (CH), 130.1 (CH), 129.25 (CH), 129.17 (CH), 128.69 (CH), 128.55 (CH), 128.41 (CH), 128.36 (CH), 128.31 (CH), 128.0 (CH), 127.7 (CH), 126.6 (CH), 125.6 (CH), 124.24 (CH), 124.09 (CH), 123.8 (CH), 123.4 (CH), 118.30 (CH), 118.27 (CH), 118.18 (CH); IR (thin film): 2117, 2085, 1738, 1604, 1509, 1289, 1246, 1176, 1034, 751 cm^{-1}. HRMS (EI) *m* / *z* calcd for $C_{20}H_{14}N_3Cl$ $(M)^+$ 331.08762, found 331.08665.

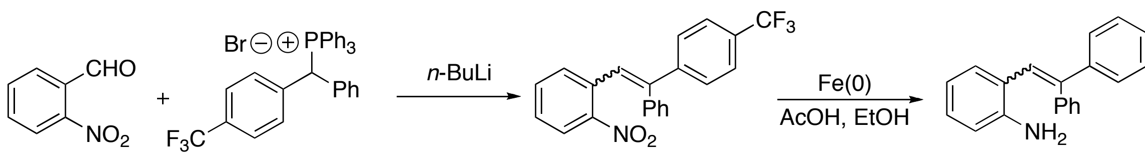

s39 s40 (s23)

Styrene s39. To a solution of 3.46 g of the phosphonium salt (6 mmol) in 60 mL of THF at –78 °C, was added 2.4 mL of *n*-BuLi (2.5 M in hexanes, 6 mmol) dropwise. After stirring for 1 h at –78 °C, the mixture was warmed to room temperature and stirred additional half an hour. Then 0.91 g of 2-nitrobenzaldehyde (6 mmol) was added, and the mixture was heated to reflux. After 16 h, the reaction mixture was diluted with 50 mL of water and extracted with CH_2Cl_2 (3 × 30 mL). The resulting organic phase was washed with 60 mL of brine and dried over Na_2SO_4. The mixture was filtered and the filtrate was concentrated *in vacuo* to afford styrene **s31**, which was carried on to the subsequent Fe-mediated reduction without any additional purification.

Aniline s40. To a solution of nitro-substituted styrene **s39** in 15 mL of AcOH and 15 mL of EtOH was added 2.7 g of Fe powder (48 mmol). The resultant mixture was heated to reflux at 80 °C. After 16 h, the reaction mixture was cooled down to ambient temperature. Once cool, the resulting mixture was filtered through a pad of Celite. The filtrate was diluted with 20 mL of water and washed with 3 × 10 mL of dichloromethane. The resulting organic phase was washed with 20 mL of brine and dried over Na_2SO_4. The resulting mixture was filtered, and the filtrate was concentrated in *vacuo*. Purification by MPLC (10:1 hexanes:EtOAc) afforded the product as a yellow solid (190 mg, 12%, two steps); ^{1}H NMR (500 MHz; $CDCl_3$) δ 7.69 (d, J = 8.5 Hz, 2.4H), 7.61 (d, J = 8.0 Hz, 1.6H), 7.57 (d, J = 8.5 Hz, 2.4H), 7.45 (s, 4H), 7.42 (d, J = 8.0 Hz, 1.6H), 7.38 – 7.37 (m, 3.6H), 7.30 – 7.29 (m, 2.4H), 7.11 (t, J = 7.5 Hz, 2H), 7.01 (d, J = 8.0 Hz, 2H); 6.92 (d, J = 8.0 Hz, 1.2H), 6.84 (d, J = 8.0 Hz, 0.8H), 6.74 (dd, J = 8.0, 3.5 Hz, 2H), 6.62 (t, J = 8.0 Hz, 2H), 3.83 (br, 4H); ^{13}C NMR (125 MHz, $CDCl_3$) δ 147.2 (C), 145.1 (C), 145.0 (C), 144.1 (C), 142.9 (C), 142.8 (C), 142.8 (C), 139.5 (C), 131.1 (CH), 130.6 (CH), 130.5 (CH), 130.4 (CH), 128.7 (CH), 128.6 (CH), 128.6 (CH), 128.5 (CH), 128.2 (CH), 128.2 (CH), 128.0 (CH), 126.4 (CH), 125.9 (CH), 125.3 (q, J_{CF} = 3.5 Hz, CF_3), 123.4 (C), 123.3 (C), 123.2 (C), 123.0 (C), 118.4 (CH), 118.4 (CH), 115.8 (CH), 115.7 (CH); *only distinguishable peaks*. HRMS (EI) *m* / *z* calcd for $C_{21}H_{16}F_3N$ $(M)^+$ 339.12348, found 339.12435.

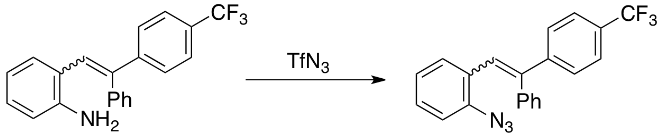

s40 27e (s24)

Aryl Azide 27e. To a solution of 0.17 g of aniline **s40** (0.5 mmol) in 5 mL of CH_2Cl_2 was added subsequently 6 mg of $CuSO_4$, 0.3 mL of Et_3N, freshly prepared triflyl azide (2 mmol) in 5 mL of CH_2Cl_2, 1 mL of water and 2 mL of MeOH. The resulting mixture was stirred at room temperature. After 16 h, the reaction mixture was diluted with 10 mL of CH_2Cl_2, and the resulting mixture was neutralized by the addition of a saturated aq. soln. of $NaHCO_3$. Once pH 7 was reached, the resulting mixture was washed with 3 × 10 mL of CH_2Cl_2. The resulting organic phase was washed with 20 mL of brine and dried over Na_2SO_4. After filtration, the filtrate was concentrated *in vacuo*. Purification by MPLC (100:1 hexane:EtOAc) afforded the product as a yellow viscous oil (0.076 g, 42%): ^{1}H NMR (500 MHz; $CDCl_3$) δ 7.59 (d, J = 8.0 Hz, 2.4H), 7.54 (d, J = 8.0 Hz, 1.6H), 7.46 (s, 2.4H), 7.44 (s, 1.6H), 7.36 – 7.28 (m, 8H), 7.23 – 7.10 (m, 8H), 6.81 – 6.77 (m, 4H); ^{13}C NMR (125 MHz, $CDCl_3$) δ 146.7 (C), 144.0 (C), 142.8 (C), 142.4 (C), 139.3 (C), 138.9 (C), 131.0 (CH), 130.7 (CH), 130.5 (CH),

130.5 (C), 128.9 (C), 128.6 (CH), 128.6 (CH), 128.4 (CH), 128.2 (CH), 128.1 (CH), 128.0 (CH), 127.9 (CH), 125.3 (q, J = 3.5 Hz, CF_3), 125.2 (q, J_{CF} = 3.5 Hz, CF_3), 124.9 (CH), 124.5 (CH), 124.3 (CH), 124.1 (CH), 118.3 (CH), 118.2 (CH) *only distinguishable peaks*. IR (thin film): 1752, 1727, 1368, 1228, 1216, 750 cm^{-1}.

B. Syntheses of 2,3-Diarylindoles

2-Phenyl-3-arylindoles were synthesized from alkynyltrifluoroacetanilide **s41** following the method reported by Cacchi and co-workers.[15]

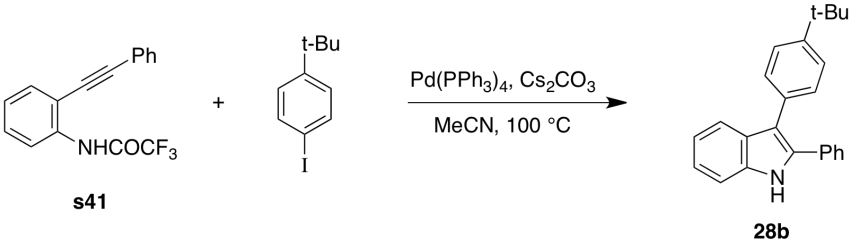

(s25)

Indole 28b.[15] To a solution of 0.59 g of acetylene (2.0 mmol) in 15 mL of MeCN was added subsequently 1.43 g of Cs_2CO_3, 0.1 mol of $Pd(PPh_3)_4$ (5 mol %), 1.14 g of 4-*tert*-butyliodobenzene (4.4 mmol). The resulting mixture was stirred at 100 °C until complete conversion (2h). After this time, the reaction mixture was cooled to room temperature, diluted with 30 mL of water and extracted with CH_2Cl_2 (3 × 20 mL). The resulting organic phase was washed with 60 mL of brine and dried over Na_2SO_4. Purification by MPLC (30:1 hexane:EtOAc) afforded the product **28b** as a yellow solid (0.59 g, 91%). The spectral data matched that reported by Cacchi and co-workers:[15] ^{1}H NMR (500 MHz, $CDCl_3$) δ 8.18 (br, 1H), 7.76 (d, J = 8.0 Hz, 1H), 7.40 – 7.48 (m, 7H), 7.26 – 7.36 (m, 4H), 7.17 – 7.20 (m, 1H) 1.49 (s, 9H); ^{13}C NMR (125 MHz, $CDCl_3$) δ 149.0 (C), 136.0 (C), 134.0 (C), 133.0 (C), 132.0 (C), 129.7 (CH), 129.0 (C), 128.7 (CH), 128.3 (CH), 127.6 (CH), 125.5 (CH), 122.7 (CH), 120.3 (CH), 120.0 (CH), 115.0 (C), 110.9 (CH), 34.6 (C), 31.5 (CH_3); IR (thin film): 3457, 1761, 1442, 1378, 1223, 732 cm^{-1}. HRMS (EI) m / z calcd for $C_{24}H_{23}N$ $(M)^+$ 325.18404, found 325.18305.

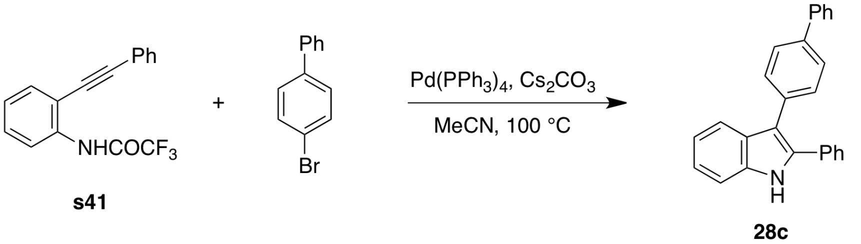

(s26)

Indole 28c.[15] To a solution of 0.59 g of acetylene (2.0 mmol) in 15 mL of MeCN was added subsequently 1.43 g of Cs_2CO_3, 0.1 mol of $Pd(PPh_3)_4$ (5 mol %), 1.03 g of 4-bromobiphenyl (4.4 mmol). The resulting mixture was stirred at 100 °C until complete conversion (2h). After this time, the reaction mixture was cooled to room temperature, diluted with 30 mL of water and extracted with CH_2Cl_2 (3 × 20 mL). The resulting organic phase was washed with 60 mL of brine and dried over Na_2SO_4. Purification by MPLC (30:1 hexane:EtOAc) afforded the product **28c** as a yellow solid (0.54 g, 78%). Spectral data matched that reported by Cacchi and co-workers:[15] ^{1}H NMR (500 MHz, $CDCl_3$) δ 8.25 (br, 1H), 7.78 (d, J = 7.5 Hz, 1H), 7.69 (d, J = 8.0 Hz, 2H), 7.65 (d, J = 8.0 Hz, 2H), 7.55 (d, J = 7.5 Hz, 2H), 7.45 – 7.55 (m, 5H), 7.22 – 7.39 (m, 5H), 7.19 – 7.22 (m, 1H); ^{13}C NMR (125 MHz, $CDCl_3$) δ 141.0 (C), 138.8 (C), 136.0 (C), 134.29 (C), 134.20 (C), 132.8 (C), 130.5 (CH),

128.81 (CH), 128.76 (CH), 128.3 (CH), 127.8 (CH), 127.23 (CH), 127.18 (CH), 126.99 (CH), 122.8 (CH), 120.6 (CH), 119.8 (CH), 114.6 (C), 111.0 (CH); IR (thin film): 3415, 1770, 1455, 1248, 903, 725, 698, 649 cm^{-1}. HRMS (EI) *m / z* calcd for $C_{26}H_{19}N$ $(M)^+$ 345.15175, found 345.25258.

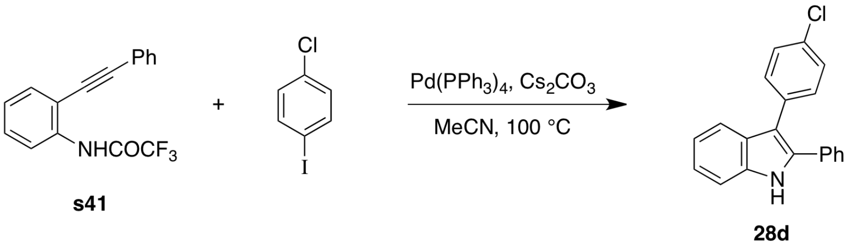

(s27)

Indole 28d.[15] To a solution of 0.59 g of acetylene (2.0 mmol) in 15 mL of MeCN was added subsequently 1.43 g of Cs_2CO_3, 0.1 mol of $Pd(PPh_3)_4$ (5 mol %), 1.05 g of 1-chloro-4-iodobenzene (4.4 mmol). The resulting mixture was stirred at 100 °C until complete conversion (2h). After this time, the reaction mixture was cooled to room temperature, diluted with 30 mL of water and extracted with CH_2Cl_2 (3 × 20 mL). The resulting organic phase was washed with 60 mL of brine and dried over Na_2SO_4. Purification by MPLC (30:1 hexane:EtOAc) afforded the product **28d** as a yellow solid (0.53 g, 88%). The spectral data matched that reported by Cacchi and co-workers:[15] ^{1}H NMR (500 MHz, $CDCl_3$) δ 8.23 (br, 1H), 7.68 (d, *J* = 8.0 Hz, 1H), 7.34 -7.45 (m, 10H), 7.29 (t, J = 7.5 Hz, 1H), 7.20 – 7.23 (m, 1H); ^{13}C NMR (125 MHz, $CDCl_3$) δ 135.9 (C), 134.4 (C), 133.7 (C), 132.4 (C), 132.1 (C), 131.4 (CH), 128.88 (CH), 128.84 (CH), 128.5 (C), 128.3 (CH), 128.0 (CH), 122.9 (CH), 120.7 (CH), 119.5 (CH), 113.8 (C), 111.1 (CH); IR (thin film): 3411, 1770, 1601, 1500, 1248, 1091, 1014, 905, 728 cm^{-1}. HRMS (EI) *m / z* calcd for $C_{20}H_{14}NCl$ $(M)^+$ 303.08147, found 303.08070.

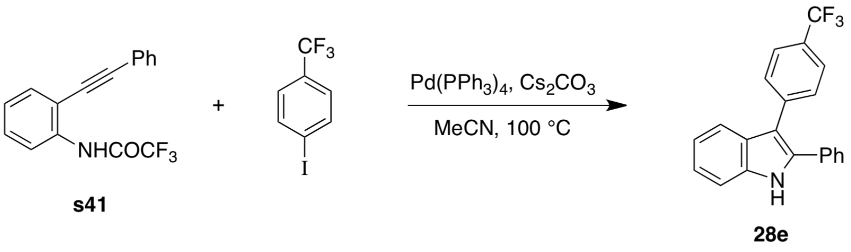

(s28)

Indole 28e.[16] To a solution of 0.59 g of acetylene (2.0 mmol) in 15 mL of MeCN was added subsequently 1.43 g of Cs_2CO_3, 0.1 mol of $Pd(PPh_3)_4$ (5 mol %), 0.97 g of 4-trifluromethyliodobenzene (4.4 mmol). The resulting mixture was stirred at 100 °C until complete conversion (2h). After this time, the reaction mixture was cooled to room temperature, diluted with 30 mL of water and extracted with CH_2Cl_2 (3 × 20 mL). The resulting organic phase was washed with 60 mL of brine and dried over Na_2SO_4. Purification by MPLC (30:1 hexane:EtOAc) afforded the product **28e** as a yellow solid (0.63 g, 94%). The spectral data matched that reported by Wang and co-workers:[16] ^{1}H NMR (500 MHz, $CDCl_3$) δ 8.29 (br, 1H), 7.71 (d, *J* = 8.0 Hz, 1H), 7.65 (d, *J* = 8.0 Hz, 2H), 7.57 (d, *J* = 8.0 Hz, 2H), 7.36 – 7.47 (m, 6H), 7.29 – 7.32 (m, 1H), 7.20 – 7.24 (m, 1H); ^{13}C NMR (125 MHz, $CDCl_3$) δ 139.1 (C), 136.0 (C), 135.0 (C), 132.2 (C), 130.2 (CH), 129.0 (CH), 128.4 (CH), 128.2 (CH), 128.1 (d, J_{CF} = 38.5 Hz, CH), 125.5 (d, J_{CF} = 3.6 Hz, CH), 124.5 (d, J_{CF} = 270.0 Hz, C), 123.1 (C), 121.3 (C), 120.9 (CH), 119.3 (CH), 113.6 (C), 111.2 (CH); IR (thin film): 3405, 1614, 1448, 1321, 1108, 1065, 905, 850 cm^{-1}. HRMS (EI) *m / z* calcd for $C_{20}H_{14}NF_3$ $(M)^+$ 337.10783, found 337.10868.

C. Intramolecular Competition Experiments.

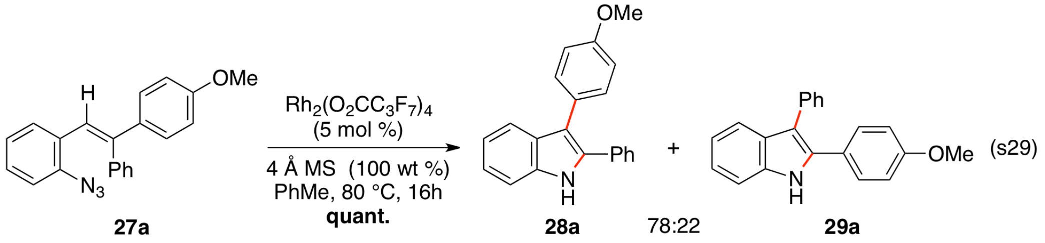

To a mixture of 0.023 g of aryl azide **27a** (0.07 mmol), 0.023 g of crushed 4 Å mol sieves, and 0.004 g of $Rh_2(O_2CC_3F_7)_4$ (0.004 mol) in Schlenk tube was added 0.50 mL of PhMe. The resulting mixture was heated at 80 °C, after 16 h, the heterogenous mixture was filtered through a short pad of Al_2O_3. The filtrate was concentrated *in vacuo* to afford 0.021 g of a mixture of **28a** and **29a** (78:22). The resulting solid was dissolved in 1.5 mL of $CDCl_3$. The areas of the C–H peak on the methoxy position were compared to derive a ratio (78:22). The products were identified by comparison with published ^{1}H NMR spectra of **28a.**[17]

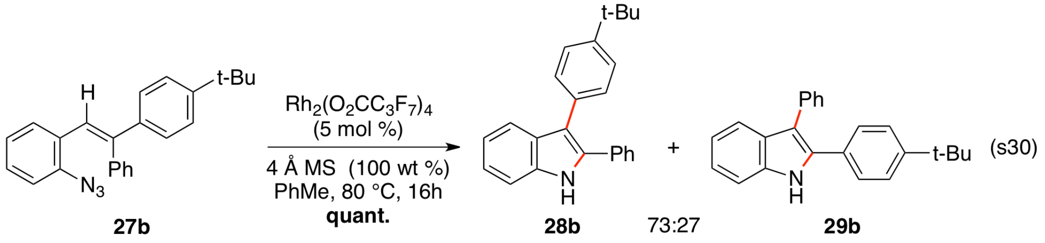

To a mixture of 0.058 g of aryl azide **27b** (0.16 mmol), 0.058 g of crushed 4 Å mol sieves, and 0.009 g of $Rh_2(O_2CC_3F_7)_4$ (0.008 mol) in Schlenk tube was added 0.50 mL of PhMe. The resulting mixture was heated at 80 °C, after 16 h, the heterogenous mixture was filtered through a short pad of Al_2O_3. The filtrate was concentrated *in vacuo* to afford 0.053 g of a mixture of **28b** and **29b** (73:27). The resulting solid was dissolved in 1.5 mL of $CDCl_3$. The areas of the C–H peak on the aromatic region were compared to derive a ratio (73:27). The products were identified by comparison with ^{1}H NMR spectra of **28b**.

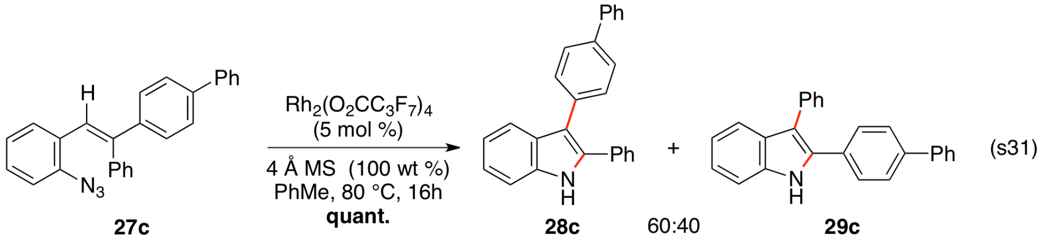

To a mixture of 0.060 g of aryl azide **27c** (0.16 mmol), 0.060 g of crushed 4 Å mol sieves, and 0.009 g of $Rh_2(O_2CC_3F_7)_4$ (0.008 mol) in Schlenk tube was added 0.50 mL of PhMe. The resulting mixture was heated at 80 °C, after 16 h, the heterogenous mixture was filtered through a short pad of Al_2O_3. The filtrate was concentrated *in vacuo* to afford 0.054 g of a mixture of **28c** and **29c** (60:40). The resulting solid was dissolved in 1.5 mL of $CDCl_3$. The areas of the C–H peak on the aromatic region were compared to derive a ratio (60:40). The products were identified by comparison with ^{1}H NMR spectra of **28c**.

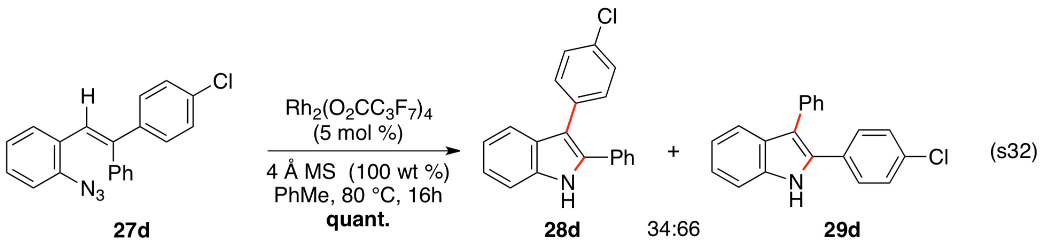

27d   28d   34:66   29d

To a mixture of 0.043 g of aryl azide **27d** (0.13 mmol), 0.043 g of crushed 4 Å mol sieves, and 0.007 g of $Rh_2(O_2CC_3F_7)_4$ (0.006 mol) in Schlenk tube was added 0.50 mL of PhMe. The resulting mixture was heated at 80 °C, after 16 h, the heterogenous mixture was filtered through a short pad of Al_2O_3. The filtrate was concentrated *in vacuo* to afford 0.039 g of a mixture of **28d** and **29d** (34:66). The resulting solid was dissolved in 1.5 mL of $CDCl_3$. The areas of the C–H peak on the aromatic region were compared to derive a ratio (34:66). The products were identified by comparison with ^{1}H NMR spectra of **28d**.

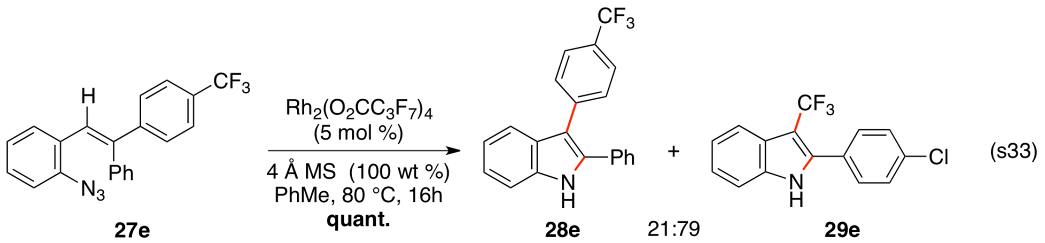

27e   28e   21:79   29e

To a mixture of 0.040 g of aryl azide **27e** (0.11 mmol), 0.040 g of crushed 4 Å mol sieves, and 0.006 g of $Rh_2(O_2CC_3F_7)_4$ (0.005 mol) in Schlenk tube was added 0.50 mL of PhMe. The resulting mixture was heated at 80 °C, after 16 h, the heterogenous mixture was filtered through a short pad of Al_2O_3. The filtrate was concentrated *in vacuo* to afford 0.032 g of a mixture of **28e** and **29e** (21:79). The resulting solid was dissolved in 1.5 mL of $CDCl_3$. The areas of the C–H peak on the aromatic region were compared to derive a ratio (21:79). The products were identified by comparison with ^{1}H NMR spectra of **28e**.

D. Correlation of Product Ratio with Hammett Plot.

The log of the product ratios were correlated with Hammett σ-values to ascertain the existence of any linear free energy relationships.

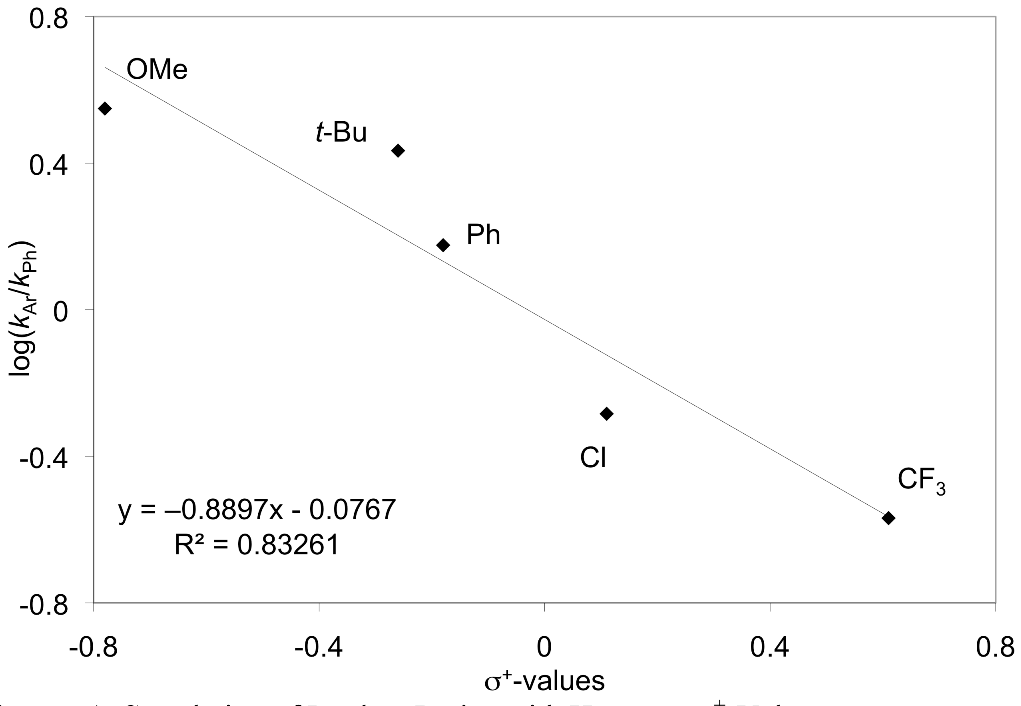

Figure s1. Correlation of Product Ratios with Hammett σ^+-Values.

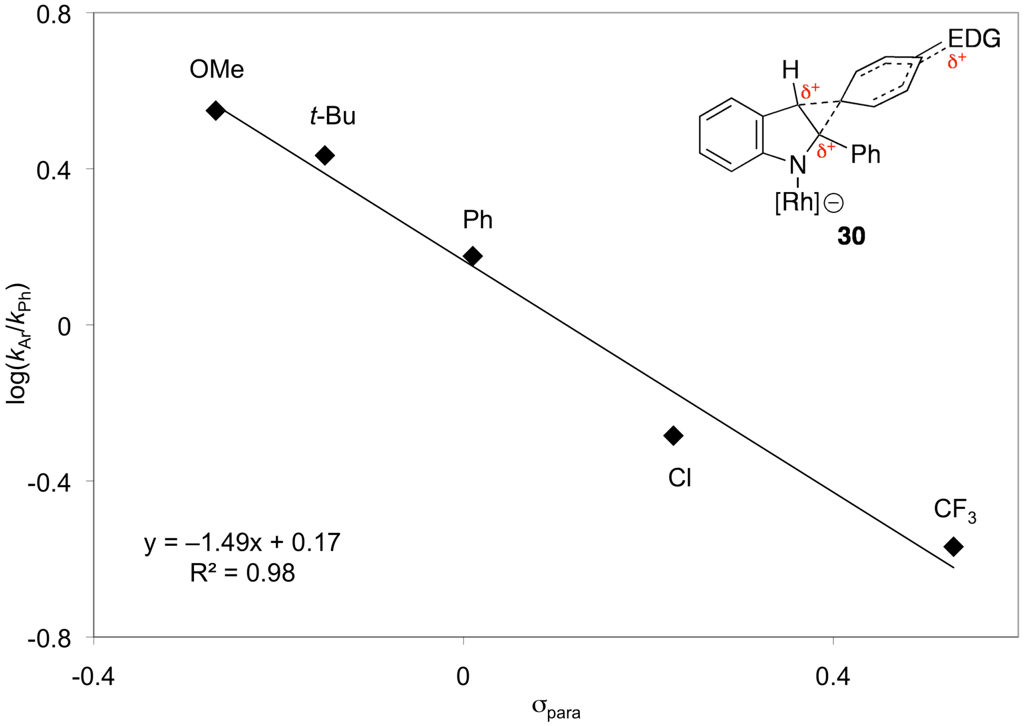

Figure s2. Correlation of Product Ratios with Hammett σ_{para}-Values.

D. Intermolecular Competition Experiment.

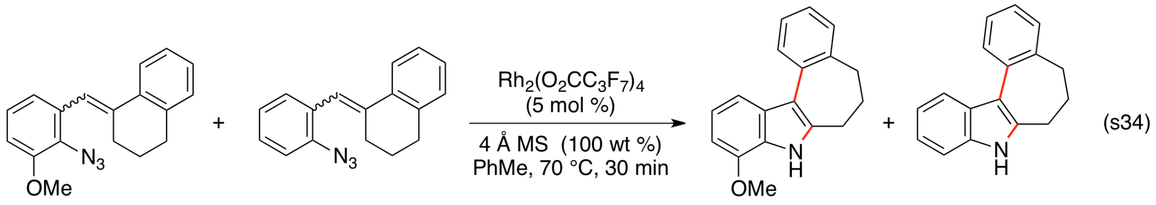

11a 1 : 1 **8** *60% conversion* **12a** 84 : 16 **9**

To a mixture of 0.013 g of styryl azide **11a** (0.05 mmol) and 0.0145 g of styryl azide **8** (0.05 mmol), 100 % w/w of crushed 4 Å mol sieves, and $Rh_2(hpfb)_4$ (5 mol %) in Schlenk tube was added 0.50 mL of PhMe. The resulting mixture was heated at 70 °C. After 30 min, the heterogenous mixture was filtered through a short pad of Al_2O_3. The filtrate was concentrated *in vacuo* to afford analytically pure indoles. Analysis of the ^{1}H NMR spectrum showed 98% conversion of the styryl azide **11a** into **12a** and 23% conversion of the styryl azide **8** into indole **9**. ^{1}H NMR (500 MHz, $CDCl_3$) δ 4.02 (s, 3 H) vs 3.94 (s, 0.0734 H) vs 7.07 – 7.08 (m, 0.774 H) = **12a** vs **11a** vs **8**.

D. Examination of Rates of Reaction of *E*- and *Z*-Isomers.

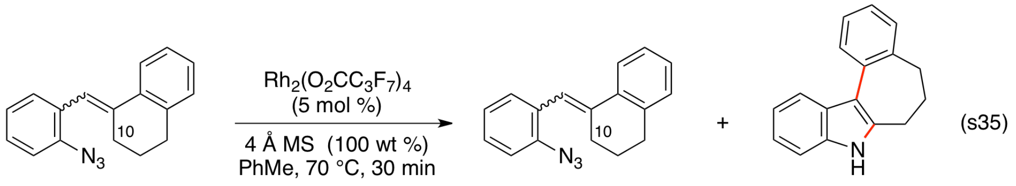

8 (5.3 : 1 *E*:*Z*) *35% conversion* **8** (5.4 : 1 *E*:*Z*) **9**

To a mixture of 0.043 g of aryl azide **8** (0.16 mmol), 0.043 g of crushed 4 Å mol sieves, and 0.009 g of $Rh_2(O_2CC_3F_7)_4$ (0.008 mol) in Schlenk tube was added 0.60 mL of PhMe. The resulting mixture was heated at 70 °C, after 30 min, the heterogenous mixture was filtered through a short pad of Al_2O_3. The filtrate was concentrated *in vacuo*. The resulting solid (oil) was dissolved in 1.5 mL of $CDCl_3$. The areas of the C–H peak on the carbon **9** in starting material aryl azide **8** was compared to derive a *E:Z* ratio.

VI. References.

(1) Pangborn, A. B.; Giardello, M. A.; Grubbs, R. H.; Rosen, R. K.; Timmers, F. J. *Organometallics* **1996**, *15*, 1518-1520.
(2) Stokes, B. J.; Vogel, C. V.; Urnezis, L. K.; Pan, M.; Driver, T. G. *Org. Lett.* **2010**, *12*, 2884-2887.
(3) Brawn, R. A.; Welzel, M.; Lowe, J. T.; Panek, J. S. *Org. Lett.*, *12*, 336-339.
(4) Main, C. A.; Petersson, H. M.; Rahman, S. S.; Hartley, R. C. *Tetrahedron* **2007**, *64*, 901-914.
(5) Hill, T. J.; Hughes, R. K.; Scott, L. T. *Tetrahedron* **2008**, *64*, 11360-11369.
(6) Chong, P. Y.; Janicki, S. Z.; Petillo, P. A. *J. Org. Chem.* **1998**, *63*, 8515-8521.
(7) Liu, Q.; Tor, Y. *Org. Lett.* **2003**, *5*, 2571-2572.
(8) Sun, K.; Sachwani, R.; Richert, K. J.; Driver, T. G. *Org. Lett.* **2009**, *11*, 3598-3601.
(9) Smith, P. A. S.; Rowe, C. D.; Hansen, D. W. *Tetrahedron Lett.* **1983**, *24*, 5169-5172.
(10) Katritzky, A. R.; Wang, Z. *J. Heterocycl. Chem.* **1988**, *25*, 671-675.
(11) Tan, Y.; Hartwig, J. F. *J. Am. Chem. Soc.* **2010**, *132*, 3676-3677.
(12) Chiba, S.; Hattori, G.; Narasaka, K. *Chem. Lett.* **2007**, *36*, 52-53.
(13) Leogane, O.; Lebel, H. *Angew. Chem., Int. Ed.* **2008**, *47*, 350-352.
(14) Banwell, M. G.; Kelly, B. D.; Kokas, O. J.; Lupton, D. W. *Org. Lett.* **2003**, *5*, 2497-2500.
(15) Cacchi, S.; Fabrizi, G.; Lamba, D.; Marinelli, F.; Parisi, L. M. *Synthesis* **2003**, 728-734.
(16) Wang, X.; Gribkov, D. V.; Sames, D. *J. Org. Chem.* **2007**, *72*, 1476-1479.
(17) Cacchi, S.; Fabrizi, G.; Goggiamani, A.; Perboni, A.; Sferrazza, A.; Stabile, P. *Org. Lett.* **2010**, *12*, 3279-3281.

Contents

VII. Spectral Data for Substrate Preparation via Wittig Route ………………………………..*s*-46

VIII. Spectral Data for Substrate Preparation via Horner–Wadsworth–Emmons Route……..*s*-88

IX. Spectral Data for Substrate Preparation via Suzuki Route…………………………….*s*-100

X. Spectral Data for 2,3-Disubstituted Indoles……………………………………………*s*-134

XI. Spectral Data for Mechanistic Experiments……………………………………………*s*-178

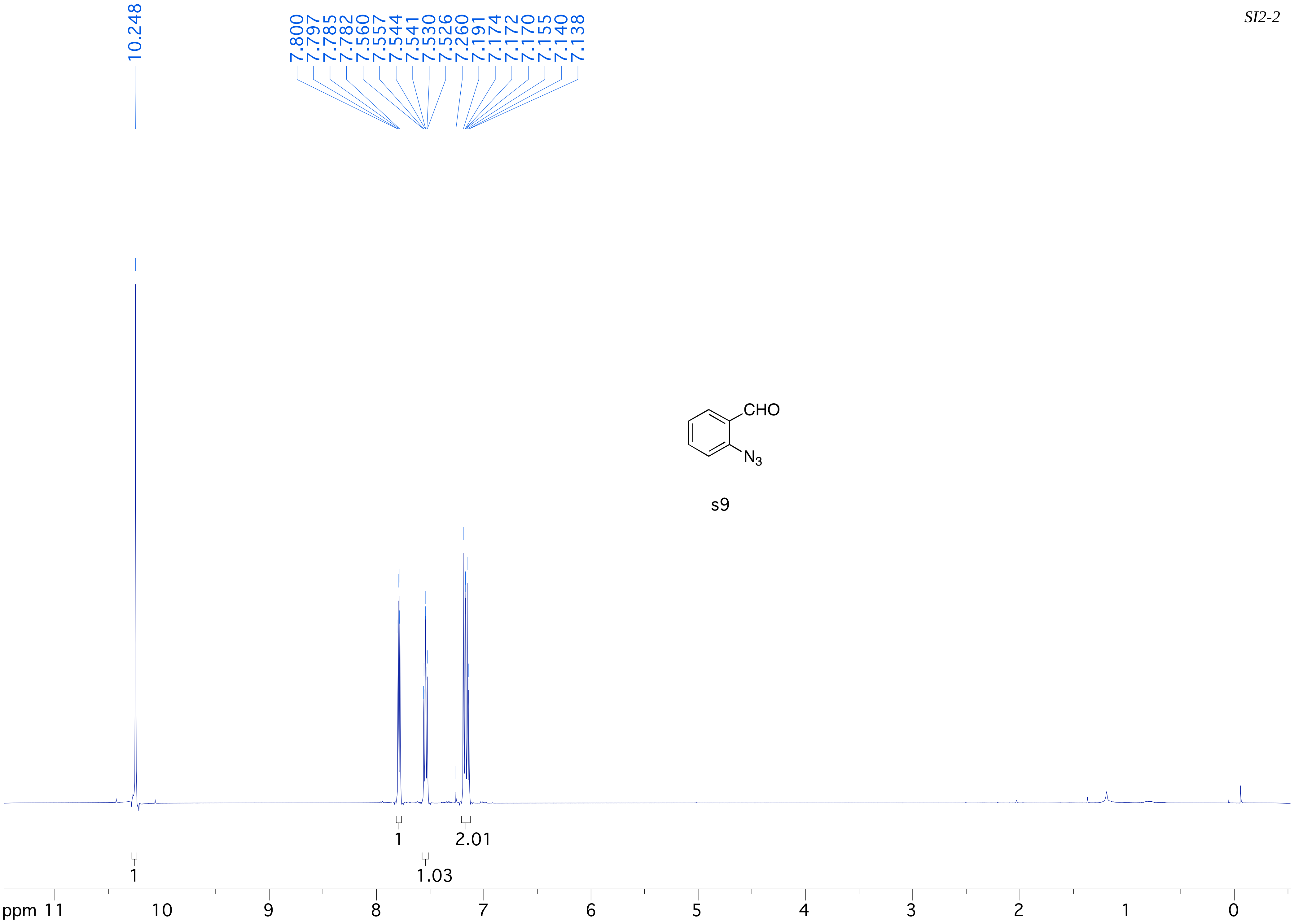
10.248
7.800
7.797
7.785
7.782
7.560
7.557
7.544
7.541
7.530
7.526
7.260
7.191
7.174
7.172
7.170
7.155
7.140
7.138
1
1.03
1
2.01
ppm 11
10
9
8
7
6
5
4
3
2
1
0
CHO
N_3

s9

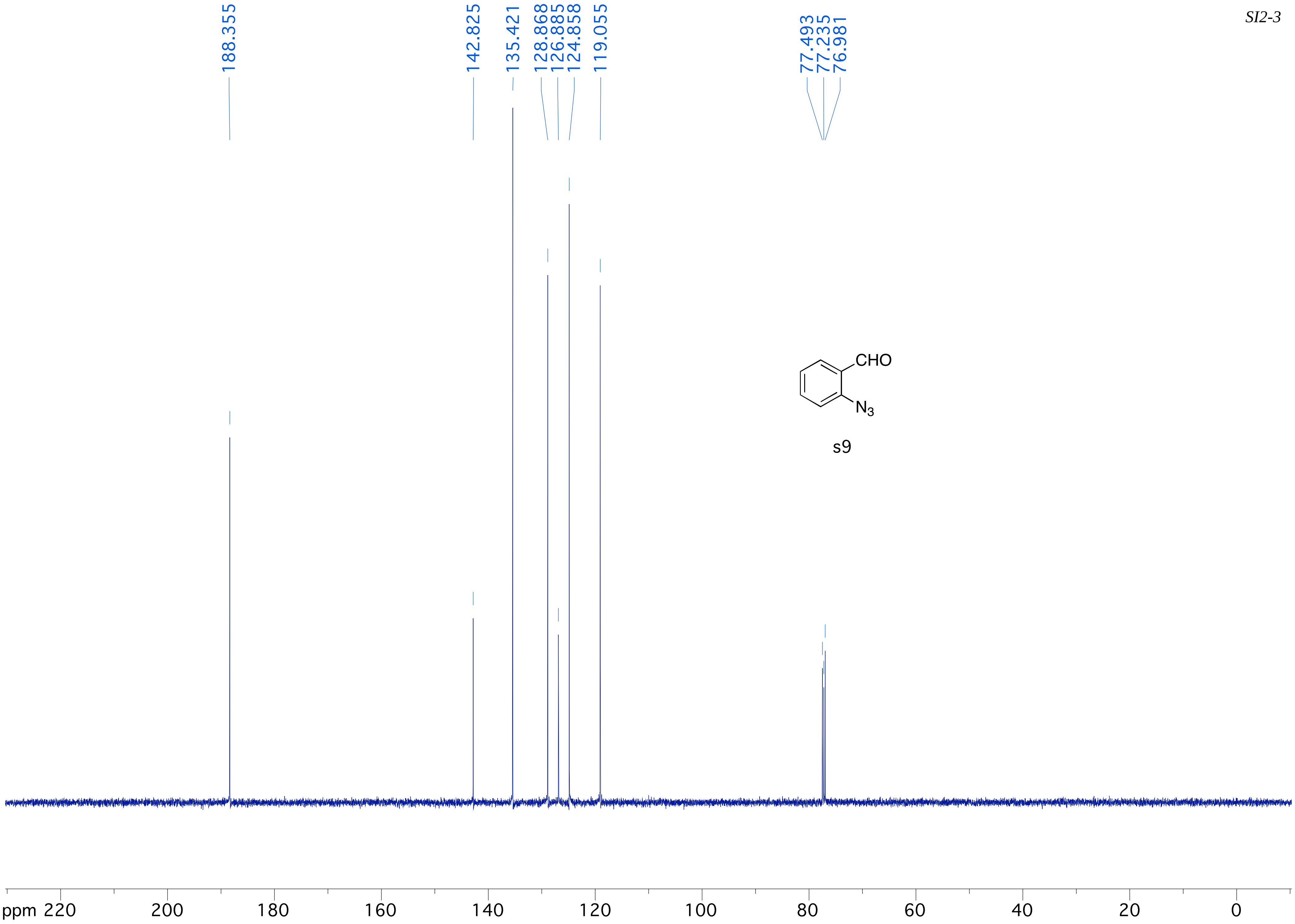
188.355
142.825
135.421
128.868
126.885
124.858
119.055
77.493
77.235
76.981
CHO
N3
s9
ppm 220
200
180
160
140
120
100
80
60
40
20
0

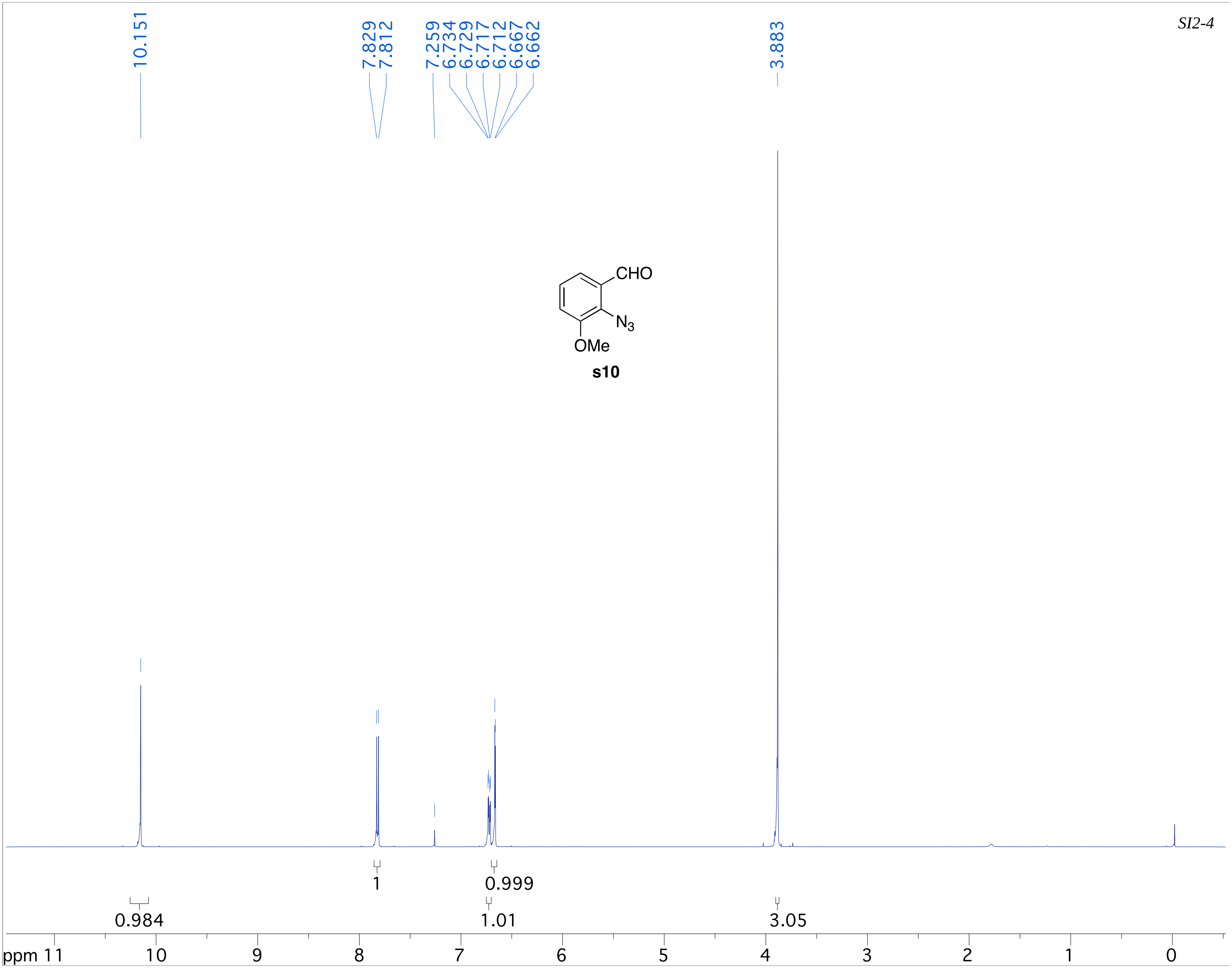
10.151
7.829
7.812
7.259
6.734
6.729
6.717
6.712
6.667
6.662
3.883
CHO
N3
OMe
s10
1
0.999
0.984
1.01
3.05
ppm 11
10
9
8
7
6
5
4
3
2
1
0

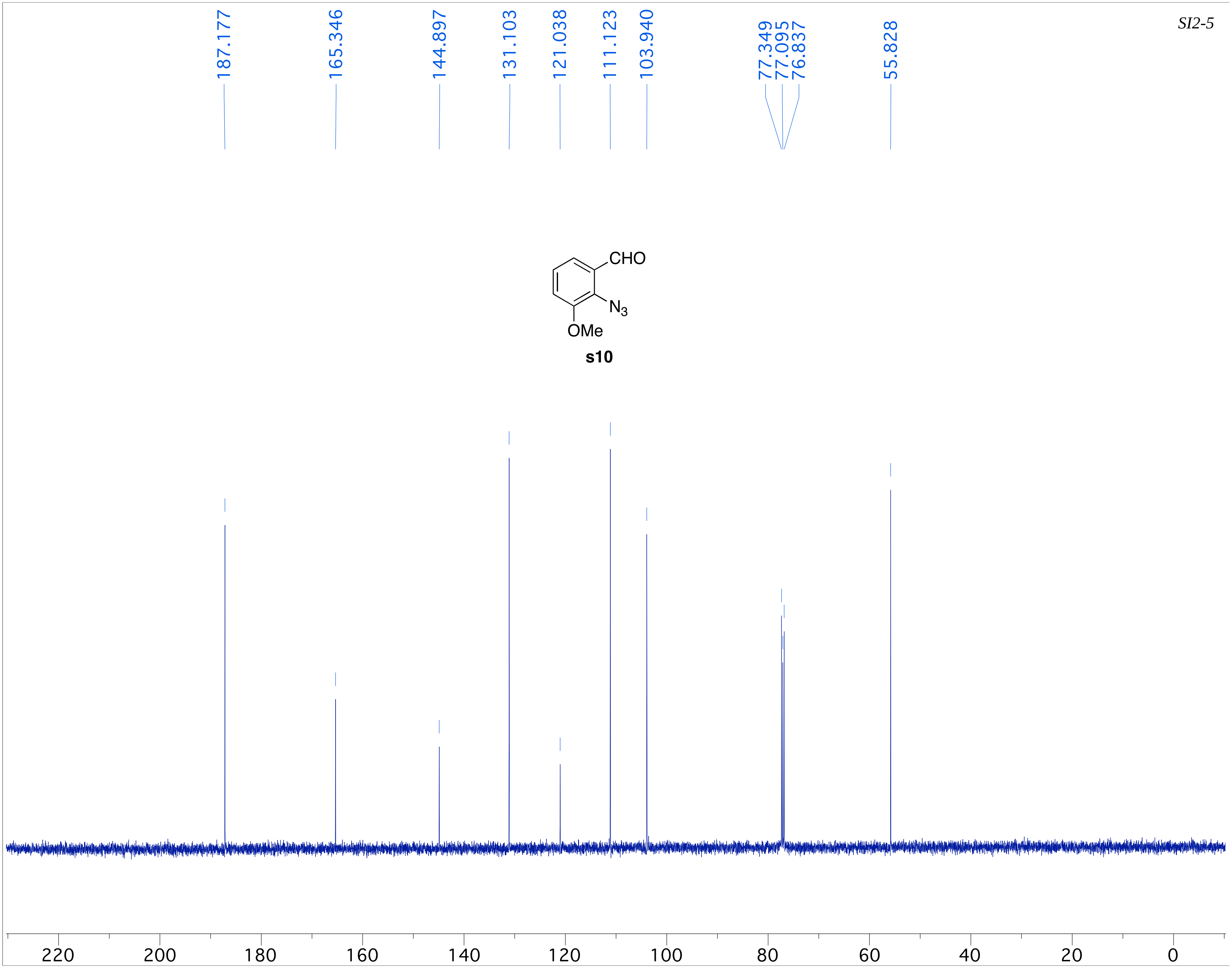
187.177
165.346
144.897
131.103
121.038
111.123
103.940
77.349
77.095
76.837
55.828
CHO
N3
OMe
s10
220
200
180
160
140
120
100
80
60
40
20
0

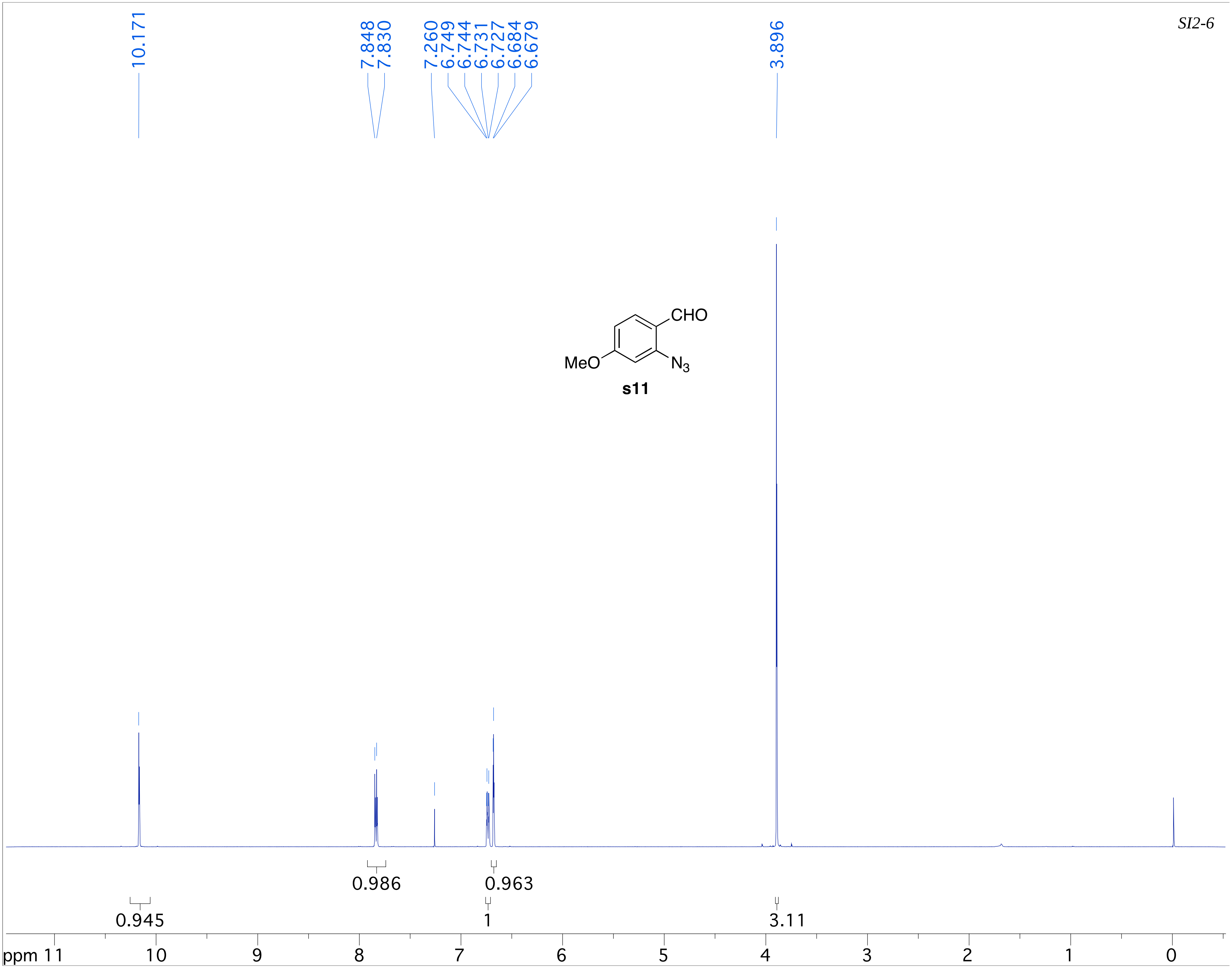
10.171
7.848
7.830
7.260
6.749
6.744
6.731
6.727
6.684
6.679
3.896
CHO
MeO
N3
s11
0.986
0.963
0.945
1
3.11
ppm 11
10
9
8
7
6
5
4
3
2
1
0

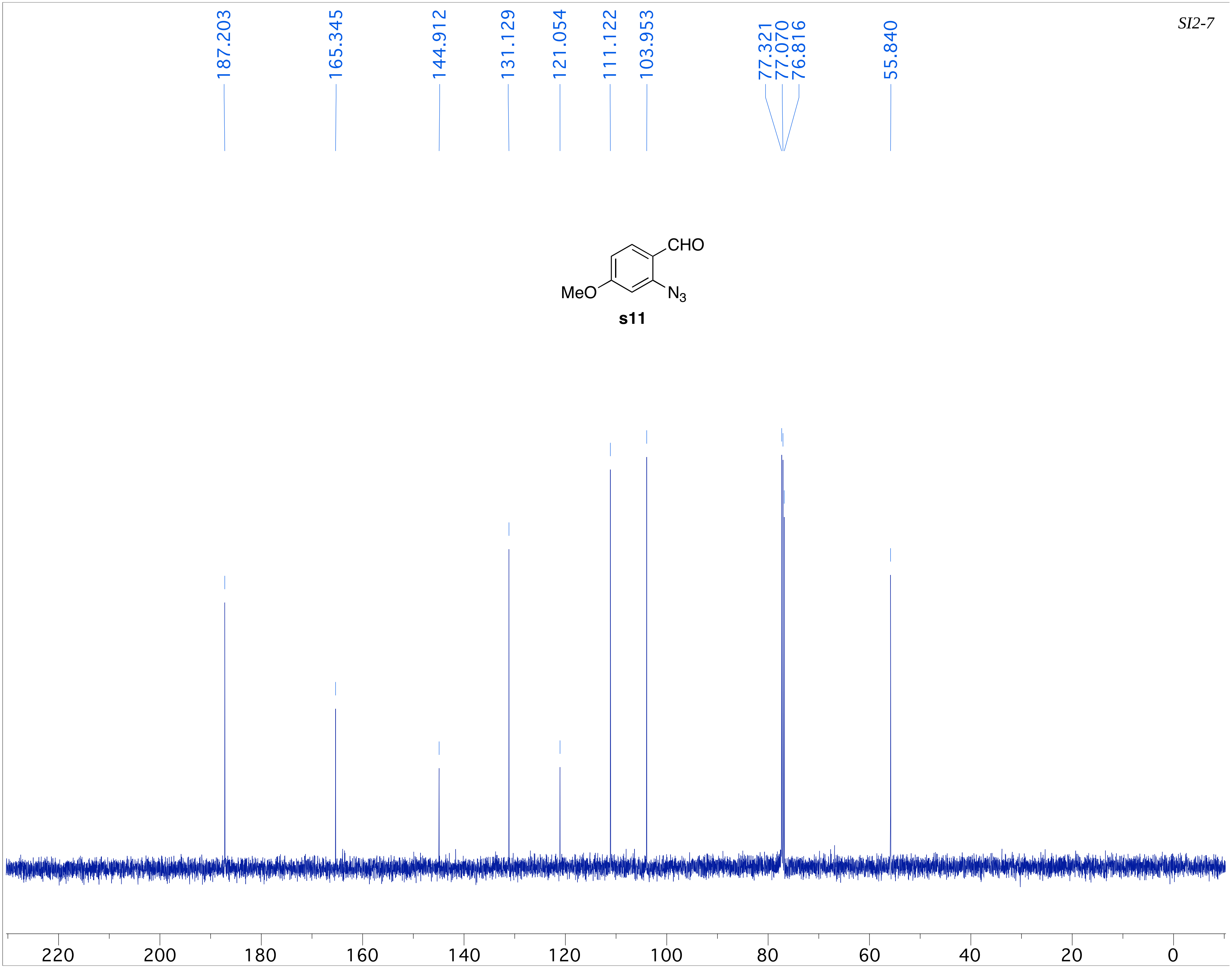
187.203
165.345
144.912
131.129
121.054
111.122
103.953
77.321
77.070
76.816
55.840
CHO
MeO
N3
s11
220
200
180
160
140
120
100
80
60
40
20
0

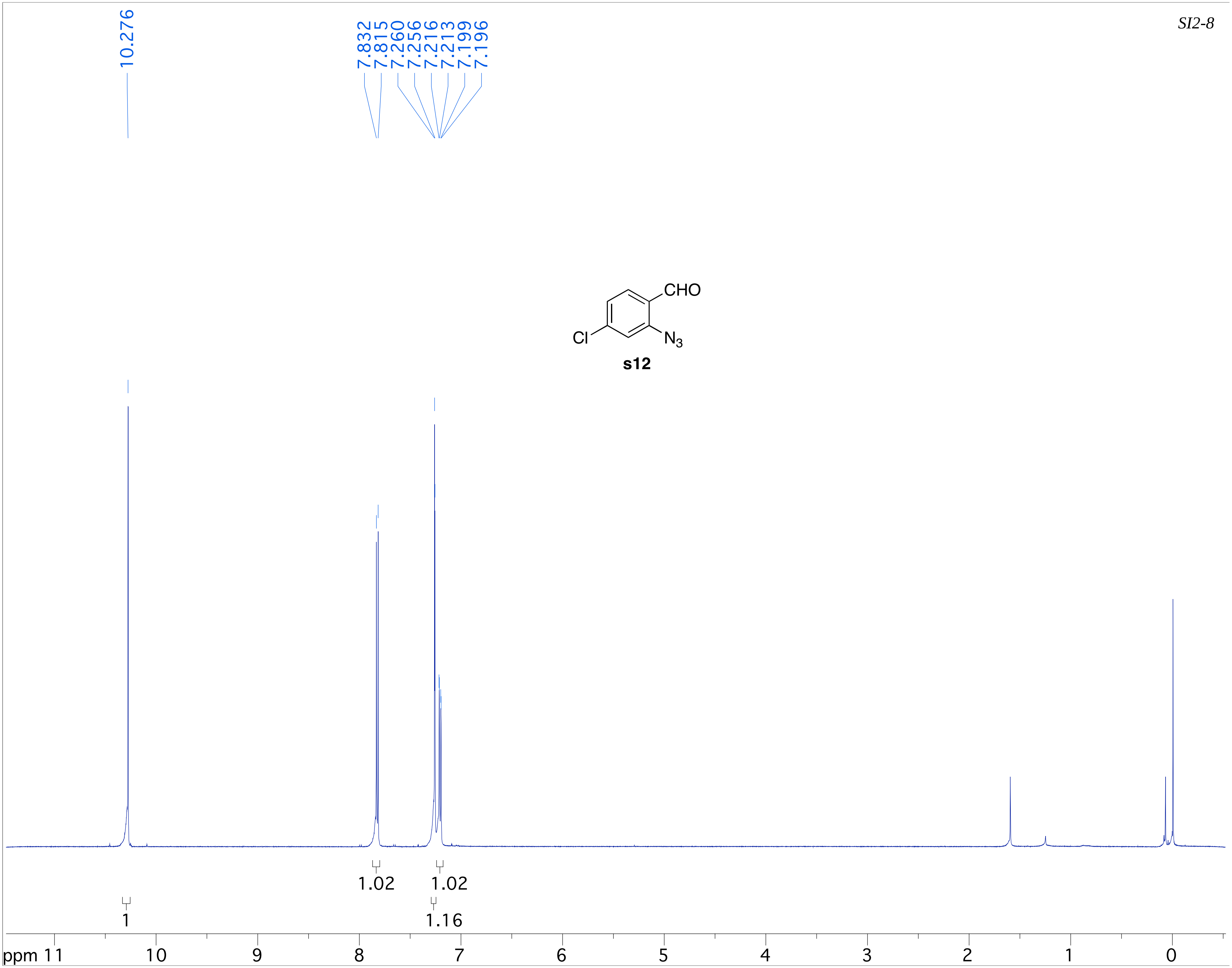
10.276
7.832
7.815
7.260
7.256
7.216
7.213
7.199
7.196
CHO
Cl
N3
s12
1.02
1.02
1
1.16
ppm 11
10
9
8
7
6
5
4
3
2
1
0

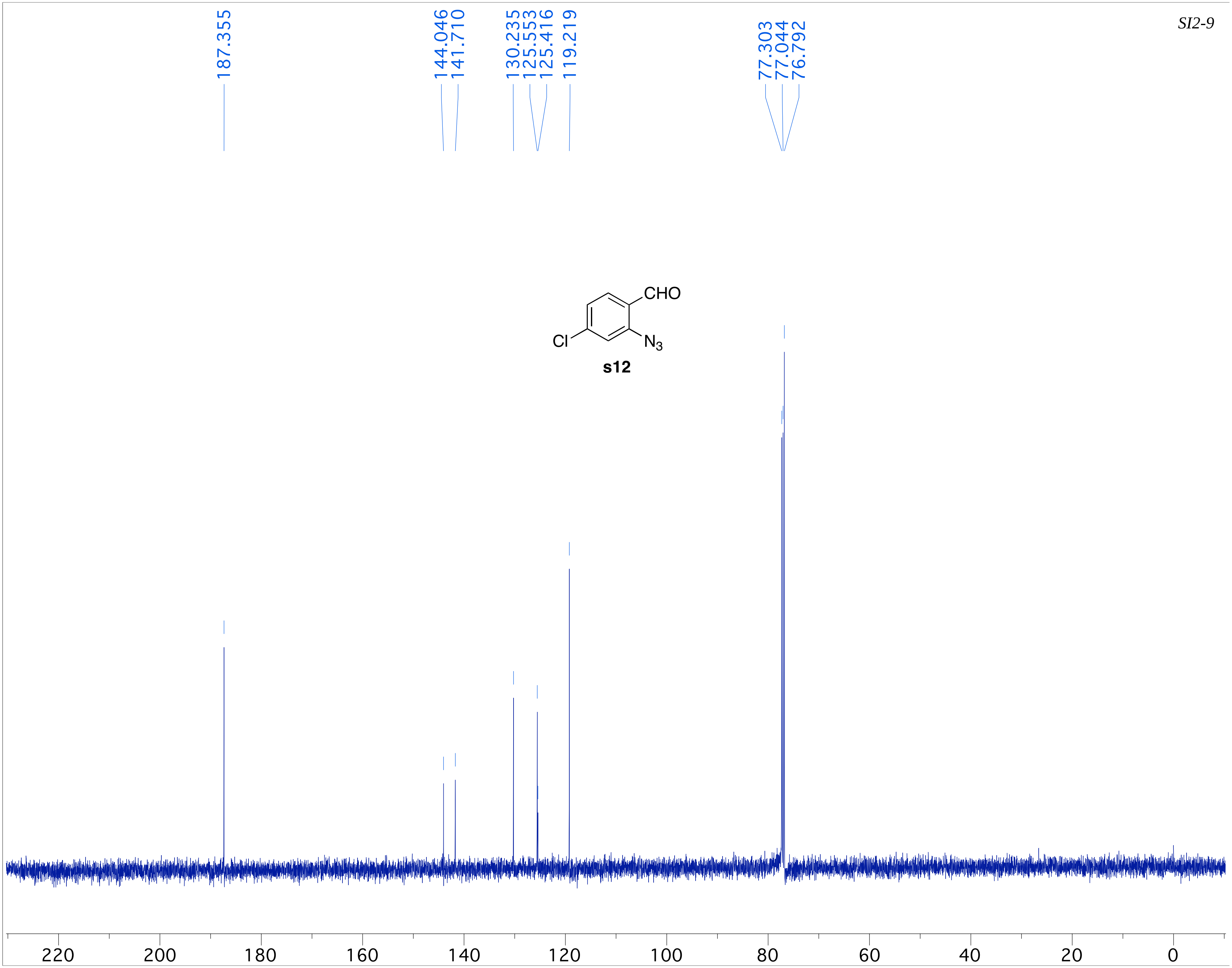
187.355
144.046
141.710
130.235
125.553
125.416
119.219
77.303
77.044
76.792
CHO
Cl
N3
s12
220
200
180
160
140
120
100
80
60
40
20
0

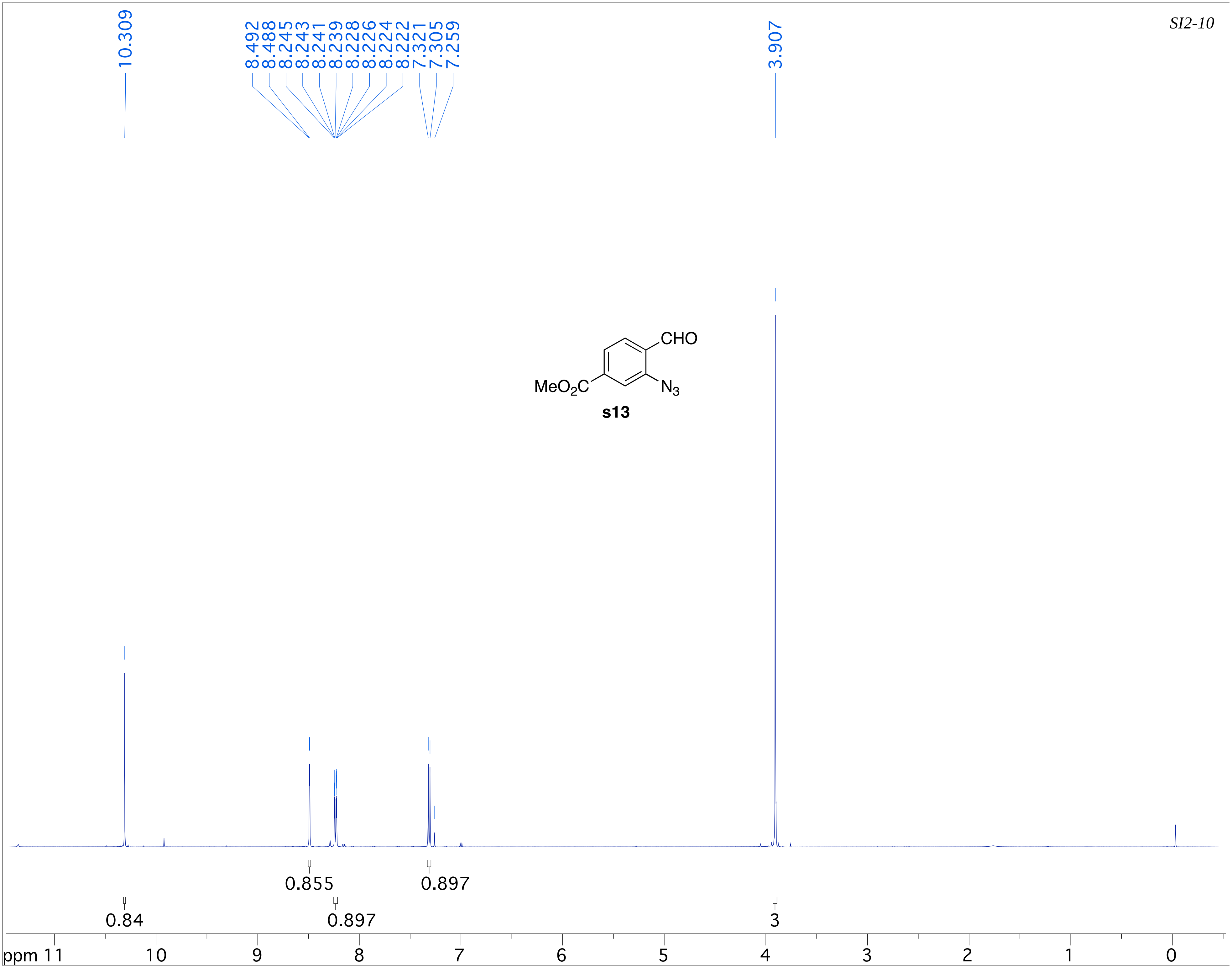
10.309
8.492
8.488
8.245
8.243
8.241
8.239
8.228
8.226
8.224
8.222
7.321
7.305
7.259
3.907
CHO
MeO_2C
N_3
s13
0.855
0.897
0.84
0.897
3
ppm 11 10 9 8 7 6 5 4 3 2 1 0

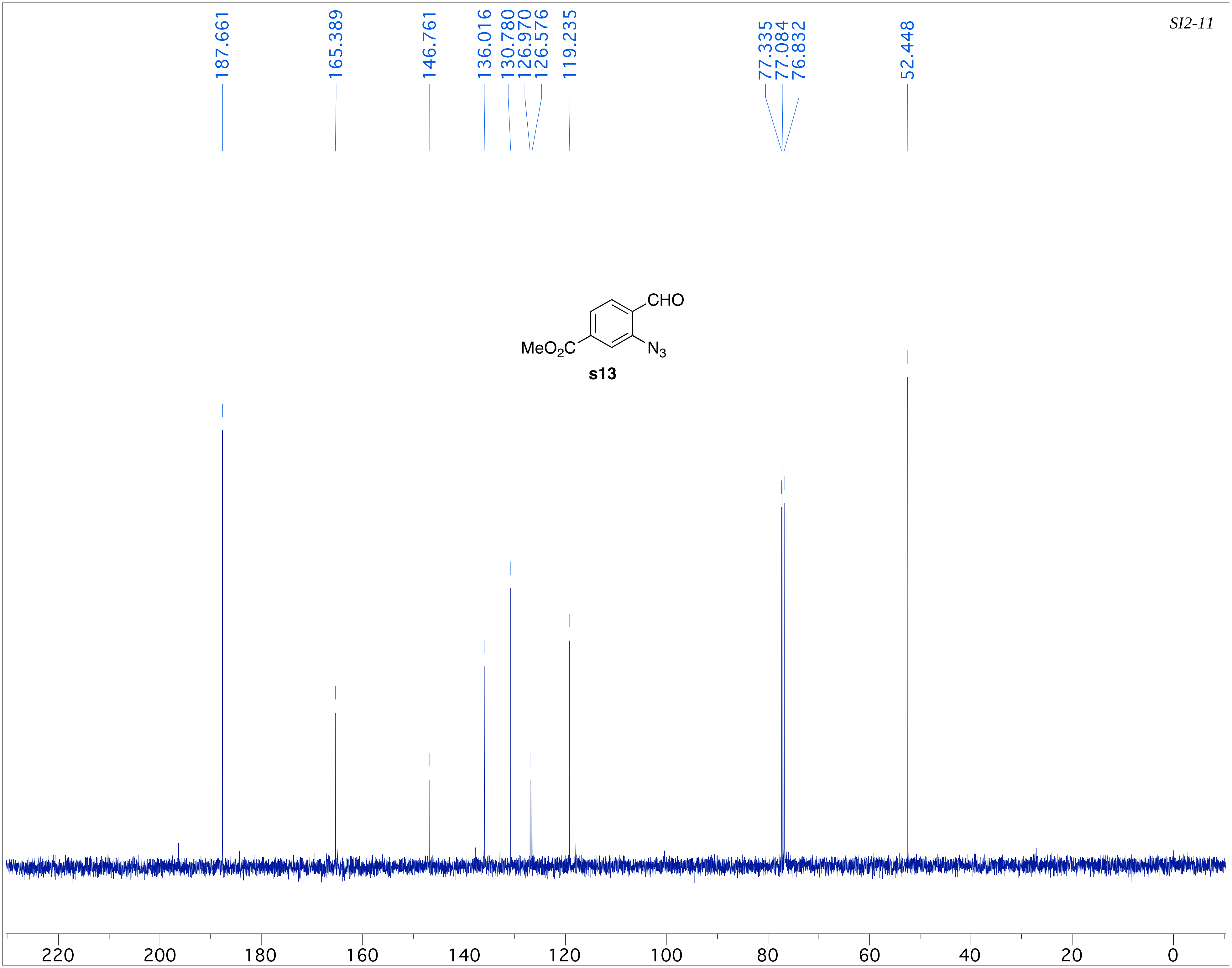
187.661
165.389
146.761
136.016
130.780
126.970
126.576
119.235
77.335
77.084
76.832
52.448
CHO
MeO2C
N3
220
200
180
160
140
120
100
80
60
40
20
0

s13

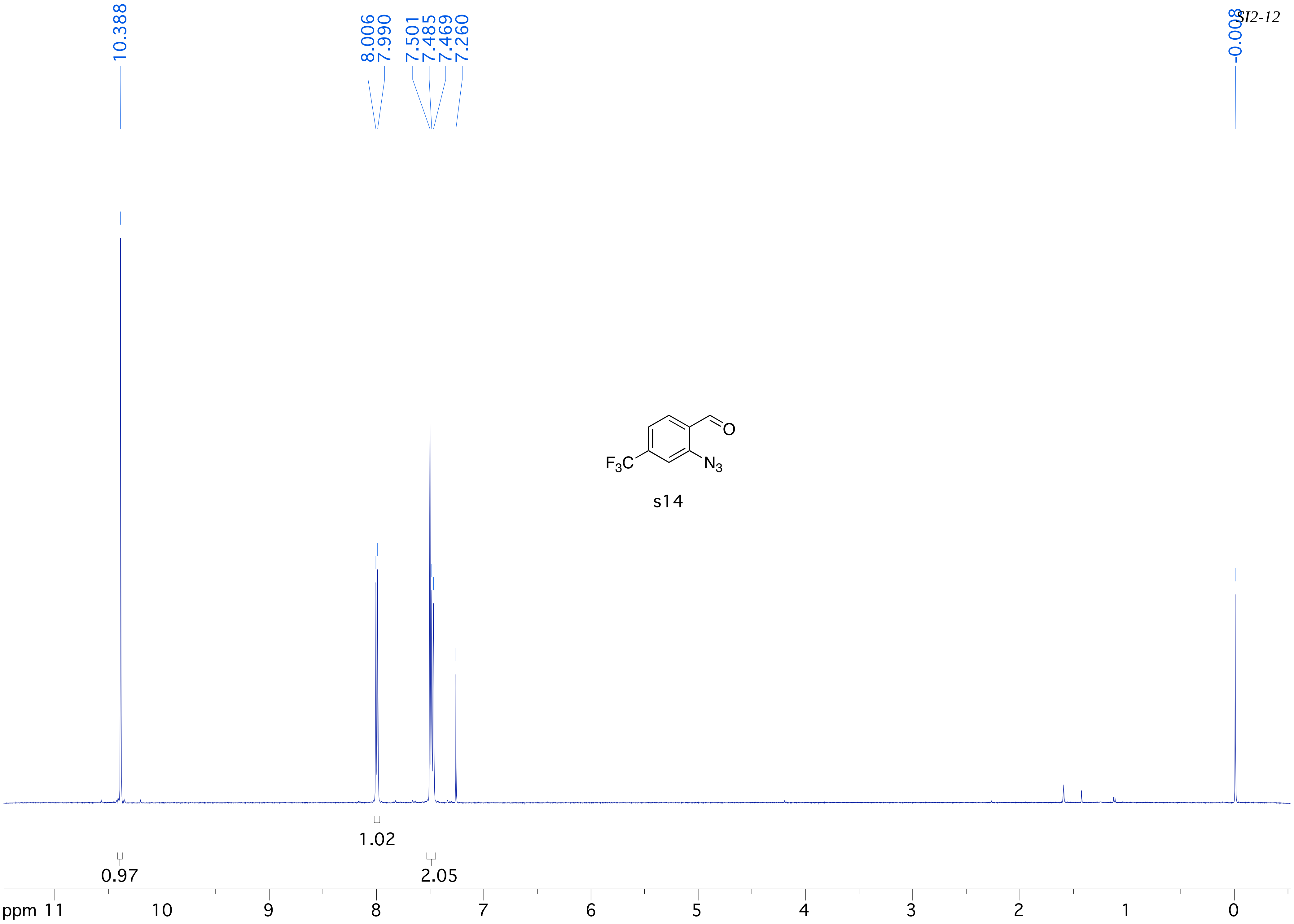
10.388
8.006
7.990
7.501
7.485
7.469
7.260
-0.008
F3C
N3
O
s14
1.02
0.97
2.05
ppm 11
10
9
8
7
6
5
4
3
2
1
0

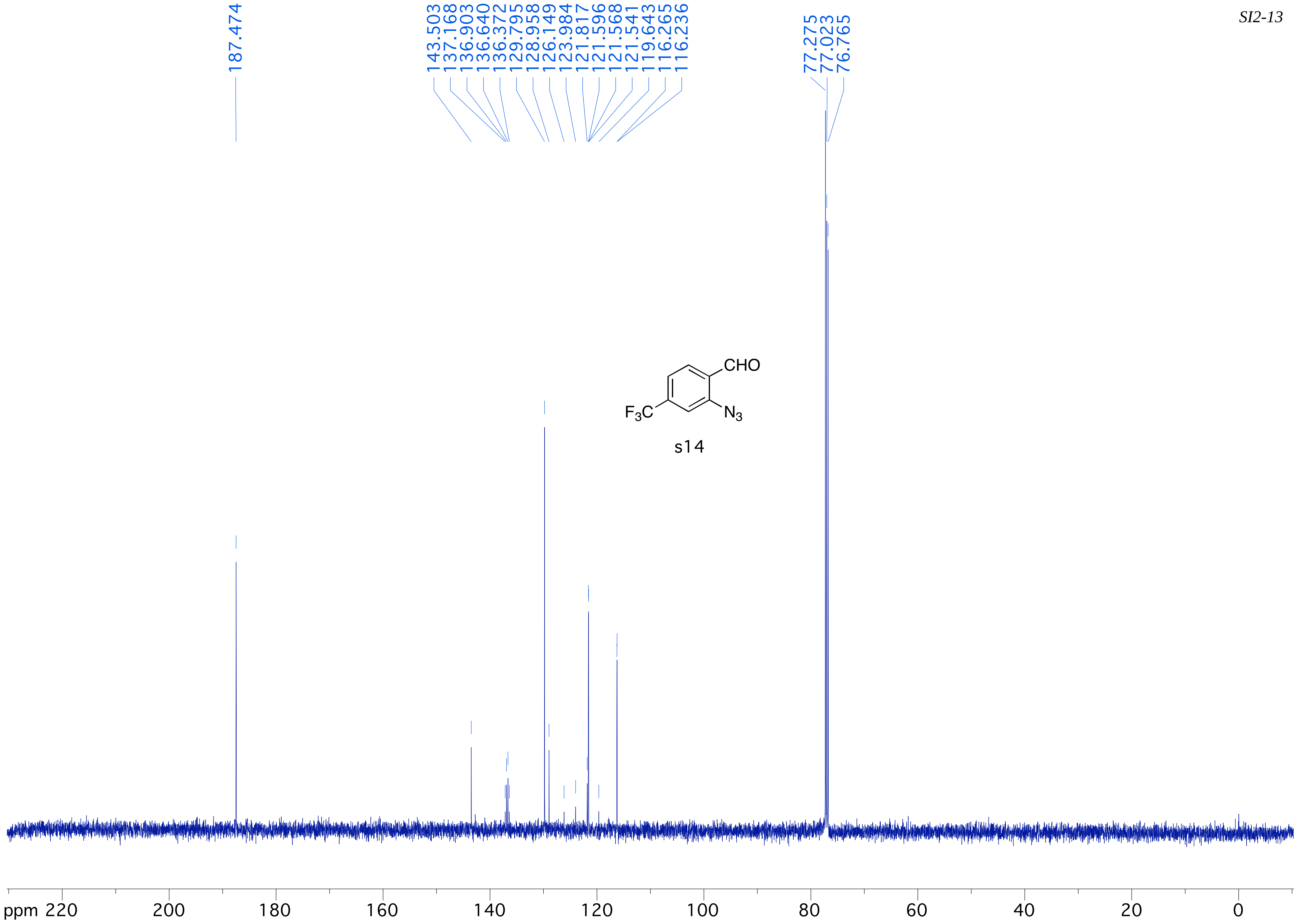
187.474
143.503
137.168
136.903
136.640
136.372
129.795
128.958
126.149
123.984
121.817
121.596
121.568
121.541
119.643
116.265
116.236
77.275
77.023
76.765
CHO
F_3C
N_3
s14
ppm 220
200
180
160
140
120
100
80
60
40
20
0

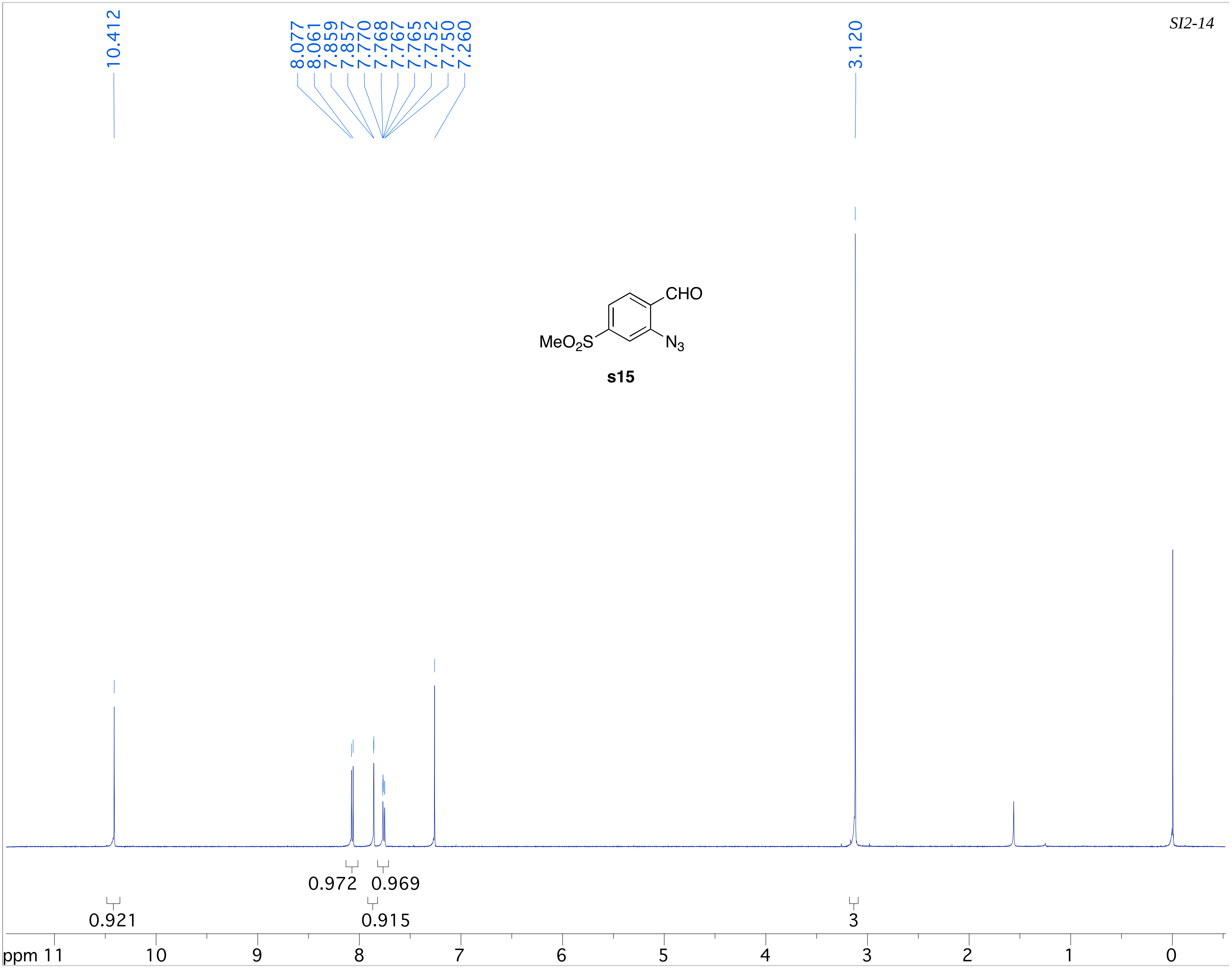
10.412
8.077
8.061
7.859
7.857
7.770
7.768
7.767
7.765
7.752
7.750
7.260
3.120
CHO
MeO2S
N3
s15
0.972
0.969
0.921
0.915
3
ppm 11
10
9
8
7
6
5
4
3
2
1
0

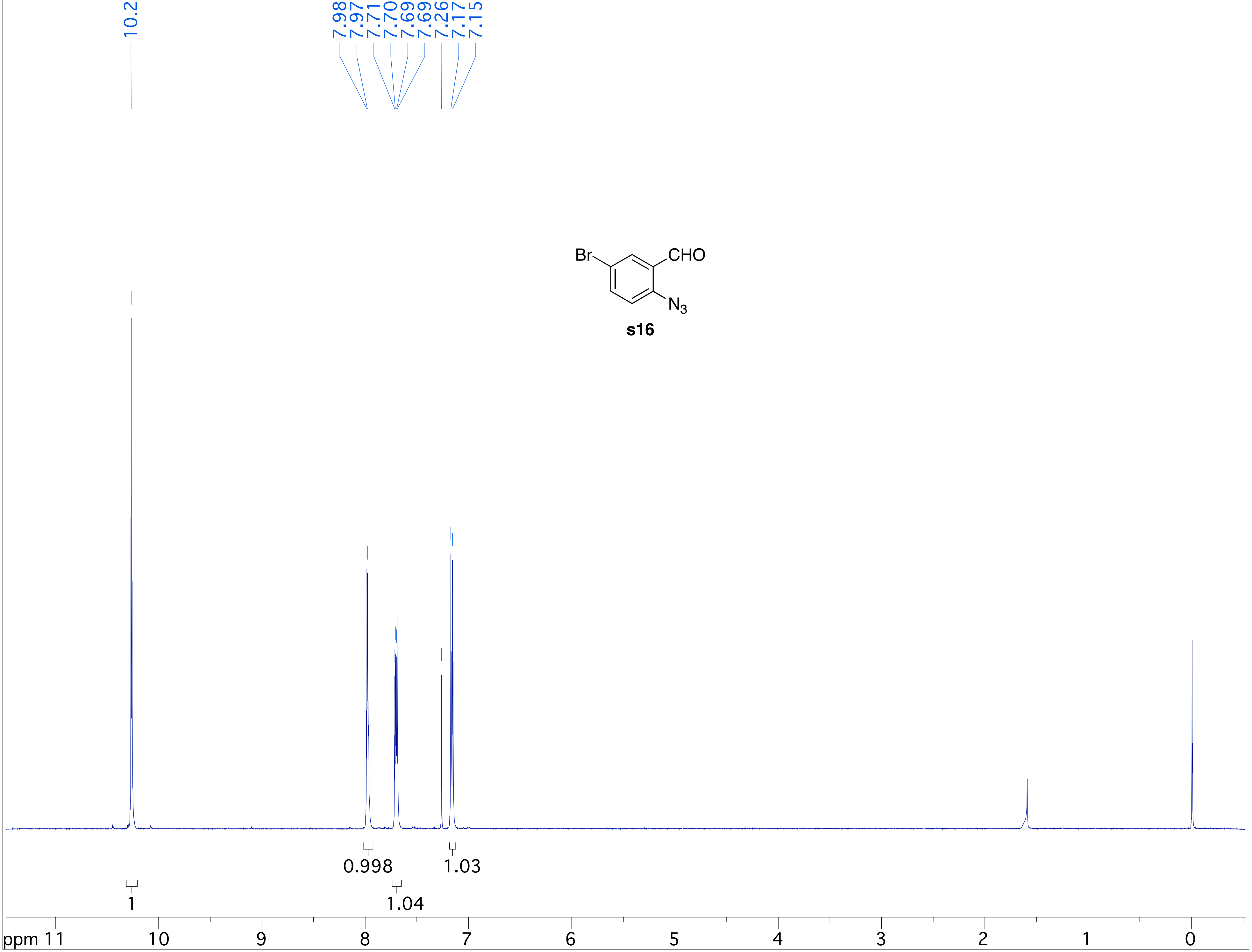
10.265
7.983
7.978
7.713
7.708
7.696
7.691
7.260
7.171
7.154
Br
CHO
N3
s16
0.998
1.03
1
1.04
ppm 11
10
9
8
7
6
5
4
3
2
1
0

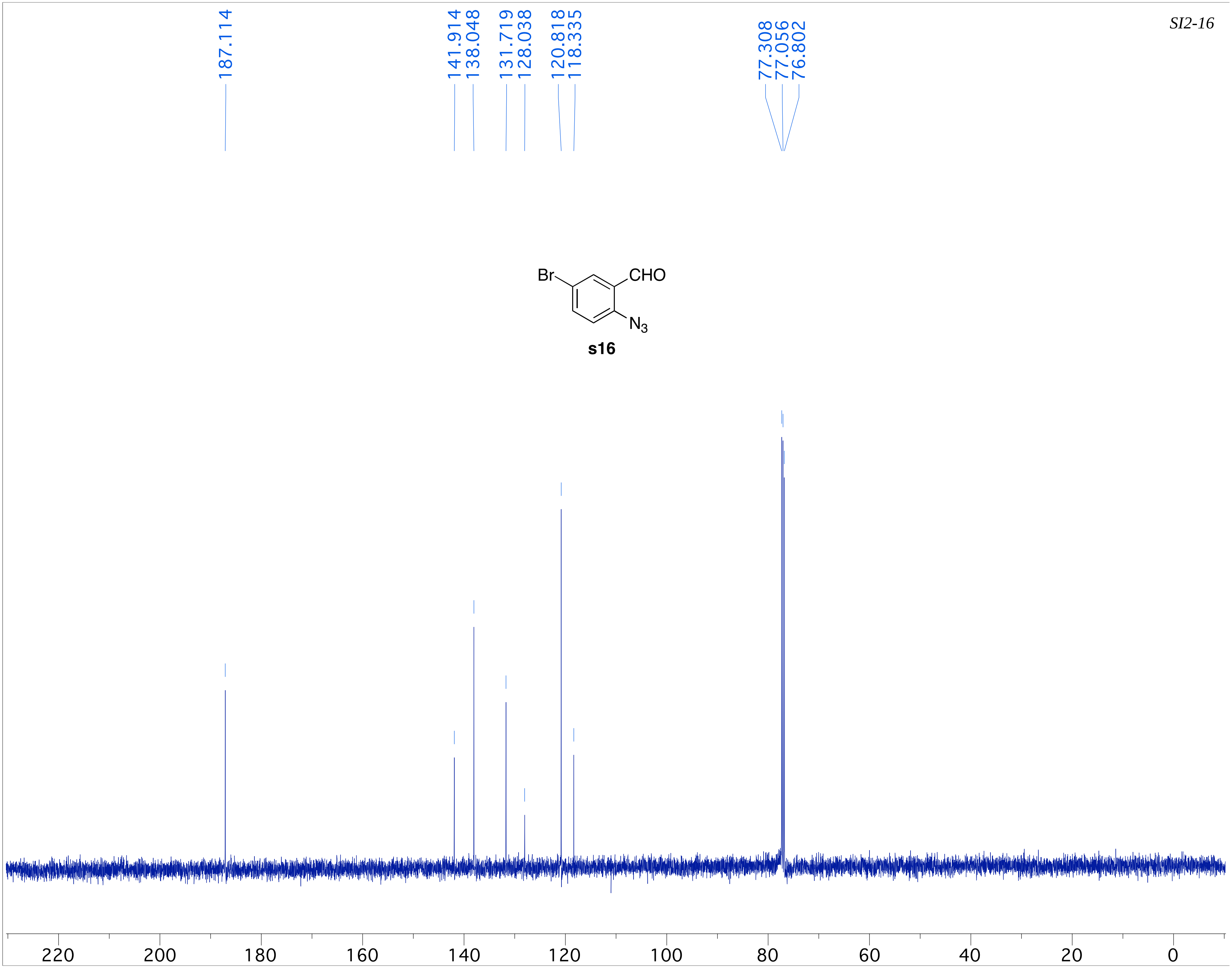

SI2-16
187.114
141.914
138.048
131.719
128.038
120.818
118.335
77.308
77.056
76.802
Br
CHO
N3
s16
220
200
180
160
140
120
100
80
60
40
20
0

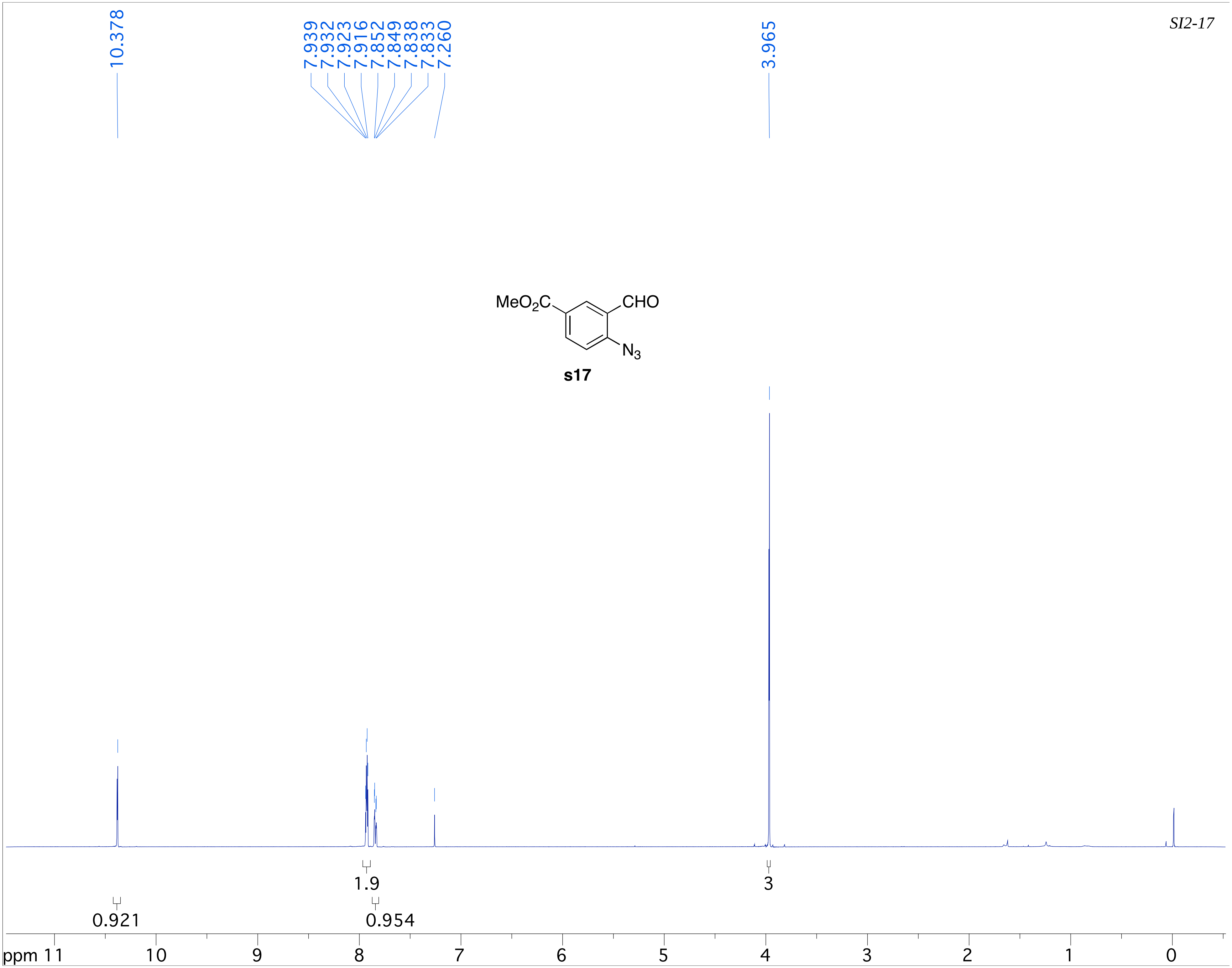
10.378
7.939
7.932
7.923
7.916
7.852
7.849
7.838
7.833
7.260
3.965
MeO2C
CHO
N3
s17
1.9
3
0.921
0.954
ppm 11
10
9
8
7
6
5
4
3
2
1
0

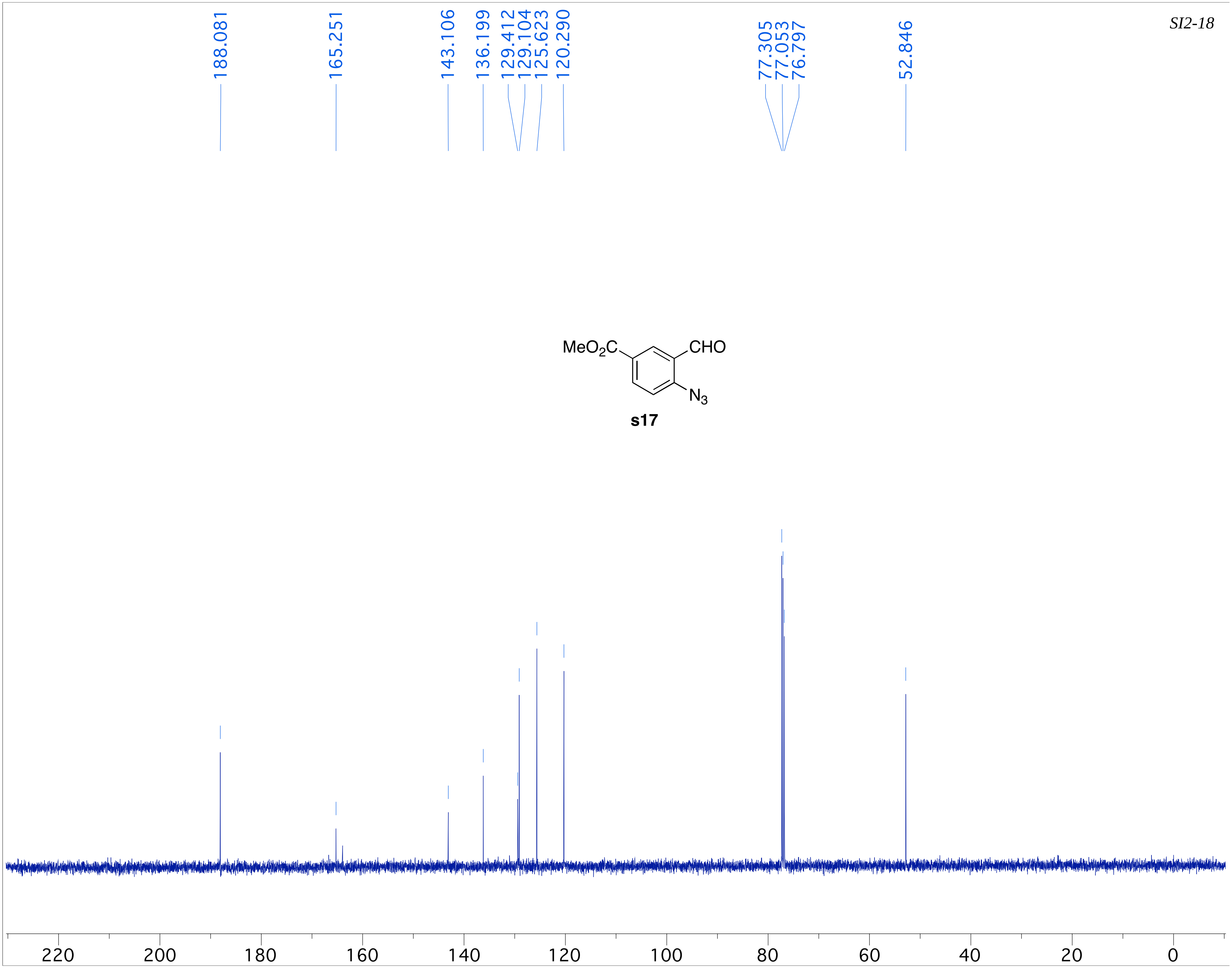
188.081
165.251
143.106
136.199
129.412
129.104
125.623
120.290
77.305
77.053
76.797
52.846
MeO2C
CHO
N3
s17
220
200
180
160
140
120
100
80
60
40
20
0

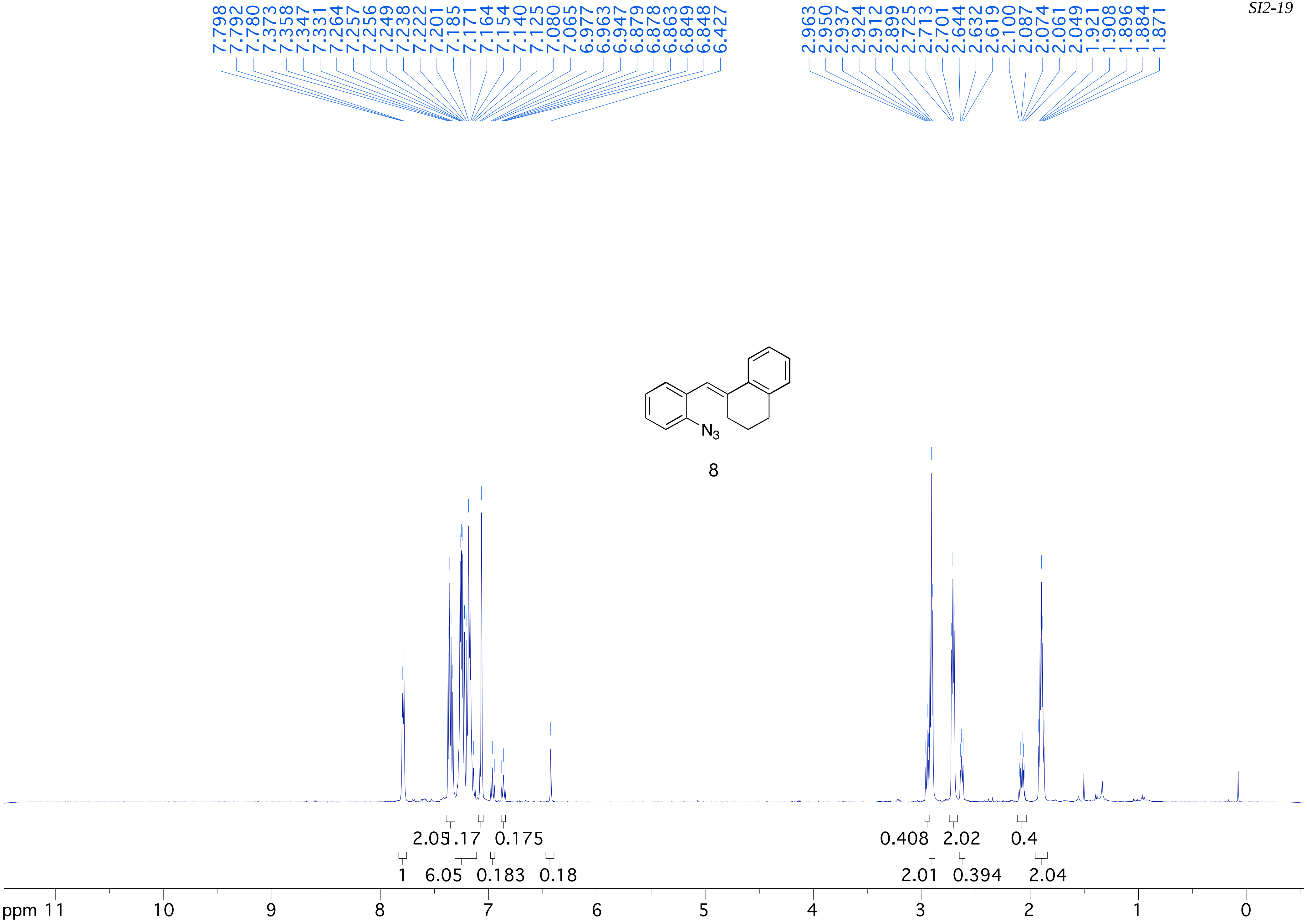
7.798
7.792
7.780
7.373
7.358
7.347
7.331
7.264
7.257
7.256
7.249
7.238
7.222
7.201
7.185
7.171
7.164
7.154
7.140
7.125
7.080
7.065
6.977
6.963
6.947
6.879
6.878
6.863
6.849
6.848
6.427
2.963
2.950
2.937
2.924
2.912
2.899
2.725
2.713
2.701
2.644
2.632
2.619
2.100
2.087
2.074
2.061
2.049
1.921
1.908
1.896
1.884
1.871
N3
8
2.05
1.17
0.175
0.408
2.02
0.4
1
6.05
0.183
0.18
2.01
0.394
2.04
ppm 11
10
9
8
7
6
5
4
3
2
1
0

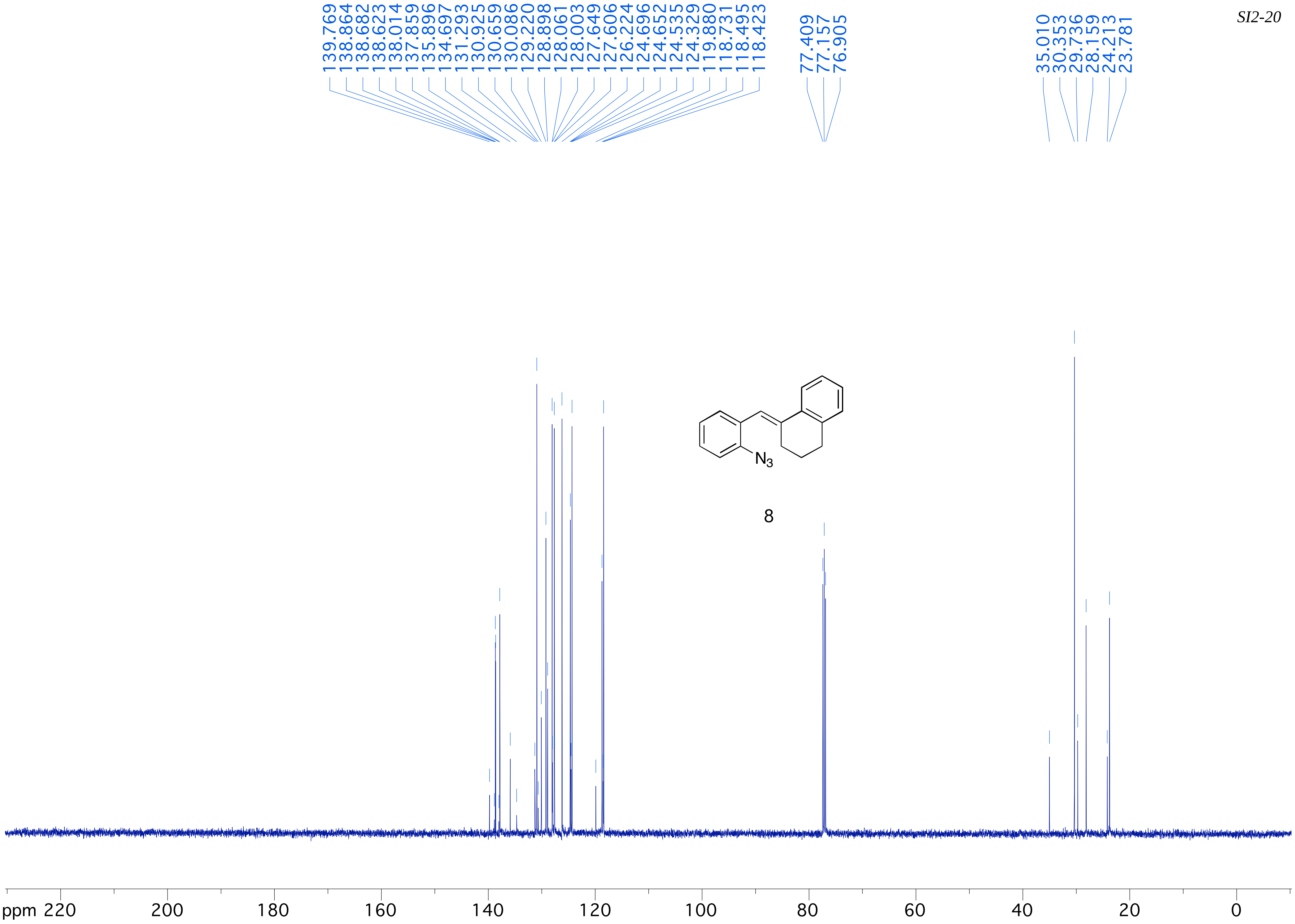
139.769
138.864
138.682
138.623
138.014
137.859
135.896
134.697
131.293
130.925
130.659
130.086
129.220
128.898
128.061
128.003
127.649
127.606
126.224
124.696
124.652
124.535
124.329
119.880
118.731
118.495
118.423
77.409
77.157
76.905
35.010
30.353
29.736
28.159
24.213
23.781
ppm 220
200
180
160
140
120
100
80
60
40
20
0
N3

8

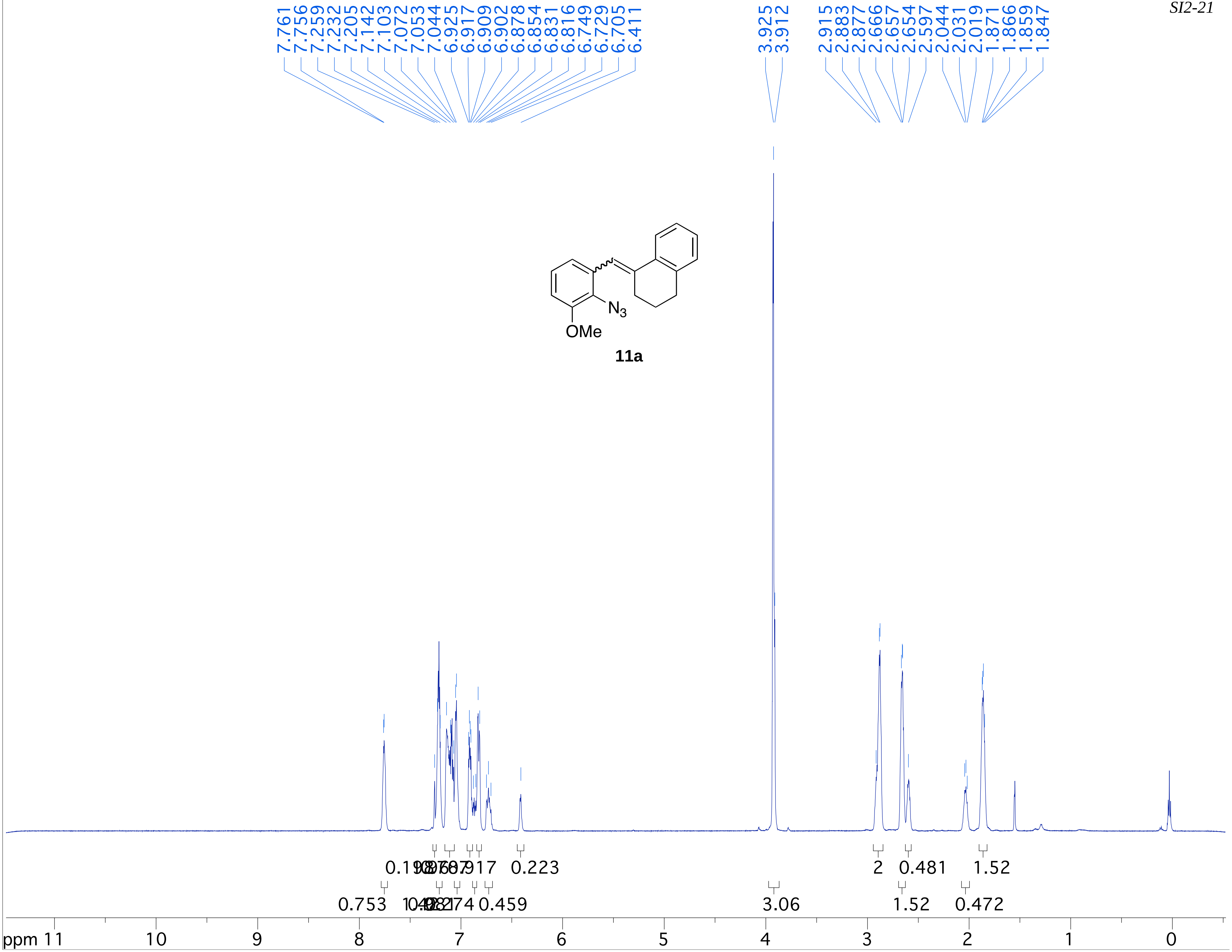
7.761
7.756
7.259
7.232
7.205
7.142
7.103
7.072
7.053
7.044
6.925
6.917
6.909
6.902
6.878
6.854
6.831
6.816
6.749
6.729
6.705
6.411
3.925
3.912
2.915
2.883
2.877
2.666
2.657
2.654
2.597
2.044
2.031
2.019
1.871
1.866
1.859
1.847
N3
OMe
11a
0.223
2
0.481
1.52
0.753
0.459
3.06
1.52
0.472
ppm 11
10
9
8
7
6
5
4
3
2
1
0

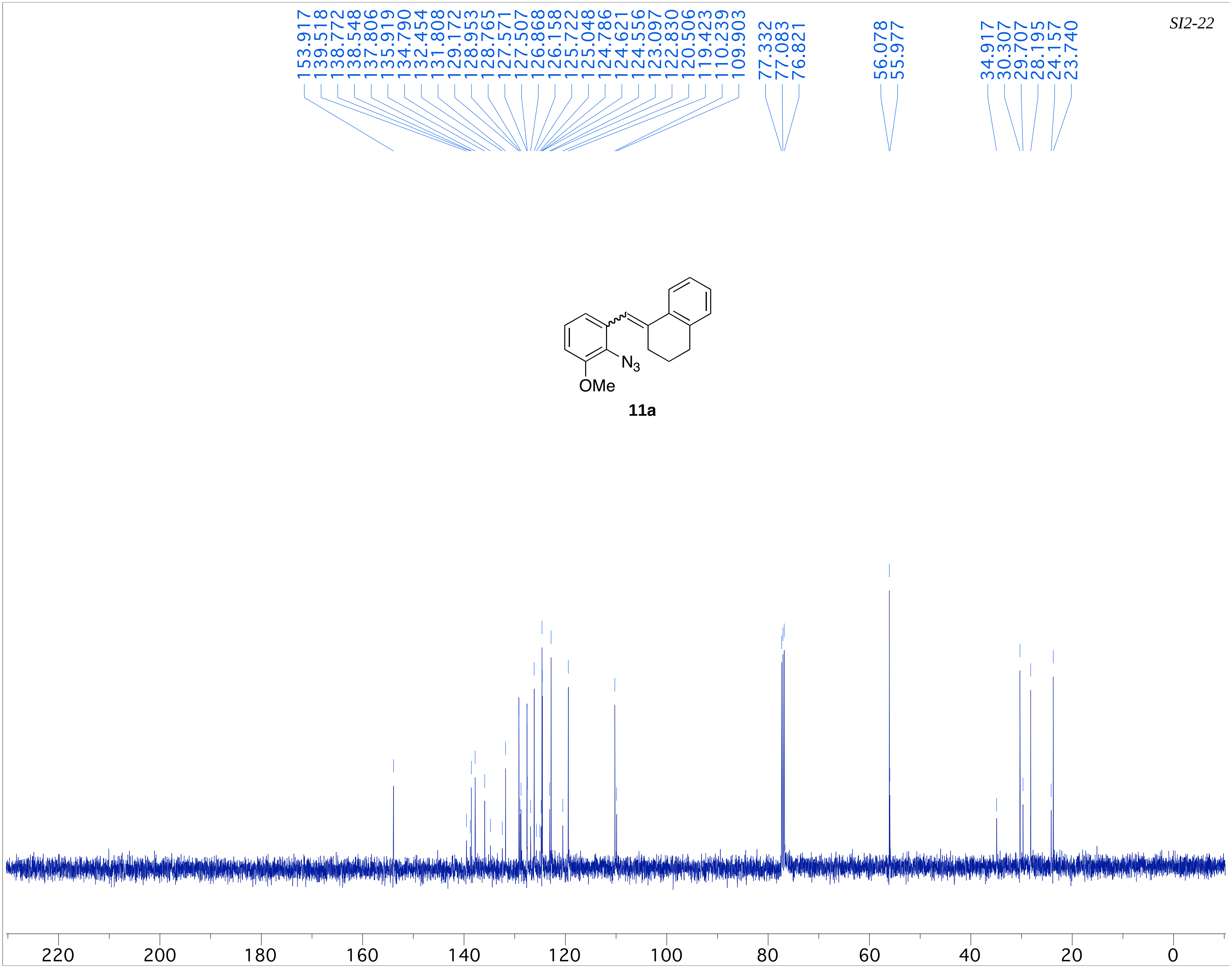
153.917
139.518
138.772
138.548
137.806
135.919
134.790
132.454
131.808
129.172
128.953
128.765
127.571
127.507
126.868
126.158
125.722
125.048
124.786
124.621
124.556
123.097
122.830
120.506
119.423
110.239
109.903
77.332
77.083
76.821
56.078
55.977
34.917
30.307
29.707
28.195
24.157
23.740
N_3
OMe
11a
220
200
180
160
140
120
100
80
60
40
20
0

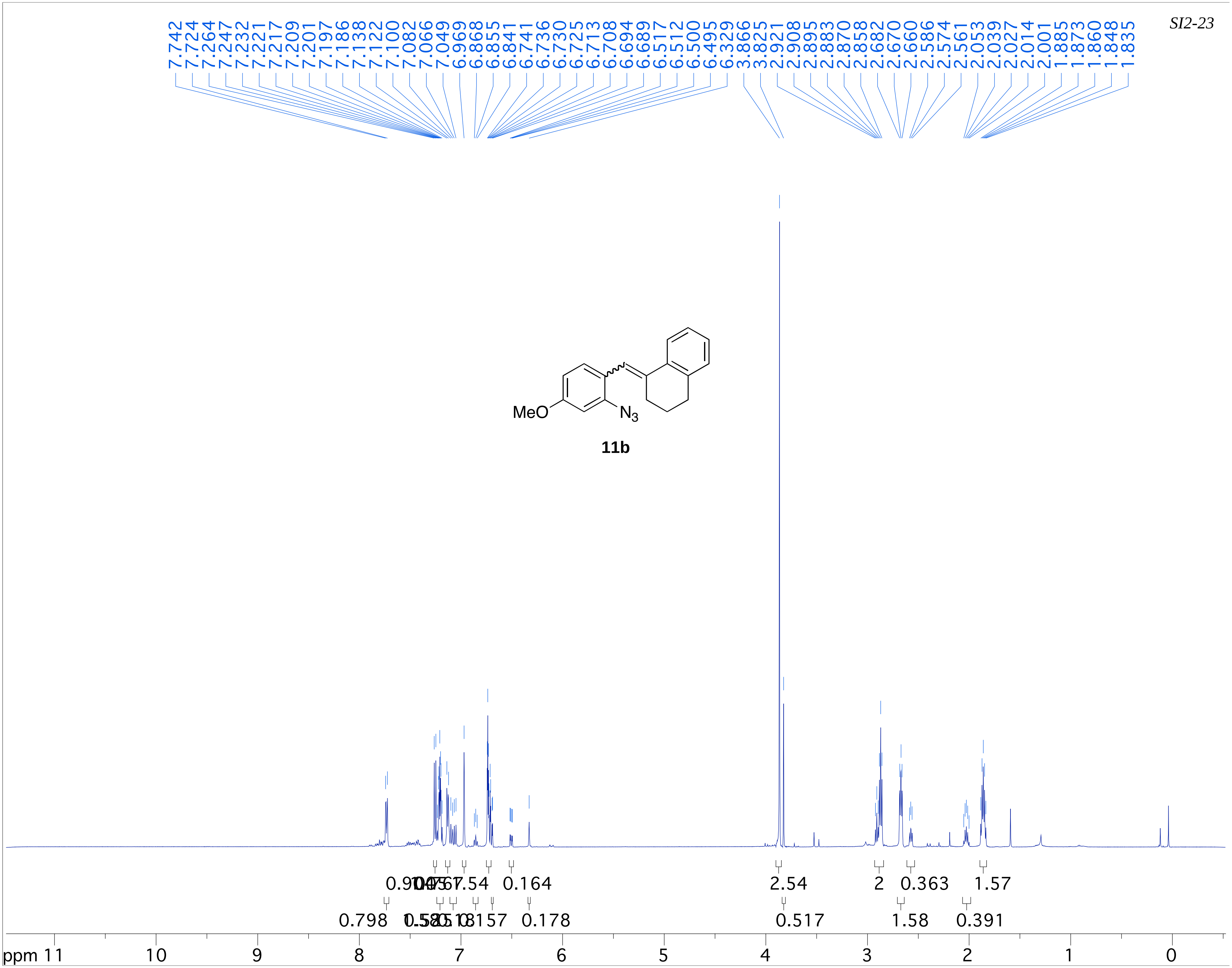
7.742
7.724
7.264
7.247
7.232
7.221
7.217
7.209
7.201
7.197
7.186
7.138
7.122
7.100
7.082
7.066
7.049
6.969
6.868
6.855
6.841
6.741
6.736
6.730
6.725
6.713
6.708
6.694
6.689
6.517
6.512
6.500
6.495
6.329
3.866
3.825
2.921
2.908
2.895
2.883
2.870
2.858
2.682
2.670
2.660
2.586
2.574
2.561
2.053
2.039
2.027
2.014
2.001
1.885
1.873
1.860
1.848
1.835
MeO
N3
11b
0.164
2.54
2
0.363
1.57
0.798
0.178
0.517
1.58
0.391
ppm 11
10
9
8
7
6
5
4
3
2
1
0

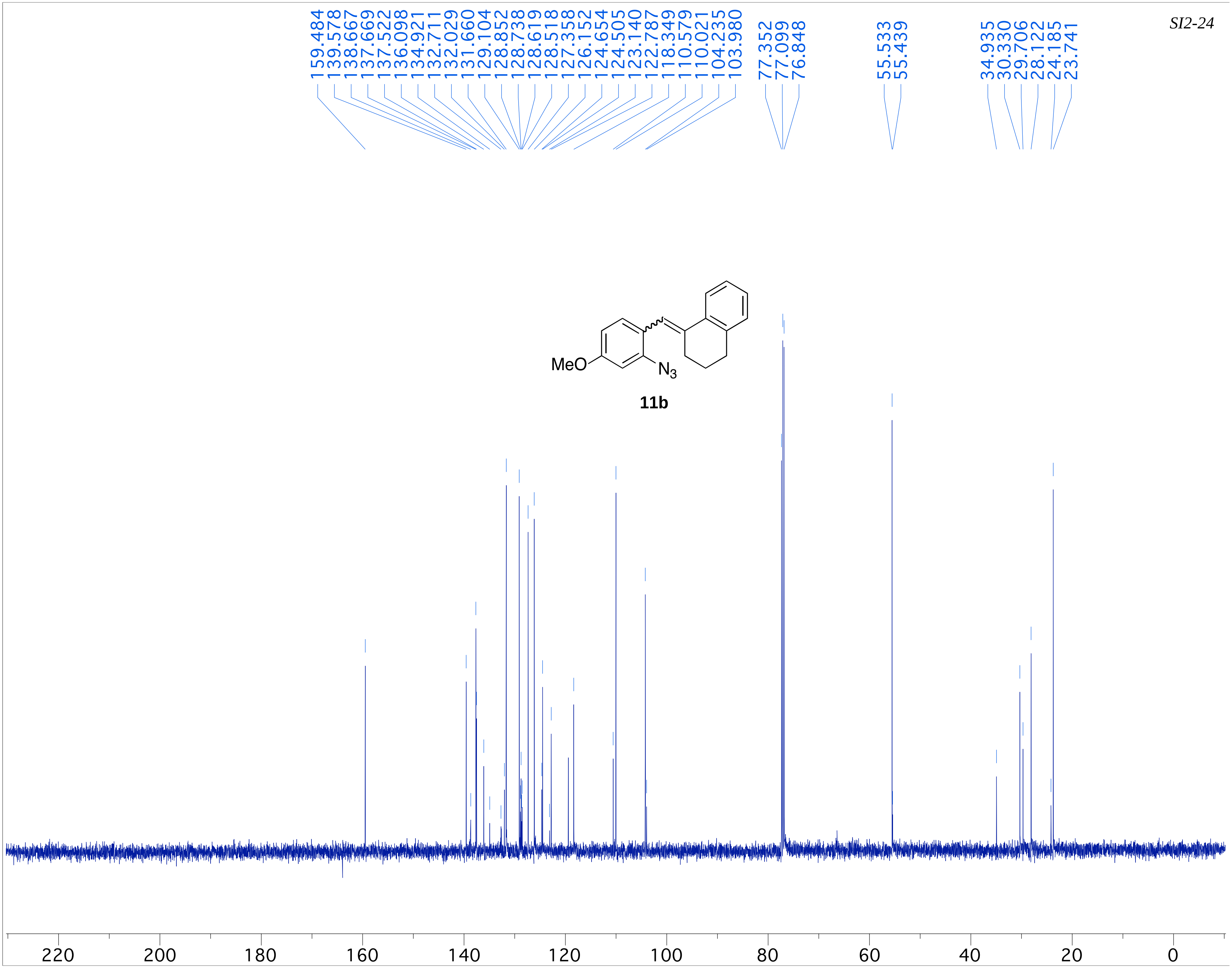
159.484
139.578
138.667
137.669
137.522
136.098
134.921
132.711
132.029
131.660
129.104
128.852
128.738
128.619
128.518
127.358
126.152
124.654
124.505
123.140
122.787
118.349
110.579
110.021
104.235
103.980
77.352
77.099
76.848
55.533
55.439
34.935
30.330
29.706
28.122
24.185
23.741
MeO
N_3
11b
220
200
180
160
140
120
100
80
60
40
20
0

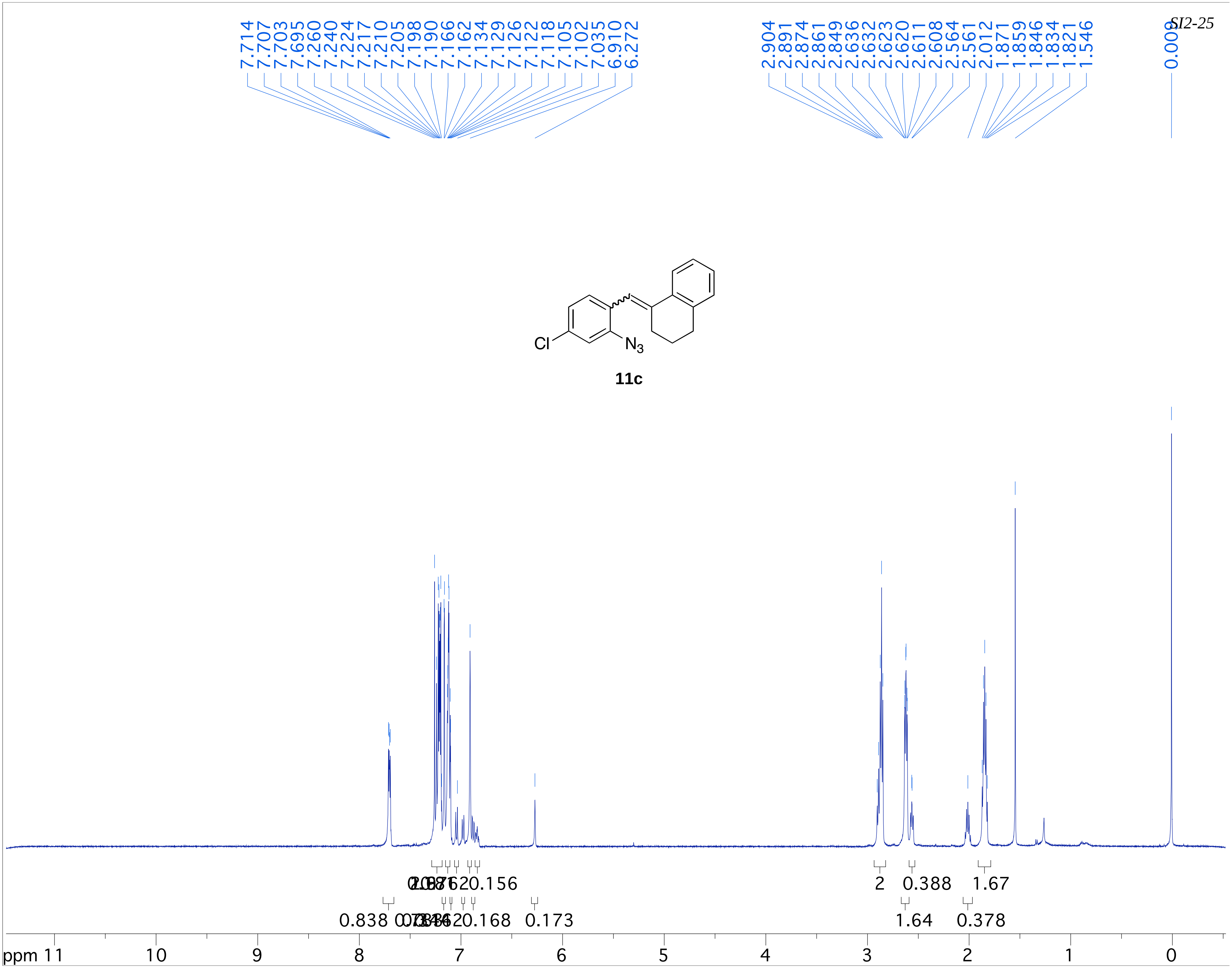
7.714
7.707
7.703
7.695
7.260
7.240
7.224
7.217
7.210
7.205
7.198
7.190
7.166
7.162
7.134
7.129
7.126
7.122
7.118
7.105
7.102
7.035
6.910
6.272
2.904
2.891
2.874
2.861
2.849
2.636
2.632
2.623
2.620
2.611
2.608
2.564
2.561
2.012
1.871
1.859
1.846
1.834
1.821
1.546
0.009
Cl
N3
11c
0.838
0.168
0.173
2
0.388
1.67
1.64
0.378
ppm 11
10
9
8
7
6
5
4
3
2
1
0

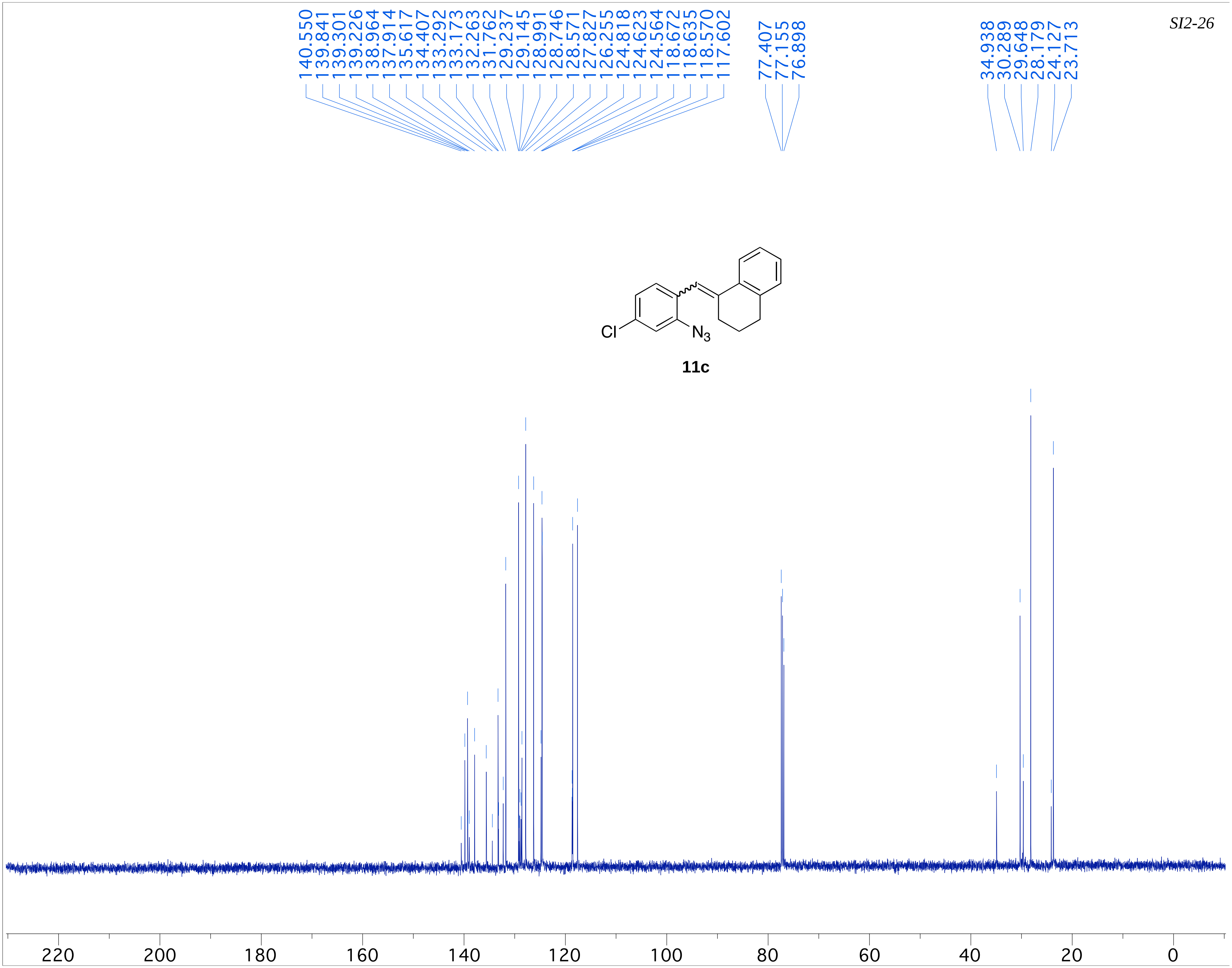
140.550
139.841
139.301
139.226
138.964
137.914
135.617
134.407
133.292
133.173
132.263
131.762
129.237
129.145
128.991
128.746
128.571
127.827
126.255
124.818
124.623
124.564
118.672
118.635
118.570
117.602
77.407
77.155
76.898
34.938
30.289
29.648
28.179
24.127
23.713
Cl
N_3
11c
220
200
180
160
140
120
100
80
60
40
20
0

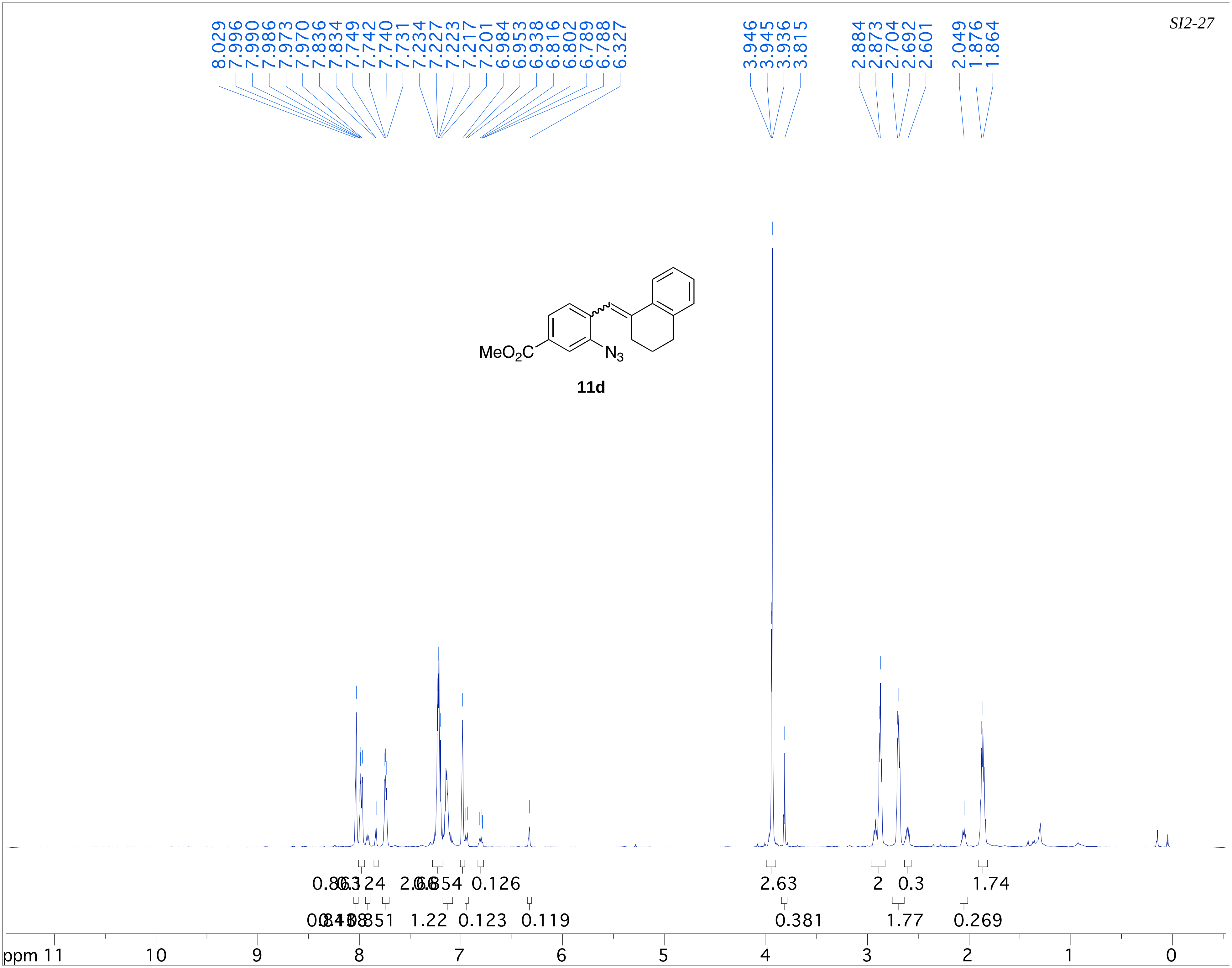
8.029
7.996
7.990
7.986
7.973
7.970
7.836
7.834
7.749
7.742
7.740
7.731
7.234
7.227
7.223
7.217
7.201
6.984
6.953
6.938
6.816
6.802
6.789
6.788
6.327
3.946
3.945
3.936
3.815
2.884
2.873
2.704
2.692
2.601
2.049
1.876
1.864
MeO2C
N3
11d
0.126
2.63
2
0.3
1.74
1.22
0.123
0.119
0.381
1.77
0.269
ppm 11
10
9
8
7
6
5
4
3
2
1
0

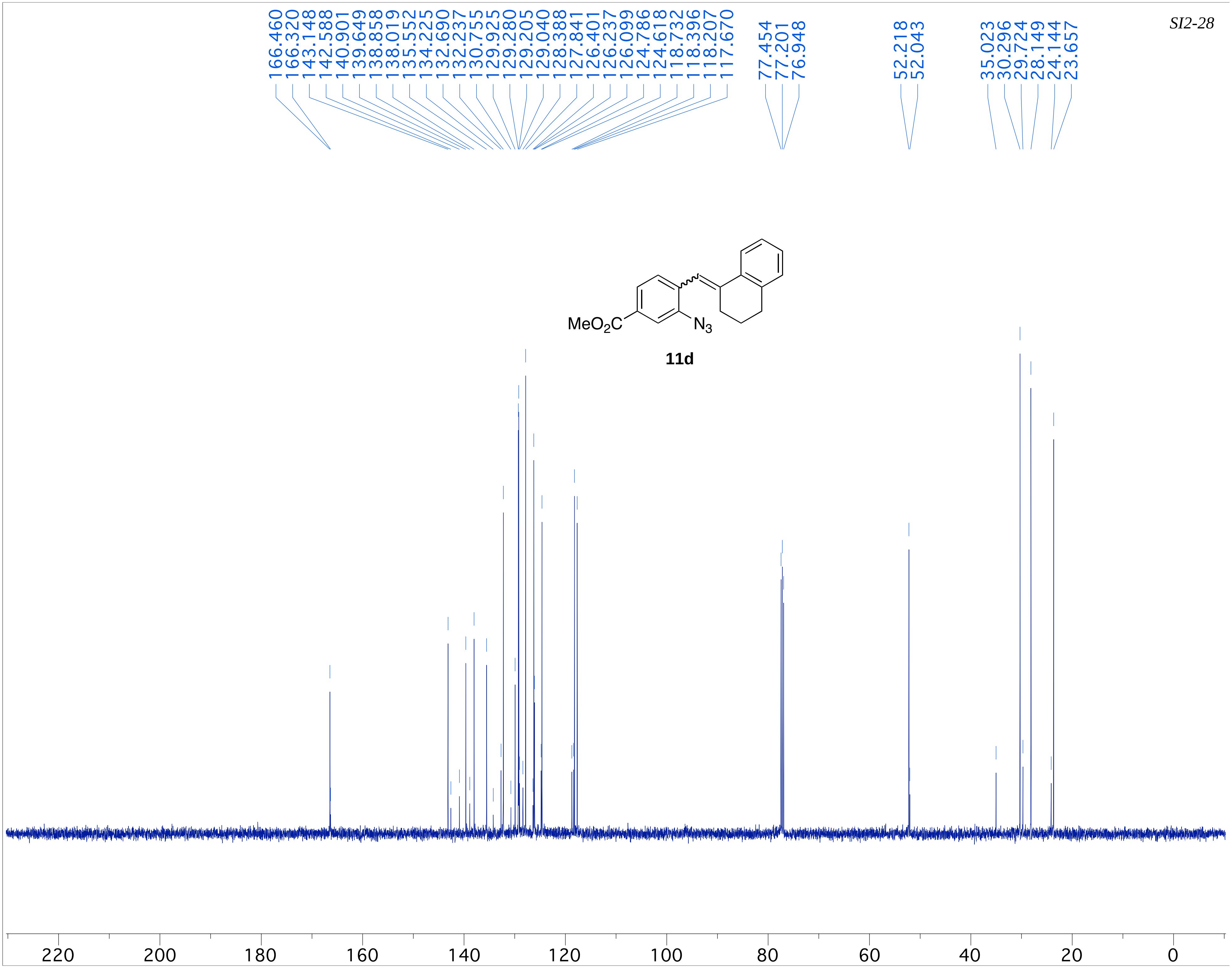
166.460
166.320
143.148
142.588
140.901
139.649
138.858
138.019
135.552
134.225
132.690
132.237
130.755
129.925
129.280
129.205
129.040
128.388
127.841
126.401
126.237
126.099
124.786
124.618
118.732
118.396
118.207
117.670
77.454
77.201
76.948
52.218
52.043
35.023
30.296
29.724
28.149
24.144
23.657
MeO2C
N3
11d
220
200
180
160
140
120
100
80
60
40
20
0

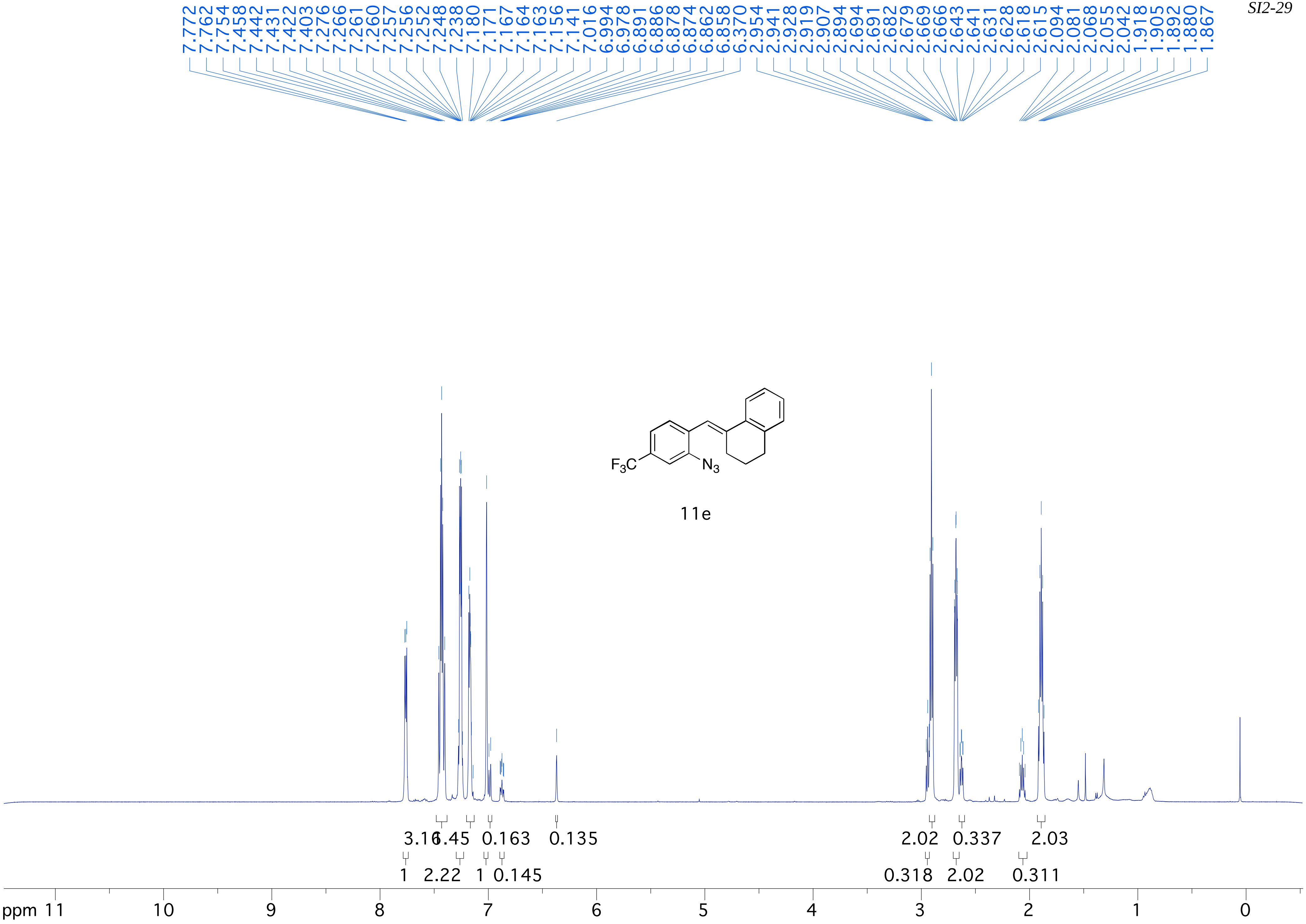
7.772
7.762
7.754
7.458
7.442
7.431
7.422
7.403
7.276
7.266
7.261
7.260
7.257
7.256
7.252
7.248
7.238
7.180
7.171
7.167
7.164
7.163
7.156
7.141
7.016
6.994
6.978
6.891
6.886
6.878
6.874
6.862
6.858
6.370
2.954
2.941
2.928
2.919
2.907
2.894
2.694
2.691
2.682
2.679
2.669
2.666
2.643
2.641
2.631
2.628
2.618
2.615
2.094
2.081
2.068
2.055
2.042
1.918
1.905
1.892
1.880
1.867
F3C
N3
11e
3.16
1.45
0.163
0.135
2.02
0.337
2.03
1
2.22
1
0.145
0.318
2.02
0.311
ppm 11
10
9
8
7
6
5
4
3
2
1
0

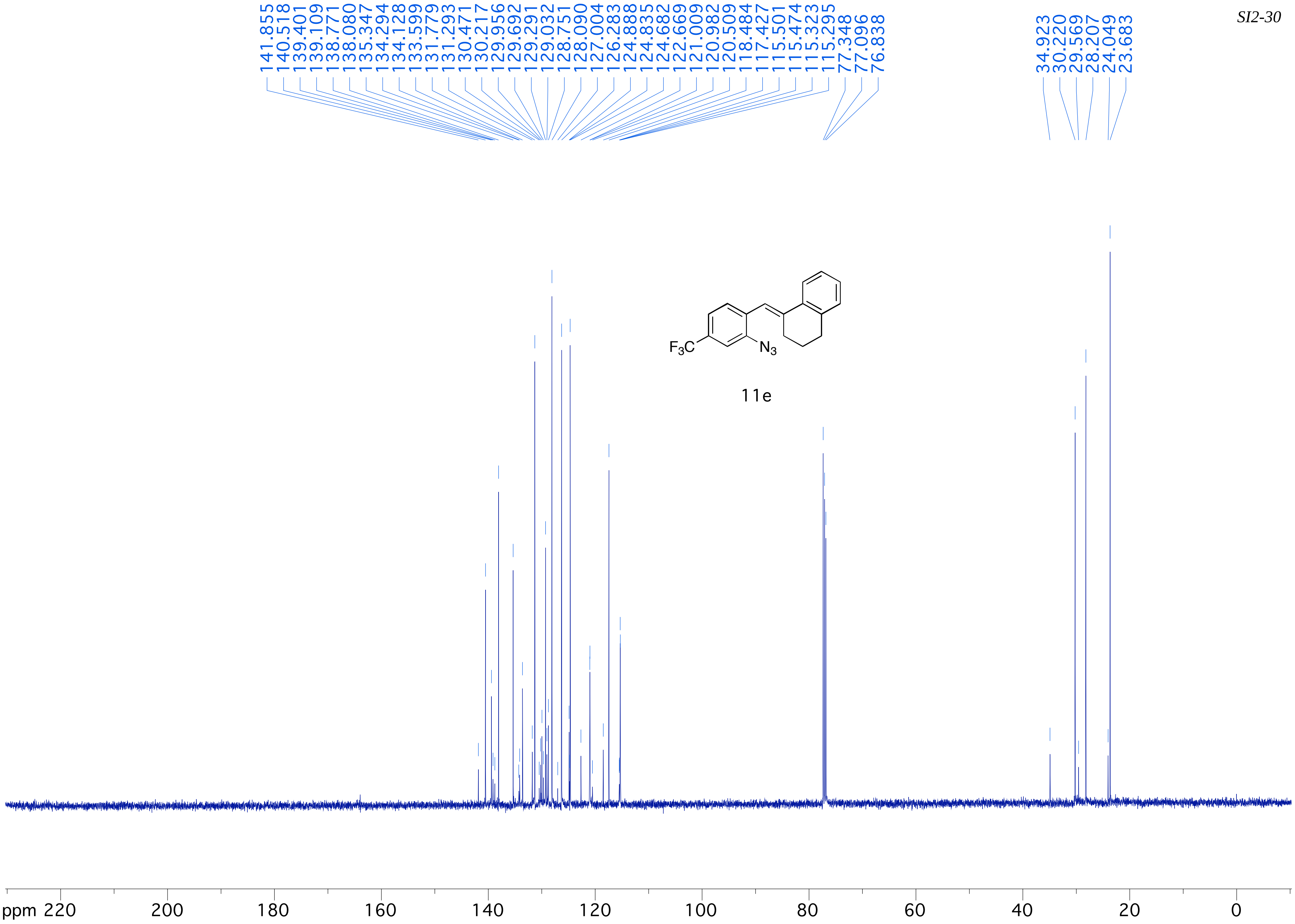
141.855
140.518
139.401
139.109
138.771
138.080
135.347
134.294
134.128
133.599
131.779
131.293
130.471
130.217
129.956
129.692
129.291
129.032
128.751
128.090
127.004
126.283
124.888
124.835
124.682
122.669
121.009
120.982
120.509
118.484
117.427
115.501
115.474
115.323
115.295
77.348
77.096
76.838
34.923
30.220
29.569
28.207
24.049
23.683
F3C
N3
11e
ppm 220
200
180
160
140
120
100
80
60
40
20
0

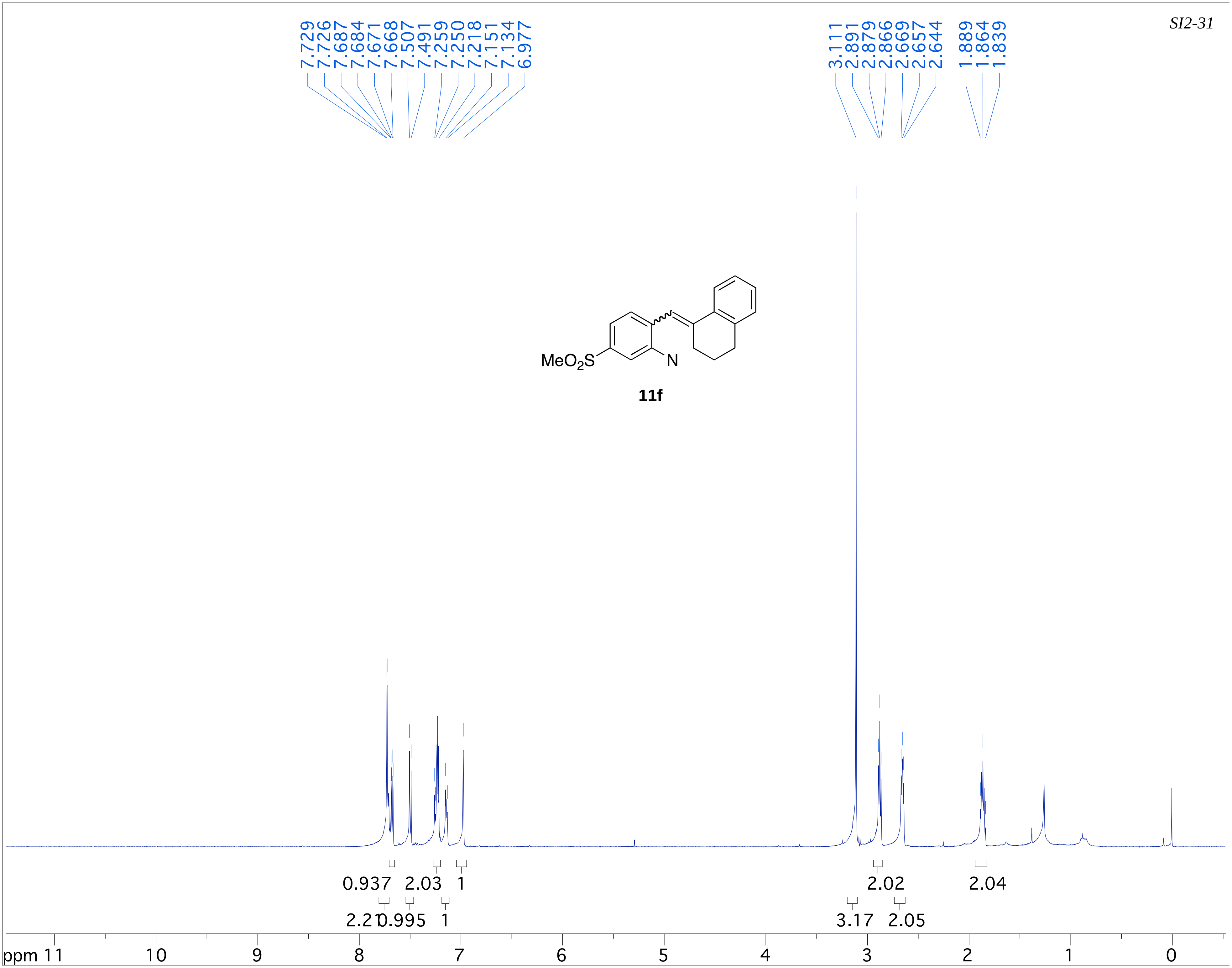
7.729
7.726
7.687
7.684
7.671
7.668
7.507
7.491
7.259
7.250
7.218
7.151
7.134
6.977
3.111
2.891
2.879
2.866
2.669
2.657
2.644
1.889
1.864
1.839
MeO2S
N
11f
0.937
2.03
1
2.21
0.995
1
2.02
2.04
3.17
2.05
ppm 11
10
9
8
7
6
5
4
3
2
1
0

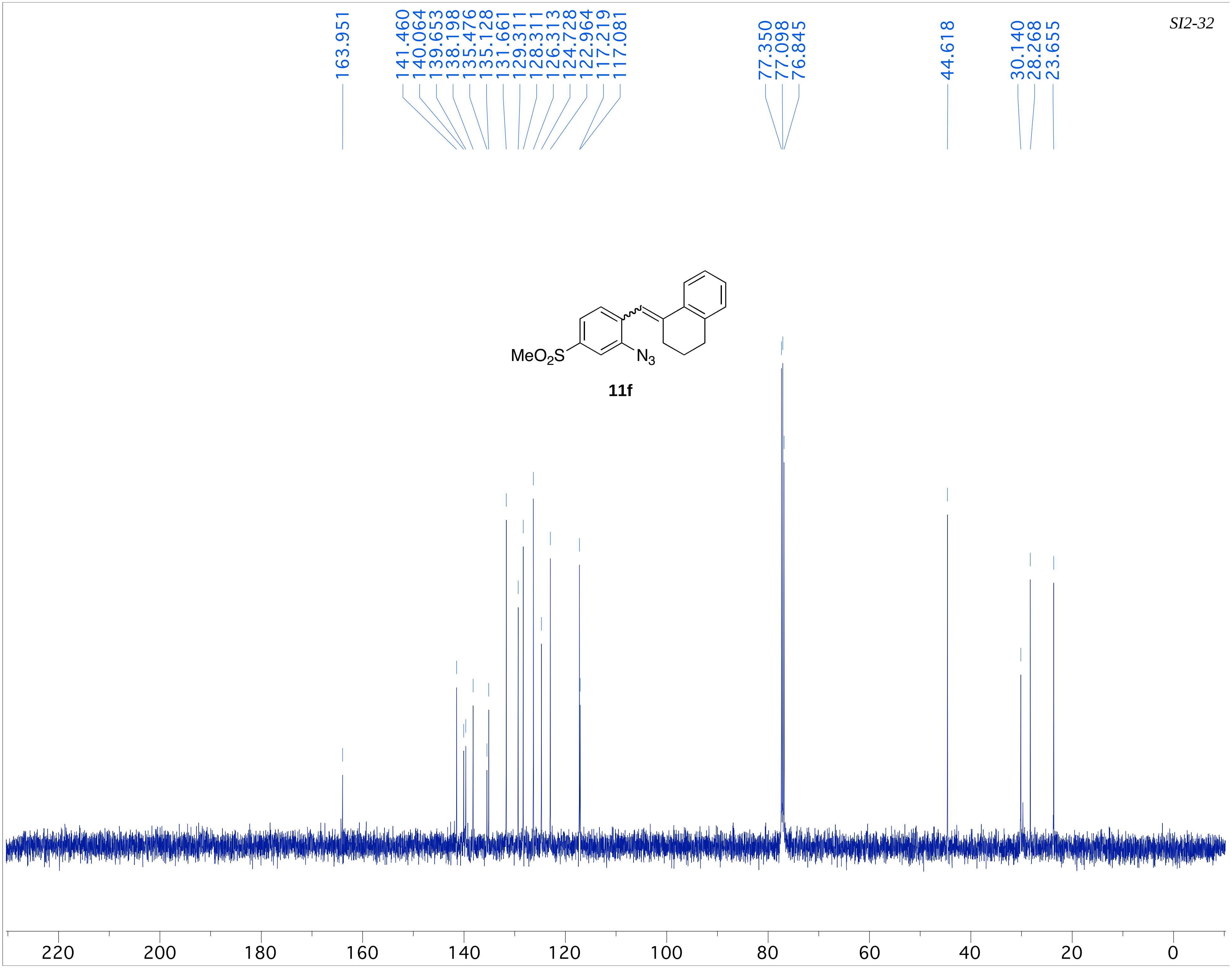
163.951
141.460
140.064
139.653
138.198
135.476
135.128
131.661
129.311
128.311
126.313
124.728
122.964
117.219
117.081
77.350
77.098
76.845
44.618
30.140
28.268
23.655
MeO2S
N3
11f
220
200
180
160
140
120
100
80
60
40
20
0

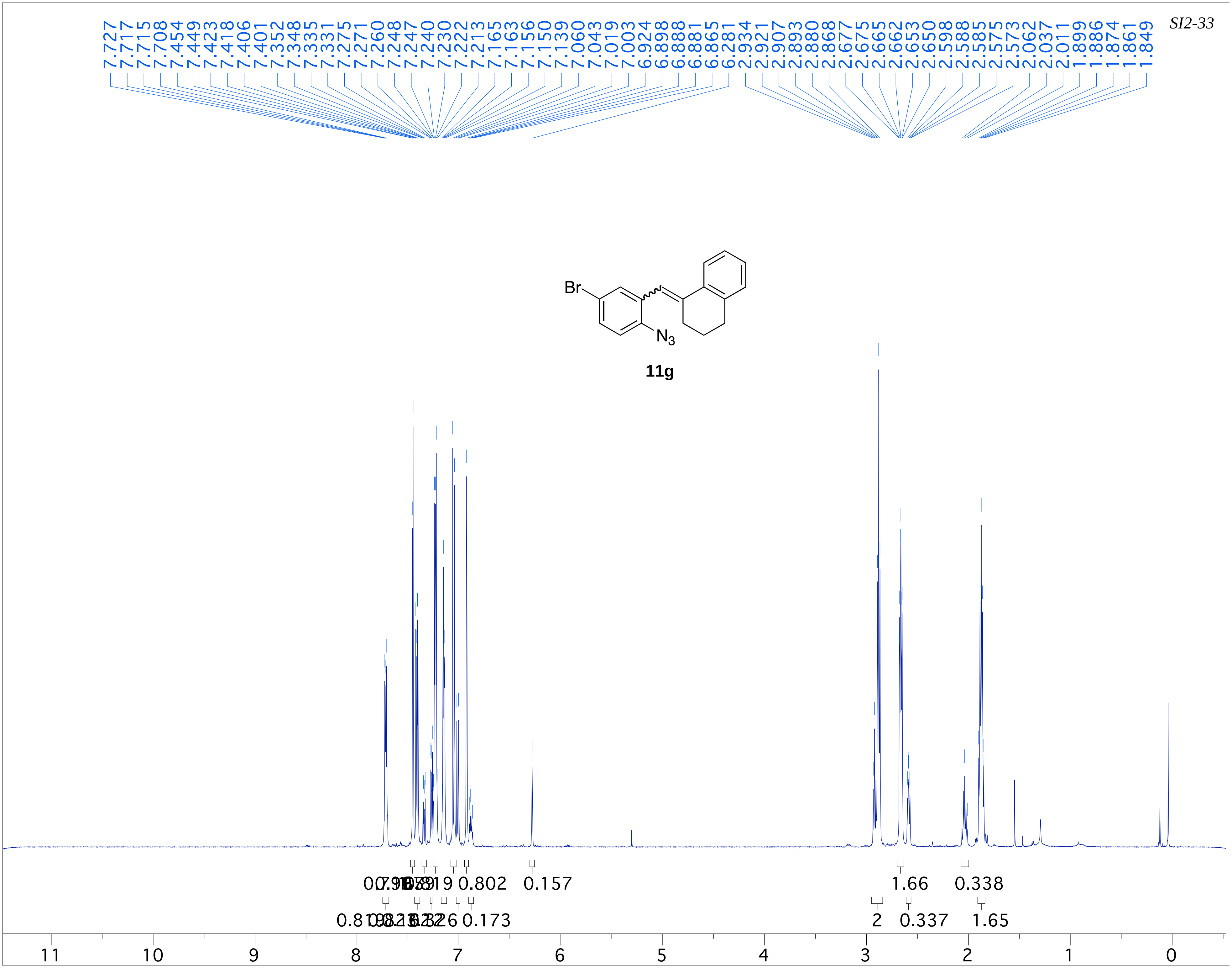
7.727
7.717
7.715
7.708
7.454
7.449
7.423
7.418
7.406
7.401
7.352
7.348
7.335
7.331
7.275
7.271
7.260
7.248
7.247
7.240
7.230
7.222
7.213
7.165
7.163
7.156
7.150
7.139
7.060
7.043
7.019
7.003
6.924
6.898
6.888
6.881
6.865
6.281
2.934
2.921
2.907
2.893
2.880
2.868
2.677
2.675
2.665
2.662
2.653
2.650
2.598
2.588
2.585
2.575
2.573
2.062
2.037
2.011
1.899
1.886
1.874
1.861
1.849
Br
N3
11g
0.802
0.157
1.66
0.338
0.173
2
0.337
1.65
11
10
9
8
7
6
5
4
3
2
1
0

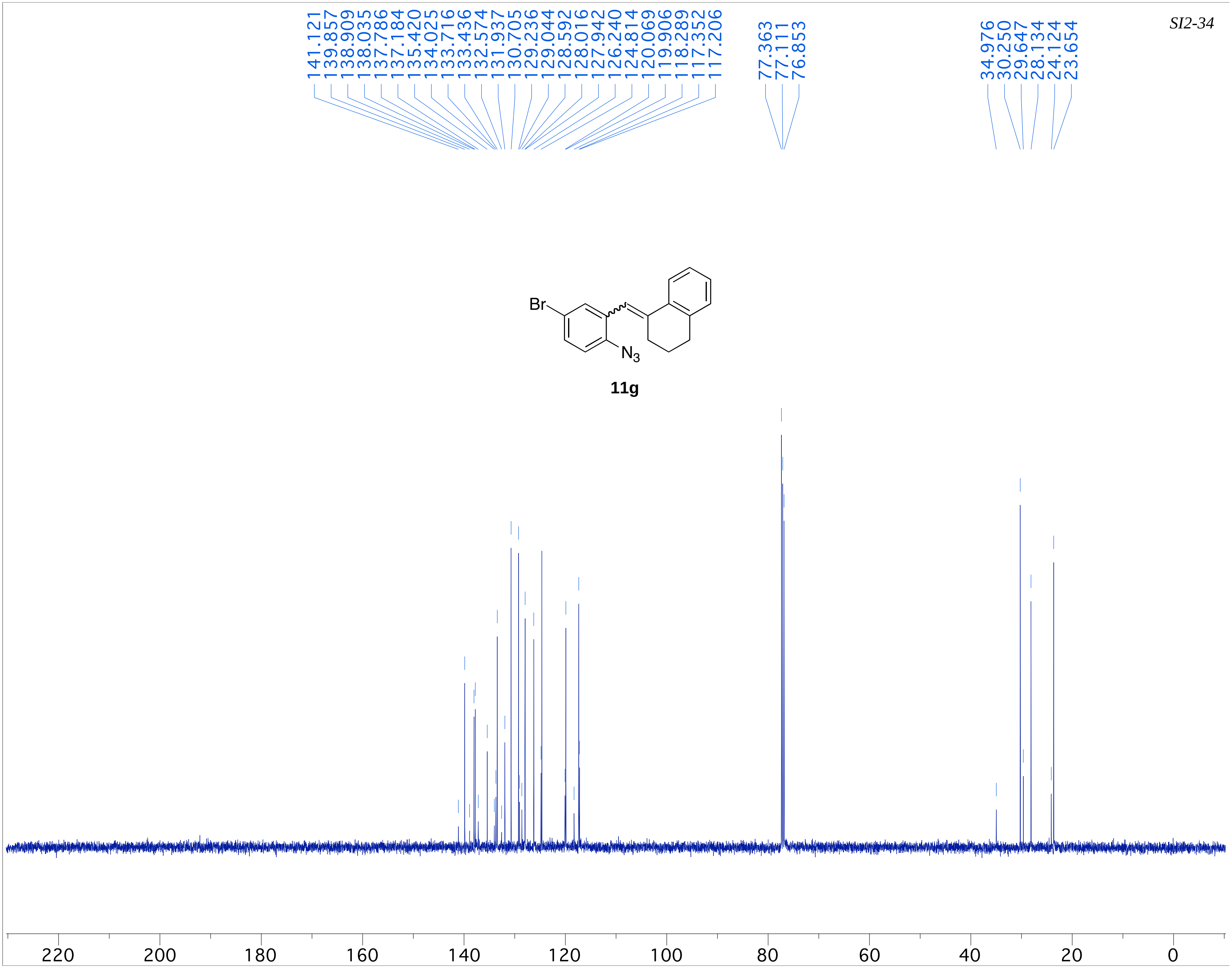
141.121
139.857
138.909
138.035
137.786
137.184
135.420
134.025
133.716
133.436
132.574
131.937
130.705
129.236
129.044
128.592
128.016
127.942
126.240
124.814
120.069
119.906
118.289
117.352
117.206
77.363
77.111
76.853
34.976
30.250
29.647
28.134
24.124
23.654
Br
N3
11g
220
200
180
160
140
120
100
80
60
40
20
0

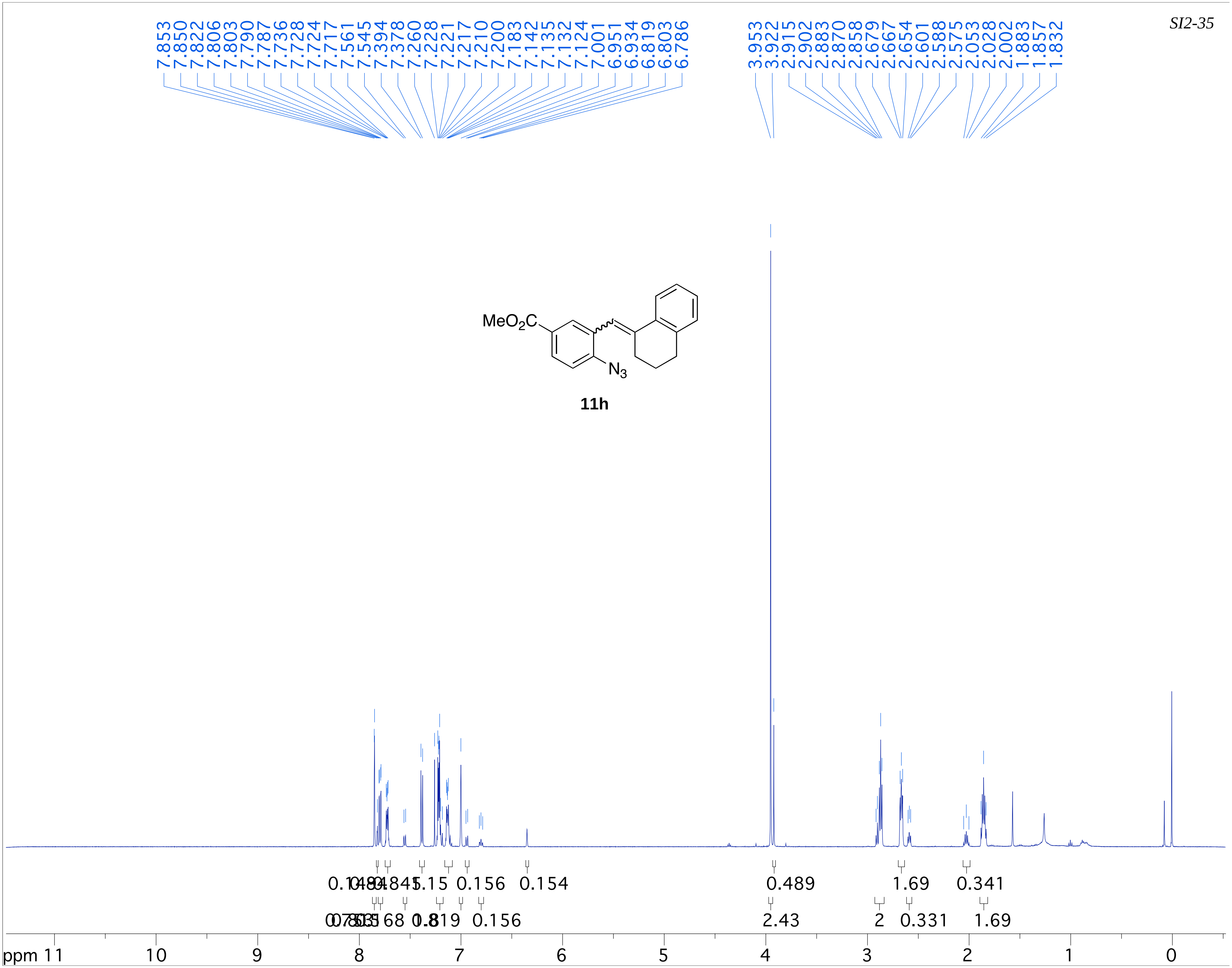
SI2-35
7.853 7.850 7.822 7.806 7.803 7.790 7.787 7.736 7.728 7.724 7.717 7.561 7.545 7.394 7.378 7.260 7.228 7.221 7.217 7.210 7.200 7.183 7.142 7.135 7.132 7.124 7.001 6.951 6.934 6.819 6.803 6.786
3.953 3.922 2.915 2.902 2.883 2.870 2.858 2.679 2.667 2.654 2.601 2.588 2.575 2.053 2.028 2.002 1.883 1.857 1.832
MeO2C
N3
11h
0.154
0.489
1.69
0.341
2.43
2
0.331
1.69
0.156
ppm 11
10
9
8
7
6
5
4
3
2
1
0

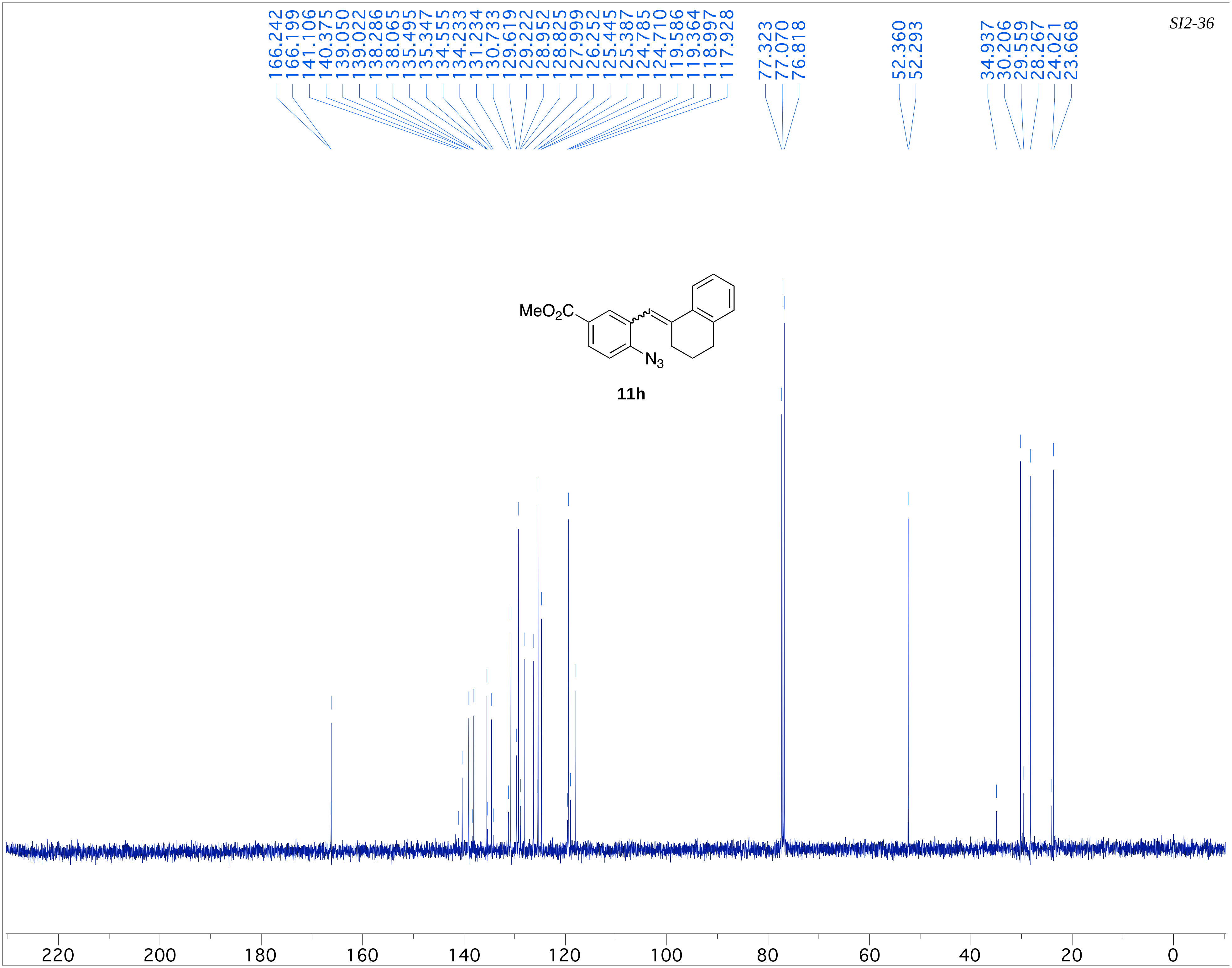
166.242
166.199
141.106
140.375
139.050
139.022
138.286
138.065
135.495
135.347
134.555
134.233
131.234
130.733
129.619
129.222
128.952
128.825
127.999
126.252
125.445
125.387
124.785
124.710
119.586
119.364
118.997
117.928
77.323
77.070
76.818
52.360
52.293
34.937
30.206
29.559
28.267
24.021
23.668
MeO2C
N3
11h
220
200
180
160
140
120
100
80
60
40
20
0

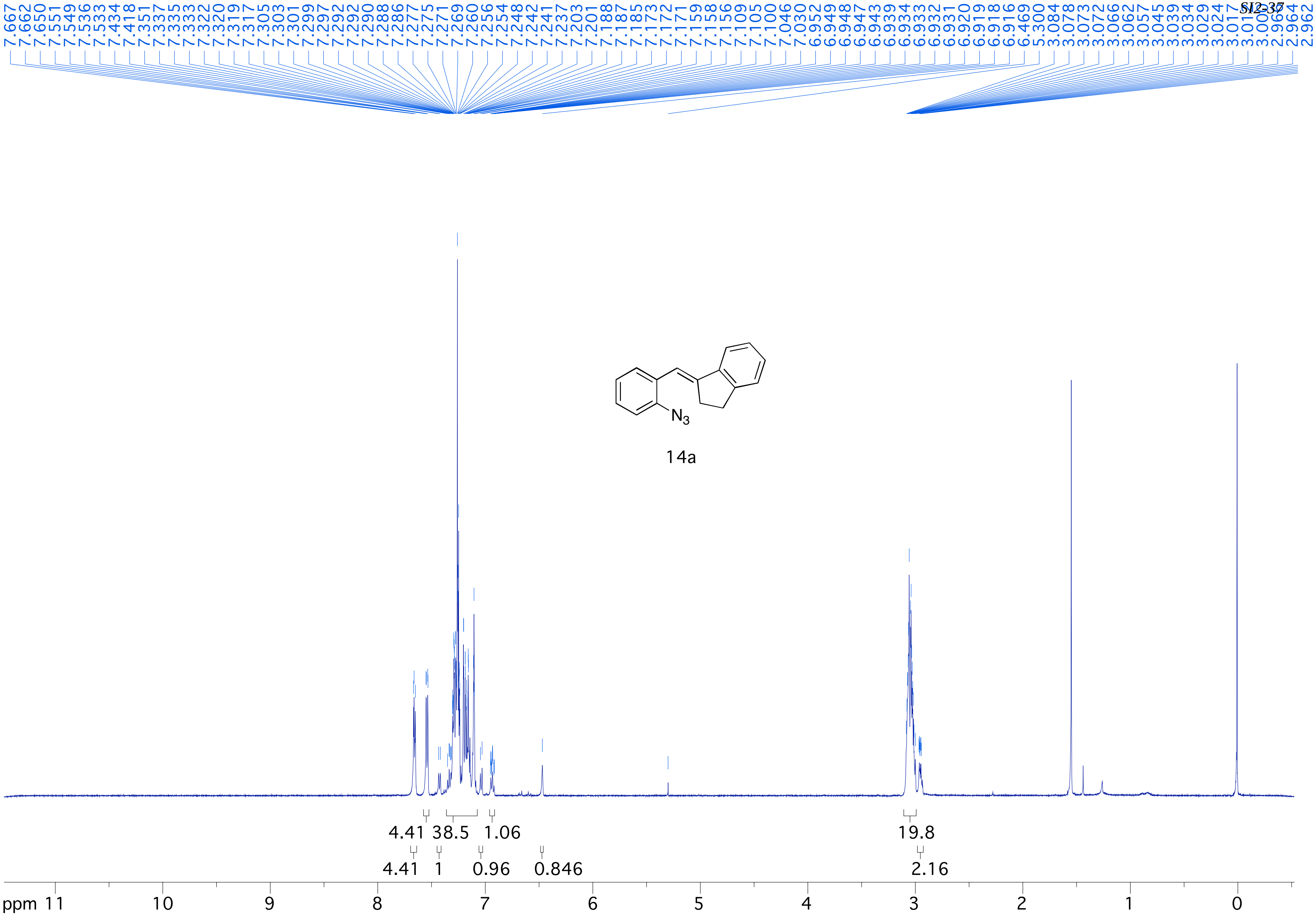
7.667
7.662
7.650
7.551
7.549
7.536
7.533
7.434
7.418
7.351
7.337
7.335
7.333
7.322
7.320
7.319
7.317
7.305
7.303
7.301
7.299
7.297
7.292
7.292
7.290
7.288
7.286
7.277
7.275
7.271
7.269
7.260
7.256
7.254
7.248
7.242
7.241
7.237
7.203
7.201
7.188
7.187
7.185
7.173
7.172
7.171
7.159
7.158
7.156
7.109
7.105
7.100
7.046
7.030
6.952
6.949
6.948
6.947
6.943
6.939
6.934
6.933
6.932
6.931
6.920
6.919
6.918
6.916
6.469
5.300
3.084
3.078
3.073
3.072
3.066
3.062
3.057
3.045
3.039
3.034
3.029
3.024
2.964
2.962
N_3
14a
4.41
38.5
1.06
19.8
4.41
1
0.96
0.846
2.16
ppm
11
10
9
8
7
6
5
4
3
2
1
0

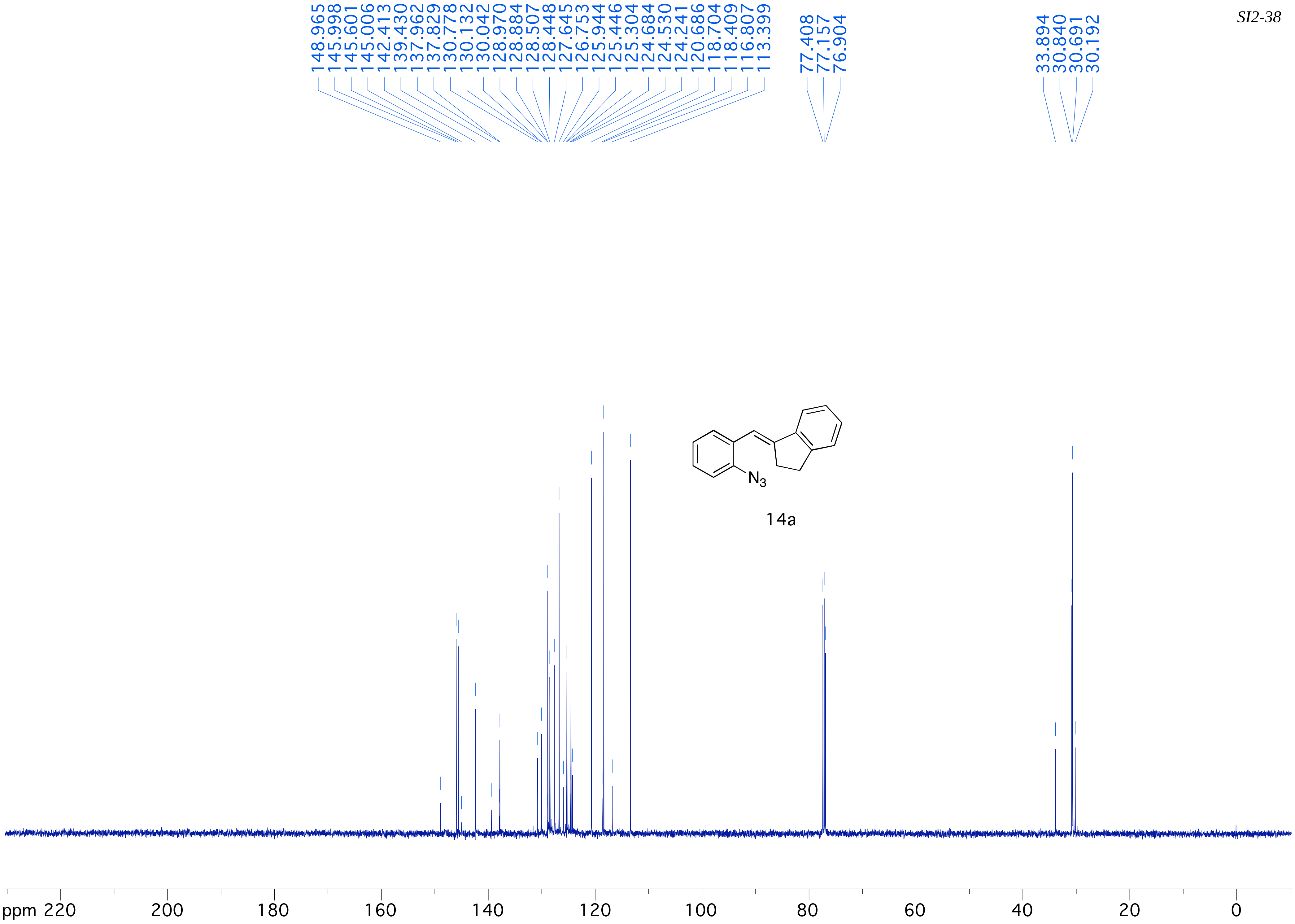
148.965
145.998
145.601
145.006
142.413
139.430
137.962
137.829
130.778
130.132
130.042
128.970
128.884
128.507
128.448
127.645
126.753
125.944
125.446
125.304
124.684
124.530
124.241
120.686
118.704
118.409
116.807
113.399
77.408
77.157
76.904
33.894
30.840
30.691
30.192
N_3
14a
ppm 220 200 180 160 140 120 100 80 60 40 20 0

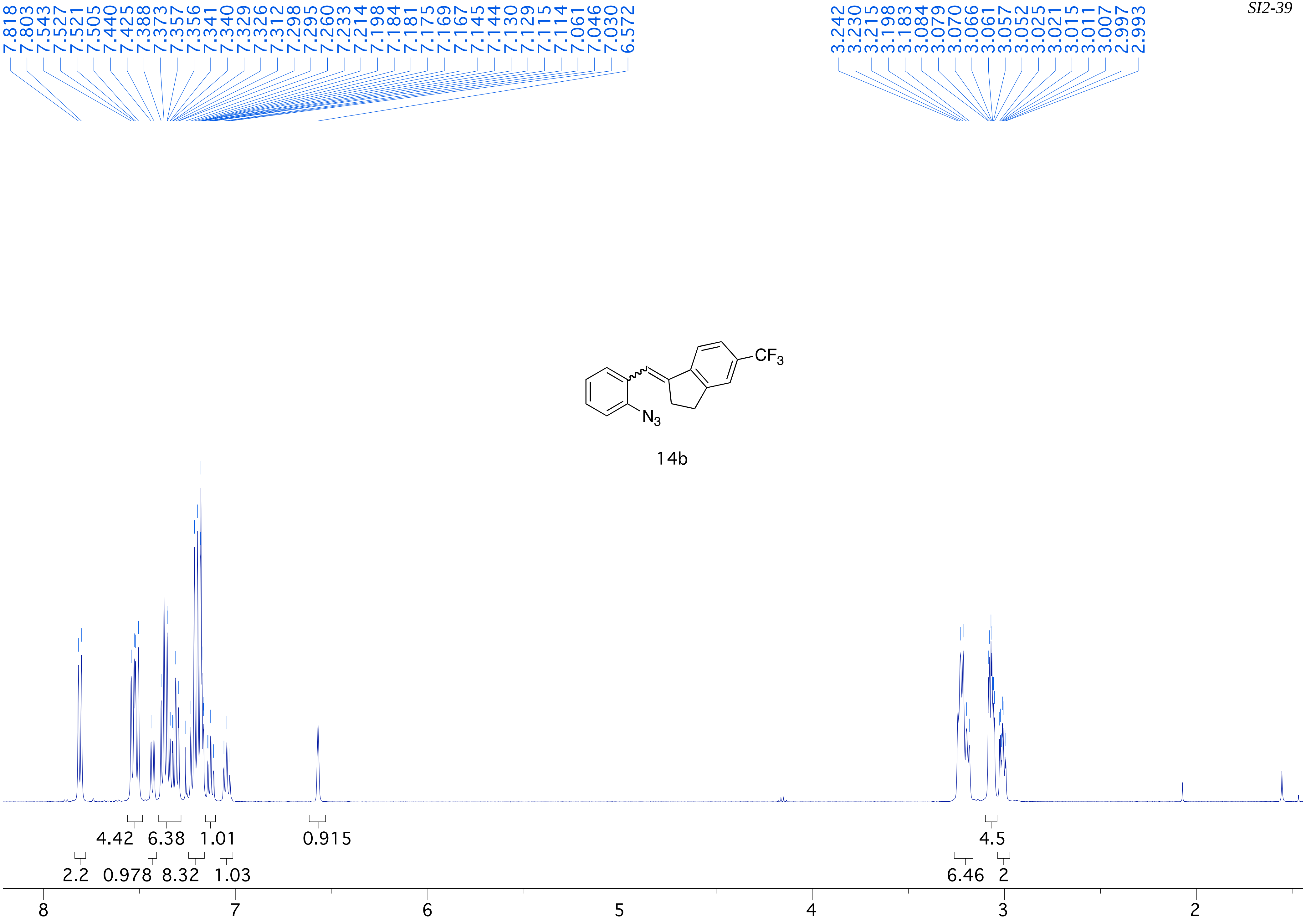

7.818
7.803
7.543
7.527
7.521
7.505
7.440
7.425
7.388
7.373
7.357
7.356
7.341
7.340
7.329
7.326
7.312
7.298
7.295
7.260
7.233
7.214
7.198
7.184
7.181
7.175
7.169
7.167
7.145
7.144
7.130
7.129
7.115
7.114
7.061
7.046
7.030
6.572
3.242
3.230
3.215
3.198
3.183
3.084
3.079
3.070
3.066
3.061
3.057
3.052
3.025
3.021
3.015
3.011
3.007
2.997
2.993
4.42
6.38
1.01
0.915
4.5
2.2
0.978
8.32
1.03
6.46
2
8
7
6
5
4
3
2
CF3
N3

14b

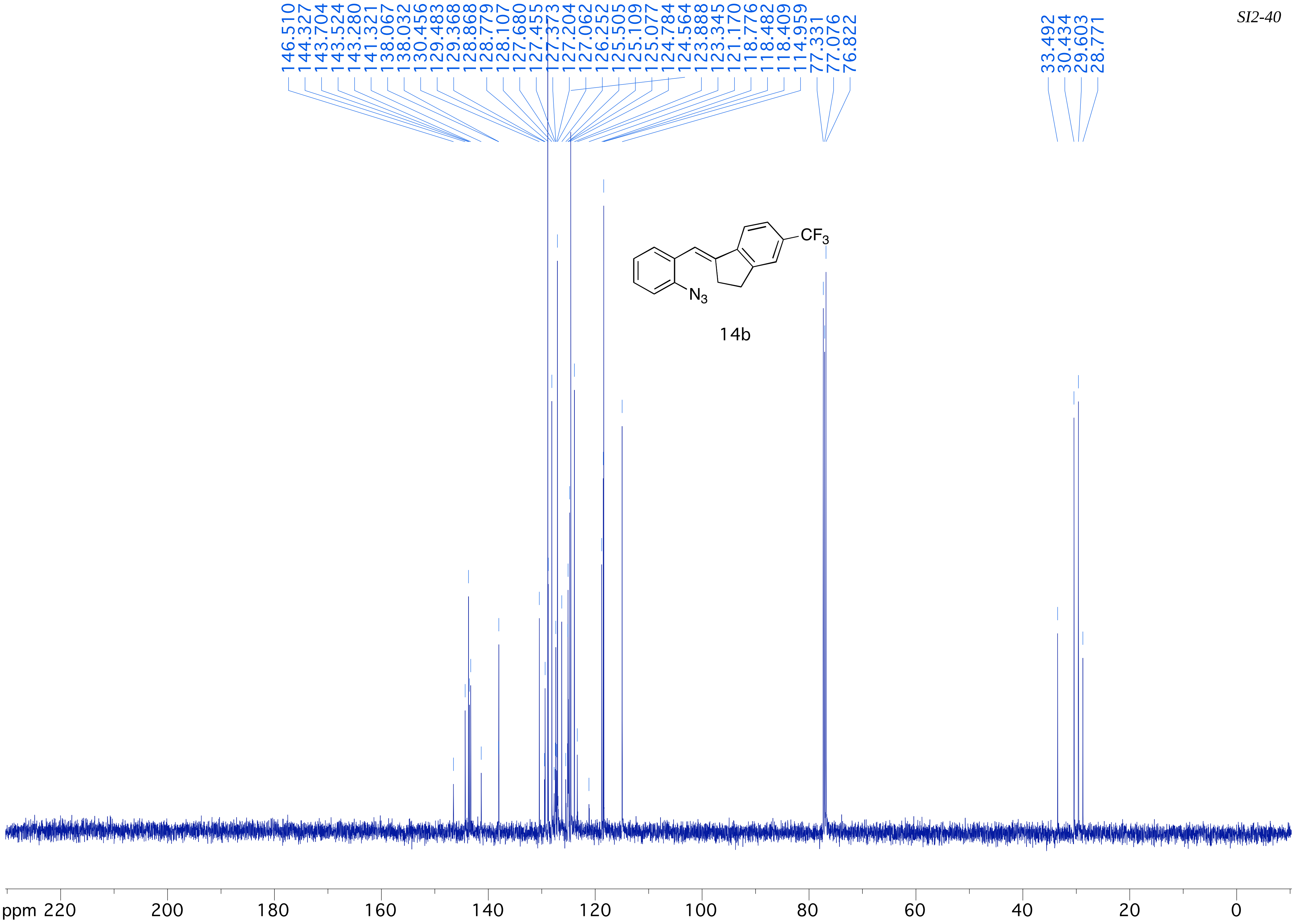
146.510
144.327
143.704
143.524
143.280
141.321
138.067
138.032
130.456
129.483
129.368
128.868
128.779
128.107
127.680
127.455
127.373
127.204
127.062
126.252
125.505
125.109
125.077
124.784
124.564
123.888
123.345
121.170
118.776
118.482
118.409
114.959
77.331
77.076
76.822
33.492
30.434
29.603
28.771
CF3
N3
14b
ppm 220
200
180
160
140
120
100
80
60
40
20
0

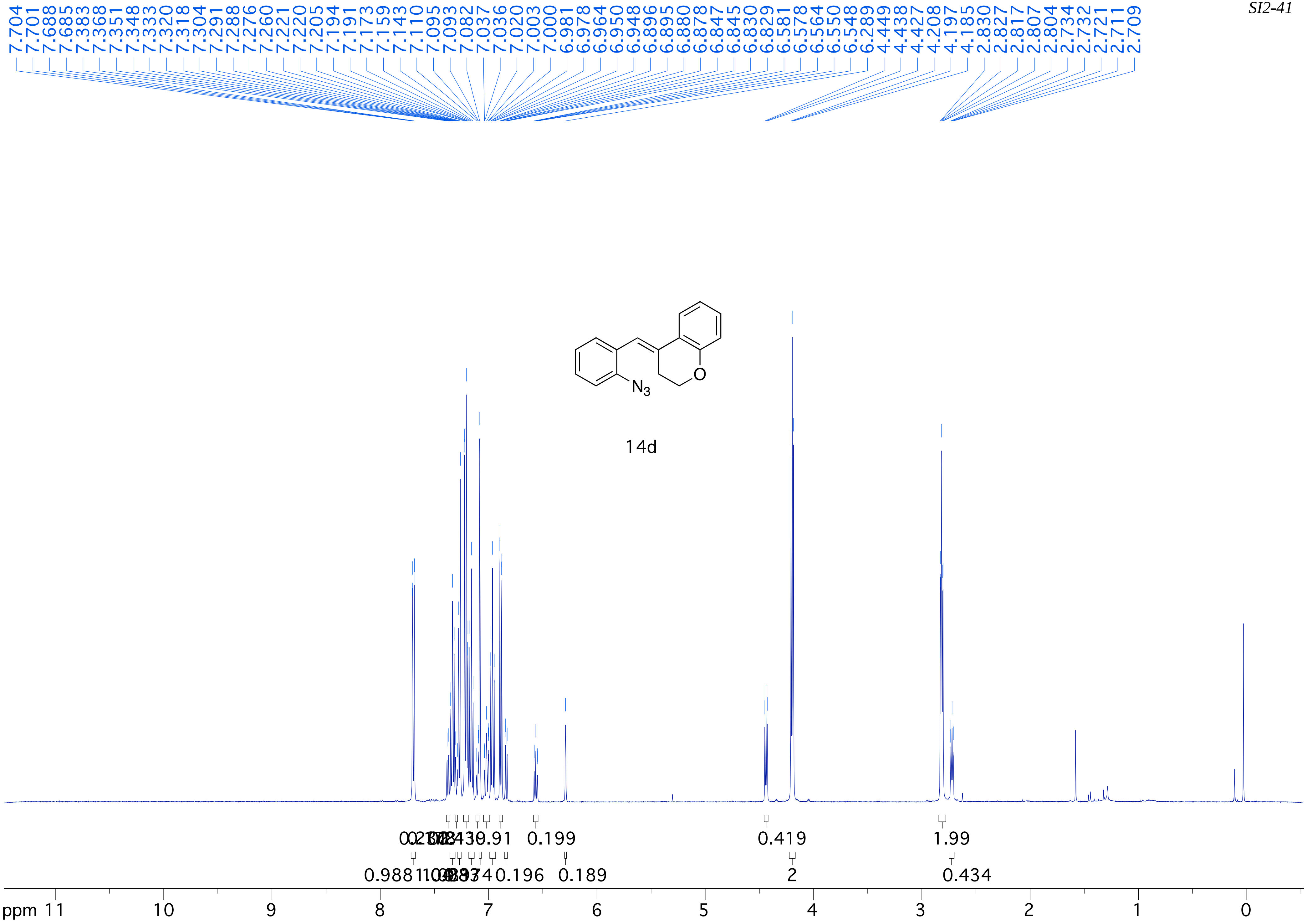
7.704
7.701
7.688
7.685
7.383
7.368
7.351
7.348
7.333
7.320
7.318
7.304
7.291
7.288
7.276
7.260
7.221
7.220
7.205
7.194
7.191
7.173
7.159
7.143
7.110
7.095
7.093
7.082
7.037
7.036
7.020
7.003
7.000
6.981
6.978
6.964
6.950
6.948
6.896
6.895
6.880
6.878
6.847
6.845
6.830
6.829
6.581
6.578
6.564
6.550
6.548
6.289
4.449
4.438
4.427
4.208
4.197
4.185
2.830
2.827
2.817
2.807
2.804
2.734
2.732
2.721
2.711
2.709
N3
O
14d
0.199
0.419
1.99
0.988
0.196
0.189
2
0.434
ppm 11
10
9
8
7
6
5
4
3
2
1
0

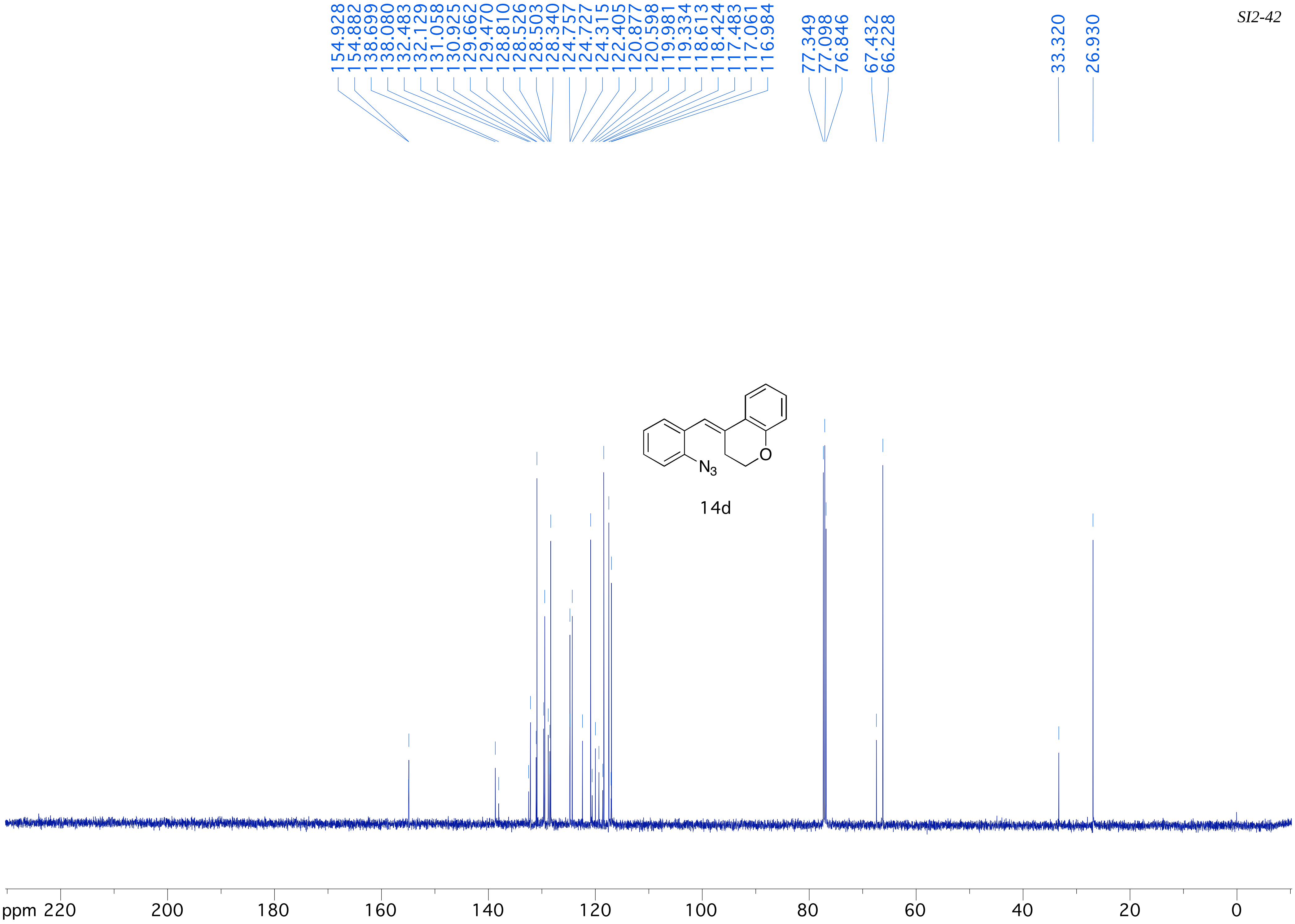
154.928
154.882
138.699
138.080
132.483
132.129
131.058
130.925
129.662
129.470
128.810
128.526
128.503
128.340
124.757
124.727
124.315
122.405
120.877
120.598
119.981
119.334
118.613
118.424
117.483
117.061
116.984
77.349
77.098
76.846
67.432
66.228
33.320
26.930
N3
O
14d
ppm 220
200
180
160
140
120
100
80
60
40
20
0

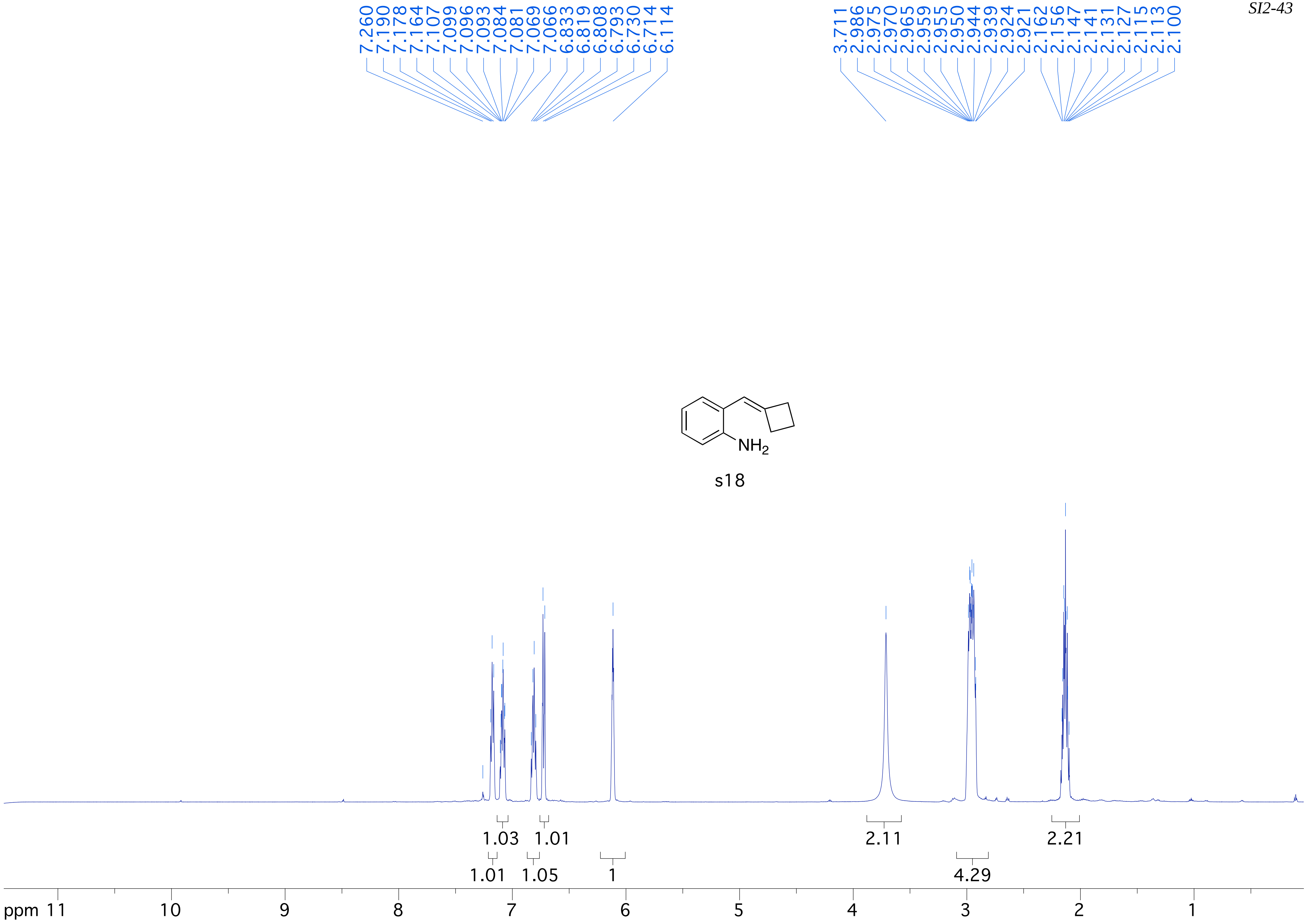
7.260
7.190
7.178
7.164
7.107
7.099
7.096
7.093
7.084
7.081
7.069
7.066
6.833
6.819
6.808
6.793
6.730
6.714
6.114
3.711
2.986
2.975
2.970
2.965
2.959
2.955
2.950
2.944
2.939
2.924
2.921
2.162
2.156
2.147
2.141
2.131
2.127
2.115
2.113
2.100
NH2
s18
1.03
1.01
2.11
2.21
1.01
1.05
1
4.29
ppm 11
10
9
8
7
6
5
4
3
2
1

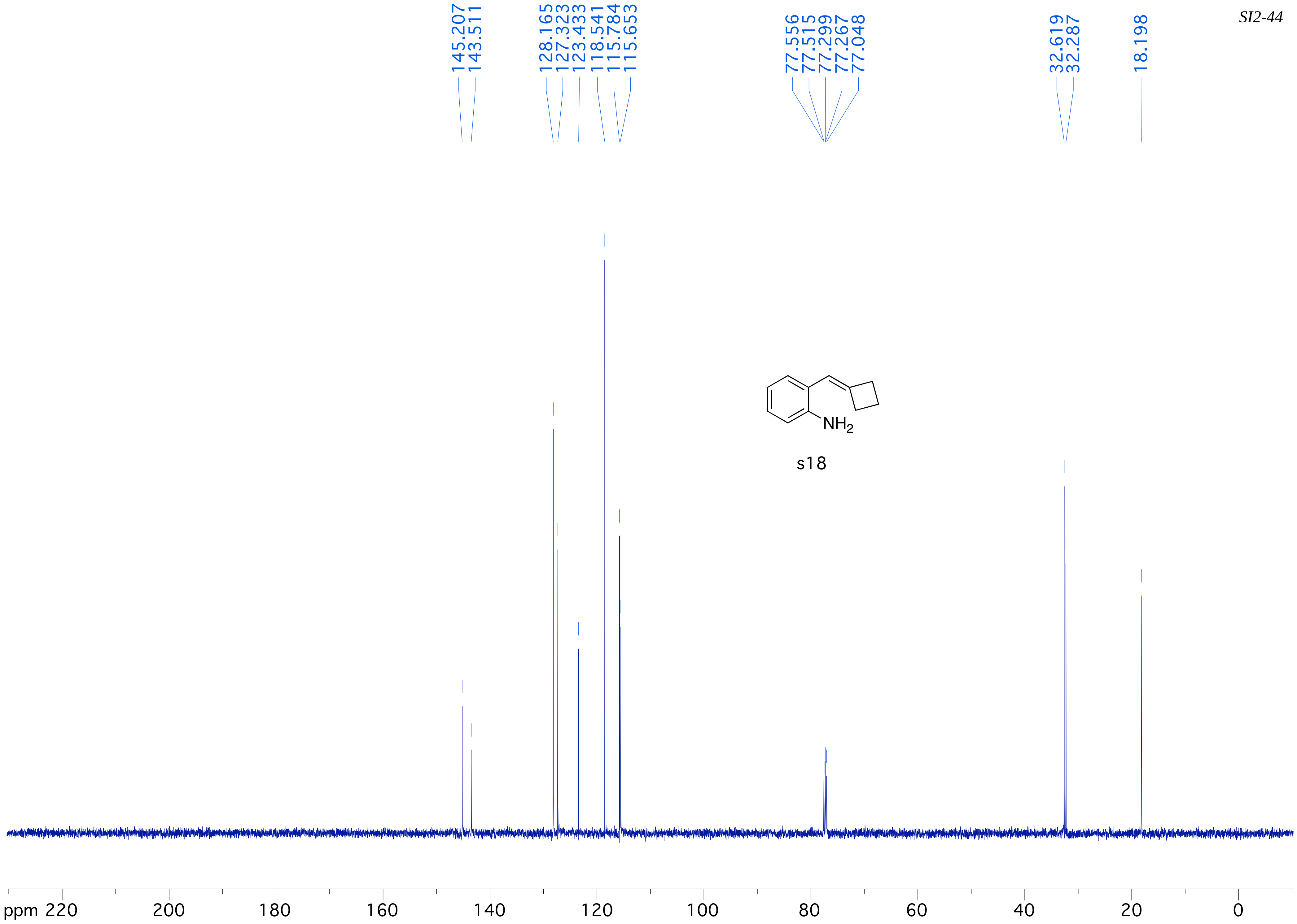
145.207
143.511
128.165
127.323
123.433
118.541
115.784
115.653
77.556
77.515
77.299
77.267
77.048
32.619
32.287
18.198
NH_2
s18
ppm 220
200
180
160
140
120
100
80
60
40
20
0

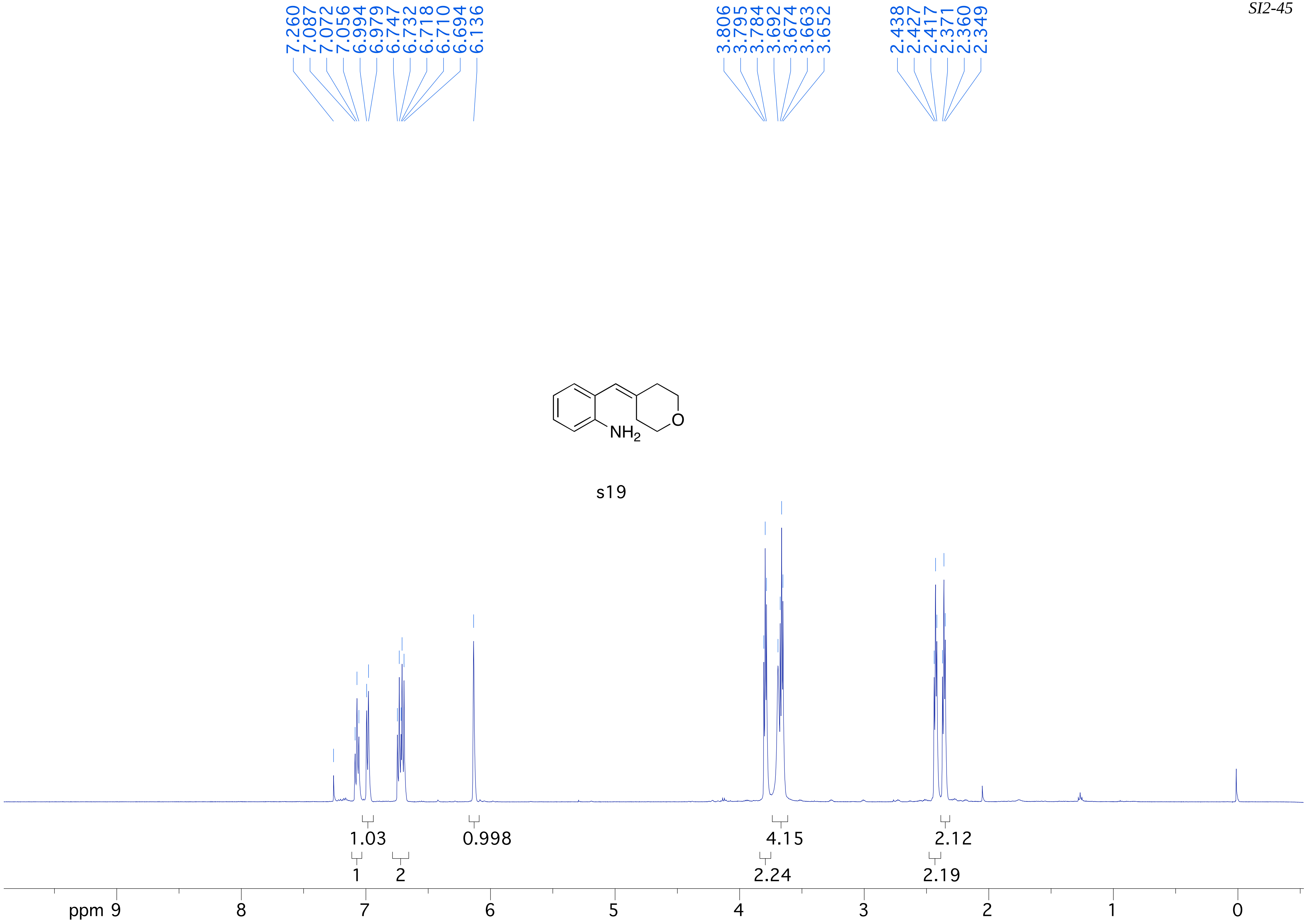
7.260
7.087
7.072
7.056
6.994
6.979
6.747
6.732
6.718
6.710
6.694
6.136
3.806
3.795
3.784
3.692
3.674
3.663
3.652
2.438
2.427
2.417
2.371
2.360
2.349
NH2
O
s19
1.03
0.998
4.15
2.12
1
2
2.24
2.19
ppm 9
8
7
6
5
4
3
2
1
0

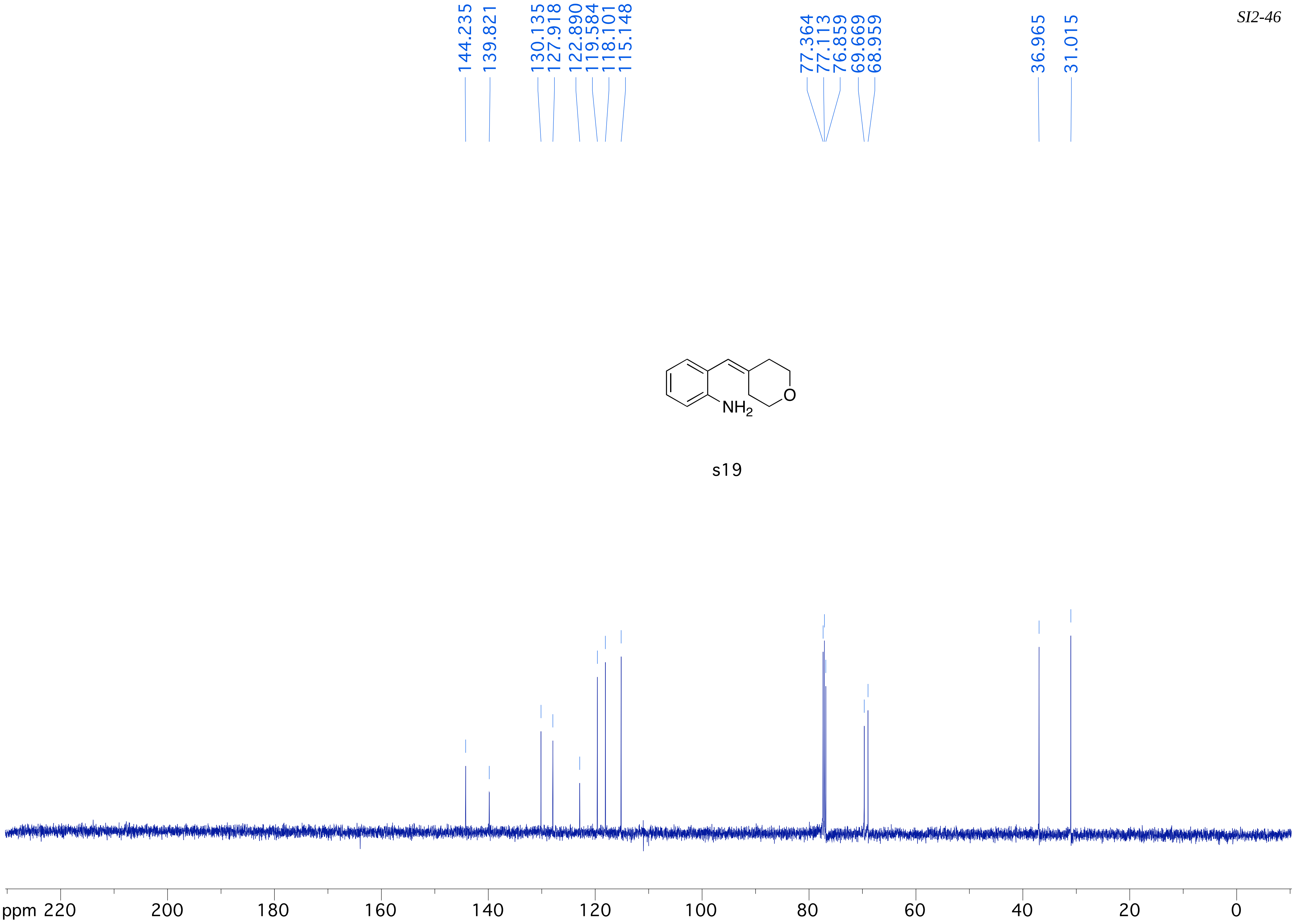
144.235
139.821
130.135
127.918
122.890
119.584
118.101
115.148
77.364
77.113
76.859
69.669
68.959
36.965
31.015
NH2
O
s19
ppm 220
200
180
160
140
120
100
80
60
40
20
0

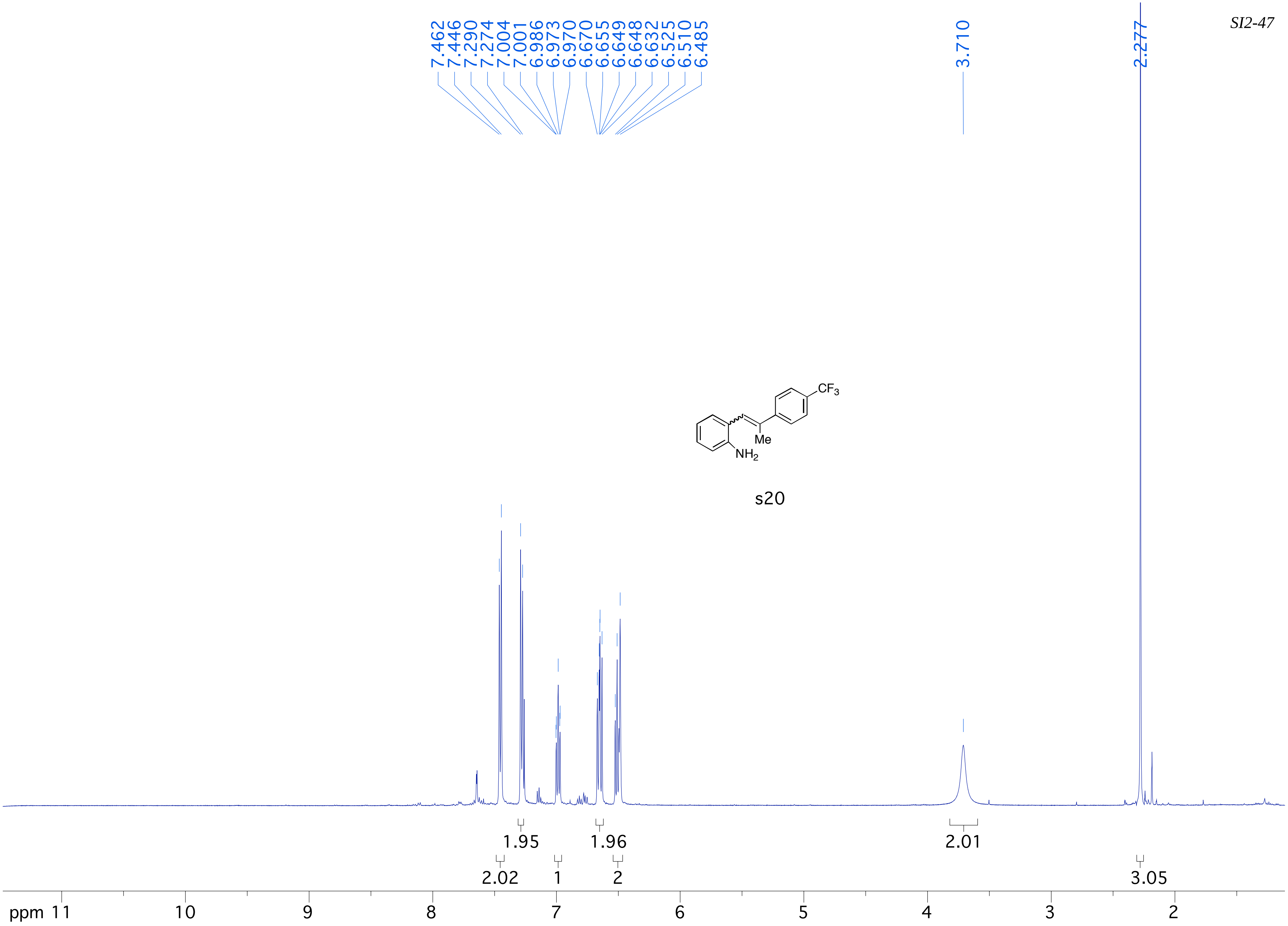
7.462
7.446
7.290
7.274
7.004
7.001
6.986
6.973
6.970
6.670
6.655
6.649
6.648
6.632
6.525
6.510
6.485
3.710
2.277
CF3
Me
NH2
1.95
1.96
2.01
2.02
1
2
3.05
ppm 11
10
9
8
7
6
5
4
3
2

s20

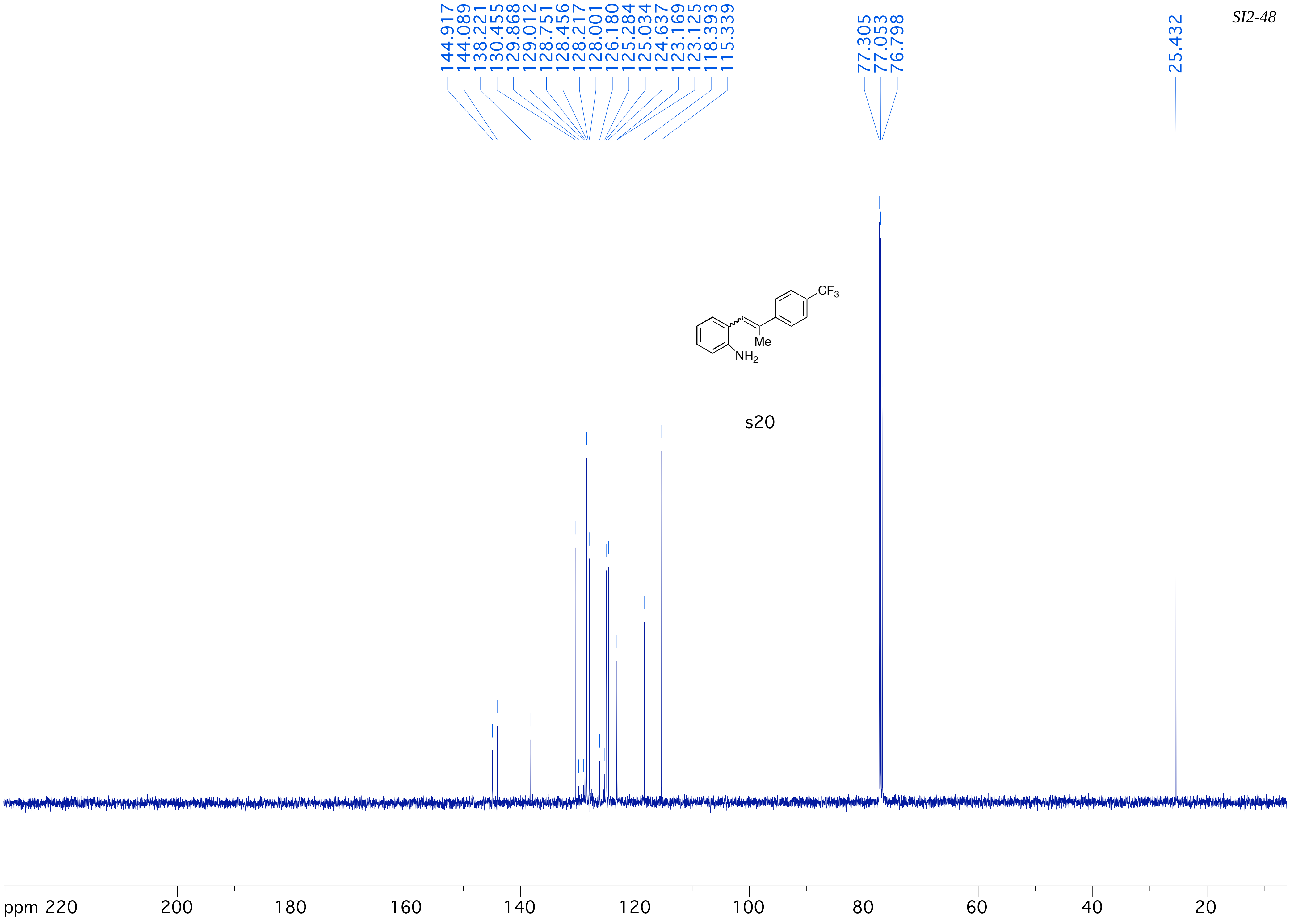
144.917
144.089
138.221
130.455
129.868
129.012
128.751
128.456
128.217
128.001
126.180
125.284
125.034
124.637
123.169
123.125
118.393
115.339
77.305
77.053
76.798
25.432
CF3
Me
NH2
s20
ppm 220
200
180
160
140
120
100
80
60
40
20

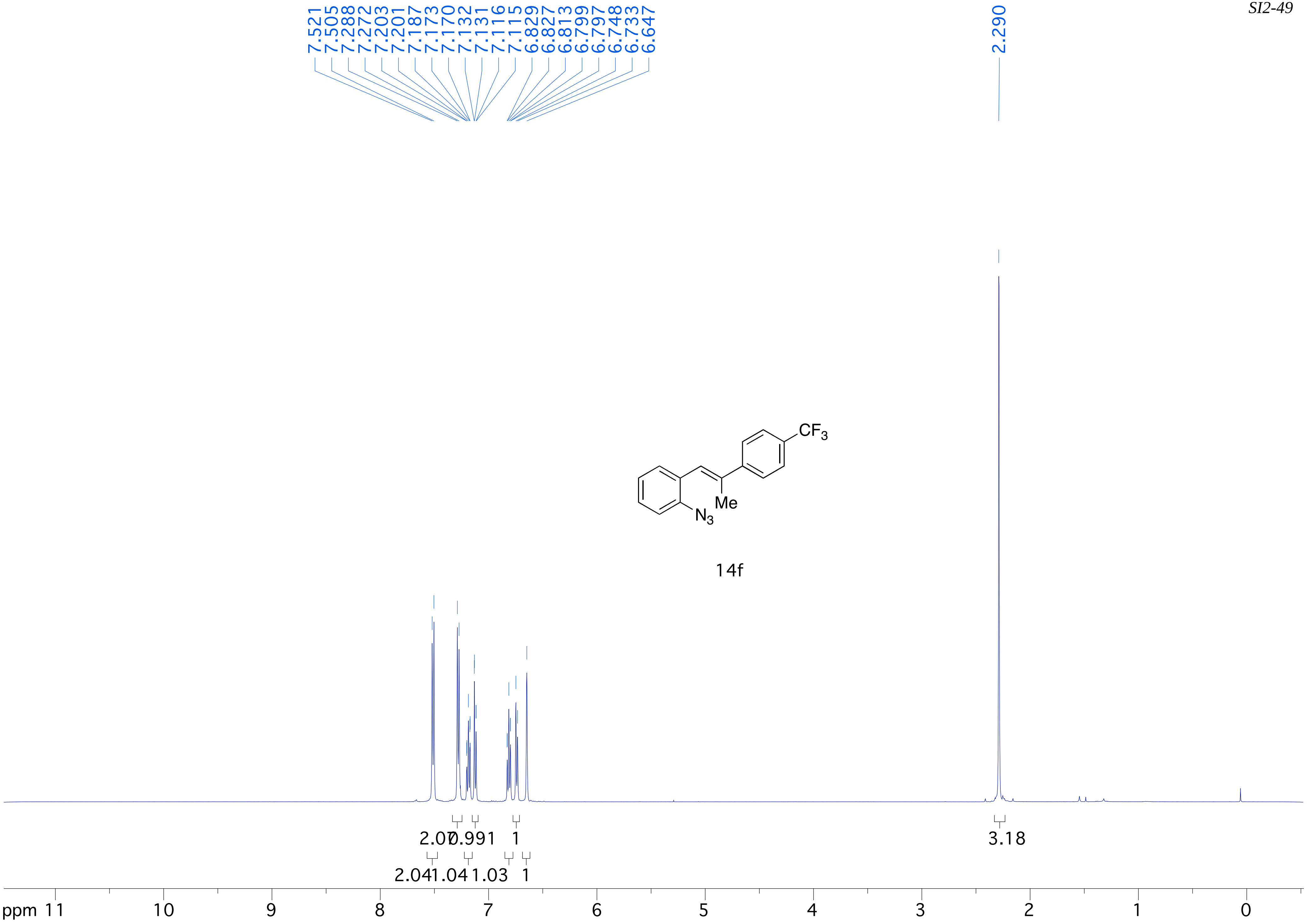
7.521
7.505
7.288
7.272
7.203
7.201
7.187
7.173
7.170
7.132
7.131
7.116
7.115
6.829
6.827
6.813
6.799
6.797
6.748
6.733
6.647
2.290
CF3
Me
N3
14f
2.07
0.99
1
1
3.18
2.04
1.04
1.03
1
ppm 11
10
9
8
7
6
5
4
3
2
1
0

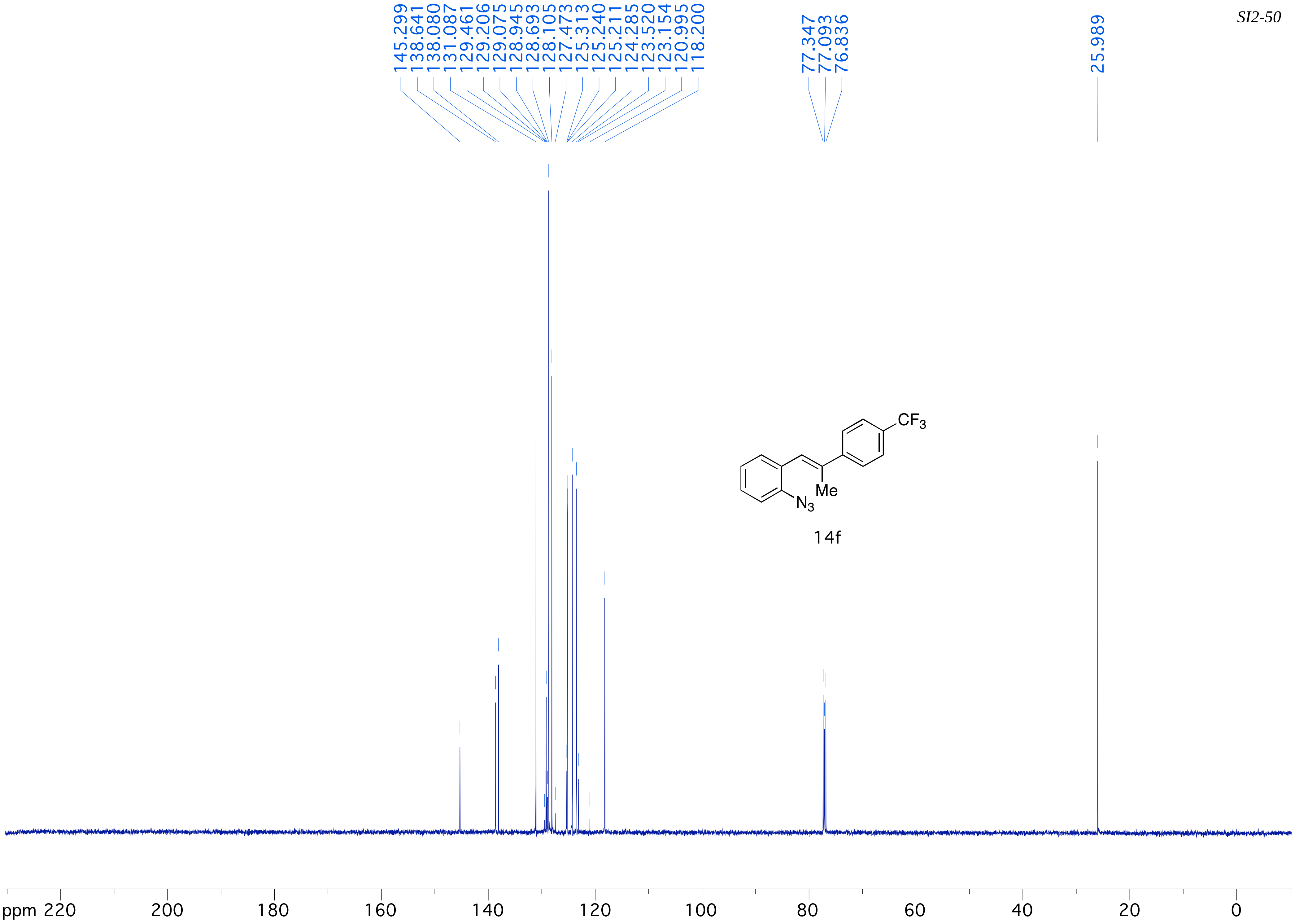
145.299
138.641
138.080
131.087
129.461
129.206
129.075
128.945
128.693
128.105
127.473
125.313
125.240
125.211
124.285
123.520
123.154
120.995
118.200
77.347
77.093
76.836
25.989
CF3
Me
N3
14f
ppm 220
200
180
160
140
120
100
80
60
40
20
0

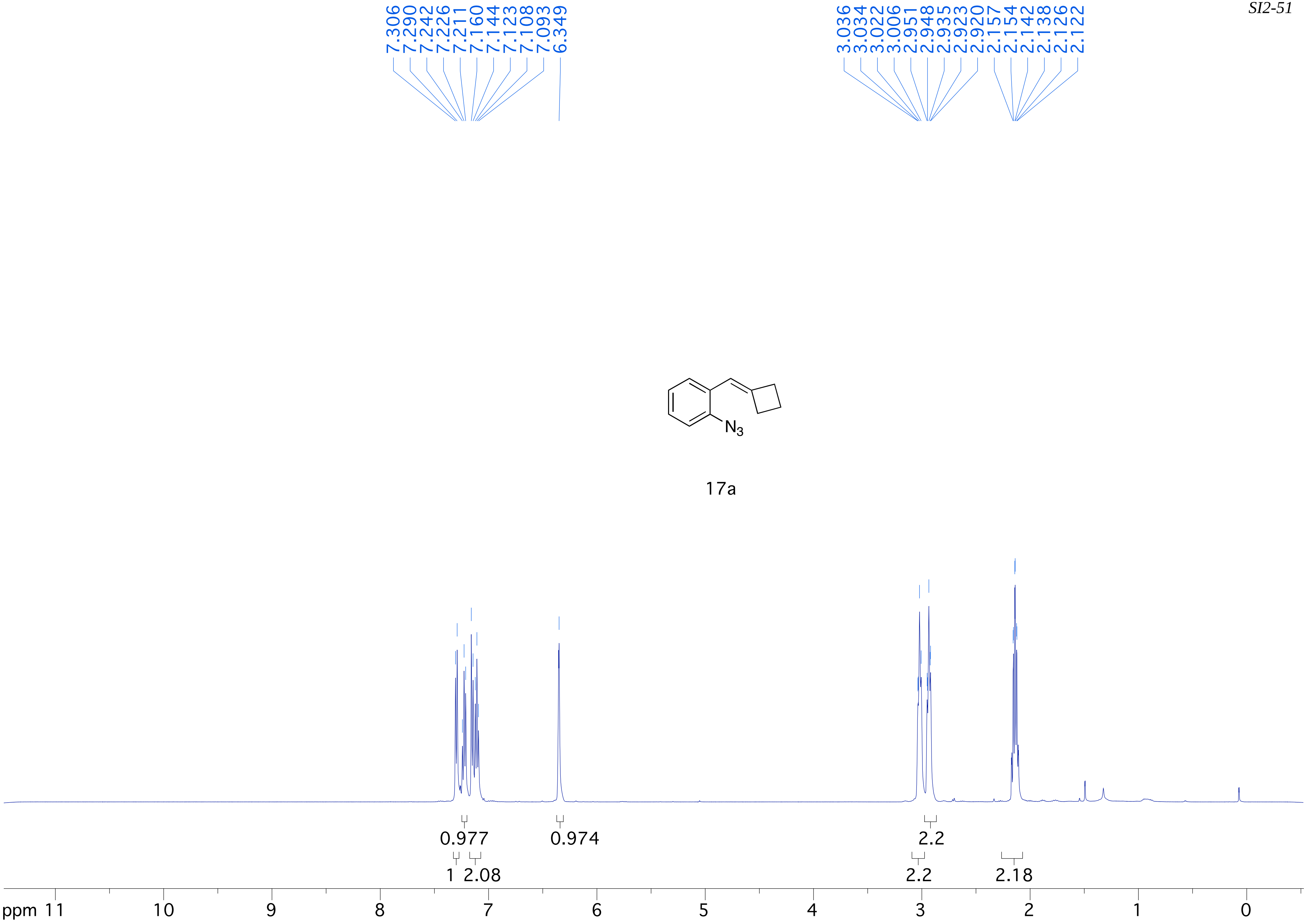
7.306
7.290
7.242
7.226
7.211
7.160
7.144
7.123
7.108
7.093
6.349
3.036
3.034
3.022
3.006
2.951
2.948
2.935
2.923
2.920
2.157
2.154
2.142
2.138
2.126
2.122
0.977
0.974
2.2
1
2.08
2.2
2.18
ppm 11
10
9
8
7
6
5
4
3
2
1
0
N3

17a

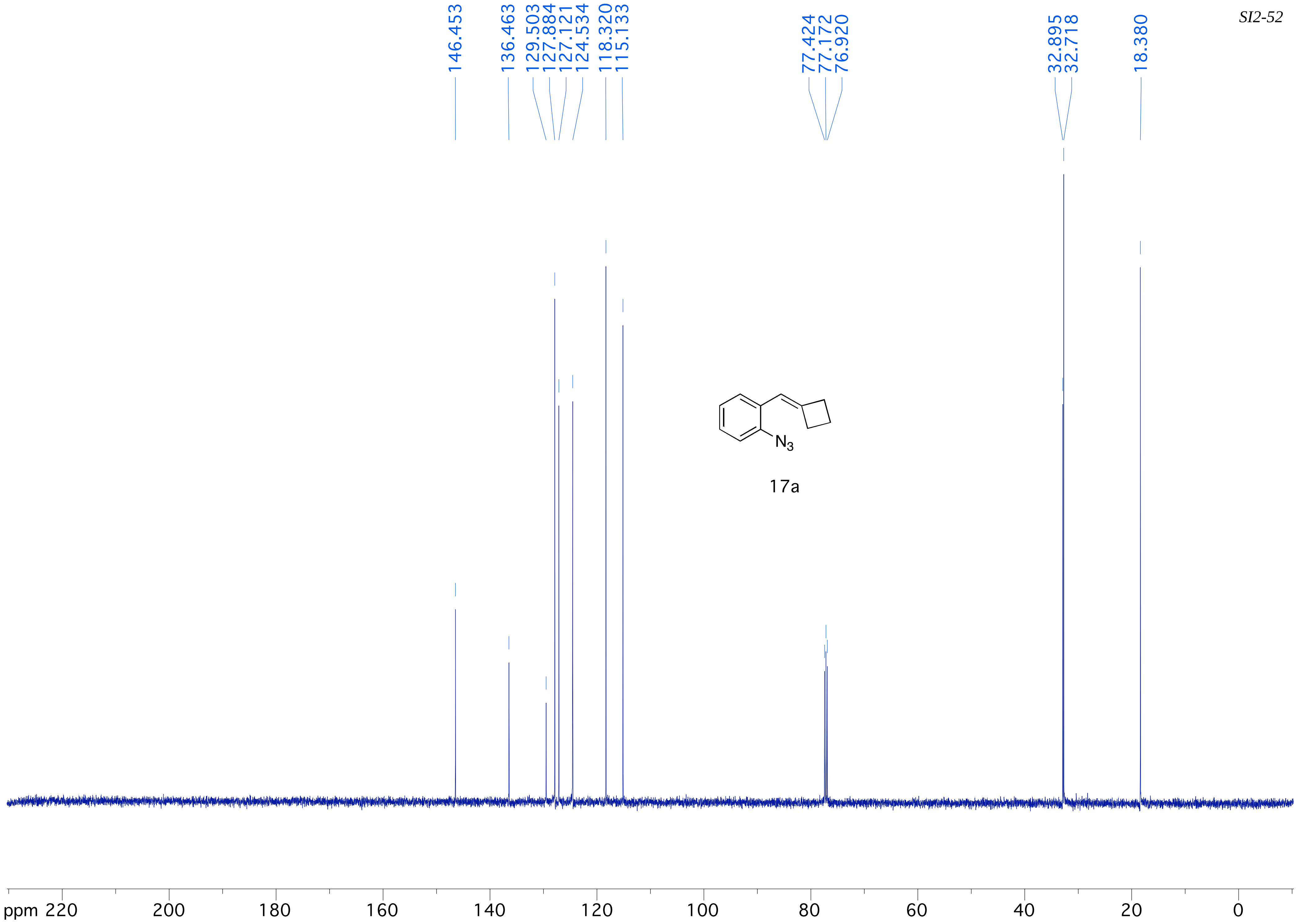
146.453
136.463
129.503
127.884
127.121
124.534
118.320
115.133
77.424
77.172
76.920
32.895
32.718
18.380
N3
17a
ppm 220 200 180 160 140 120 100 80 60 40 20 0

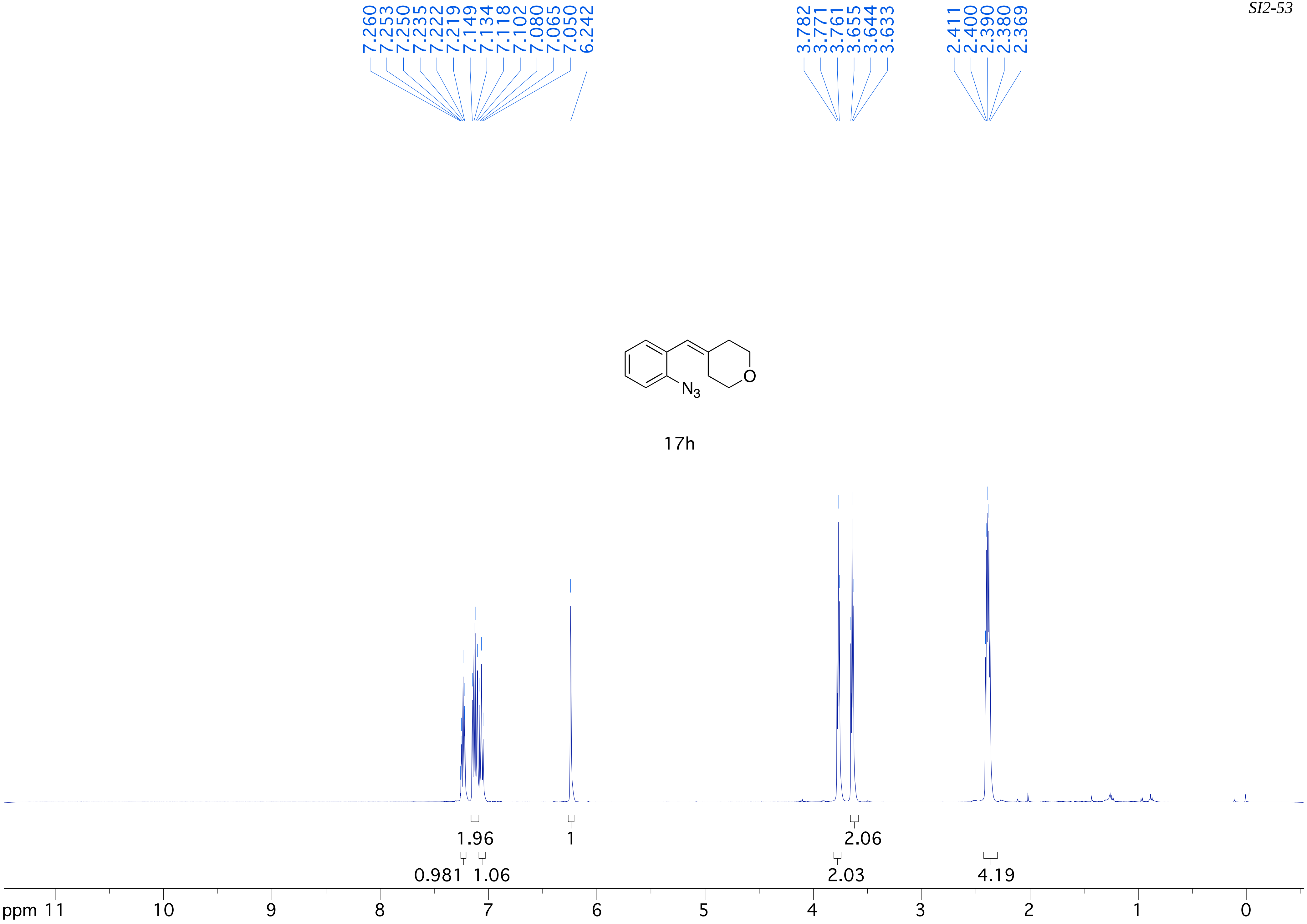
7.260
7.253
7.250
7.235
7.222
7.219
7.149
7.134
7.118
7.102
7.080
7.065
7.050
6.242
3.782
3.771
3.761
3.655
3.644
3.633
2.411
2.400
2.390
2.380
2.369
N3
O
17h
1.96
1
2.06
0.981
1.06
2.03
4.19
ppm 11
10
9
8
7
6
5
4
3
2
1
0

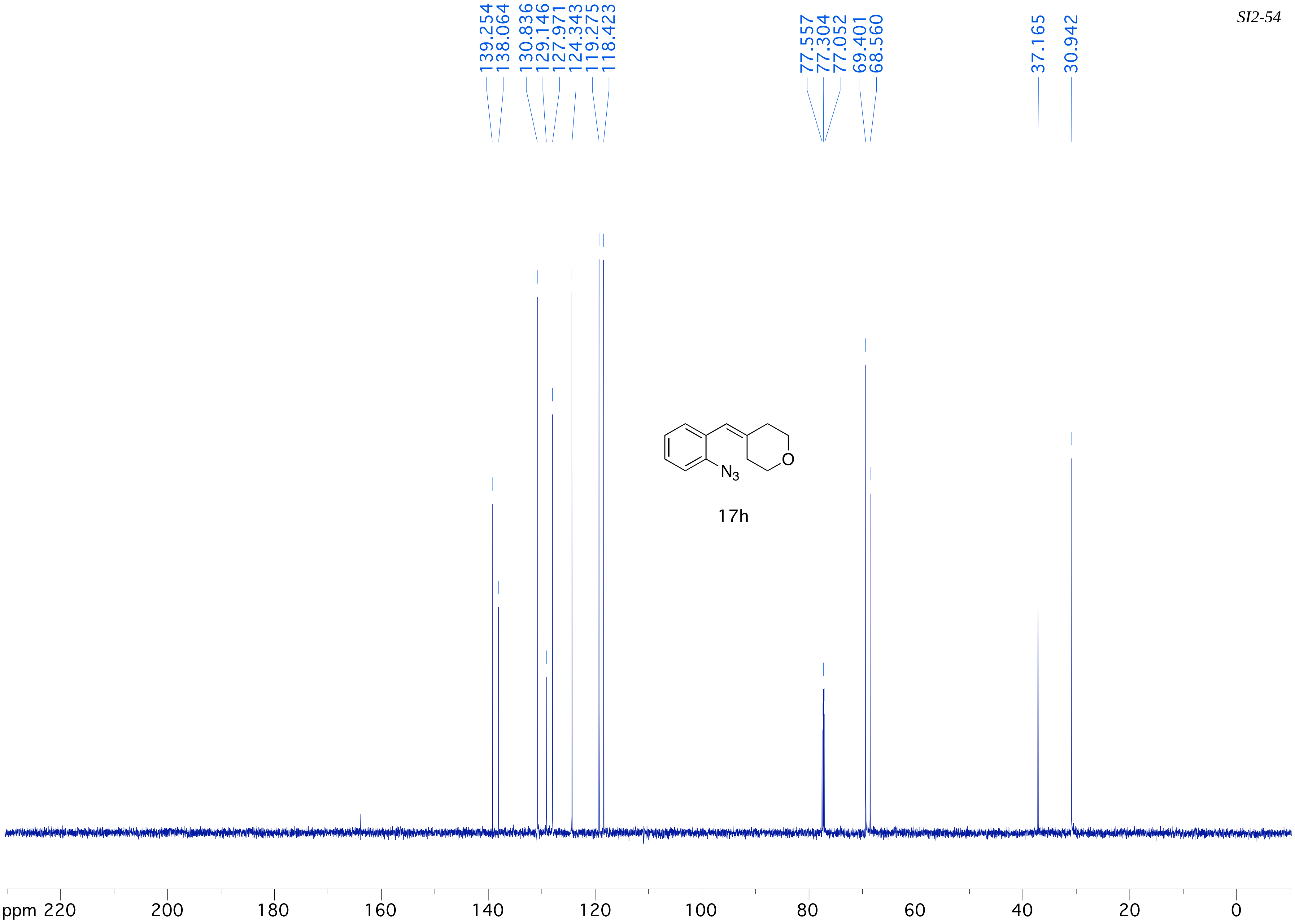
139.254
138.064
130.836
129.146
127.971
124.343
119.275
118.423
77.557
77.304
77.052
69.401
68.560
37.165
30.942
N_3
O
17h
ppm 220
200
180
160
140
120
100
80
60
40
20
0

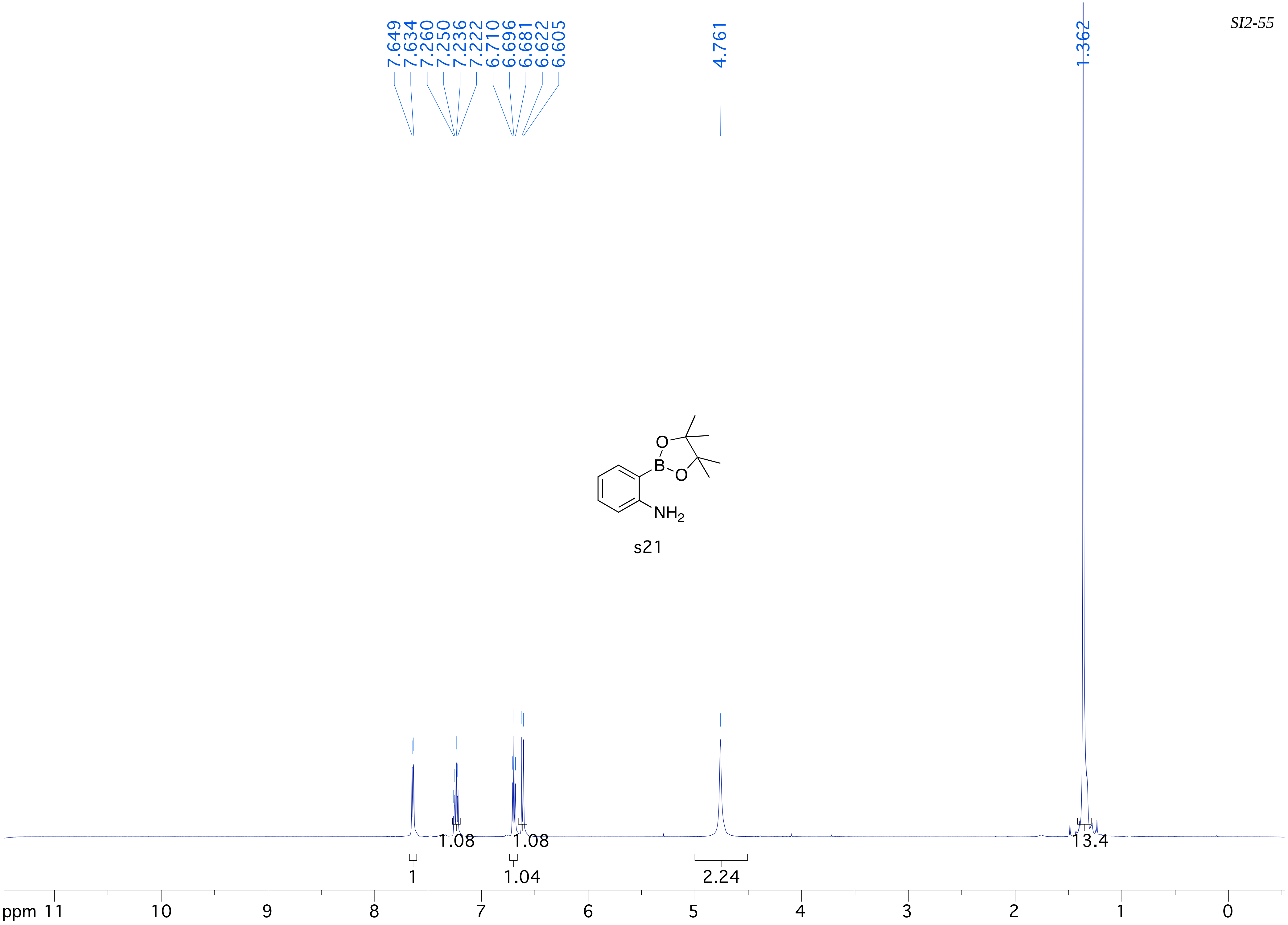
7.649
7.634
7.260
7.250
7.236
7.222
6.710
6.696
6.681
6.622
6.605
4.761
1.362
O
B
O
NH2
s21
1.08
1.08
13.4
1
1.04
2.24
ppm 11 10 9 8 7 6 5 4 3 2 1 0

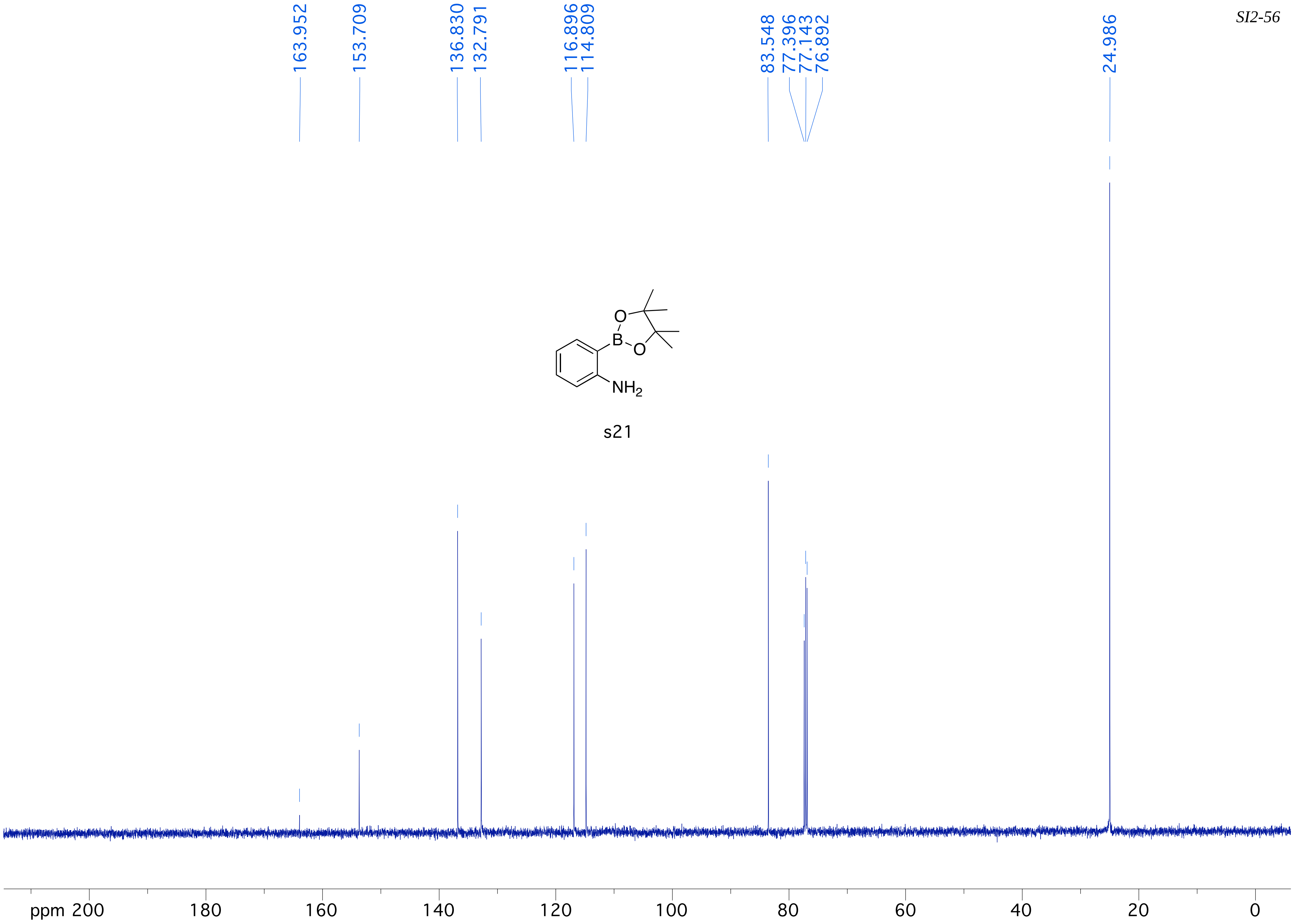
163.952
153.709
136.830
132.791
116.896
114.809
83.548
77.396
77.143
76.892
24.986
O
B
O
NH2
s21
ppm 200
180
160
140
120
100
80
60
40
20
0

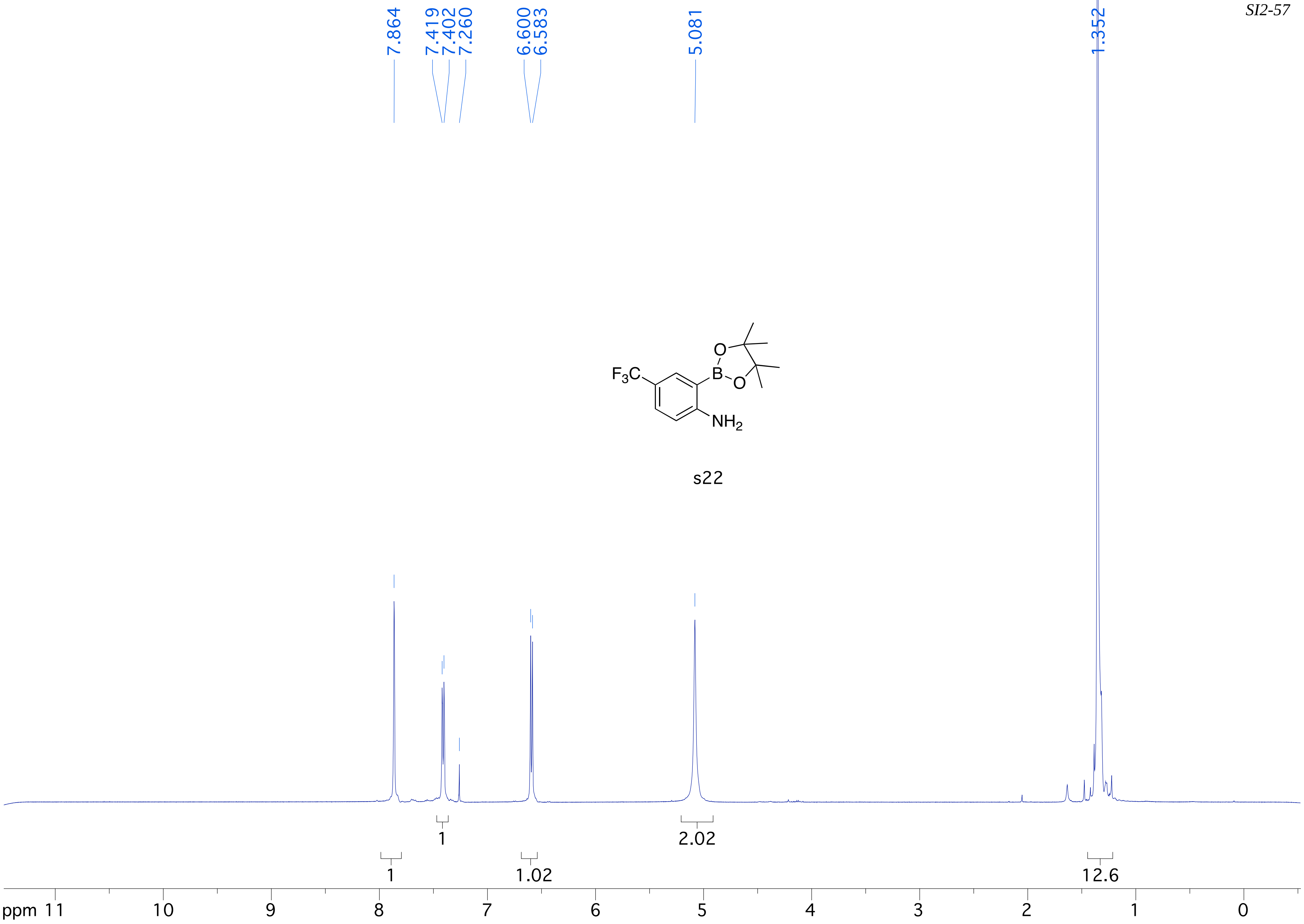
7.864
7.419
7.402
7.260
6.600
6.583
5.081
1.352
F3C
B
O
O
NH2
s22
1
2.02
1
1.02
12.6
ppm 11
10
9
8
7
6
5
4
3
2
1
0

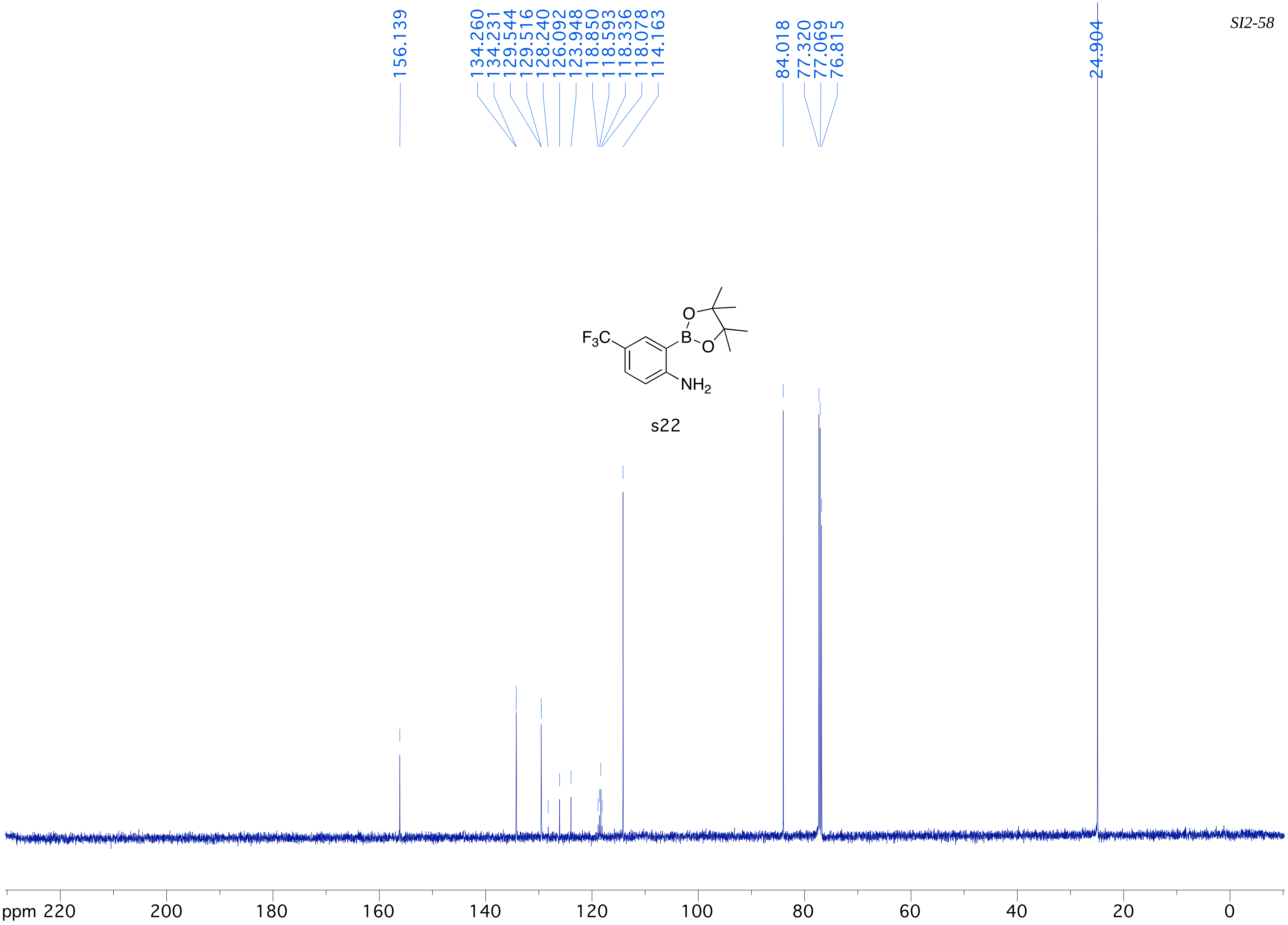
156.139
134.260
134.231
129.544
129.516
128.240
126.092
123.948
118.850
118.593
118.336
118.078
114.163
84.018
77.320
77.069
76.815
24.904
F_3C
B
O
O
NH_2
ppm 220
200
180
160
140
120
100
80
60
40
20
0

s22

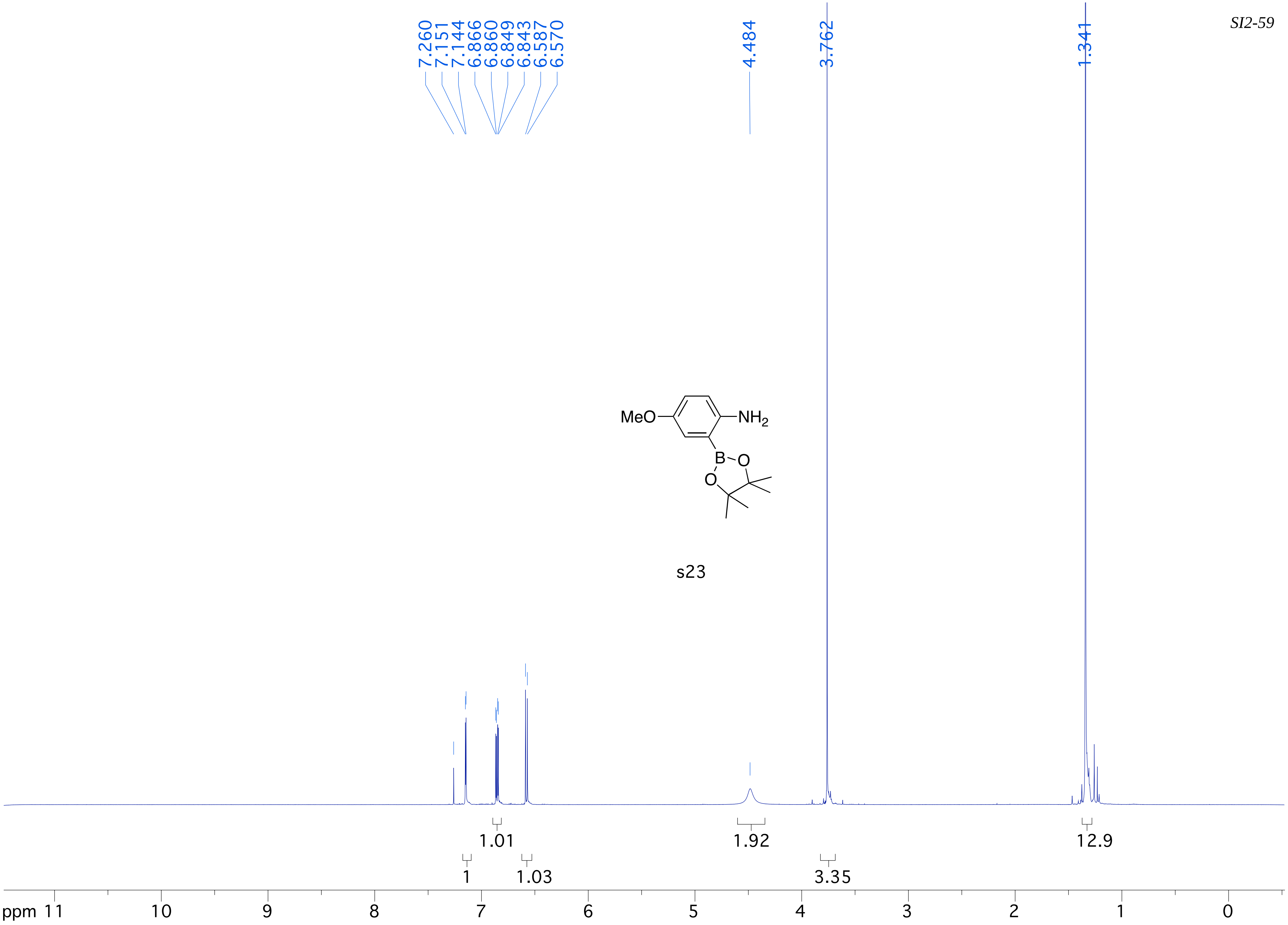
7.260
7.151
7.144
6.866
6.860
6.849
6.843
6.587
6.570
4.484
3.762
1.341
MeO
NH2
B
O
O
s23
1.01
1.92
12.9
1
1.03
3.35
ppm 11
10
9
8
7
6
5
4
3
2
1
0

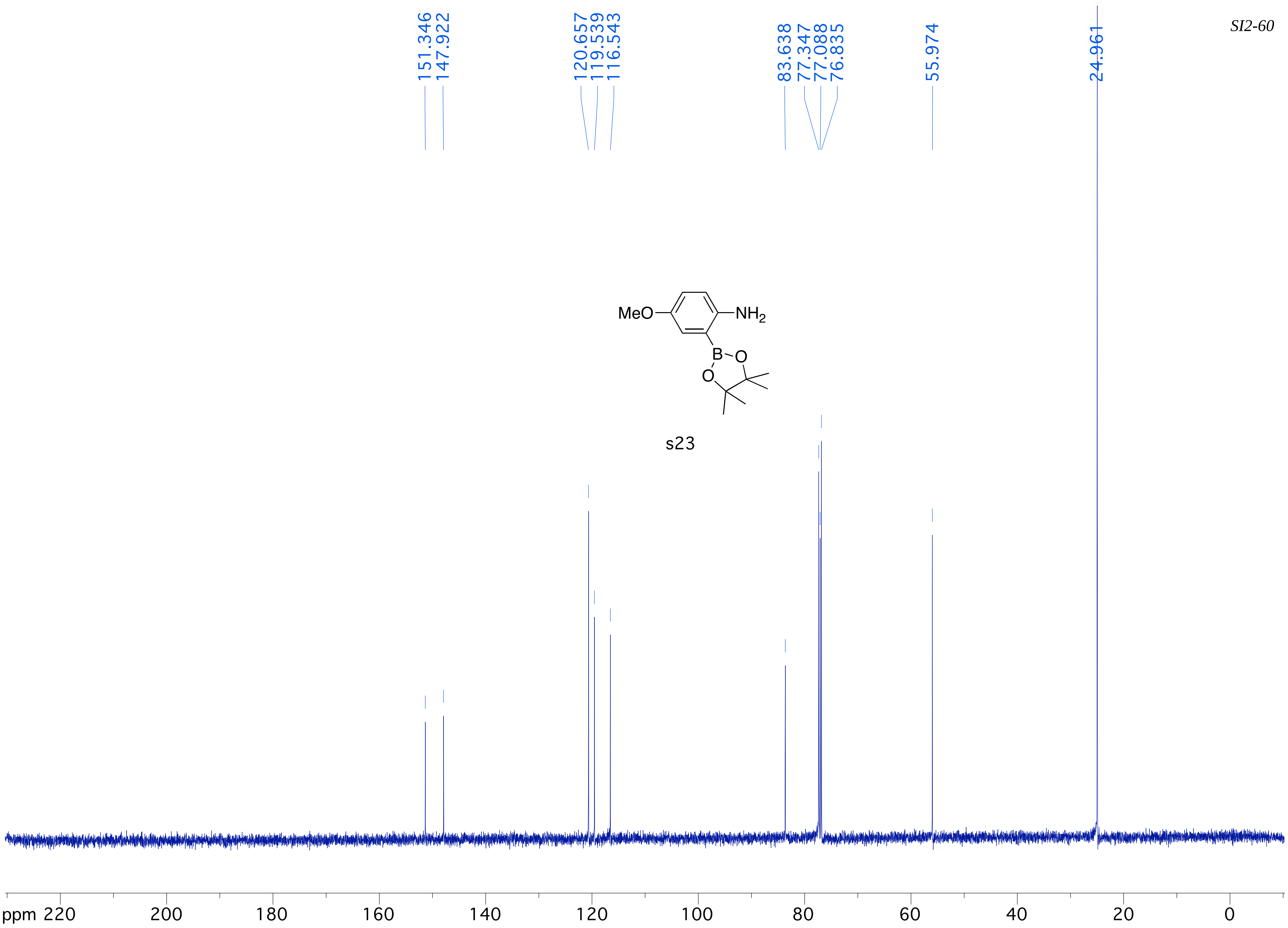
151.346
147.922
120.657
119.539
116.543
83.638
77.347
77.088
76.835
55.974
24.961
MeO
NH2
B
O
O
s23
ppm 220
200
180
160
140
120
100
80
60
40
20
0

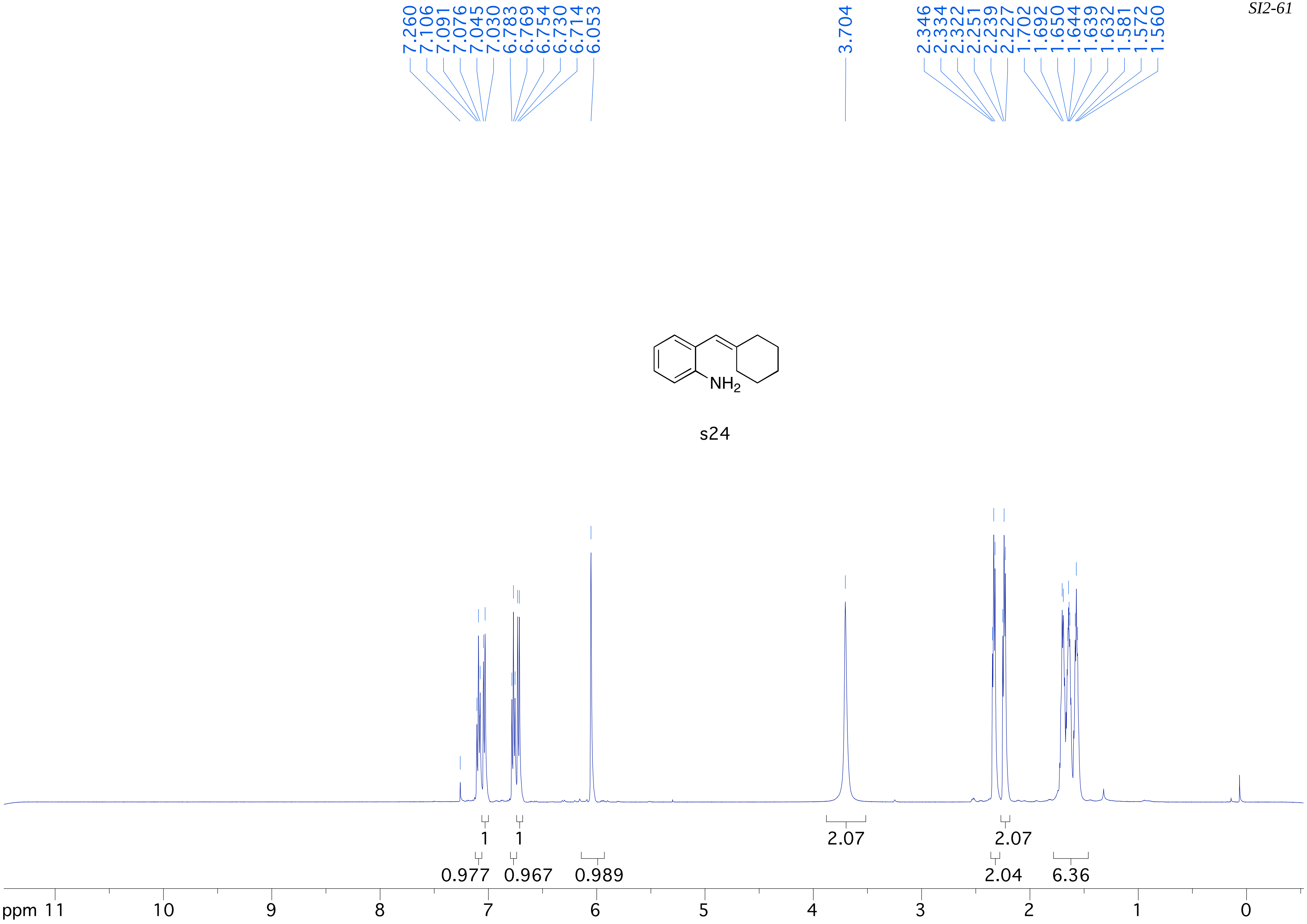
7.260
7.106
7.091
7.076
7.045
7.030
6.783
6.769
6.754
6.730
6.714
6.053
3.704
2.346
2.334
2.322
2.251
2.239
2.227
1.702
1.692
1.650
1.644
1.639
1.632
1.581
1.572
1.560
NH2
s24
1
1
2.07
2.07
0.977
0.967
0.989
2.04
6.36
ppm 11
10
9
8
7
6
5
4
3
2
1
0

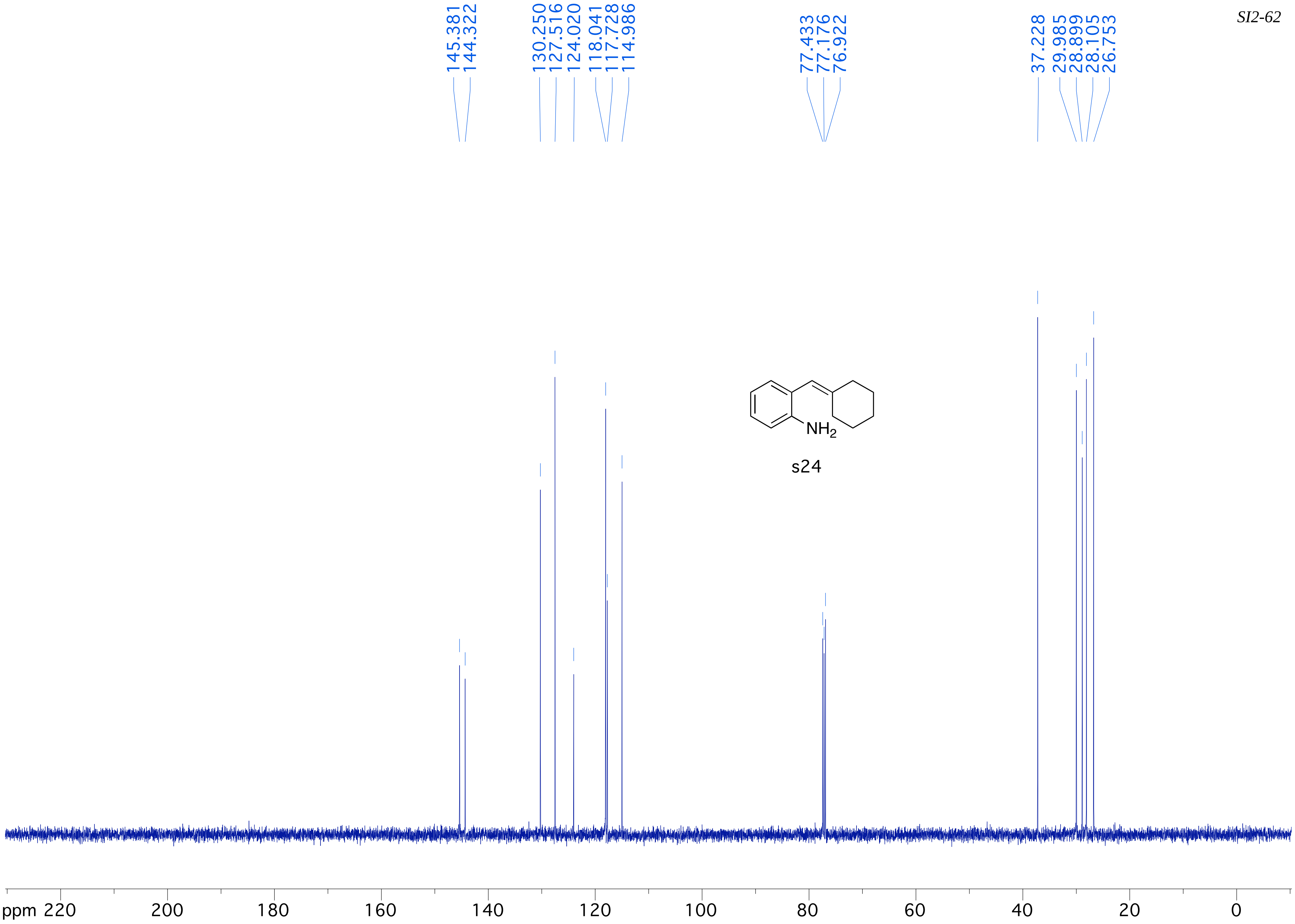
145.381
144.322
130.250
127.516
124.020
118.041
117.728
114.986
77.433
77.176
76.922
37.228
29.985
28.899
28.105
26.753
NH2
s24
ppm 220
200
180
160
140
120
100
80
60
40
20
0

SI2-63

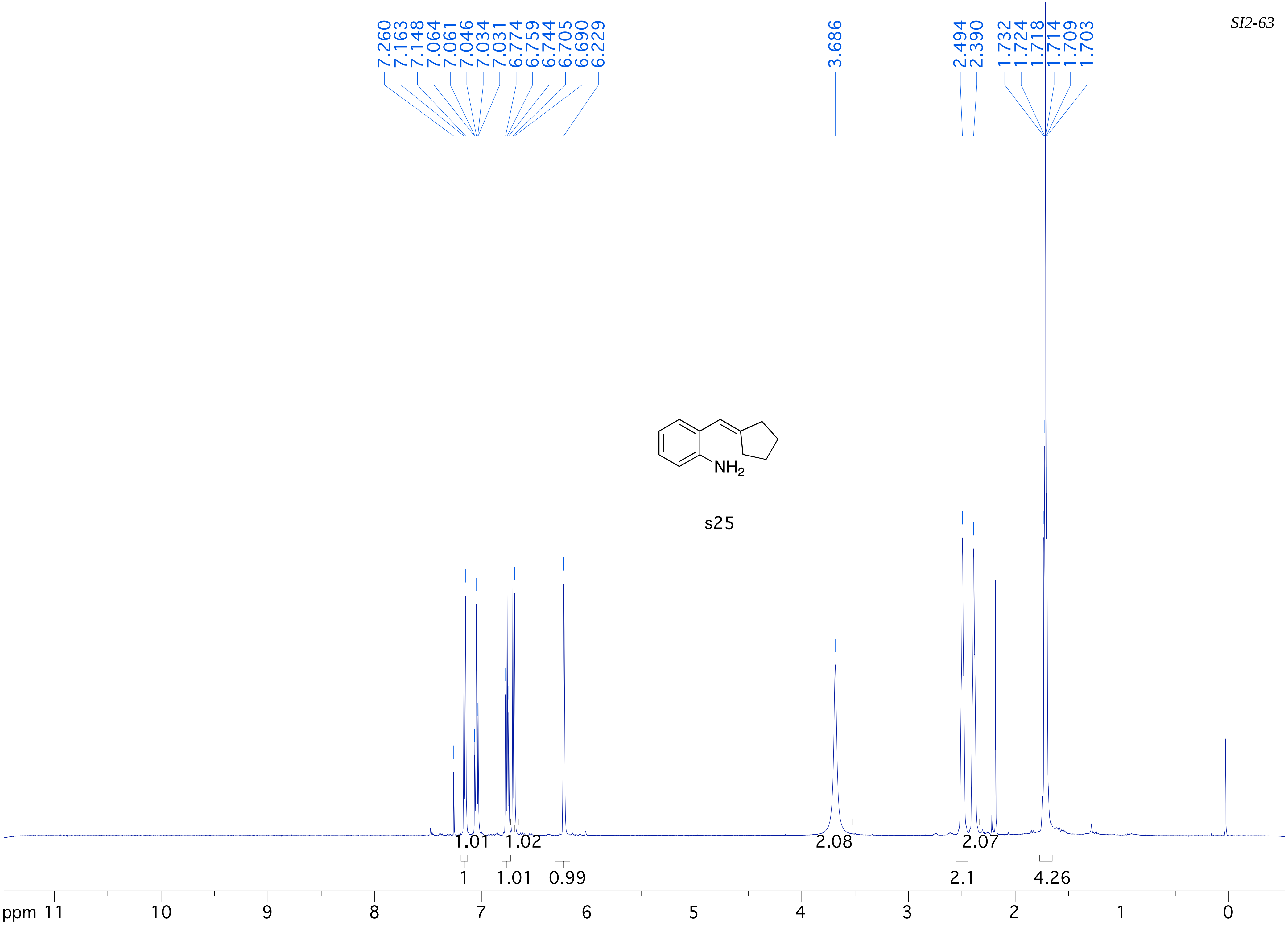
7.260
7.163
7.148
7.064
7.061
7.046
7.034
7.031
6.774
6.759
6.744
6.705
6.690
6.229
3.686
2.494
2.390
1.732
1.724
1.718
1.714
1.709
1.703
NH_2
1.01
1.02
2.08
2.07
1
1.01
0.99
2.1
4.26
ppm 11
10
9
8
7
6
5
4
3
2
1
0

s25

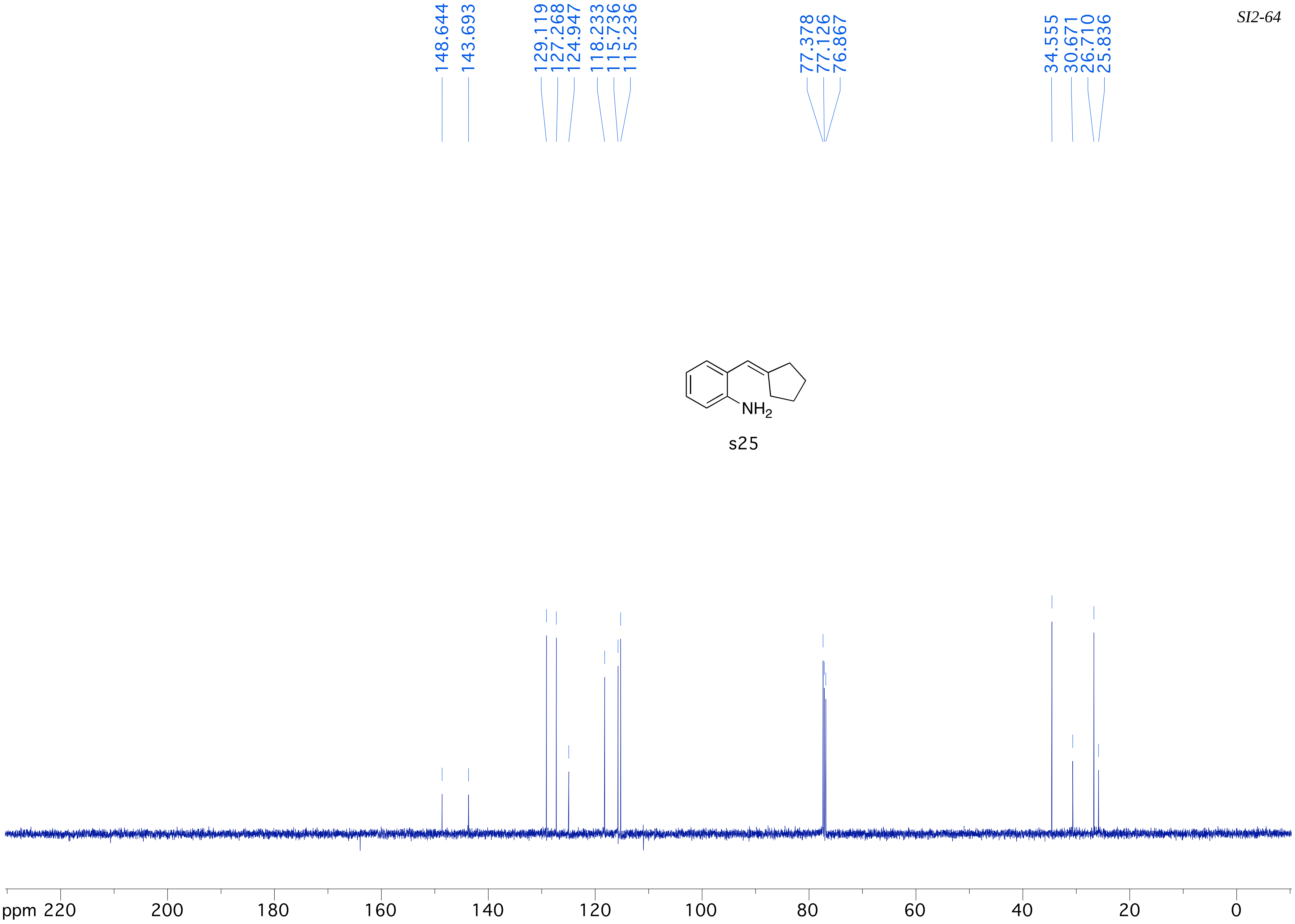
148.644
143.693
129.119
127.268
124.947
118.233
115.736
115.236
77.378
77.126
76.867
34.555
30.671
26.710
25.836
NH2
s25
ppm 220
200
180
160
140
120
100
80
60
40
20
0

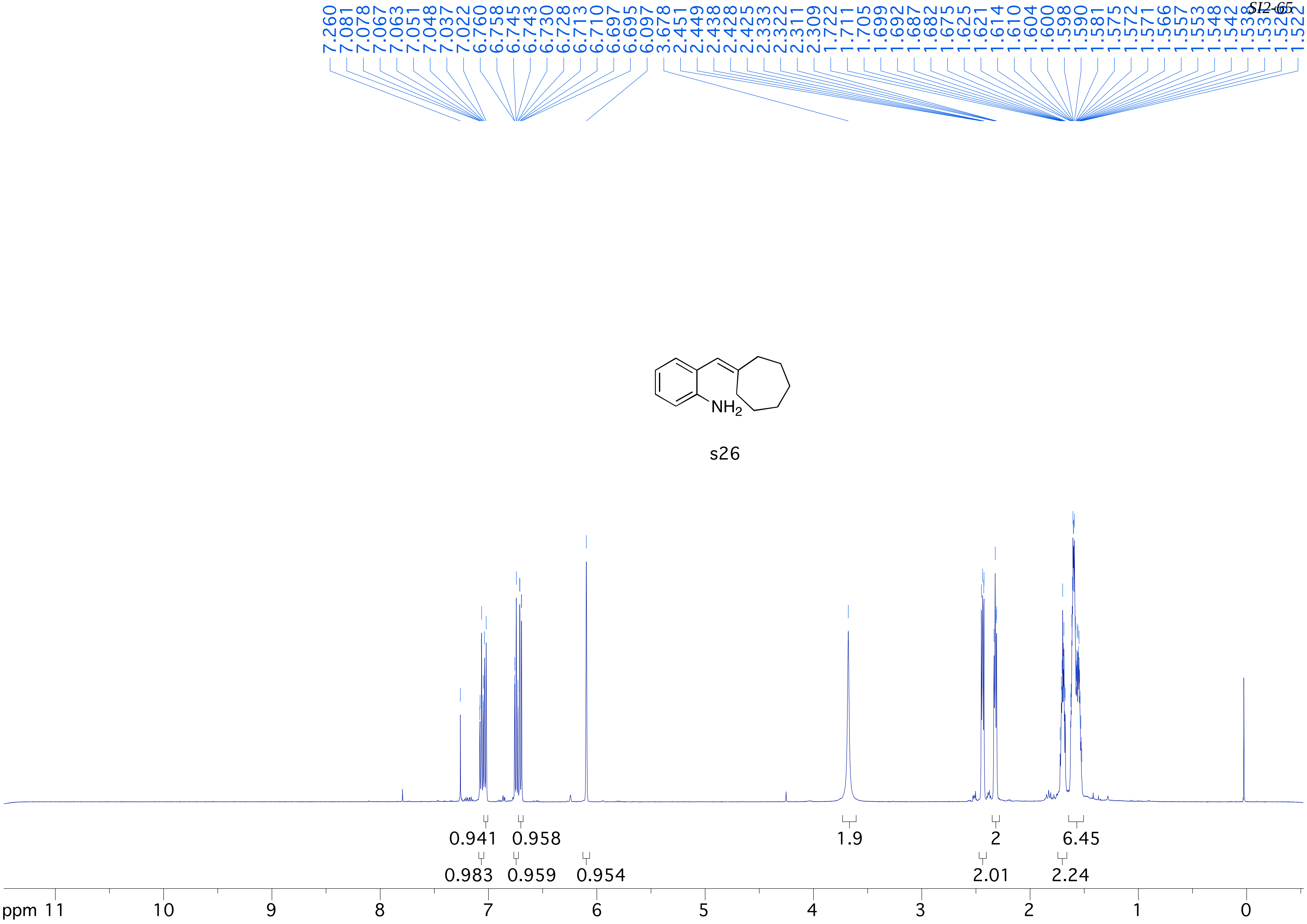
7.260
7.081
7.078
7.067
7.063
7.051
7.048
7.037
7.022
6.760
6.758
6.745
6.743
6.730
6.728
6.713
6.710
6.697
6.695
6.097
3.678
2.451
2.449
2.438
2.428
2.425
2.333
2.322
2.311
2.309
1.722
1.711
1.705
1.699
1.692
1.687
1.682
1.675
1.625
1.621
1.614
1.610
1.604
1.600
1.598
1.590
1.581
1.575
1.572
1.571
1.566
1.557
1.553
1.548
1.542
1.538
1.531
1.525
1.522
0.941
0.958
1.9
2
6.45
0.983
0.959
0.954
2.01
2.24
ppm 11
10
9
8
7
6
5
4
3
2
1
0
NH2

s26

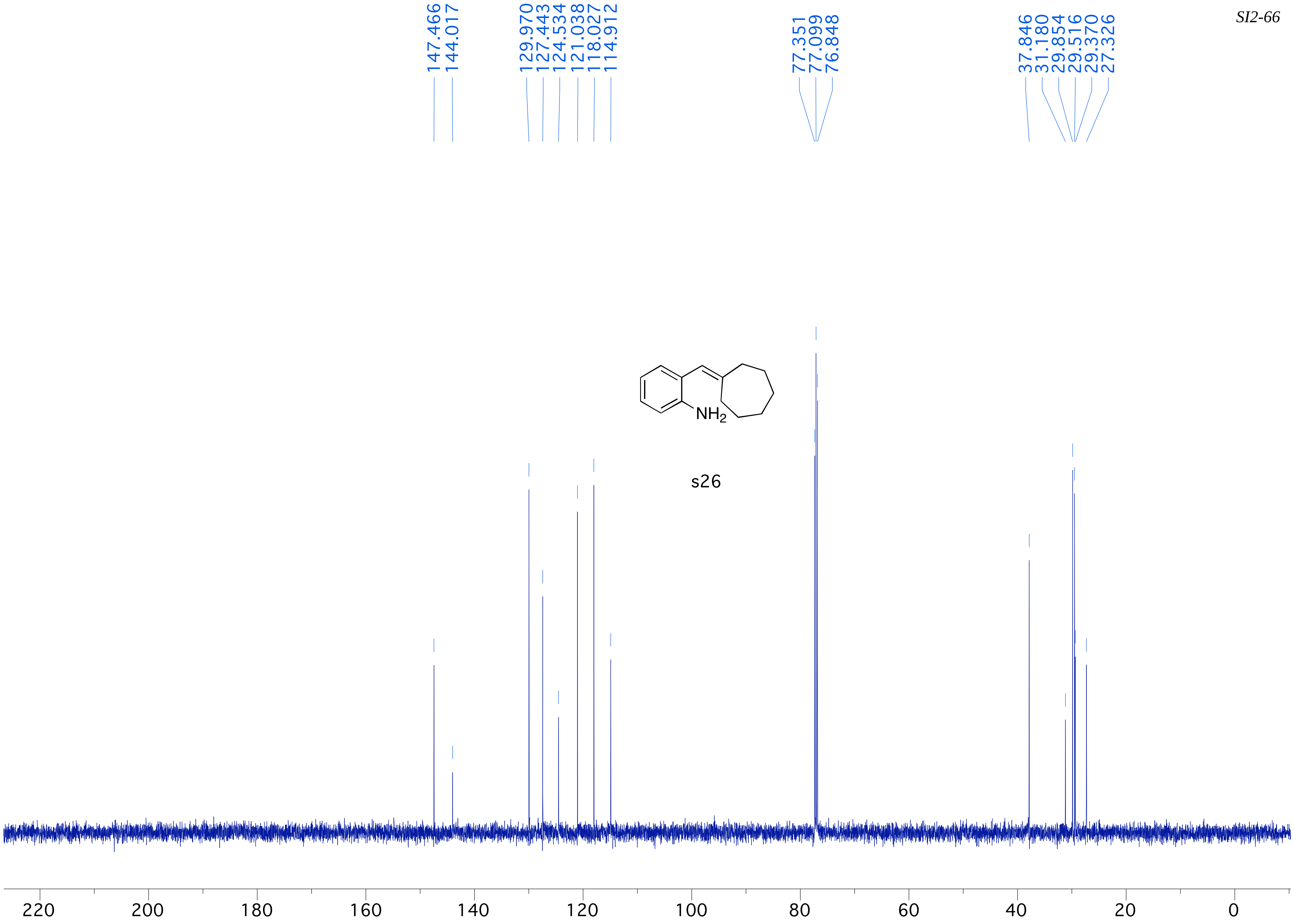
147.466
144.017
129.970
127.443
124.534
121.038
118.027
114.912
77.351
77.099
76.848
37.846
31.180
29.854
29.516
29.370
27.326
NH_2
s26
220
200
180
160
140
120
100
80
60
40
20
0

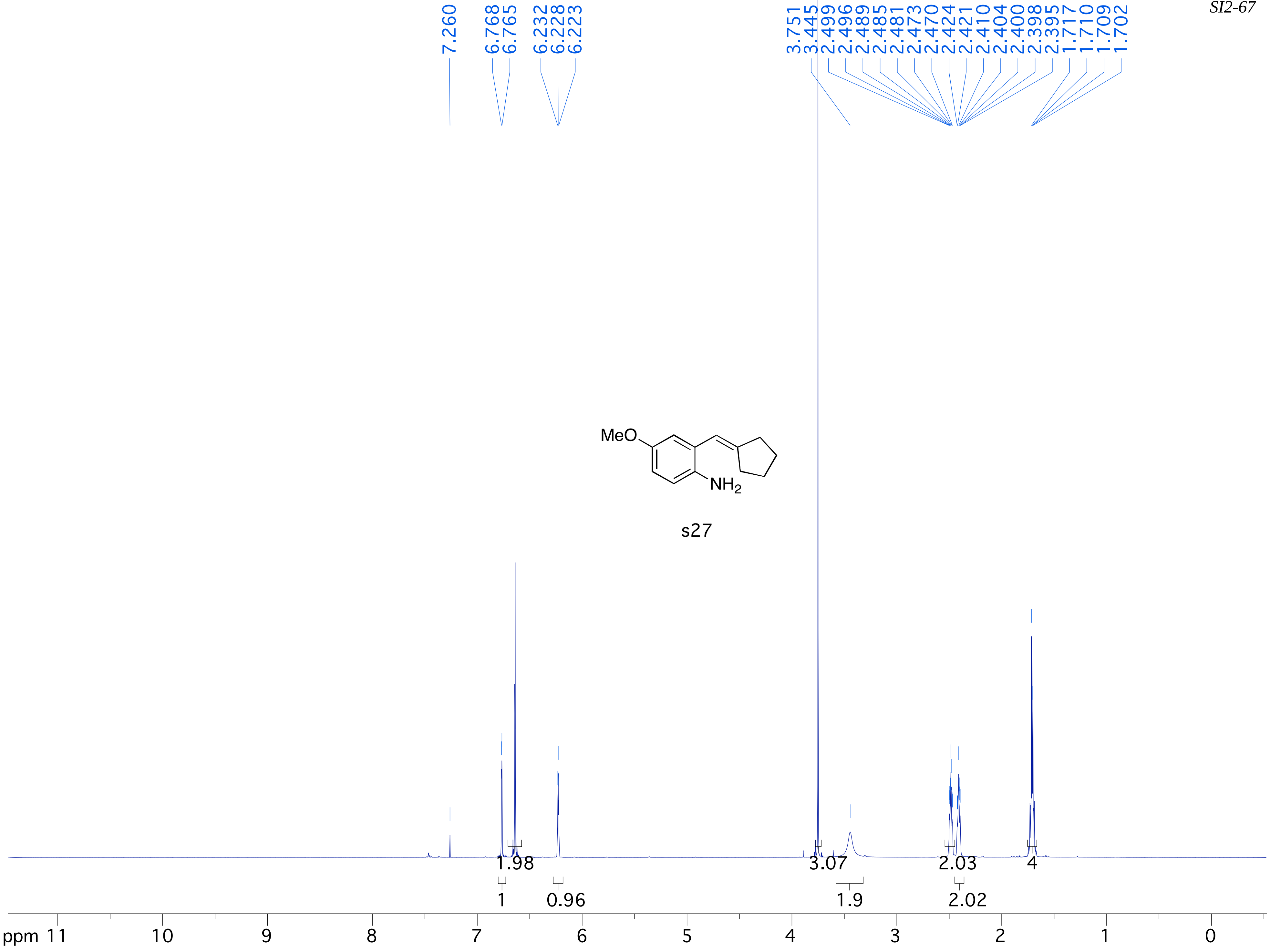
7.260
6.768
6.765
6.232
6.228
6.223
3.751
3.445
2.499
2.496
2.489
2.485
2.481
2.473
2.470
2.424
2.421
2.410
2.404
2.400
2.398
2.395
1.717
1.710
1.709
1.702
MeO
NH2
s27
1.98
1
0.96
3.07
1.9
2.03
2.02
4
ppm 11
10
9
8
7
6
5
4
3
2
1
0

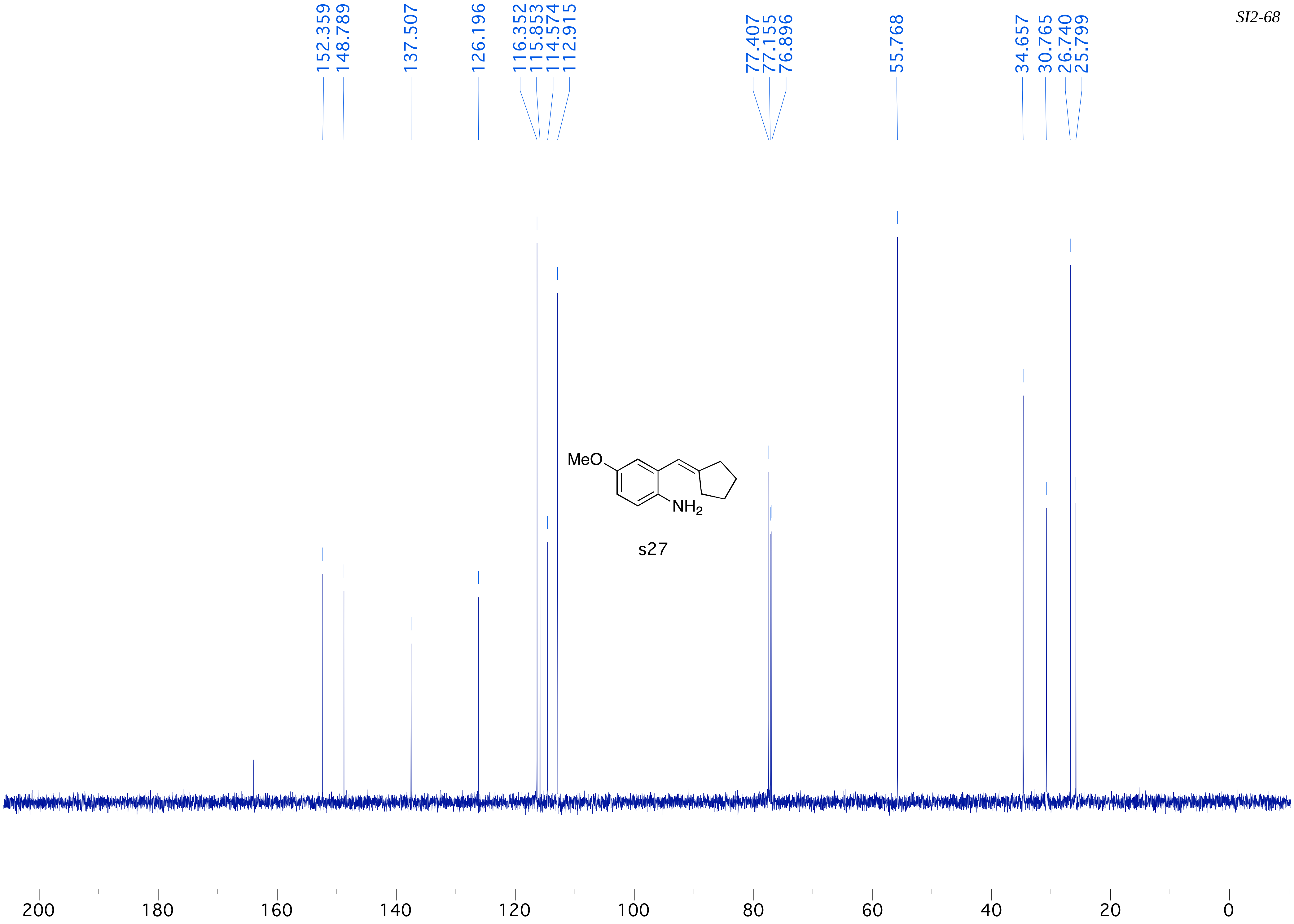
152.359
148.789
137.507
126.196
116.352
115.853
114.574
112.915
77.407
77.155
76.896
55.768
34.657
30.765
26.740
25.799
MeO
NH2
s27
200
180
160
140
120
100
80
60
40
20
0

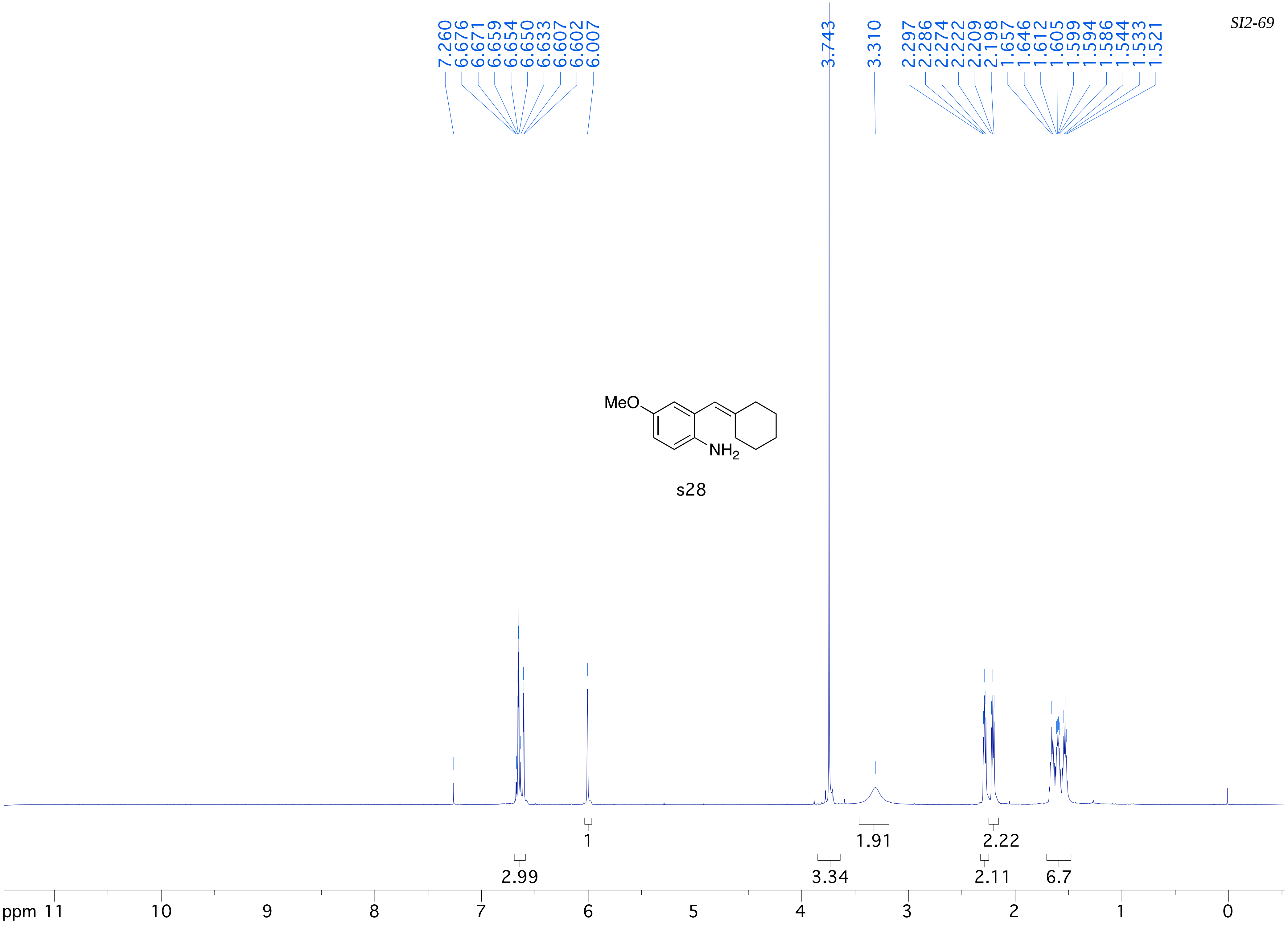
7.260
6.676
6.671
6.659
6.654
6.650
6.633
6.607
6.602
6.007
3.743
3.310
2.297
2.286
2.274
2.222
2.209
2.198
1.657
1.646
1.612
1.605
1.599
1.594
1.586
1.544
1.533
1.521
MeO
NH2
s28
1
1.91
2.22
2.99
3.34
2.11
6.7
ppm 11
10
9
8
7
6
5
4
3
2
1
0

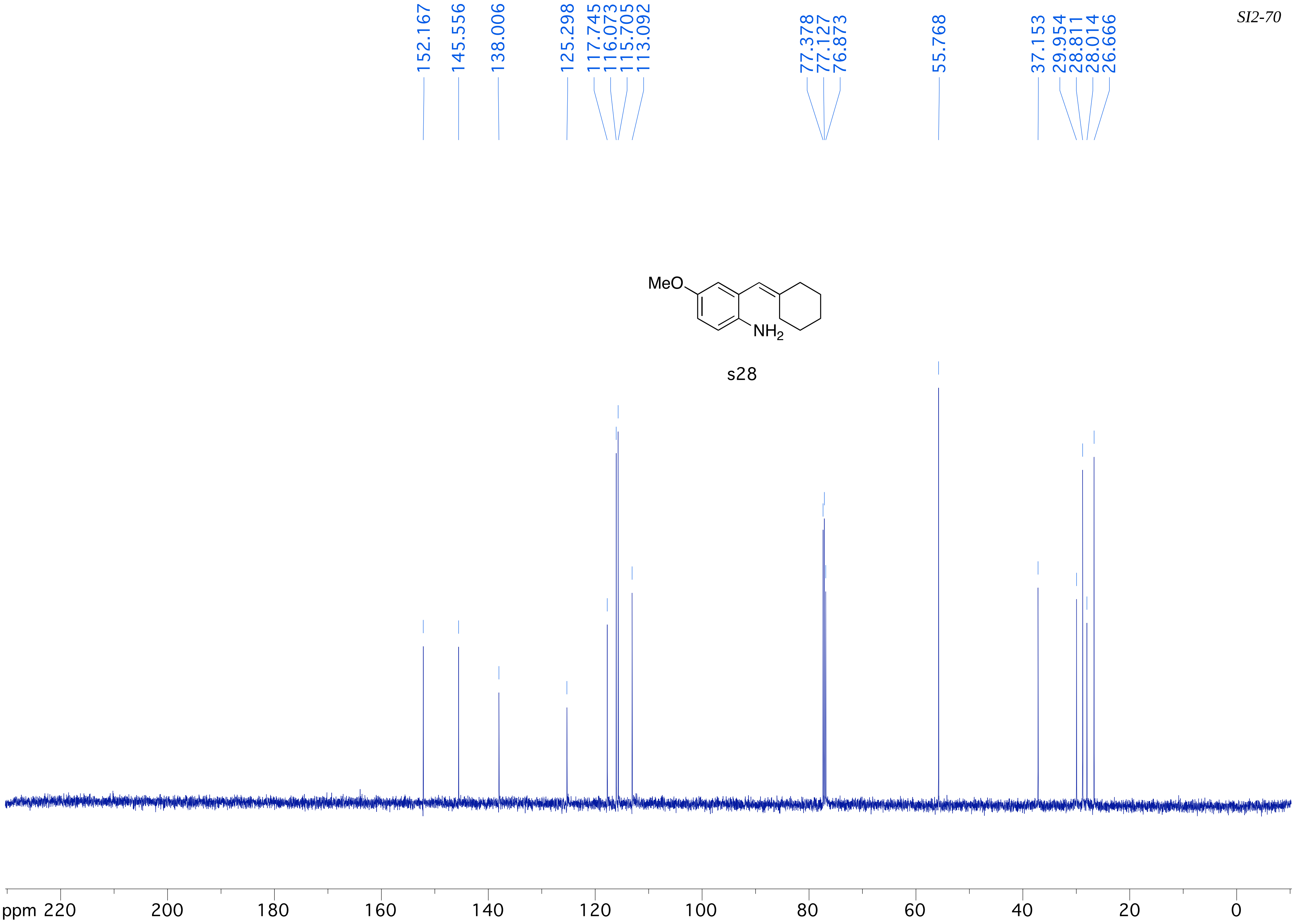
152.167
145.556
138.006
125.298
117.745
116.073
115.705
113.092
77.378
77.127
76.873
55.768
37.153
29.954
28.811
28.014
26.666
MeO
NH2
s28
ppm 220
200
180
160
140
120
100
80
60
40
20
0

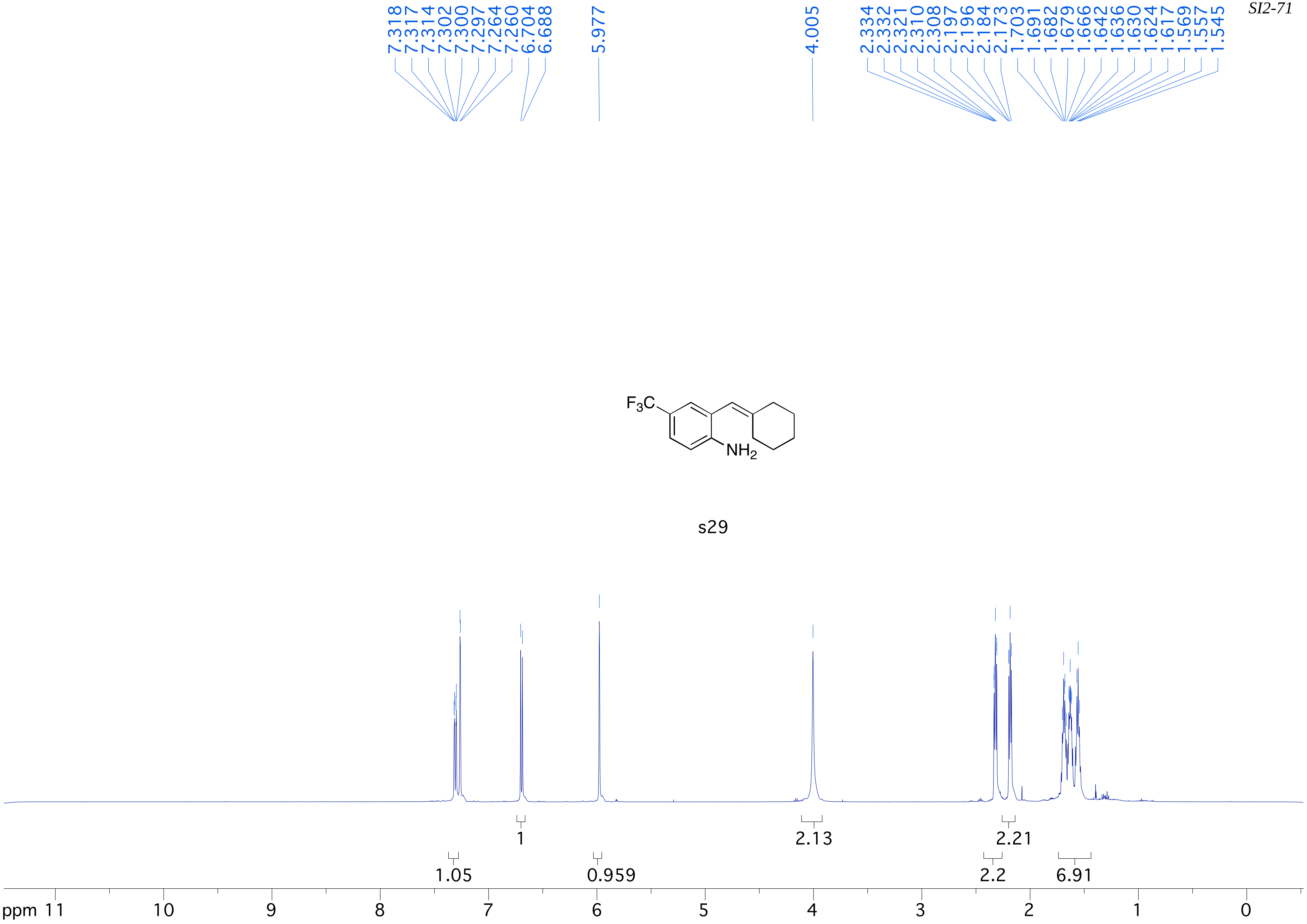
7.318
7.317
7.314
7.302
7.300
7.297
7.264
7.260
6.704
6.688
5.977
4.005
2.334
2.332
2.321
2.310
2.308
2.197
2.196
2.184
2.173
1.703
1.691
1.682
1.679
1.666
1.642
1.636
1.630
1.624
1.617
1.569
1.557
1.545
F3C
NH2
s29
1
2.13
2.21
1.05
0.959
2.2
6.91
ppm 11
10
9
8
7
6
5
4
3
2
1
0

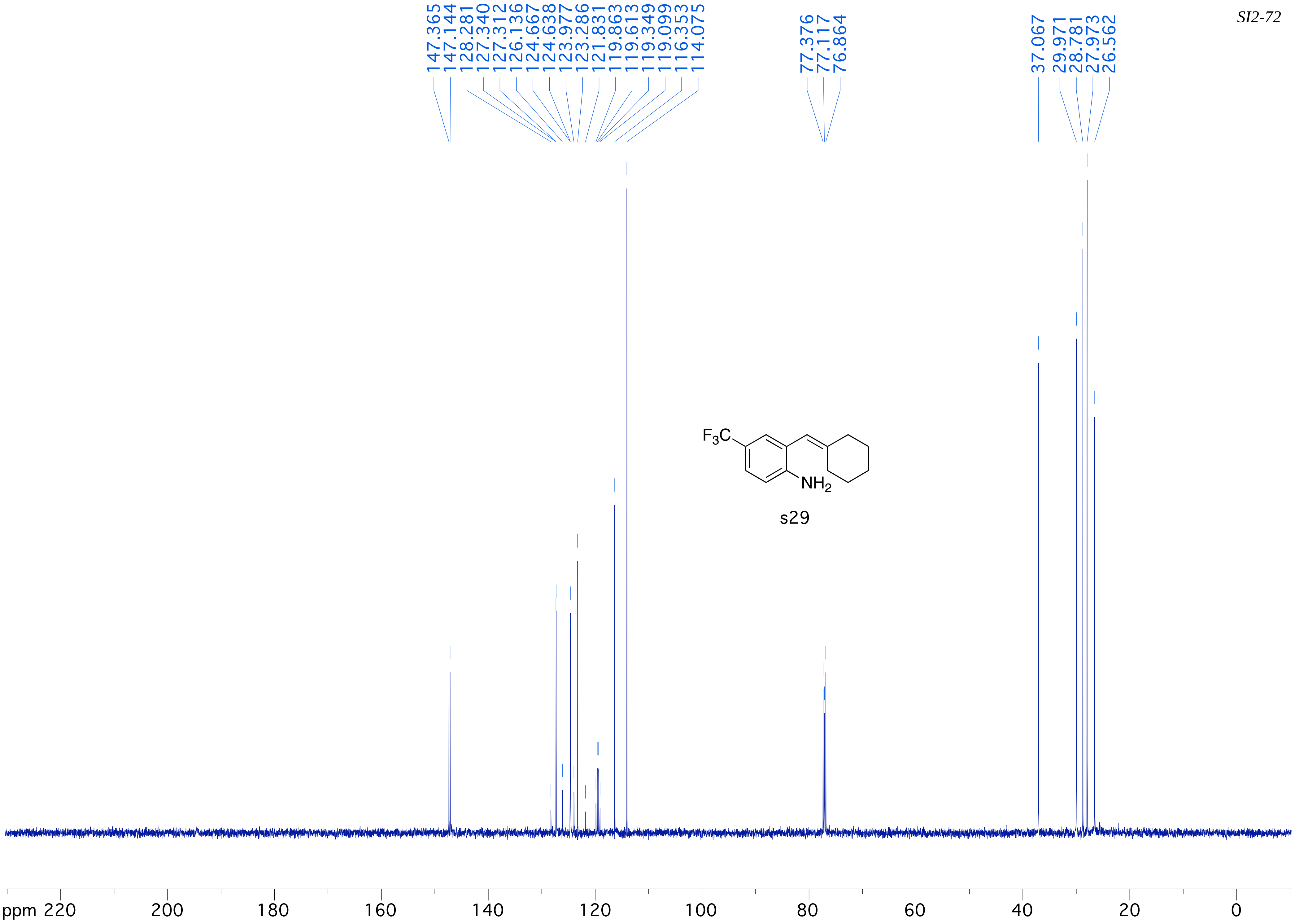
147.365
147.144
128.281
127.340
127.312
126.136
124.667
124.638
123.977
123.286
121.831
119.863
119.613
119.349
119.099
116.353
114.075
77.376
77.117
76.864
37.067
29.971
28.781
27.973
26.562
F3C
NH2
s29
ppm 220
200
180
160
140
120
100
80
60
40
20
0

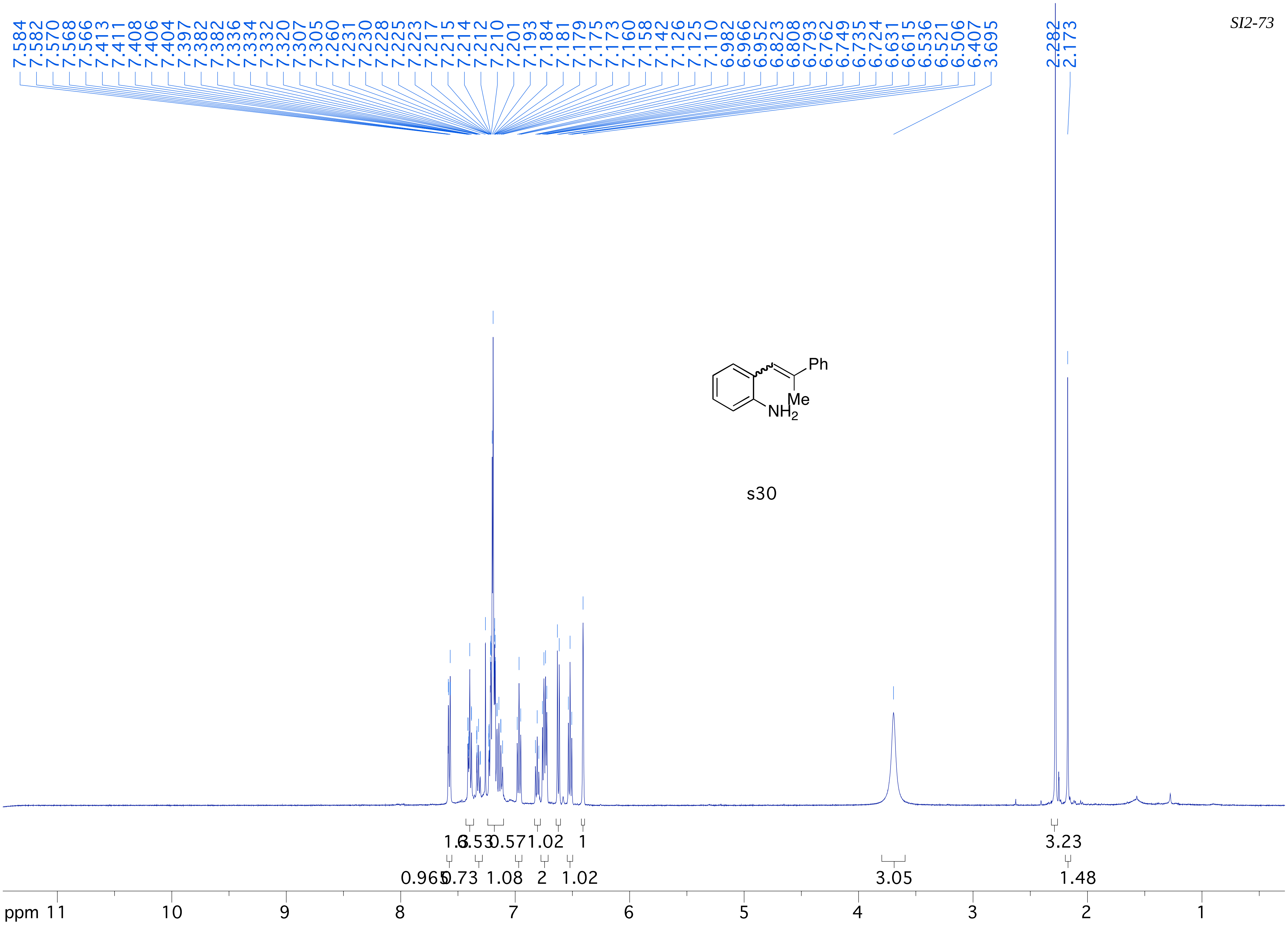
7.584 7.582 7.570 7.568 7.566 7.413 7.411 7.408 7.406 7.404 7.397 7.382 7.382 7.336 7.334 7.332 7.320 7.307 7.305 7.260 7.231 7.230 7.228 7.225 7.223 7.217 7.215 7.214 7.212 7.210 7.201 7.193 7.184 7.181 7.179 7.175 7.173 7.160 7.158 7.142 7.126 7.125 7.110 6.982 6.966 6.952 6.823 6.808 6.793 6.762 6.749 6.735 6.724 6.631 6.615 6.536 6.521 6.506 6.407 3.695
2.282 2.173
Ph
Me
NH2
s30
0.57
1.02
1
3.23
0.73
1.08
2
1.02
3.05
1.48
ppm 11 10 9 8 7 6 5 4 3 2 1

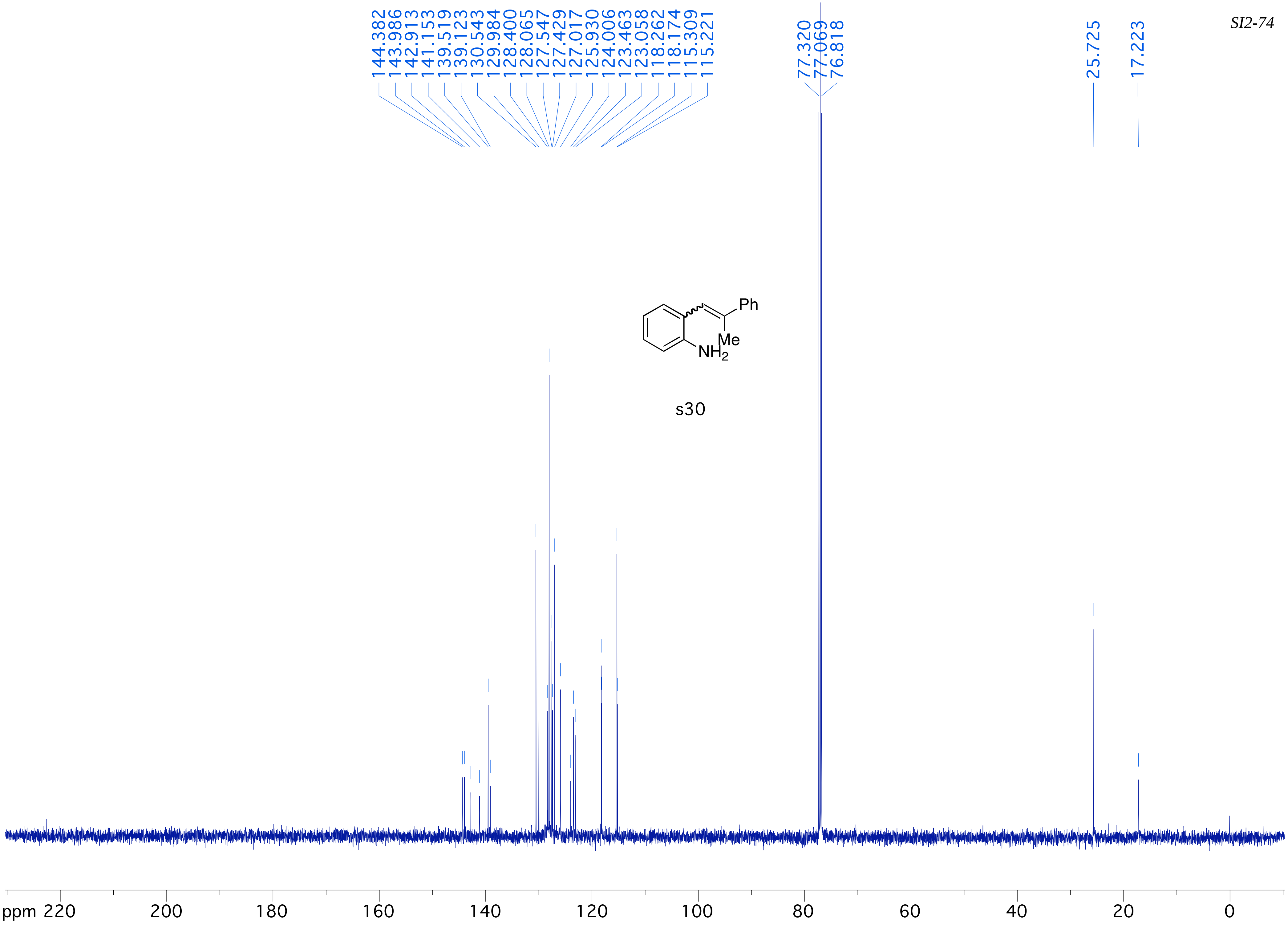
144.382
143.986
142.913
141.153
139.519
139.123
130.543
129.984
128.400
128.065
127.547
127.429
127.017
125.930
124.006
123.463
123.058
118.262
118.174
115.309
115.221
77.320
77.069
76.818
25.725
17.223
Ph
Me
NH2
s30
ppm 220
200
180
160
140
120
100
80
60
40
20
0

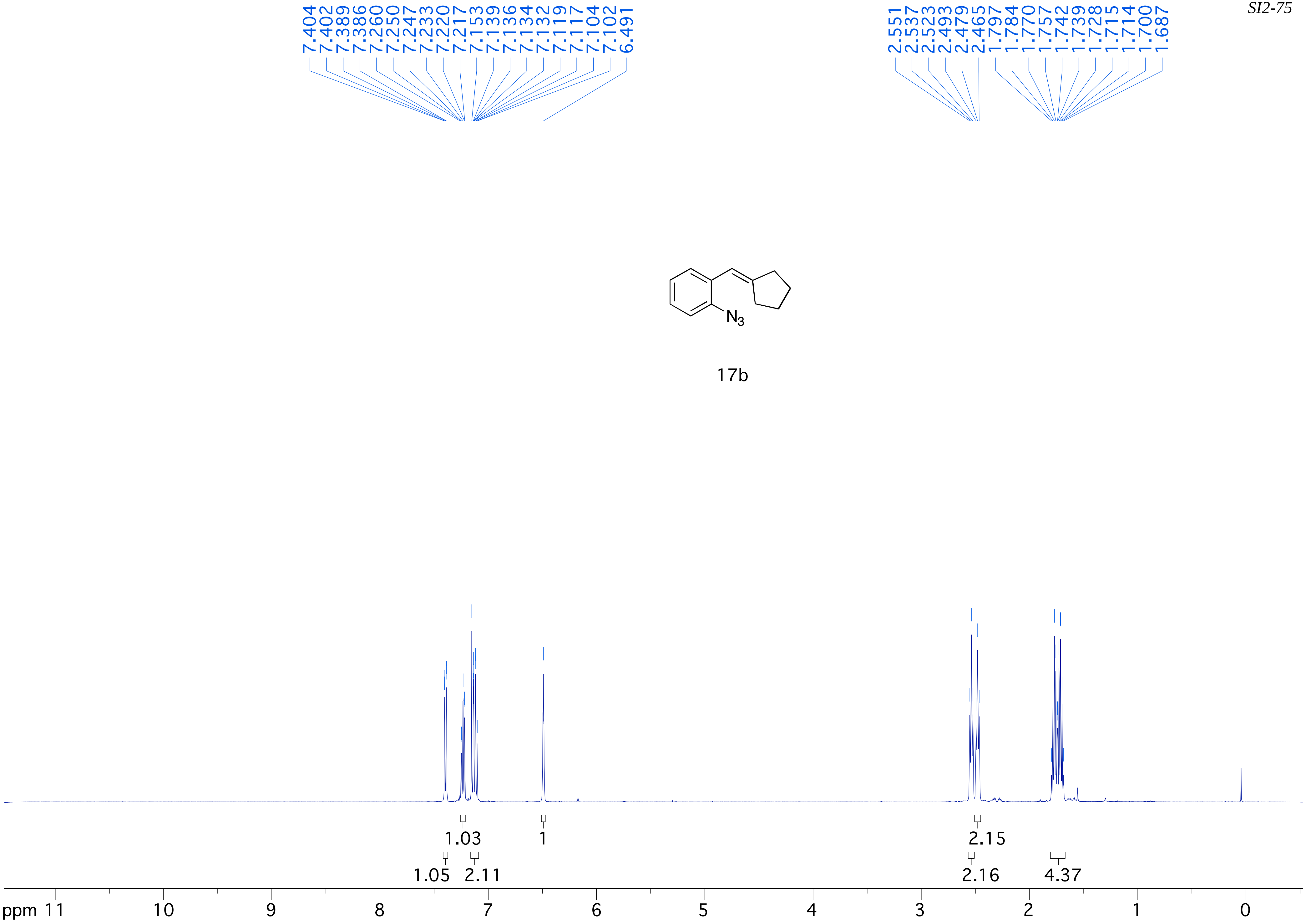
7.404
7.402
7.389
7.386
7.260
7.250
7.247
7.233
7.220
7.217
7.153
7.139
7.136
7.134
7.132
7.119
7.117
7.104
7.102
6.491
2.551
2.537
2.523
2.493
2.479
2.465
1.797
1.784
1.770
1.757
1.742
1.739
1.728
1.715
1.714
1.700
1.687
N3
17b
1.03
1
2.15
1.05
2.11
2.16
4.37
ppm 11
10
9
8
7
6
5
4
3
2
1
0

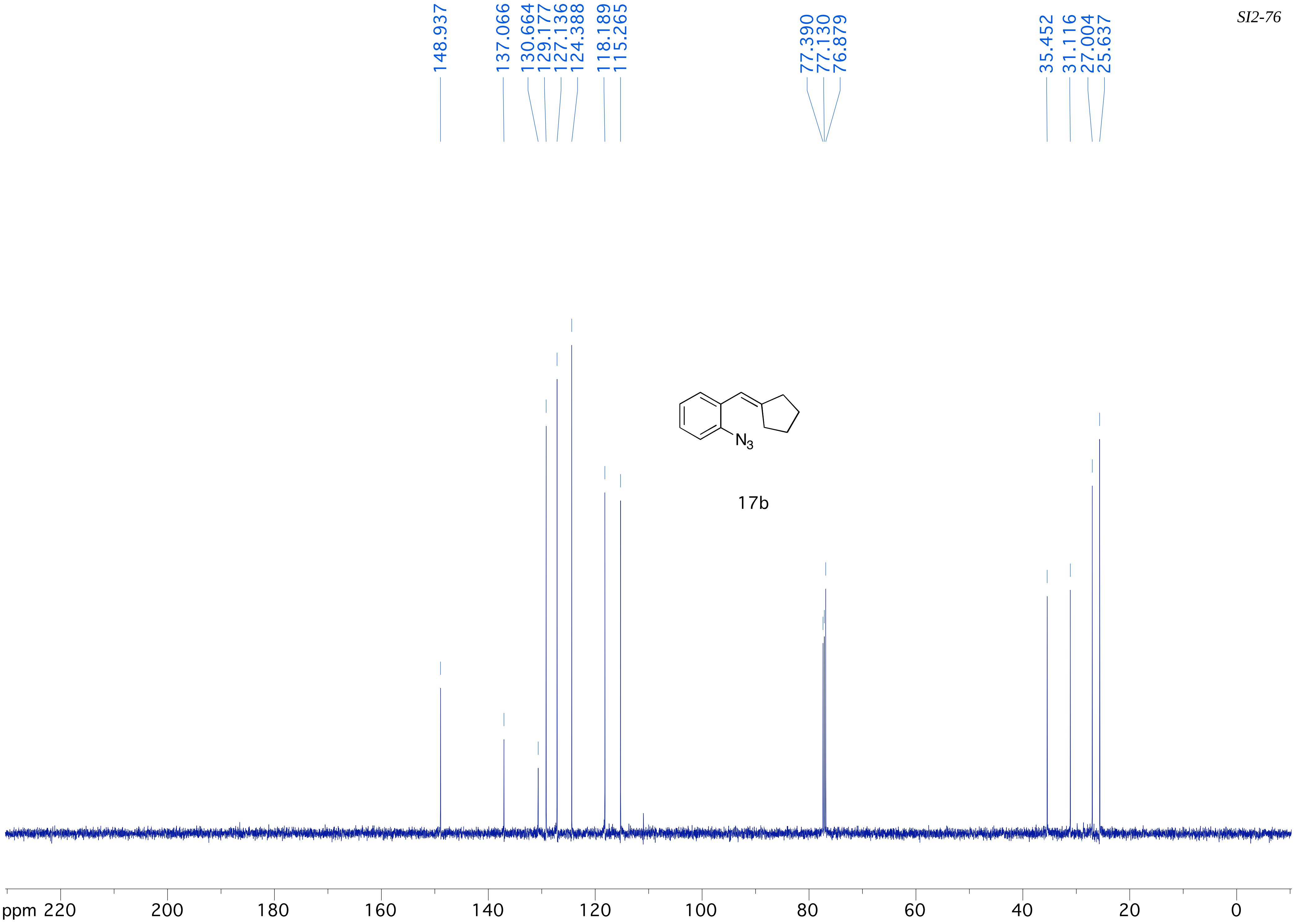
148.937
137.066
130.664
129.177
127.136
124.388
118.189
115.265
77.390
77.130
76.879
35.452
31.116
27.004
25.637
ppm 220
200
180
160
140
120
100
80
60
40
20
0

17b

SI2-77

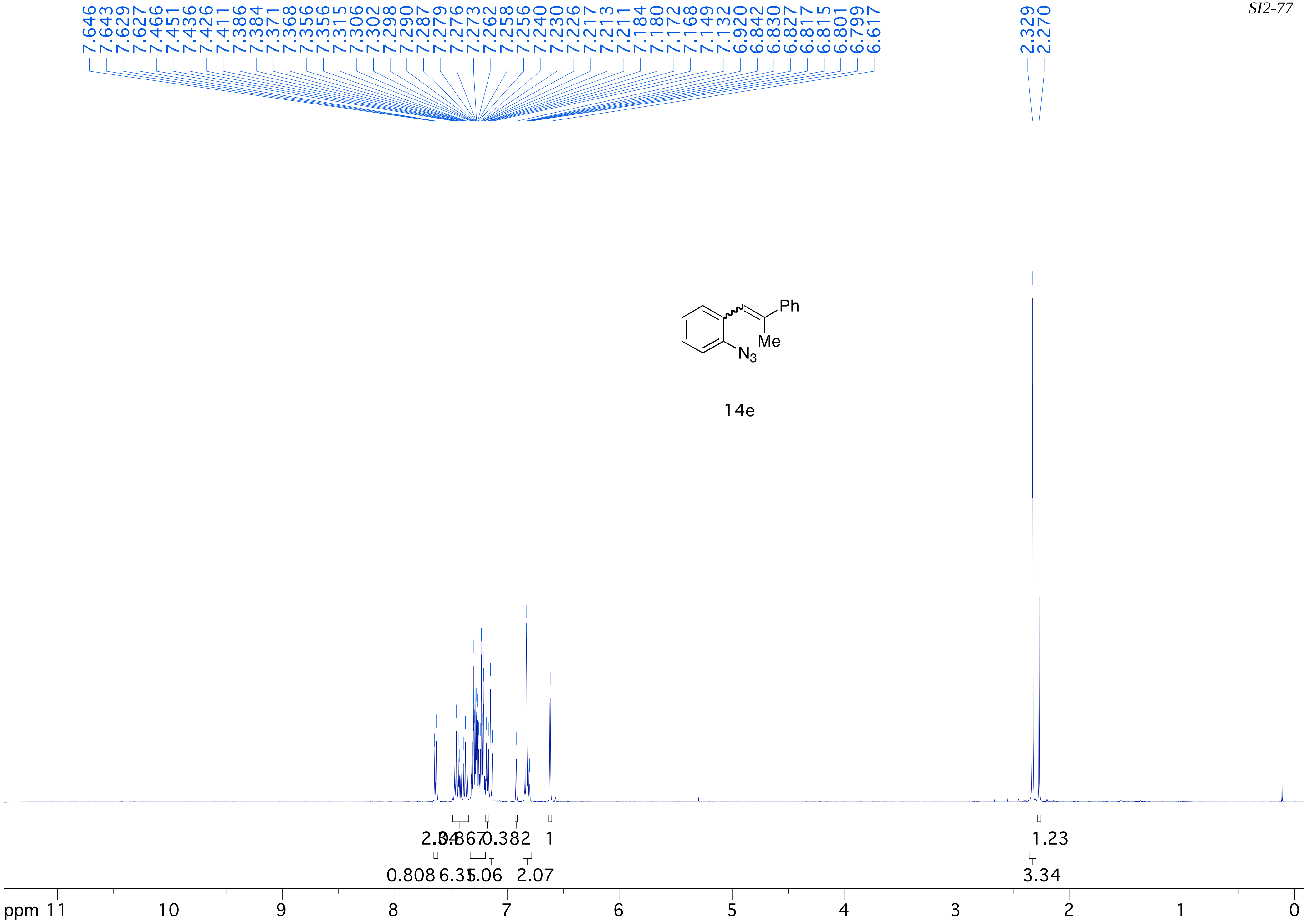
7.646
7.643
7.629
7.627
7.466
7.451
7.436
7.426
7.411
7.386
7.384
7.371
7.368
7.356
7.356
7.315
7.306
7.302
7.298
7.290
7.287
7.279
7.276
7.273
7.262
7.258
7.256
7.240
7.230
7.226
7.217
7.213
7.211
7.184
7.180
7.172
7.168
7.149
7.132
6.920
6.842
6.830
6.827
6.817
6.815
6.801
6.799
6.617
2.329
2.270
Ph
Me
N_3
14e
1
1.23
0.808
2.07
3.34
ppm 11
10
9
8
7
6
5
4
3
2
1
0

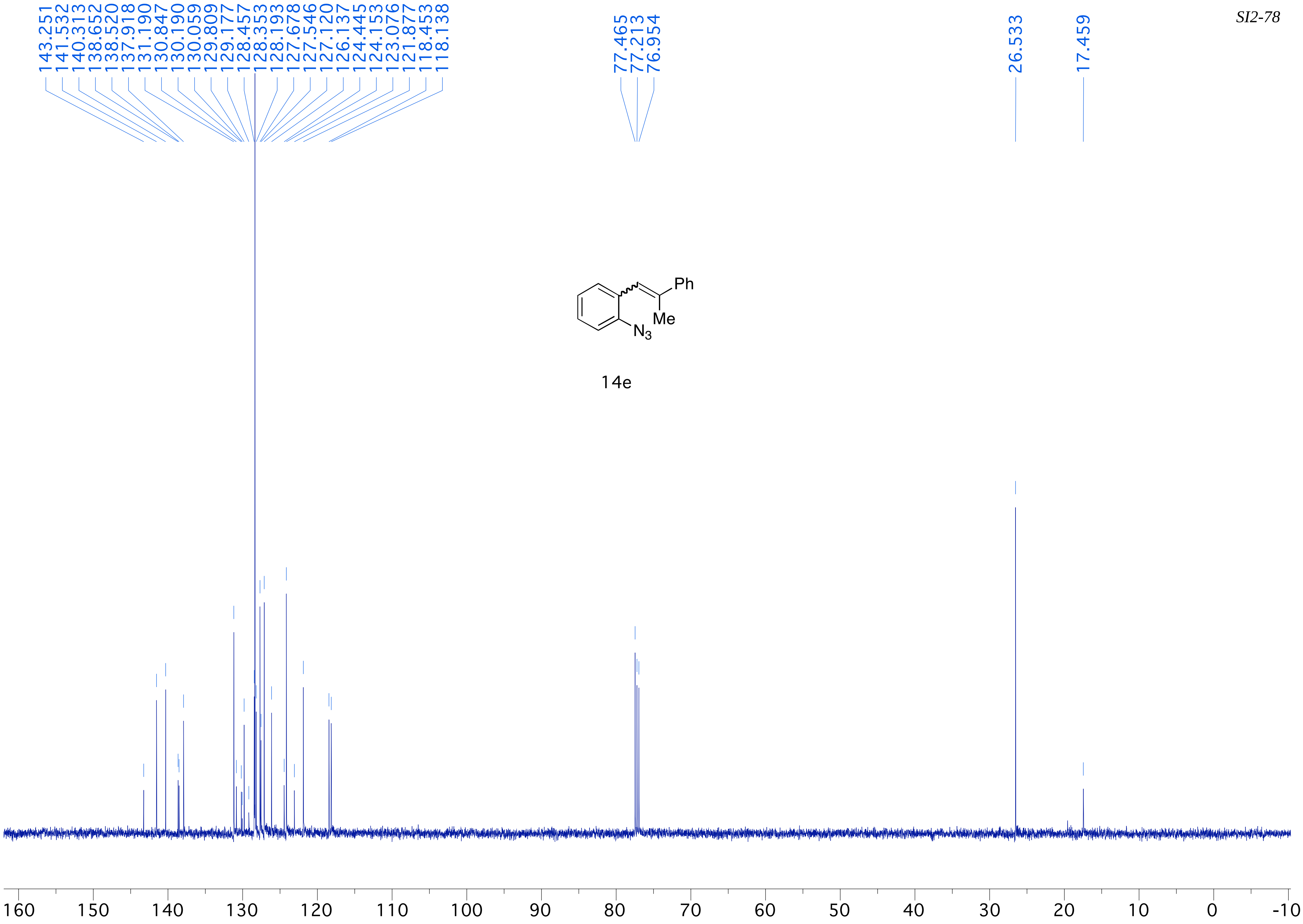
143.251
141.532
140.313
138.652
138.520
137.918
131.190
130.847
130.190
130.059
129.809
129.177
128.457
128.353
128.193
127.678
127.546
127.120
126.137
124.445
124.153
123.076
121.877
118.453
118.138
77.465
77.213
76.954
26.533
17.459
Ph
Me
N_3
14e
160 150 140 130 120 110 100 90 80 70 60 50 40 30 20 10 0 -10

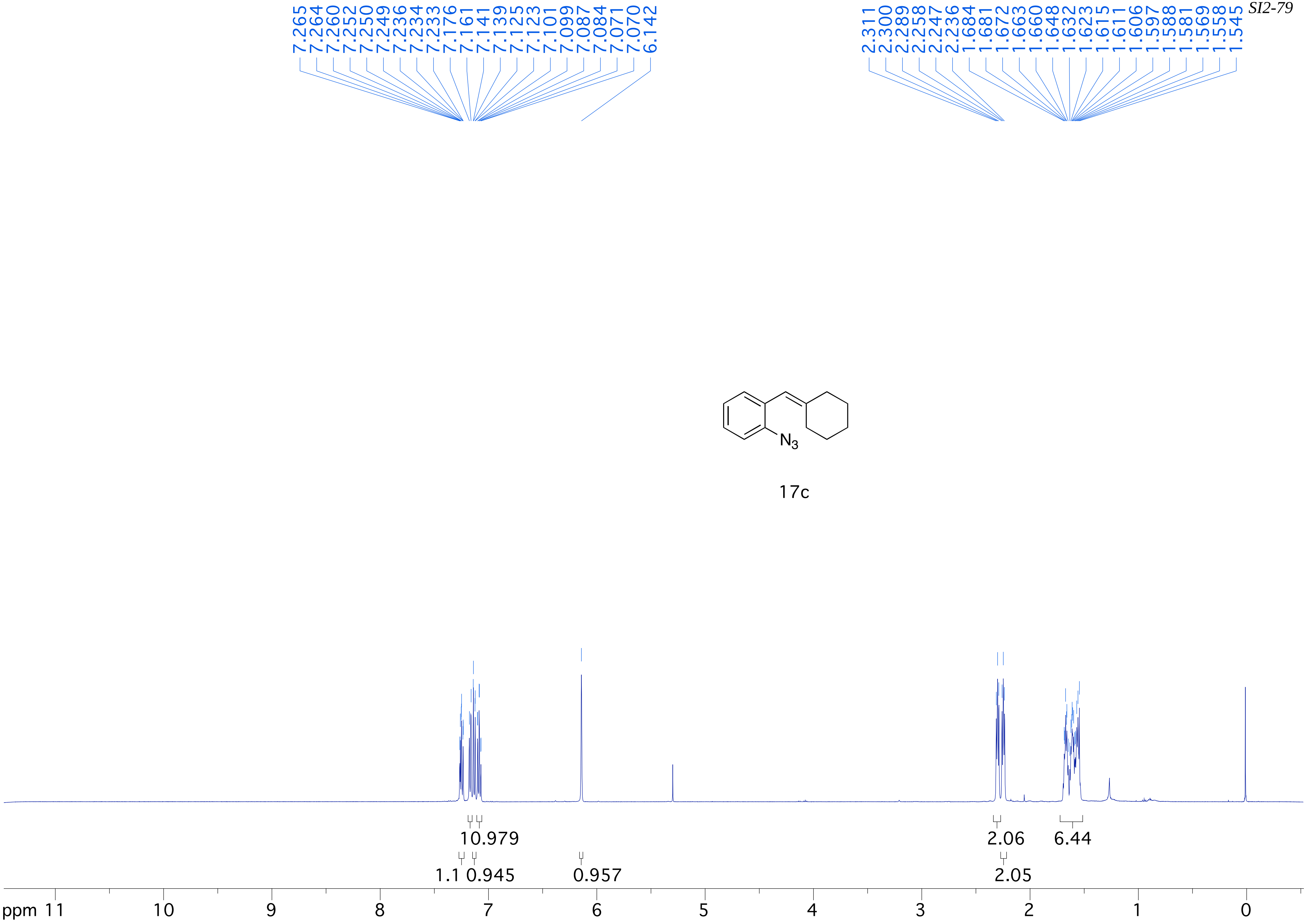
7.265
7.264
7.260
7.252
7.250
7.249
7.236
7.234
7.233
7.176
7.161
7.141
7.139
7.125
7.123
7.101
7.099
7.087
7.084
7.071
7.070
6.142
2.311
2.300
2.289
2.258
2.247
2.236
1.684
1.681
1.672
1.663
1.660
1.648
1.632
1.623
1.615
1.611
1.606
1.597
1.588
1.581
1.569
1.558
1.545
10.979
2.06
6.44
1.1
0.945
0.957
2.05
ppm 11
10
9
8
7
6
5
4
3
2
1
0
N3

17c

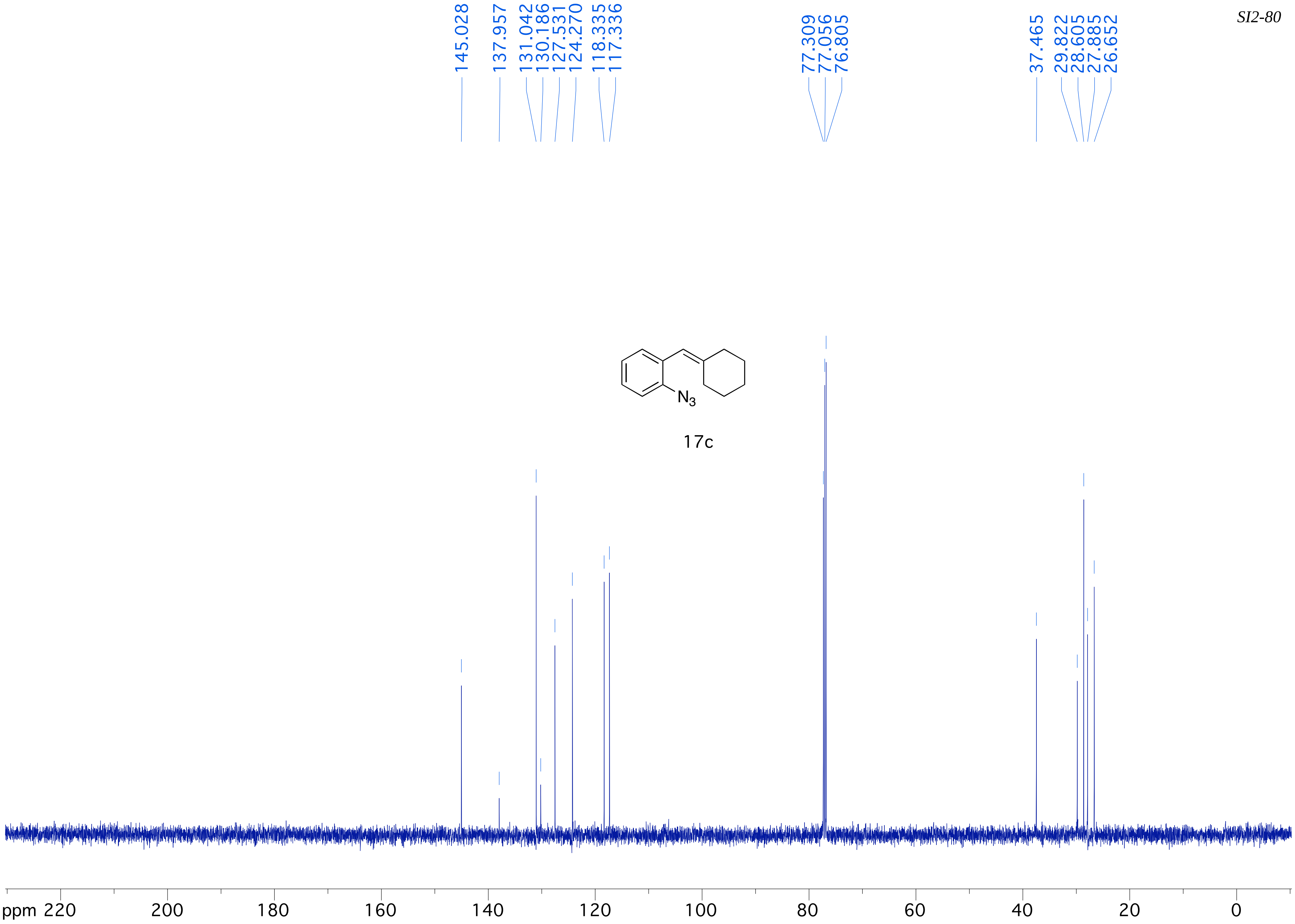
145.028
137.957
131.042
130.186
127.531
124.270
118.335
117.336
77.309
77.056
76.805
37.465
29.822
28.605
27.885
26.652
N3
17c
ppm 220
200
180
160
140
120
100
80
60
40
20
0

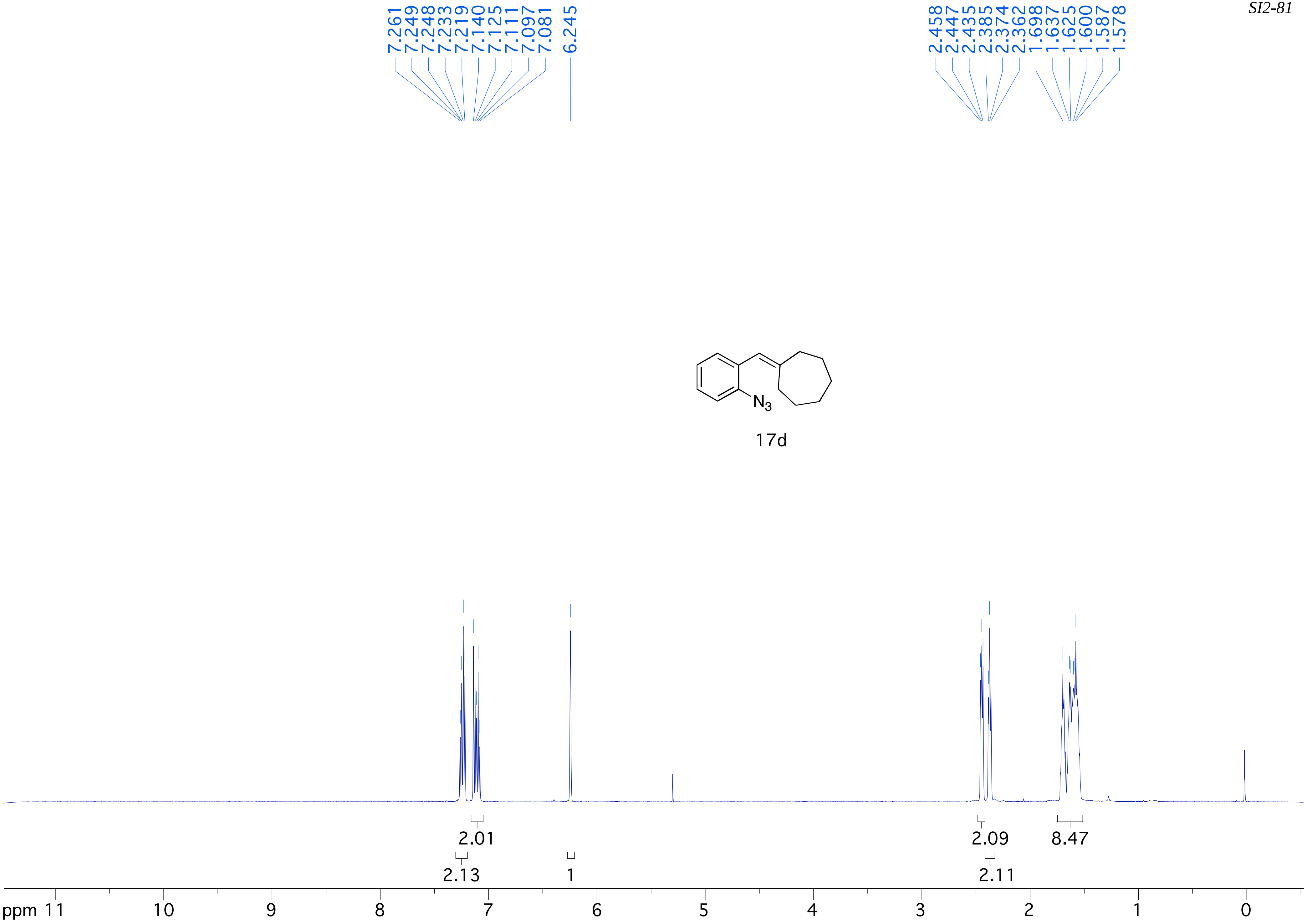
7.261
7.249
7.248
7.233
7.219
7.140
7.125
7.111
7.097
7.081
6.245
2.458
2.447
2.435
2.385
2.374
2.362
1.698
1.637
1.625
1.600
1.587
1.578
2.01
2.13
1
2.09
2.11
8.47
ppm 11 10 9 8 7 6 5 4 3 2 1 0
N3

17d

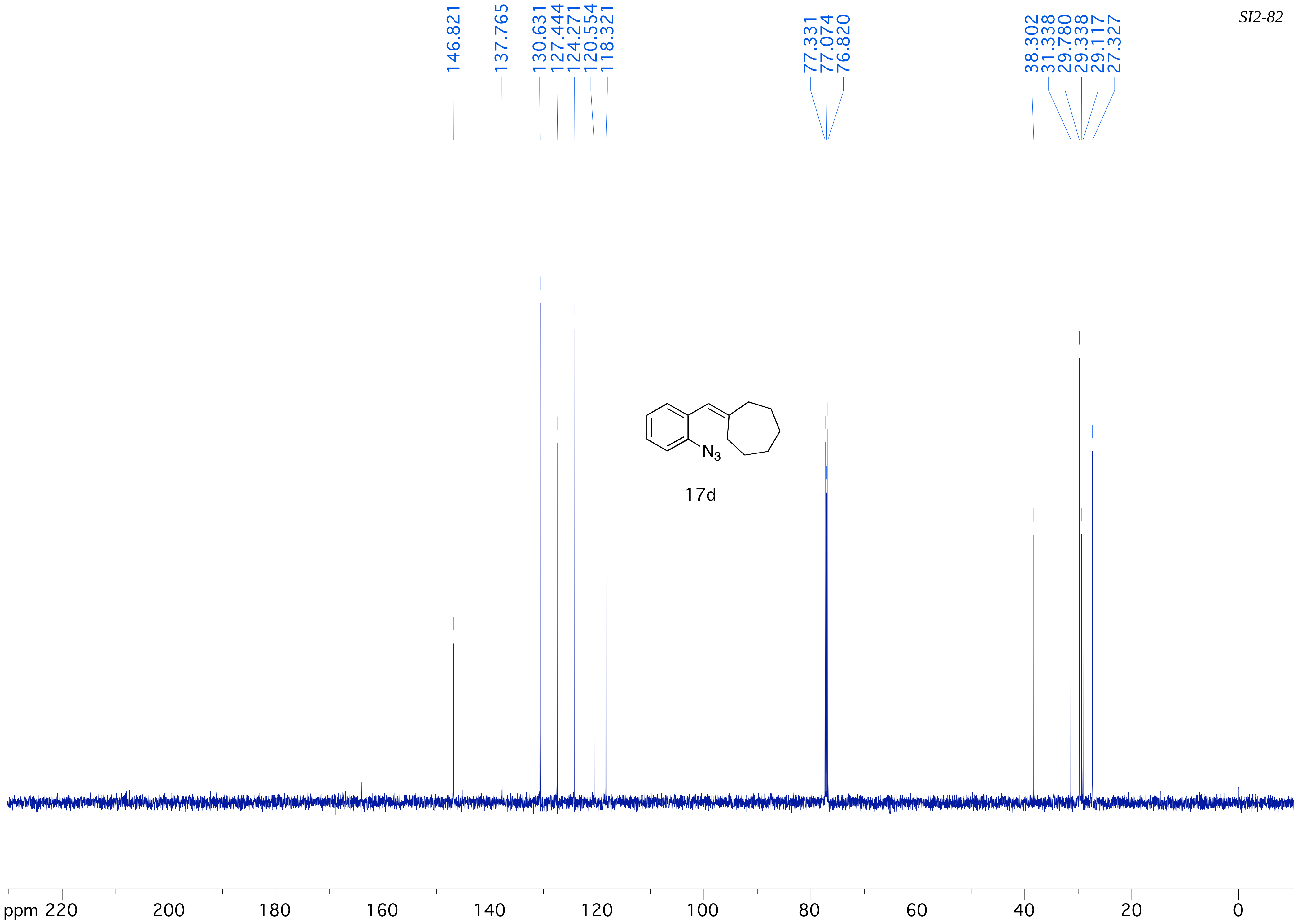
146.821
137.765
130.631
127.444
124.271
120.554
118.321
77.331
77.074
76.820
38.302
31.338
29.780
29.338
29.117
27.327
N_3
17d
ppm 220
200
180
160
140
120
100
80
60
40
20
0

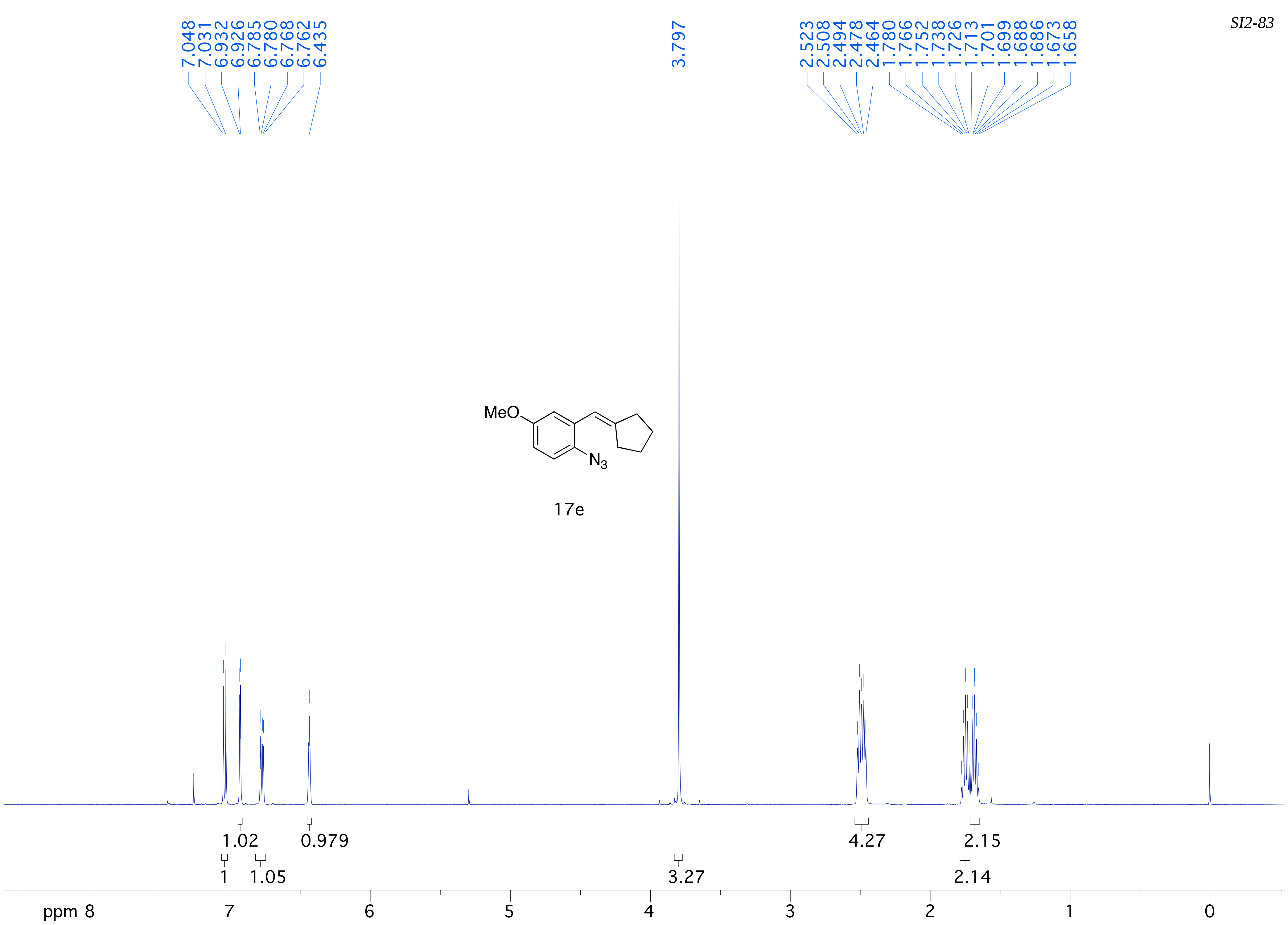
7.048
7.031
6.932
6.926
6.785
6.780
6.768
6.762
6.435
3.797
2.523
2.508
2.494
2.478
2.464
1.780
1.766
1.752
1.738
1.726
1.713
1.701
1.699
1.688
1.686
1.673
1.658
MeO
N3
17e
1.02
0.979
4.27
2.15
1
1.05
3.27
2.14
ppm 8
7
6
5
4
3
2
1
0

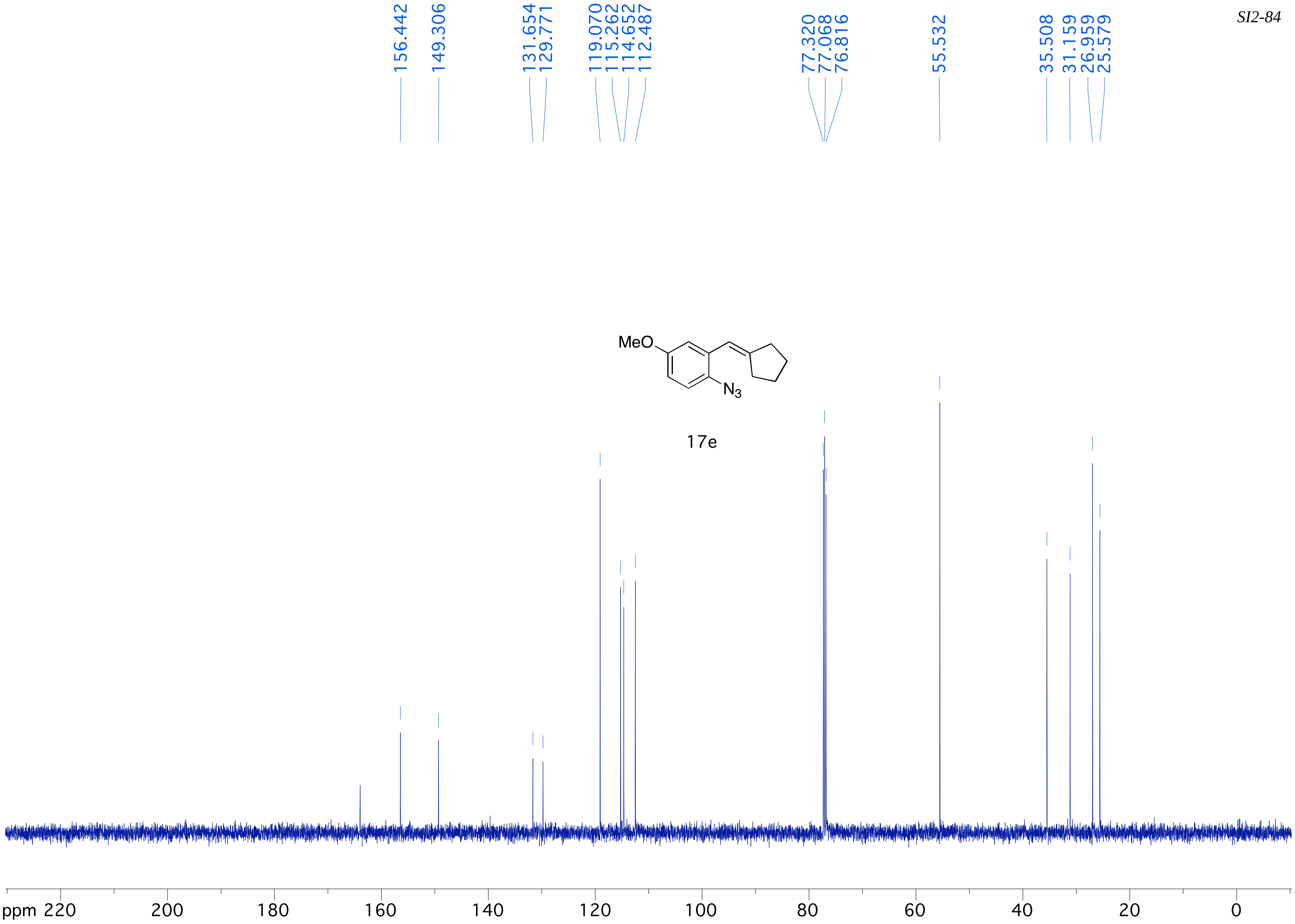
156.442
149.306
131.654
129.771
119.070
115.262
114.652
112.487
77.320
77.068
76.816
55.532
35.508
31.159
26.959
25.579
MeO
N3
17e
ppm 220
200
180
160
140
120
100
80
60
40
20
0

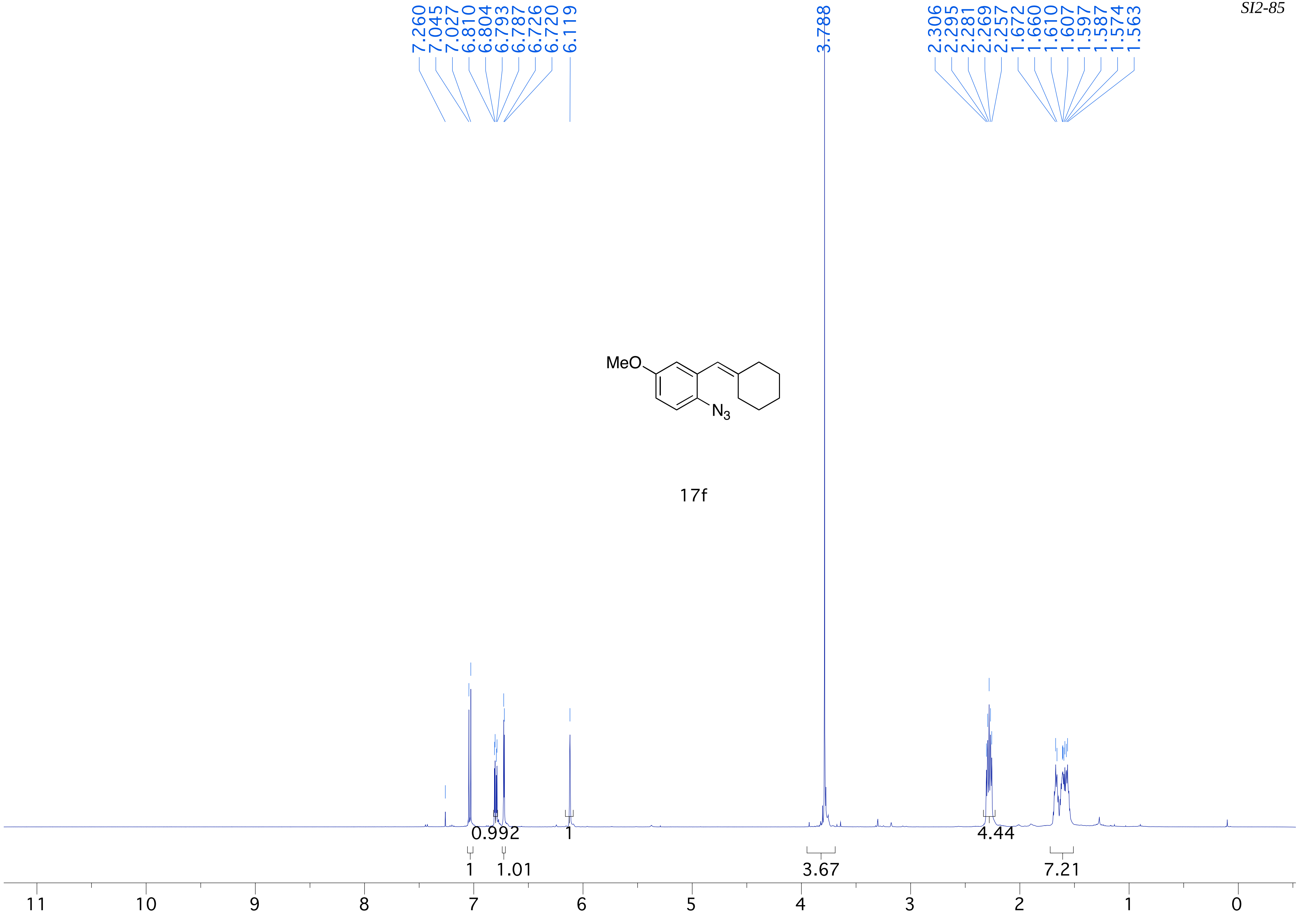
7.260
7.045
7.027
6.810
6.804
6.793
6.787
6.726
6.720
6.119
3.788
2.306
2.295
2.281
2.269
2.257
1.672
1.660
1.610
1.607
1.597
1.587
1.574
1.563
MeO
N3
17f
0.992
1
4.44
1
1.01
3.67
7.21
11
10
9
8
7
6
5
4
3
2
1
0

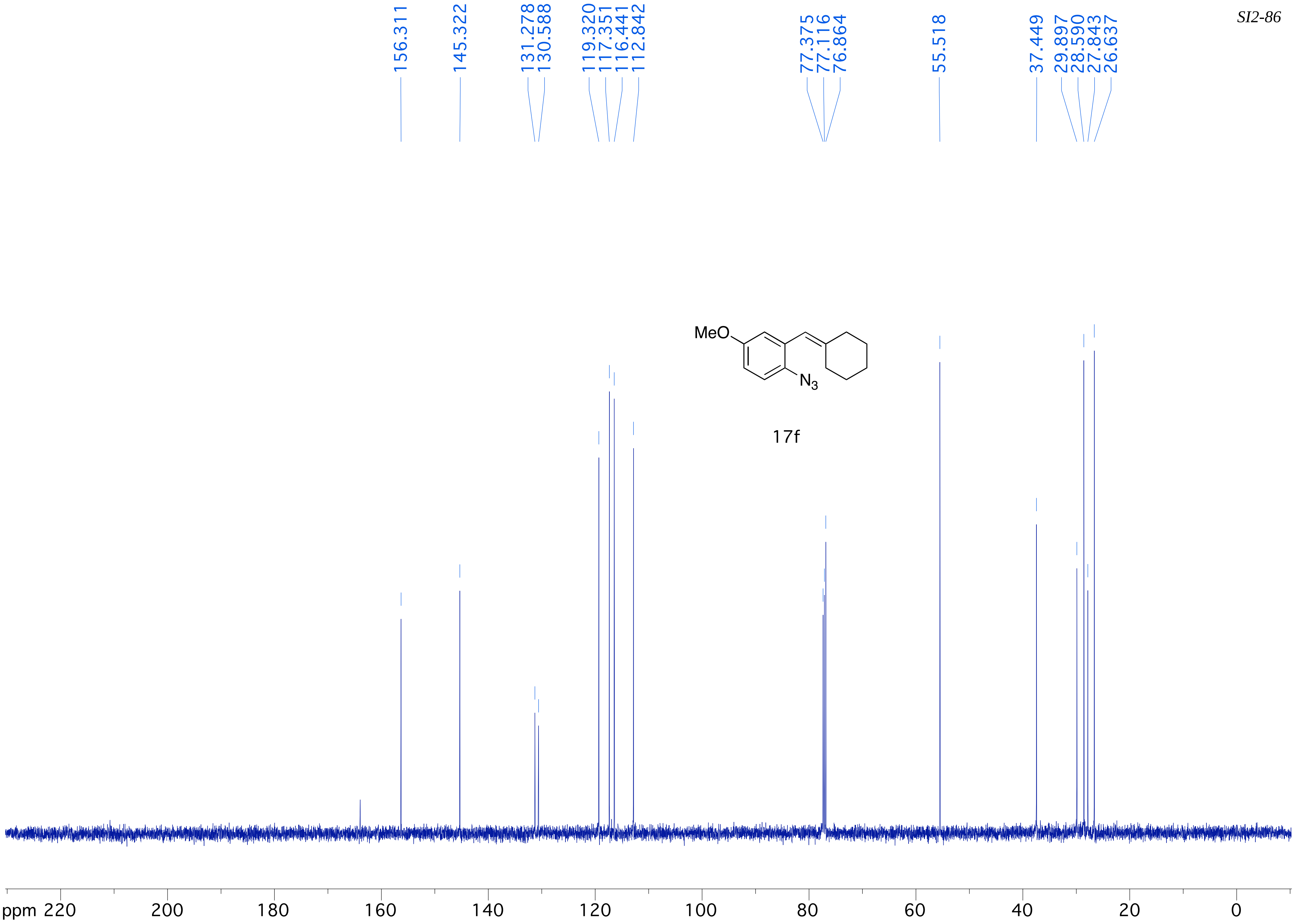
156.311
145.322
131.278
130.588
119.320
117.351
116.441
112.842
77.375
77.116
76.864
55.518
37.449
29.897
28.590
27.843
26.637
MeO
N_3
17f
ppm 220
200
180
160
140
120
100
80
60
40
20
0

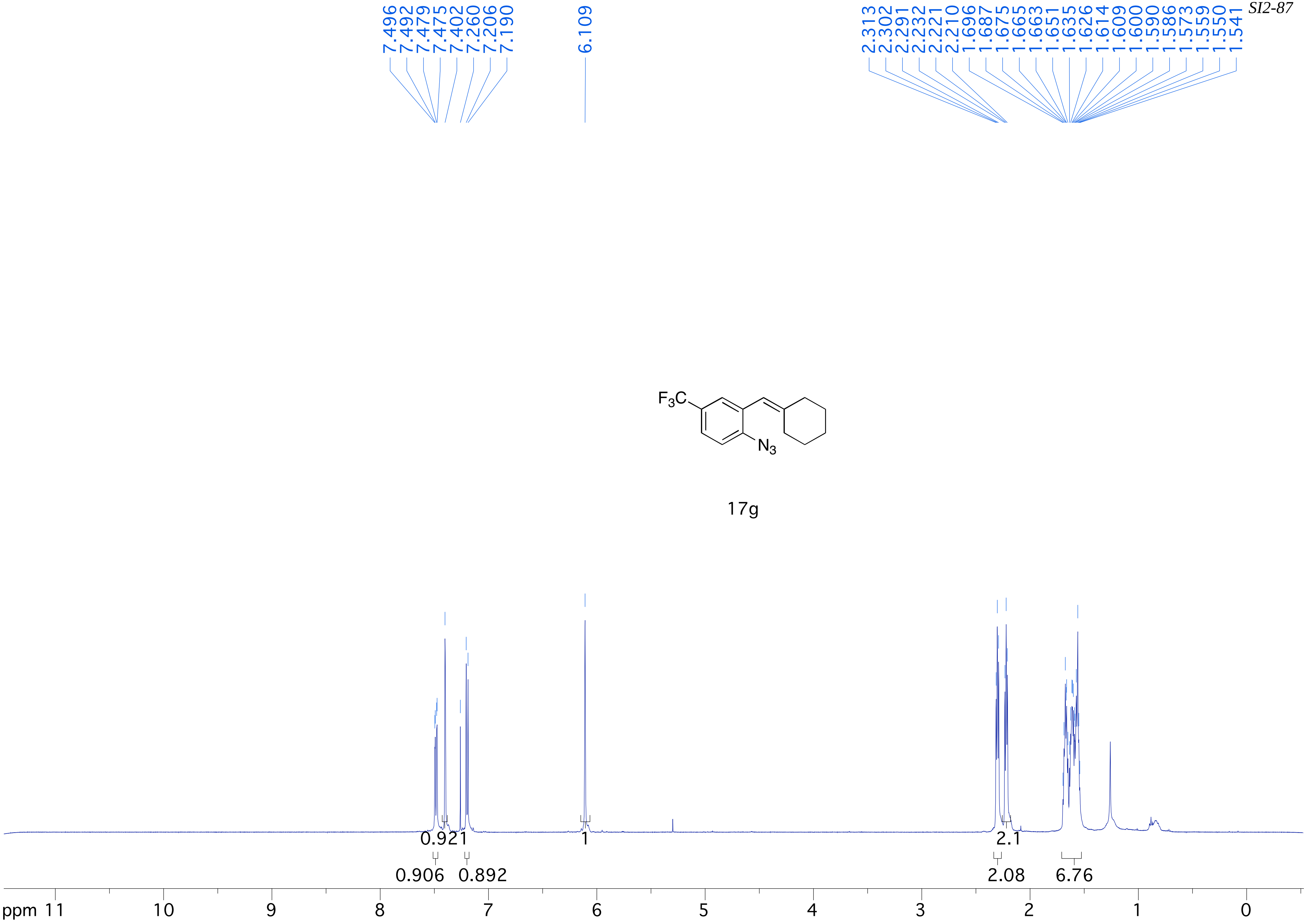
7.496
7.492
7.479
7.475
7.402
7.260
7.206
7.190
6.109
2.313
2.302
2.291
2.232
2.221
2.210
1.696
1.687
1.675
1.665
1.663
1.651
1.635
1.626
1.614
1.609
1.600
1.590
1.586
1.573
1.559
1.550
1.541
F_3C
N_3
17g
0.921
1
2.1
0.906
0.892
2.08
6.76
ppm 11 10 9 8 7 6 5 4 3 2 1 0

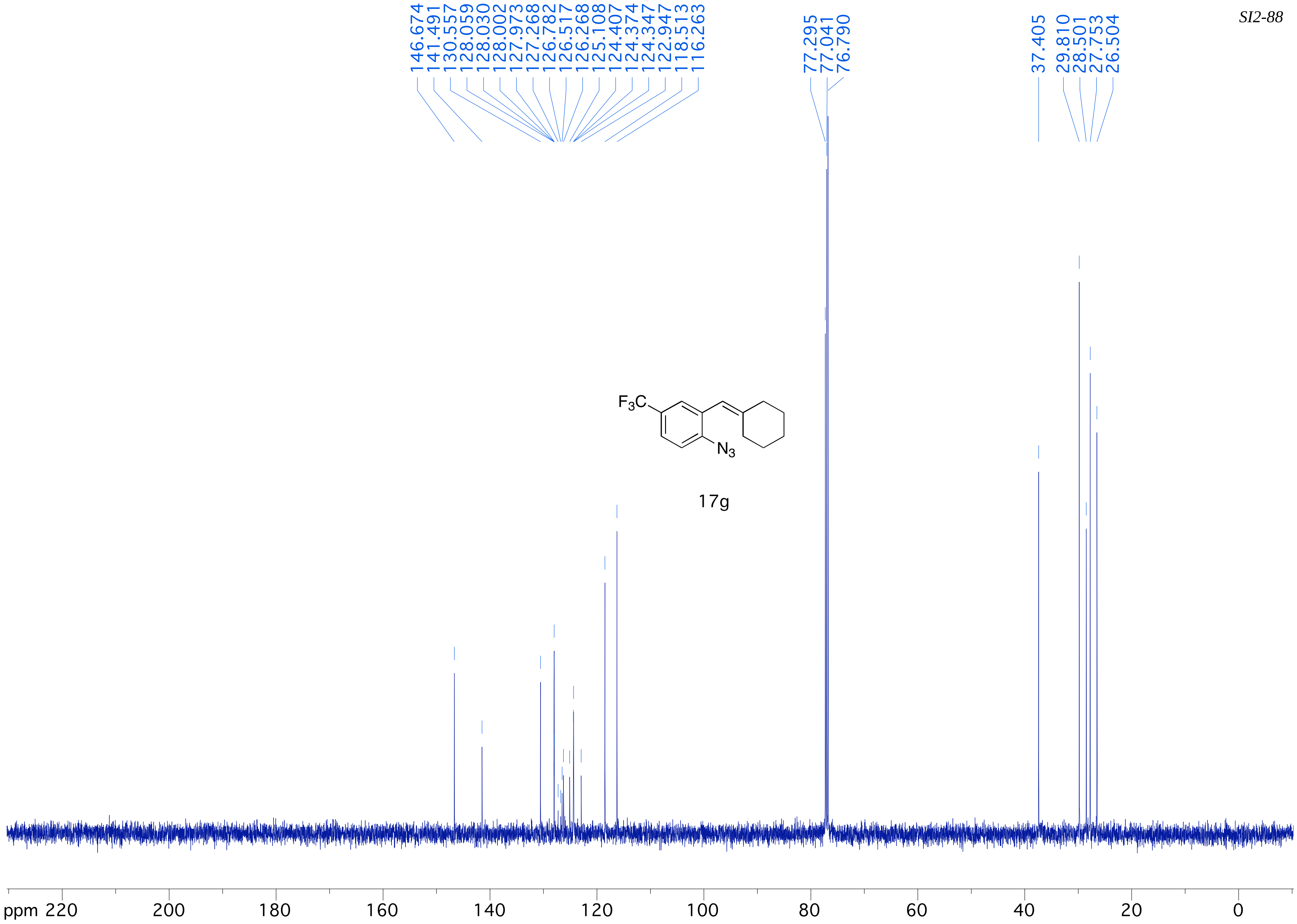
146.674
141.491
130.557
128.059
128.030
128.002
127.973
127.268
126.782
126.517
126.268
125.108
124.407
124.374
124.347
122.947
118.513
116.263
77.295
77.041
76.790
37.405
29.810
28.501
27.753
26.504
F3C
N3
17g
ppm 220
200
180
160
140
120
100
80
60
40
20
0

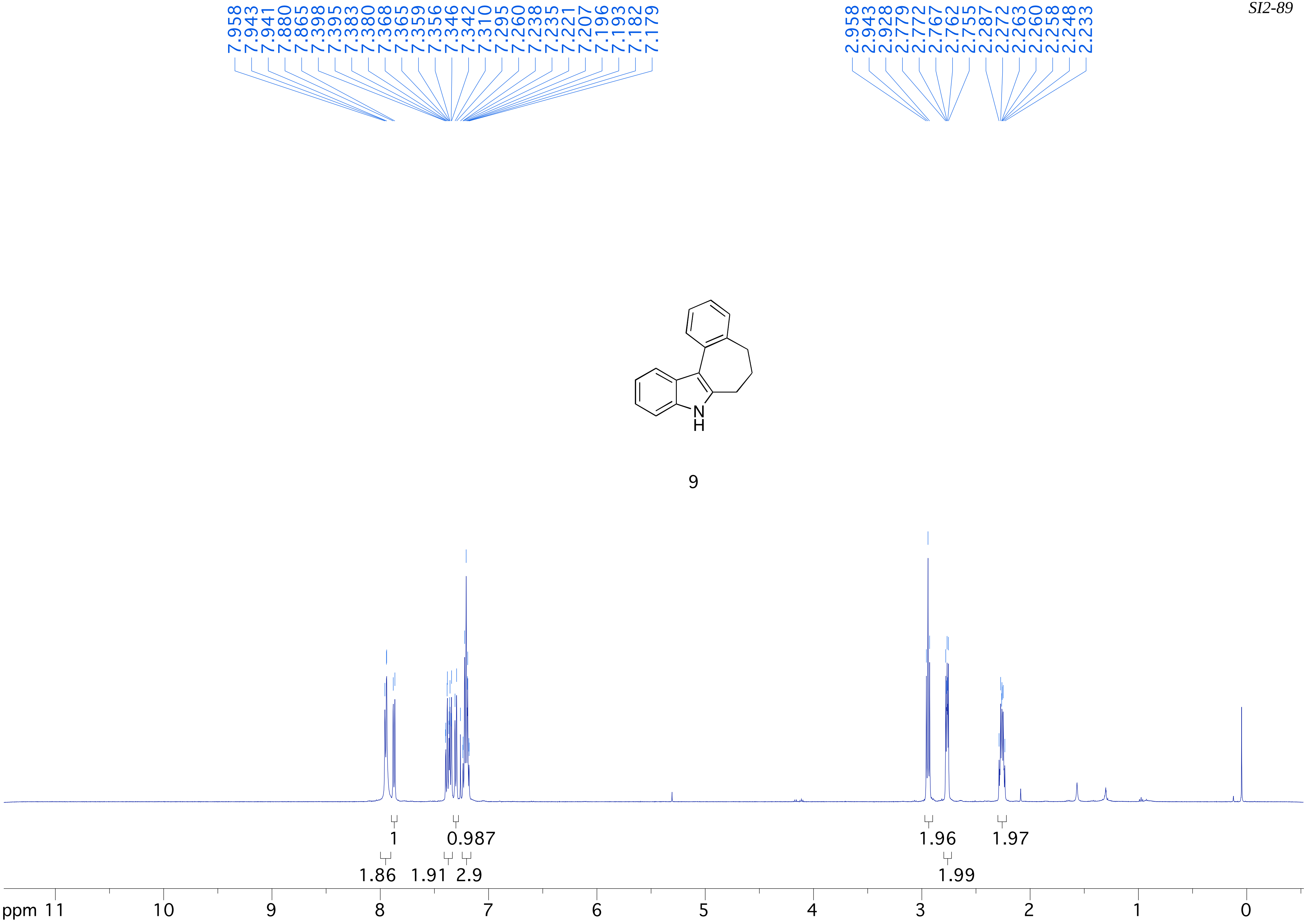
7.958
7.943
7.941
7.880
7.865
7.398
7.395
7.383
7.380
7.368
7.365
7.359
7.356
7.346
7.342
7.310
7.295
7.260
7.238
7.235
7.221
7.207
7.196
7.193
7.182
7.179
2.958
2.943
2.928
2.779
2.772
2.767
2.762
2.755
2.287
2.272
2.263
2.260
2.258
2.248
2.233
N
H
9
1
0.987
1.96
1.97
1.86
1.91
2.9
1.99
ppm
11
10
9
8
7
6
5
4
3
2
1
0

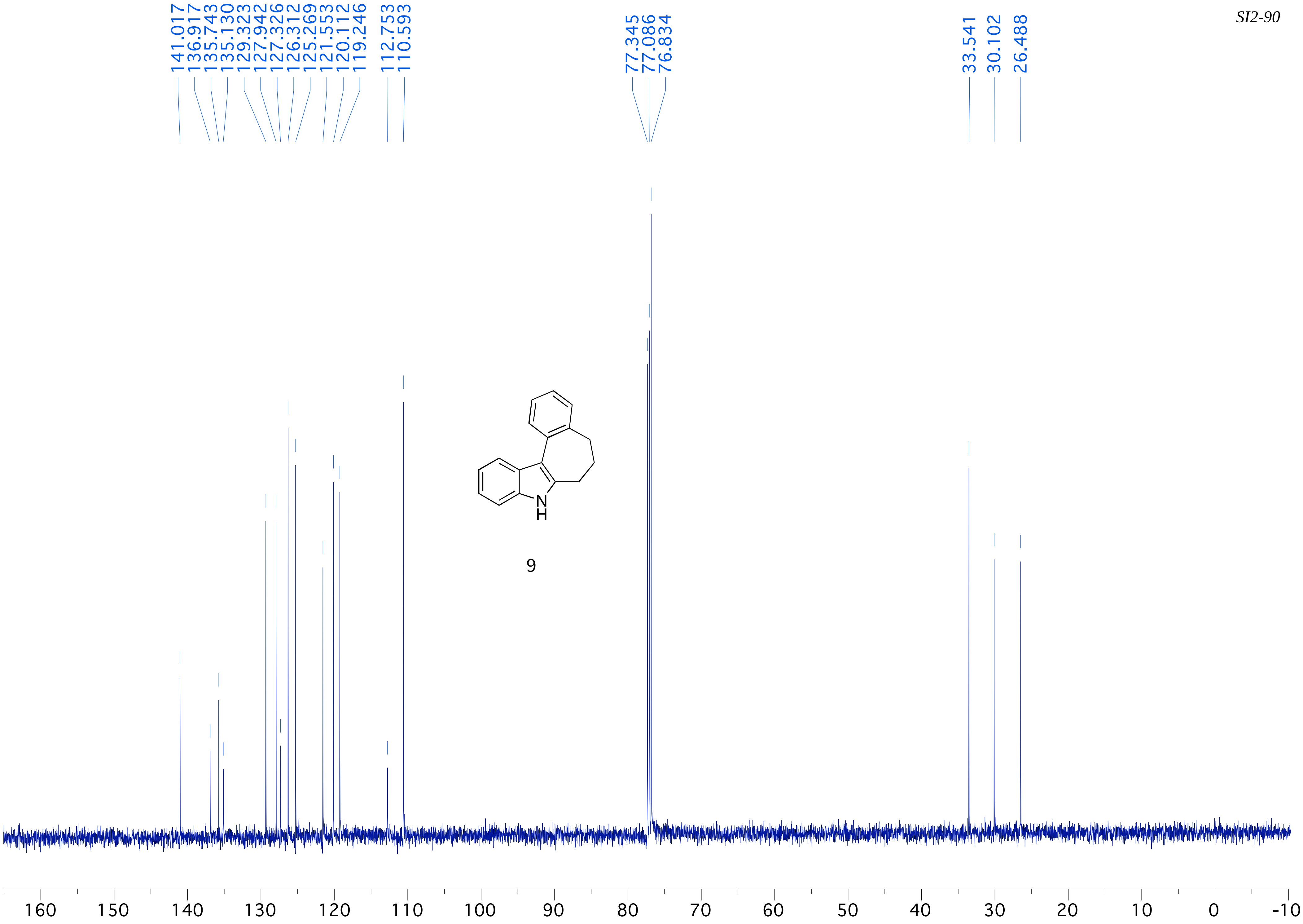
141.017
136.917
135.743
135.130
129.323
127.942
127.326
126.312
125.269
121.553
120.112
119.246
112.753
110.593
77.345
77.086
76.834
33.541
30.102
26.488
N
H
160
150
140
130
120
110
100
90
80
70
60
50
40
30
20
10
0
-10

9

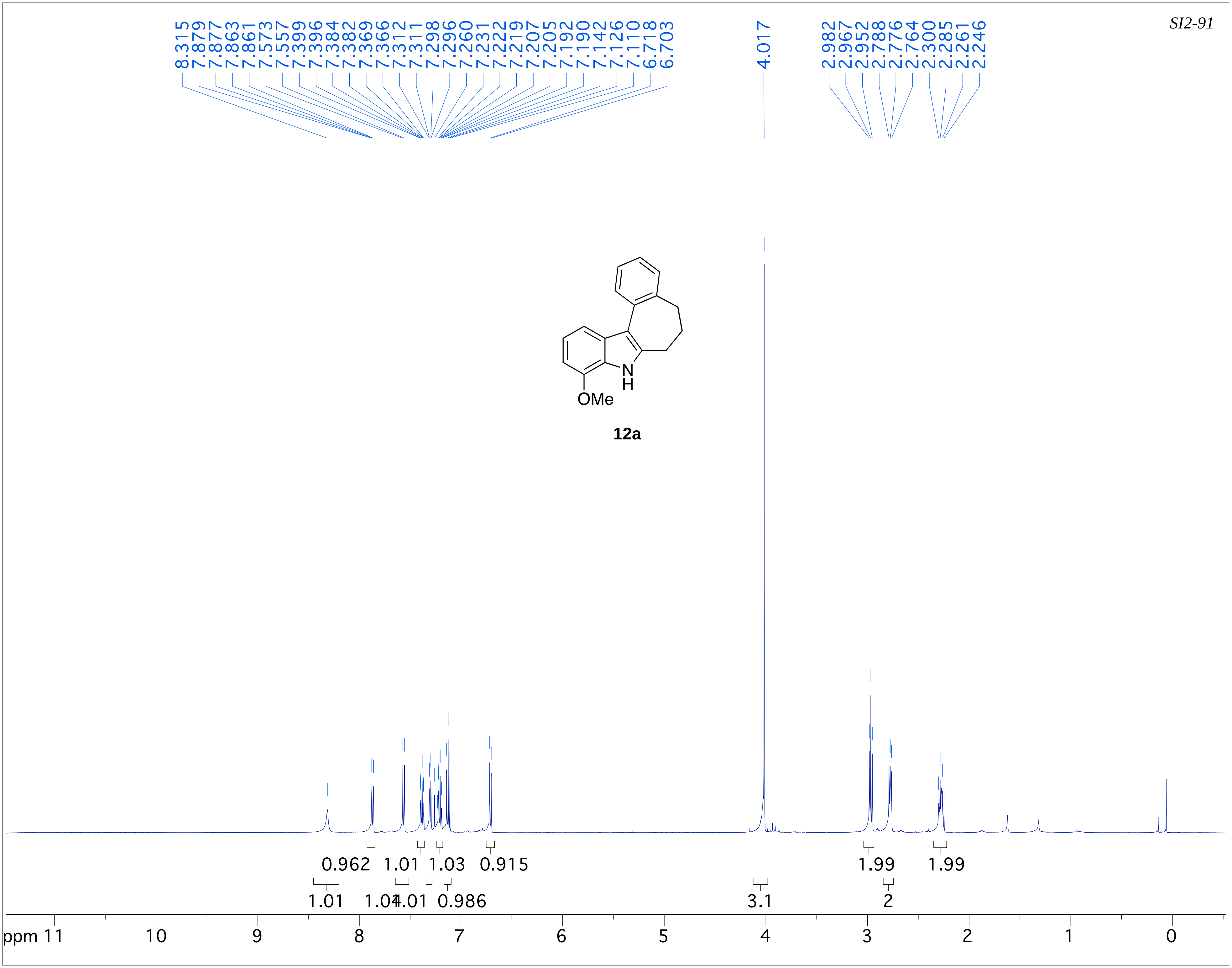
SI2-91
8.315
7.879
7.877
7.863
7.861
7.573
7.557
7.399
7.396
7.384
7.382
7.369
7.366
7.312
7.311
7.298
7.296
7.260
7.231
7.222
7.219
7.207
7.205
7.192
7.190
7.142
7.126
7.110
6.718
6.703
4.017
2.982
2.967
2.952
2.788
2.776
2.764
2.300
2.285
2.261
2.246
N
H
OMe
12a
0.962
1.01
1.03
0.915
1.99
1.99
1.01
1.04
1.01
0.986
3.1
2
ppm 11
10
9
8
7
6
5
4
3
2
1
0

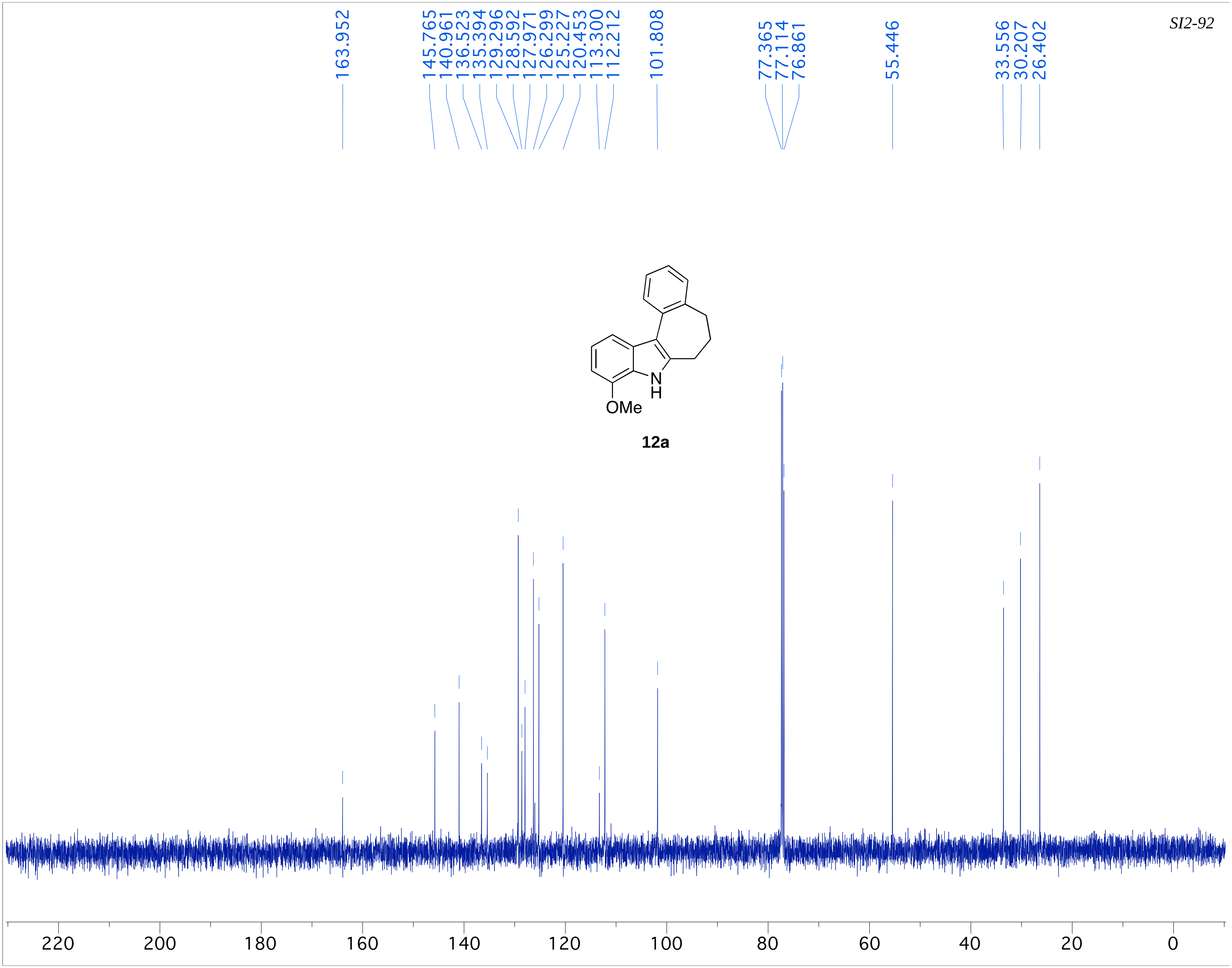
163.952
145.765
140.961
136.523
135.394
129.296
128.592
127.971
126.299
125.227
120.453
113.300
112.212
101.808
77.365
77.114
76.861
55.446
33.556
30.207
26.402
N
H
OMe
12a
220
200
180
160
140
120
100
80
60
40
20
0

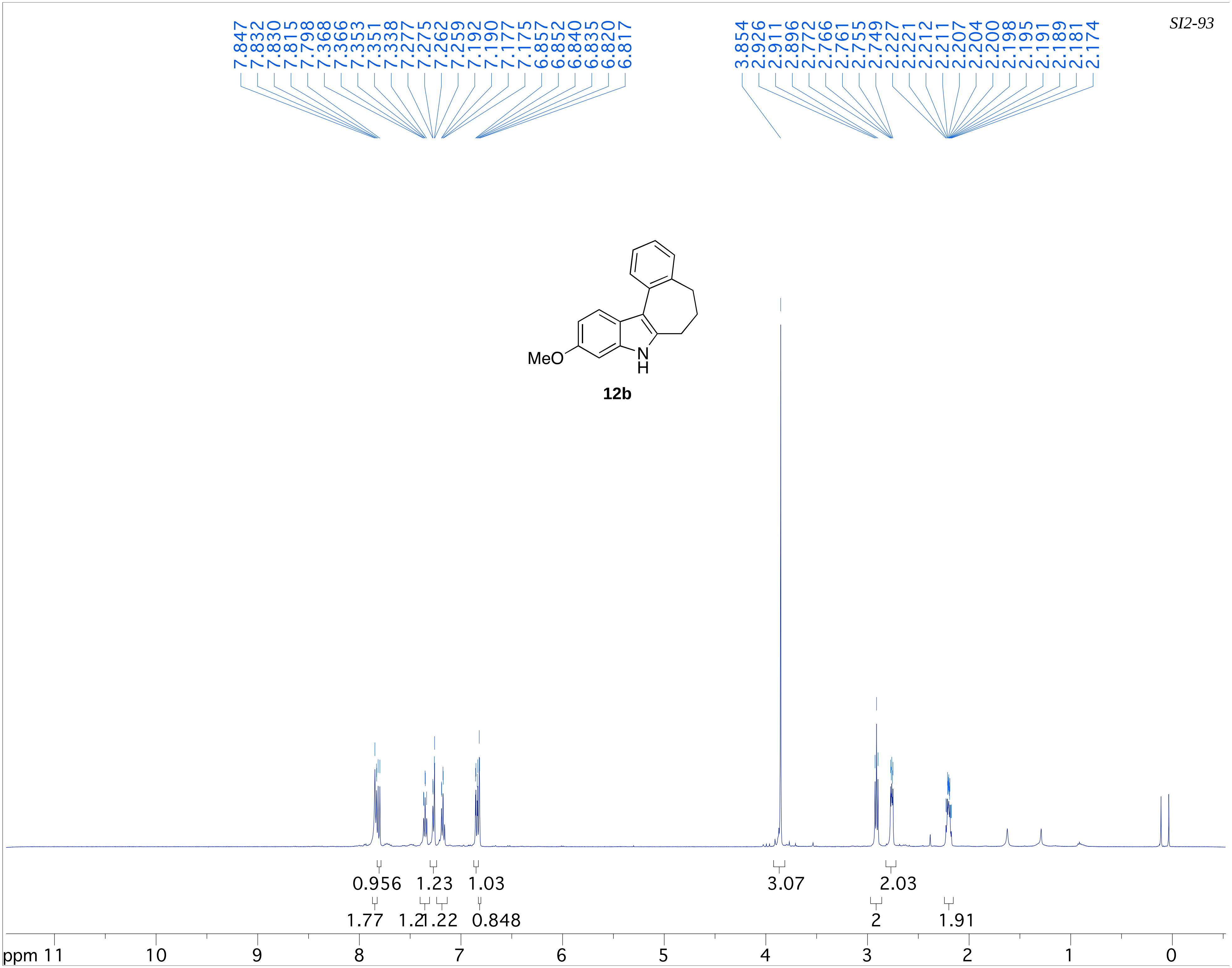
SI2-93
7.847
7.832
7.830
7.815
7.798
7.368
7.366
7.353
7.351
7.338
7.277
7.275
7.262
7.259
7.192
7.190
7.177
7.175
6.857
6.852
6.840
6.835
6.820
6.817
3.854
2.926
2.911
2.896
2.772
2.766
2.761
2.755
2.749
2.227
2.221
2.212
2.211
2.207
2.204
2.200
2.198
2.195
2.191
2.189
2.181
2.174
MeO
N
H
12b
0.956
1.23
1.03
3.07
2.03
1.77
1.21
1.22
0.848
2
1.91
ppm 11
10
9
8
7
6
5
4
3
2
1
0

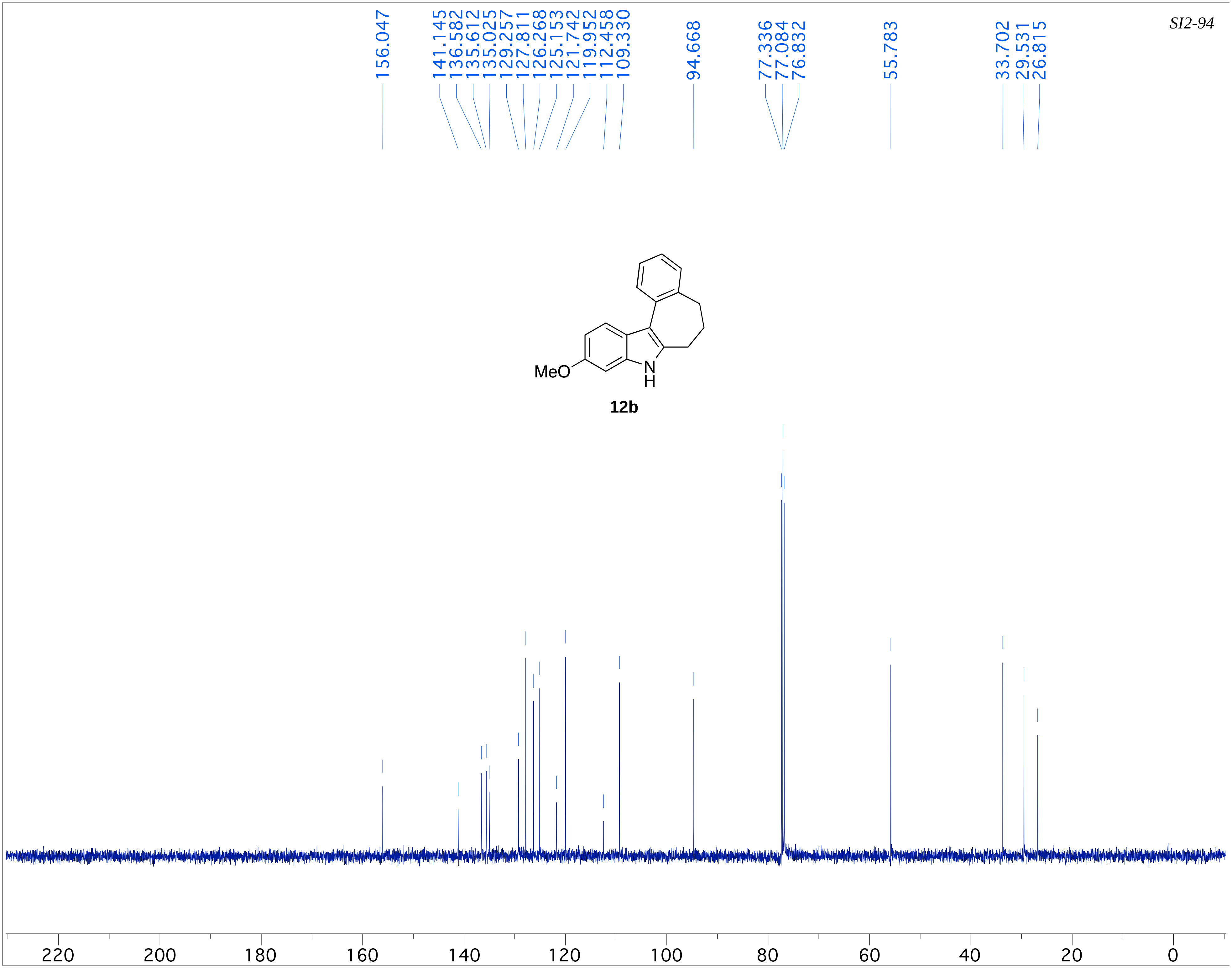
156.047
141.145
136.582
135.612
135.025
129.257
127.811
126.268
125.153
121.742
119.952
112.458
109.330
94.668
77.336
77.084
76.832
55.783
33.702
29.531
26.815
MeO
N
H
12b
220
200
180
160
140
120
100
80
60
40
20
0

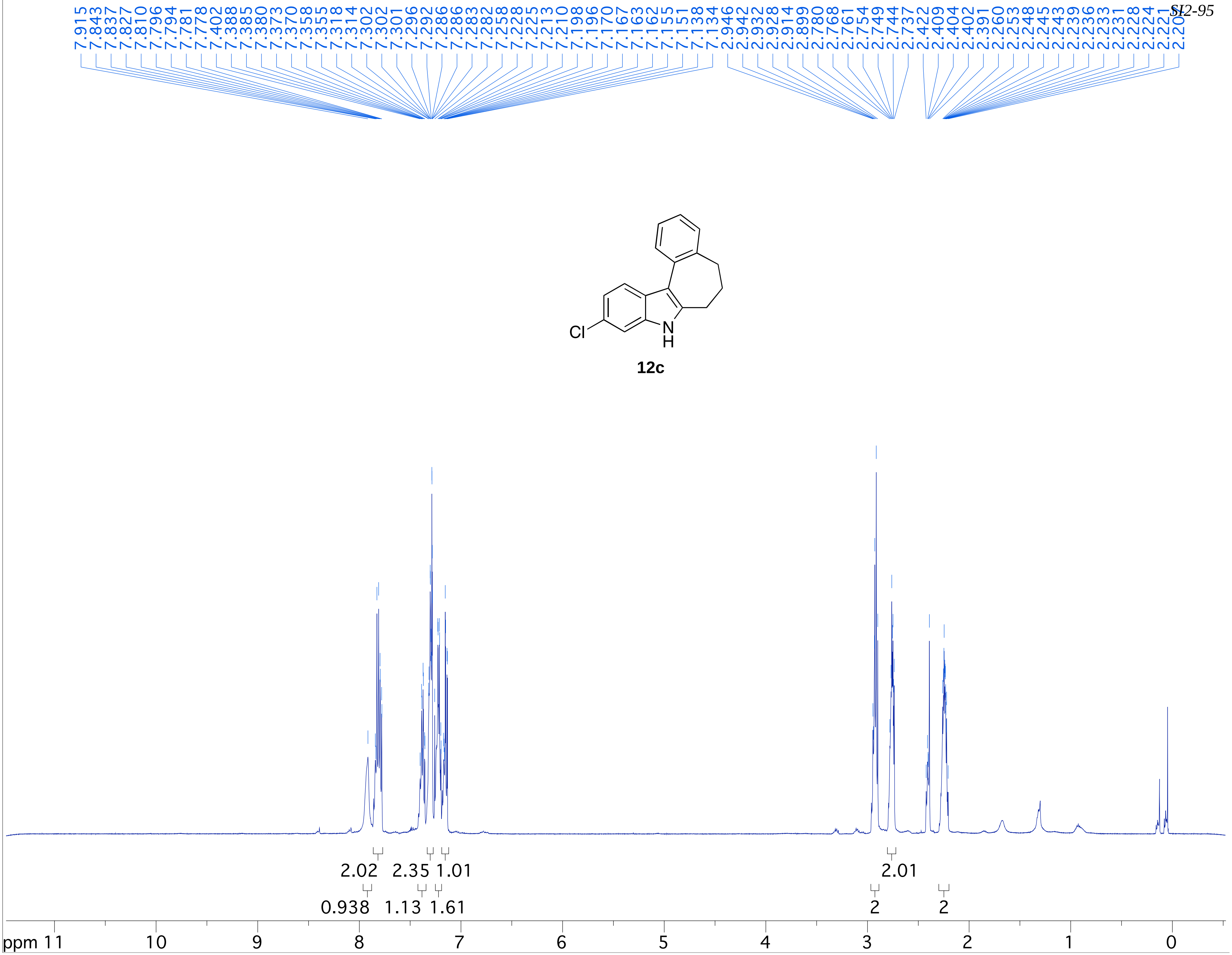
7.915
7.843
7.837
7.827
7.810
7.796
7.794
7.781
7.778
7.402
7.388
7.385
7.380
7.373
7.370
7.358
7.355
7.318
7.314
7.302
7.302
7.301
7.296
7.292
7.286
7.286
7.283
7.282
7.258
7.228
7.225
7.213
7.210
7.198
7.196
7.170
7.167
7.163
7.162
7.155
7.151
7.138
7.134
2.946
2.942
2.932
2.928
2.914
2.899
2.780
2.768
2.761
2.754
2.749
2.744
2.737
2.422
2.409
2.404
2.402
2.391
2.260
2.253
2.248
2.245
2.243
2.239
2.236
2.233
2.231
2.228
2.224
2.221
2.220
Cl
N
H
12c
2.02
2.35
1.01
2.01
0.938
1.13
1.61
2
2
ppm 11
10
9
8
7
6
5
4
3
2
1
0

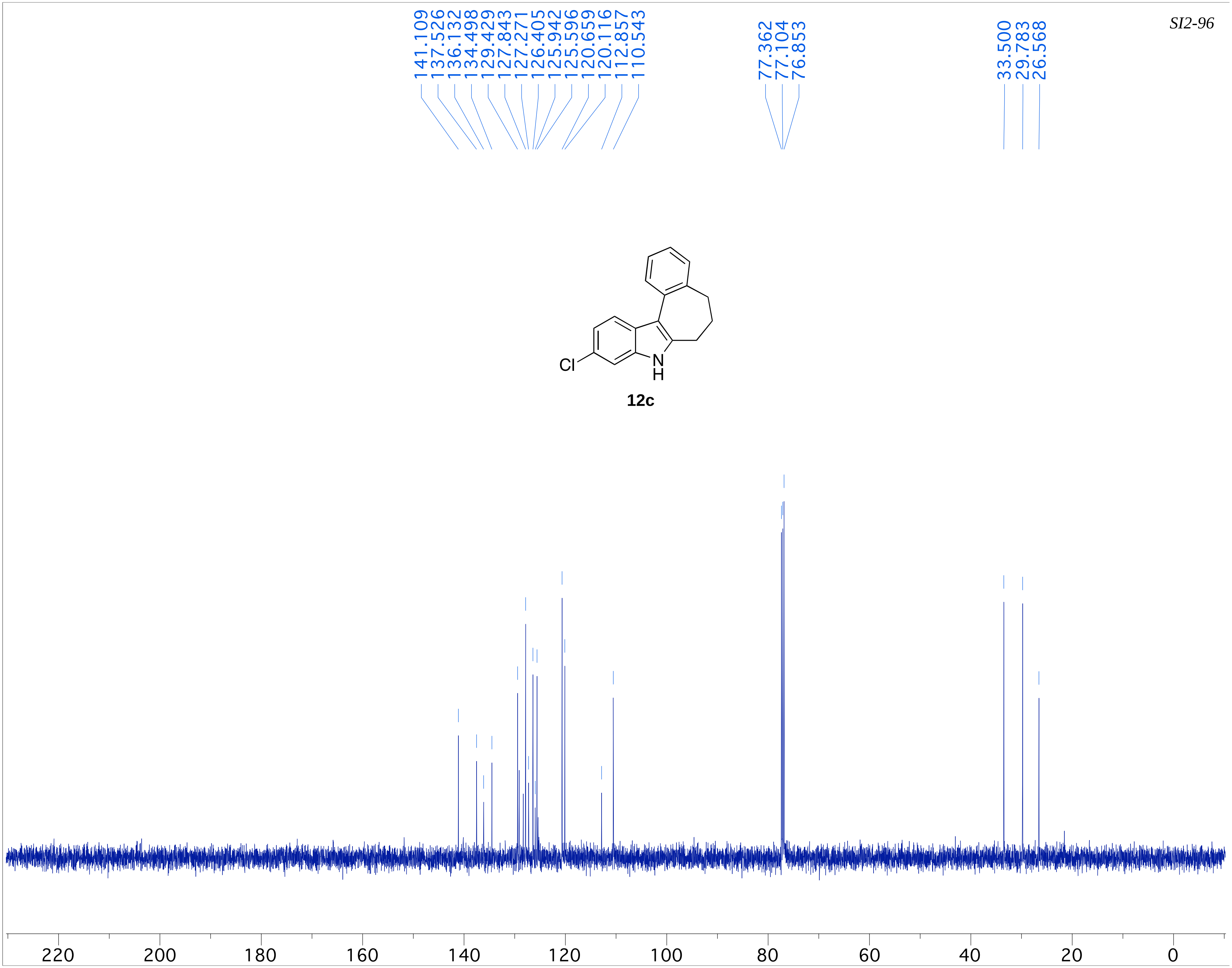
SI2-96
141.109
137.526
136.132
134.498
129.429
127.843
127.271
126.405
125.942
125.596
120.659
120.116
112.857
110.543
77.362
77.104
76.853
33.500
29.783
26.568
Cl
N
H
12c
220
200
180
160
140
120
100
80
60
40
20
0

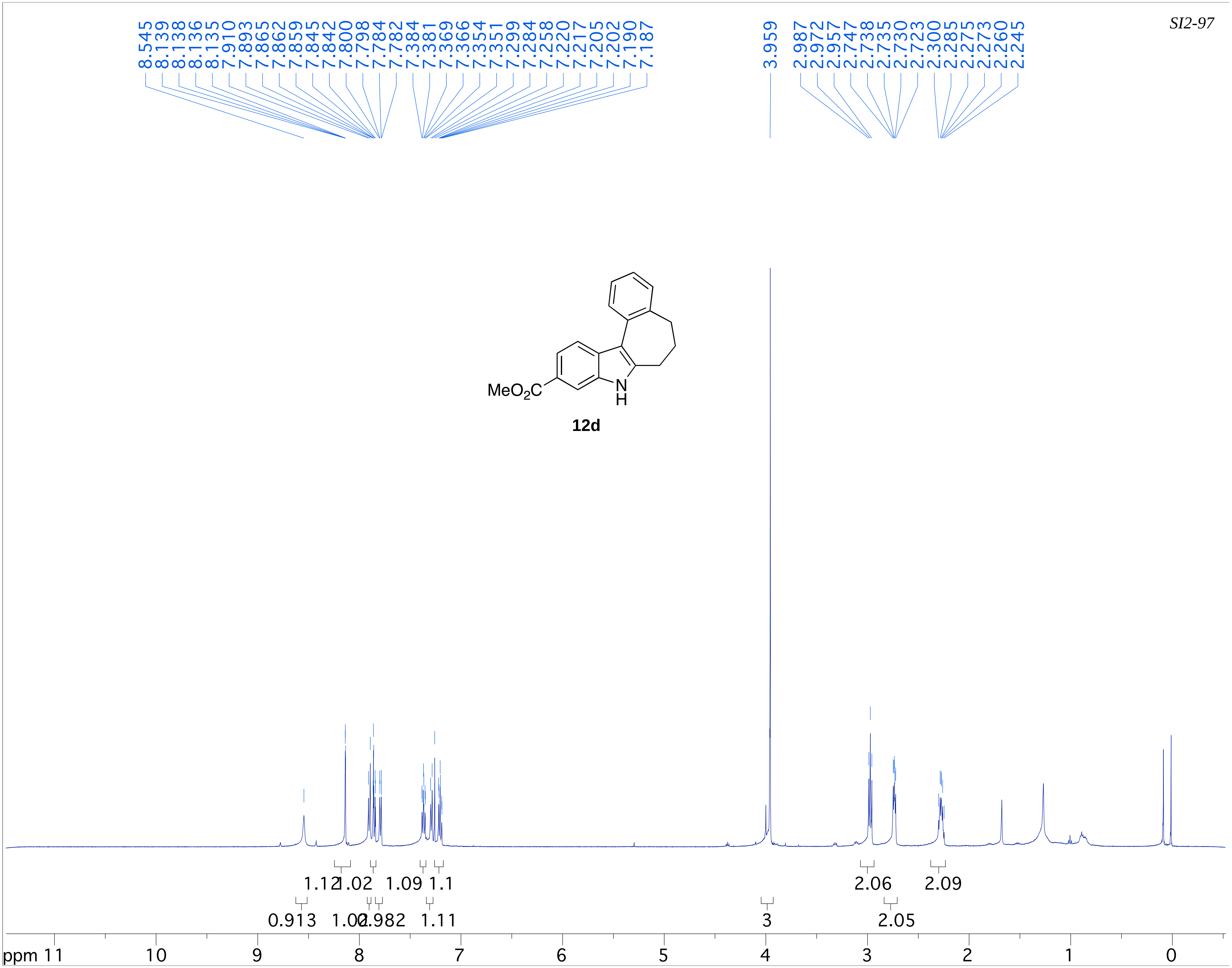
SI2-97
8.545
8.139
8.138
8.136
8.135
7.910
7.893
7.865
7.862
7.859
7.845
7.842
7.800
7.798
7.784
7.782
7.384
7.381
7.369
7.366
7.354
7.351
7.299
7.284
7.258
7.220
7.217
7.205
7.202
7.190
7.187
3.959
2.987
2.972
2.957
2.747
2.738
2.735
2.730
2.723
2.300
2.285
2.275
2.273
2.260
2.245
MeO2C
N
H
12d
1.12
1.02
1.09
1.1
2.06
2.09
0.913
1.00
0.982
1.11
3
2.05
ppm 11
10
9
8
7
6
5
4
3
2
1
0

SI2-98

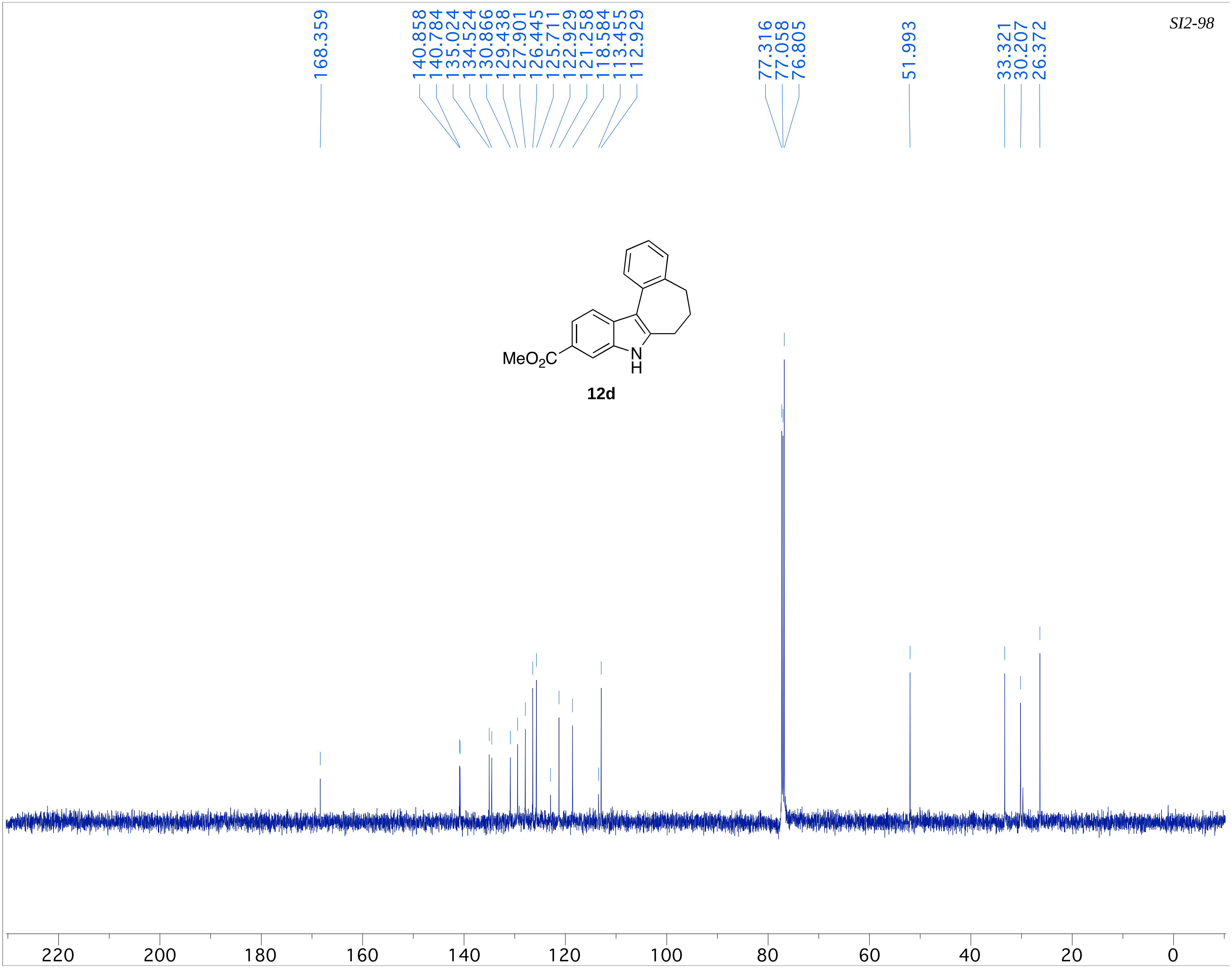
168.359
140.858
140.784
135.024
134.524
130.866
129.438
127.901
126.445
125.711
122.929
121.258
118.584
113.455
112.929
77.316
77.058
76.805
51.993
33.321
30.207
26.372
MeO2C
N
H
12d
220
200
180
160
140
120
100
80
60
40
20
0

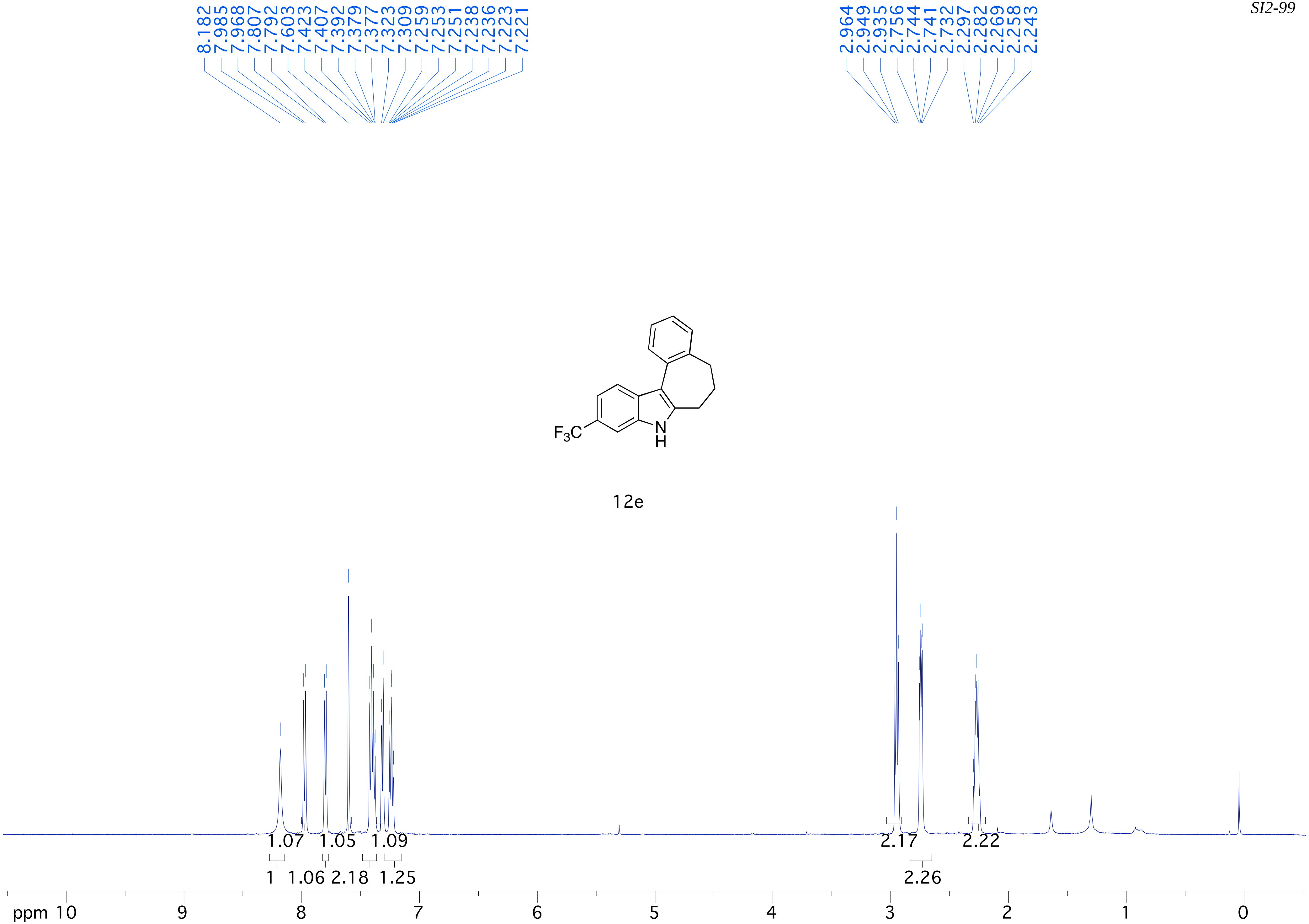
F3C
N
H
12e
8.182
7.985
7.968
7.807
7.792
7.603
7.423
7.407
7.392
7.379
7.377
7.323
7.309
7.259
7.253
7.251
7.238
7.236
7.223
7.221
2.964
2.949
2.935
2.756
2.744
2.741
2.732
2.297
2.282
2.269
2.258
2.243
1.07
1.05
1.09
2.17
2.22
1
1.06
2.18
1.25
2.26
ppm 10
9
8
7
6
5
4
3
2
1
0

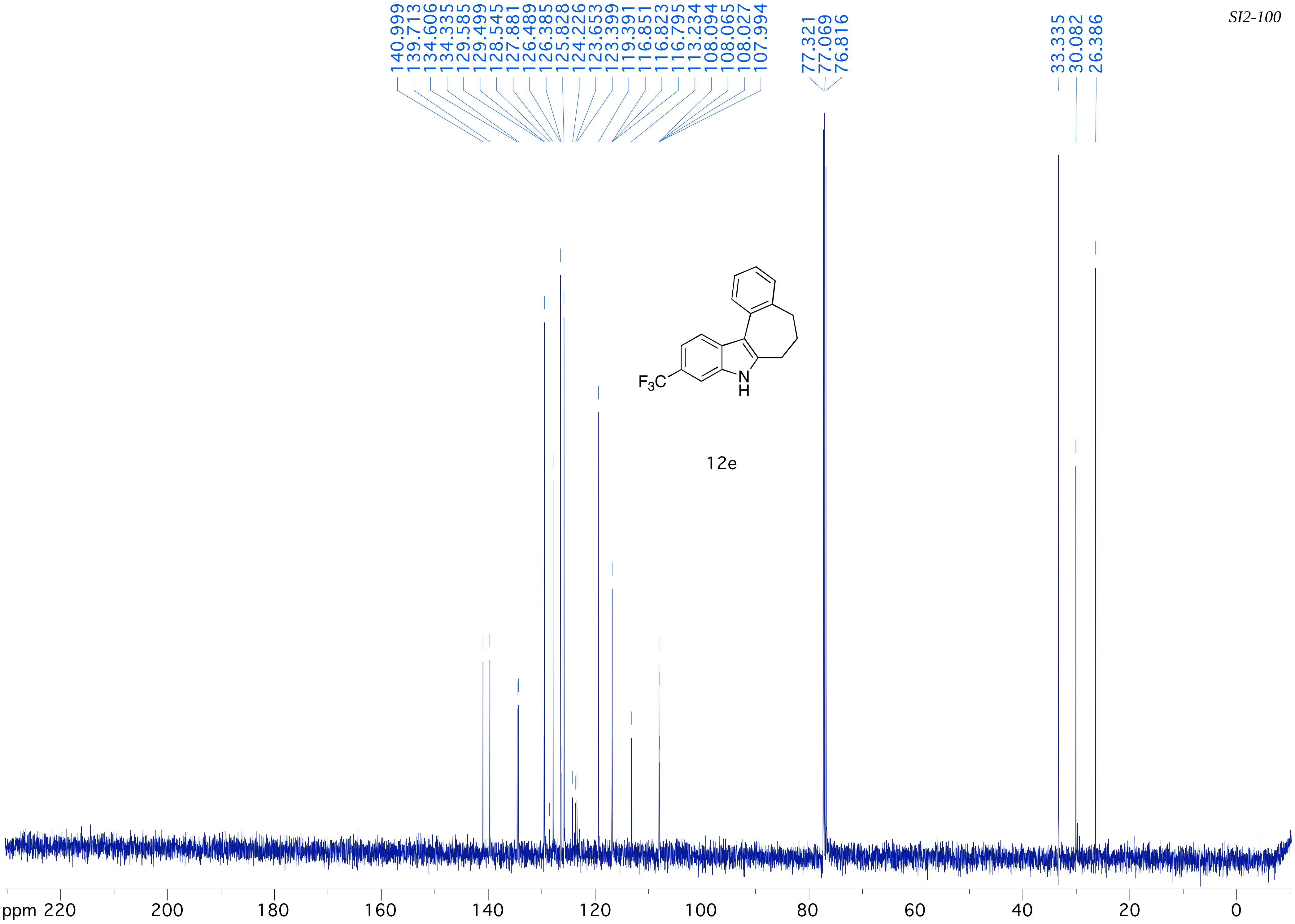
140.999
139.713
134.606
134.335
129.585
129.499
128.545
127.881
126.489
126.385
125.828
124.226
123.653
123.399
119.391
116.851
116.823
116.795
113.234
108.094
108.065
108.027
107.994
77.321
77.069
76.816
33.335
30.082
26.386
ppm 220
200
180
160
140
120
100
80
60
40
20
0
F3C
N
H

12e

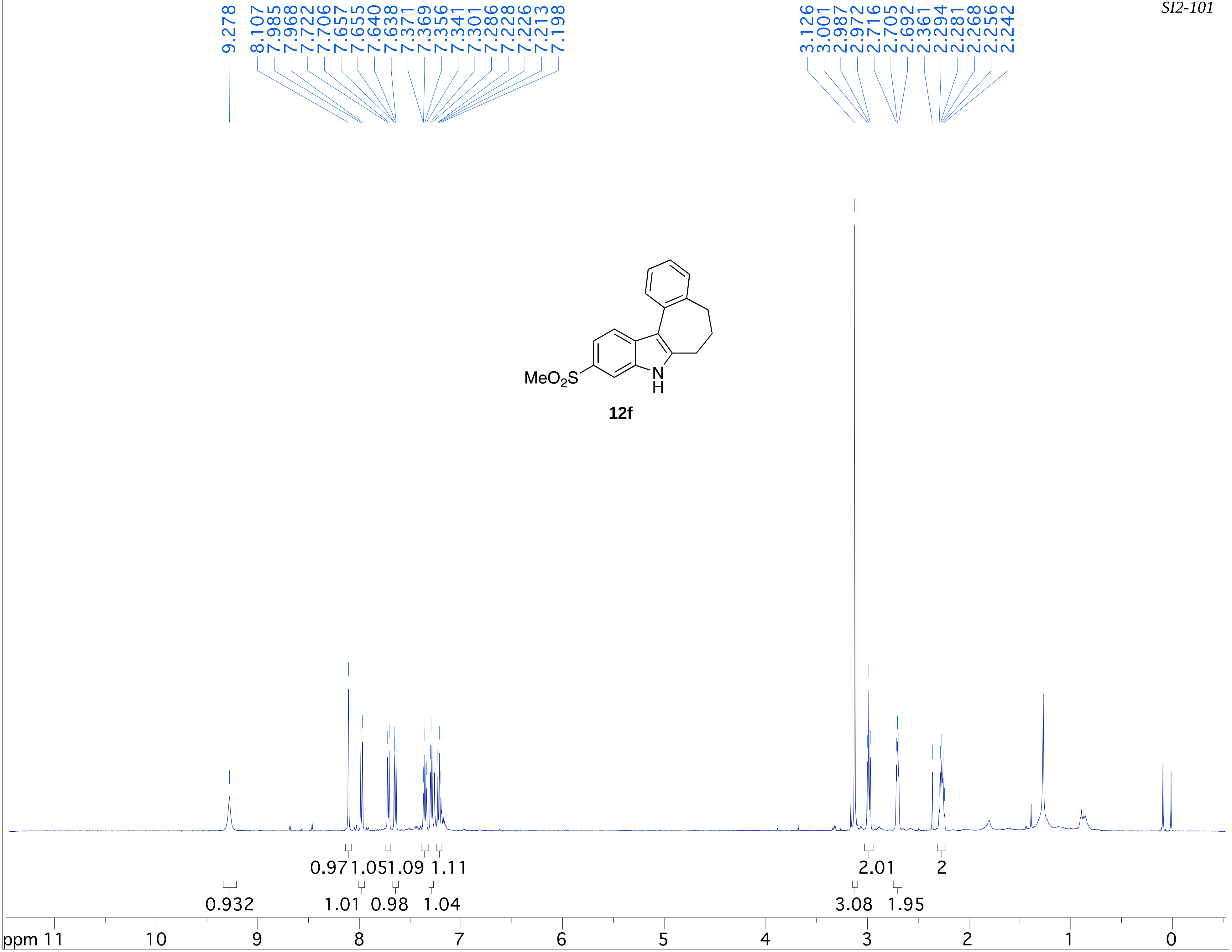
9.278
8.107
7.985
7.968
7.722
7.706
7.657
7.655
7.640
7.638
7.371
7.369
7.356
7.341
7.301
7.286
7.228
7.226
7.213
7.198
3.126
3.001
2.987
2.972
2.716
2.705
2.692
2.361
2.294
2.281
2.268
2.256
2.242
MeO2S
N
H
12f
0.97
1.05
1.09
1.11
2.01
2
0.932
1.01
0.98
1.04
3.08
1.95
ppm 11
10
9
8
7
6
5
4
3
2
1
0

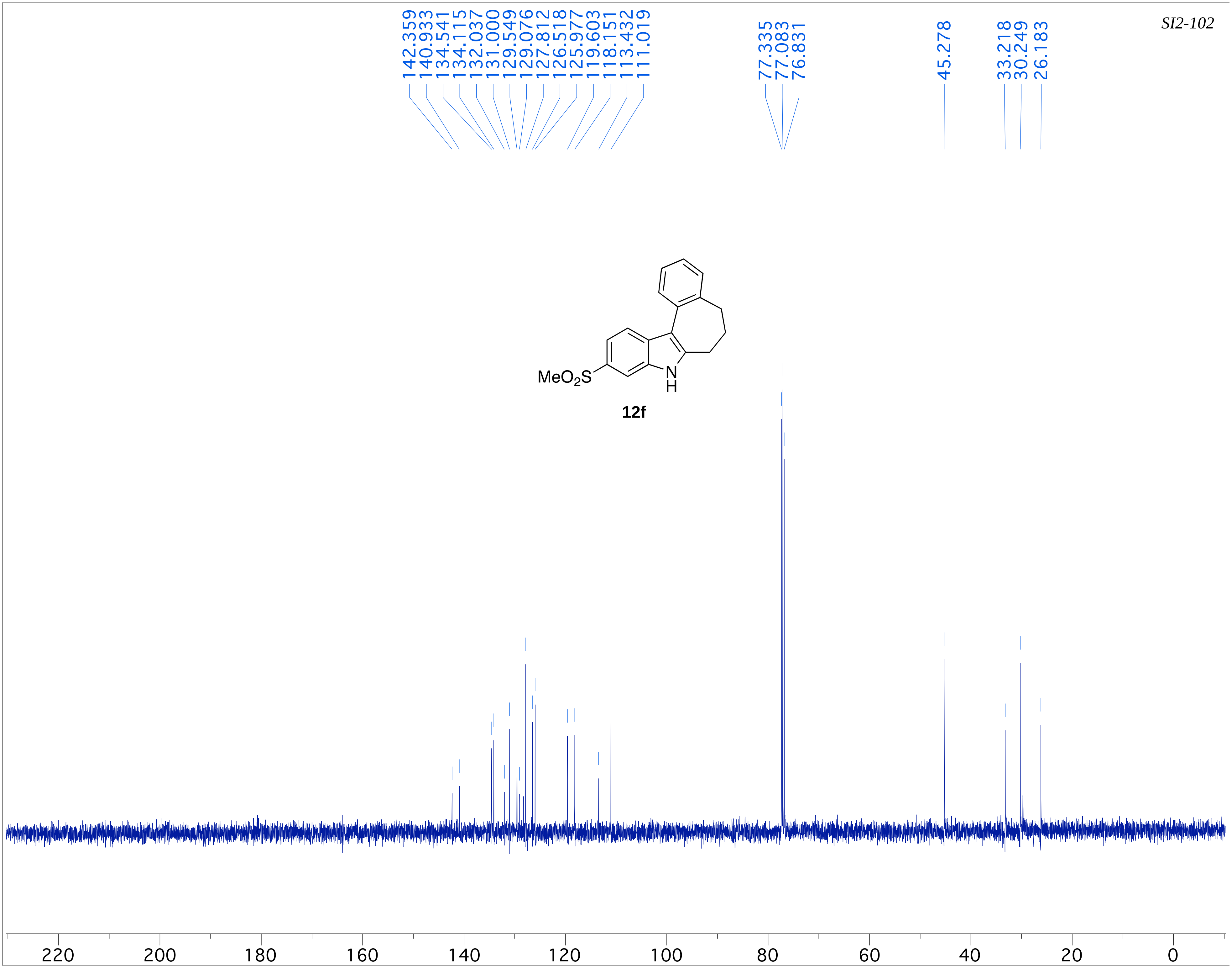
142.359
140.933
134.541
134.115
132.037
131.000
129.549
129.076
127.812
126.518
125.977
119.603
118.151
113.432
111.019
77.335
77.083
76.831
45.278
33.218
30.249
26.183
MeO2S
N
H
12f
220
200
180
160
140
120
100
80
60
40
20
0

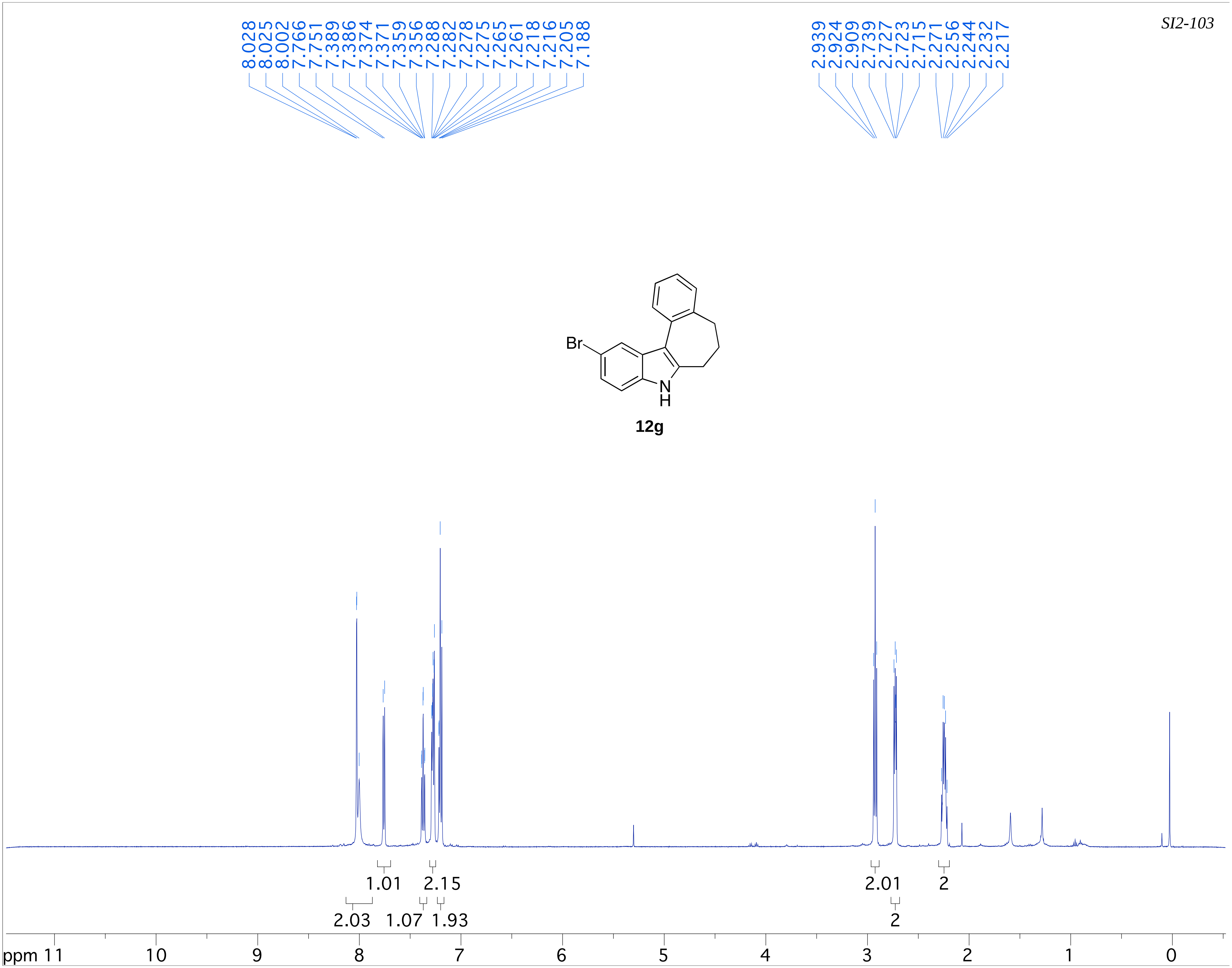
8.028
8.025
8.002
7.766
7.751
7.389
7.386
7.374
7.371
7.359
7.356
7.288
7.282
7.278
7.275
7.265
7.261
7.218
7.216
7.205
7.188
2.939
2.924
2.909
2.739
2.727
2.723
2.715
2.271
2.256
2.244
2.232
2.217
Br
N
H
12g
1.01
2.15
2.01
2
2.03
1.07
1.93
2
ppm 11
10
9
8
7
6
5
4
3
2
1
0

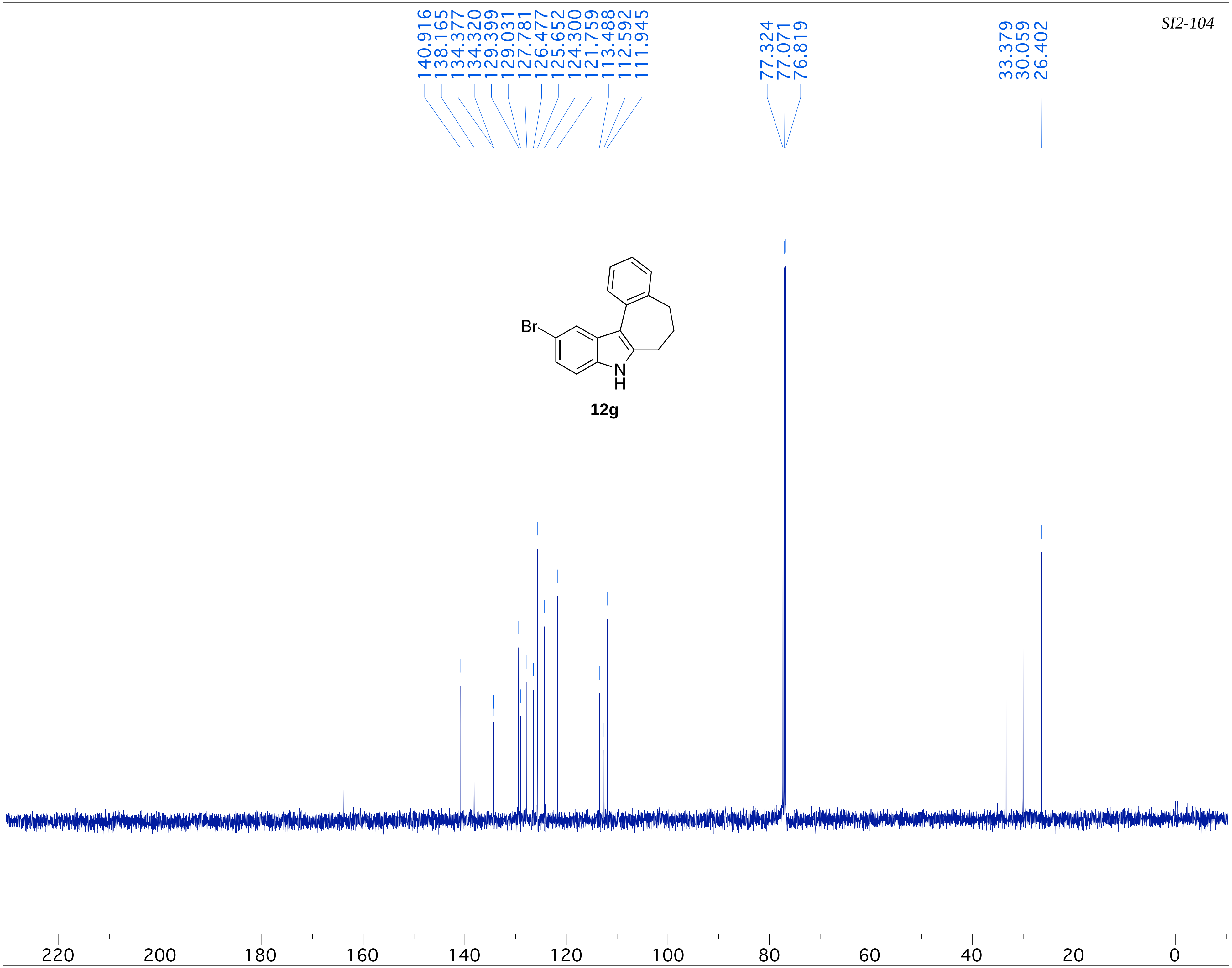
SI2-104
140.916
138.165
134.377
134.320
129.399
129.031
127.781
126.477
125.652
124.300
121.759
113.488
112.592
111.945
77.324
77.071
76.819
33.379
30.059
26.402
Br
N
H
12g
220
200
180
160
140
120
100
80
60
40
20
0

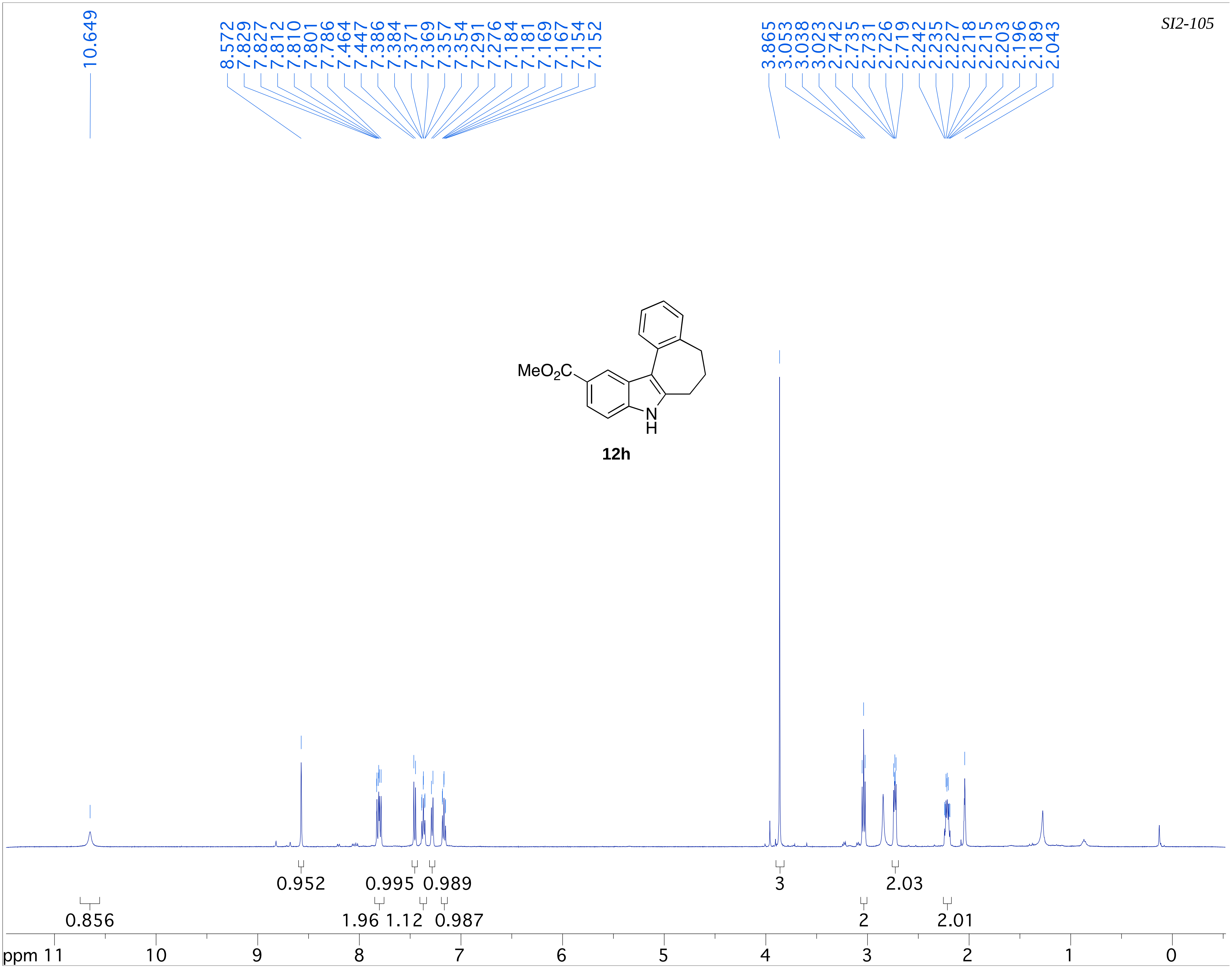

SI2-105
10.649
8.572 7.829 7.827 7.812 7.810 7.801 7.786 7.464 7.447 7.386 7.384 7.371 7.369 7.357 7.354 7.291 7.276 7.184 7.181 7.169 7.167 7.154 7.152
3.865 3.053 3.038 3.023 2.742 2.735 2.731 2.726 2.719 2.242 2.235 2.227 2.218 2.215 2.203 2.196 2.189 2.043
MeO2C
N
H
12h
0.952 0.995 0.989 3 2.03
0.856 1.96 1.12 0.987 2 2.01
ppm 11 10 9 8 7 6 5 4 3 2 1 0

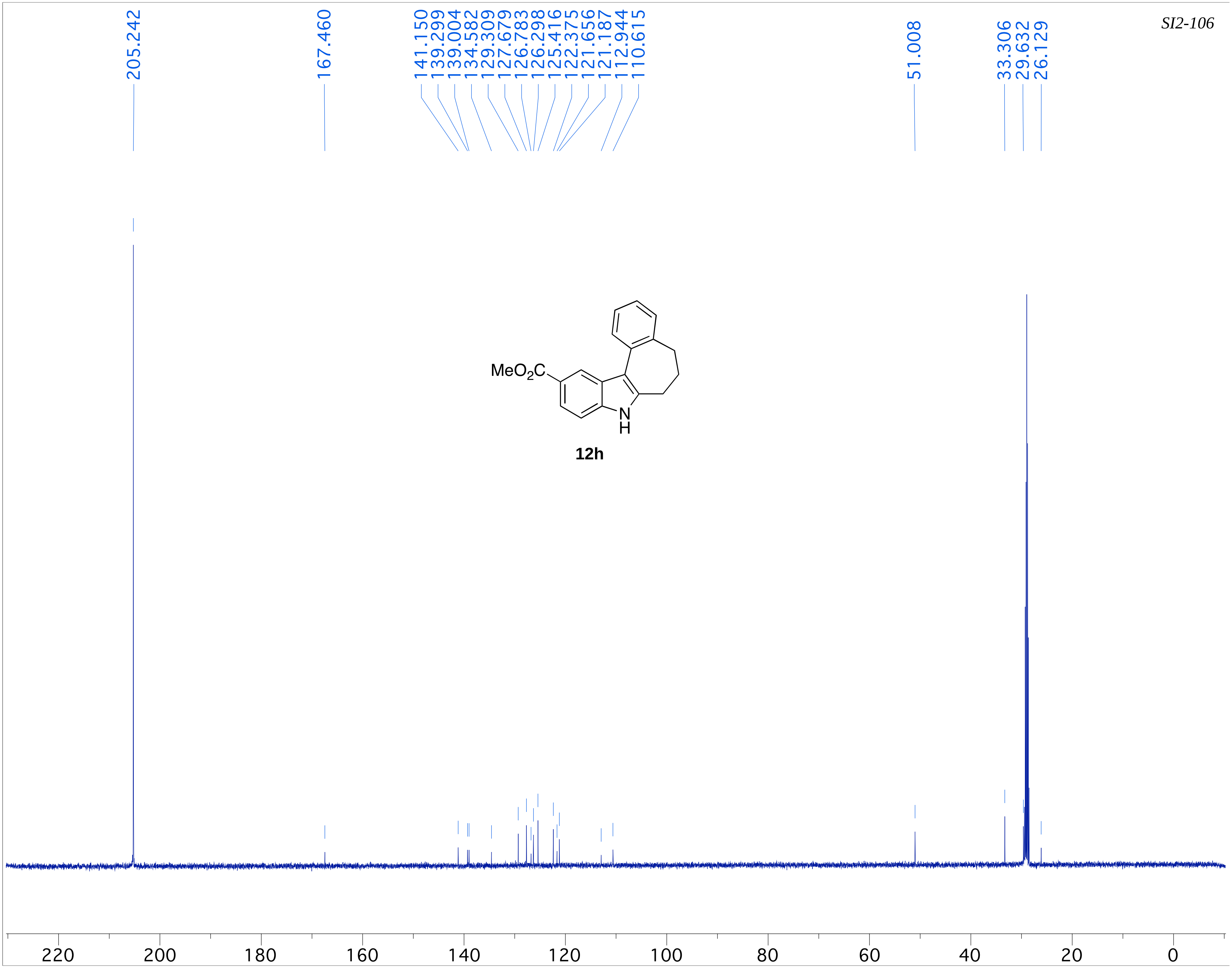

205.242
167.460
141.150
139.299
139.004
134.582
129.309
127.679
126.783
126.298
125.416
122.375
121.656
121.187
112.944
110.615
51.008
33.306
29.632
26.129
SI2-106
MeO2C
N
H
12h
220
200
180
160
140
120
100
80
60
40
20
0

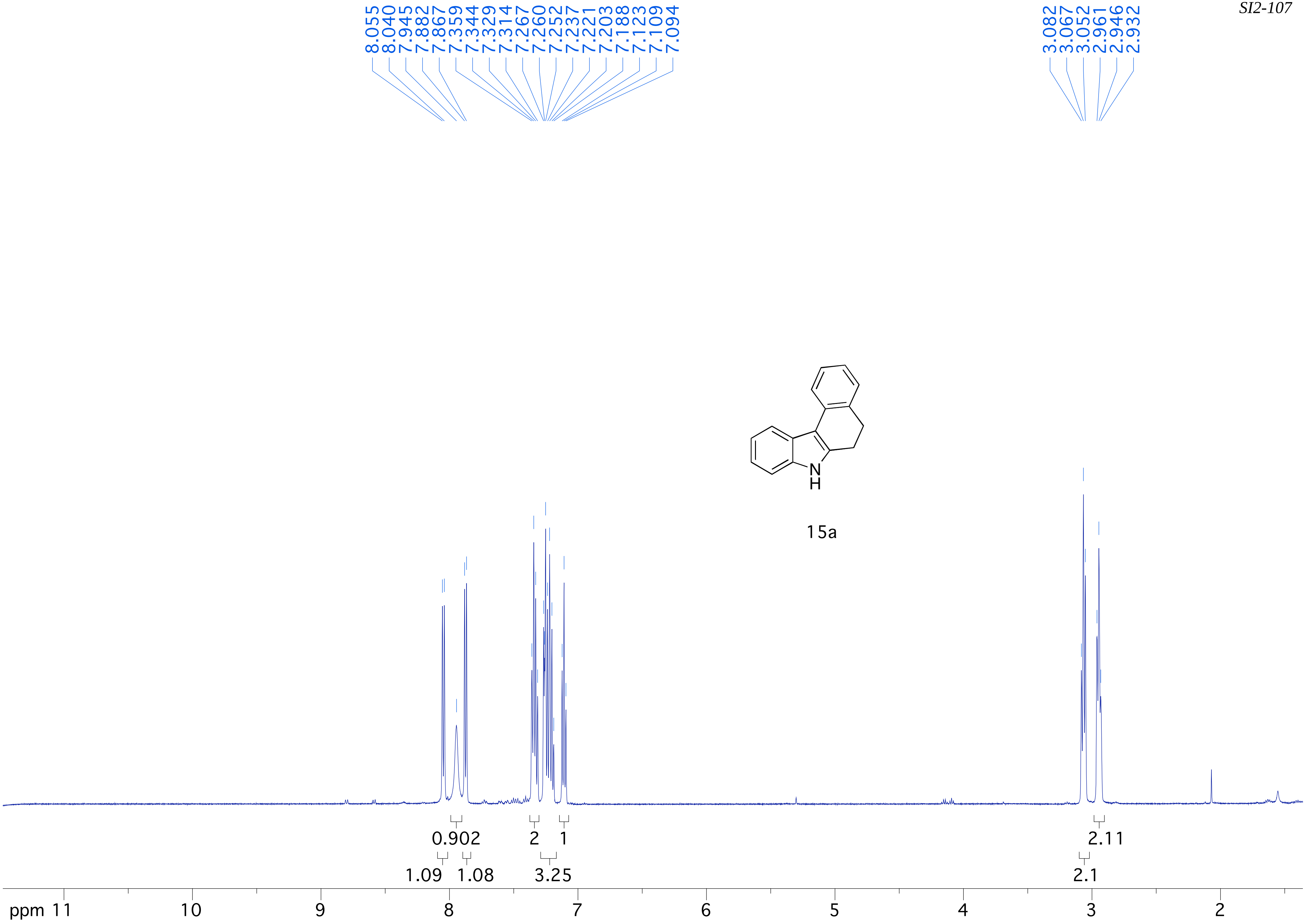
8.055
8.040
7.945
7.882
7.867
7.359
7.344
7.329
7.314
7.267
7.260
7.252
7.237
7.221
7.203
7.188
7.123
7.109
7.094
3.082
3.067
3.052
2.961
2.946
2.932
N
H
15a
0.902
2
1
2.11
1.09
1.08
3.25
2.1
ppm 11
10
9
8
7
6
5
4
3
2

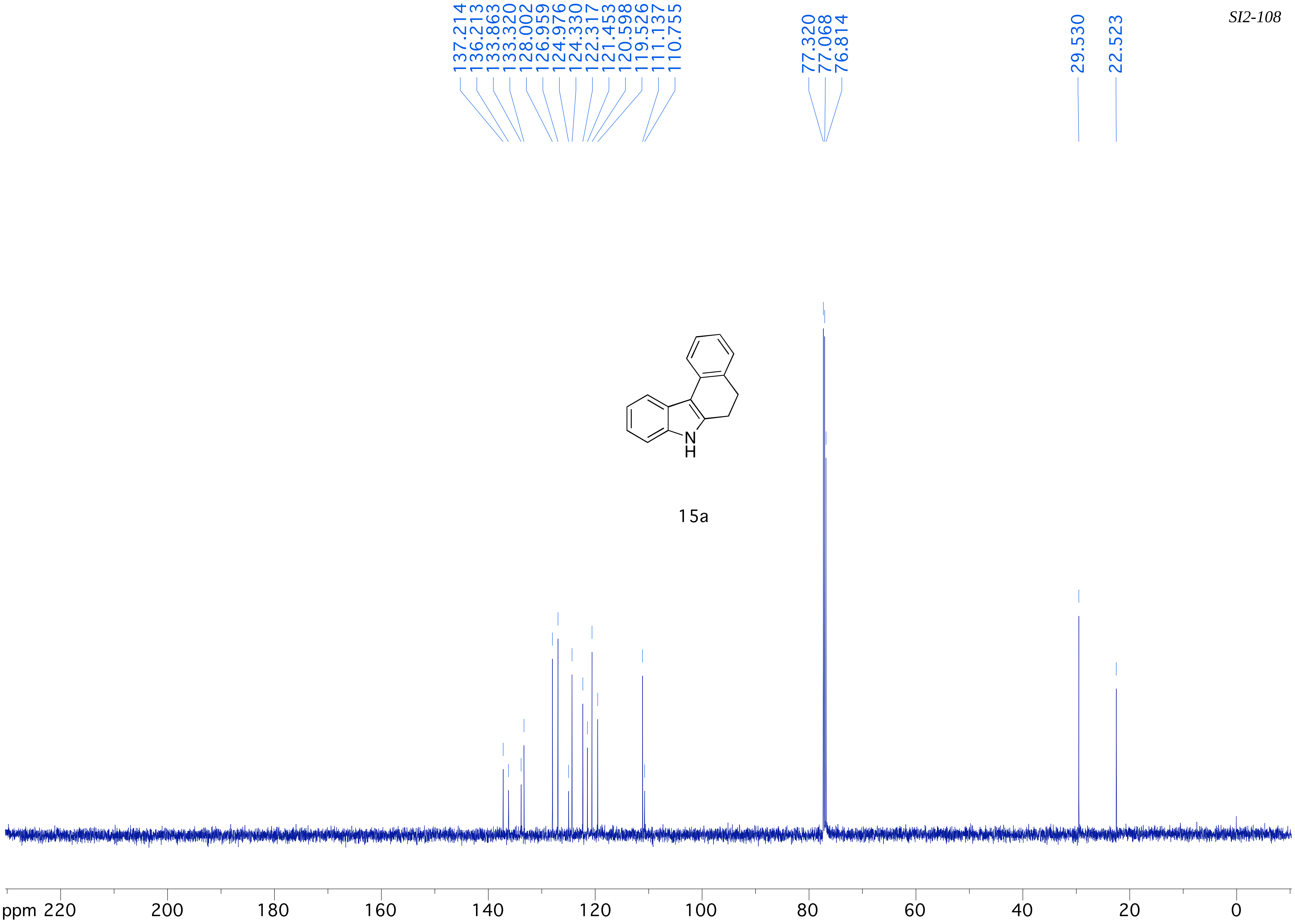
137.214
136.213
133.863
133.320
128.002
126.959
124.976
124.330
122.317
121.453
120.598
119.526
111.137
110.755
77.320
77.068
76.814
29.530
22.523
N
H
15a
ppm 220
200
180
160
140
120
100
80
60
40
20
0

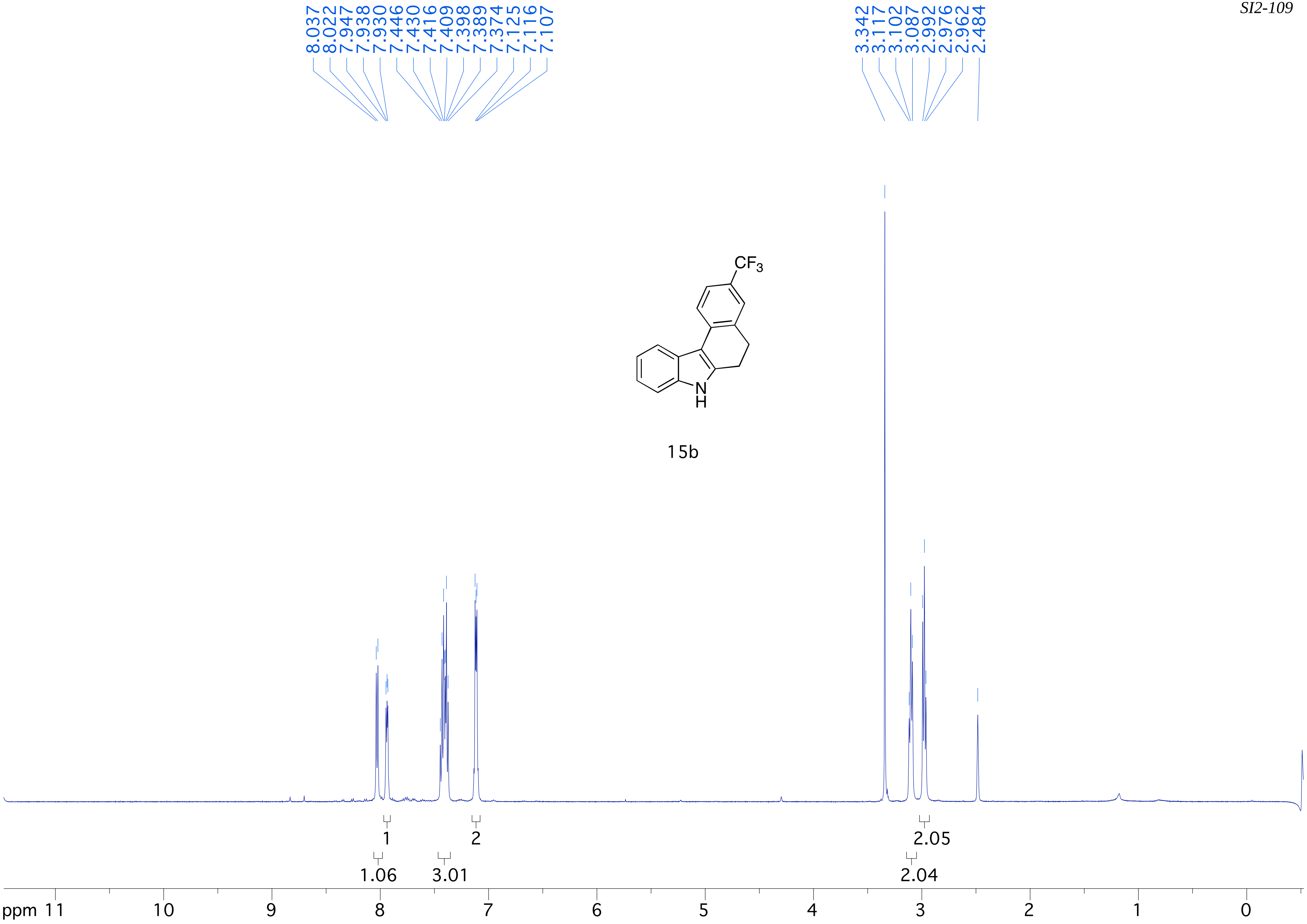

8.037
8.022
7.947
7.938
7.930
7.446
7.430
7.416
7.409
7.398
7.389
7.374
7.125
7.116
7.107
3.342
3.117
3.102
3.087
2.992
2.976
2.962
2.484
CF3
N
H
15b
1
2
2.05
1.06
3.01
2.04
ppm 11
10
9
8
7
6
5
4
3
2
1
0

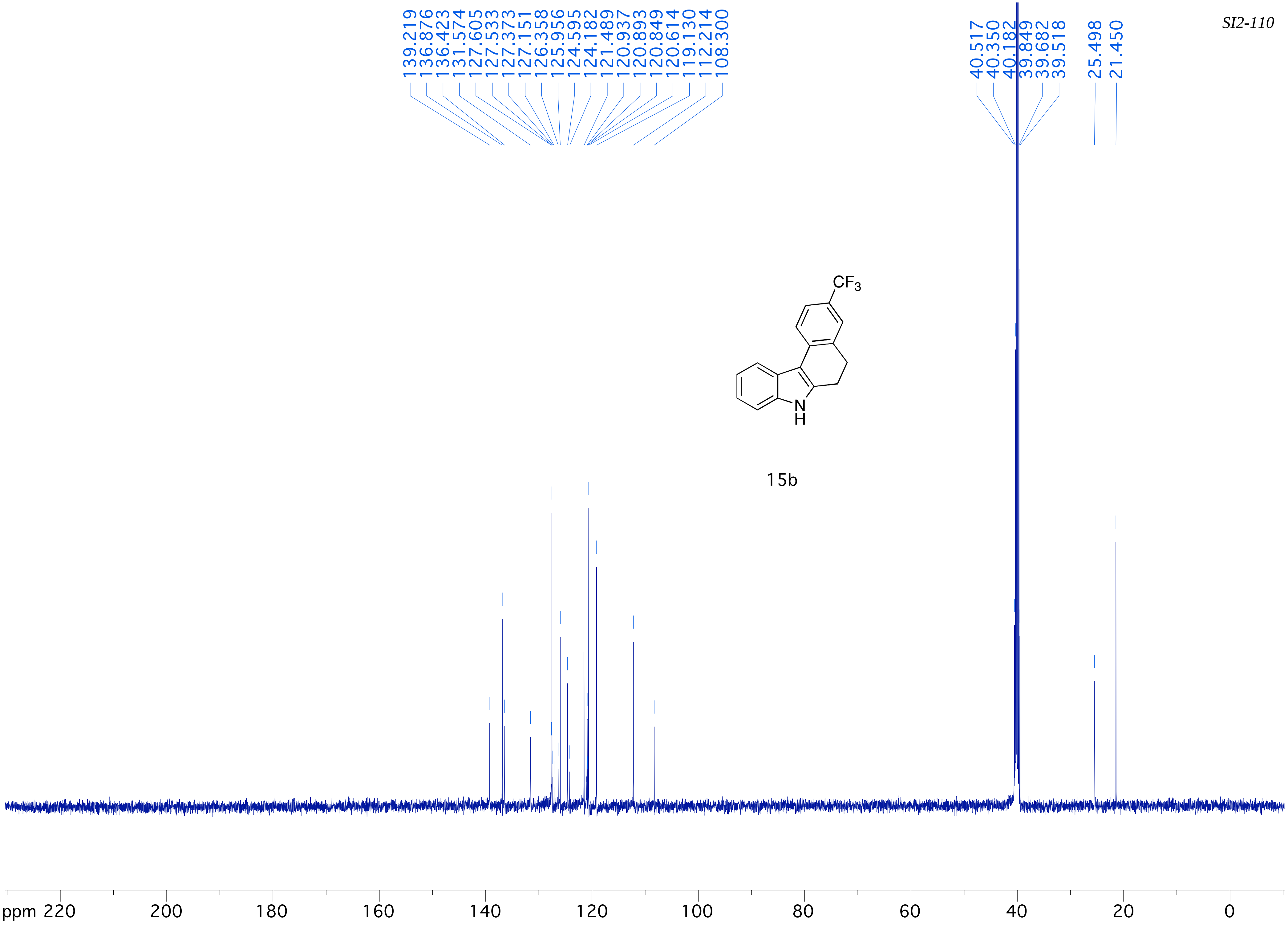

139.219
136.876
136.423
131.574
127.605
127.533
127.373
127.151
126.358
125.956
124.595
124.182
121.489
120.937
120.893
120.849
120.614
119.130
112.214
108.300
40.517
40.350
40.182
39.849
39.682
39.518
25.498
21.450
CF3
N
H
15b
ppm 220
200
180
160
140
120
100
80
60
40
20
0

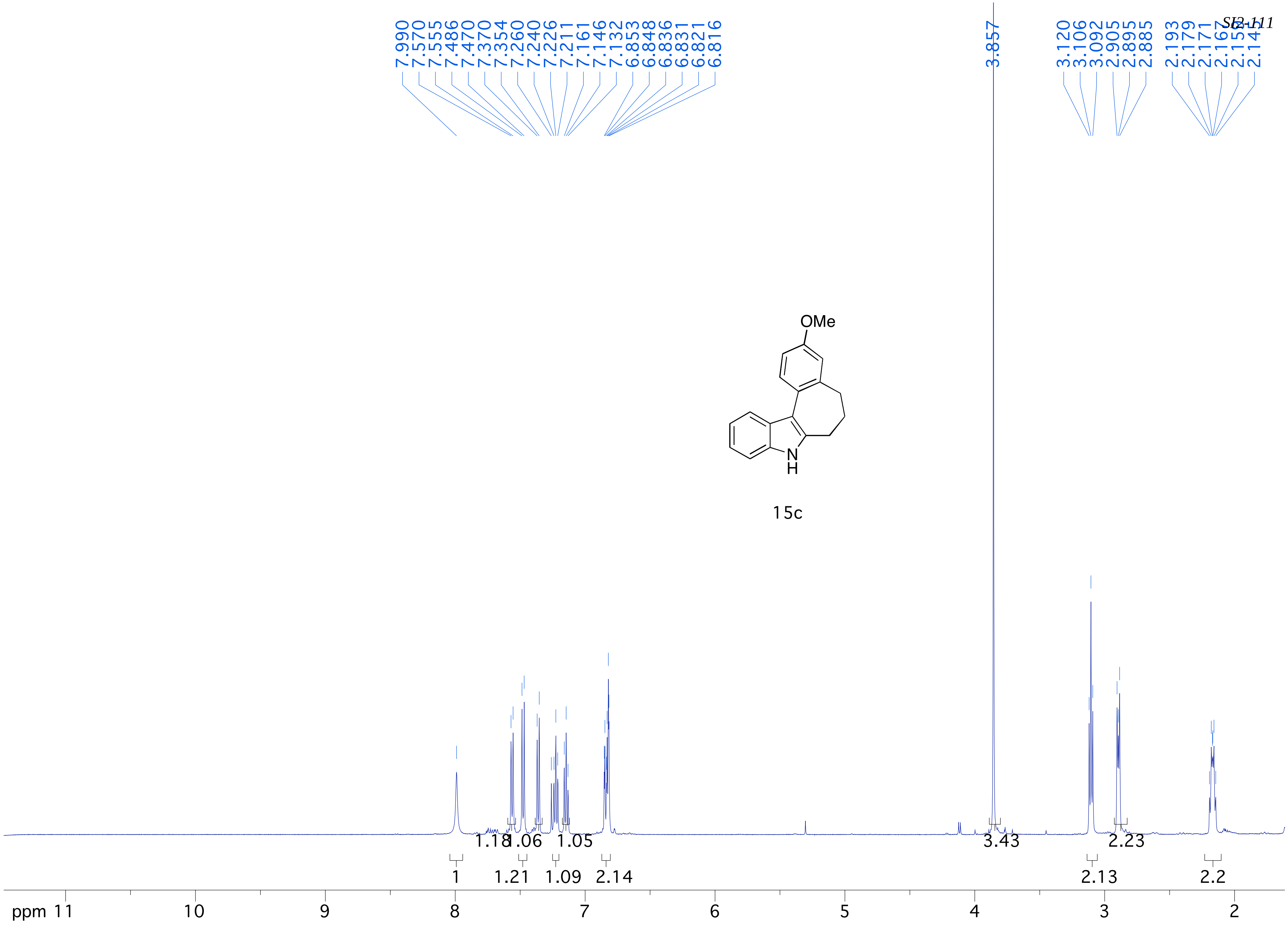
7.990
7.570
7.555
7.486
7.470
7.370
7.354
7.260
7.240
7.226
7.211
7.161
7.146
7.132
6.853
6.848
6.836
6.831
6.821
6.816
3.857
3.120
3.106
3.092
2.905
2.895
2.885
2.193
2.179
2.171
2.167
2.153
2.14
OMe
N
H
15c
1.18
1.06
1.05
3.43
2.23
1
1.21
1.09
2.14
2.13
2.2
ppm 11
10
9
8
7
6
5
4
3
2

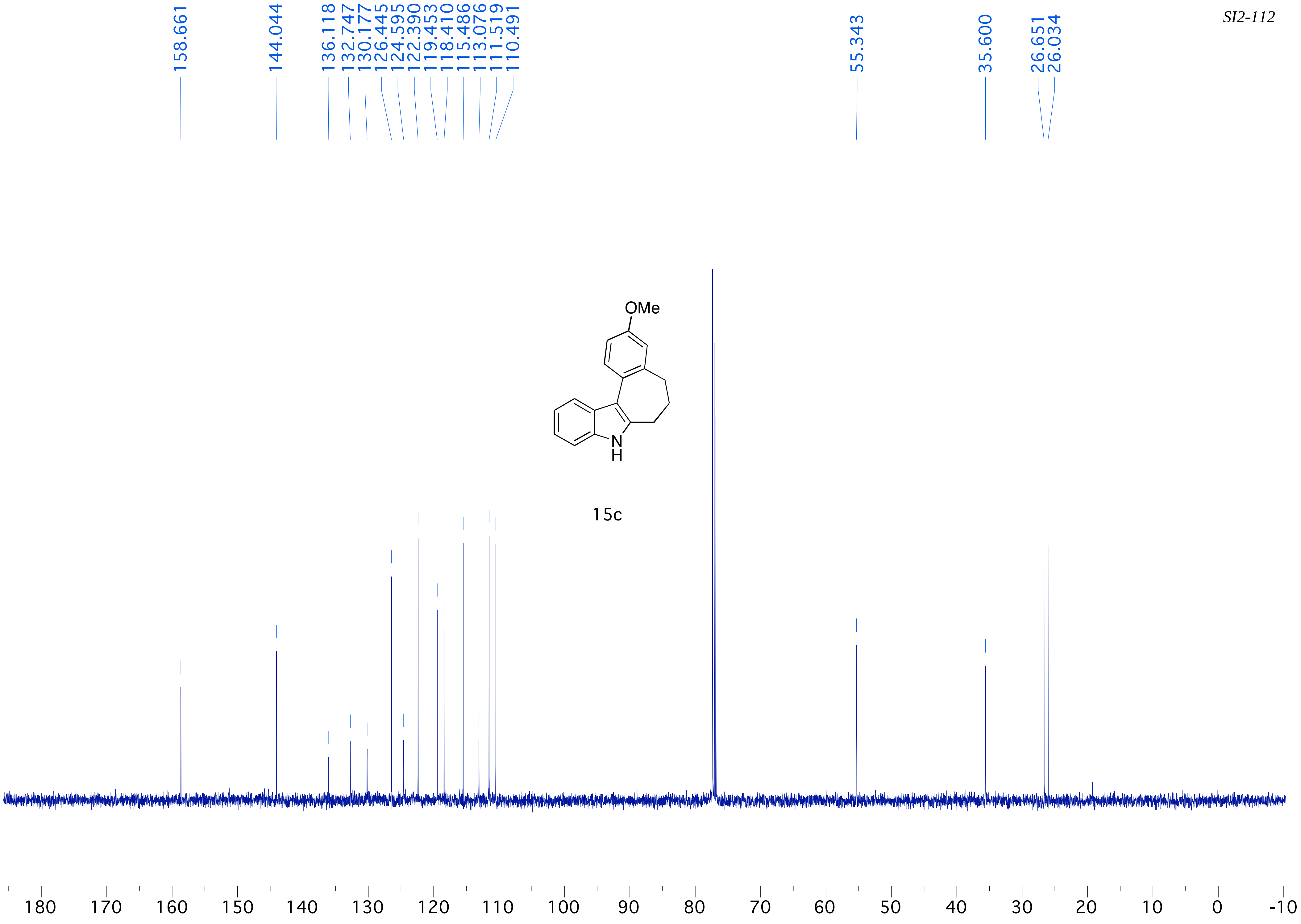
158.661
144.044
136.118
132.747
130.177
126.445
124.595
122.390
119.453
118.410
115.486
113.076
111.519
110.491
55.343
35.600
26.651
26.034
OMe
N
H
15c
180 170 160 150 140 130 120 110 100 90 80 70 60 50 40 30 20 10 0 -10

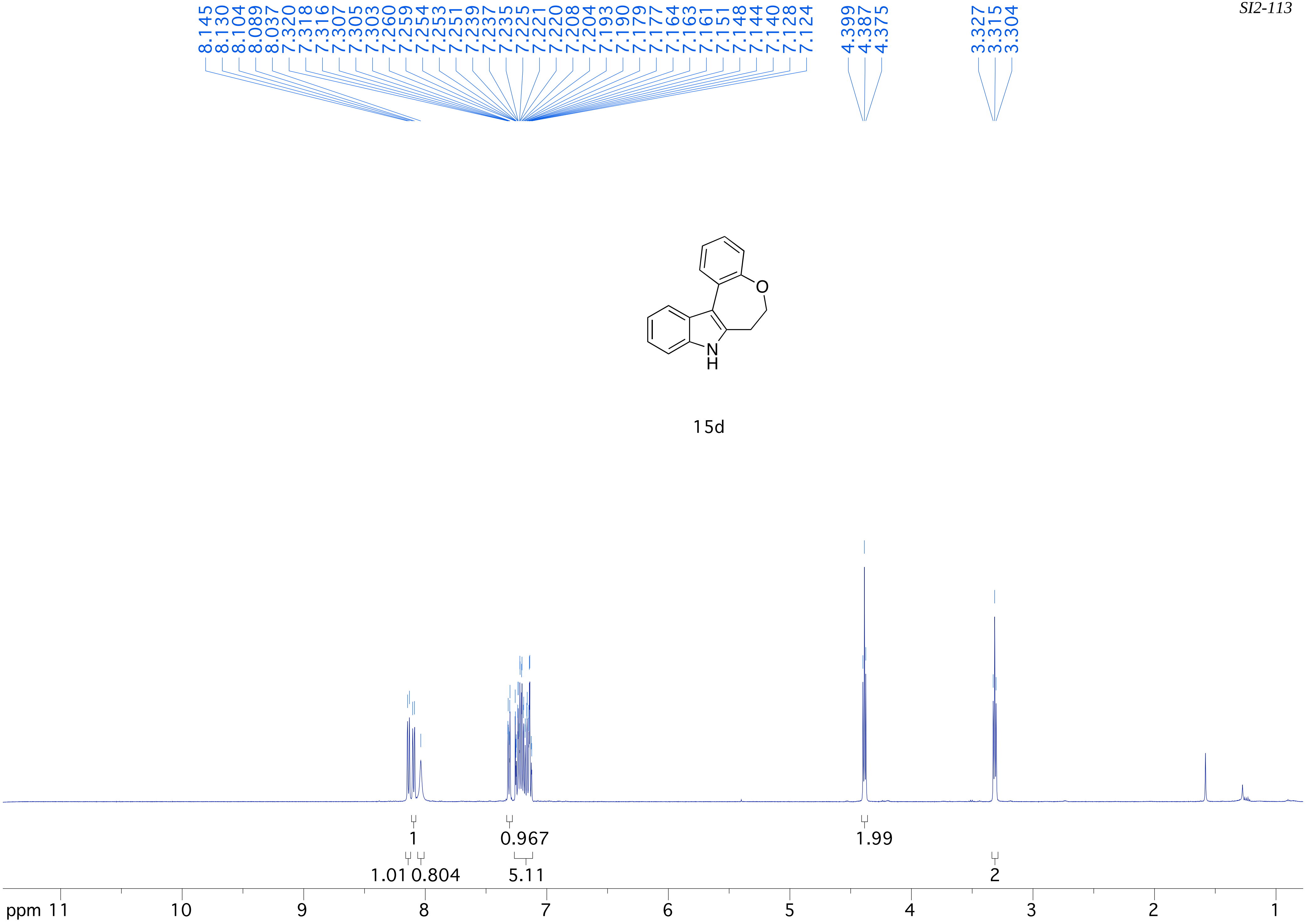
8.145
8.130
8.104
8.089
8.037
7.320
7.318
7.316
7.307
7.305
7.303
7.260
7.259
7.254
7.253
7.251
7.239
7.237
7.235
7.225
7.221
7.220
7.208
7.204
7.193
7.190
7.179
7.177
7.164
7.163
7.161
7.151
7.148
7.144
7.140
7.128
7.124
4.399
4.387
4.375
3.327
3.315
3.304
O
N
H
15d
1
0.967
1.99
1.01
0.804
5.11
2
ppm 11
10
9
8
7
6
5
4
3
2
1

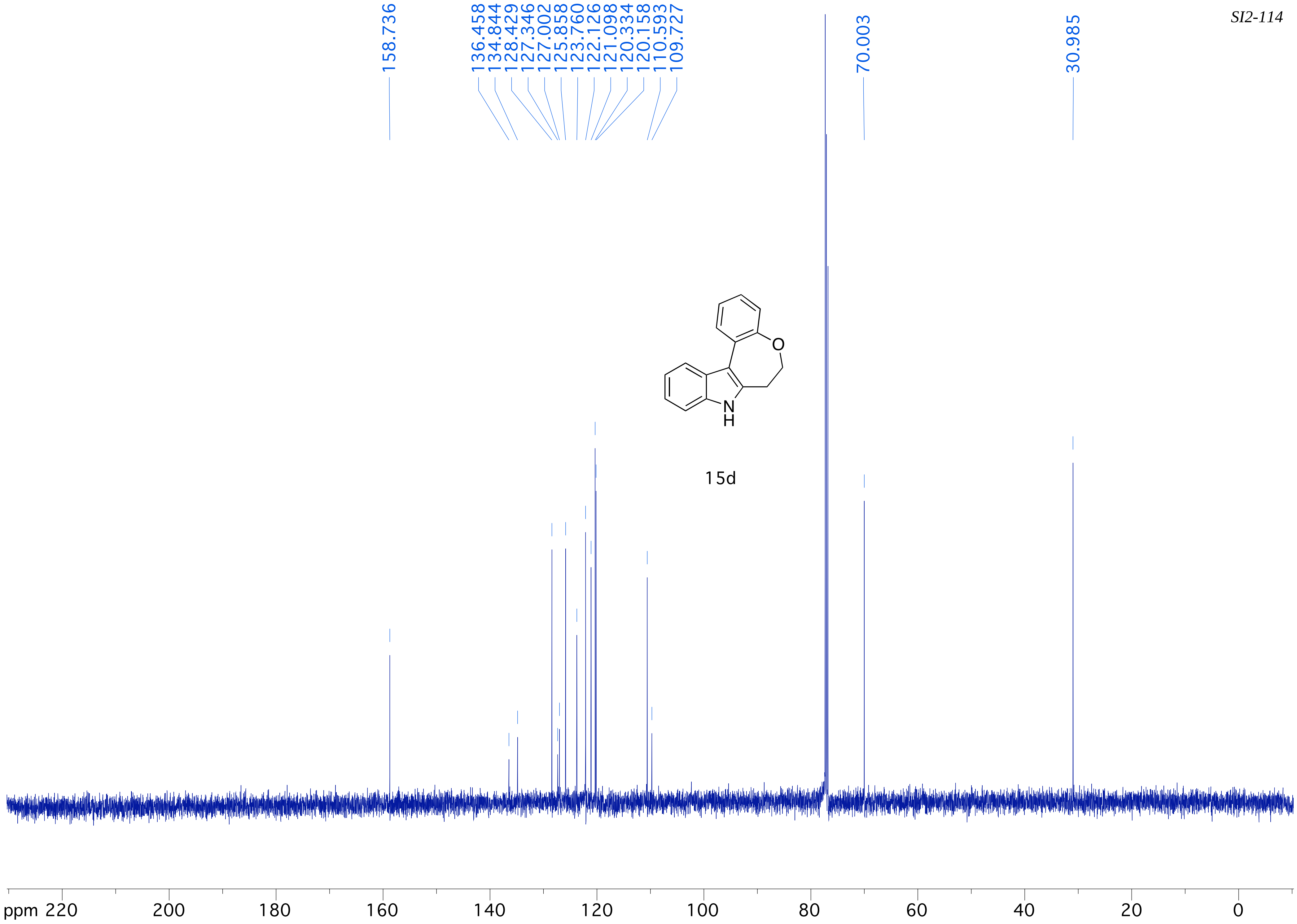
158.736
136.458
134.844
128.429
127.346
127.002
125.858
123.760
122.126
121.098
120.334
120.158
110.593
109.727
70.003
30.985
O
N
H
15d
ppm 220
200
180
160
140
120
100
80
60
40
20
0

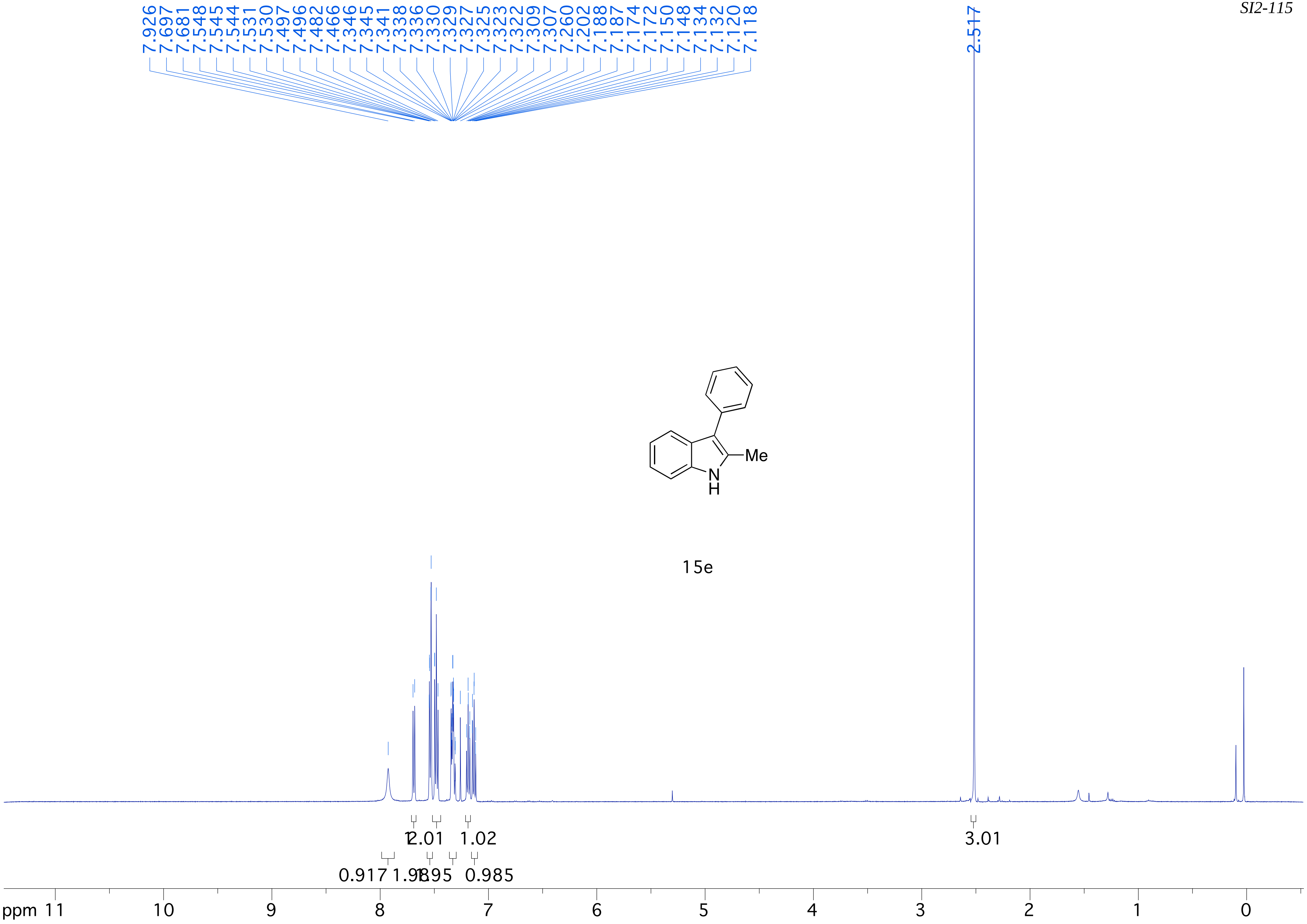
7.926
7.697
7.681
7.548
7.545
7.544
7.531
7.530
7.497
7.496
7.482
7.466
7.346
7.345
7.341
7.338
7.336
7.330
7.329
7.327
7.325
7.323
7.322
7.309
7.307
7.260
7.202
7.188
7.187
7.174
7.172
7.150
7.148
7.134
7.132
7.120
7.118
2.517
Me
N
H
15e
1.02
3.01
0.917
0.985
ppm 11
10
9
8
7
6
5
4
3
2
1
0

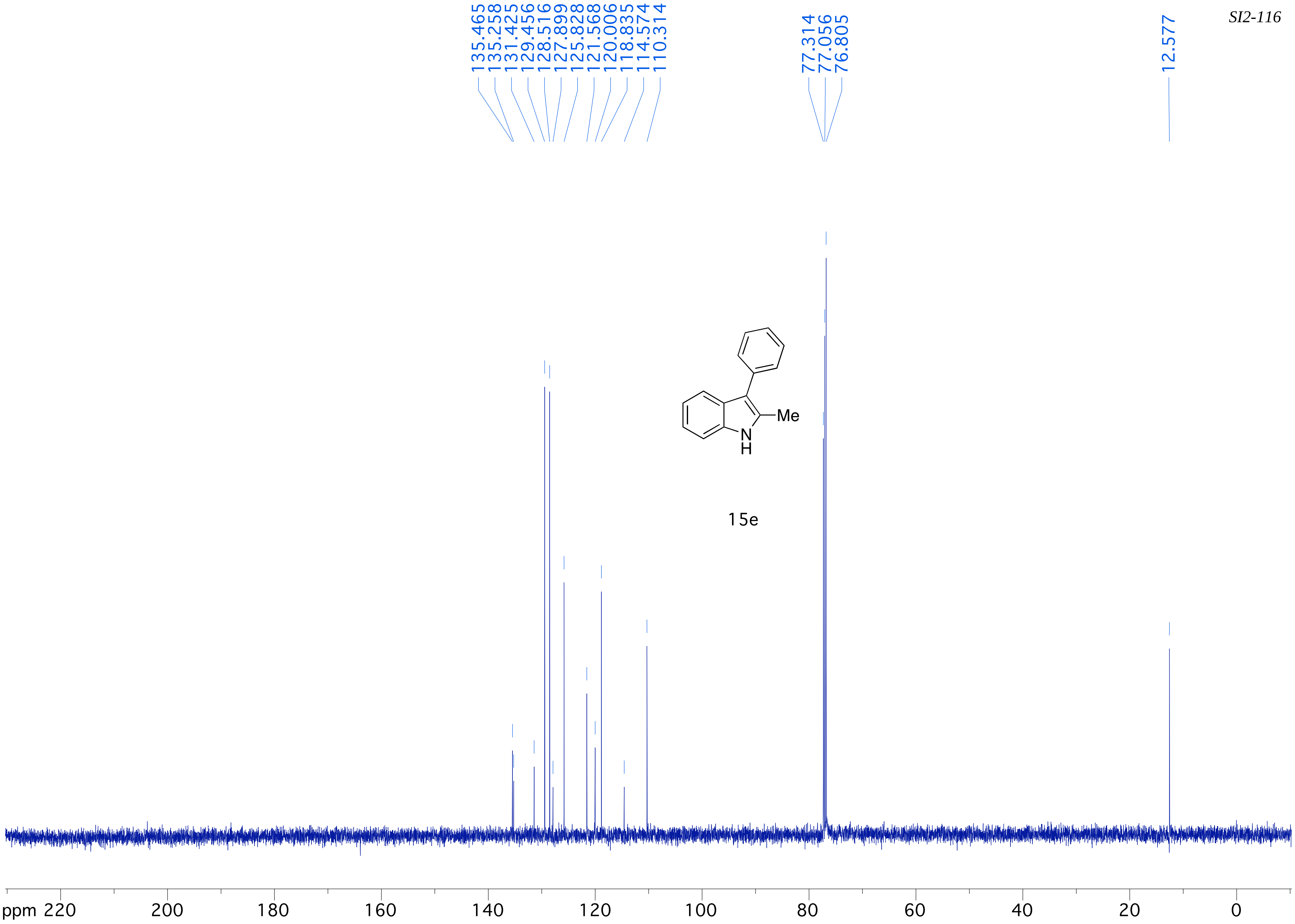
135.465
135.258
131.425
129.456
128.516
127.899
125.828
121.568
120.006
118.835
114.574
110.314
77.314
77.056
76.805
12.577
Me
N
H
15e
ppm 220
200
180
160
140
120
100
80
60
40
20
0

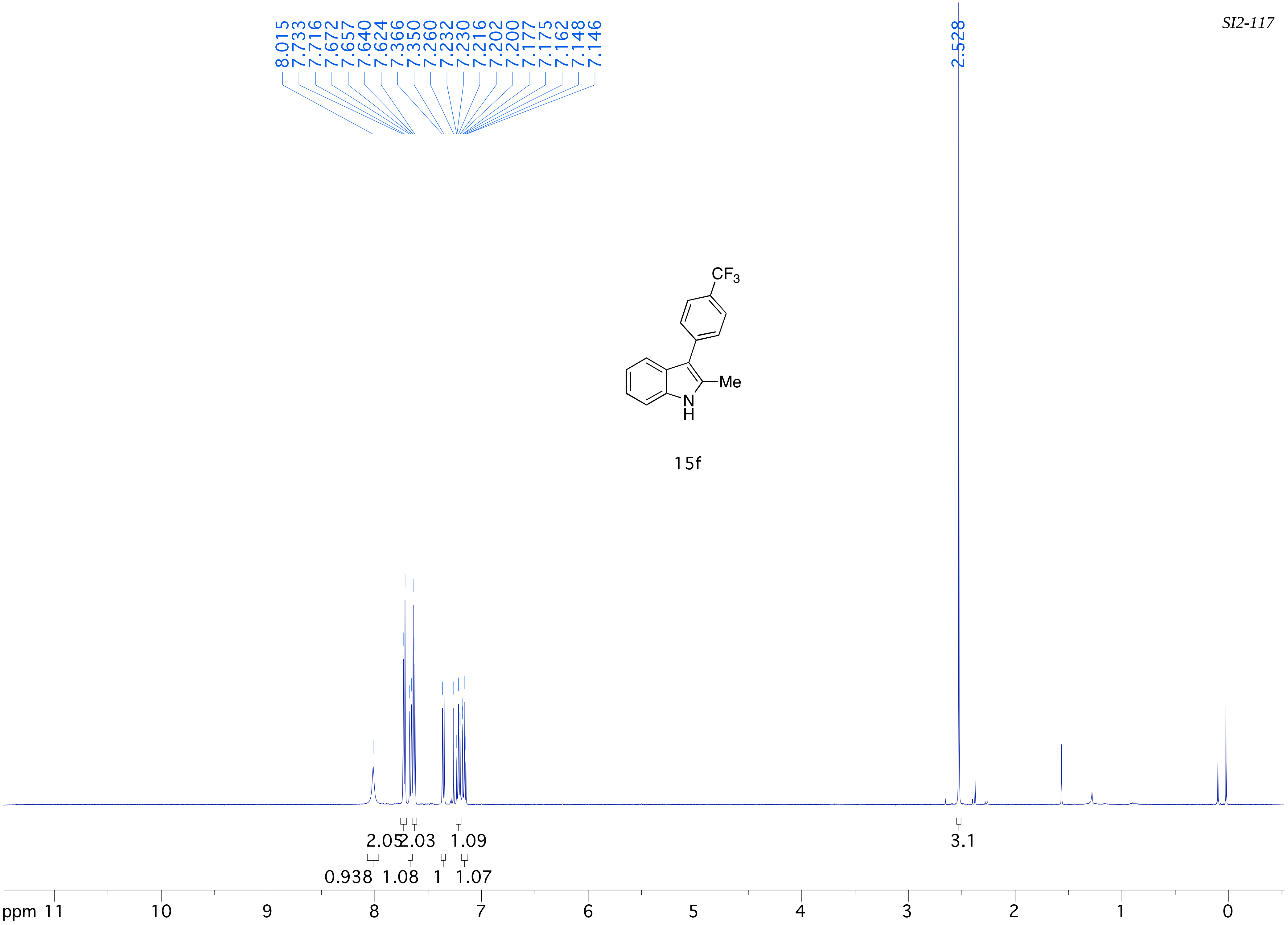
8.015
7.733
7.716
7.672
7.657
7.640
7.624
7.366
7.350
7.260
7.232
7.230
7.216
7.202
7.200
7.177
7.175
7.162
7.148
7.146
2.528
CF3
Me
N
H
15f
2.05
2.03
1.09
3.1
0.938
1.08
1
1.07
ppm 11
10
9
8
7
6
5
4
3
2
1
0

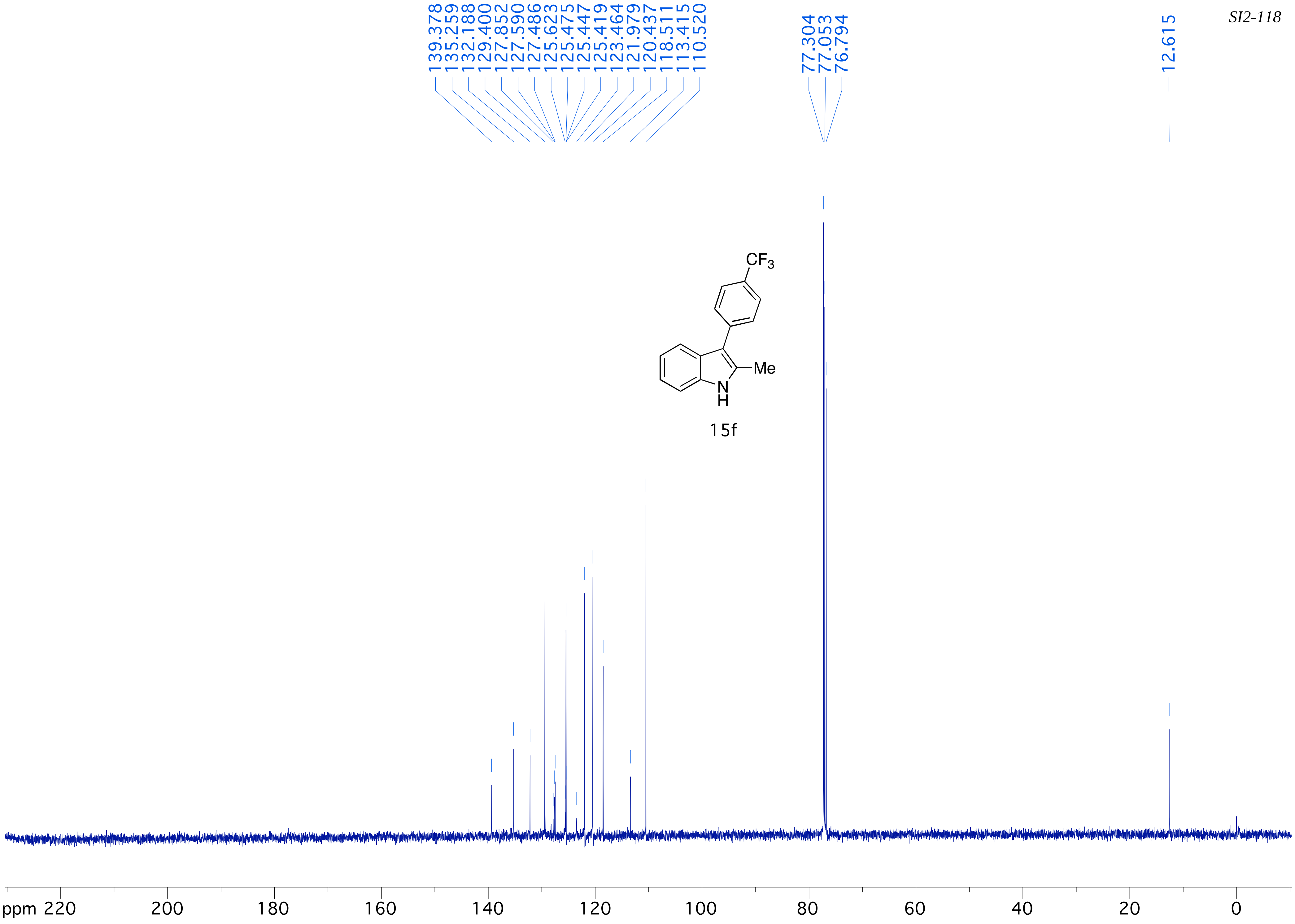
139.378
135.259
132.188
129.400
127.852
127.590
127.486
125.623
125.475
125.447
125.419
123.464
121.979
120.437
118.511
113.415
110.520
77.304
77.053
76.794
12.615
CF3
Me
N
H
15f
ppm 220
200
180
160
140
120
100
80
60
40
20
0

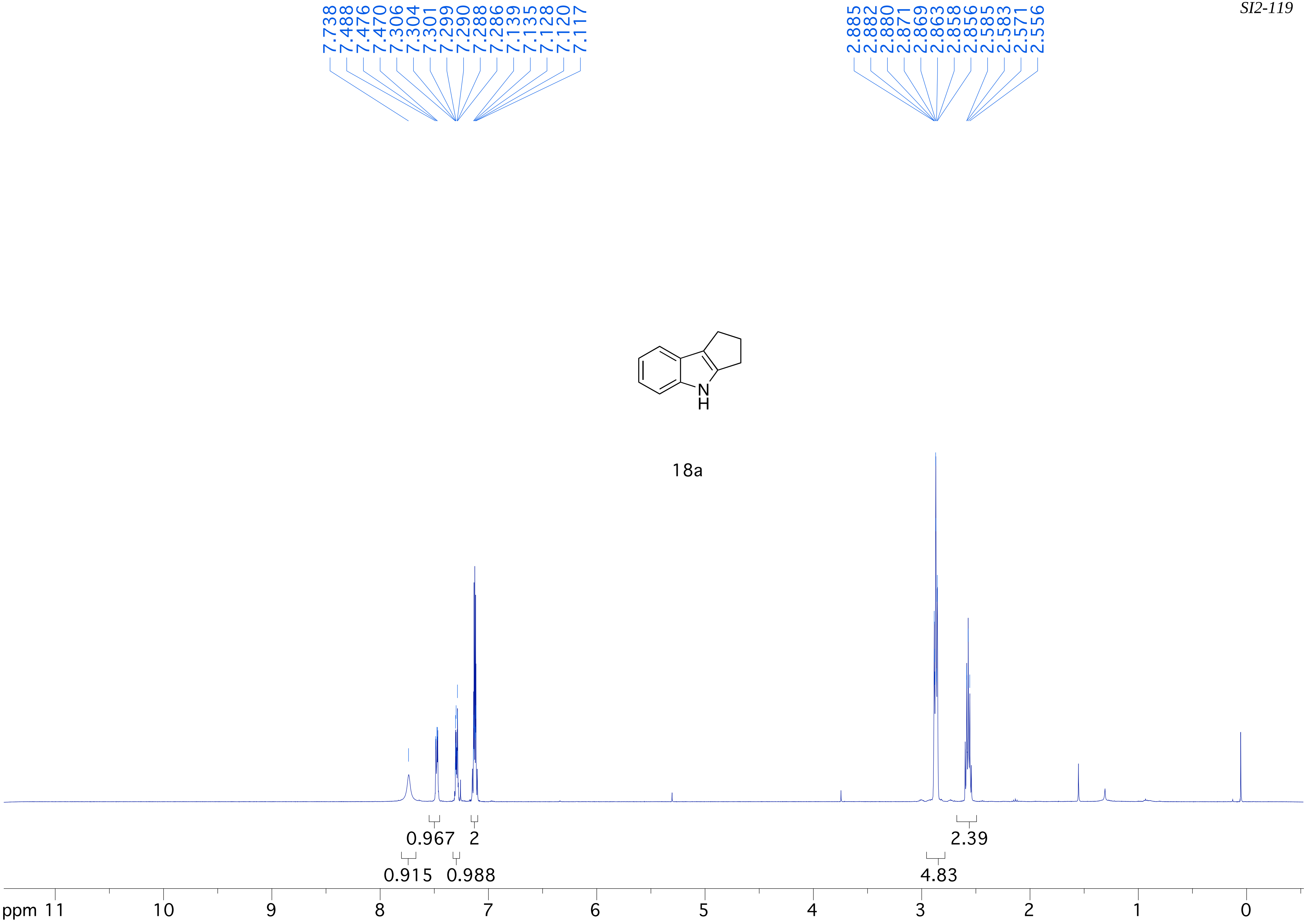
7.738
7.488
7.476
7.470
7.306
7.304
7.301
7.299
7.290
7.288
7.286
7.139
7.135
7.128
7.120
7.117
2.885
2.882
2.880
2.871
2.869
2.863
2.858
2.856
2.585
2.583
2.571
2.556
N
H
18a
0.967
2
2.39
0.915
0.988
4.83
ppm 11
10
9
8
7
6
5
4
3
2
1
0

SI2-120

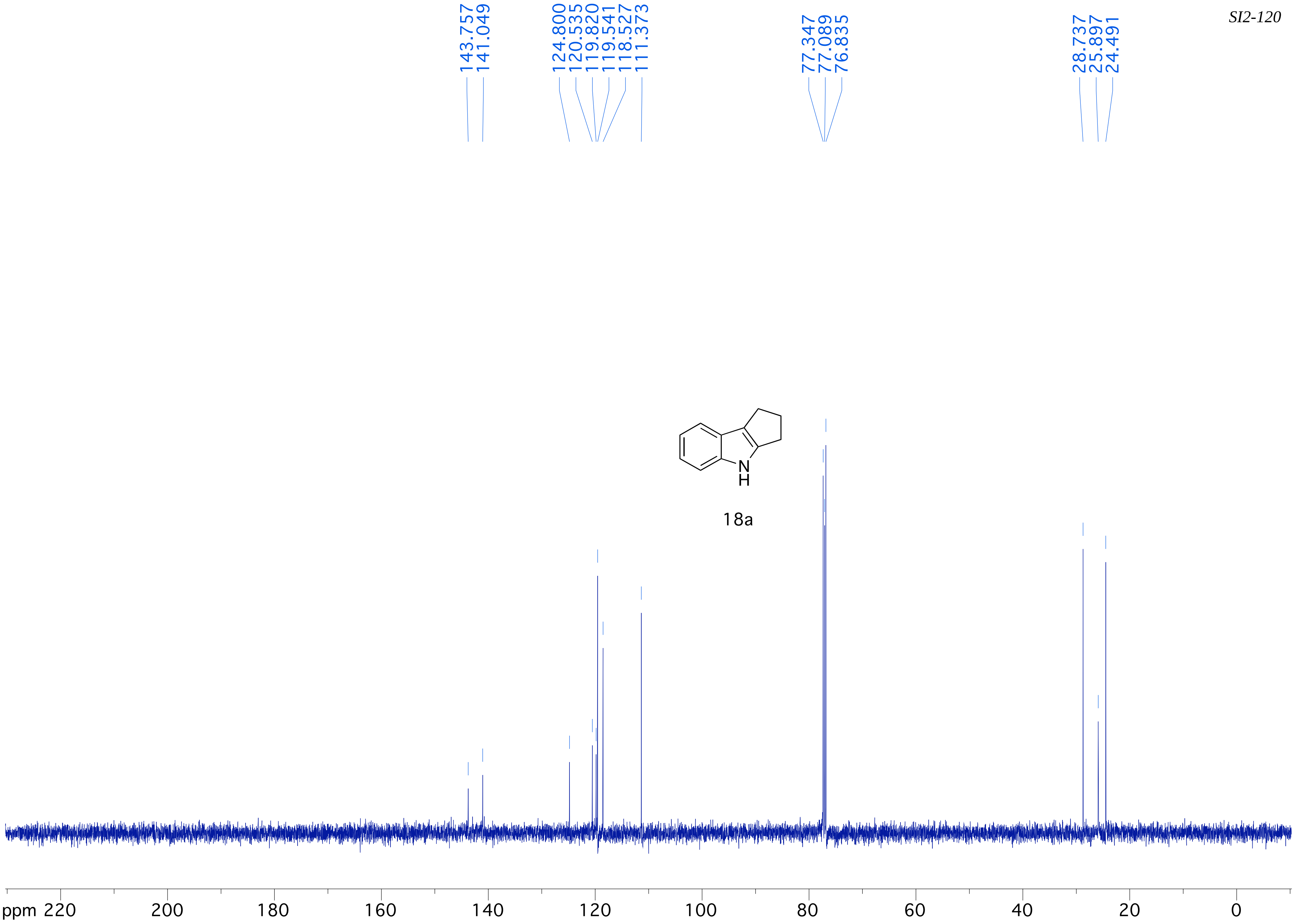
143.757
141.049
124.800
120.535
119.820
119.541
118.527
111.373
77.347
77.089
76.835
28.737
25.897
24.491
N
H
18a
ppm 220
200
180
160
140
120
100
80
60
40
20
0

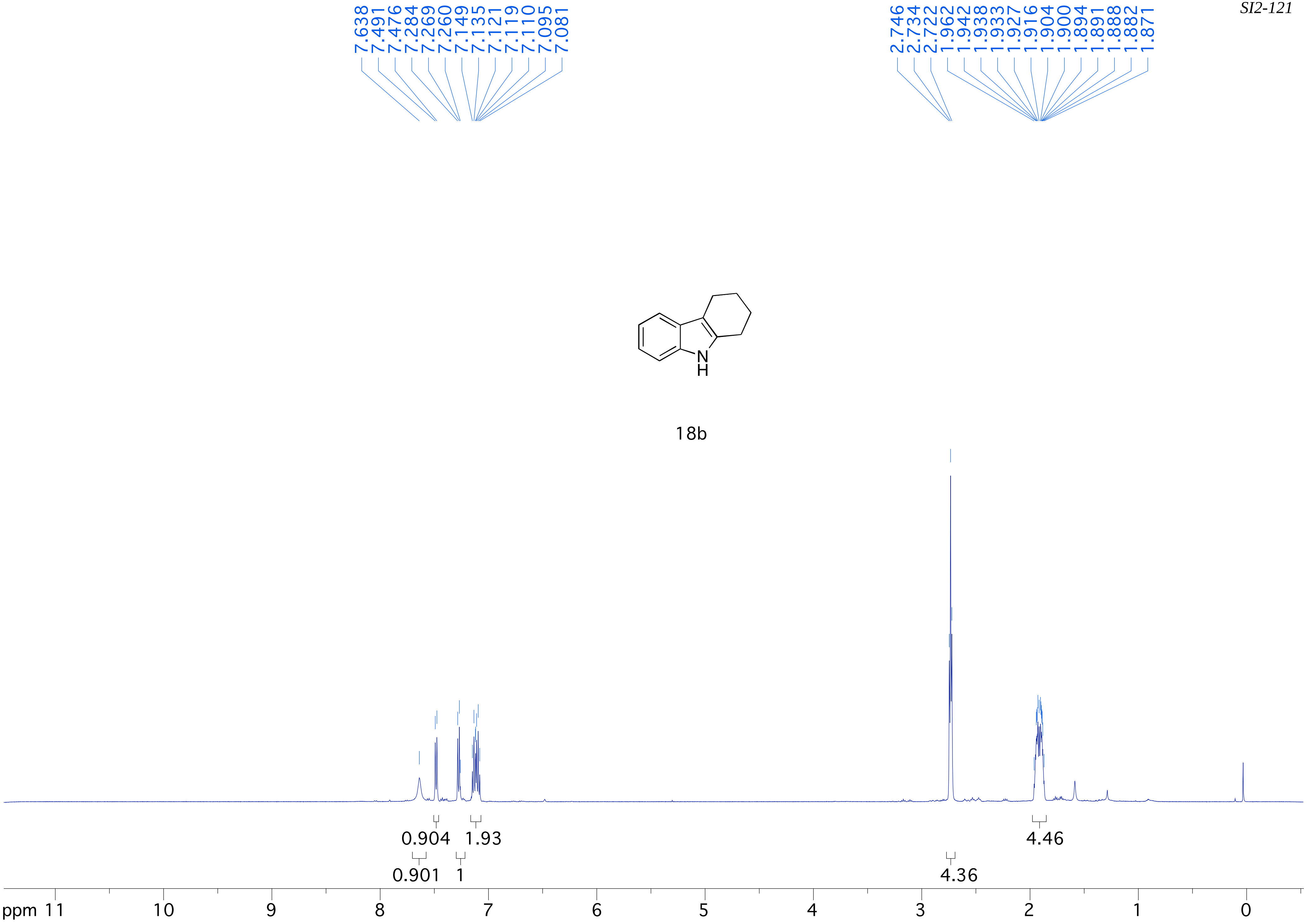
7.638
7.491
7.476
7.284
7.269
7.260
7.149
7.135
7.121
7.119
7.110
7.095
7.081
2.746
2.734
2.722
1.962
1.942
1.938
1.933
1.927
1.916
1.904
1.900
1.894
1.891
1.888
1.882
1.871
N
H
18b
0.904
1.93
4.46
0.901
1
4.36
ppm 11
10
9
8
7
6
5
4
3
2
1
0

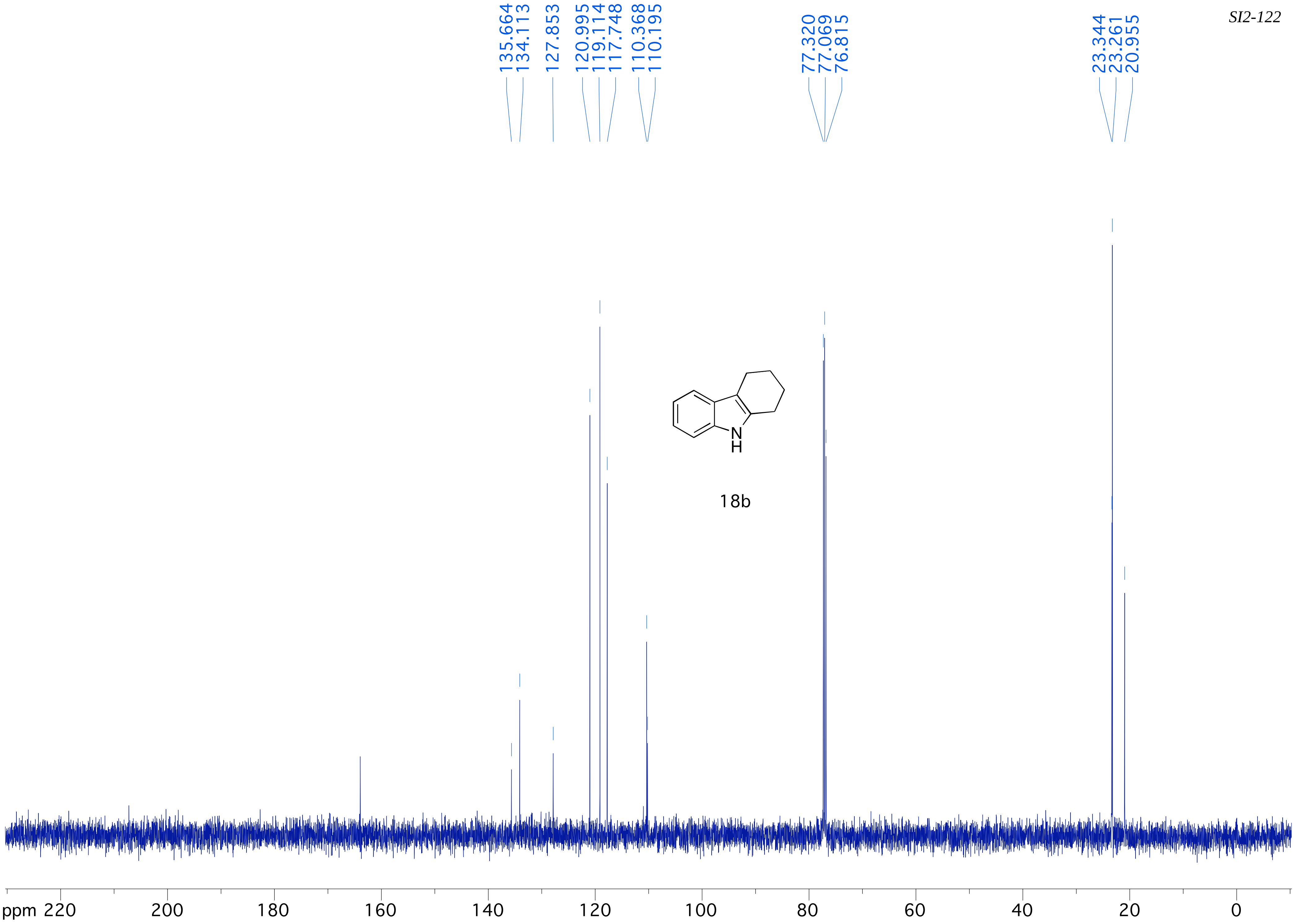

135.664
134.113
127.853
120.995
119.114
117.748
110.368
110.195
77.320
77.069
76.815
23.344
23.261
20.955
N
H
ppm 220
200
180
160
140
120
100
80
60
40
20
0

18b

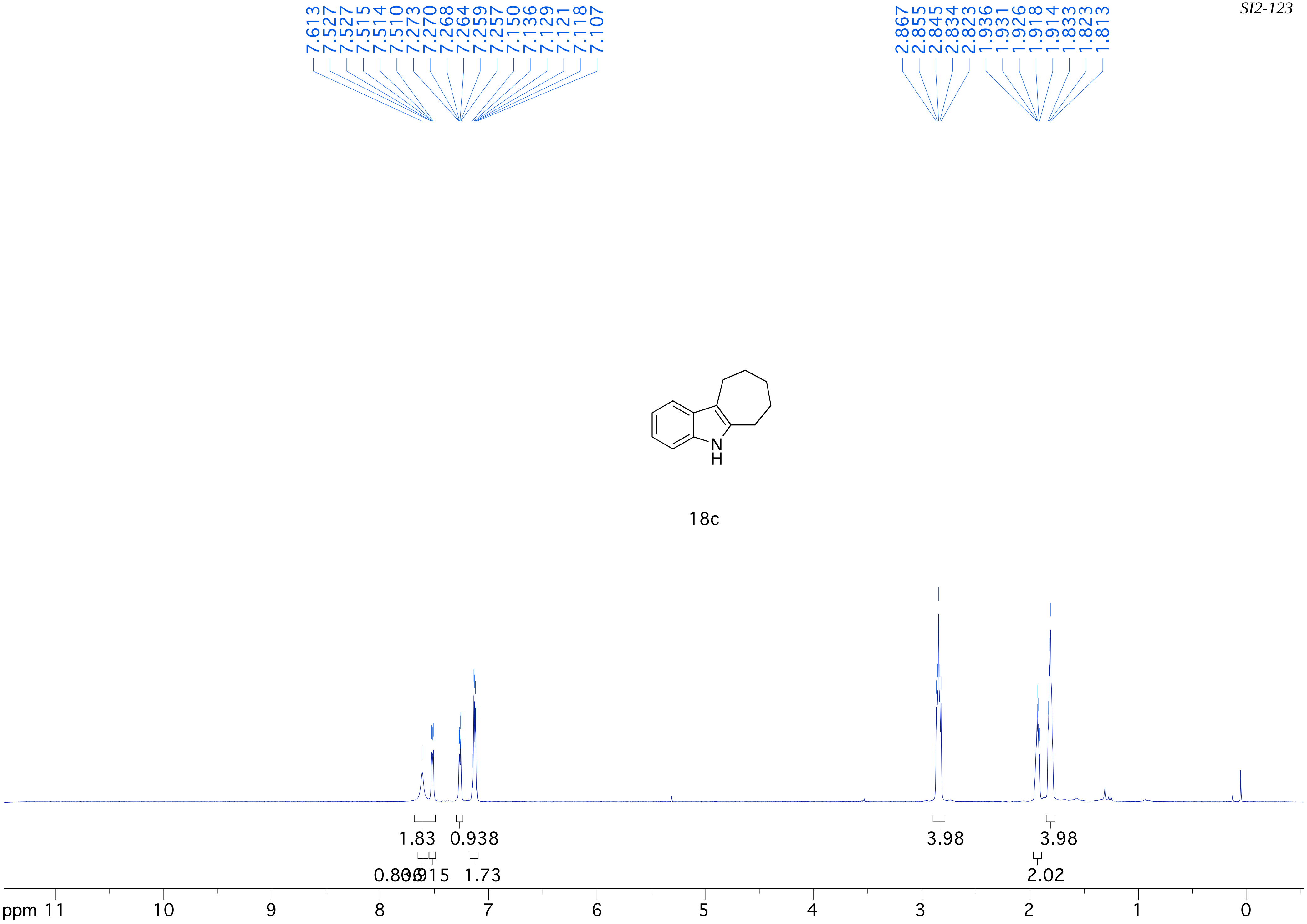

7.613
7.527
7.527
7.515
7.514
7.510
7.273
7.270
7.268
7.264
7.259
7.257
7.150
7.136
7.129
7.121
7.118
7.107
2.867
2.855
2.845
2.834
2.823
1.936
1.931
1.926
1.918
1.914
1.833
1.823
1.813
N
H
18c
1.83
0.938
3.98
3.98
0.806
0.915
1.73
2.02
ppm 11
10
9
8
7
6
5
4
3
2
1
0

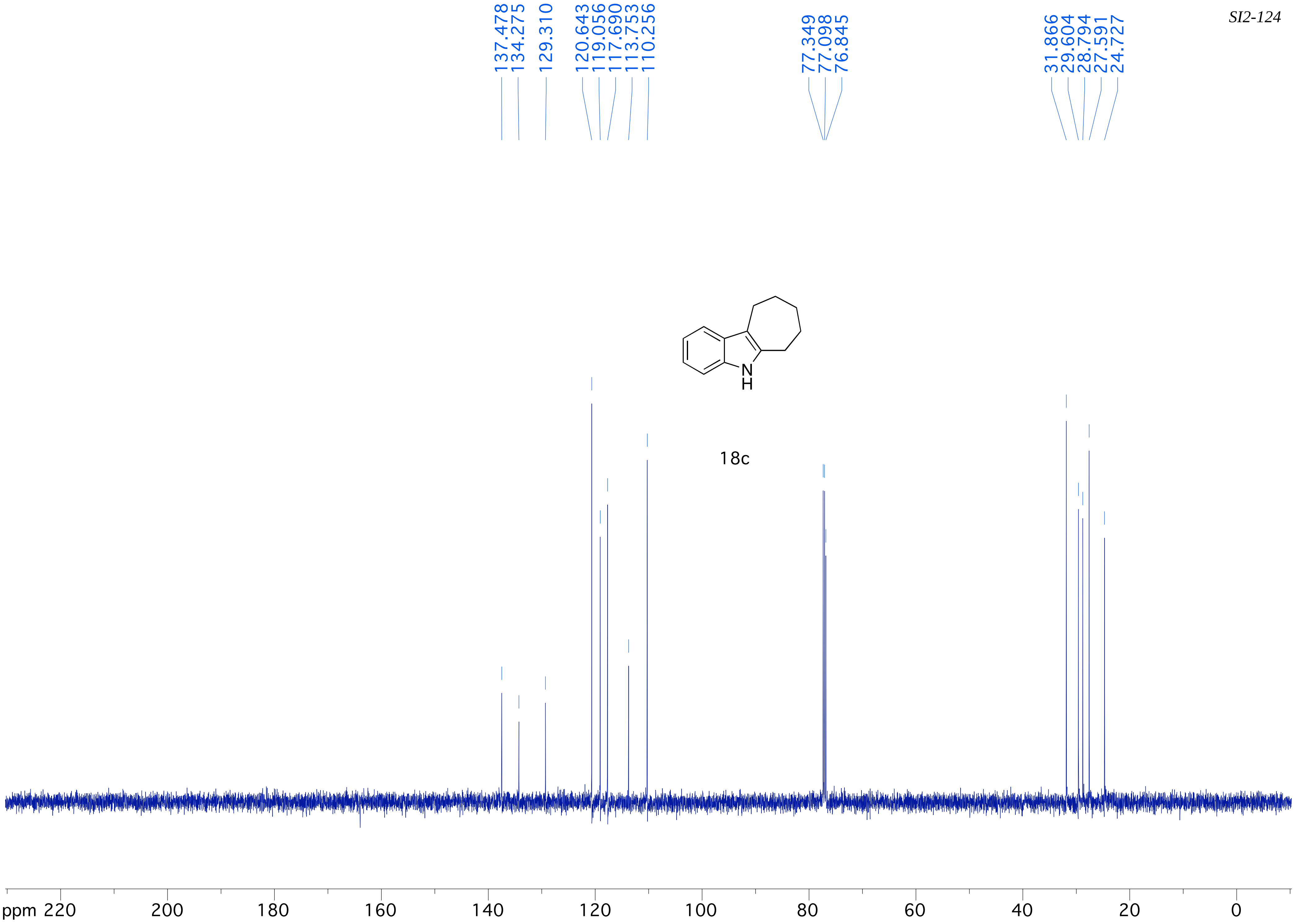
137.478
134.275
129.310
120.643
119.056
117.690
113.753
110.256
77.349
77.098
76.845
31.866
29.604
28.794
27.591
24.727
ppm 220
200
180
160
140
120
100
80
60
40
20
0

18c

SI2-125

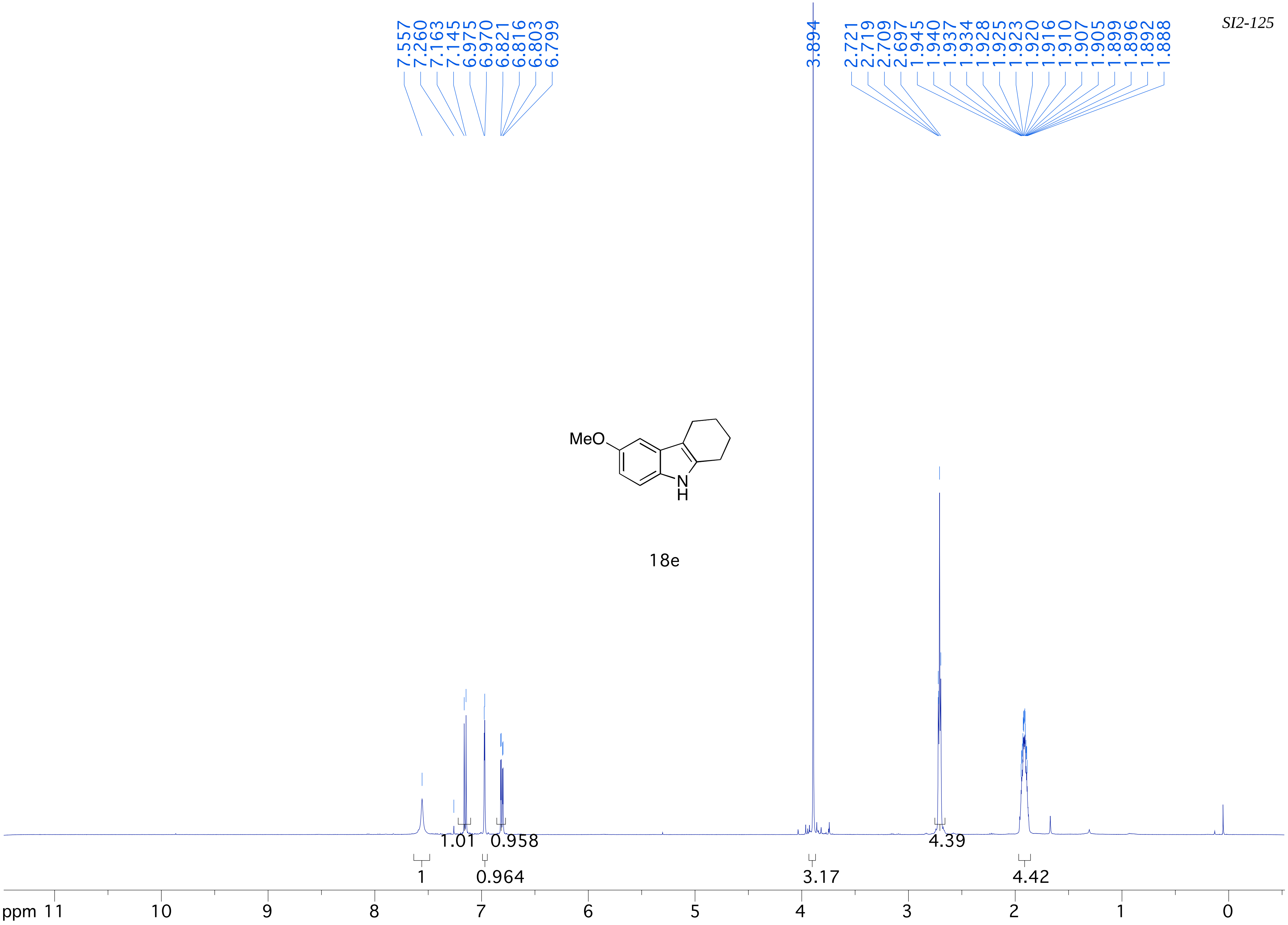

7.557
7.260
7.163
7.145
6.975
6.970
6.821
6.816
6.803
6.799
3.894
2.721
2.719
2.709
2.697
1.945
1.940
1.937
1.934
1.928
1.925
1.923
1.920
1.916
1.910
1.907
1.905
1.899
1.896
1.892
1.888
MeO
N
H
18e
1.01
0.958
4.39
1
0.964
3.17
4.42
ppm 11
10
9
8
7
6
5
4
3
2
1
0

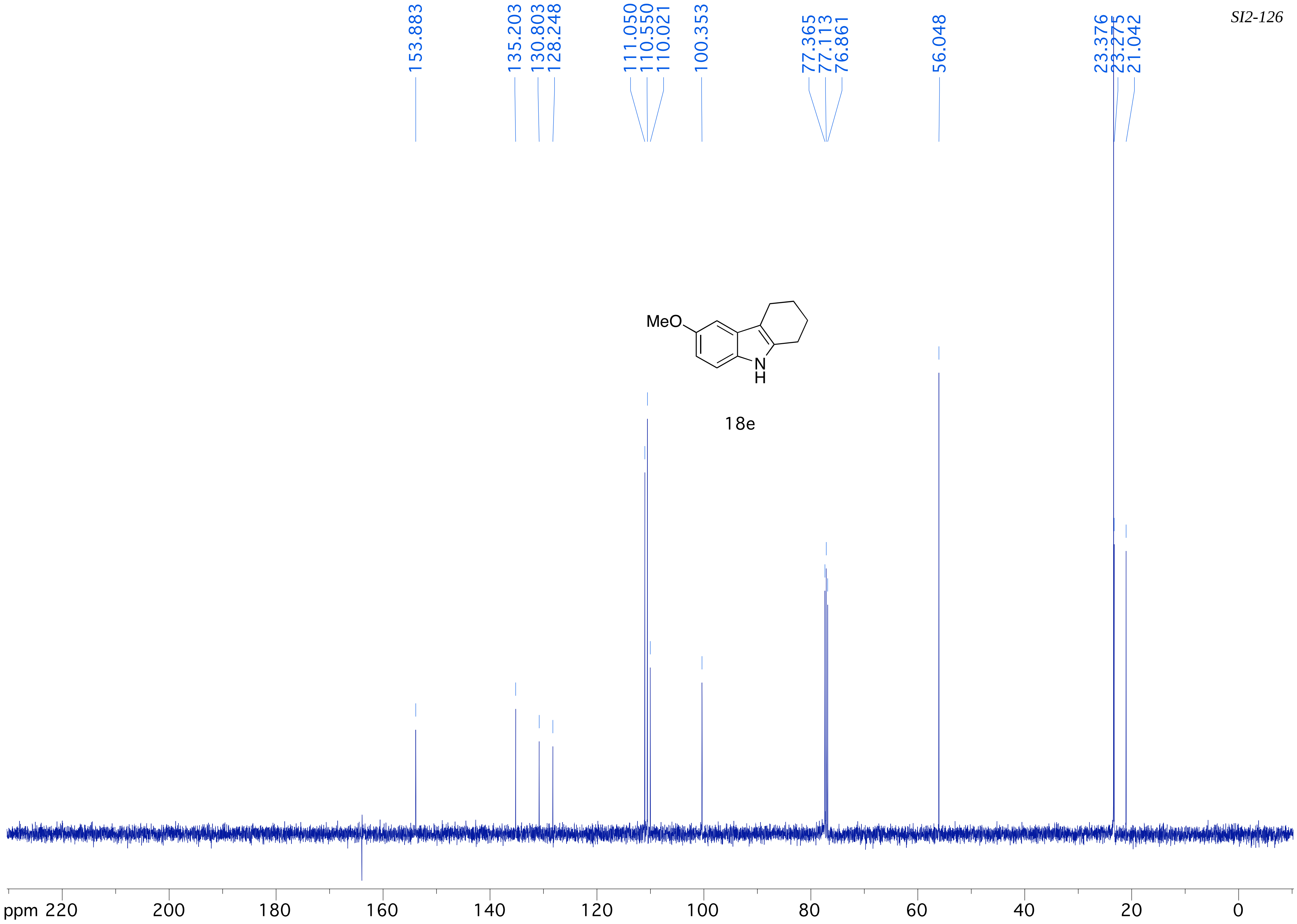
153.883
135.203
130.803
128.248
111.050
110.550
110.021
100.353
77.365
77.113
76.861
56.048
23.376
23.275
21.042
MeO
N
H
18e
ppm 220
200
180
160
140
120
100
80
60
40
20
0

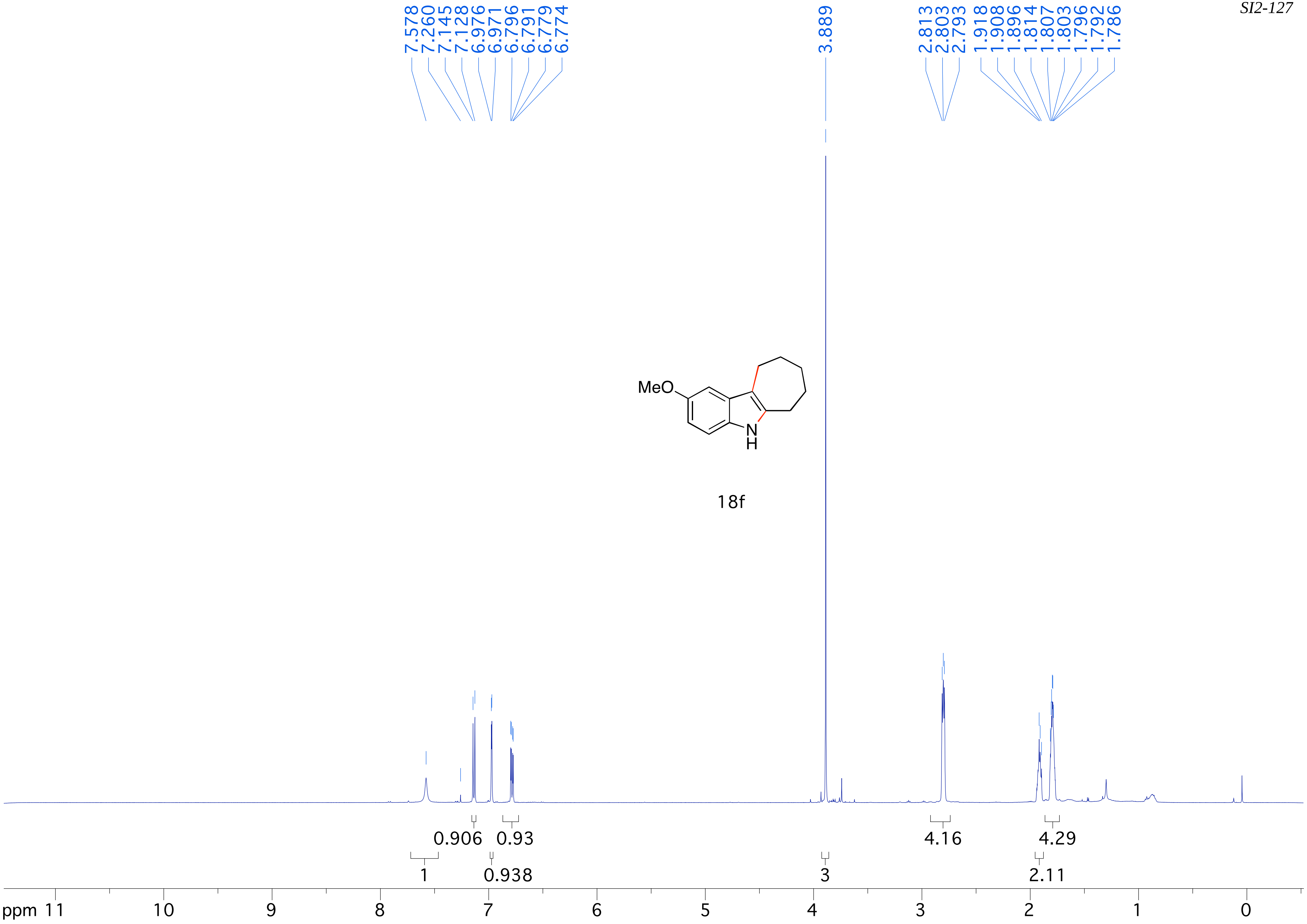
7.578
7.260
7.145
7.128
6.976
6.971
6.796
6.791
6.779
6.774
3.889
2.813
2.803
2.793
1.918
1.908
1.896
1.814
1.807
1.803
1.796
1.792
1.786
MeO
N
H
18f
0.906
0.93
4.16
4.29
1
0.938
3
2.11
ppm 11
10
9
8
7
6
5
4
3
2
1
0

SI2-128

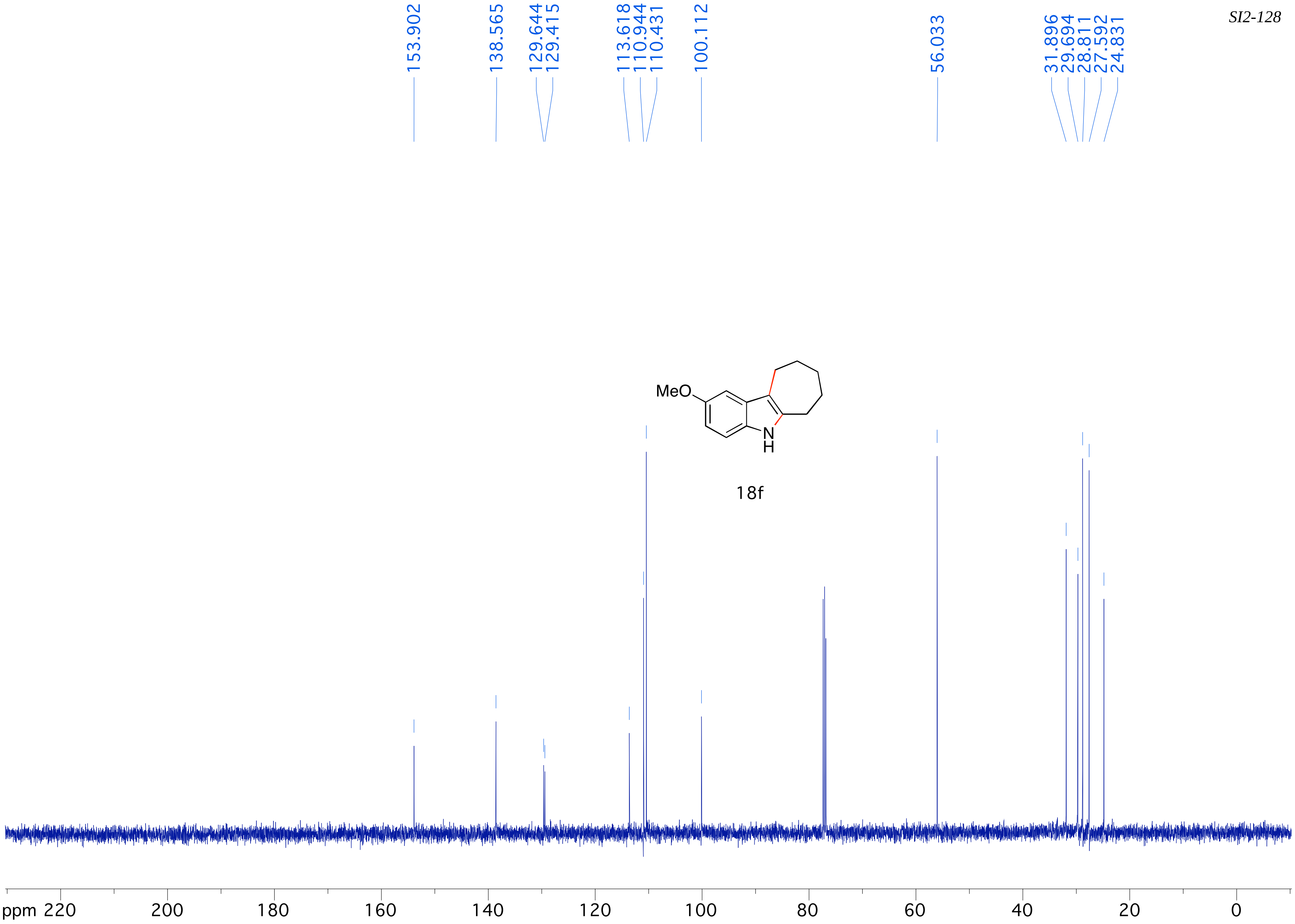

153.902
138.565
129.644
129.415
113.618
110.944
110.431
100.112
56.033
31.896
29.694
28.811
27.592
24.831
MeO
N
H
18f
ppm 220
200
180
160
140
120
100
80
60
40
20
0

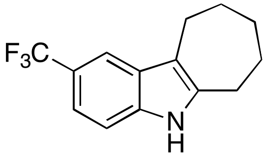

18g

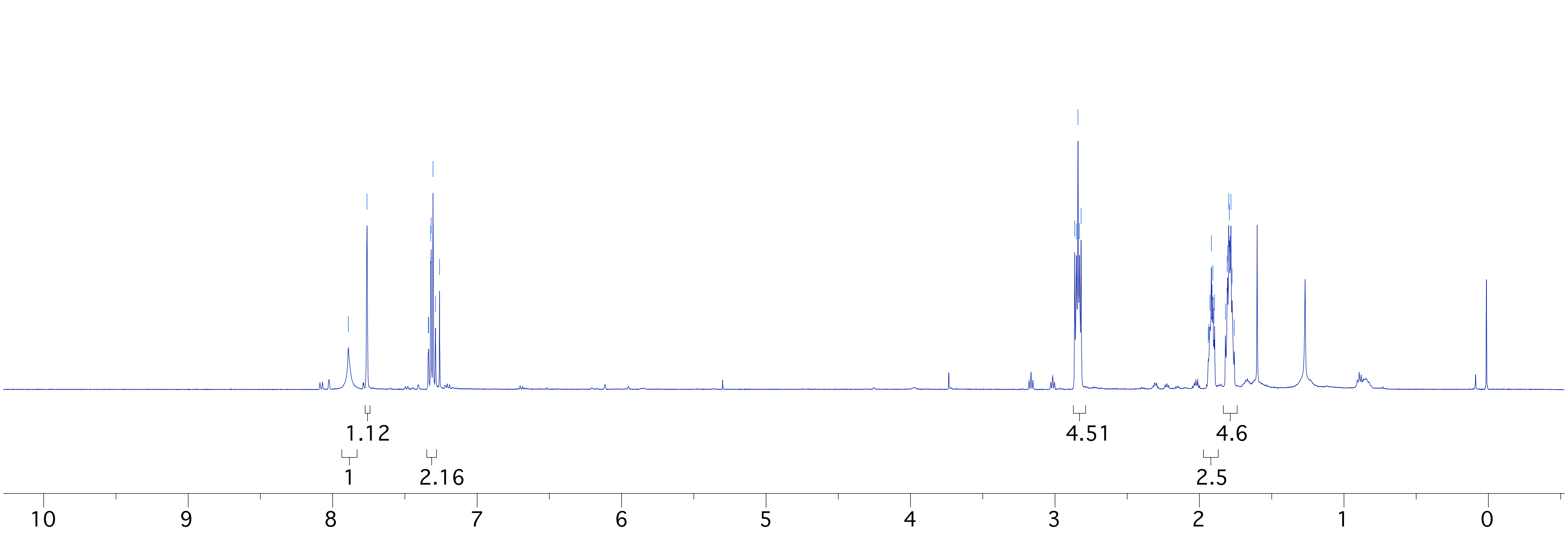
7.891
7.763
7.338
7.335
7.321
7.318
7.305
7.288
7.260
2.863
2.852
2.841
2.831
2.819
1.939
1.935
1.928
1.918
1.913
1.907
1.900
1.895
1.820
1.808
1.805
1.797
1.794
1.788
1.782
1.774
1.759
1.12
4.51
4.6
1
2.16
2.5
10
9
8
7
6
5
4
3
2
1
0

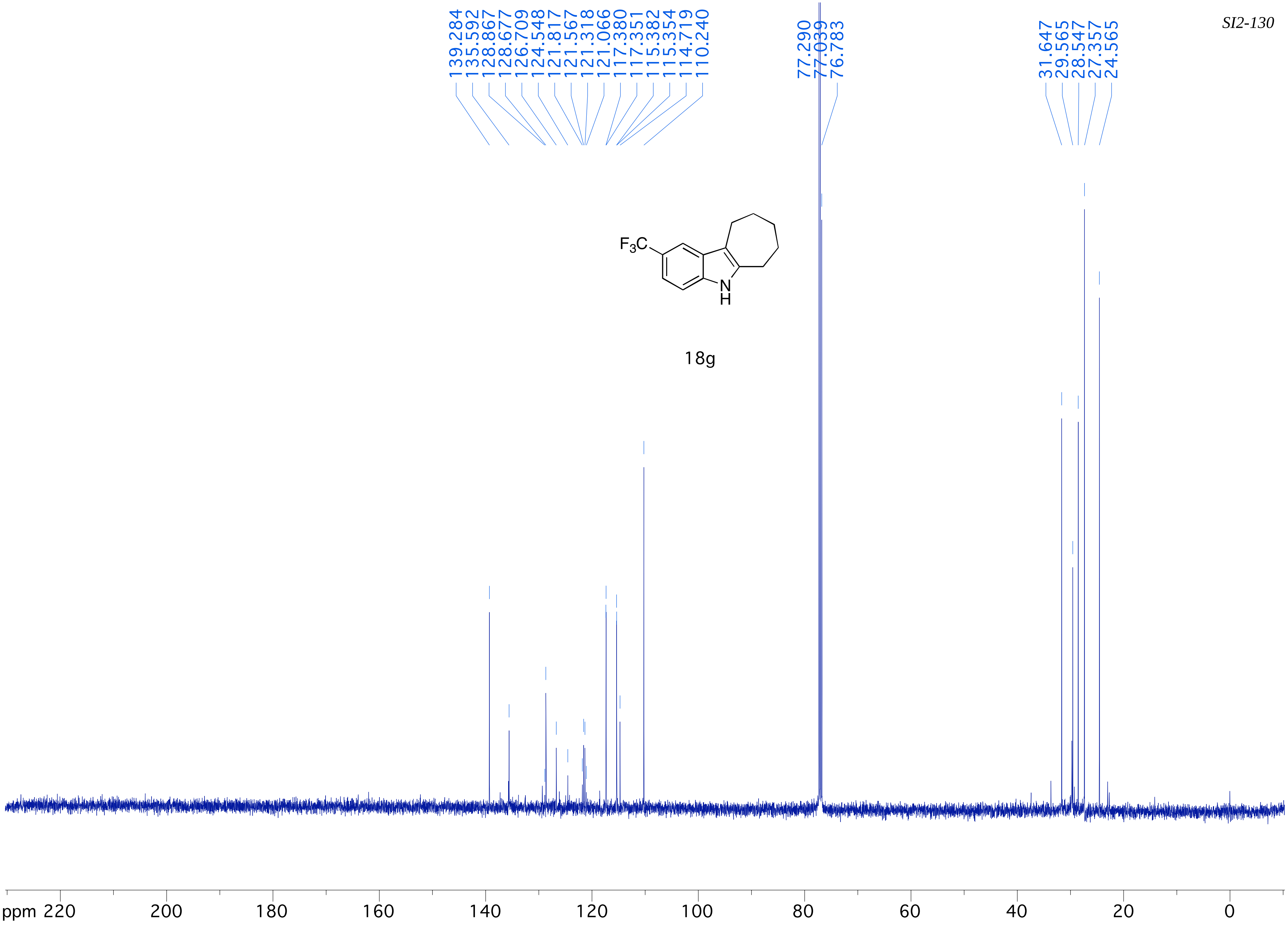
SI2-130
139.284
135.592
128.867
128.677
126.709
124.548
121.817
121.567
121.318
121.066
117.380
117.351
115.382
115.354
114.719
110.240
77.290
77.039
76.783
31.647
29.565
28.547
27.357
24.565
F3C
N
H
18g
ppm 220
200
180
160
140
120
100
80
60
40
20
0

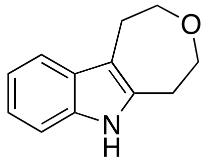

18h

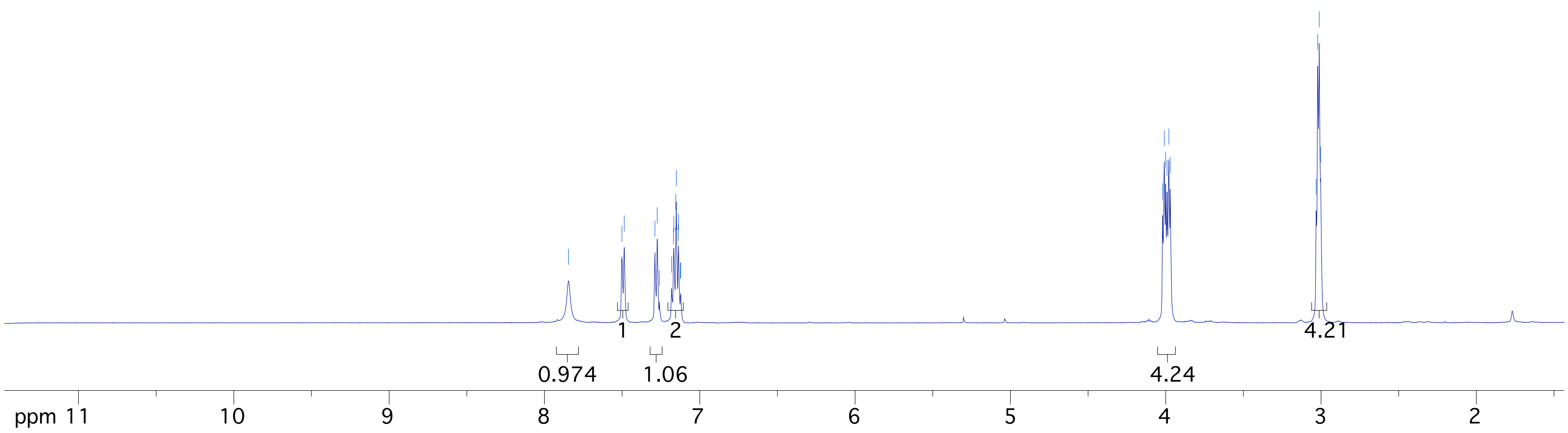
7.845
7.501
7.486
7.289
7.274
7.260
7.181
7.170
7.167
7.154
7.151
7.139
7.137
7.125
7.122
4.021
4.009
4.000
3.991
3.980
3.971
3.031
3.022
3.012
3.003
1
2
4.21
0.974
1.06
4.24
ppm 11
10
9
8
7
6
5
4
3
2

SI2-132

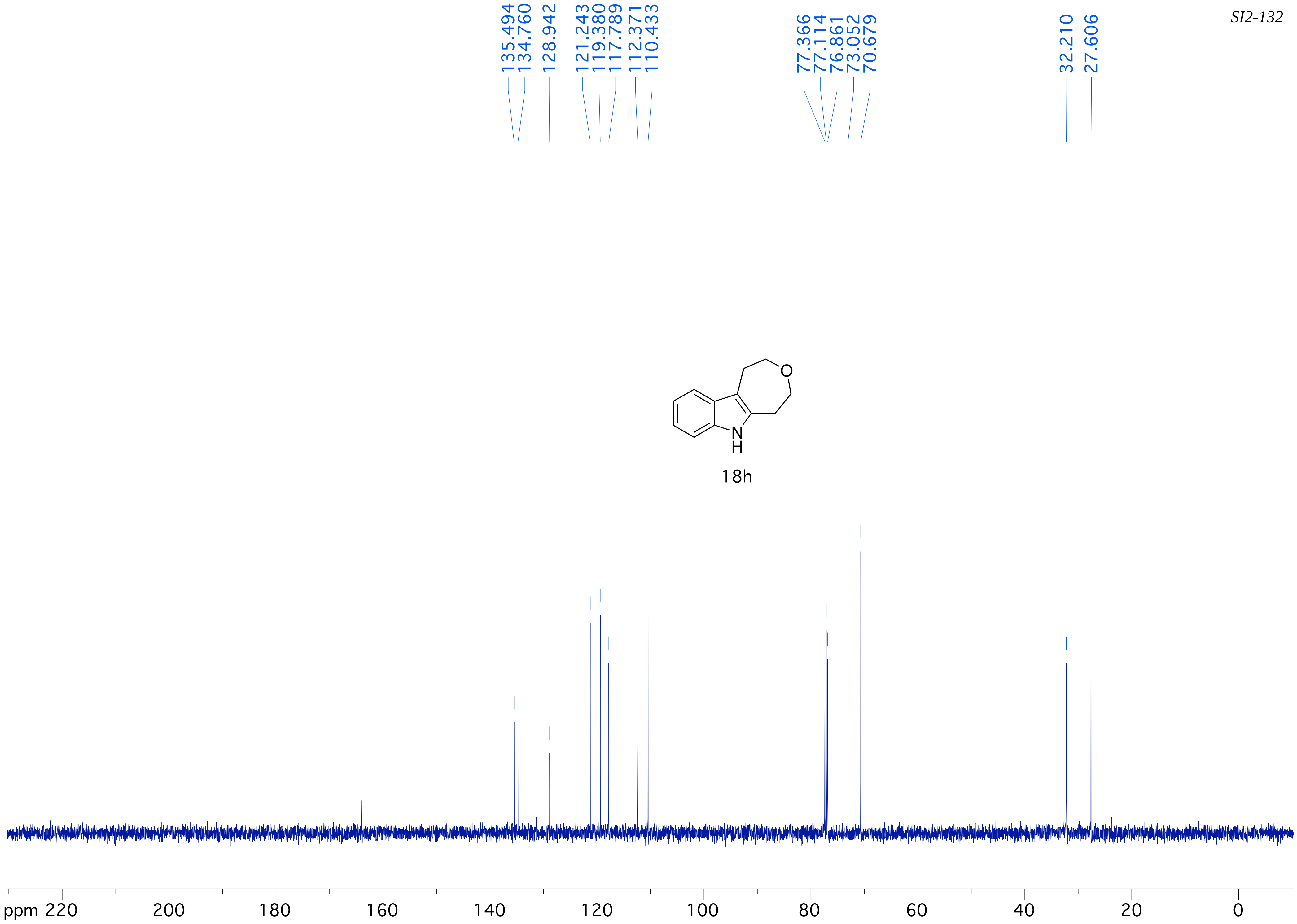
135.494
134.760
128.942
121.243
119.380
117.789
112.371
110.433
77.366
77.114
76.861
73.052
70.679
32.210
27.606
O
N
H
18h
ppm 220
200
180
160
140
120
100
80
60
40
20
0

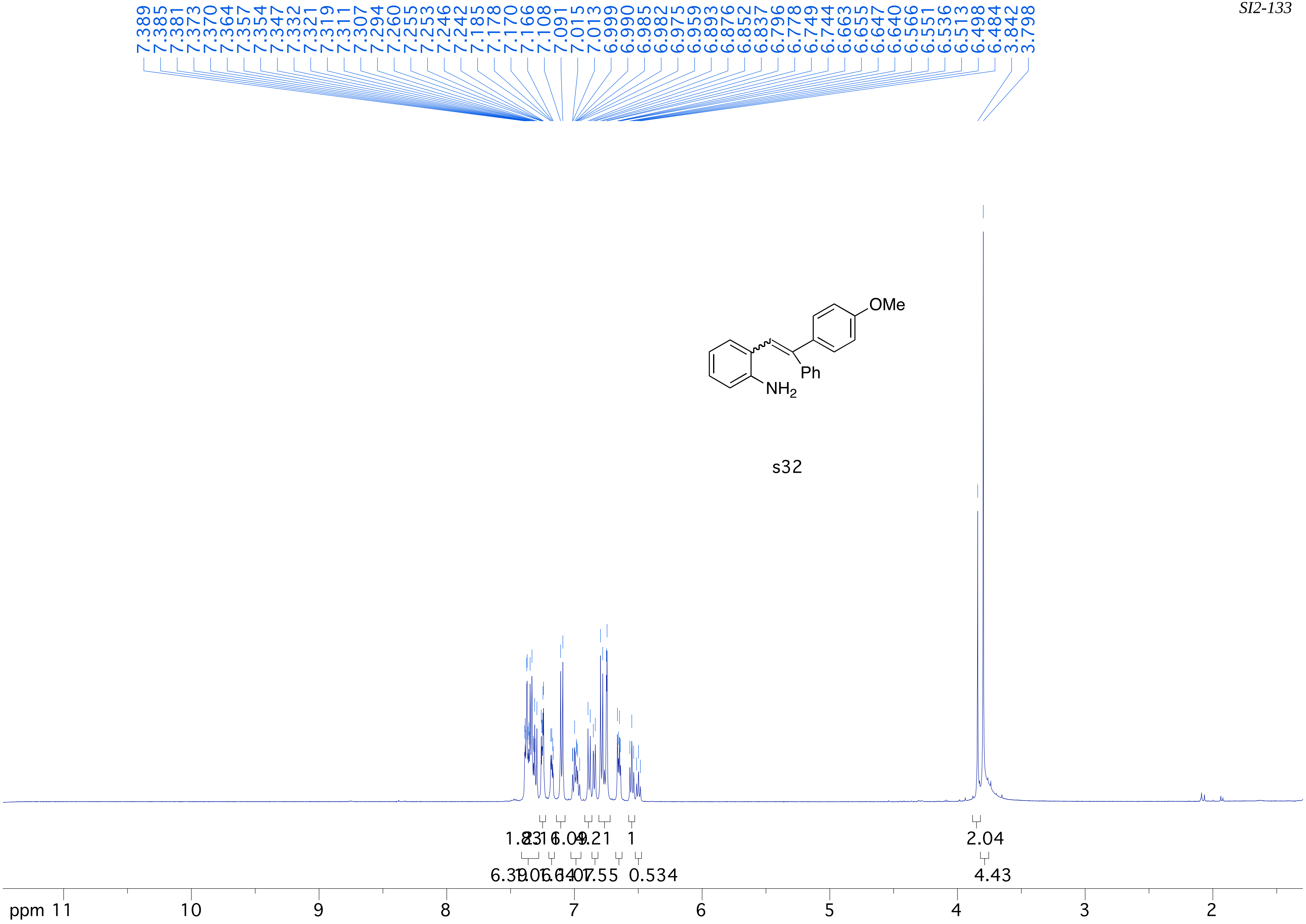
7.389
7.385
7.381
7.373
7.370
7.364
7.357
7.354
7.347
7.332
7.321
7.319
7.311
7.307
7.294
7.260
7.255
7.253
7.246
7.242
7.185
7.178
7.170
7.166
7.108
7.091
7.015
7.013
6.999
6.990
6.985
6.982
6.975
6.959
6.893
6.876
6.852
6.837
6.796
6.778
6.749
6.744
6.663
6.655
6.647
6.640
6.566
6.551
6.536
6.513
6.498
6.484
3.842
3.798
OMe
Ph
NH2
2.04
4.43
1
0.534
ppm 11
10
9
8
7
6
5
4
3
2

s32

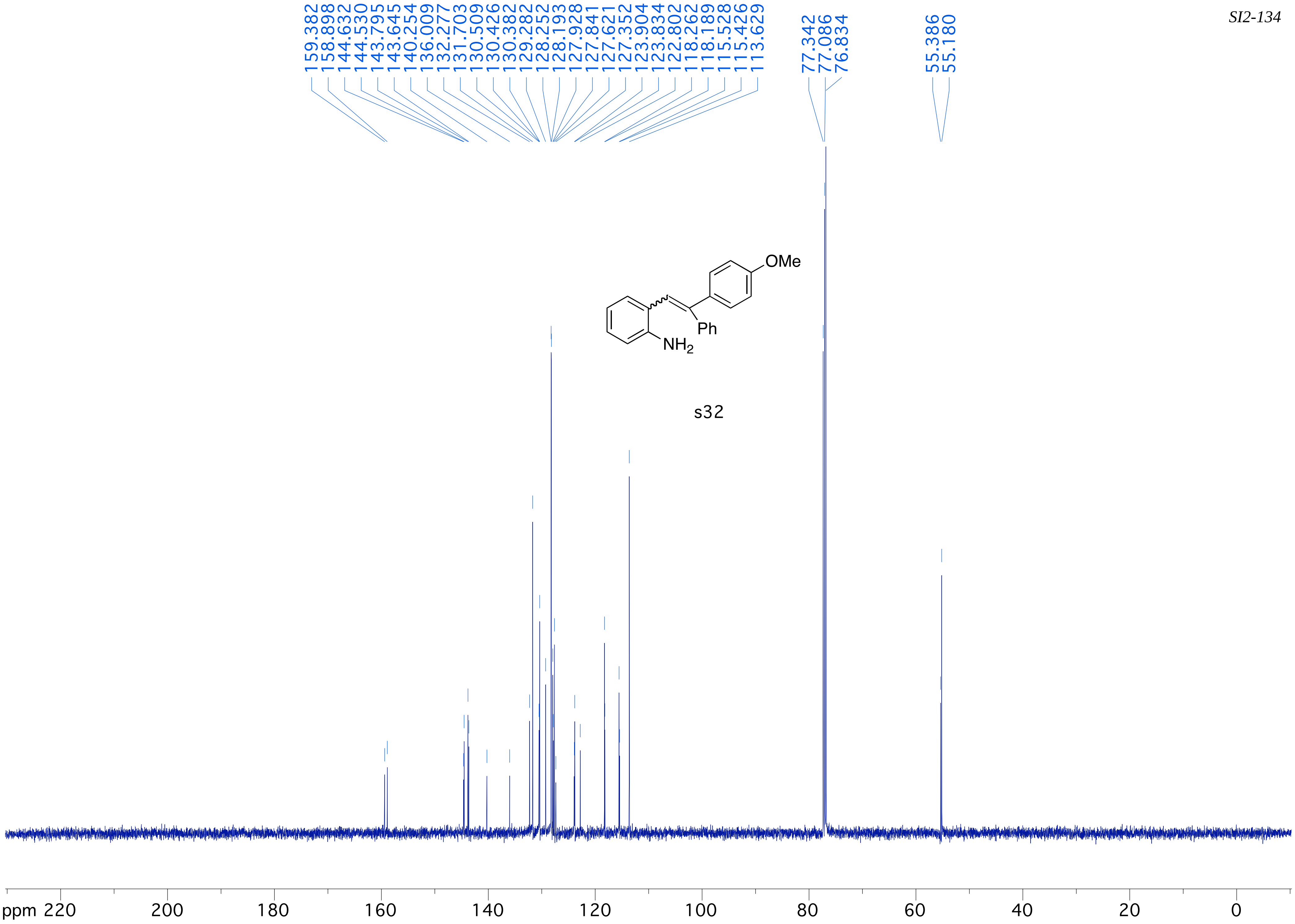
159.382
158.898
144.632
144.530
143.795
143.645
140.254
136.009
132.277
131.703
130.509
130.426
130.382
129.282
128.252
128.193
127.928
127.841
127.621
127.352
123.904
123.834
122.802
118.262
118.189
115.528
115.426
113.629
77.342
77.086
76.834
55.386
55.180
OMe
Ph
NH2
s32
ppm 220
200
180
160
140
120
100
80
60
40
20
0

SI2-135

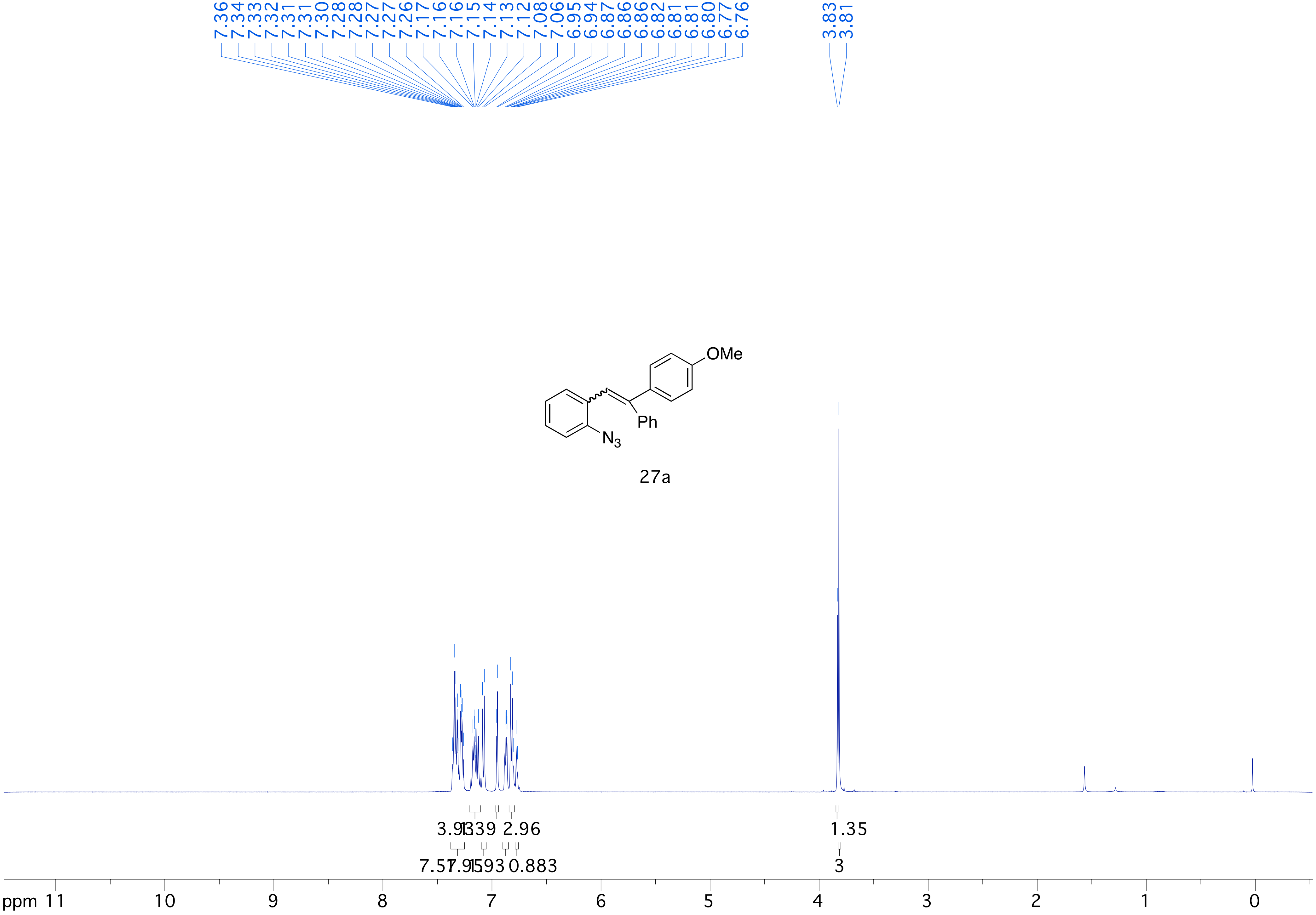
7.361
7.345
7.332
7.320
7.316
7.312
7.303
7.289
7.281
7.276
7.272
7.260
7.174
7.164
7.161
7.153
7.148
7.137
7.124
7.087
7.069
6.957
6.949
6.879
6.867
6.862
6.829
6.816
6.811
6.804
6.778
6.766
3.833
3.819
OMe
Ph
N3
27a
3.91
1.39
2.96
1.35
7.57
7.91
5.93
0.88
3
3
ppm 11
10
9
8
7
6
5
4
3
2
1
0

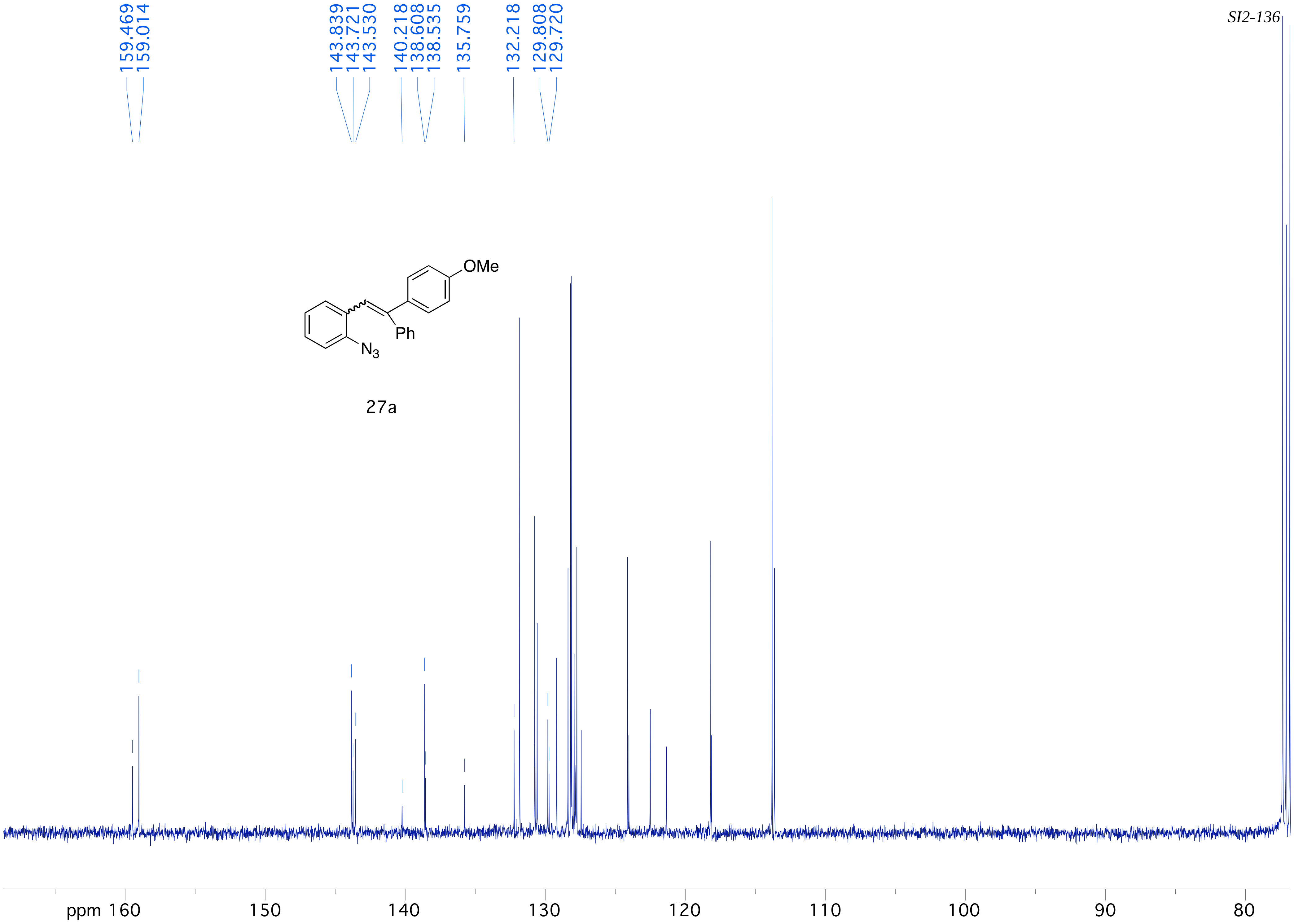

159.469
159.014
143.839
143.721
143.530
140.218
138.608
138.535
135.759
132.218
129.808
129.720
SI2-136
OMe
Ph
N_3
27a
ppm 160
150
140
130
120
110
100
90
80

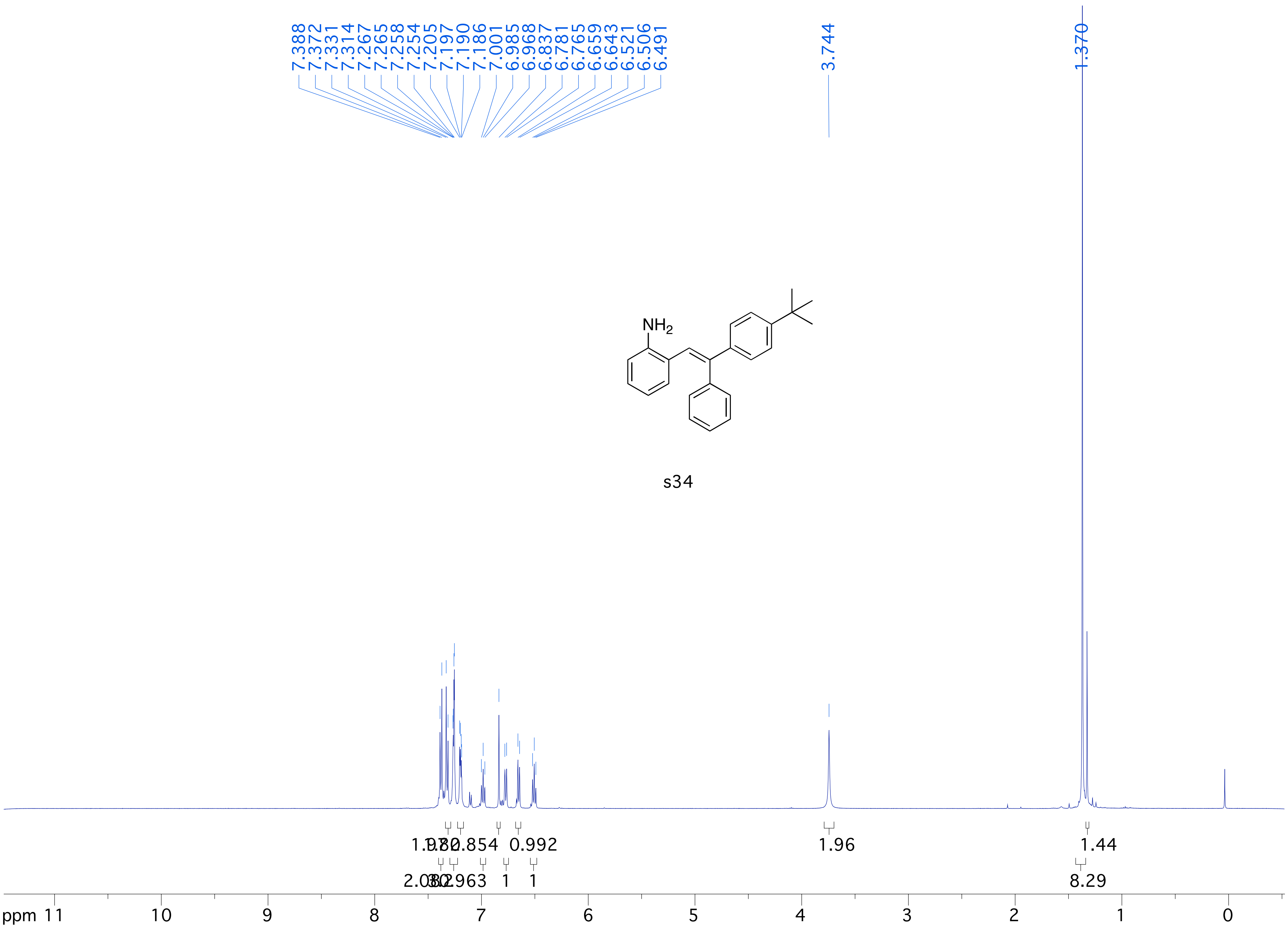
7.388
7.372
7.331
7.314
7.267
7.265
7.258
7.254
7.205
7.197
7.190
7.186
7.001
6.985
6.968
6.837
6.781
6.765
6.659
6.643
6.521
6.506
6.491
3.744
1.370
NH2
s34
1.96
1.44
8.29
0.992
1
1
ppm 11 10 9 8 7 6 5 4 3 2 1 0

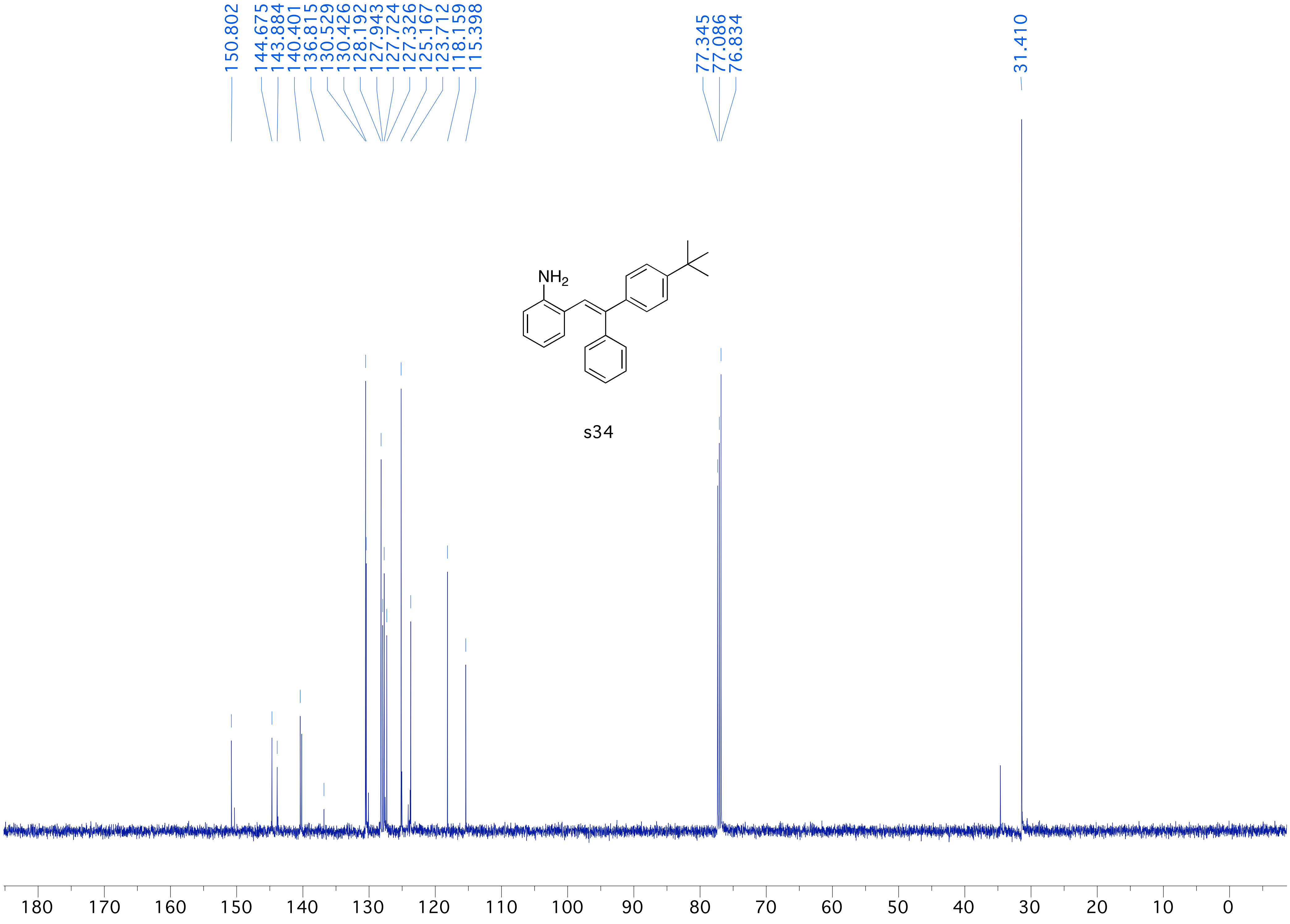
150.802
144.675
143.884
140.401
136.815
130.529
130.426
128.192
127.943
127.724
127.326
125.167
123.712
118.159
115.398
77.345
77.086
76.834
31.410
NH_2
s34
180 170 160 150 140 130 120 110 100 90 80 70 60 50 40 30 20 10 0

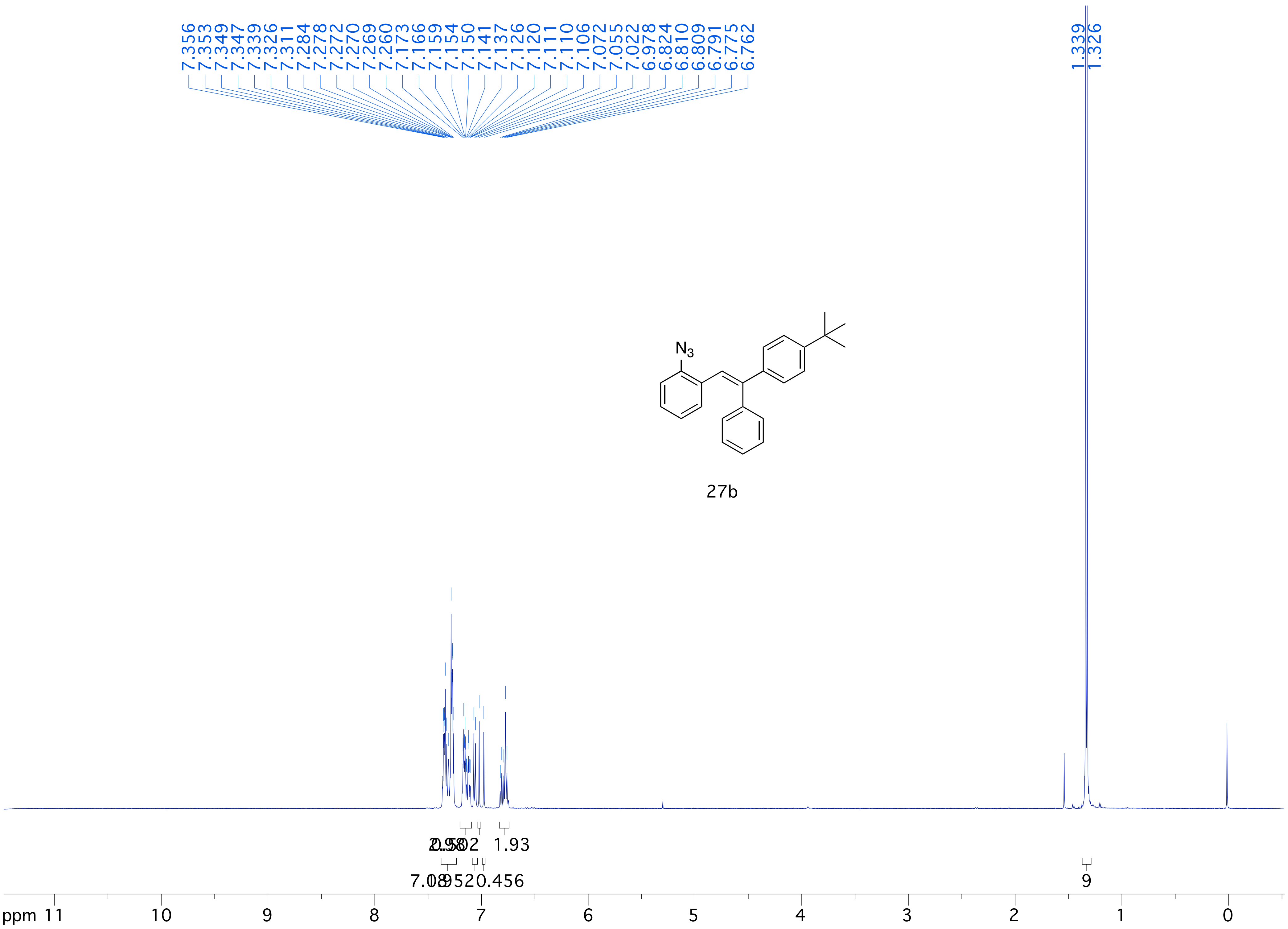

7.356
7.353
7.349
7.347
7.339
7.326
7.311
7.284
7.278
7.272
7.270
7.269
7.260
7.173
7.166
7.159
7.154
7.150
7.141
7.137
7.126
7.120
7.111
7.110
7.106
7.072
7.055
7.022
6.978
6.824
6.810
6.809
6.791
6.775
6.762
1.339
1.326
N3
27b
1.93
9
ppm 11
10
9
8
7
6
5
4
3
2
1
0

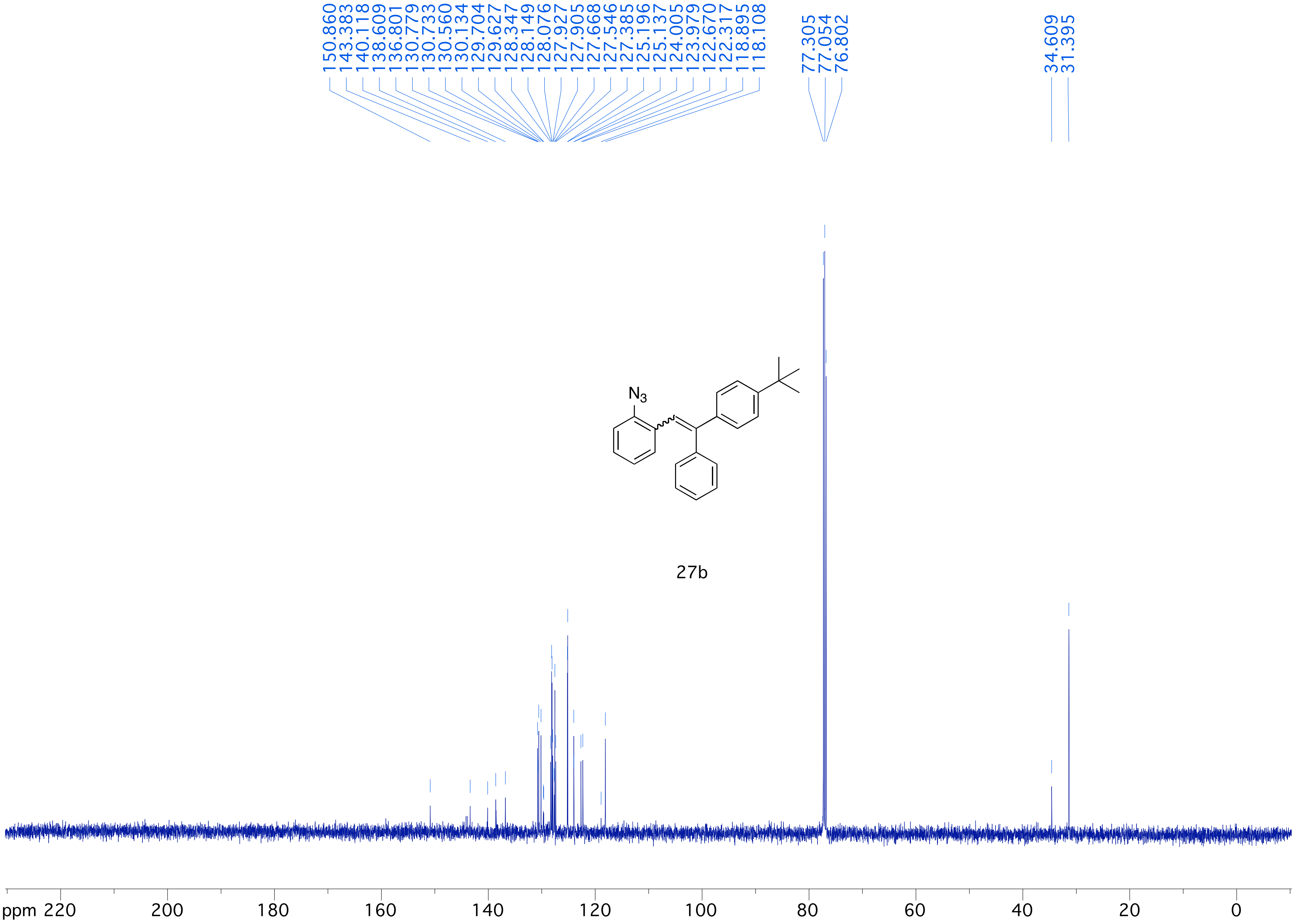
150.860
143.383
140.118
138.609
136.801
130.779
130.733
130.560
130.134
129.704
129.627
128.347
128.149
128.076
127.927
127.905
127.668
127.546
127.385
125.196
125.137
124.005
123.979
122.670
122.317
118.895
118.108
77.305
77.054
76.802
34.609
31.395
N3
ppm 220
200
180
160
140
120
100
80
60
40
20
0

27b

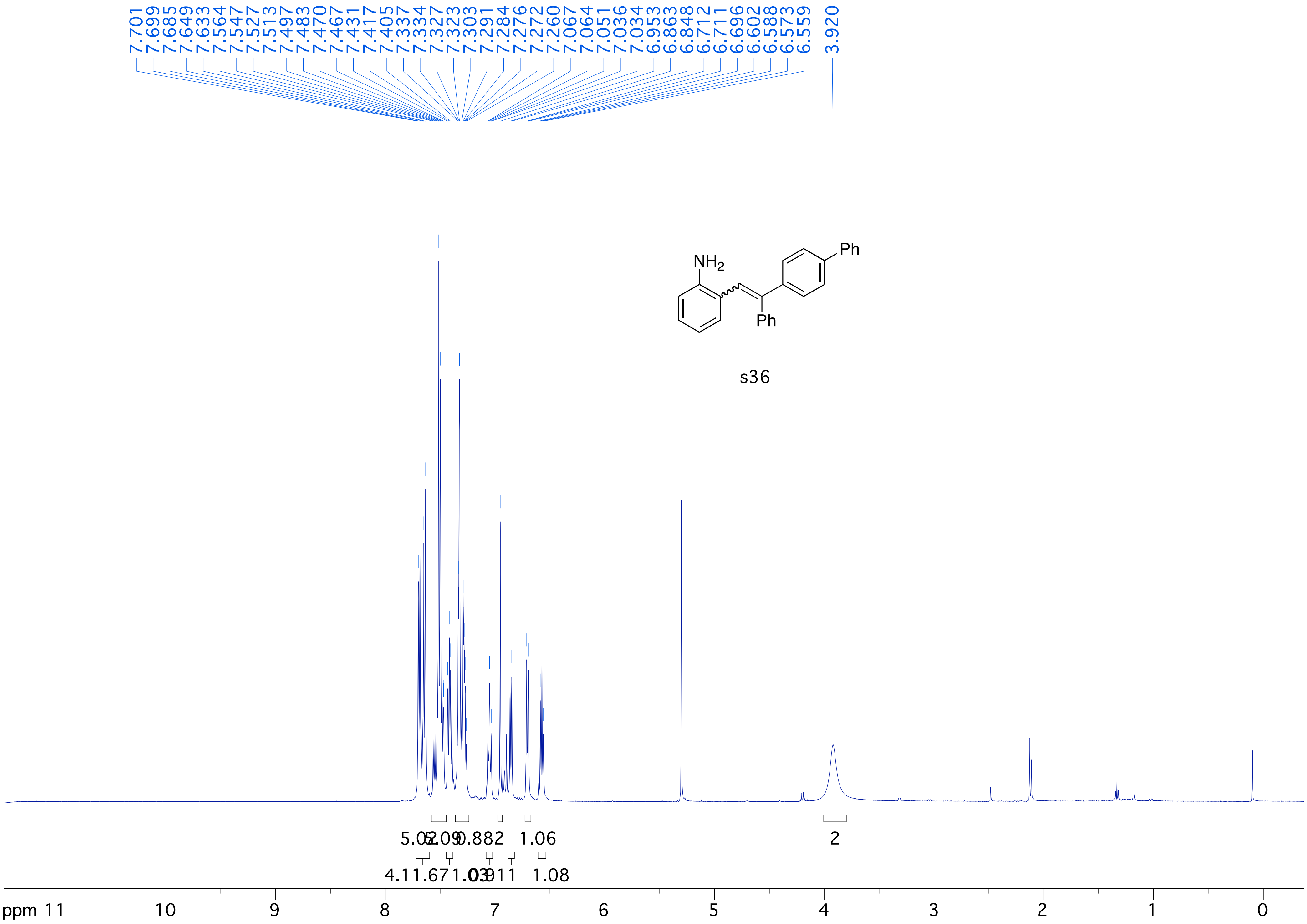

7.701
7.699
7.685
7.649
7.633
7.564
7.547
7.527
7.513
7.497
7.483
7.470
7.467
7.431
7.417
7.405
7.337
7.334
7.327
7.323
7.303
7.291
7.284
7.276
7.272
7.260
7.067
7.064
7.051
7.036
7.034
6.953
6.863
6.848
6.712
6.711
6.696
6.692
6.602
6.588
6.573
6.559
3.920
NH2
Ph
Ph
s36
1.06
2
1.08
ppm 11
10
9
8
7
6
5
4
3
2
1
0

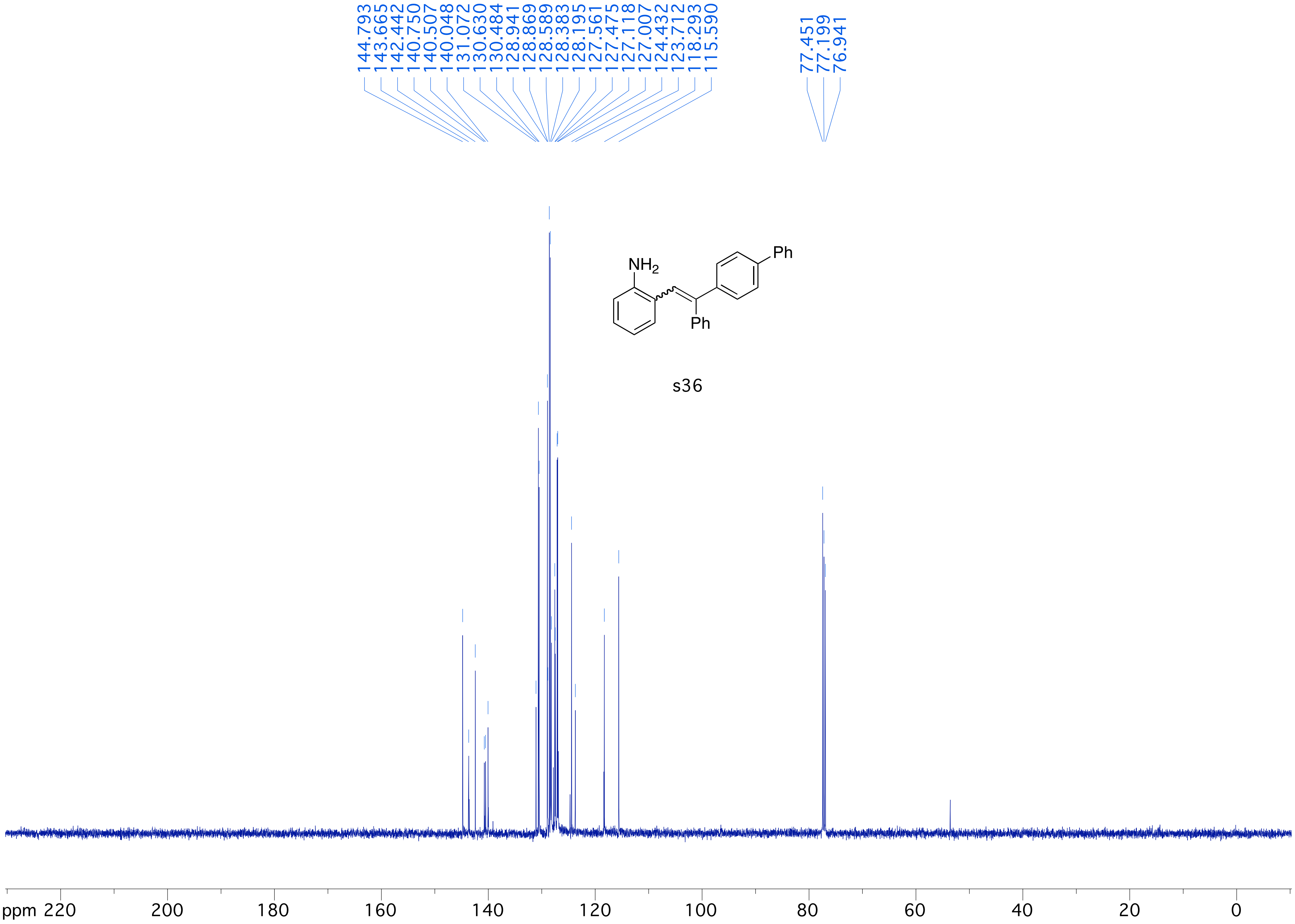
144.793
143.665
142.442
140.750
140.507
140.048
131.072
130.630
130.484
128.941
128.869
128.589
128.383
128.195
127.561
127.475
127.118
127.007
124.432
123.712
118.293
115.590
77.451
77.199
76.941
NH2
Ph
Ph
s36
ppm 220
200
180
160
140
120
100
80
60
40
20
0

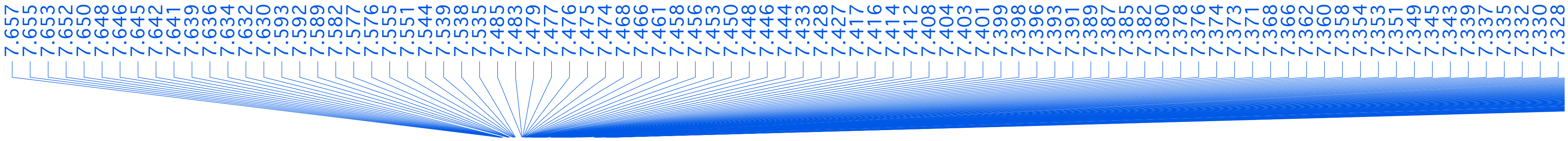
7.657
7.655
7.653
7.652
7.650
7.648
7.646
7.645
7.642
7.641
7.639
7.636
7.634
7.632
7.630
7.593
7.592
7.589
7.582
7.577
7.576
7.555
7.551
7.544
7.539
7.538
7.535
7.485
7.483
7.479
7.477
7.476
7.475
7.474
7.468
7.466
7.461
7.458
7.456
7.453
7.450
7.448
7.446
7.444
7.433
7.428
7.427
7.417
7.416
7.414
7.412
7.408
7.404
7.403
7.401
7.399
7.398
7.396
7.393
7.391
7.389
7.387
7.385
7.382
7.380
7.378
7.376
7.374
7.373
7.371
7.368
7.366
7.362
7.360
7.358
7.354
7.353
7.351
7.349
7.345
7.343
7.339
7.337
7.335
7.332
7.330
7.328

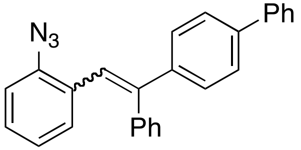
N3
Ph
Ph

27c

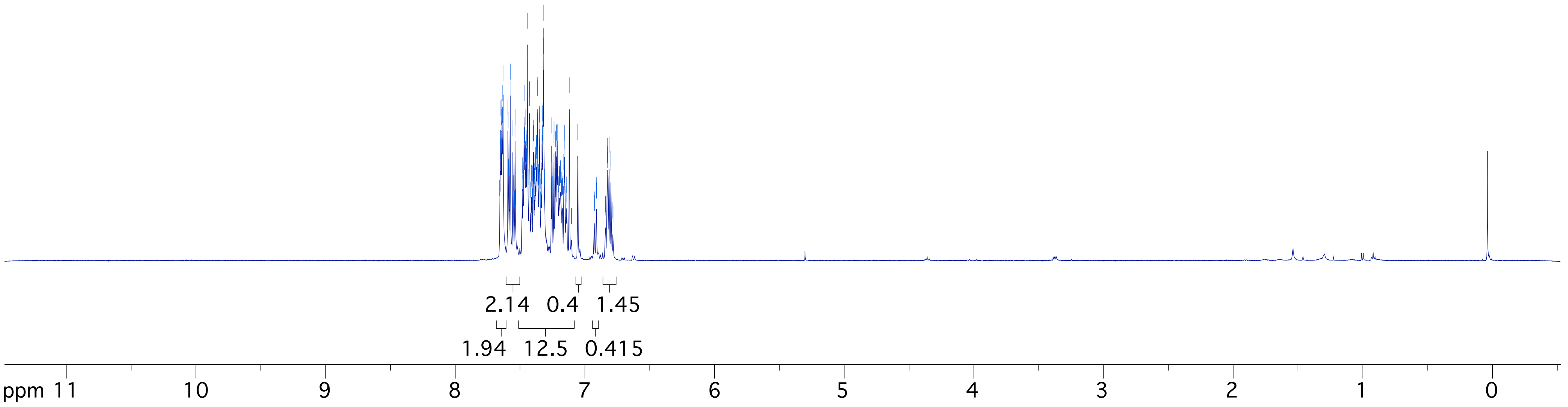
2.14
0.4
1.45
1.94
12.5
0.415
ppm 11
10
9
8
7
6
5
4
3
2
1
0

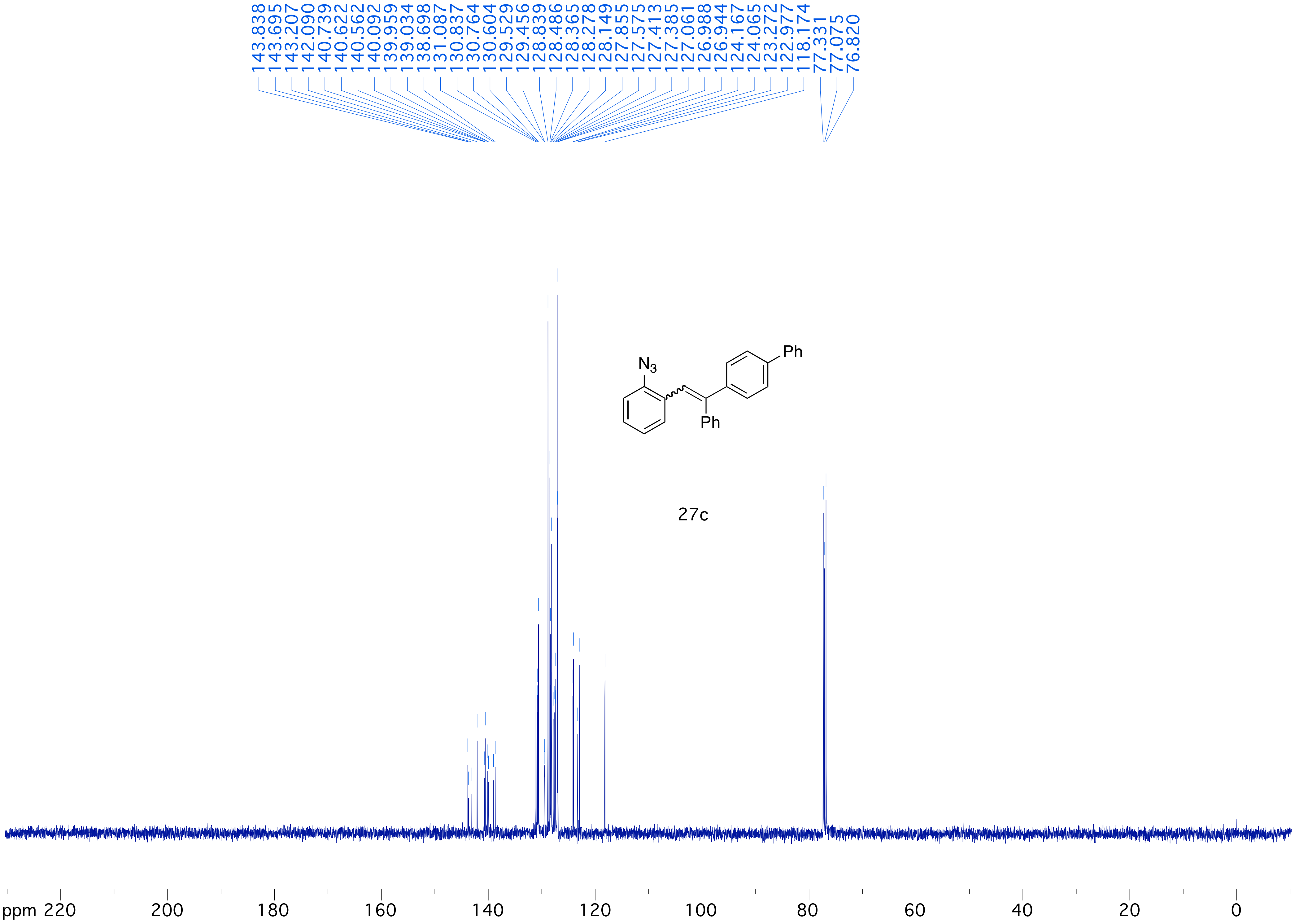
143.838
143.695
143.207
142.090
140.739
140.622
140.562
140.092
139.959
139.034
138.698
131.087
130.837
130.764
130.604
129.529
129.456
128.839
128.486
128.365
128.278
128.149
127.855
127.575
127.413
127.385
127.061
126.988
126.944
124.167
124.065
123.272
122.977
118.174
77.331
77.075
76.820
N3
Ph
Ph
27c
ppm 220
200
180
160
140
120
100
80
60
40
20
0

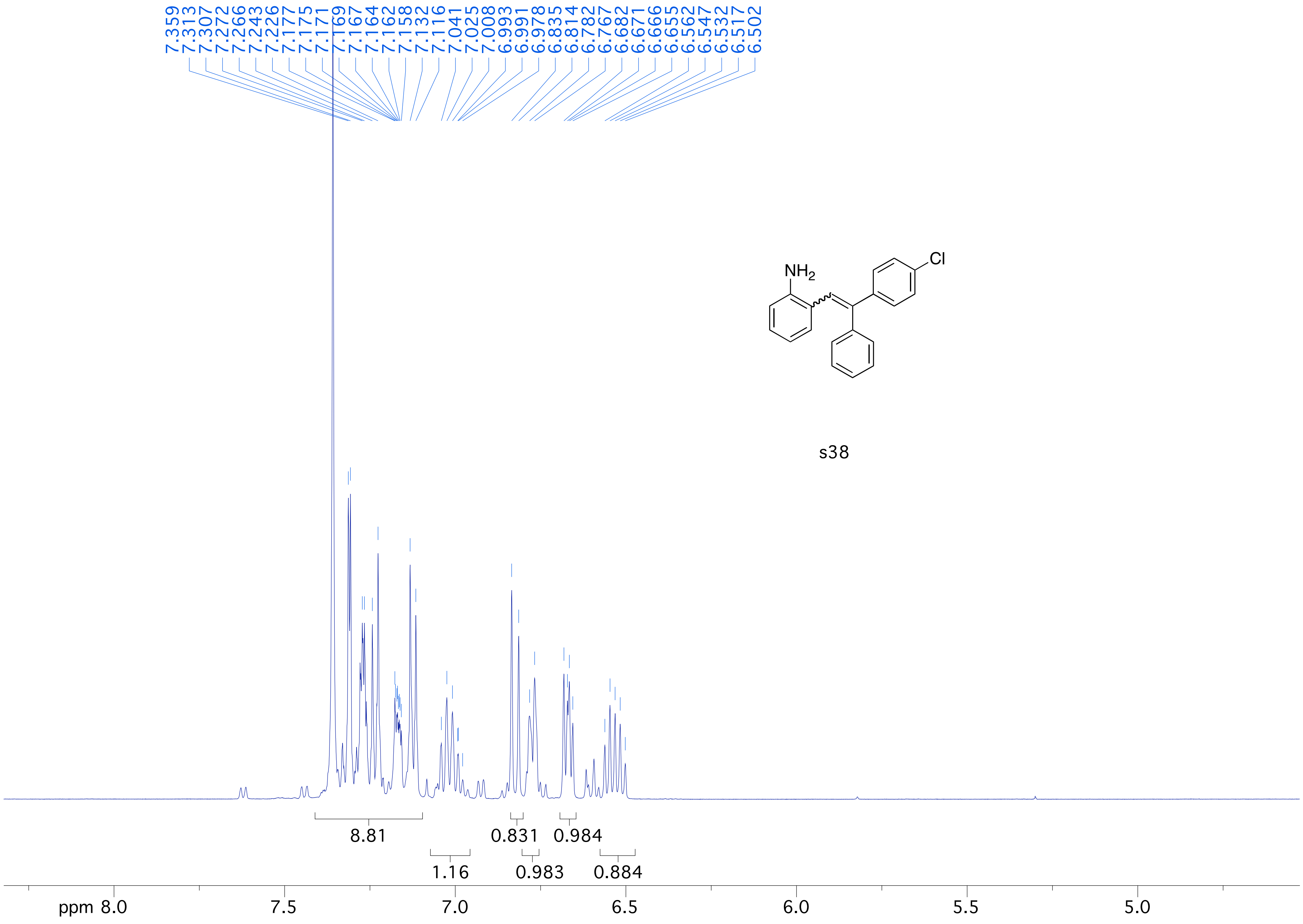
7.359
7.313
7.307
7.272
7.266
7.243
7.226
7.177
7.175
7.171
7.169
7.167
7.164
7.162
7.158
7.132
7.116
7.041
7.025
7.008
6.993
6.991
6.978
6.835
6.814
6.782
6.767
6.682
6.671
6.666
6.655
6.562
6.547
6.532
6.517
6.502
NH2
Cl
s38
8.81
0.831
0.984
1.16
0.983
0.884
ppm 8.0
7.5
7.0
6.5
6.0
5.5
5.0

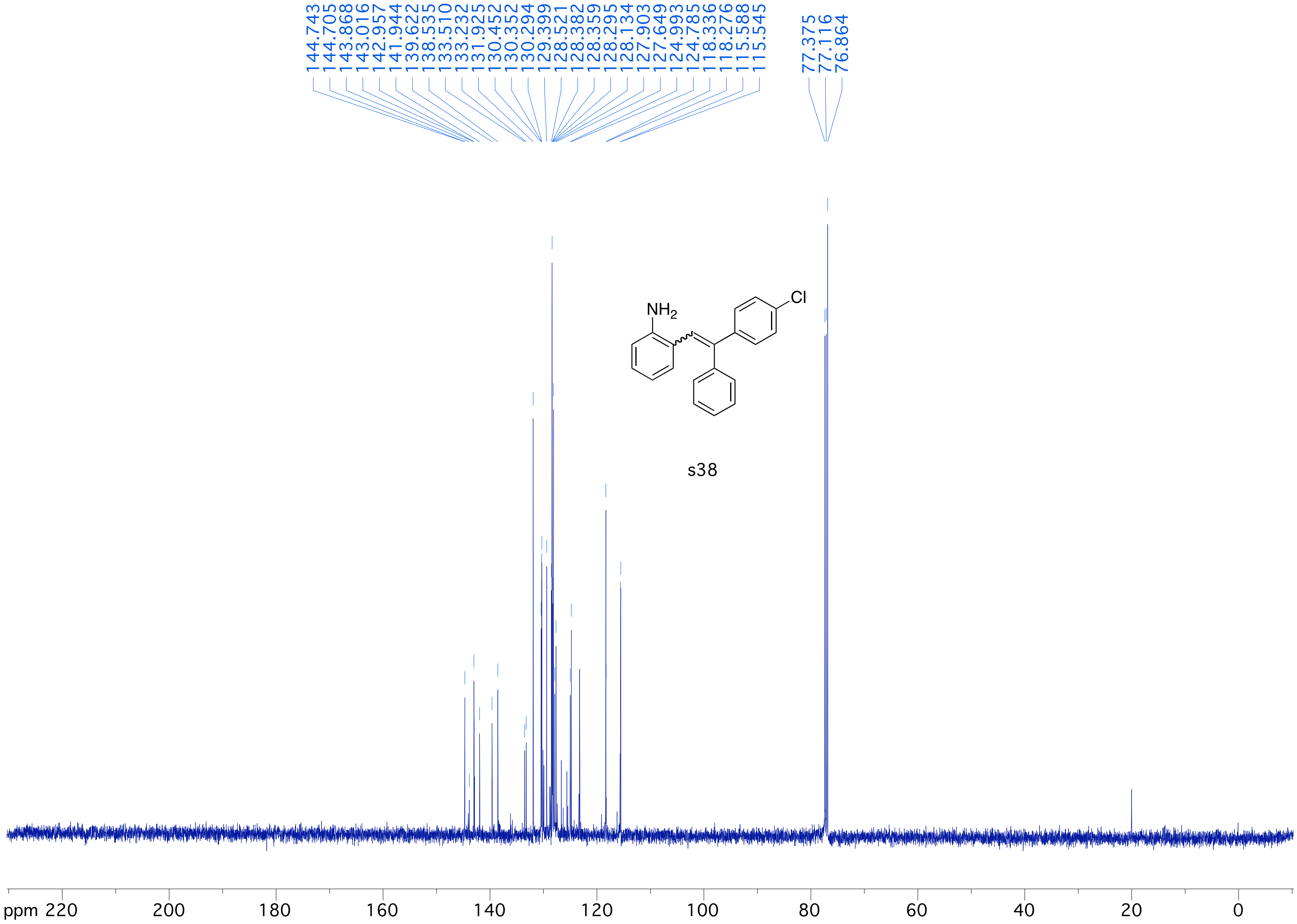
144.743
144.705
143.868
143.016
142.957
141.944
139.622
138.535
133.510
133.232
131.925
130.452
130.352
130.294
129.399
128.521
128.382
128.359
128.295
128.134
127.903
127.649
124.993
124.785
118.336
118.276
115.588
115.545
77.375
77.116
76.864
NH2
Cl
s38
ppm 220
200
180
160
140
120
100
80
60
40
20
0

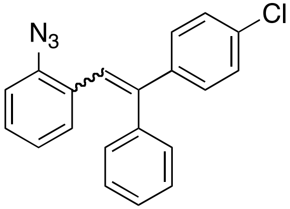

27d

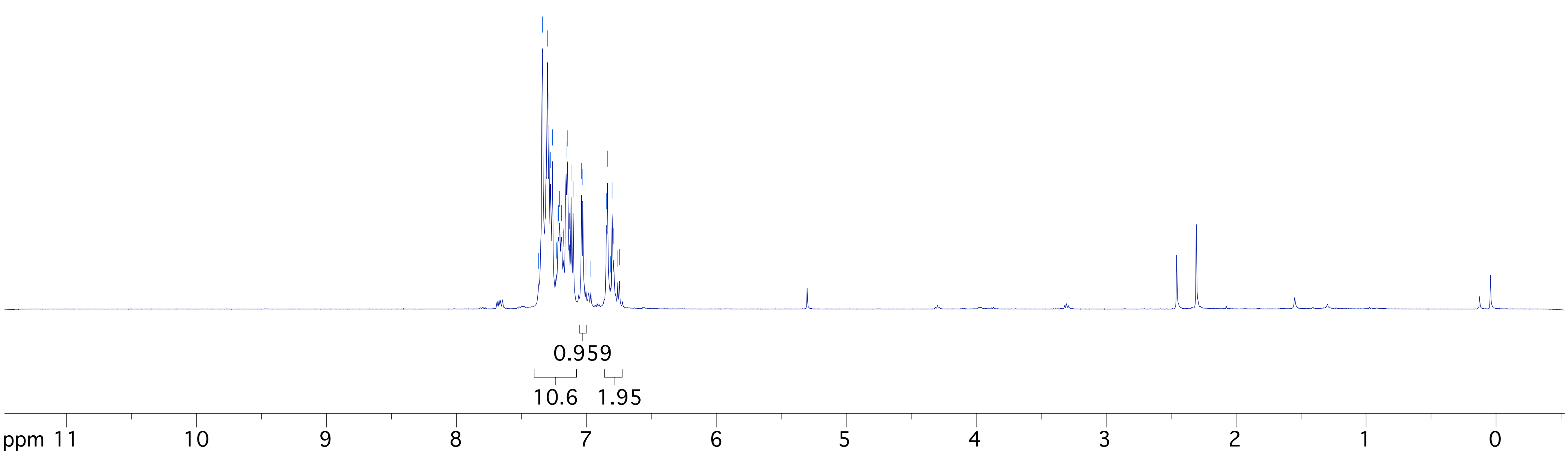

7.366
7.338
7.317
7.310
7.299
7.287
7.276
7.260
7.231
7.231
7.218
7.213
7.206
7.191
7.178
7.173
7.156
7.146
7.131
7.118
7.101
7.036
7.027
7.003
6.965
6.843
6.836
6.814
6.801
6.789
6.758
6.745
0.959
10.6
1.95
ppm 11
10
9
8
7
6
5
4
3
2
1
0

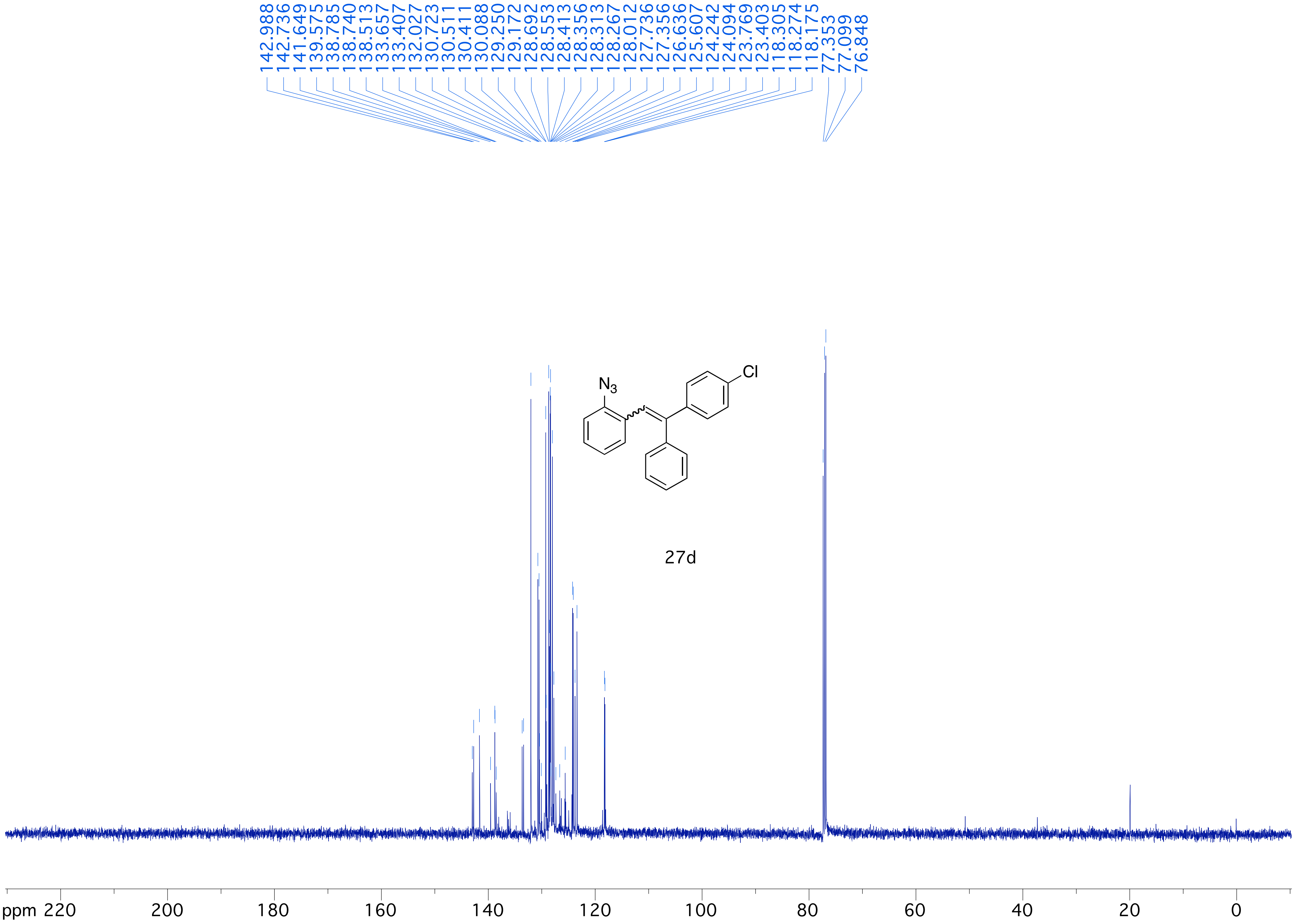
142.988
142.736
141.649
139.575
138.785
138.740
138.513
133.657
133.407
132.027
130.723
130.511
130.411
130.088
129.250
129.172
128.692
128.553
128.413
128.356
128.313
128.267
128.012
127.736
127.356
126.636
125.607
124.242
124.094
123.769
123.403
118.305
118.274
118.175
77.353
77.099
76.848
N3
Cl
27d
ppm 220
200
180
160
140
120
100
80
60
40
20
0

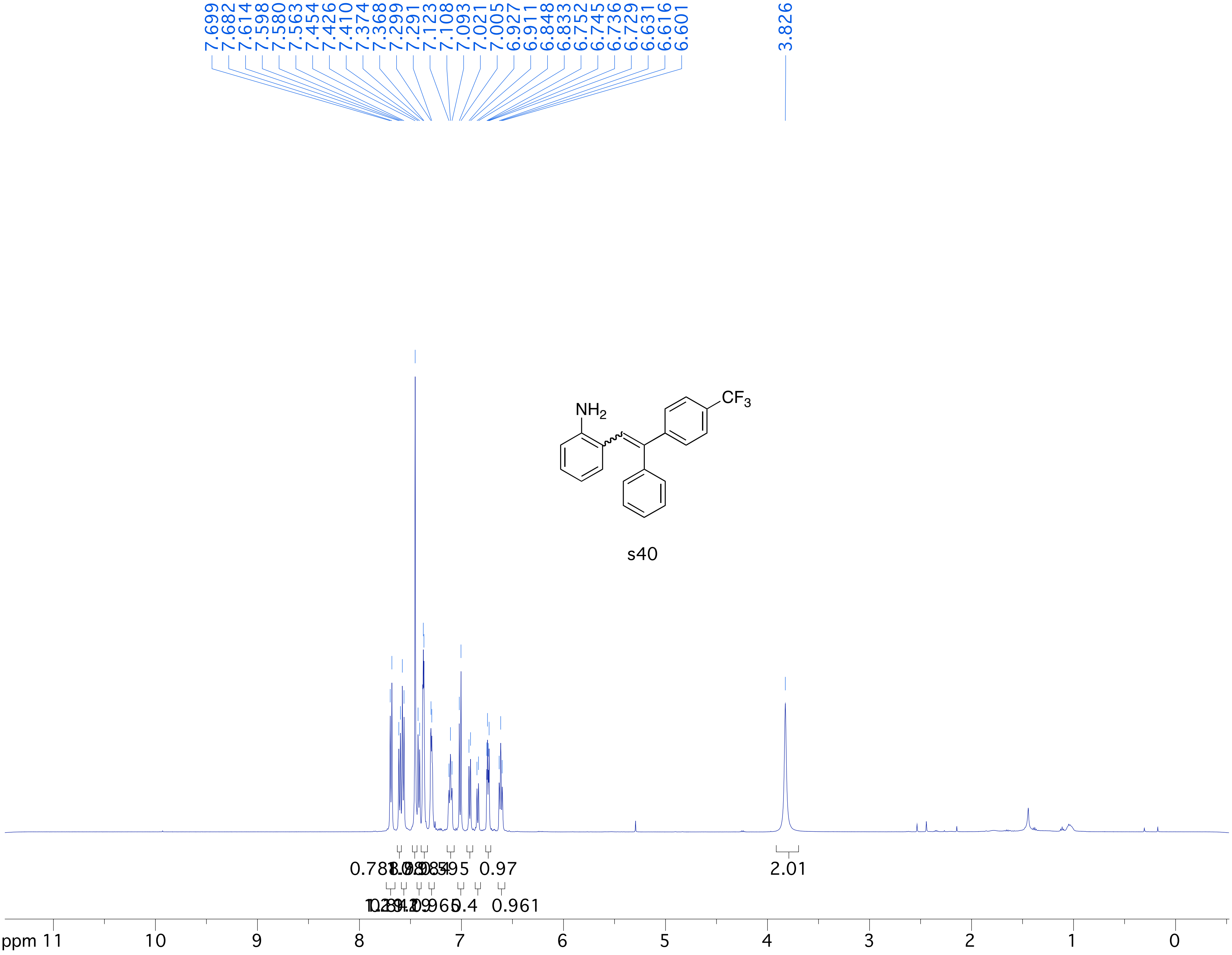
7.699
7.682
7.614
7.598
7.580
7.563
7.454
7.426
7.410
7.374
7.368
7.299
7.291
7.123
7.108
7.093
7.021
7.005
6.927
6.911
6.848
6.833
6.752
6.745
6.736
6.729
6.631
6.616
6.601
3.826
NH_2
CF_3
0.97
2.01
0.961
ppm 11
10
9
8
7
6
5
4
3
2
1
0

s40

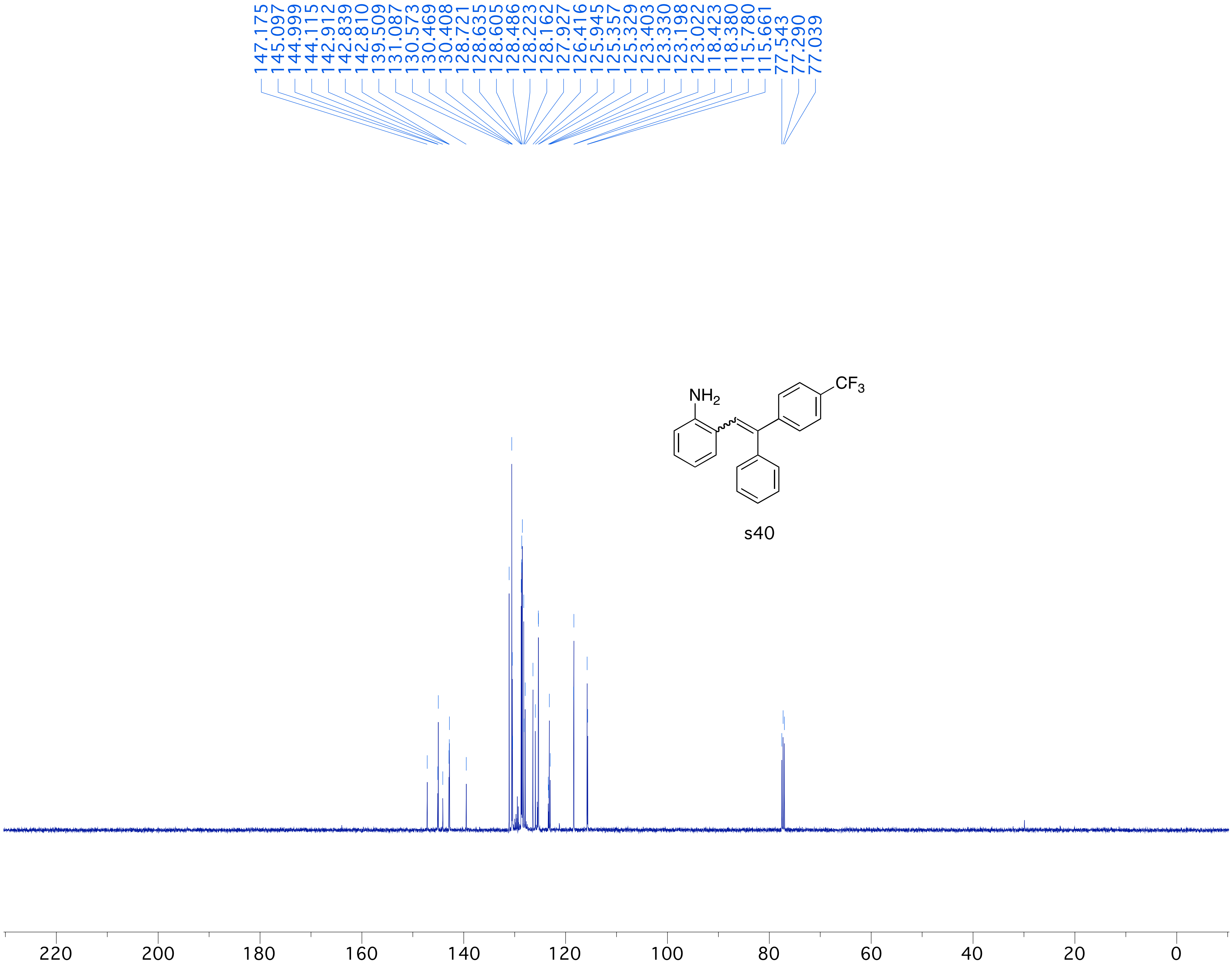
147.175
145.097
144.999
144.115
142.912
142.839
142.810
139.509
131.087
130.573
130.469
130.408
128.721
128.635
128.605
128.486
128.223
128.162
127.927
126.416
125.945
125.357
125.329
123.403
123.330
123.198
123.022
118.423
118.380
115.780
115.661
77.543
77.290
77.039
NH2
CF3
s40
220
200
180
160
140
120
100
80
60
40
20
0

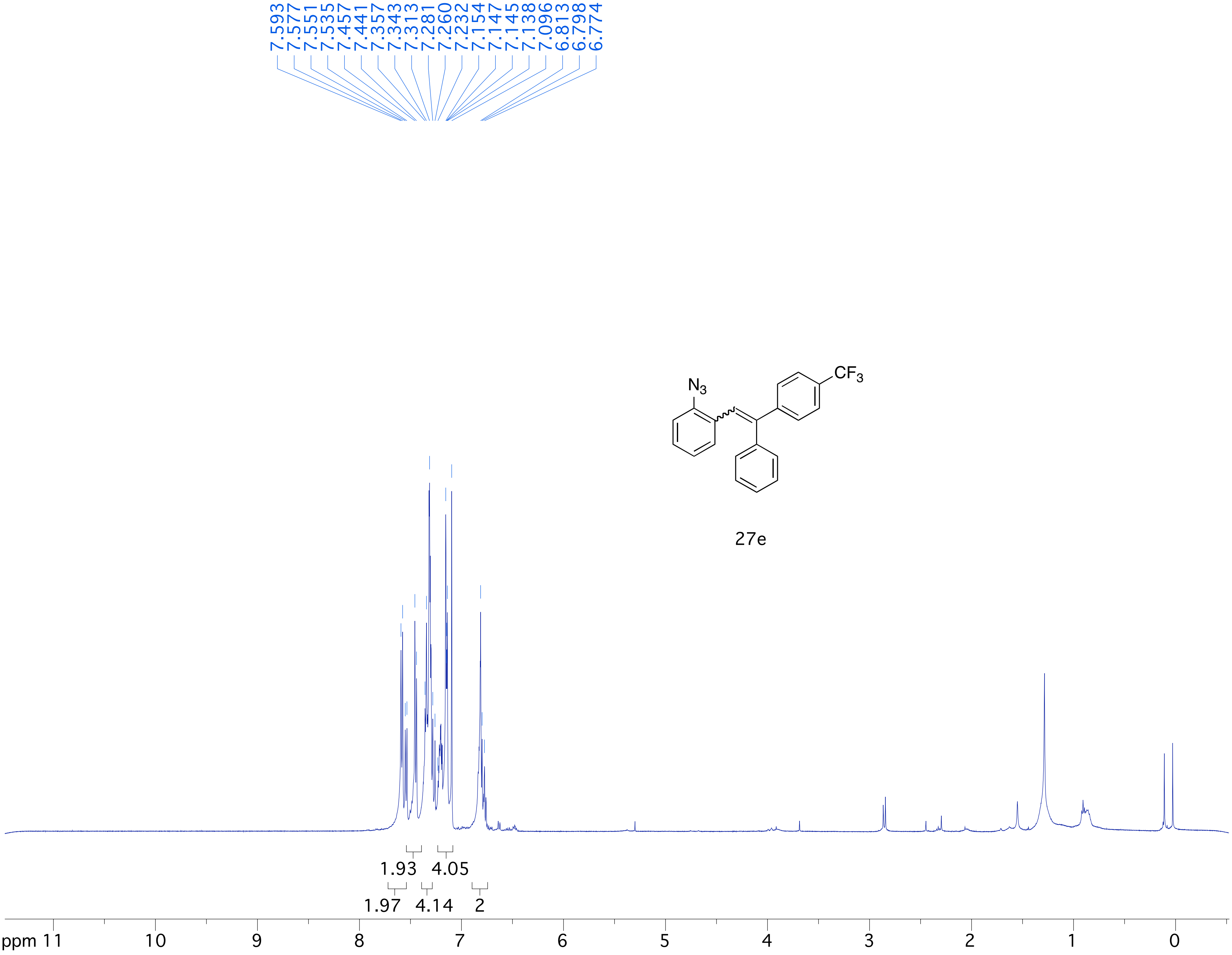

7.593
7.577
7.551
7.535
7.457
7.441
7.357
7.343
7.313
7.281
7.260
7.232
7.154
7.147
7.145
7.138
7.096
6.813
6.798
6.774
N3
CF3
1.93
4.05
1.97
4.14
2
ppm 11
10
9
8
7
6
5
4
3
2
1
0

27e

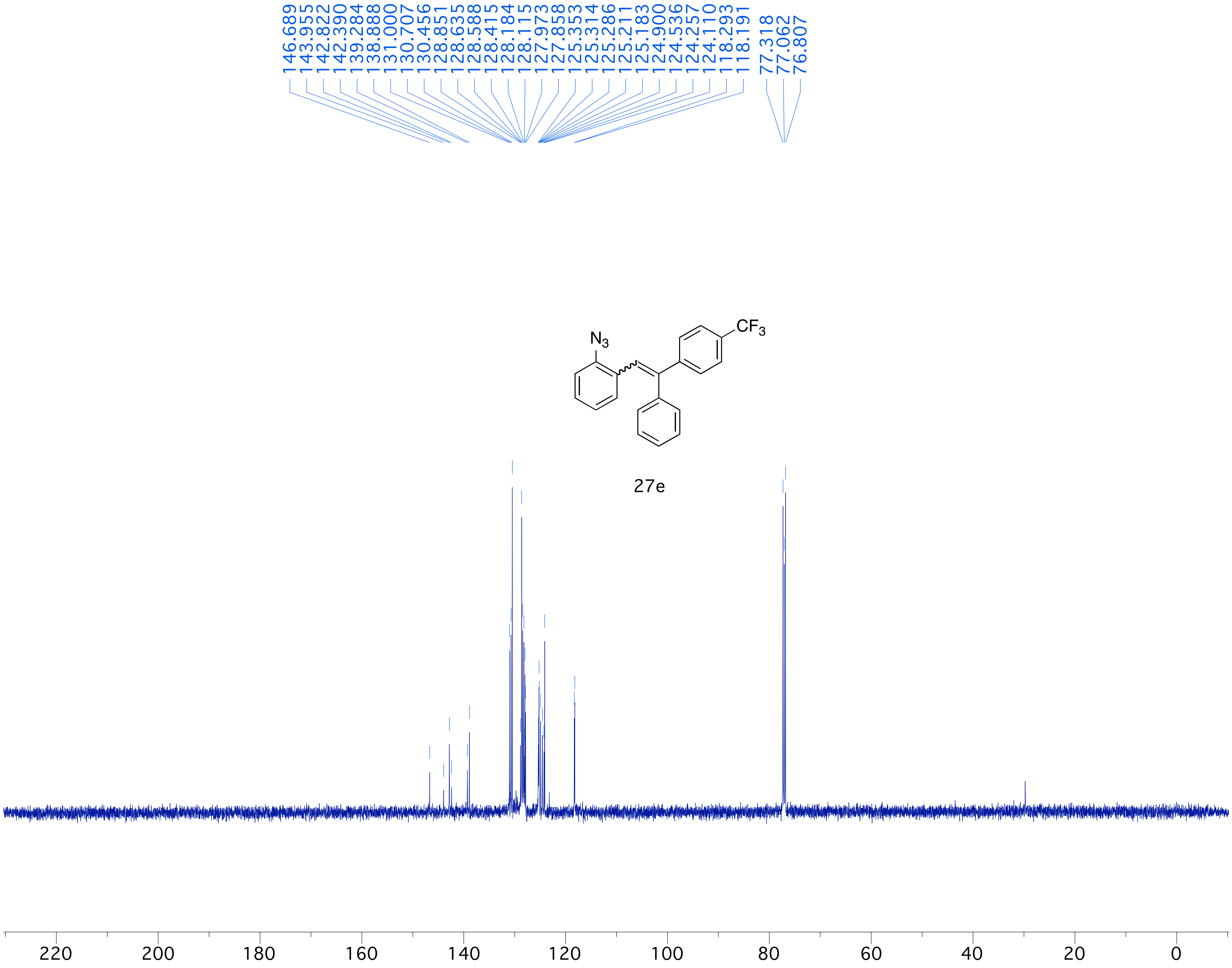
146.689
143.955
142.822
142.390
139.284
138.888
131.000
130.707
130.456
128.851
128.635
128.588
128.415
128.184
128.115
127.973
127.858
125.353
125.314
125.286
125.211
125.183
124.900
124.536
124.257
124.110
118.293
118.191
77.318
77.062
76.807
N3
CF3
220
200
180
160
140
120
100
80
60
40
20
0

27e

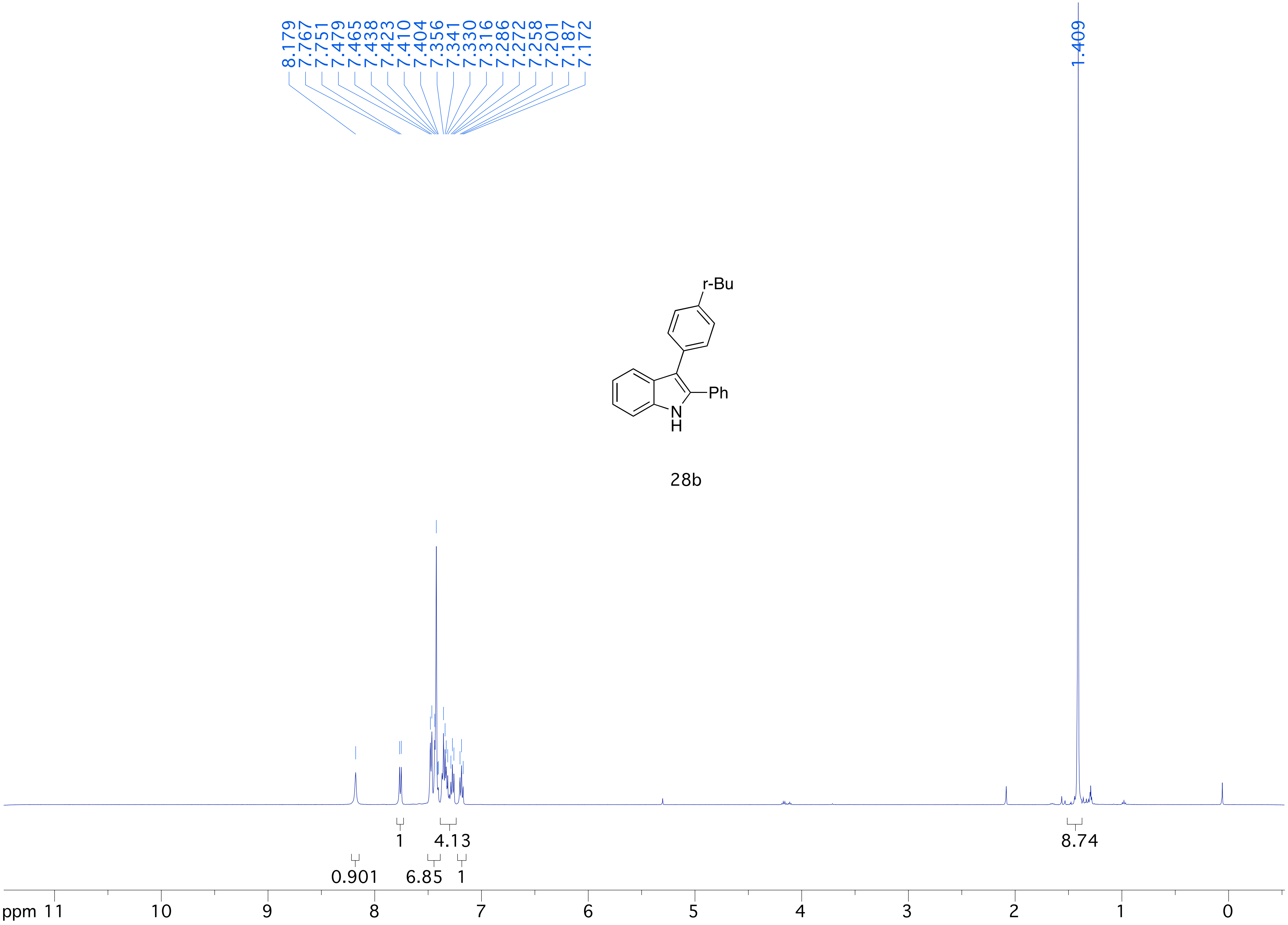
8.179
7.767
7.751
7.479
7.465
7.438
7.423
7.410
7.404
7.356
7.341
7.330
7.316
7.286
7.272
7.258
7.201
7.187
7.172
1.409
r-Bu
Ph
N
H
1
4.13
8.74
0.901
6.85
1
ppm 11
10
9
8
7
6
5
4
3
2
1
0

28b

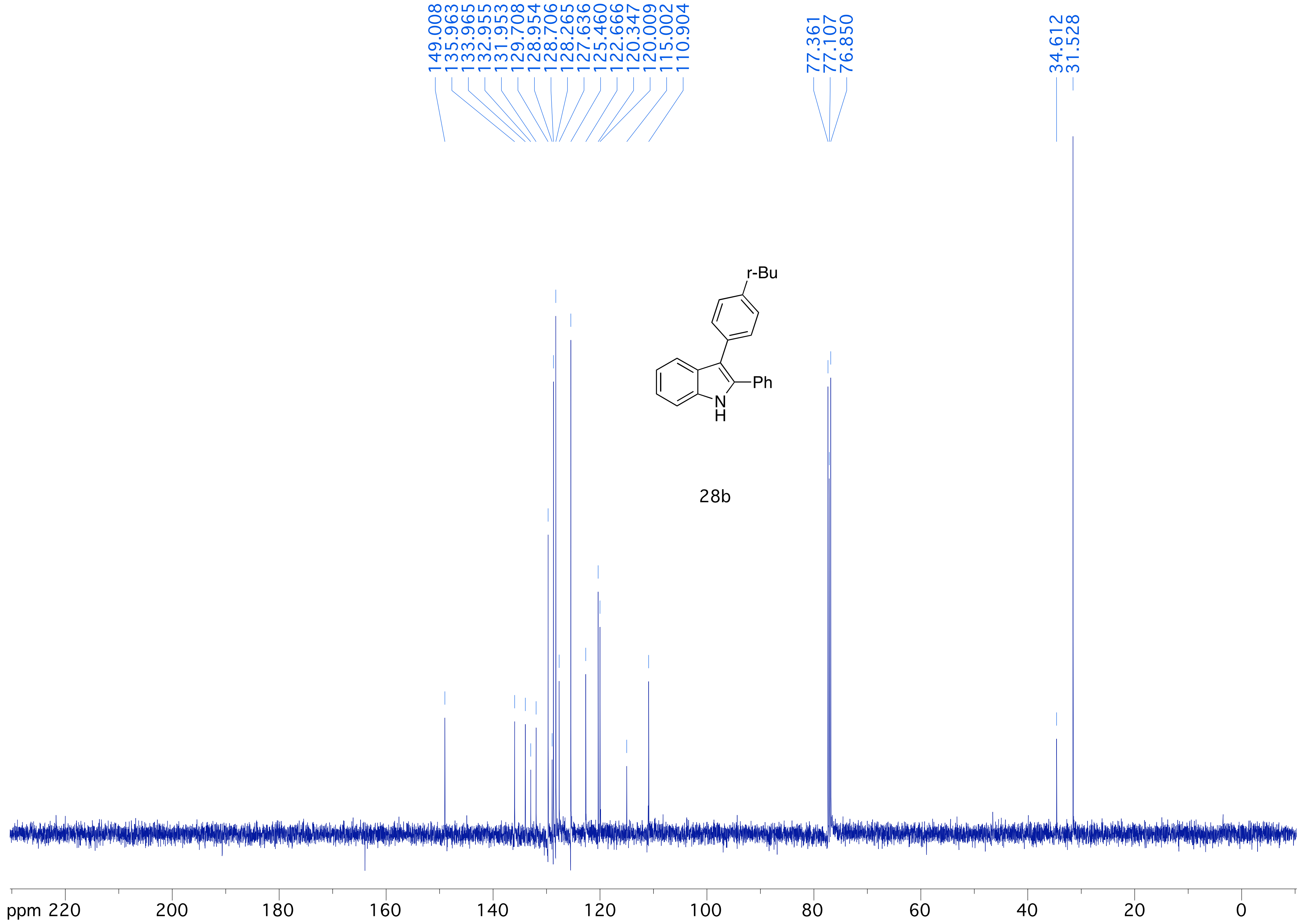
149.008
135.963
133.965
132.955
131.953
129.708
128.954
128.706
128.265
127.636
125.460
122.666
120.347
120.009
115.002
110.904
77.361
77.107
76.850
34.612
31.528
r-Bu
Ph
N
H
28b
ppm 220
200
180
160
140
120
100
80
60
40
20
0

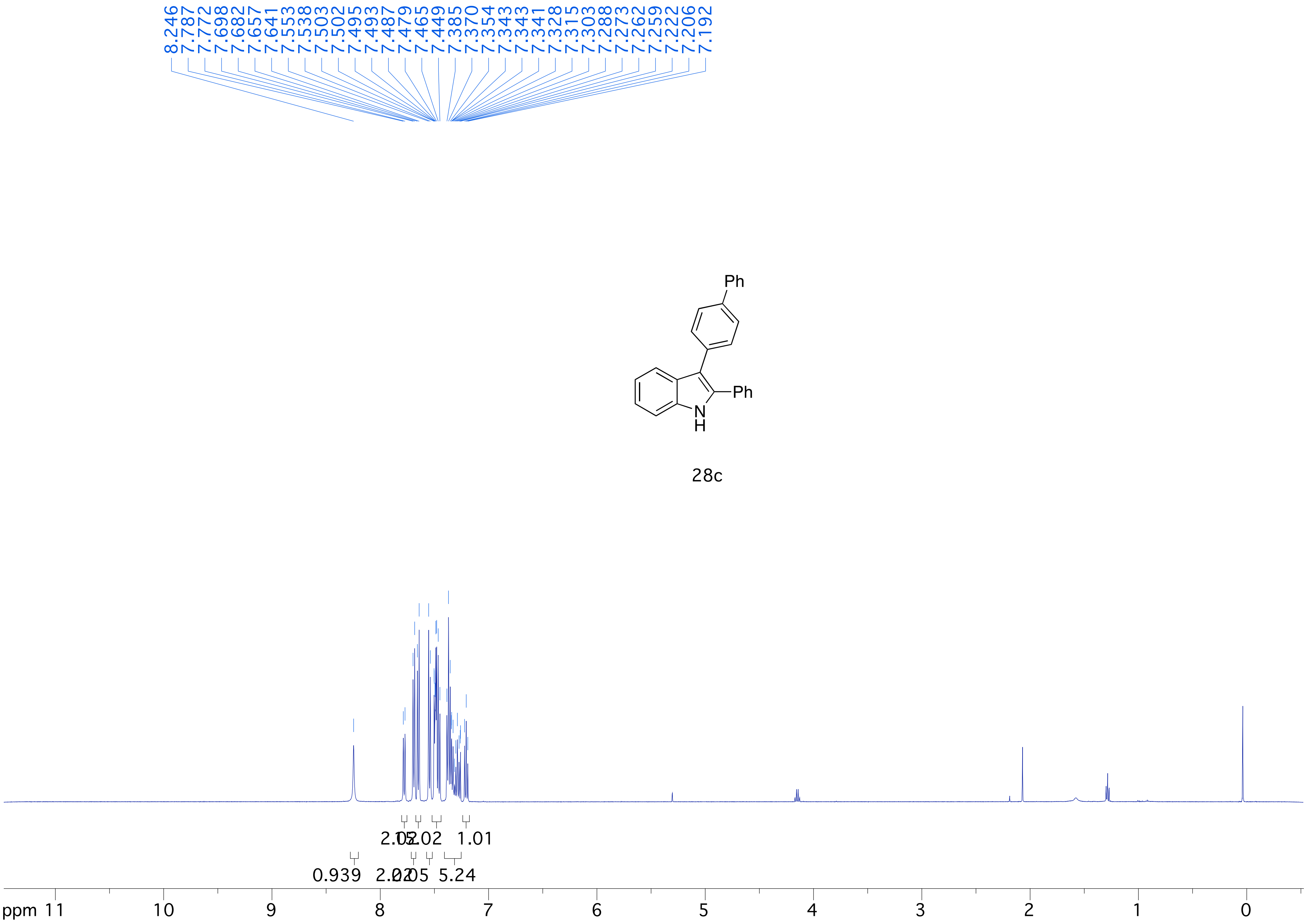

Ph
Ph
N
H
8.246
7.787
7.772
7.698
7.682
7.657
7.641
7.553
7.538
7.503
7.502
7.495
7.493
7.487
7.479
7.465
7.449
7.385
7.370
7.354
7.343
7.343
7.341
7.328
7.315
7.303
7.288
7.273
7.262
7.259
7.222
7.206
7.192
0.939
5.24
1.01
ppm 11
10
9
8
7
6
5
4
3
2
1
0

28c

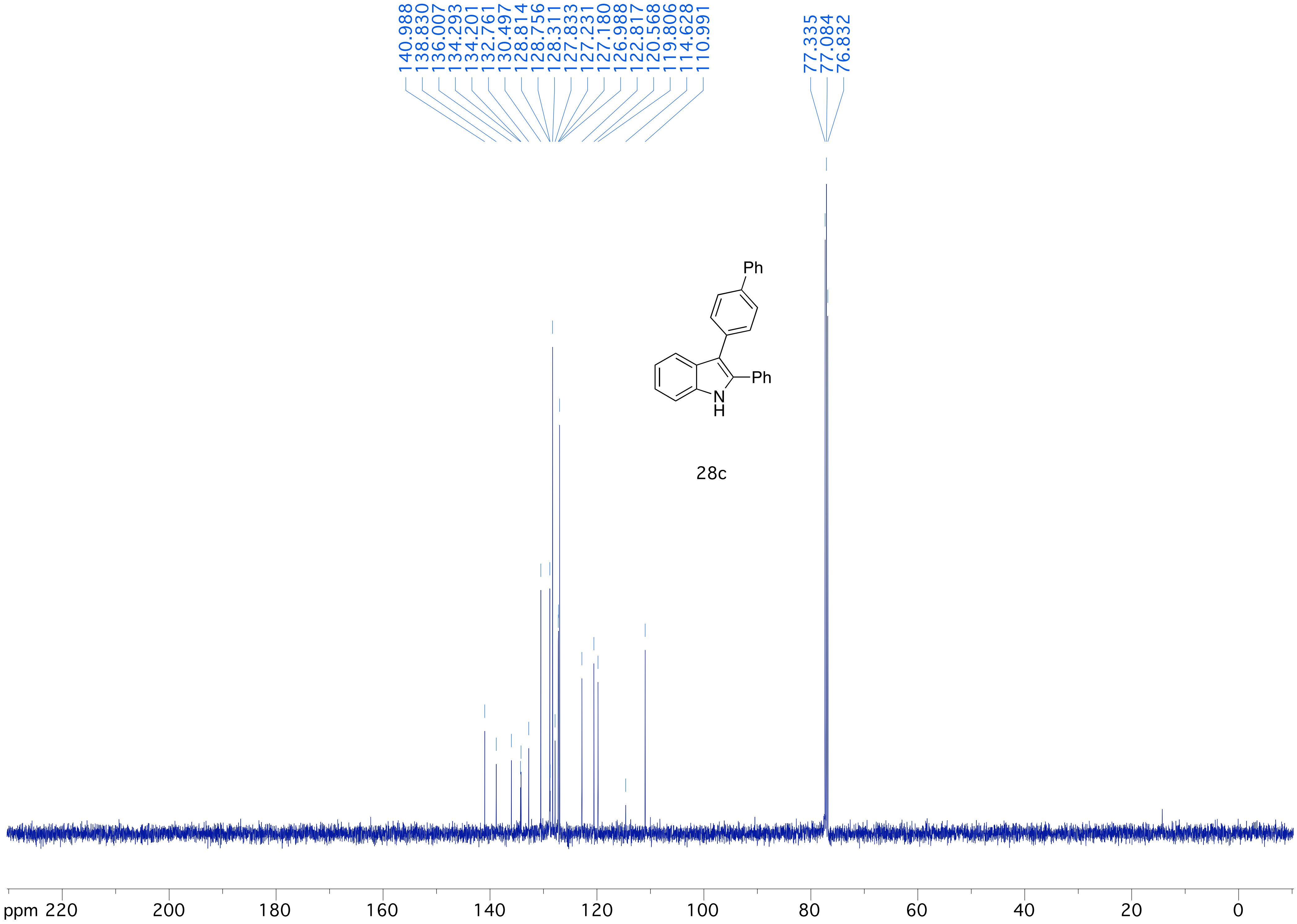
140.988
138.830
136.007
134.293
134.201
132.761
130.497
128.814
128.756
128.311
127.833
127.231
127.180
126.988
122.817
120.568
119.806
114.628
110.991
77.335
77.084
76.832
Ph
Ph
N
H
28c
ppm 220
200
180
160
140
120
100
80
60
40
20
0

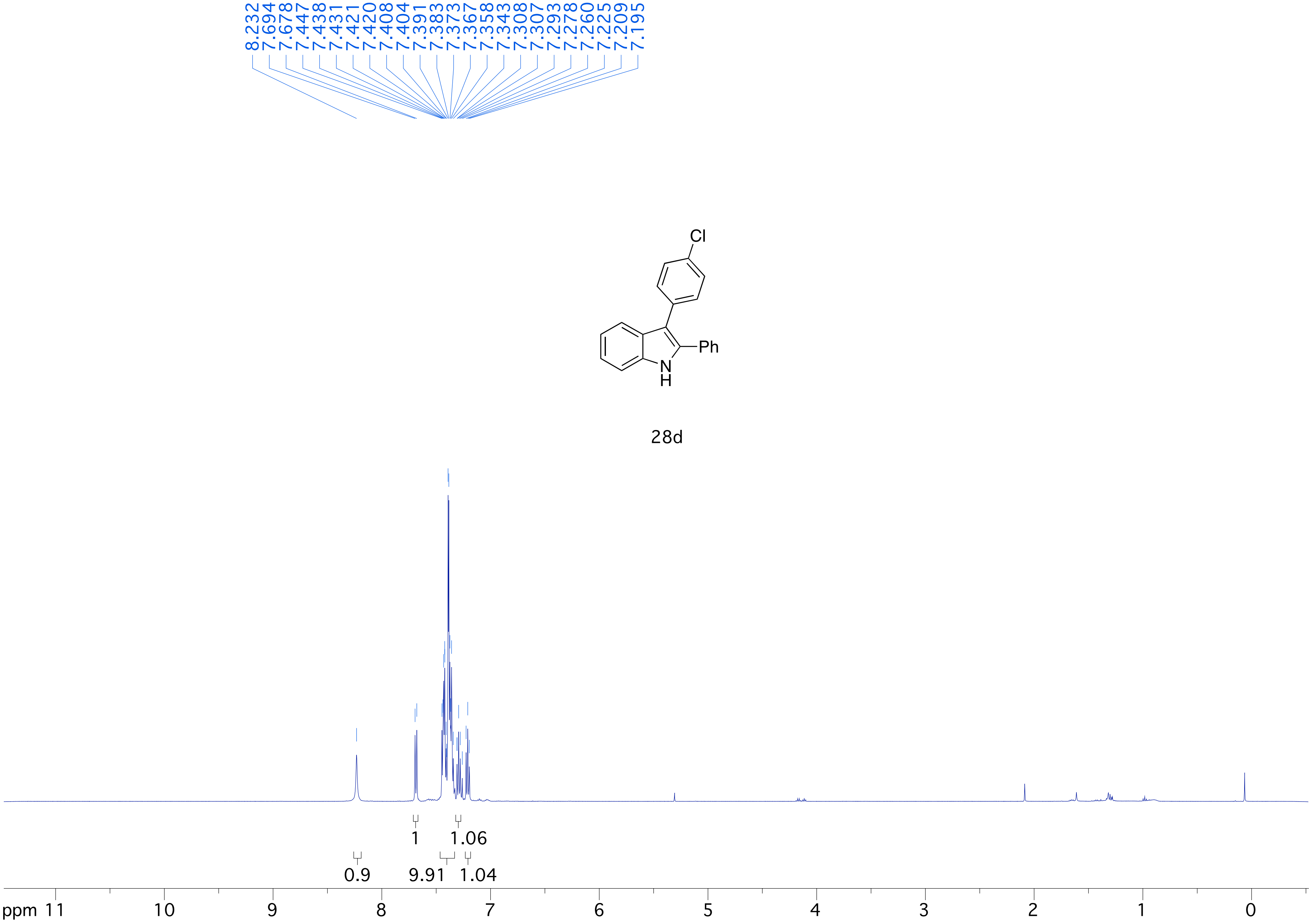
8.232
7.694
7.678
7.447
7.438
7.431
7.421
7.420
7.408
7.404
7.391
7.383
7.373
7.367
7.358
7.343
7.308
7.307
7.293
7.278
7.260
7.225
7.209
7.195
Cl
Ph
N
H
28d
1
1.06
0.9
9.91
1.04
ppm 11
10
9
8
7
6
5
4
3
2
1
0

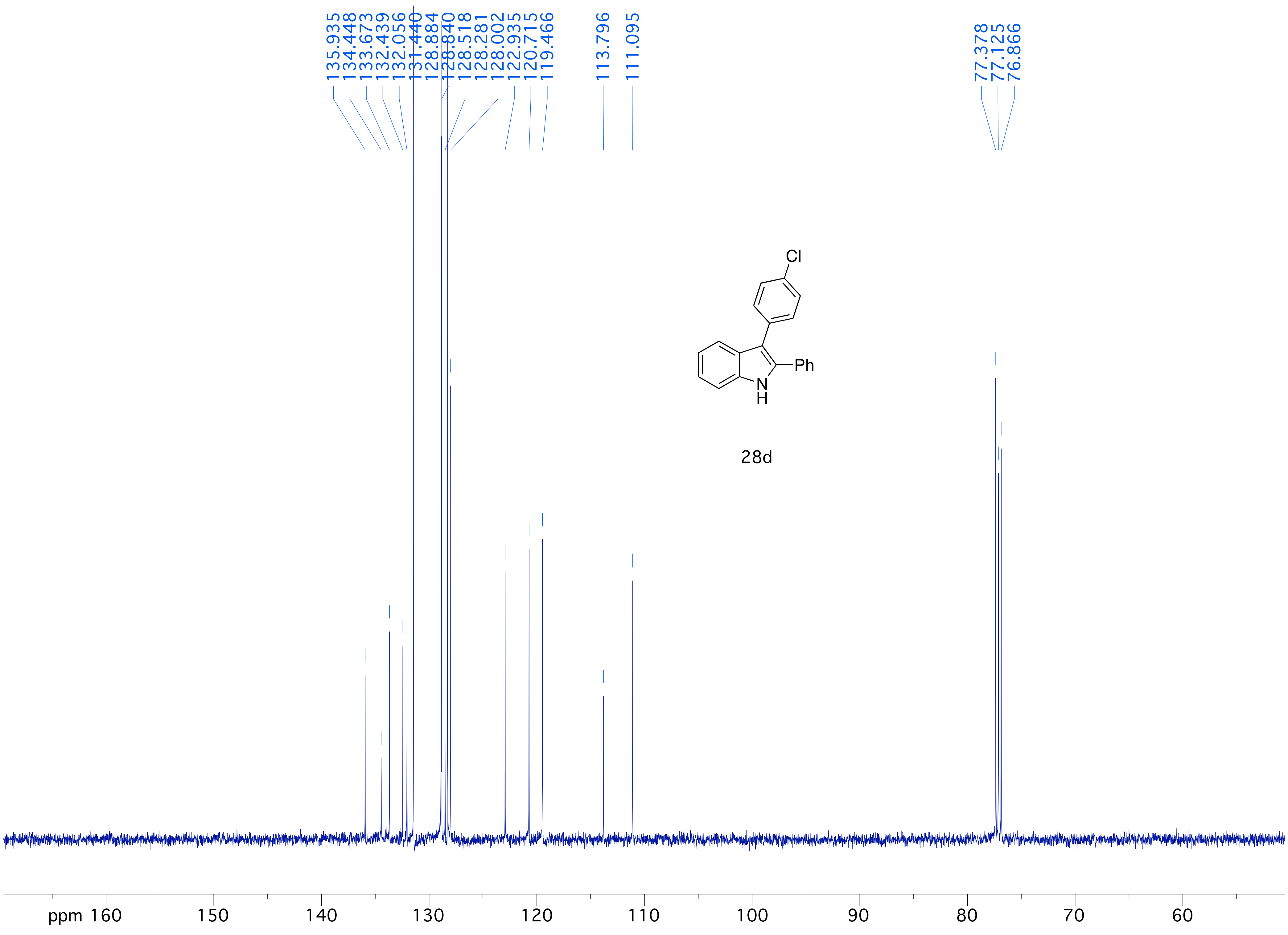
135.935
134.448
133.673
132.439
132.056
131.440
128.884
128.840
128.518
128.281
128.002
122.935
120.715
119.466
113.796
111.095
77.378
77.125
76.866
Cl
Ph
N
H
28d
ppm 160
150
140
130
120
110
100
90
80
70
60

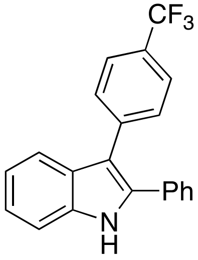
CF3
Ph
N
H

28e

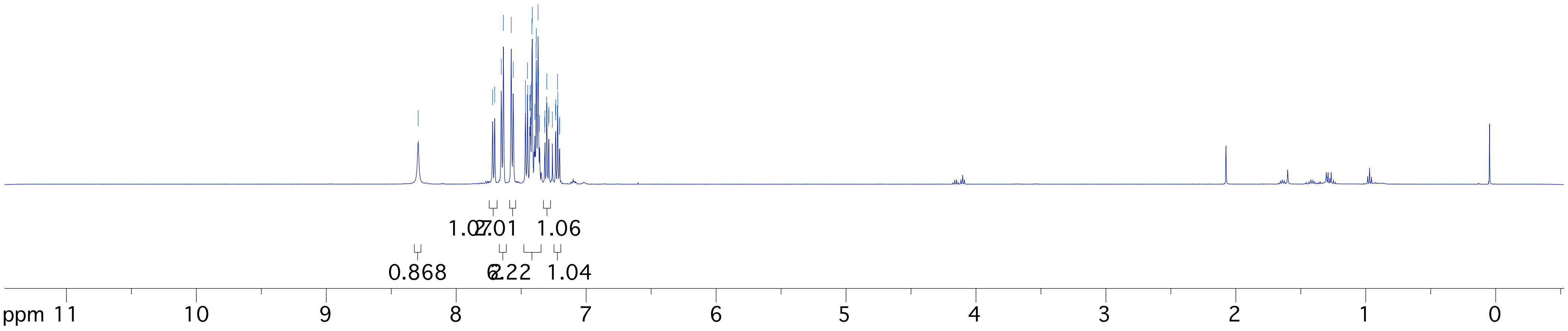
8.293
7.720
7.704
7.653
7.637
7.577
7.561
7.468
7.466
7.451
7.450
7.434
7.430
7.428
7.426
7.421
7.418
7.414
7.395
7.385
7.384
7.381
7.373
7.370
7.367
7.359
7.319
7.317
7.305
7.303
7.301
7.289
7.287
7.260
7.236
7.234
7.222
7.220
7.218
7.206
7.204
1.02
2.01
1.06
0.868
6.22
1.04
ppm 11
10
9
8
7
6
5
4
3
2
1
0

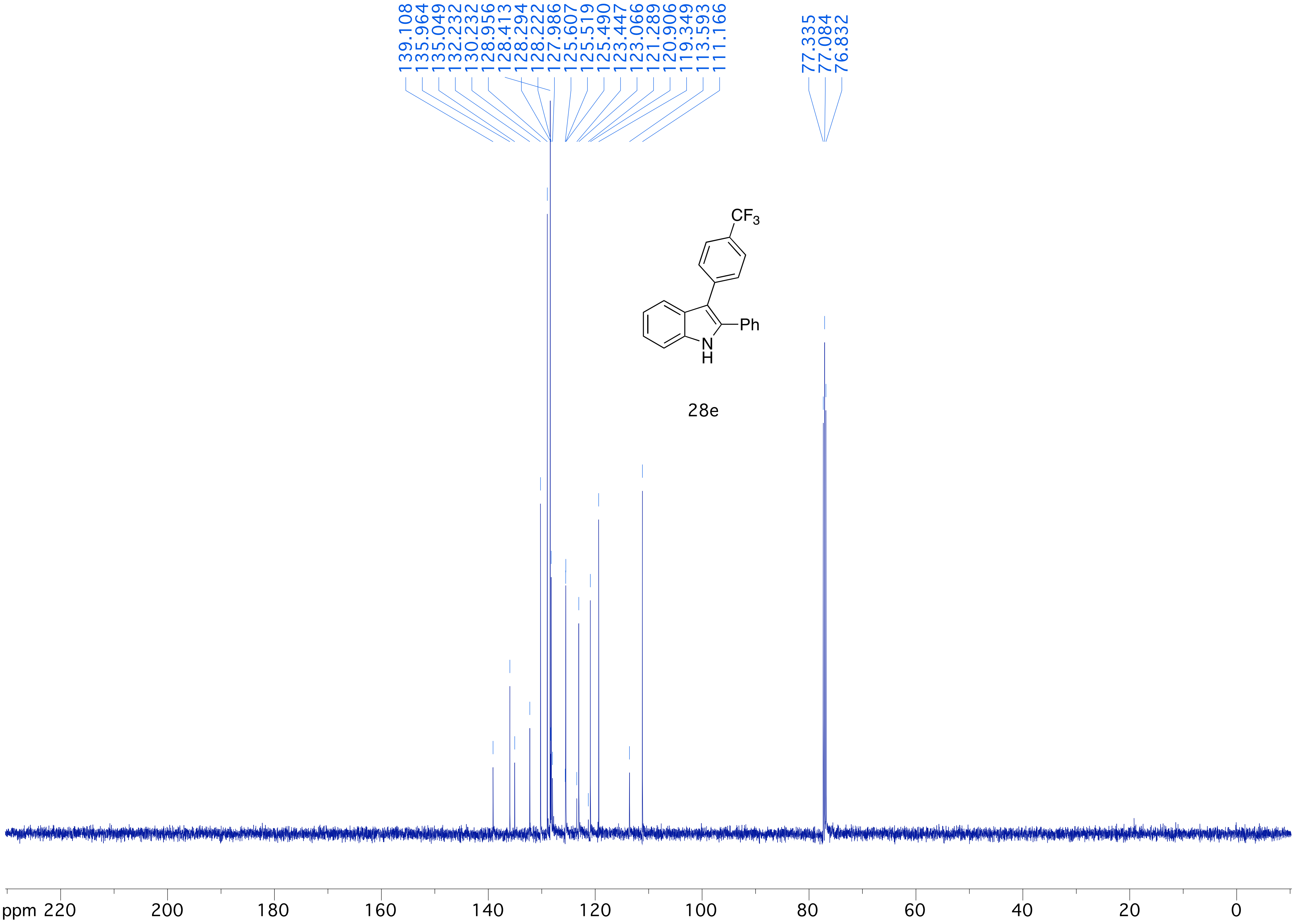

139.108
135.964
135.049
132.232
130.232
128.956
128.413
128.294
128.222
127.986
125.607
125.519
125.490
123.447
123.066
121.289
120.906
119.349
113.593
111.166
77.335
77.084
76.832
CF3
Ph
N
H
28e
ppm 220
200
180
160
140
120
100
80
60
40
20
0

Intramolecular Competition Experiment

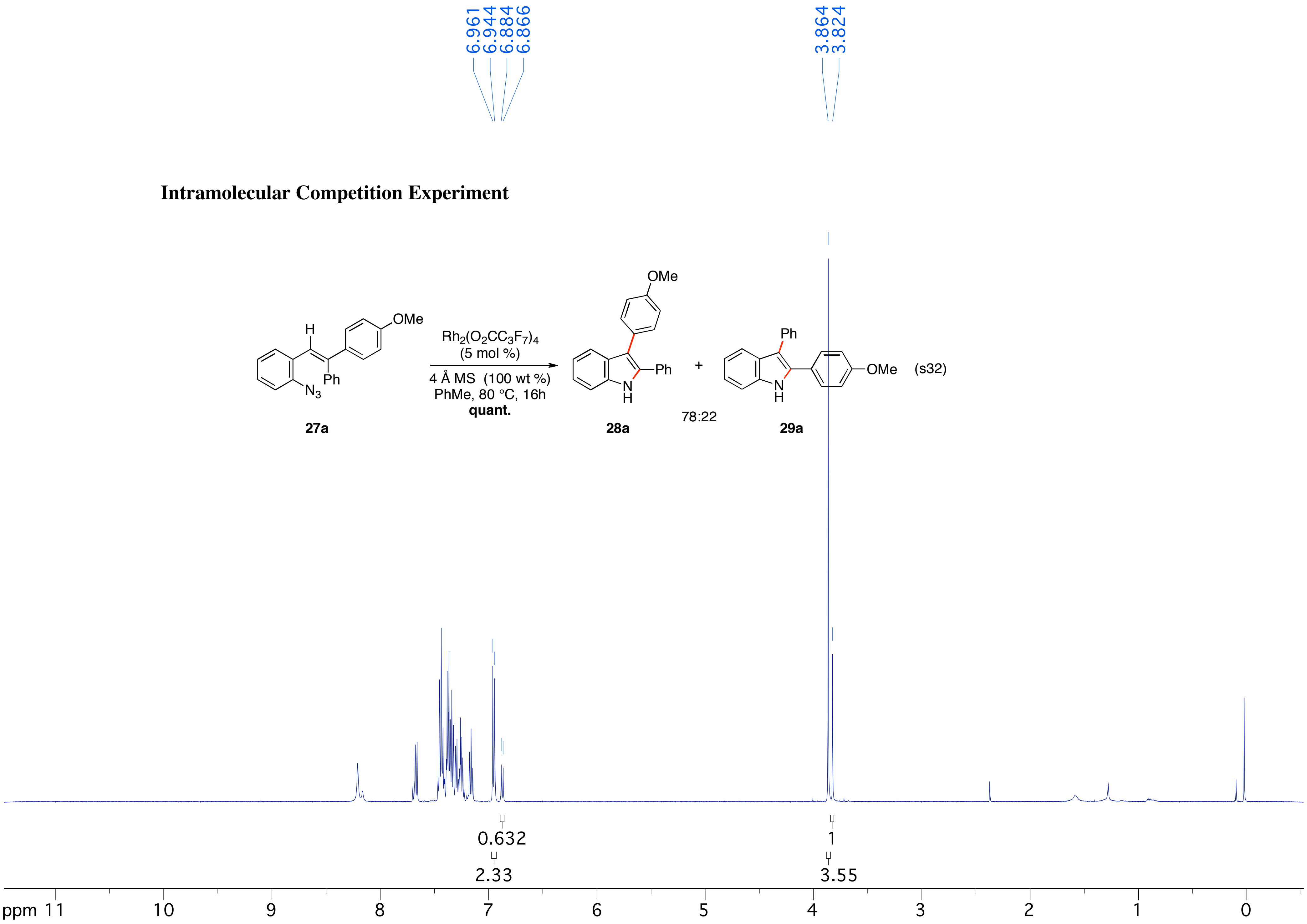

Intramolecular Competition Experiment

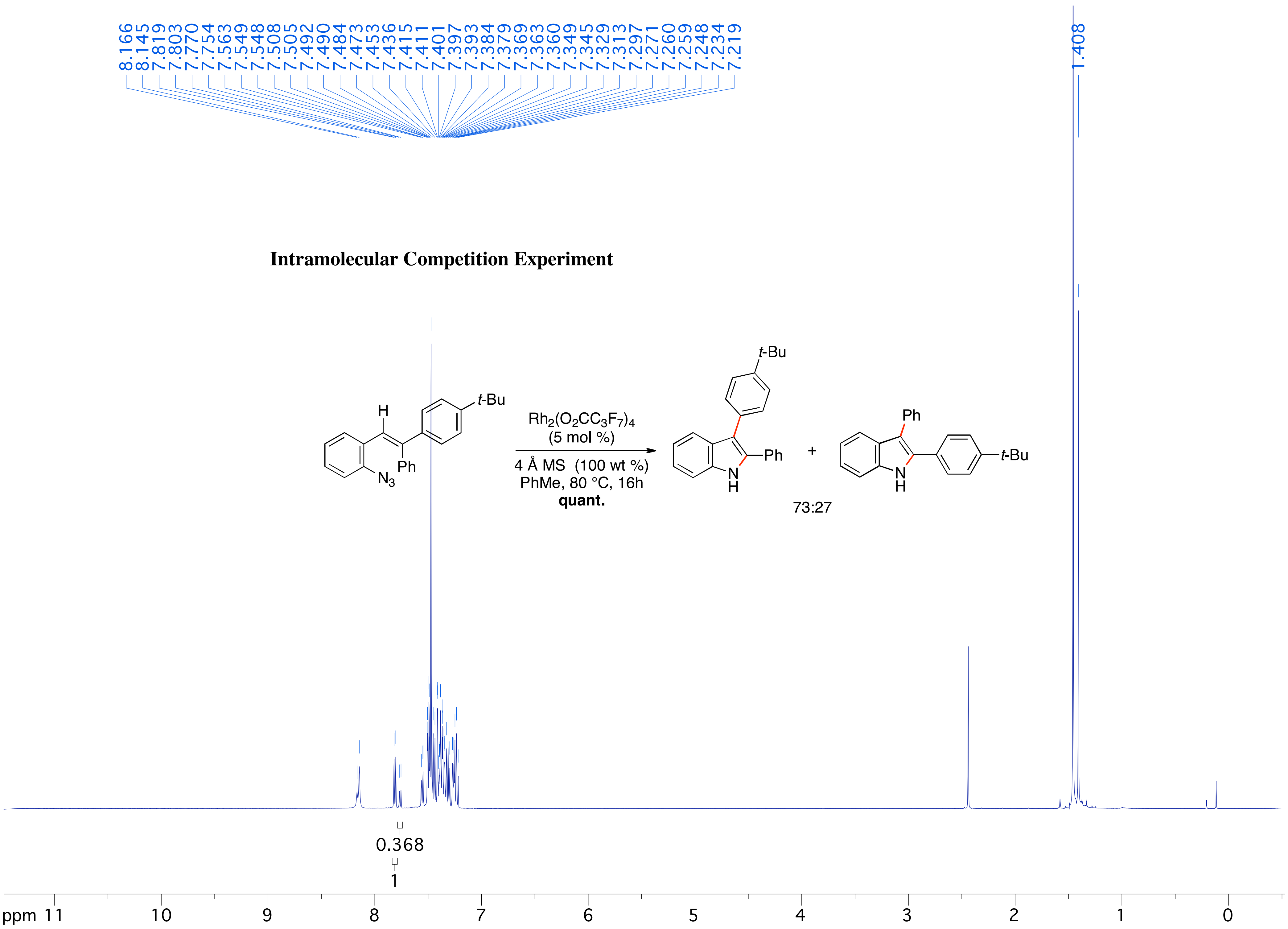

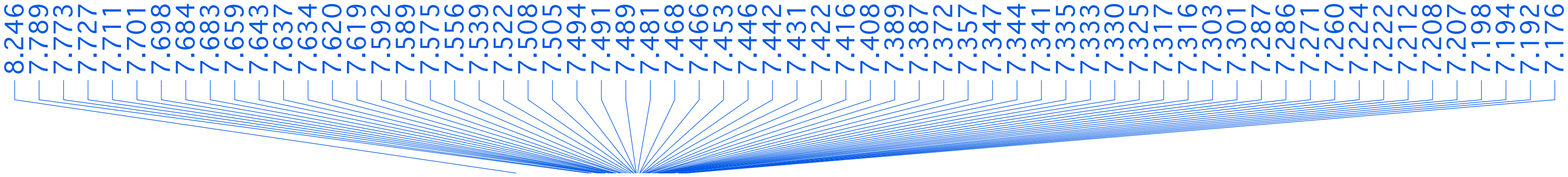

Intramolecular Competition Experiment

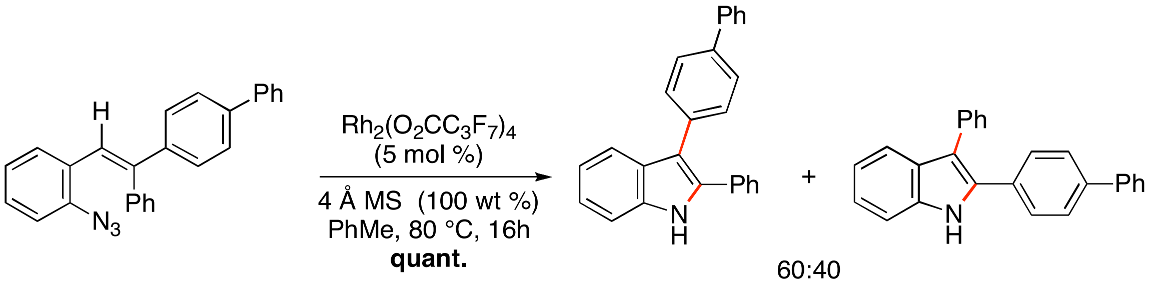

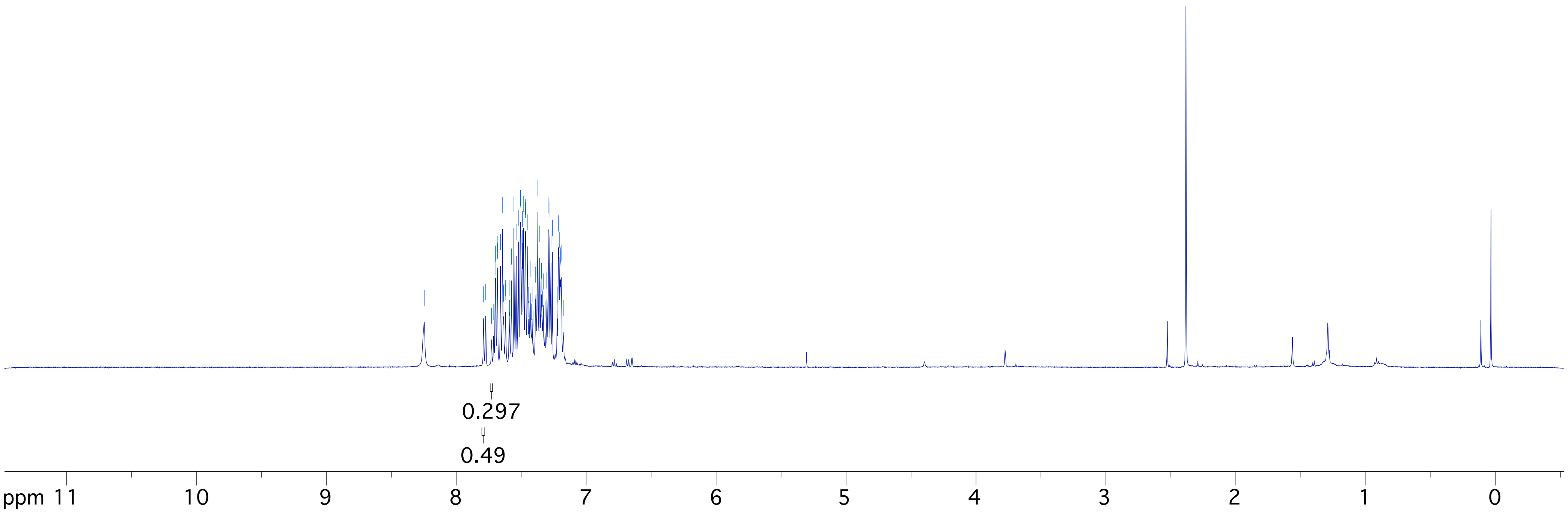

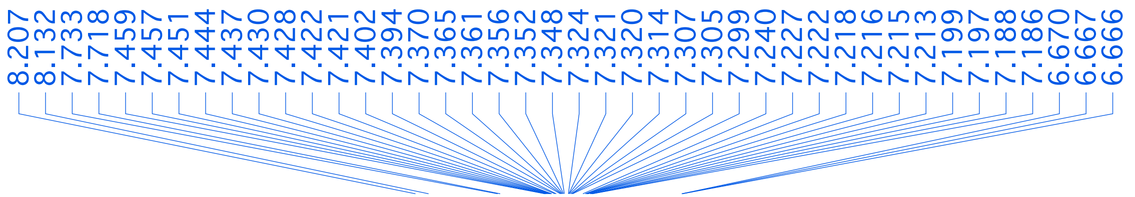

Intramolecular Competition Experiment

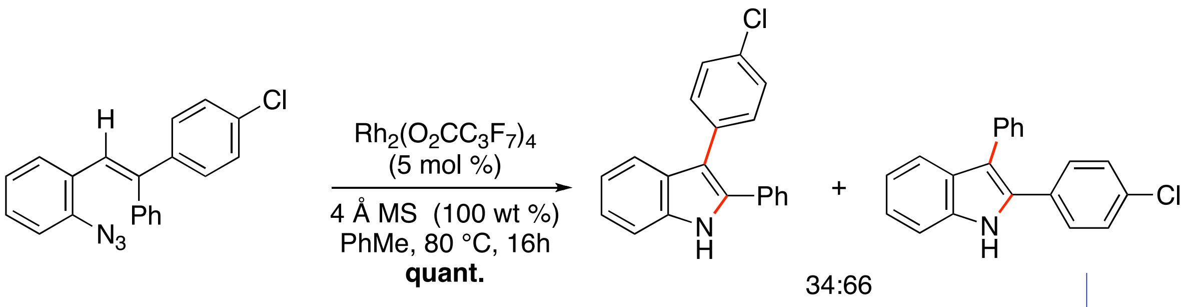

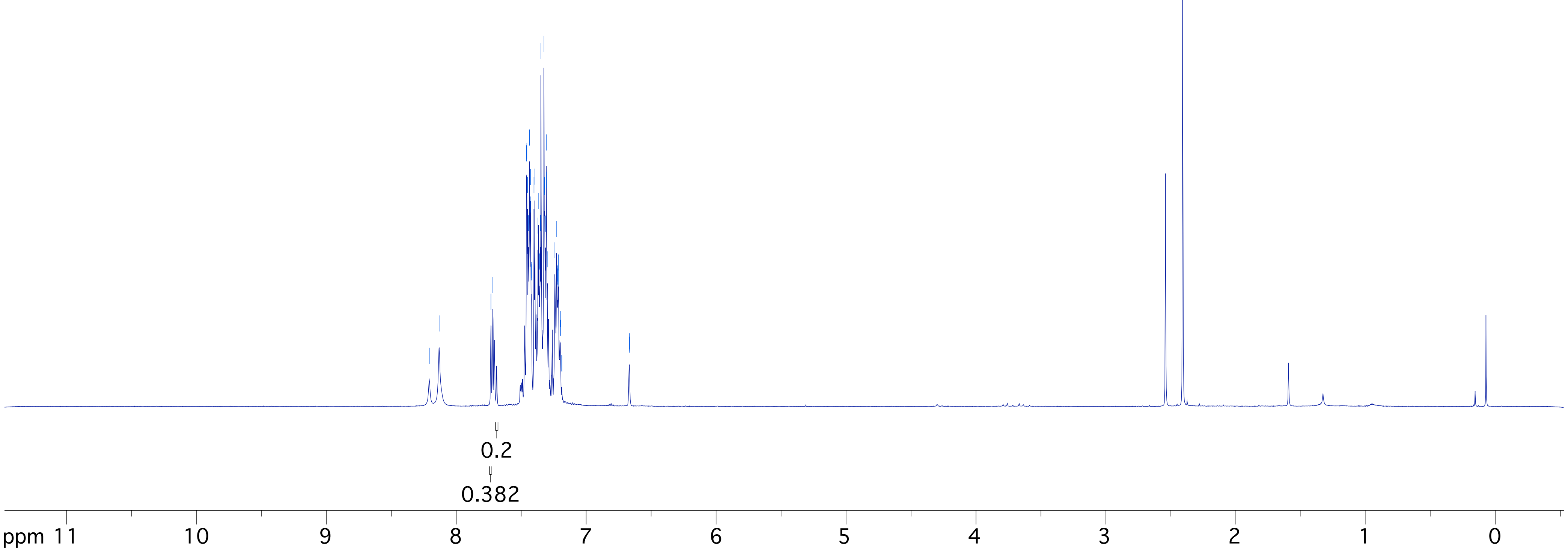

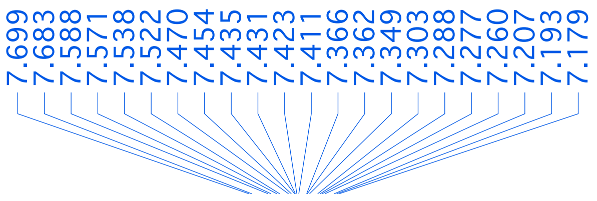

Intramolecular Competition Experiment

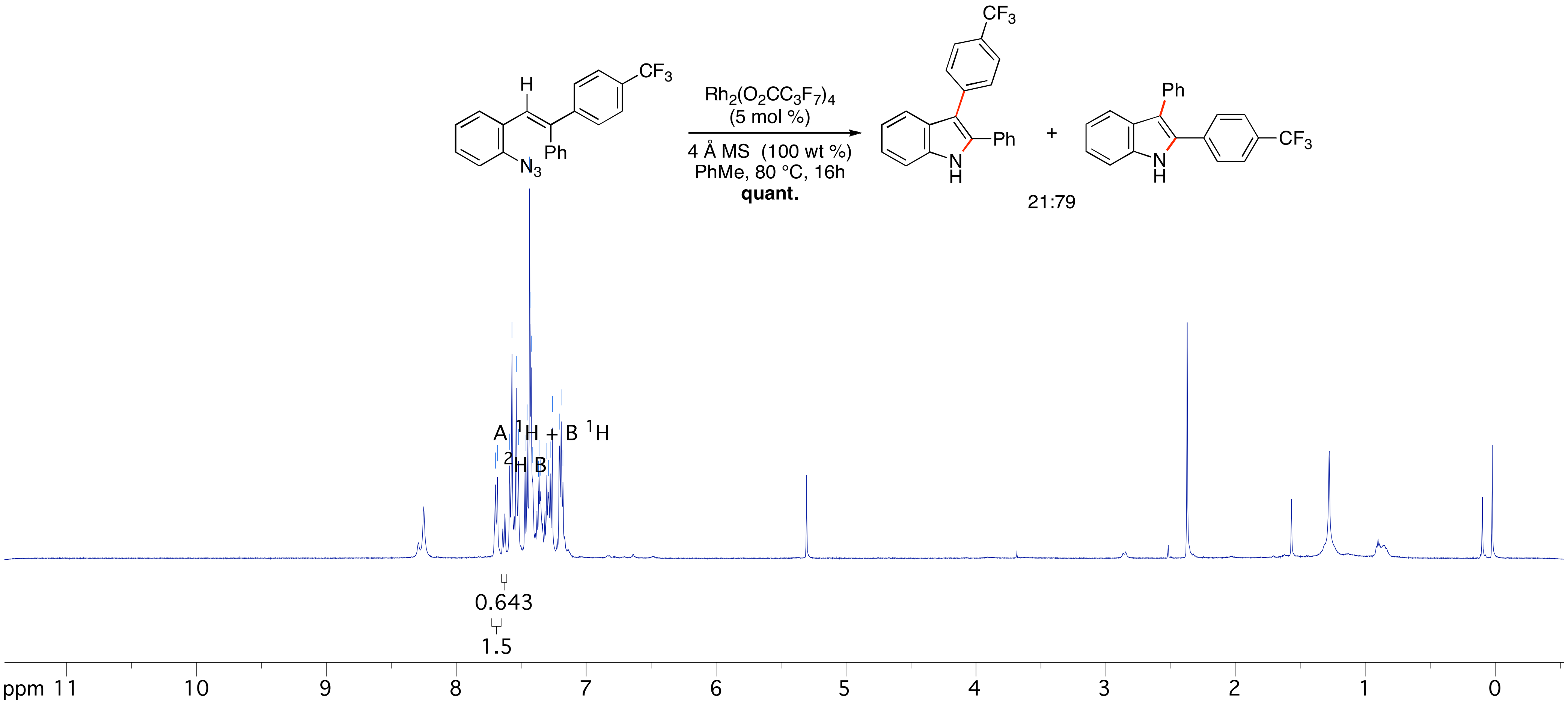

Intermolecular Competition Experiment

5 vs **8a**

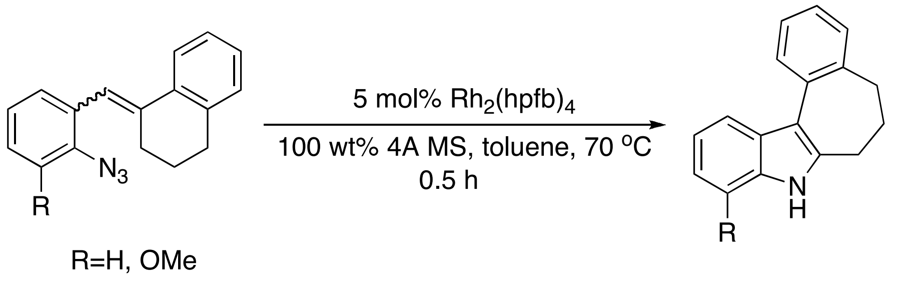

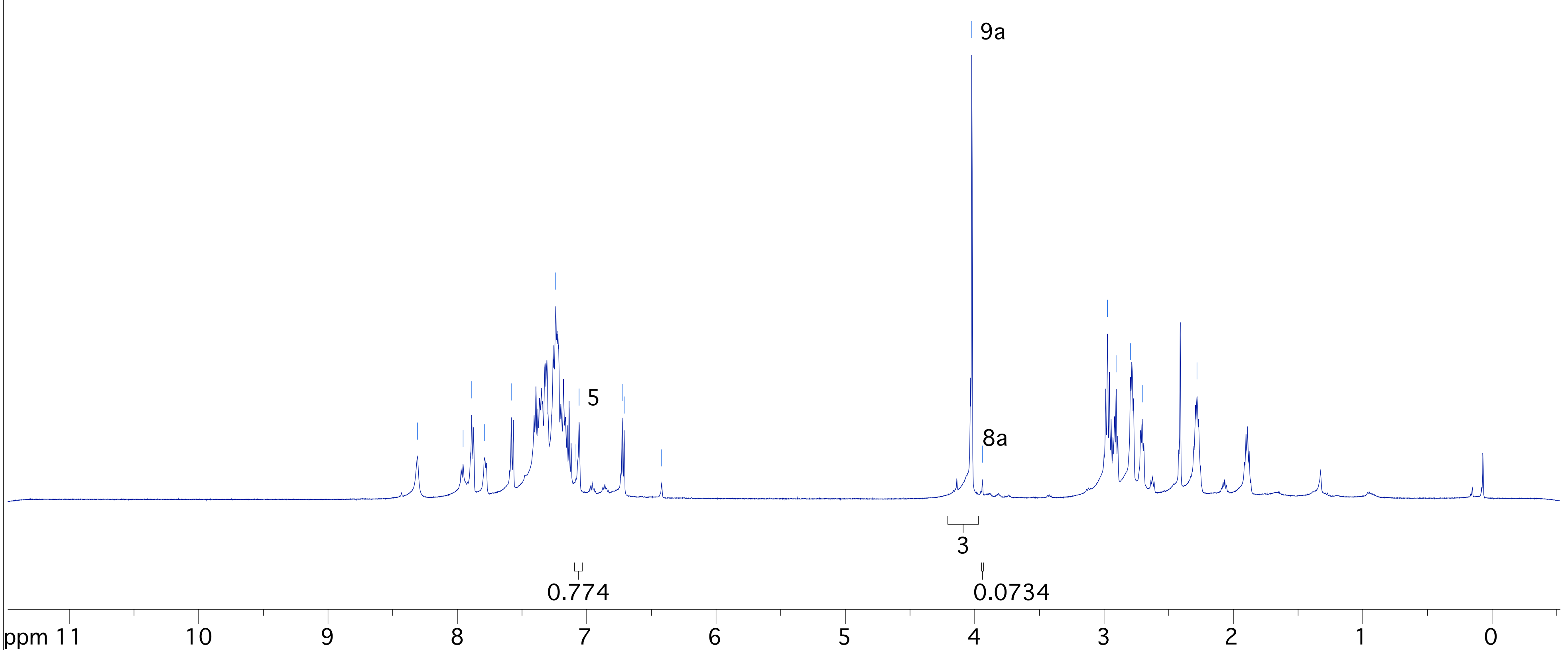

Examination of Rates of Reaction of E- and Z-Isomers

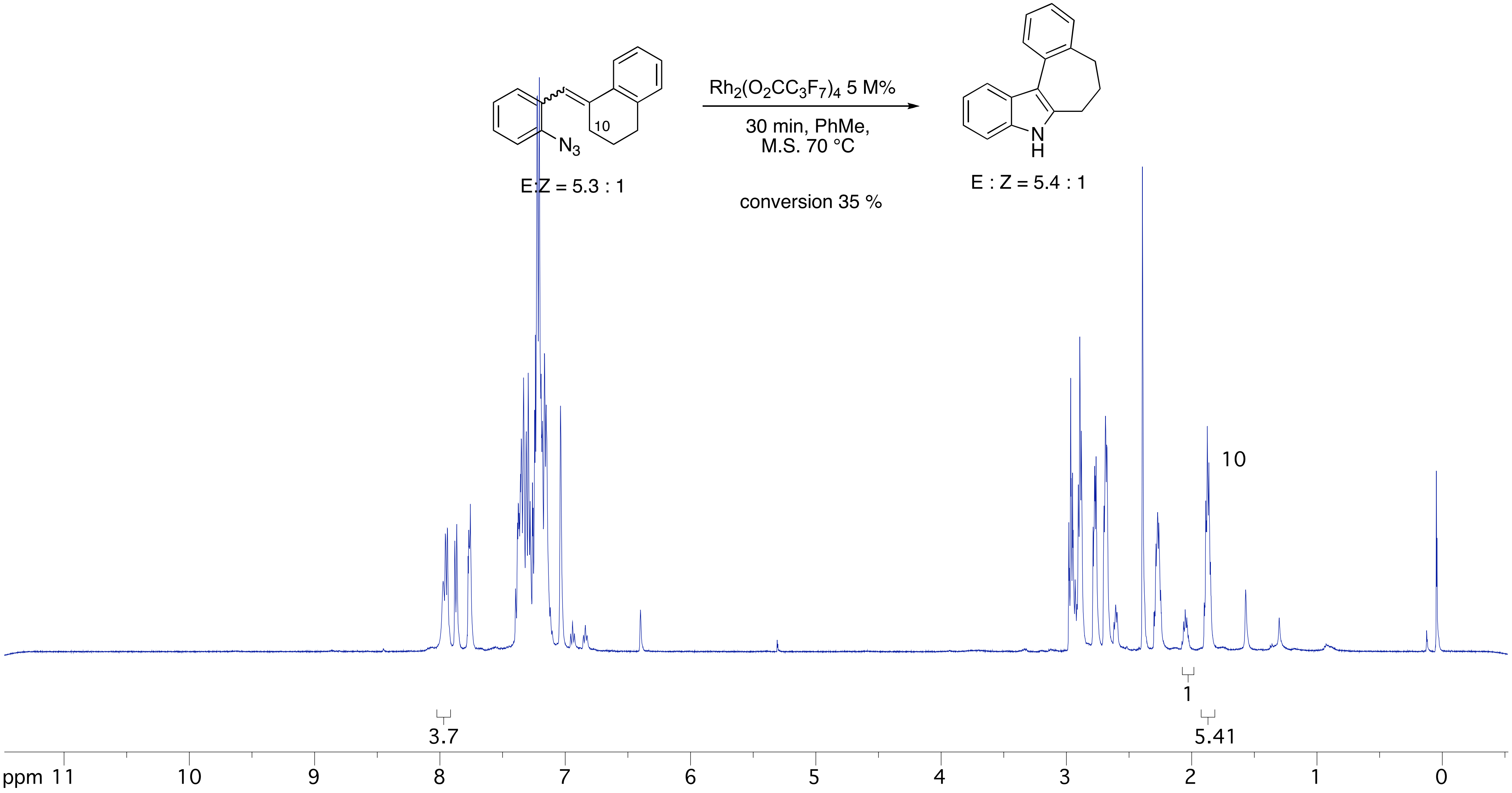

Supplement: Supplementary file 1 [file anie0050-1702-SD1.pdf]
